# Oral 8-aminoguanine against age-related retinal degeneration

**DOI:** 10.1038/s42003-025-08242-1

**Published:** 2025-05-26

**Authors:** Abhishek Vats, Yibo Xi, Amanda S. Wolf-Johnston, Owen D. Clinger, Riley K. Arbuckle, Li Sheng, Xingcan Jiang, Chase D. Dermond, Jonathan Li, Donna B. Stolz, Anthony J. St. Leger, José-Alain Sahel, Edwin K. Jackson, Lori A. Birder, Yuanyuan Chen

**Affiliations:** 1 Department of Ophthalmology, University of Pittsburgh School of Medicine, Pittsburgh, PA USA; 2 Department of Medicine, University of Pittsburgh School of Medicine, Pittsburgh, PA USA; 3 Department of Human Genetics, University of Pittsburgh School of Public Health, Pittsburgh, PA USA; 4 Department of Cell Biology, University of Pittsburgh School of Medicine, Pittsburgh, PA USA; 5 Department of Immunology, University of Pittsburgh School of Medicine, Pittsburgh, PA USA; 6 Department of Pharmacology and Chemical Biology, University of Pittsburgh School of Medicine, Pittsburgh, PA USA

**Keywords:** Pharmacodynamics, Retinal diseases, Neuroscience

## Abstract

Vision decline in the elderly, often due to retinal aging, predisposes individuals to pathologies like age-related macular degeneration. Currently, there are few effective oral treatments for this condition. Our study introduces an oral agent, 8-aminoguanine (8-AG), which targets age-related retinal degeneration using an aged Fischer 344 rat model. When administered in drinking water at a low dose for 8 weeks starting at 22 months of age, 8-AG significantly preserves retinal structure and function, as evidenced by increased retinal thickness, enhanced photoreceptor integrity, and improved electroretinogram responses. 8-AG reduces apoptosis, oxidative damage, and microglial/macrophage activation in aging retinae. 8-AG also mitigates retinal inflammation at transcriptional and cytokine levels. Extending treatment to 17 weeks further amplifies these protective effects. Given its efficacy in various disease models, 8-AG shows great promise as an anti-aging compound with the potential to mitigate common hallmarks of aging.

## Abstract (web summary)

Oral treatment of a purine metabolite, 8-aminoguanine (8-AG) protects the retinal morphology and function during aging by mitigating hallmarks of aging, highlighting its potential in treating age-related retinal degeneration.

## Introduction

Vision decline is a prevalent health issue among the elderly, influenced by both optical and neural changes within the eye. The neural retina thins significantly with age, with studies showing a 30% decrease in rods in the central retina from the third to the ninth decade of life^1,2^. Functional vision decline is marked by faster peripheral visual sensitivity loss, delayed dark adaptation, and decreased electroretinogram (ERG) a- and b-waves. Concurrently, retinal ganglion cell (RGC) loss parallels rod loss, maintaining a steady rod:RGC ratio. While foveal cone density—critical for central vision—remains stable through decades, cones are severely affected in age-related macular degeneration (AMD)^3,4^. AMD is a complex condition influenced by both environmental and genetic factors, with age being the primary non-modifiable risk factor that significantly influences disease progression. The incidence of AMD quadruples with each passing decade after 55 years of age^5^. Hence, understanding age-related retinal changes is essential to understanding aging’s contribution to AMD pathogenesis.

Age-related retinal changes also include alterations of Bruch’s membrane^6,7^, lipofuscin granule accumulation in retinal pigment epithelium (RPE)^8–10^, and chronic inflammation (para-inflammation)^11,12^. The age-related oxidative and metabolic cell stress, along with the activation of microglia and the complement pathway, may precipitate pathological conditions. Together, these changes contribute to the aging retina’s vulnerability to diseases such as AMD, highlighting the importance of understanding these mechanisms to develop potential interventions.

Numerous animal models have been developed and employed to study dry-AMD: 1) cigarette smoke models to study superoxide effects on retina and RPE, demonstrating how oxidative stress exacerbates retinal aging^13–15^; 2) the light damage models such as BALB/c mice, Fischer 344 (F344) rats^16–19^, the *Abca4*^*−/−*^*, Rdh8*^*−/−*^^20^ or the RPE65 L450M mice^21^ to study the effect of light on retinal degeneration and lipofuscin accumulation; 3) models with deficiencies in antioxidant defense mechanisms, such as the superoxide dismutase (SOD1 or SOD2)^22,23^ or nuclear factor erythroid 2-related factor 2 (NRF2)^24^ knockout, the autophagy-deficient^25,26^, and the vitamin E deficient^27^ mouse models to study oxidative stress and its impact on retinal integrity; 4) complement activation models, such as the complement factor H knockout (*Cfh*^*−/−*^)^28,29^, the humanized CFH Y402H^30^, and the C3 overexpression mouse^31^ models, to assess the role of inflammation in AMD, showcasing the interplay between immune responses and RPE damage; 5) Lipid metabolism dysfunction models, including apolipoprotein E knockout (*Apoe*^*−/−*^)^32^, humanized APO(*)E3-Leiden^33^, APOB100^34,35^, low-density lipoprotein *receptor* knockout (*Ldlr*^*−/−*^)^36^ and very low-density lipoprotein receptor knockout *(vldlr*^−/−^)^37^ mouse models, which highlight lipid metabolic contributions to AMD.

The impact of aging is studied in mice and rats^16,18,38^, revealing morphological and functional retinal changes^39^, altered epigenetic profiles^40^, mitochondrial dysfunction^40^, elevated inflammation^41,42^, and metabolic shifts^43–45^. The F344 rat is extensively used in aging research^46–51^, demonstrating progressive retinal thinning and light-induced degeneration^16–19^. Photoreceptor loss starts peripherally at 12–18 months, extending centrally by 22–24 months. Retinal degeneration occurs earlier in males than in females. Additionally, age-related RGC loss was reported in these rats, accompanied by axon loss in the optic nerve^19^. Although the F344 rat lacks a cone-dense region, it is valuable for studying age-related neuronal loss and testing treatments, making it a crucial model for understanding retinal aging processes.

**Fig. 1:**
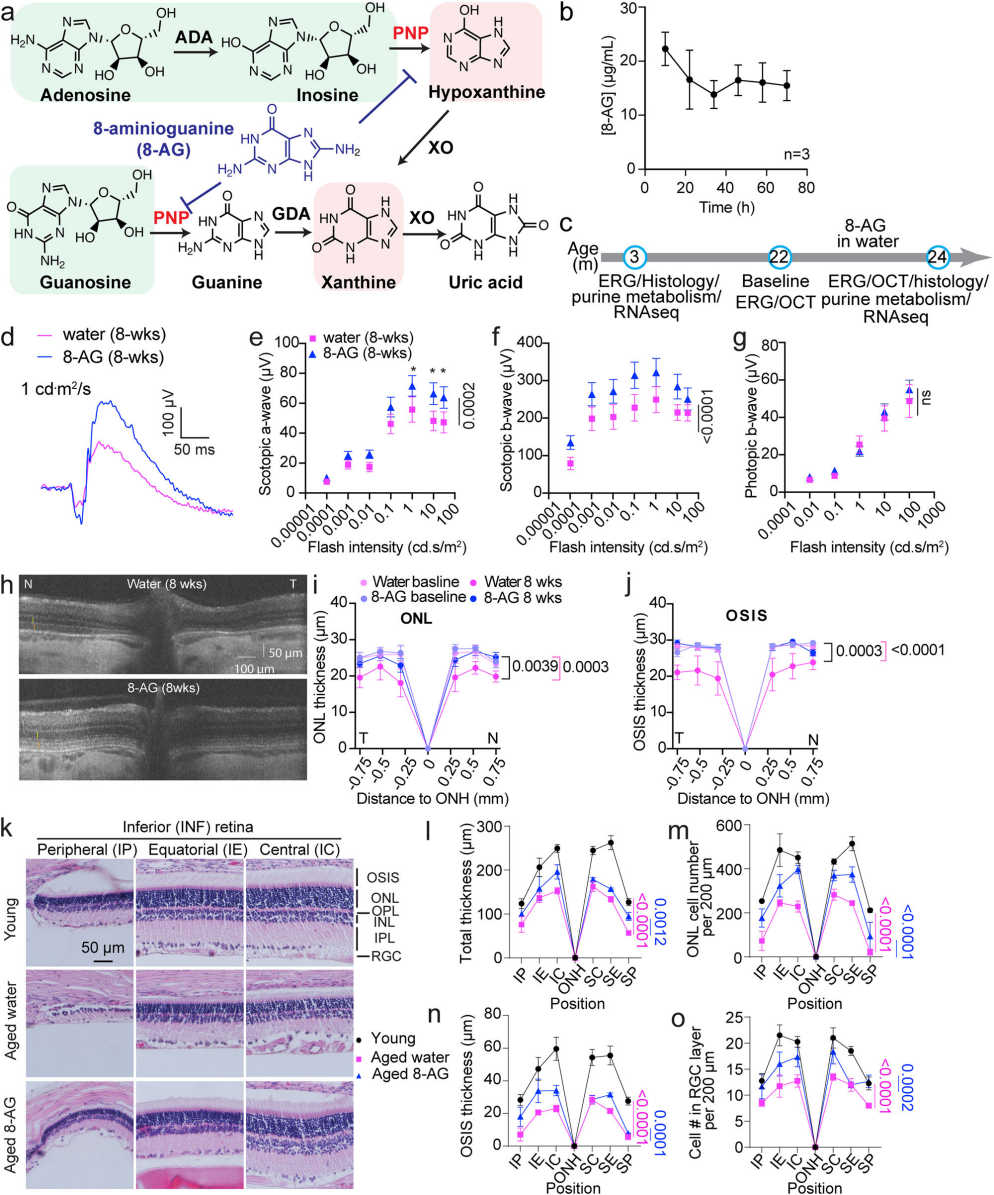
Oral 8-aminoguanine rescues the aged Fischer 344 (F344) rat retinae from degeneration. **a** An illustration shows the degradation of purine nucleosides. The tissue-protecting purines are highlighted in green, and tissue-damaging purines are marked in a pink background. The 8-AG shown in blue is an inhibitor of PNPase. **b** Stability of 8-AG in water at room temperature for 3 days using HPLC, showing only slight decay of 8-AG from 22 to 17 μg/mL in 24 h. *N* = 3. Data are shown as mean ± SD. **c** Treatment regimen and experiments using the F344 rats. Young rats were 3 months old, aged rats were 22 months old when started on a daily 8-AG supplement in water for 8 weeks. The spectral domain-optic coherence tomography (SD-OCT) and electroretinogram (ERG) were collected at baseline and endpoint. At the endpoint, the animals were euthanized for histology, purine metabolome, and RNA-seq studies. **d**–**g** are ERG results. **d** Representative scotopic ERG responses at 1 cd s/m^2^. **e**–**g** are the semi-log plots of scotopic a, b-wave and photopic b-wave responses in µV as a function of flash intensities (cd s/m^2^) for water and 8-AG treated F344 rats at the endpoint. Data from 8-AG treated and water-treated animals were shown in blue triangles and magenta squares, respectively, as means ± SEM. *N* = 8. **h**–**j** are SD-OCT results. **h** Representative B-scans of water-treated (upper panel) and 8-AG-treated (lower panel) rats at the endpoint. The vertical and horizontal scale bars are 50 and 100 µm, respectively. **i**, **j** The spidergrams of the thickness of the outer nuclear layer (ONL) and outer-inner segment layer (OSIS), respectively, measured from SD-OCT B scans. Data from the water-treated group are shown in light and dark magenta for baseline and endpoint, respectively, and those from the 8-AG-treated group are shown in light and dark blue for baseline and endpoint results, respectively. *N* = 8. Data are shown as means ± SEM. **k**–**o** are the retinal hematoxylin and eosin (H&E) staining results. **k** Representative H&E staining images at central (SC&IC), equatorial (SE&IE) and peripheral (SP&IP) regions of retinal paraffin sections on the inferior (**I**) side of optic nerve head (ONH). Scale bar, 50 μm. OS + IS, outer and inner segments; ONL outer nuclear layer, OPL outer plexiform layer, INL inner nuclear layer, IPL inner plexiform layer, RGCs retinal ganglion cells. **l**–**o** Spidergrams of measurements from H&E staining images as shown in **k**, including total thickness of all retinal layers (**l**), cell number in ONL per 200 µm length of the retinal section (**m**), thickness of OS + IS (**n**), number of cells in the retinal ganglion cells (RGC) per 200 μm length of the retinal section **o**. Data from young, water-treated aged and 8-AG-treated aged rats are shown in black circles, magenta squares, and blue triangles, respectively. *N* = 8. Statistical analysis for ERG, OCT, and histology data was performed using two-way ANOVA. The black bracket or line shows the *P* between water and 8-AG-treated groups at endpoint; the magenta bracket/line shows the *P* comparing the water-treated group at baseline and endpoint; and the blue line shows the *P* comparing the 8-AG-treated group at baseline and endpoint. ns is for non-significant. Data points and error bars are means and SEMs, respectively.

While changes in carbohydrate metabolism during aging are well-studied, purine metabolism changes have recently caught attention as well^43,52^. Purine metabolism is critical for DNA/RNA synthesis, energy-carrying molecules (ATP, GTP), signaling molecules (cAMP, cGMP), coenzymes (NAD, NADP), and purine receptor signaling. The rapid turnover of purine metabolites is particularly important for the central nervous system, including the retina^53^, due to its high energy demands. The enzyme purine nucleoside phosphorylase (PNPase) catalyzes the hydrolysis of inosine and guanosine to make hypoxanthine and guanine, respectively, which in turn are recycled back to inosine monophosphate (IMP) and guanosine monophosphate (GMP) by the hypoxanthine-guanine phosphoribosyltransferase (HGPRT)^54^, or they are catalyzed by guanine deaminase (GDA/GDH) and/or the xanthine oxidase (XO) to eventually form uric acid (Fig. 1a), pushing the purine metabolites to flow out to the blood circulation. Because XO-catalyzed reactions generate reactive oxygen species, the accumulation of hypoxanthine and xanthine may promote oxidative tissue damage. Thus, PNPase regulates the balance between inosine-guanosine (which exerts antioxidant, anti-inflammatory, and tissue-protective effects^55–61^) versus hypoxanthine-xanthine (which promotes oxidative stress). This balance may influence retinal health. Although depletion of ATP and accumulation of xanthine and uric acid are evident in brain injuries^53,62^, the role of purine changes in aging retinae is poorly understood.

Emerging evidence suggests PNPase inhibition benefits multiple tissues. For example, 8-aminoguanine (8-AG), as an endogenous PNPase inhibitor^63,64^, improves kidney function^65,66^, and attenuates high-salt induced hypertension in rat models^67^. These beneficial renal and cardiovascular effects of 8-AG are due, at least in part, to the inhibition of PNPase, leading to elevated inosine levels in the kidney^68^, which in turn activate adenosine A_2B_ receptors^68^, thereby increasing renal medullary blood flow. As recently reviewed^68^, oral administration of 8-AG prevents strokes and extends lifespan in Dahl SS rats on a high-salt diet^66^, attenuates the progression of pulmonary hypertension, and improves outcomes in animal models of diabetes and sickle cell disease. Further, the Birder laboratory demonstrated that 8-AG effectively restored the lower urinary tract to a youthful and healthier cellular, structural, and functional state in the aged F344 rats^69,70^, suggesting the anti-aging/reverse-aging effects of PNPase inhibition in the bladder and urethra.

Here, we present our findings that inhibiting PNPase with 8-AG ameliorates oxidative damage, reduces retinal immune response,s and slows the progression of retinal degeneration in this rat model of retinal aging.

## Results

### Treatment regimen

To assess 8-AG’s effect on age-related retinal degeneration, we administered 8-AG at 5 mg/kg body weight (bw) per day to 22-month-old F344 female rats via drinking water over 8 weeks, as this dose has previously been shown to be safe and efficacious in restoring LUT morphology and function^69,70^. Due to the different age of onset for retinal degeneration between males and females^17^, only female rats were used in this study. HPLC analysis showed that 8-AG remained stable in water at room temperature for up to 3 days (Fig. 1b), allowing for consistent dosing through daily water replacement with fresh 8-AG solution. We evaluated the efficacy, safety, and mechanism of action of 8-AG through ERG, spectral domain optic coherence tomography (SD-OCT), and endpoint assessments as illustrated in Fig. 1c.

### 8-AG improves the function of aged rat retinae

We recorded ERGs at baseline and 8 weeks of treatment in the aged rats (Fig. 1d–g and Supplementary Fig. S1). Substantially lower scotopic and photopic ERG responses in the 2-year-old rats, compared to young rats, revealed age-related declines in rod and cone functions (Supplementary Fig. S1a–c). Over the treatment period, water-treated aged rats showed stable scotopic a- and b-waves but displayed decreased photopic responses (Supplementary Fig. S1d–e). Conversely, 8-AG-treated aged rats showed a significant left-shift in the scotopic a-wave response curve (*P* < 0.0001, Supplementary Fig. S1g) and enhanced scotopic b-wave responses (*P* < 0.0001, Supplementary Fig. S1h), indicating improved rod photosensitivity and function during the treatment period. Photopic b-wave responses of the 8-AG-treated animals declined at rates comparable to controls (*P* < 0.001, Supplementary Fig. S1f, i). At the endpoint, scotopic a- and b-wave responses were significantly higher in 8-AG-treated animals compared to water-treated controls (*P* = 0.0002 and <0.0001; Fig. 1d–f), whereas photopic responses showed no notable differences (Fig. 1g). These findings indicate that 8-AG treatment enhances rod function in aged F344 rats without substantially affecting cone function.

### 8-AG preserved the structure of aged rat retinae

We then used SD-OCT to quantify the retinal morphology changes (Fig. 1h–j and Supplementary Data File 1). In the control group, a significant reduction in the thickness of the outer nuclear layer (ONL) and the outer+inner segment (OSIS) was observed between baseline and endpoint, indicating retinal degeneration occurring between 22 and 24 months of age. In contrast, the 8-AG-treated group maintained stable ONL and OSIS thickness throughout the treatment period, demonstrating that 8-AG effectively halts the aged retinae from degeneration.

At the endpoint, we conducted retinal histological analysis (Fig. 1k–o and Supplementary Data File 2). Compared to young retinae, water-treated aged retinae exhibited significant age-related thinning, reduced ONL, thinner OSIS layers, and fewer nuclei in the RGC layer. In contrast, 8-AG-treated animals displayed significantly thicker retinae (*P* < 0.01), higher ONL cell counts (*P* < 0.0001), thicker OSIS layers (*P* < 0.001), and more cell counts in the RGC layer (*P* < 0.001) compared to water-treated controls (Fig. 1k–o). Notably, 8-AG preserved the ONL at the peripheral ends, a region where photoreceptor layers were completely absent in some control retinae (Fig. 1k).

### 8-AG reduces cell death and apoptosis in aged rat retinae

**Fig. 2:**
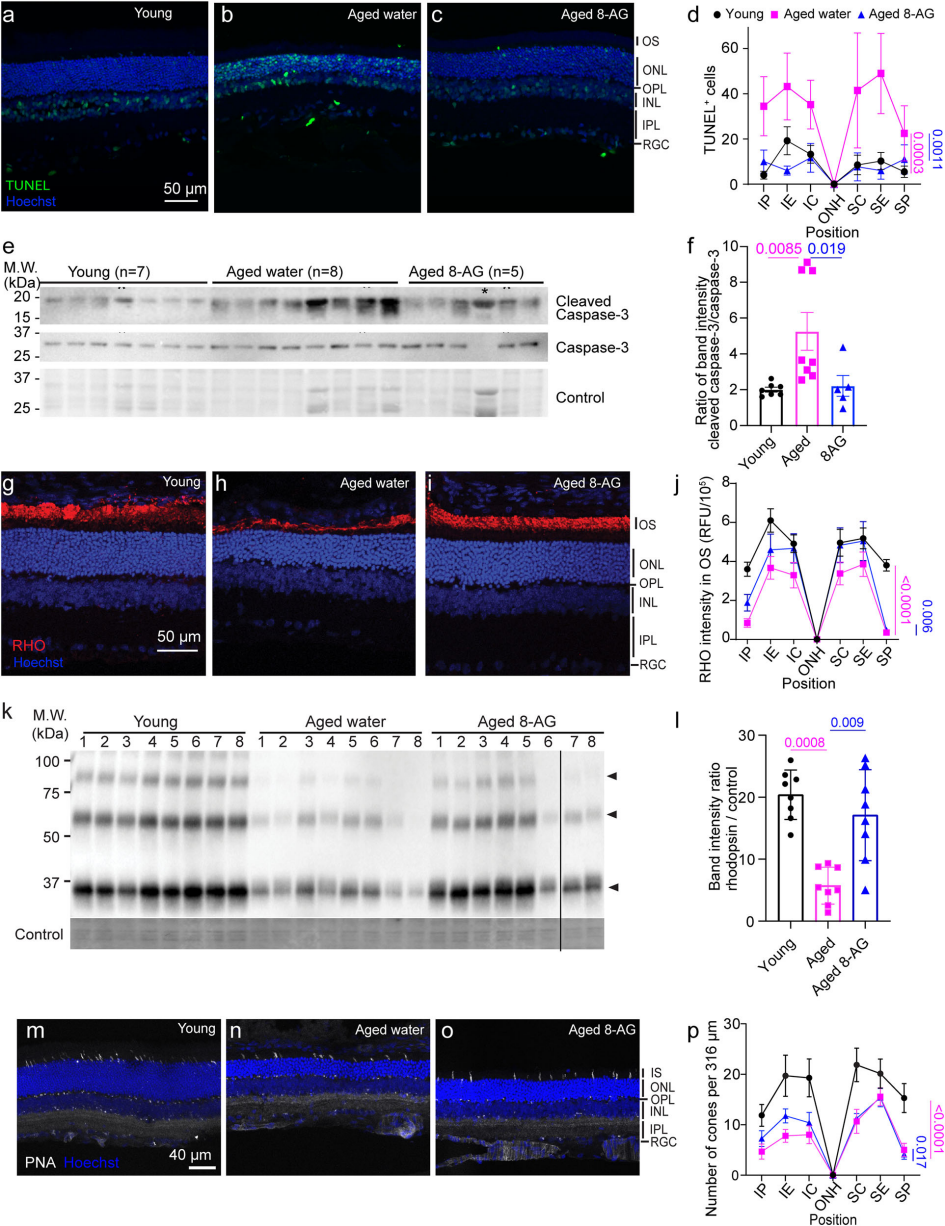
8-AG treatment reduces apoptosis and protects photoreceptors in aged F344 rats. The young (3 months), water-treated aged (24 months), or aged F344 rats treated with 8-AG for 8 weeks (24 months) were euthanized, and eyes were enucleated and prepared for immunohistochemistry or immunoblots. **a**–**c** are representative TUNEL-stained immunofluorescence images for young (**a**), water-treated aged (**b**), and 8-AG-treated aged rats (**c**), respectively, at the superior equatorial region of the retina sections, TUNEL^+^ cells are in green, and nuclei are stained with Hoechst33b342 in blue. Scale bar, 50 μm. **d** The Spidergrams of TUNEL^+^ cell numbers at different positions of the retinae. *N* = 3-4. **e** Immunoblots against the cleaved (top, 17, and 19 kDs) and intact caspase-3 (middle, 30 kDa) from rat retinal lysates. Ponceau stain (bottom) was used as the loading control. Each lane represents one rat retina. * Marks the excluded sample due to abnormal Ponceau stain. **f**. Bar plot of the band intensity ratio of cleaved: intact caspase-3. *N* = 7, 8, and 5 for young, water-treated aged, and 8-AG-treated aged F344 rat retinae. Data and error bars are means ± SDs. **g**–**i** Immunofluorescence images of rhodopsin (RHO, red) and Hoechst33342 for nucleus (blue) at superior central (SC) region of retinal cryosections of young (**g**), aged water-treated (**h**), and aged 8-AG-treated rats **i**. Scale bar, 50 μm. **j** The spidergram of RHO immunofluorescence intensity in the OS at different positions of the retinae. *N* = 8, 8, and 7 for young, water-treated aged, and 8-AG-treated-aged group, respectively. **k** Immunoblot of RHO from rat retinae. Each number represents one rat retina. Arrowheads show tetramer, dimer, and monomer of RHO from top to bottom, respectively. **l** Bar graph of RHO immunoblot intensity normalized by loading control. *N* = 8. **m**–**o**. Immunofluorescence images of peanut agglutinin (PNA, a marker of cones) and Hoechst33342 for nucleus (blue) at inferior central regions of retinal cryosections of young (m), aged water-treated (**n**) and aged 8-AG treated rats (**o)**. Scale bar, 40 μm. *N* = 8. **p** The spidergram of PNA+ cone numbers at different positions of the retinae. Black circles, young rats; magenta squares, aged rats; blue triangles, aged rats treated with 8-AG. For spidergrams, data points and error bars are means and SEMs, and *P* values were calculated by two-way ANOVA. For the bar graph, column, and error bars are means and SDs, and *P* values were calculated by Kruskal–Wallis. Pink lines show *P* comparing the water-treated aged vs. young control, and blue lines show *P* comparing the 8-AG-treated aged vs. water-treated aged control.

We used the terminal deoxynucleotidyl transferase dUTP nick end labeling (TUNEL) assay and caspase-3 cleavage assay (Fig. 2a–f) to assess retinal cell death. Aged retinae exhibited more than double the number of TUNEL+ cells compared to young retinae (*P* < 0.001). However, 8-AG treatment reduced TUNEL+ cells in aged retinae to levels comparable to those in young retinae (Fig. 2a–d and Supplementary Data File 3, *P* < 0.01 comparing 8-AG to aged controls). Caspase-3 cleavage, a key apoptotic event triggering a cascade of programmed cell death^71^, was also analyzed. Immunoblotting showed aging doubled the cleaved-to-intact caspase-3 ratio (*P* < 0.01); 8-AG treatment reduced this ratio by 50% (Fig. 2e, f and Supplementary Data File 4, *P* < 0.05), indicating decreased age-related apoptosis. These findings suggest that oral 8-AG is safe and decreases cell death in aged retinae.

### 8-AG rescues rhodopsin level in rod photoreceptors and preserves inferior cones

Given that 8-AG enhanced rod function (Fig. 1d–g and Supplementary Fig. S1) and preserved OSIS layer lengths (Fig. 1d, n) in aged F344 rat retinae, we examined its effect on rhodopsin (RHO), the key phototransduction protein in rods (Fig. 2g–l). RHO levels decreased considerably in the aged retinae compared to young controls, shown by both immunohistochemistry (IHC) (Fig. 2g, h and Supplementary Data File 5, *P* < 0.0001) and immunoblots (Fig. 2k, l and Supplementary Data File 4, over 3-fold decrease, *P* < 0.01). 8-AG treatment significantly restored RHO levels (over 2-fold increase, *P* < 0.01, <0.05 in Fig. 2i, j–l, respectively). However, it did not affect other phototransduction components, such as ARRESTIN1 and phosphodiesterase 6B (PDE6B, Supplementary Fig. S2 and Supplementary Data File 6). These results suggest that 8-AG rescues the rods from a dormant state to a healthier, functional state by restoring OSIS length, RHO levels, and rod function.

While 8-AG didn’t improve cone function (Fig. 1g), it significantly preserved the number of cones in the inferior retina of aged rats (*P* < 0.0001, Fig. 2m–p and Supplementary Data File 7), suggesting a protective effect on cones.

### Oral 8-AG reduces oxidative damage in F344 rats

**Fig. 3:**
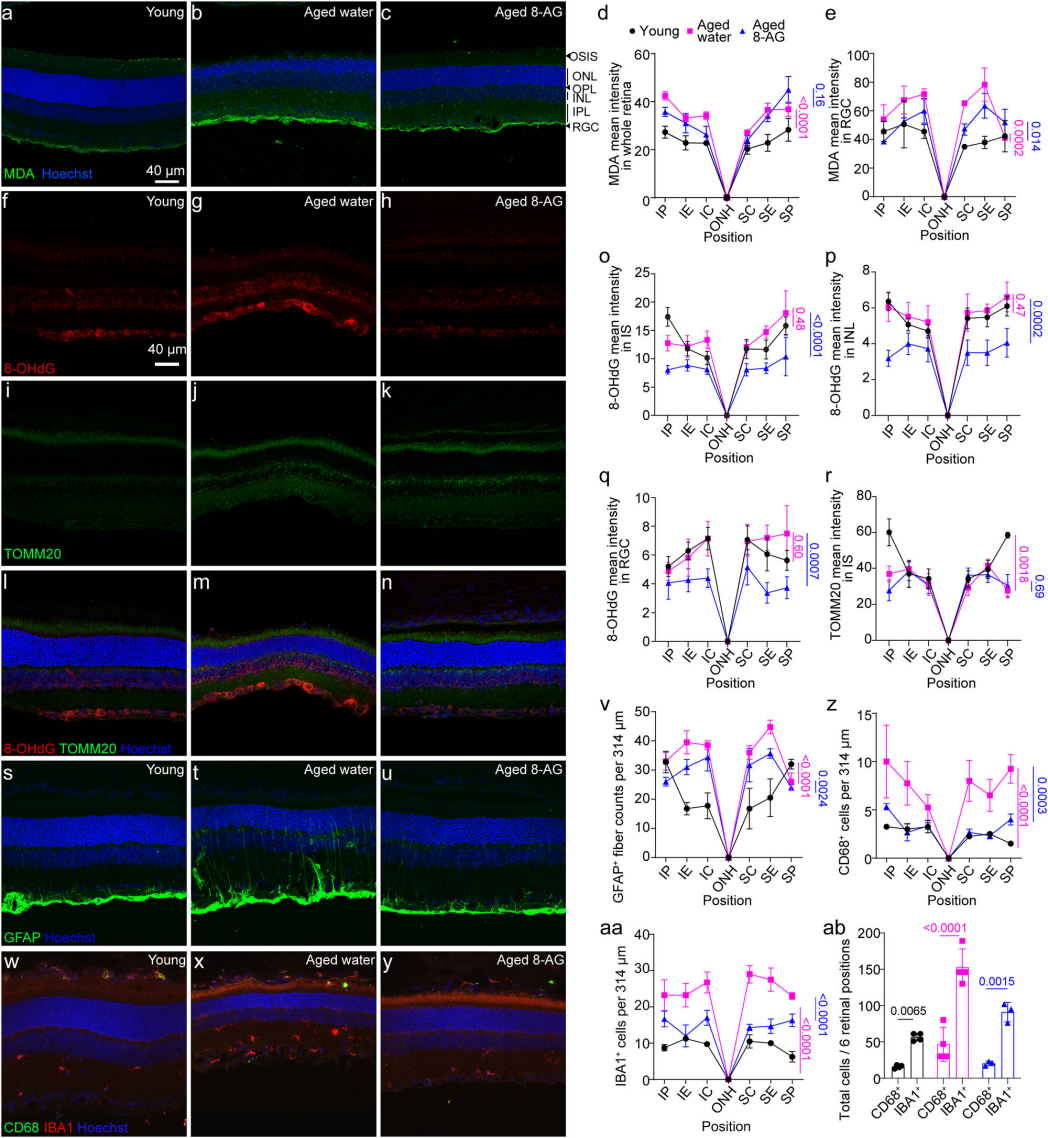
8-AG reduces retinal tissue oxidation and activation of Müller and microglia in aged F344 rat retinae. Twenty-two-month-old F344 rats were treated with 8-AG in water or water only for 8 weeks. The 3-month-old young rats are used as controls. The 24-month-old rats were euthanized, and eyes were isolated for immunohistochemistry against tissue oxidation and stress markers. **a**–**c** Representative immunofluorescence images of malondialdehyde (MDA, in green), an indicator of lipid peroxidation, at superior central positions of the retinae from young (**a**), aged water treated (**b**), and aged 8-AG treated rats (**c**). Hoechst33342 stained the nucleus blue. Scale bar, 40 µm. **d** and **e** Spidergrams of MDA intensity in the whole retina and in retinal ganglion cell layer (RGC), respectively. *N* = 3. **f**–**n** Representative immunofluorescence images of the 8-hydroxy-2’ -deoxyguanosine (8-OHdG, a marker for oxidated DNA in red) (**f**–**h**), translocase of outer mitochondrial membrane 20 (TOMM20, a mitochondrial marker in green) (**i**–**k**), and merged channel with Hoechst33342 (blue) (**l**–**n**) on the superior central region of the retinal cryosections of young (**f**, **i**, **l**), water-treated aged (**g**, **j**, **m**) and 8-AG-treated aged rats (**h**, **k**, **n**). **o**–**q** Spidergrams of immunofluorescence intensity of 8-OHdG on the inner segment layer (IS) (**o**), inner nuclear layer (INL) (**p**), and RGC layer (**q**), respectively. **r** The spidergram of immunofluorescence intensity of TOMM20 in IS. *N* = 4, 5, 4 for young, water-treated aged, and 8-AG-treated aged groups, respectively. **s**–**u** Representative immunostaining images of glial fibrillary acidic protein (GFAP), as a marker of activated Müller glia in green, for young (**s**), water-treated aged (**t**), and 8-AG-treated aged rat retina (**u**). **v** The spidergram of GFAP+ fiber counts per 314 μm at different locations of retinae. *N* = 4, 4, 3 for young, water-treated aged, and 8-AG-treated aged group, respectively. **w**–**y** Representative immunostaining images of the cluster of differentiation 68 (CD68, in green), ionized calcium-binding adaptor molecule 1 (IBA1, in red), both are markers of microglia/macrophages, from young (**w**), aged water treated (**x**), an aged 8-AG treated (**y**) at different retinal positions. Hoechst33342 stained nucleus in blue. Scale bar, 50 μm. **z** and **aa** are spidergrams of the counts of CD68+ cells (**z**), and IBA1+ cells (**aa**) per 314 μm at different retina positions. **ab** The bar plot of the total CD68+ and IBA1+ cells at six positions of the retinal cross-sections. Black circles, young group; magenta squares, water-treated aged group; blue triangles, 8-AG-treated aged group. *N* = 4, 4, 3 for young, water-treated aged, and 8-AG-treated aged group, respectively. Data points and error bars are means ± SEMs in the spidergrams, and means ± SDs in the bar graph. *P* values were analyzed by two-way ANOVA for the spidergrams and Kruskal–Wallis for the bar graph. Pink line show *P* comparing the water-treated aged vs. young control, and blue lines show *P* comparing the 8-AG-treated aged vs. water-treated aged control.

To examine aging-associated oxidative damage, we assessed malondialdehyde (MDA) for lipid oxidation, 8-hydroxy-2’-deoxyguanosine (8-OHdG) for DNA oxidation^72,73^, and translocase of outer mitochondrial membrane 20 (TOMM20) a mitochondria marker in young (3 m), water-treated aged (24 m) and 8-AG treated aged F344 rat retinae (Fig. 3a–r). Aged retinae showed increased MDA levels, particularly in the RGC layer (Fig. 3a–e and Supplementary Data File 8, *P* < 0.0001). While 8-AG did not reduce overall retinal MDA levels, it significantly decreased MDA in the RGC layer (*P* < 0.05), indicating RGC-specific protection from lipid peroxidation. Other retinal layers, including the photoreceptor OS, showed no significant change, likely due to the OS’s renewal by RPE cells^74^.

8-AG also reduced DNA oxidation, as indicated by lower 8-OHdG levels in the IS, INL, and RGC layers of treated aged retinae (Fig. 3f–h, o–q and Supplementary Data File 9). No changes in TOMM20 levels were observed (Fig. 3i–n, r and Supplementary Data File 9). These findings suggest that 8-AG exerts strong antioxidant effects in the neural retina, protecting RGCs and reducing DNA oxidation in photoreceptor mitochondria and inner retinal neurons. Interestingly, the ONL was free of 8-OHdG staining, suggesting minimal chromosomal DNA damage in photoreceptors, possibly due to their condensed chromatin.

### 8-AG treatment reduces the injury-induced activation of Müller glia and microglia in rat retina

Müller glia and microglia are activated in response to retinal neuron stress^75^. Immunostaining for glial fibrillary acidic protein (GFAP) as a stress marker in Müller glia, and cluster of Differentiation 68 (CD68) and ionized calcium-binding adapter molecule 1 (IBA1) as markers of microglia/macrophages in F344 rat retinae (Fig. 3s–ab) showed increased GFAP+ filaments in aged retinae, which 8-AG significantly reduced in central and equatorial regions (*P* < 0.01), though not in the degenerated periphery (Fig. 3s–v and Supplementary Data File 10). 8-AG also decreased elevated CD68+ and IBA1+ cells in aged retinae (*P* < 0.0001, Fig. 3w–ab and Supplementary Data File 11), demonstrating anti-inflammatory effects. Notably, all CD68+ cells were IBA1, but not vice versa, indicating distinct macrophage subpopulations. These findings suggest 8-AG mitigates age-related retinal inflammation by reducing Müller glial activation and microglial responses.

### 8-AG reduces the age-related accumulation of phagosomes

**Fig. 4:**
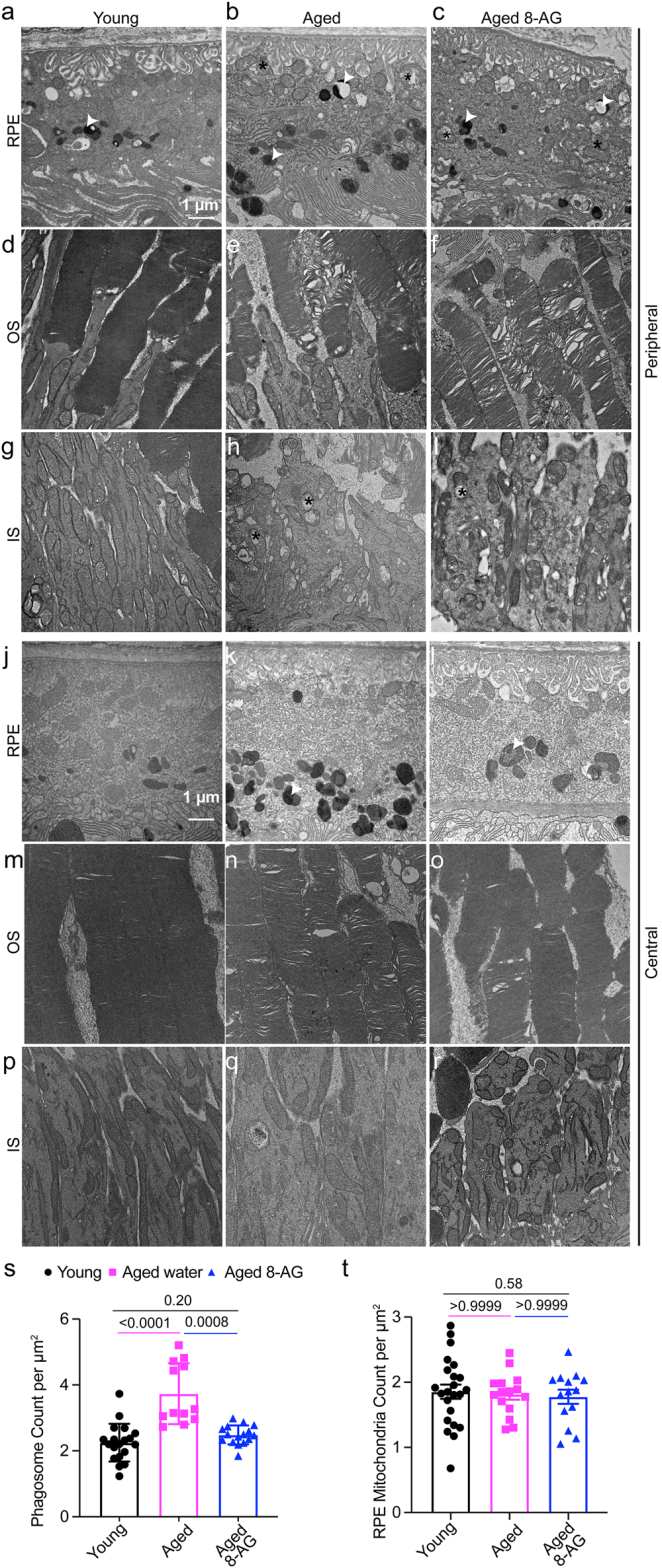
8-AG reduces the number of phagosomes in aged F344 rat retina. Young (3 m), water-treated aged (24 m), or 8-AG-treated aged F344 rats (24 m) were euthanized, and eyes were isolated and processed for transmission electron microscopy (TEM). **a**–**r** Representative TEM images of young, aged, and aged 8-AG treated rat’s RPE (**a**–**c** and **j**–**l**), photoreceptor’s outer segment (OS) disc membrane (**d**–**f** and **m**–**o**) and inner segment (IS) (**g**–**I** and **p**–**r**) at peripheral (**a**–**i**) and central region (**j**–**r**) of the retinae. **a**–**c** and **j**–**l** TEM images of RPE from the peripheral and central region, respectively, showing autophagosome (white arrowhead) and mitochondria (black asterisk). Scale bar, 1 µm. **d**–**f** and **m**–**o**. TEM images showing the integrity of photoreceptors OS disc membrane at the peripheral and central retina, respectively. **g**–**i** and **p**–**r** TEM images of mitochondria (black asterisk) in the photoreceptor’s IS of the peripheral and central region of rat’s retina, respectively. **s**, **t** Bar graphs represent the number of phagosomes and mitochondria, respectively, in the RPE at the peripheral region of young (black circle), aged (magenta square), and aged 8-AG treated (blue triangle) rat’s retina. *N* = 6–7. Each data point is from one TEM image. Data points and error bars are means ± SD, and *P* values were analyzed by Kruskal–Wallis.

Transmission electron microscopy (TEM) of retinal cross-sections from young, aged, and 8-AG-treated aged rats (Fig. 4, Supplementary Data File 12) revealed more severe structural damage in the peripheral retina of aged rats, including swollen mitochondria (*), electron-dense phagosomes (white arrowheads), disorganized photoreceptor OS membranes, and fragmented IS mitochondria (Fig. 4a–r). IS diameter increased with age, while OS diameter remained stable (Fig. 4g, h, p, q; 4d, e, m, n). 8-AG significantly reduced phagosome accumulation in the RPE (Fig. 4b, c, k, l, s) but had no effect on the number or morphology of mitochondria in RPE or IS (Fig. 4t). These findings suggest 8-AG reduced aging-related RPE phagosome accumulation.

### The transcriptome of 8-AG-treated retina shows downregulation of immune and stress responses

**Fig. 5:**
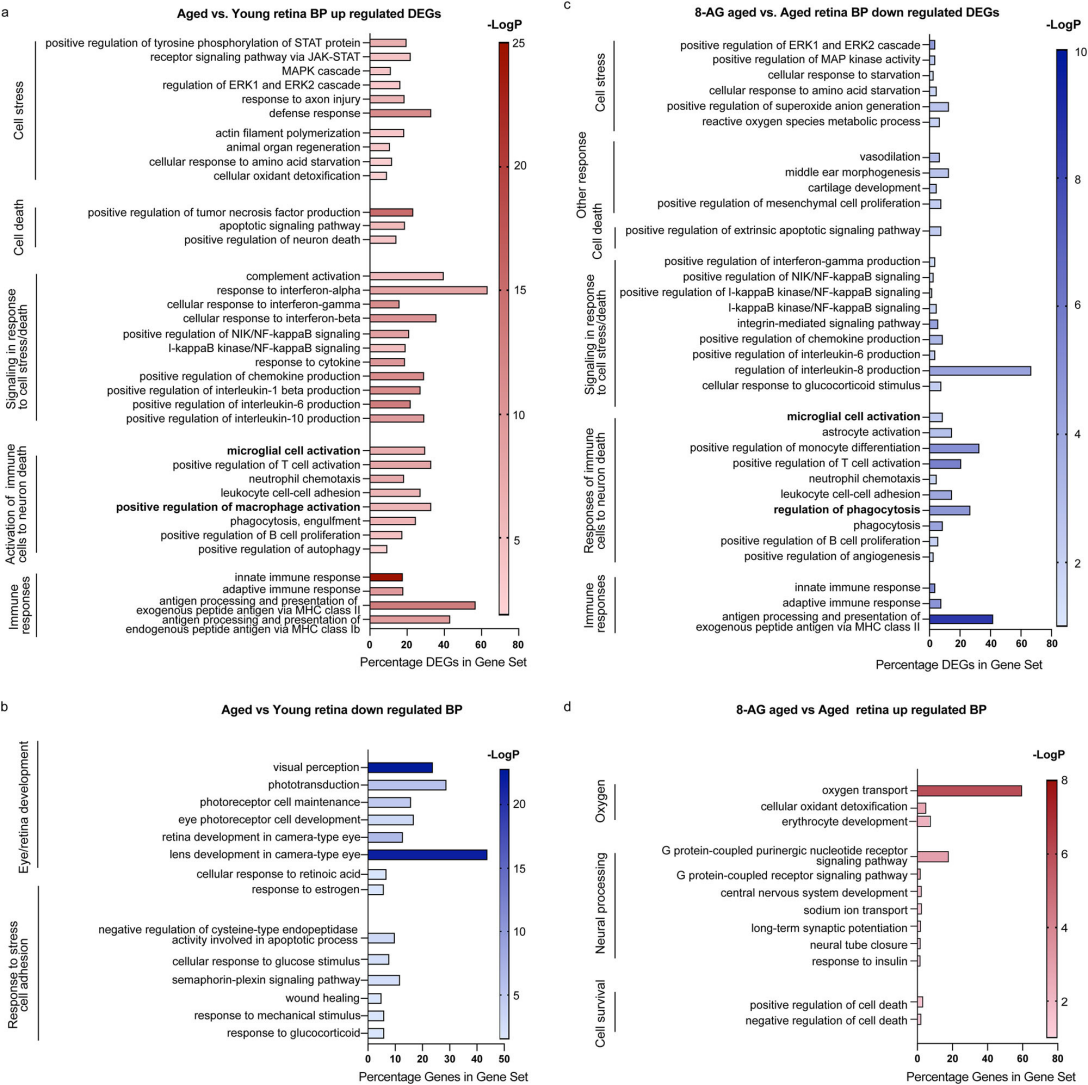
Transcriptomics reveal reduced immune and stress responses in 8-AG-treated rats. Young, aged, and 8-AG-treated aged rats’ whole retinae were processed for transcriptomic analysis using bulk RNA-seq. The differentially expressed genes (DEGs) among aged untreated vs. young and aged 8-AG treated vs. aged untreated were identified which showed >1.5-fold changes with *P* values of <0.05. Significantly affected biological processes were identified by gene ontology (GO) analysis. *N* = 3. **a**, **b** are the biological pathways (BPs) of upregulated and downregulated DEGs, respectively, in the aged untreated vs. young rat’s whole retina. **c**, **d** are the BPs of downregulated and upregulated DEGs, respectively, in the aged 8-AG-treated vs. aged rat’s whole retina. *X* axis represents the percentage of genes in a gene set of certain BPs and the *Y* axis represents the BPs. On the right side, the labeling of all graphs lists the cellular pathways.

To explore the molecular changes caused by aging and 8-AG treatment, we performed bulk RNA-Seq on retinae and RPE/choroid from young, water-treated aged and 8-AG-treated aged rats (Supplementary Fig. S3, Supplementary Data Files 13–16, and GEO accession number GSE254123). Aged retinae showed 293 upregulated and 814 downregulated differentially expressed genes (DEGs), compared to the young control (*P* < 0.05, >1.5-fold, Supplementary Data File 13). Gene ontology (GO) analysis revealed upregulated stress responses, including the Janus kinase-signal transducer and activator of transcription (JAK-STAT), mitogen-activated protein kinase (MAPK), extracellular signal-regulated kinase (ERK) cascades, axon injury; and pro-inflammatory signaling including interferons, nuclear factor kappa-light-chain-enhancer of activated B cells (NF-κB), and cytokines such as interleukin-1 β (*Il–1β*), *Il-6*, and *Il-10*. Pathways for immune cell activation (microglia, macrophages, T cells, neutrophils, and B cells) and complement system components (*C1*, *C3*, *C4a/b*, and complement factor H/*Cfh*) were also elevated, supporting an inflammatory environment as observed in AMD. Markers of activated microglia (*Cd68*, allograft inflammatory factor 1/*Aif1*) and Müller glia (*Gfap*) were similarly upregulated. Downregulated DEGs included pathways linked to retinal development, blood-retinal barrier integrity, and retinal function, suggesting a compromised retinal blood barrier and reduced retinal function (Fig. 5a, b and Supplementary Data File 13).

8-AG treatment reversed many aging-related changes, resulting in 80 downregulated and 87 upregulated DEGs (Supplementary Data File 14). Key stress-response pathways, including ERK/MAPK signaling and pro-inflammatory signaling (e.g., interferon-gamma, NF-κB, and chemokine production), were downregulated (Fig. 5c). Immune cell activation, phagocytosis, and angiogenesis pathways were also downregulated. Upregulated DEGs were associated with oxygen transport, neural processing, and anti-inflammatory effects, including neuroprotective transcripts such as growth hormone-releasing hormone receptor (*Ghrhr*)^76,77^, G-protein-coupled receptor 171 (*Gpr171*)^78^, and microRNA 124 (*Mir-124*)^79,80^ (Fig. 5d and Supplementary Data File 14). Collectively, these transcriptomic changes suggest that 8-AG reduces cell stress, suppresses inflammation, and supports retinal protection.

### The transcriptome of RPE/choroids suggests an age-related weakening of tight junctions and upregulated inflammatory signals, partially reversed by 8-AG

**Fig. 6:**
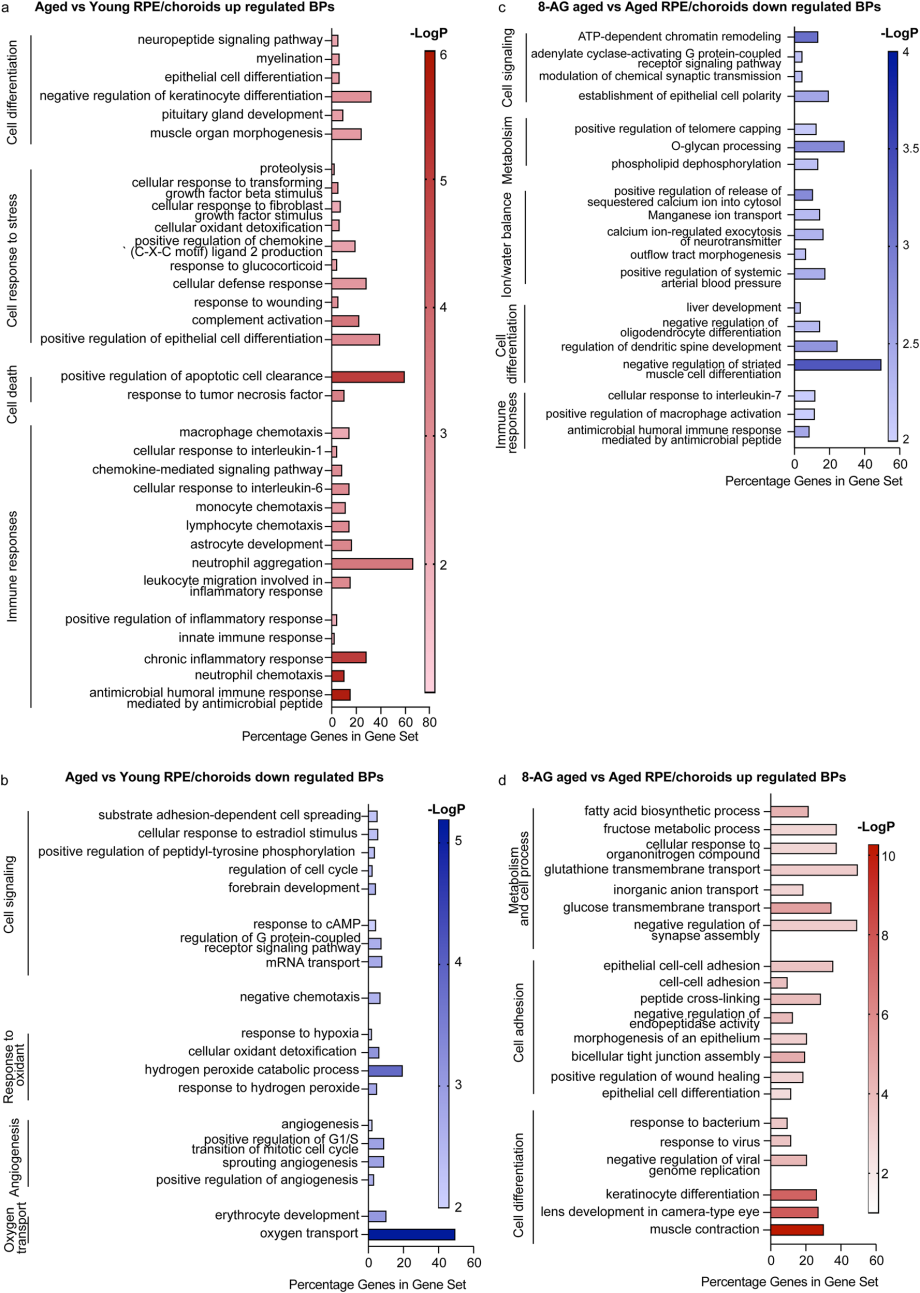
Transcriptomics of RPE/choroid reveals partial recovery of declined tight junctions and attenuation of upregulated inflammatory signals in 8-AG-treated rats. RPE/choroids of young, aged, and 8-AG-treated aged rats were manually separated from the retinae were processed for transcriptomic analysis using bulk RNA-seq. Aged untreated vs. young and aged 8-AG treated vs. aged untreated DEGs were analyzed by GO to identify affected biological pathways (BPs). DEGs were identified with >1.5-fold changes with *P* values < 0.05. *N* = 3. **a**, **b** are up- and downregulated BPs, respectively, in RPEs/choroids from aged untreated vs. young rats. **c**, **d** are up and downregulated BPs, respectively, in RPEs/choroids in aged 8-AG-treated vs. aged untreated rats. The *X* axis represents the DEGs percentage in a gene set, and the *Y* axis represents the BPs. Right side of the graphs, shows the corresponding cellular pathways.

RPE/choroid tissue identity was confirmed by the enrichment of RPE-specific mRNAs, including *Cst3*, *Efemp1*, *Itgav*, *Crispld1*, *Itgb8*, *Gulp1*, *Rpe65*, *Best1*, *Rbp1*, *Rlbp1*, *Rgr*, *Lrat*, *Pmel*, *Tttr*, *Tyr*, *Tyrp1*, and *Ptgds* (Supplementary Data Files 15 and 16)^81^. Comparing aged vs. young rat RPE transcriptomes, 166 upregulated and 118 downregulated DEGs were identified (Supplementary Data File 15). Upregulated DEGs in aged RPE included immune-related pathways, such as complement activation (*C3*, *C4b*, *Cfi*), antimicrobial responses (*Camp*, *Ccl27*, *Cxcl1*), and tumor necrosis factor signaling (*Ccl2*, *Chi3l1*). Astrocyte markers (*Gfap*, *S100a8*) and TGF-β-responsive genes (*Cdkn2b*, *Wnt10a*) were also elevated (Fig. 6a). Downregulated DEGs included pathways for hydrogen peroxide catabolism (*Hba-a2*, *Pxdn*), angiogenesis (*Angpt2*, *Tek*), chemotaxis (*Itgb3*, *Sema3g*), and response to hypoxia (*Adam17*, *Angpt2*). These changes suggest compromised tight junction integrity and reduced anti-inflammatory responses in aged RPE/choroid tissues (Fig. 6b, Supplementary Data File 15).

8-AG treatment downregulated 140 genes and upregulated 519 genes in aged RPE, relative to controls (Supplementary Data File 16). Downregulated DEGs indicate suppression of pathways related to cell signaling, metabolism, ion/water homeostasis, cell differentiation, and immune responses. Specifically, immune pathways such as responses to IL-7, macrophage activation, and antimicrobial humoral responses were reduced, reversing aging-related immune activation (Fig. 6c). Downregulation of ion homeostasis pathways suggests effects on water balance and blood pressure, consistent with 8-AG’s known roles in modulating these processes in the kidney^82^. Upregulated genes were associated with metabolism, cell adhesion, and differentiation (Fig. 6d), including tight junction proteins (*Cldn2*, *Cldn3*, *Cldn4*, *Cldn7*, *Cldn19*, and *Cldn23*), which are critical for RPE integrity. Notably, *Cldn19*, predominantly expressed in RPE, was strongly upregulated by 8-AG^83,84^. These findings suggest 8-AG may restore the RPE and choroid capillary junction integrity, enhancing the blood-retinal barrier.

### 8-AG treatment reduces the expression of pro-inflammatory genes and reduces the pro-inflammatory cytokine levels in the aged F344 retinae

**Fig. 7:**
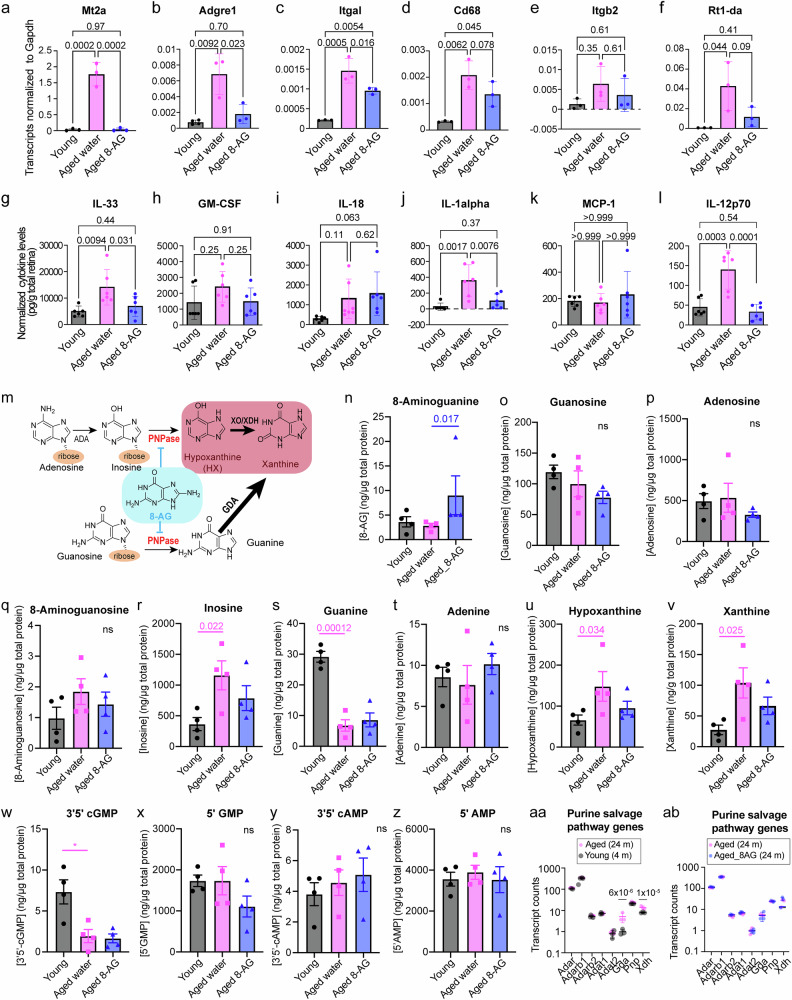
8-AG reduces the expression of pro-inflammation genes, reduces the levels of pro-inflammatory cytokines, and affects the purine metabolome in F344 rat retinae. Retina from young, water-treated aged, and 8-AG-treated aged rats were harvested, retinal lysates were used for qPCR, multiplex cytokine profiling using a flow-cytometer, and purine metabolome quantification using UPLC-MS/MS. **a**–**f** Transcript levels in the retinae for the following genes normalized with *Gapdh* level. *N* = 3. **a** *Mt2a*, **b** *Adgre1*, **c** *Itgal*, **d** *Cd68*, **e** *Itgb2*, and **f** *Rt1-da*. **g**–**l** Retinal cytokine/chemokine levels normalized by net retina weight and calibrated by standard curves. *N* = 6. **g** IL-33, **h** GM-CSF, **i** IL-28, **j** IL-1-alpha, **k** MCP-1, and **l** IL12p-70 (the 70 kDa complex of IL-12). **m** A brief illustration reminding the salvage and degradation of purine nucleosides. The tissue-damaging purines are in red background; 8-aminoguanine (8-AG) shown in light blue background, is a PNPase inhibitor. **n**–**z** Concentrations of retinal purine metabolites from young (black circle), water-treated aged (magenta square), and 8-AG-treated aged rats (blue triangle), normalized with the total protein in micrograms (ng of metabolite/µg of total protein). *N* = 4. **n** 8-AG; **o** Guanosine; **p** Adenosine; **q** Inosine; **r** 8-Aminoguanosine; **s** Guanine; **t** Adenine; **u** Hypoxanthine; **v** Xanthine; **w** 3’5’-cGMP; **x** 5’-GMP; **y** 3’5’-cAMP; **z** 5’-AMP. **aa** Dot plot of transcripts involved in the purine salvage and degradation pathway from young (black) and aged (magenta) rat retinae, using RNA-seq data. **b** Dot plot of transcripts involved in the purine salvage and degradation pathway from aged (magenta) and 8-AG-treated aged (blue) rat retinae, using the RNA-seq data. *N* = 3. Data and error bars are means±SDs. *P* values were calculated using One-way ANOVA for all the plots. ns stands for no significance.

To validate the retinal inflammation in aging and 8-AG’s anti-inflammatory effects, we performed the real-time PCR to quantify six pro-inflammatory genes and a 13 cytokine/chemokine rat immune panel assay using the F344 rat retinal lysates (Fig. 7a–l). Consistent with the retinal RNA-seq data, the transcript levels of *Mt2a* (metallothienine2A, an interferon-stimulated gene), *Adgre1* (adhesion G-protein-coupled receptor E1, or F4/80, a marker of macrophage), *Itgal* (integrin alpha L chain, also known as Cd11a, a leukocyte co-stimulatory factor), were significantly increased in the aged rat retinae and reduced by 8-AG treatment (Fig. 7a–c), and *Cd68* (encoding a macrophage lysosomal glycoprotein), *Itgb2* (integrin β2) and *Rt1-da* (encoding a Major Histocompatibility Complex class II/MHC-II molecule) showed age-related increases that were mitigated by 8-AG (Fig. 7d–f). Of the thirteen cytokine/chemokines tested, six were detected in the retina, including IL-33, granulocyte-macrophage colony-stimulating factor (GM-CSF), IL-18, IL-1α, monocyte chemoattractant protein-1 (MCP-1), and IL-12p70, Fig. 7g–l). Among these, IL-33, IL-1α, and IL-12p70 showed aging-related increases, which were significantly downregulated by 8-AG. These findings suggest that age-related retinal inflammation, marked by elevated pro-inflammatory gene expression and cytokine levels, is effectively alleviated by 8-AG treatment.

### Age-related accumulation of hypoxanthine and xanthine is accompanied by the age-related decline of guanine and 3’5’-cGMP

To assess the effects of aging and 8-AG treatment on the retinal purine metabolites, we quantitatively profile the purine metabolome of the retina using UPLC-MS/MS (Fig. 7m–z). The most abundant purines in rat retinae were adenosine, inosine, 5’-GMP, and 5’-AMP (Fig. 7p, r, x, z). In aged retinae, guanine and 3’5’-cGMP levels were significantly reduced ( > 3-fold, Fig. 7s, w), while inosine, hypoxanthine, and xanthine levels increased ( ~ 2–3-fold, Fig. 7r, u, v). Hypoxanthine accumulation resulted from elevated inosine levels (Fig. 7r). A significant reduction of deoxyguanosine and an increase of hypoxanthine has been reported in old mouse retinae and RPE/choroids^43^, which is coherent with our findings.

RNA-seq data revealed no significant age-related changes in genes encoding adenosine deaminases or PNPase (*Pnp*). However, the expression of guanine deaminase (*Gda*) and xanthine dehydrogenase/oxygenase (*Xdh/Xo*) was upregulated (5-fold and 1.7-fold, respectively, Fig. 7aa), promoting guanine consumption and xanthine production. These findings suggest that the guanine decline and xanthine accumulation are driven by elevated *Gda* and *Xdh* levels in aged retinae.

8-AG increased its retinal bioavailability (Fig. 7n) and slightly reduced hypoxanthine and xanthine levels (Fig. 7u, v) but did not significantly alter PNPase substrates (guanosine and inosine, Fig. 7o, r). This lack of effect on PNPase inhibition in the total retina lysate may be due to PNPase being predominantly expressed in a small subset of retinal cells, with the purine changes diluted by the retinal neurons, which exhibit low PNPase expression. Indeed, published single-cell RNA-seq data of mouse retina show that *Pnp* is expressed in endothelial, astrocytes, microglia, and Müller glia^85^. Additionally, peripheral inhibition of PNPase, for example in the erythrocytes which are a rich source of PNPase, by 8-AG could also be involved in the mechanism of action of 8-AG in the retina.

### Long-term efficacy of 8-AG in Fischer 344 rats

**Fig. 8:**
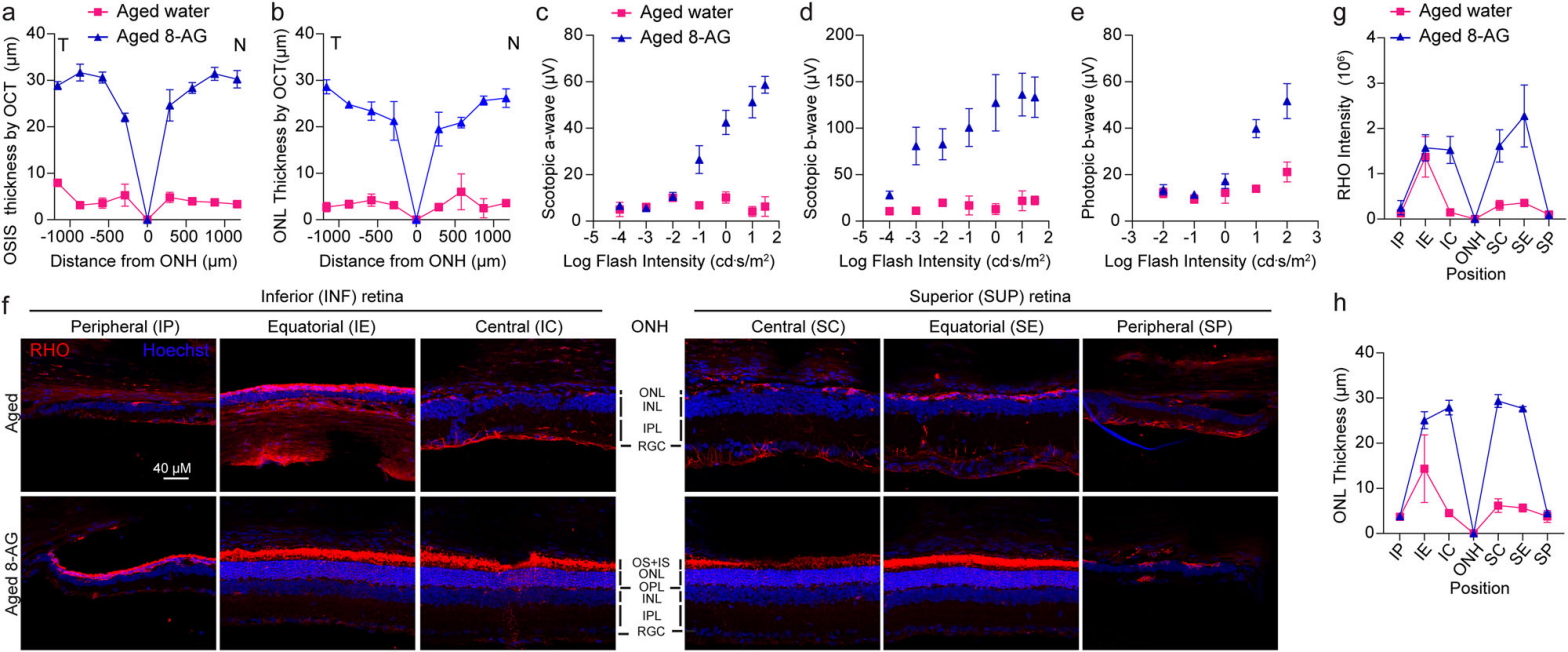
Long-term efficacy of 8-AG in F344 rats. The F344 rats were treated with 8-AG at 5 mg/kg bw daily in drinking water or with water only for 17 weeks starting at 23 months of age. SD-OCT and ERG were performed on rats at the endpoint at 27 months of age, followed by euthanasia for IHC. **a**, **b** The spidergrams for the thickness of OSIS and ONL, respectively, were measured from SD-OCT B-scans. **c**, **d** represent the temporal and nasal side of the retinae. Magenta squares, water-treated aged rats; and blue triangles, 8-AG-treated aged rats. **c**–**e** ERG response for scotopic a-wave (**c**), scotopic b-wave (**d**), and photopic b-wave (**e**) plotted as a function of flash intensity in semi-log format. **f** Representative immunofluorescence images of RHO (red) and Hoechst33342 for nucleus stain (blue) at central (SC&IC), equatorial (SE&IE), and peripheral (SP&IP) regions of the aged untreated and aged 8-AG treated rat retinae. **g**, **h** Spidergrams of RHO intensity in the OSIS (**g**) and ONL thickness (**i**), measured from IHC images shown in **f**. Data points and error bars are means ± SEMs. *N* = 2 for untreated retinae, *N* = 4 for 8-AG for treated retinae. This dataset has a low sample size because the average rat lifespan is 24 months, only one or two animals survived till 27 months for each group. Therefore, *P* values were not shown.

To evaluate the sustained effects of 8-AG, we extended the treatment to 17 weeks in 23-month-old F344 rats (Fig. 8). Due to high mortality beyond 24 months, final analyses included one water-treated (n = 2 eyes) and two 8-AG-treated (n = 4 eyes) rats. SD-OCT revealed preserved retinal structure in both 8-AG-treated rats, while the water-treated rat showed significant degeneration (Fig. 8a, b). Scotopic and photopic ERG confirmed retained rod and cone functions in 8-AG treated rats, contrasting with near-complete functional loss in control rats (Fig. 8c–e). IHC at 27 months showed rhodopsin mislocalization, OS loss, and reduced ONL nuclei (0–1 row) in untreated retinae, whereas 8-AG-treated retinae retained rhodopsin in the OS and 6–7 ONL rows, with no rescue in peripheral regions (Fig. 8f–h and Supplementary Data File 17). These results suggest that long-term 8-AG treatment provides preservation of retinal structure and function in aged rats.

### 8-AG provides temporary retinal protection in the *Rho*^*P23H/+*^ knock-in mouse model of retinitis pigmentosa (RP)

To evaluate its efficacy in another model of retinal degeneration, we administered 8-AG to the *Rho*^*P23H/+*^ knock-in mice^86^ via intraperitoneal (i.p.) injections from postnatal day (PND) 10 to 28, followed by oral dosing until PND38 or PND53 (Supplementary Fig. S4). At PND36, 8-AG enhanced scotopic a- and b-waves (*P* < 0.0001), but only scotopic b-wave improvement persisted at PND50, suggesting short-term rod function enhancement (Supplementary Fig. S4b, c, k, l). No effects on photopic b-waves were observed, consistent with findings in F344 rats. Histological analysis showed increased OSIS thickness at PND38 and 53, and slightly higher ONL cell counts at PND53 (Supplementary Fig. S4e–g, n–p, and Supplementary Data File 18). 8-AG also increased RGC layer neuron numbers at PND38 (*P* < 0.001), though the effect was not significant by PND53 (Supplementary Fig. S4e, h, n–q). RHO levels in the OSIS were elevated at PND38 (*P* < 0.0001), correlating with improved scotopic ERG responses, but this effect was absent at PND53 (Supplementary Fig. S4i, j, r, s, and Supplementary Data File 19). Collectively, 8-AG’s retinal protection appears limited to short-term, in the *Rho*^*P23H/+*^ knock-in mouse model of RP.

### 8-AG treatment reduces microglia/macrophages in *Rho*^*P23H/+*^ mice

Similar to our previous report^87^, retinal flat mounts from *Rho*^*P23H/+*^ mice at PND28 showed a 10-fold increase in activated microglia/macrophages (CD68+ and IBA1+ cells) compared to *Rho*^*+/+*^ mice, with IBA1+ cells outnumbering CD68+ cells (Supplementary Fig. S5a–h, m, n, and Supplementary Data File 20). 8-AG treatment halved the number of activated microglia/macrophages, mirroring its anti-inflammatory effects seen in F344 rats and highlighting its potential to reduce inflammation across different models of retinal degeneration (Supplementary Fig. S5e–n).

## Discussion

This study, extending from its established multi-tissue-protective benefits, highlights the retinal protective effects of 8-AG. We showed strong morphological and functional retinal protection by 8-AG in the aged F344 rats. To be noted, the treatment was oral supplementation in water, at a low dose (5 mg/kg/day), and the intervention was mainly tested at an old age (22–24 months). The 8-AG treatment led to a significantly higher number of photoreceptor cells in the ONL, thicker OSIS, and more cells in the RGC layer, enhanced scotopic ERG responses, and fewer apoptotic cells compared to controls. This marks the identification of an orally administered small molecule with high potency and efficacy in preventing age-related retinal degeneration in an established natural model of aging. The reduction in MDA and 8-OHdG immunostaining indicates 8-AG’s antioxidant action, while the decrease in CD68^+^ and IBA1^+^ cells, alongside RNA-seq, qPCR, and the multiplex cytokine/chemokine panel data, points to its anti-inflammatory impact.

The female F344 rats undergo severe age-related retinal degeneration at 22–24 months of age, likely exacerbated by chronic light damage based on their albino background^88^. Previous studies showed that retinal light damage is a process closely associated with oxidative damage and retinal inflammation, and microglia plays an important role in this process. We found that 8-AG effectively reduced lipid and DNA oxidation in the aged F344 rat retinae, and mitigated microglia activity and retinal immune responses. These can be the main mechanism of action of 8-AG treatment.

Although the ONL and OSIS were preserved, only the scotopic ERG was rescued by 8-AG, suggesting the treatment mainly rescued the rod function, but not cones. IHC did show a higher number of cones on the inferior side of the retinae, where degeneration was more severe. Similar sectorial pharmacological effects have been previously reported for fibroblast growth factor 2 (FGF2) and minocycline treatments^89^ and our previous studies with methotrexate in mice^90^. The small increase in cones on one side of the retina may not be sufficient to rescue the cone function detectable by ERG. These results suggest that the cone degeneration may be caused by other factors not affected by 8-AG treatment.

Using a previously established a UPLC-MS/MS method for measuring purines^91^, we found that cGMP levels dropped significantly in aged F344 rat retinae—a threefold decrease compared to young retinae (Fig. 7w). Considering the cGMP reduction may be due to a reduction of photoreceptor cells by aging, we normalized with ONL cell number and still obtained a two-fold reduction of cGMP levels comparing aged vs. young retinae (15.5 vs. 7.5 pg/ONL cell count across 200 µm retinal length). Further, our retinal RNA-seq data showed that the expression of *Guca1b*, which encodes the guanylate cyclase activator protein 2 (GCAP2) was decreased 1.8-fold in the aged rat retinae compared to the young control (Supplementary Data File 13). GCAP2 is an important activator of guanylate cyclases, which make cGMP in photoreceptors. Given that cGMP is a vital secondary messenger for phototransduction, its depletion could further impair aged retinal function. The reduction of cGMP level can be led to bythe combined results of reduced photoreceptor cells and reduced expression of Guca1b.

Our previous research indicates that oral 8-AG at 5 mg/kg/day increases PNPase substrates and reduces PNPase products in the urine of F344 rats, suggesting it effectively inhibits PNPase systemically^69^. However, 8-AG treatment does not appear to inhibit total PNPase activity in the retina, as retinal guanosine and inosine levels were not significantly affected (Fig. 7o, r). This could relate to the variable Pnp expression across different retinal cell types. Published single-cell-RNA-seq data of the retina provides more clues that suggest Pnp expression is high in astrocytes, Müller glia, and microglia, but low in neurons^92^. However, the potential impact of 8-AG on inhibiting PNPase in these glial cells may not be apparent in overall retinal purine metabolome analyses due to their low abundance. Thus, the whole retina lysate, containing purines mainly from retinal neurons, did not show significant effects, suggesting PNPase inhibition, although the products hypoxanthine and xanthine were non-significantly reduced in the 8-AG-treated retinae (Fig. 7u, v). Pnp levels are also high in immune cells such as T cells, B cells, neutrophils, and macrophages^93^. Inhibiting PNPase in these cells may lead to the accumulation of DNA-derived deoxyguanosine, dGTP, and subsequent suppression of DNA synthesis and cell proliferation^94^. Correlating to our RNA-seq data, qPCR and retinal immune panel assay point to 8-AG’s role in mitigating cell stress and inflammatory responses of immune cells. 8-AG’s anti-inflammatory effects may be due to its PNPase inhibition in the Müller glia, microglia, and infiltrated myeloid cells (T cells, B-cells, and neutrophils) with high Pnp expression.

Although we showed strong retinal protection by 8-AG in the natural rat model of aging, its potential in the pathological condition of AMD is still unknown. One important future study is to test 8-AG in an AMD model, such as the transgenic CFH-Y402H mouse^30^, to evaluate 8-AG’s effects in AMD-related lesions, such as sub-RPE deposit/drusen.

8-AG’s potential as a drug candidate was first discovered in the kidney and then extended to diseases of the cardiovascular system and lower urinary tract (see ref. ^67^ for review). Here we show that 8-AG, an endogenous purine metabolite and an orally active drug candidate, reverses age-related retinal degeneration. The effects of 8-AG on the retina are likely mediated by multiple mechanisms, including anti-oxidative, anti-apoptotic, and anti-inflammatory effects. The present study indicates that age-related degeneration of an “immune-privileged” tissue, i.e., the retina, can be mitigated via systems pharmacotherapy using 8-AG.

## Materials and methods

### Animals

Female young and aged F344 rats were obtained from Charles River Laboratories and the National Institute on Aging (NIA). The 3-month-old rats were used as young controls, while 22–24-month-old aged rats were used with or without 8-AG treatment. Only female rats were used in this study because of the sexual differences in retinal degeneration rate and onset. The 8-AG-treated rats received 8-AG (5 mg/kg body weight per day) via supplementation in drinking water, while the water-treated control group received water only. 8-AG-supplemented water was replaced daily. The 8-week treatment was started at 22 months of age, and the 17-week treatment was started at 23 months of age. A total of 90 rats were used in this study.

The C57 black 6 (C57BL/6 J) mice (*Rho*^*+/+*^, Stock no-000664) and (*Rho*^*P23H/P23H*^ knock-in, Stock no-017628) mice^86^ were purchased from Jackson Laboratory. The *Rho*^*P23H/+*^ mice were generated by breeding the *Rho*^*+/+*^ with *Rho*^*P23H/P23H*^ animals. Both male and female mice were included in this study. A total of 72 mice were used in this study.

All animals were housed under standard 12-h light/12-h dark conditions in the animal facility at the University of Pittsburgh. For experimental procedures, animals were anesthetized via intraperitoneal (i.p.) injection of a ketamine/xylazine cocktail (80 mg/kg ketamine and 7 mg/kg xylazine; Henry Schein, Dublin, OH, USA). Pupils were dilated with 1% tropicamide (Akorn, Lake Forest, IL, USA) and 2.5% phenylephrine (Sigma-Aldrich, Saint Louis, MO, USA) before performing SD-OCT and ERG recordings. During anesthesia, eyes were lubricated with GenTeal Tears lubricant eye gel (Alcon Laboratories, Fort Worth, TX, USA). Euthanasia was performed by CO_2_ inhalation followed by cervical dislocation. All animal experiments adhered to the Animal Welfare Act and Regulations Guide and were approved by the University of Pittsburgh Institutional Animal Care and Use Committee (IACUC; rat protocol number 23073400, mouse protocol number 23053112). We have complied with all relevant ethical regulations for animal use.

### SD-OCT

Retinal morphological changes were assessed in vivo using SD-OCT (Bioptigen, Morrisville, NC, USA). Animals were anesthetized, their pupils dilated, and their eyes lubricated. Body temperature was maintained using a heating pad. The A-scan/B-scan ratio was set at 1200 lines. Retinal B-scan images were acquired at 0°, with five frames averaged and saved. The thickness of the ONL and the OSIS was measured at six points along the OCT scan from the temporal to the nasal side and plotted as a spidergram. Statistical significance between treatment groups was determined using two-way ANOVA, with treatment condition and position relative to the optic nerve head as the two factors.

### ERG

Animals were dark-adapted overnight, and ERG recordings were performed using the Celeris system (Diagnosys, Lowell, MA, USA) under dim red light. Pupils were dilated, and animals were anesthetized, and body temperature was maintained at 37 °C. Scotopic ERG responses were recorded from dark-adapted eyes stimulated by flash intensities at 0.0001, 0.001, 0.01, 0.1, 1, 10 and 30 cd s/m^2^, with three to five sweeps per flash intensity averaged and inter-sweep intervals of 10–30 seconds. Photopic ERG responses were recorded after light adaptation at 10 cd/m^2^ for 10 minutes, with flash stimulations at 0.01, 0.1, 1, 10, and 100 cd s/m^2^ superimposed on the 10 cd/m^2^ background light. Statistical significance between treatment groups was determined using two-way ANOVA, with treatment condition and flash intensity as the two factors.

### Tissue collection and IHC

Animals were euthanized, and the superior side of each eye was marked using a cautery pen. Enucleated eyes were prepared as eye cups by removing the anterior segment, including the cornea, iris, and lens. Eye cups were fixed in 4% paraformaldehyde (PFA) for 2 hours, then dehydrated in 5%, 10%, 20%, and 40% sucrose solutions in phosphate buffered saline (PBS, pH 7.4) for 30 minutes each. They were subsequently incubated overnight in a 40% sucrose/PBS and O.C.T. compound mixture (1:3 ratio, Fisher Scientific, Houston, TX, USA). Eyes were embedded in O.C.T. and frozen in liquid nitrogen-bathed isobutane. Twelve-micron retinal cross-sections containing the optic nerve head were mounted onto SuperFrost glass slides (Fisher Scientific) for IHC.

Retinal flat mounts were prepared by making four cuts to flatten the retina, followed by removal of the sclera. Flat mounts were fixed in 4% PFA for 2 hours and stained for CD68 and IBA1.

IHC was performed on both retinal cryosections and flat mounts. Cryosections were rehydrated, permeabilized in PBS with Triton-X-100 (PBST, 0.1% Triton-X-100) for 30 minutes, and blocked in 5% goat serum in PBST for 30 minutes. Sections were incubated with primary antibodies diluted in 5% goat serum for 2 hours. The following primary antibodies were used: mouse anti-rhodopsin (1D4, 20 μg/mL, gift from Dr. Krzysztof Palczewski), mouse anti-arrestin 1 (MCA-S1288, Encor Biotechnology, 1 μg/mL), rabbit anti-PDE-6B (PA1-722, Invitrogen, 4 μg/mL), rabbit anti-TOMM20 (ab186734, Abcam, 0.5 μg/mL), rabbit anti-CD68 (ab125212, Abcam, 1 μg/mL), SPICA Dye™ 568-conjugated rabbit anti-IBA1 (015-28011, Wako Pure Chemical Corporation, 1:200), rabbit anti-GFAP (z0334, Sigma-Aldrich, 6.4 μg/mL), and rabbit anti-8-OHdG (NB110-96878, Novus Biologicals, Wrentham, MA, USA, 2 μg/mL). After three washes in PBST, tissues were incubated with secondary antibodies—goat anti-mouse Alexa Fluor 546 (A-11030, Thermo Scientific, Waltham, MA, USA, 5 μg/mL) and goat anti-rabbit Alexa Fluor 488 (A-11008, Thermo Scientific, 2 μg/mL)—in PBST containing Hoechst33342 (62249, Thermo Scientific, 2 μg/mL) for 1 hour. Samples were washed in PBST and mounted with ProLong Gold mounting solution (Thermo Scientific) before imaging on an Olympus FV1200 confocal microscope. The Click-iT Plus TUNEL Assay Alexa Fluor 594 Kit (C10618, Thermo Scientific) was used to detect in situ apoptosis, following the manufacturer’s instructions. Fluorescence intensity, thickness measurements, and cell counting were performed using Fiji software (imagej.nih.gov/ij)^95^.

### Retinal histology and hematoxylin and eosin (H&E) staining

Retinal morphology was assessed using histological analysis. Animals were euthanized and eyes were enucleated and fixed in 4% PFA and 1% glutaraldehyde for 24 hours before paraffin embedding. Paraffin sections (7-μm thick) were stained with H&E and imaged using a light microscope (Leica, Wetzlar, Germany). Retinal thickness measurements and cell counts were analyzed using Fiji (ImageJ) software.

### RNA-seq and transcriptome analysis

Retinae from young, aged untreated, and aged 8-AG-treated rats were collected in RNAzol (Sigma-Aldrich), and total RNA was extracted according to the manufacturer’s instructions. After retinal isolation, RPE/choroid tissues were collected by washing the remaining eye cups with RNAzol. Due to the low abundance of cells, RPE/choroids from two eyes were pooled to create one sample. Only samples with an RNA Integrity Number (RIN) > 7 were used for poly-A mRNA library preparation. Library quality was assessed using a Bioanalyzer, and RNA-seq was performed on the Illumina HiSeq platform by QuickBiology Inc. (Monrovia, CA, USA). Reads were mapped using the UCSC transcript set (Bowtie2 version 2.1.0), and expression levels were estimated with RSEM v1.2.15. DEG were identified using the edgeR program and filtered with criteria of false discovery rate (FDR) < 0.05 and fold change >1.5. Gene ontology (GO) enrichment analysis and pathway analysis were performed using Goseq and Kobas, respectively.

### Immunblots

Retinae were collected from euthanized animals and lysed in PBS with 0.1% SDS. Tissue lysates were sonicated at 25% power for 12 seconds on ice, centrifuged at 500 × *g* for 10 minutes to remove insoluble debris, and the supernatants were collected for immunoblotting. Total protein concentration was measured using OD_280_ on a Nanodrop Spectrophotometer. Tissue samples were resolved on a 12% SDS-PAGE gel and transferred to a nitrocellulose membrane using a wet transfer method. Membranes were blocked in 5% nonfat milk in PBST for 30 minutes and incubated with primary antibodies overnight at 4 °C. The primary antibodies used included horseradish peroxidase (HRP)-conjugated 1D4 anti-RHO antibody (0.2 μg/mL, prepared using an HRP-conjugation kit on 1D4 anti-RHO antibody shared by Dr. Krzysztof Palczewski), rabbit anti-cleaved caspase-3 (9664, Cell Signaling, Danvers, MA, USA, 1:1000), and rabbit anti-caspase-3 (9662, Cell Signaling, 1:1000). After three washes with PBST, membranes were incubated with secondary antibodies—anti-mouse IgG-HRP (Thermo Scientific, 0.1 µg/mL, 1:5000) or anti-rabbit IgG-HRP (Thermo Scientific, 0.1 µg/mL, 1:5000)—for 1 hour, followed by three additional 5-minute washes in PBST. Immunoblots were developed using the SuperSignal West Pico Chemiluminescent Substrate for HRP (Fisher Scientific). Membranes were stripped using Restore Plus Western Stripping Buffer (Fisher Scientific) for 5 minutes at room temperature, rinsed under running water, and re-immunoblotted. Ponceau staining was used as a loading control. Band intensities were quantified using Fiji (ImageJ) software.

### Transmission electron microscopy

Eyes were harvested and immersion-fixed in 2.5% glutaraldehyde and 2% PFA in PBS overnight at 4 °C. Following fixation, tissues were washed three times in PBS and post-fixed in 1% aqueous OsO_4_ and 1% K_3_Fe(CN)_6_ for 1 hour. After three additional PBS washes, tissues were dehydrated through a graded ethanol series (30%–100%), followed by 100% propylene oxide. Tissues were then infiltrated with a 1:1 mixture of propylene oxide and Polybed 812 epoxy resin (Polysciences, Warrington, PA, USA) for 1 hour. Tissues underwent several changes of 100% resin over 24 hours, were embedded in molds, cured at 37 °C overnight, and further hardened at 65 °C for 2 days. Ultrathin cross-sections (60 nm) of the retinae were collected on copper grids and stained with 1% uranyl acetate for 10 minutes, followed by 1% lead citrate for 7 minutes. Sections were imaged using a JEOL JEM 1400 Flash transmission electron microscope (Peabody, MA, USA) operating at 80 kV, and images were captured with a bottom-mount AMT 2k digital camera (Advanced Microscopy Techniques, Danvers, MA, USA).

### Retina Inflammation panel assay

The LEGENDplex™ Rat Inflammation Panel (13-plex) kit (741396, BioLegend, San Diego, CA, USA) was used to quantify 13 cytokines/chemokines in the F344 rat retinae. All components were brought to room temperature before use. The 1× Wash Buffer was prepared by diluting 20× Wash Buffer with deionized water containing 10% (v/v) 10× protease inhibitor. Standard A was dissolved in 2.5 mL Lyophilized Standard Reconstitution Buffer and incubated for 10 minutes for reconstitution, followed by reconstitution of Standard B using 250 µL of the reconstituted Standard A. A 1:4 dilution sequence with Assay Buffer was performed to prepare six standard solutions, which, along with the reconstituted standard and 1× Wash Buffer, were loaded into a V-bottom 96-well plate. Flash-frozen retinae were weighed, diluted in Assay Buffer (10 µL/mg), homogenized using the MP Biomedicals FastPrep-24™ 5 G, centrifuged at 12,000 × *g* for 10 minutes, and the supernatants were loaded onto the plate. Each well contained 25 µL of retina homogenate or standard analyte solution, 25 µL Assay Buffer and 25 µL vortexed Premixed Beads. The plate was shaken in the dark at 800 rpm for 2 hours, centrifuged at 250 ×  *g* for 5 minutes to pellet the beads, and the supernatant was discarded. Pellets were washed twice with 200 µL 1× Wash Buffer (1-minute incubation followed by centrifugation) and then incubated with 25 µL Detection Antibody for 1 hour while shaking in the dark. Subsequently, 25 µL SA-PE was added to each well, and the plate was shaken for 30 minutes in the dark at 800 rpm. After centrifugation at 250 × *g* for 5 minutes, the supernatant was discarded, and the pellets were washed twice with 200 µL 1× Wash Buffer before being resuspended in 150 µL 1× Wash Buffer. The samples were analyzed using a Beckman Coulter CytoFLEX LX flow cytometer. All retina samples and standard solutions were prepared and analyzed in duplicates at room temperature.

### Real-time PCR

Rat retinae were isolated, and total RNA was extracted using RNAzol reagent according to the manufacturer’s instructions. Reverse transcription was performed using the High-Capacity RNA-to-cDNA Kit (4387406, Life Technologies). Real-time PCR was conducted with PowerTrack SYBR Green Master Mix (Thermo Scientific) under the following conditions: 95 °C for 10 minutes, followed by 40 cycles of 95 °C for 15 seconds and 55 °C for 1 minute. *Gapdh* was used as the internal control. The primers used are listed in Table 1.

**Table 1:** Primer sequence for selected genes

Primer	Sequence
*Mt2a* forward	5-CAGCGATCTCTCGTTGATCT-3
*Mt2a* reverse	5-AACAGCAGCTTTTCTTGCAG-3
*Adgre1* forward	5-ACTCCAAGAGCTTCATGCAC-3
*Adgre1* reverse	5-TCATAGTTGCAAGGCACAGA-3
*Itgal* forward	5-CCTACCAGTTTGCTGCTGTT-3
*Itgal* reverse	5-GGCACCAGACTCTTCTTTGA-3
*Cd68* forward	5-ACAGTTTCTCCCACCACAAA-3
*Cd68* reverse	5-CCTGGGTCAGGTACAAGATG-3
*Itgb2* forward	5-AGCCACCTCTGTGTGAAGAG-3
*Itgb2* reverse	5-TCTTGAAAGGGACAGTCTGC-3
*Rt1-Da* forward	5-ACAACACTCCAGATGCCAAT-3
*Rt1-Da* reverse	5-GGAGGGGAGAACTTGTCAAT-3
*Gapdh* forward	5-AGTGCCAGCCTCGTCTCATA-3
*Gapdh* reverse	5-GATGGTGATGGGTTTCCCGT-3

### Purine metabolome analyses

Retina was isolated, put in a vial and flash-frozen in liquid nitrogen. Each frozen retina was put in a 50 mL tube and rapidly submerged with 6 mL of ice-cold tissue lysis buffer containing a mixture of acetonitrile, methanol and water at 1:2:2 volume ratio. The tissue was homogenized using a manual homogenizer on ice. Then, the tissue homogenate was heated at 60 °C for 10 min for complete enzyme denaturation. One mL of the tissue homogenate was aliquoted and centrifuged at 3000 × *g* for 90 minutes at 4 °C, and the supernatant was transferred to a clean tube. The pellet was then mixed with additional 0.5 mL tissue lysis buffer and centrifuged at 3000 × g for 30 minutes at 4 °C and this supernatant was added to the supernatant collected from the first round of centrifugation. The 1.5 mL of supernatant was then dried in a sample concentrator down to ~0.3 mL, followed by an addition of 0.7 mL of water to make 1 mL. Each sample was filtered using a 30 kDa Micron YM-30 centrifugal unit (MiliPore). One hundred µL of the ultrafiltrated sample was then spiked with the internal standard for UPLC-MS/MS analyses. Our UPLC-MS/MS purine metabolome assay was recently updated in reference^91^.

All procedures were approved by the University of Pittsburgh Institutional Biosafety Committee (IBC201700072).

### Statistics and reproducibility

All statistical analyses were performed using Graphpad Prism. Each treatment group contains 6 animals, and the treatment was repeated five times. Sample sizes were determined by a combined factor of limited animals per cohort provided by NIA (<20 animals per cohort) and a power analysis to show significant between-groups with an effect size of 2 with α error<0.05 and power >0.8. Power analysis was performed using G*Power^96^. Treatment groups were selected randomly by cage number, with each cage housing two animals, as each cage shared the same bottle of drinking water with or without 8-AG supplementation. Treatment, efficacy test, histology, and image analyses were performed by different researchers, and sample groups were matched to the animal ID after data analyses to avoid subjective biases. Animals were excluded if they showed a visible corneal wound, cataract, or died before the endpoint. Samples were excluded if they showed poor quality due to damage during processing. A two-way analysis of variance (ANOVA) was used to assess statistical differences between two groups of animals for retinal thickness measurements from OCT B-scans, retinal layer thickness, nucleus counts, immunofluorescence intensity from retinal histology and IHC images, and ERG a- and b-wave amplitudes. The analysis considered two factors: treatment and positions relative to the optic nerve head (ONH) for OCT, IHC, and retinal histology); or treatment and flash amplitude for ERG. For comparisons between two groups of data, an unpaired two-tailed Student’s *t* test was applied. For data involving three or more groups, Kruskal–Wallis one-way ANOVA followed by Dunn’s multiple comparisons test was performed. Data were presented as means ± SEMs or SDs, as specified in the figure legends. Except for TEM, each measurement was taken from distinct samples from different animals. For TEM, each measurement was taken from one TEM image, and statistical significance was determined when *P* < 0.05. The following experiments were performed multiple times: OCT (five times), ERG (four times), immunoblots (two times), retinal histology and H&E staining (three times), TEM (two times), RNA-seq of the neural retina (two times), purine metabolome analysis (three times), rat retina inflammation panel assay (two times, each sample tested in duplicates), and real-time PCR (two times, each sample tested in duplicates). TEM data were pooled for analysis, and only one representative dataset was shown in the figures for each experiment.

#### Reporting summary

Further information on research design is available in the Nature Portfolio Reporting Summary linked to this article.

## Supplementary information

Supplementary information

Description of the Supplementary Data Files

Supplementary Data 1

Supplementary Data 2

Supplementary Data 3

Supplementary Data 4

Supplementary Data 5

Supplementary Data 6

Supplementary Data 7

Supplementary Data 8

Supplementary Data 9

Supplementary Data 10

Supplementary Data 11

Supplementary Data 12

Supplementary Data 13

Supplementary Data 14

Supplementary Data 15

Supplementary Data 16

Supplementary Data 17

Supplementary Data 18

Supplementary Data 19

Supplementary Data 20

Supplementary Data 21

Reporting summary

## Data Availability

RNA-seq data generated in this study have been deposited in the GEO database under the accession number GSE254123. The Supplementary Data Files include all histology, IHC, immunoblot, List of DEGs, and TEM images. Full scans of immunoblots were listed in Supplementary Data File 4. Raw data for the plots shown in the Figures were uploaded as Supplementary Data File 21. All procedures were approved by the University of Pittsburgh Institutional Biosafety Committee (IBC201700072).
