# Supplementary material for: Oral 8-aminoguanine against age-related retinal degeneration
Source: Commun Biol. 2025 May 26;8:812. doi: 10.1038/s42003-025-08242-1 (PMC12106806; doi:10.1038/s42003-025-08242-1)
Supplement: Supplementary file 1 — Supplementary information [file 42003_2025_8242_MOESM1_ESM.pdf]

Supplementary materials

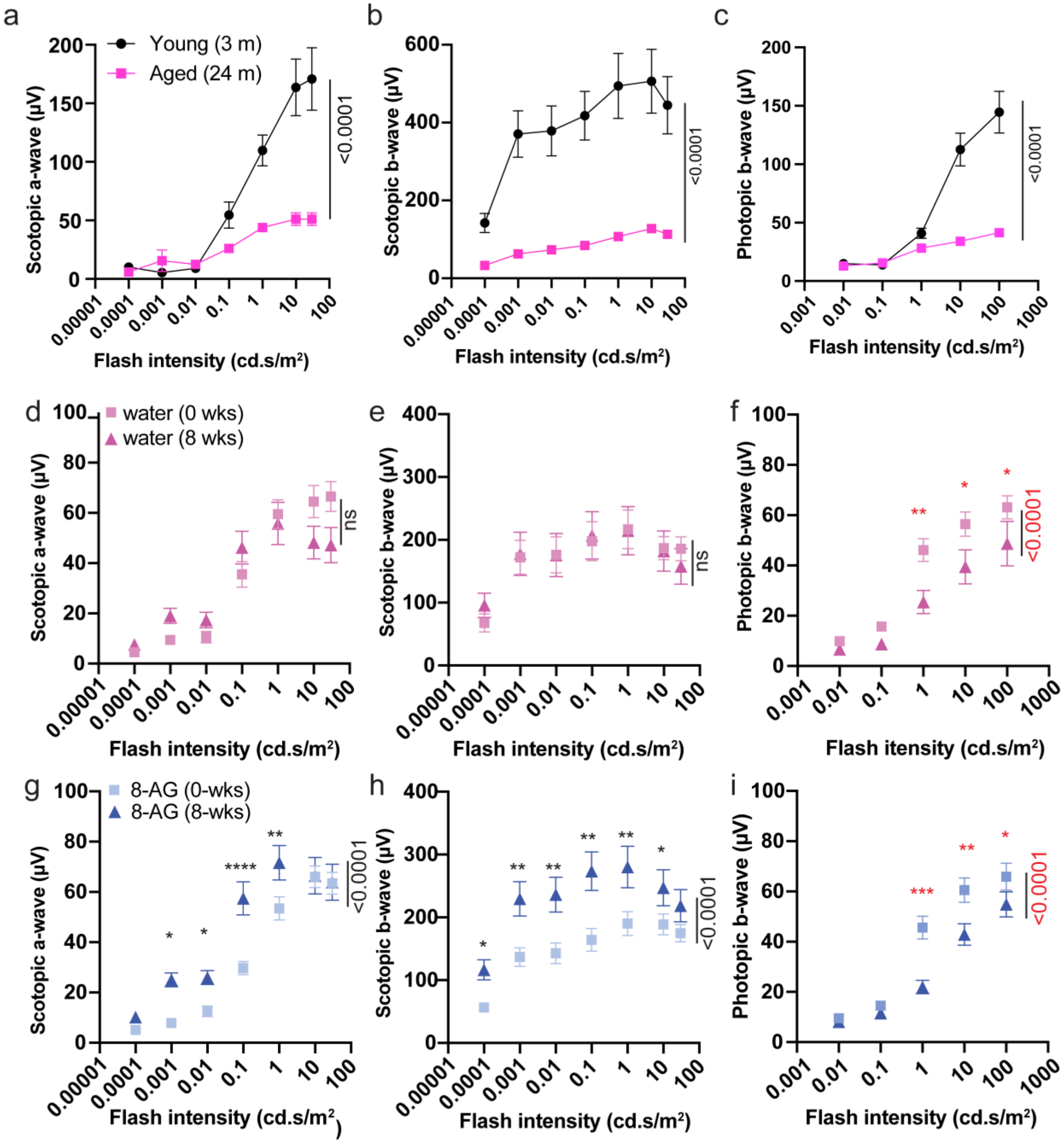

Supplementary Figure S1. Electroretinograms (ERG) of young and aged rats. Twenty-two-month-old aged Fischer344 (F344) rats were supplemented with 8-AG in drinking water at 5 mg/kg for 8 weeks. Electroretinograms (ERG) were recorded for baseline at 0 weeks (at 22 months), and after 8 weeks of treatment (at 24 months). Aged F344 (24 months) and young rats treated with water were included as controls. **a-c**. The amplitudes of scotopic a-wave responses, scotopic b-wave responses, and photopic b-wave responses of rats, respectively, are plotted as a function of flash intensity in semi-log format. N=6. Black circles, young rats; and magenta squares, aged rats. **d-i** are the semi-log plots of ERG responses in μV as a function of flash intensities (cd.s/m^2) for water and 8-AG treated F344 rats. **d, e,** and **f** are the scotopic a-

wave, scotopic b-wave, and photopic b-wave responses, respectively, for the control rats treated with only water at 0 weeks (light magenta squares, baseline) and 8 weeks (dark magenta triangles). **g, h,** and **i** are the scotopic a-wave, scotopic b-wave, and photopic b-wave responses, respectively, for 8-AG treated rats at 0 weeks (light blue squares, baseline) and after 8 weeks of 8-AG treatment (dark blue triangle). Data and error bars are means±SEM. *P* values were calculated by a two-way ANOVA.

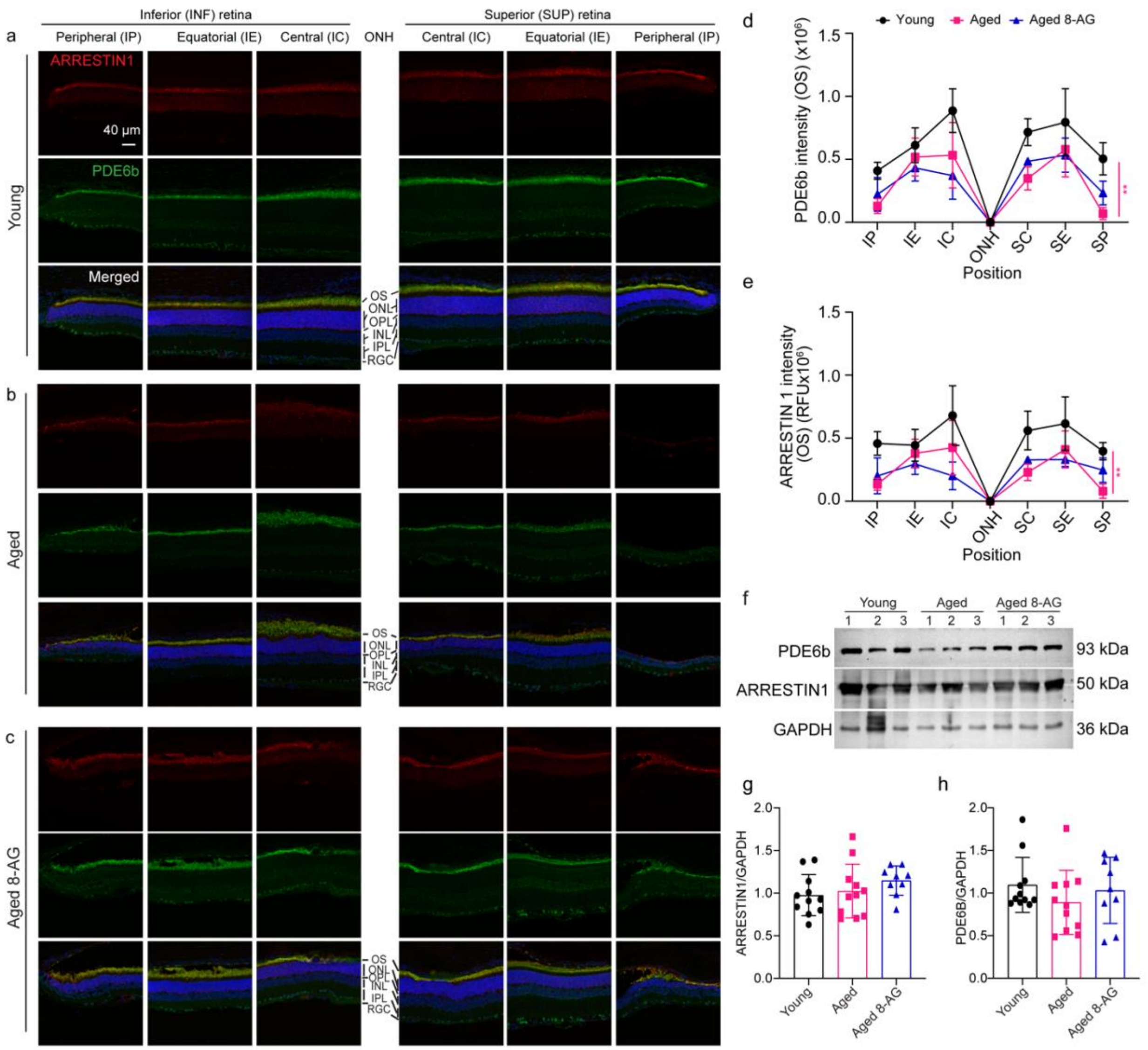

Supplementary Figure S2. Immunostaining and immunoblots of Arrestin 1 and PDE6b using the retinal cryosections and retinal lysates from the young (4 months), Aged (24 months) and 8-AG treated aged rats (Aged 8-AG, 24 months). **a-c.** Immunostaining of Arresting 1 (red), PED6b (green) and Hoechst33343 (blue) on the cryosections of the young, aged and 8-AG treated aged rat retinae, respectively. Scale, 40 µm. **d and e** are spidergrams of PDE6b and Arrestin1 immunofluorescence intensities in the outer segments (OS), respectively. Black, young rats; magenta, aged rats; blue, aged 8-AG. N=4-5. Data and errors are means±SD. **, *P*<0.01 by a two-way ANOVA. **F.** Immunoblots of PDE6, Arrestin1 and GAPDH (loading control) from retinal lysates of young, aged and 8-AG treated aged rats. **g and h** are column plots of Arrestin 1 and PDE6B band intensities, respectively, normalized by GAPDH loading control. N=9-11.

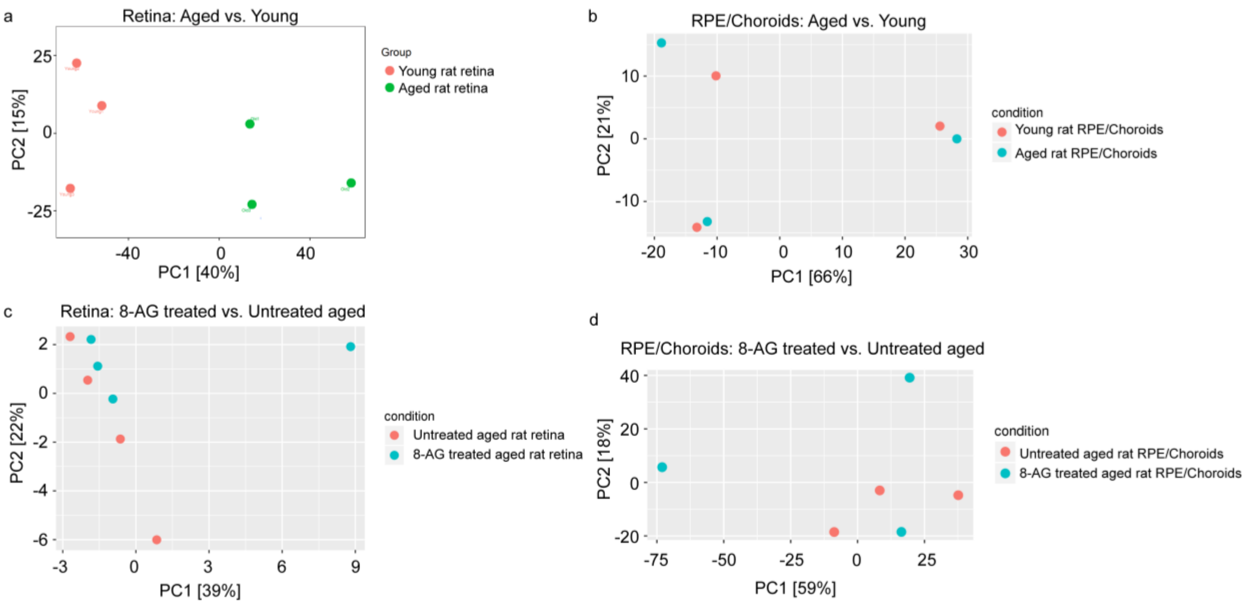

Supplementary Figure S3. Principal Component Analysis (PCA) plots of RNA-seq data from the neural retina (**a** and **c**) and RPE/choroids (**b** and **d**) comparing samples from different groups. **a**, retinae of aged (24 months) vs young rats (4 months). **b**, RPE/choroids of aged vs. young rats. **c**, retinae of 8-AG treated aged (24 months) vs untreated aged rats (24 months). **d**, RPE/choroids of 8-AG treated vs untreated aged rats. The x and y axis are the first two PCs extracted from the dataset and the percentage values in the square brackets indicate the variance in the data by each PC. N=3-4.

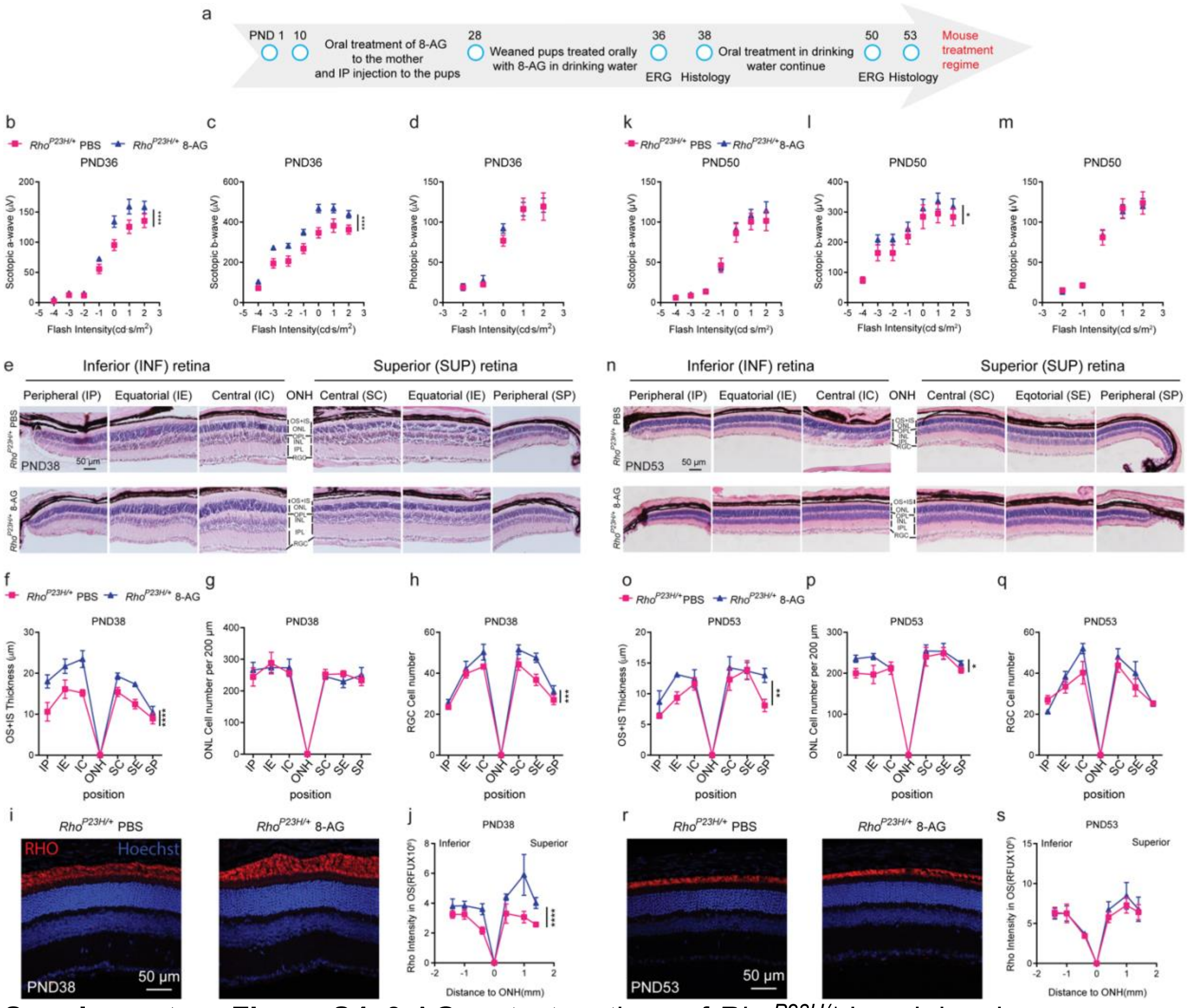

Supplementary Figure S4. 8-AG protects retinae of *Rho*$^{P23H/+}$ knock-in mice. a. *Rho*$^{P23H/+}$ mice treated with 8-AG at 11 mg/kg bw daily starting at PND 10 as shown in the treatment regimen in **a.** ERG recordings were performed at PND 36 and 50 for animals with 2 and 4 weeks of treatment, respectively. Mice were euthanized and eyes were enucleated at PND 38 and PND 53 for H&E staining or immunohistochemistry on retinal cross-sections. PBS-treated animals are used as vehicle control. **b-j** are data set obtained from animals treated with 8-AG or vehicle for 2 weeks. **k-s** are data sets from animals treated with 8-AG or vehicle for 4 weeks. **b**&**k, c**&**l,** and **d**&**m** are scotopic a-wave, b-wave and photopic b-wave responses plotted as a function of flash intensity in semi-log format at PND 36 and 50, respectively. Magenta squares, PBS-treated *Rho*$^{P23H/+}$ mice; and blue triangles, 8-AG-treated *Rho*$^{P23H/+}$ mice. N=4. **e**&**n** are hematoxylin and eosin staining images on the superior (S) and inferior (I) sides of optic nerve head (ONH) at central (SC&IC), equatorial (SE&IE) and peripheral (SP&IP) regions of retinal paraffin sections, Scale bar, 50 µm. **f**&**o**, **g**&**p**, and **h**&**q** show the spidergrams of OS+IS thickness, ONL cell number, and RGC cell number per 200 µm retinal section, respectively, at PND 38 and 53. **i**&**r** are the representative immunofluorescence image of mice retinal cross-sections at central position at PND38 and 53, respectively. Red, RHO; and blue, Hoechst33343 for nucleus staining. Scale

bar, 50 μm. **j**&**s** are spidergrams of RHO intensity in OS at different positions of the retinae generated from I and R, respectively. N=5. Data points and error bars are means and SEMs, respectively. *, **, ***, ****, P<0.05,0.01,0.001, and 0.0001, respectively, by two-way ANOVA.

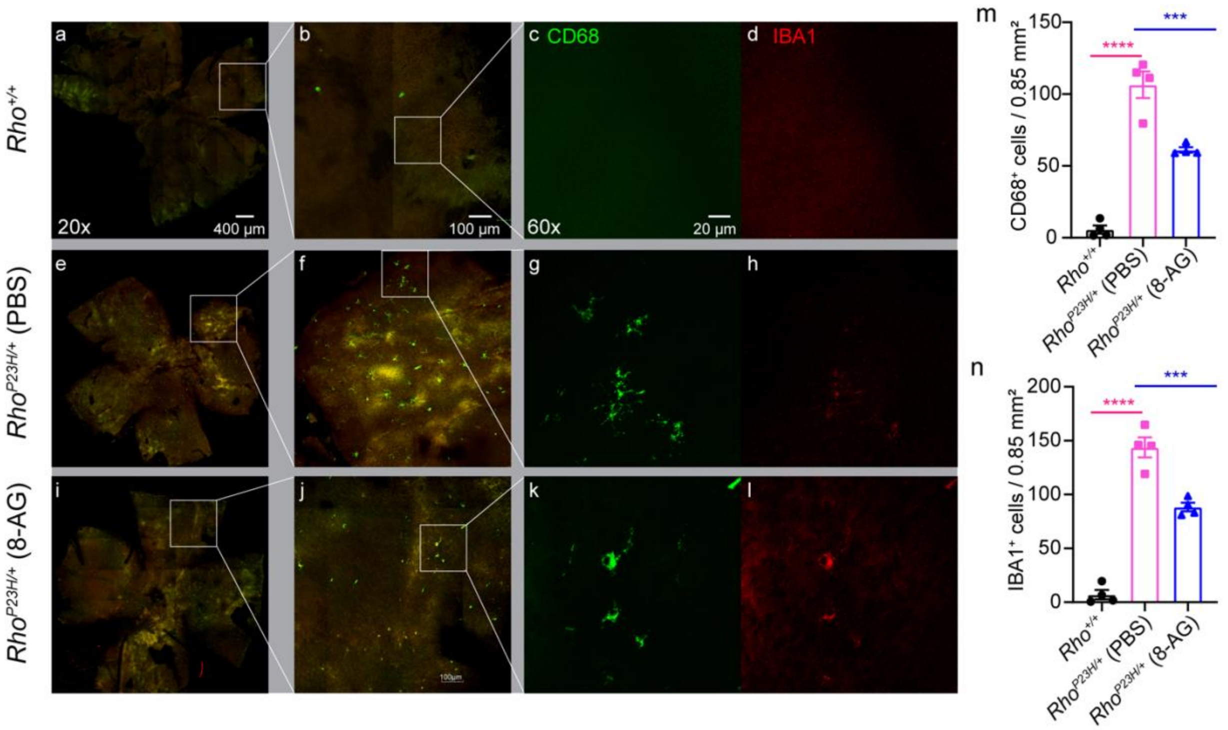

Supplementary Figure S5. 8-AG reduces macrophages in *Rho*$^{P23H/+}$ knock-in mice. *Rho*$^{P23H/+}$ mice were treated with 8-AG at 11 mg/kg bw via daily IP injections from PND 10 to 28 followed by oral 8-AG treatment (Figure S4A). Mice were euthanized and eyes were enucleated for retinal flat mount immunostaining. **a-l.** Immunofluorescence images of retinal flat mounts stained with CD68 and IBA1 as markers of microglia/macrophages. **a-d.** Top panels, retina from *Rho*$^{+/+}$ mice; **e-h** middle panels, retina from *Rho*$^{P23H/+}$ mice treated with PBS control; and **i-l** bottom panel, retinae from *Rho*$^{P23H/+}$ treated with 8-AG. Images from left to right are stitched composite images of whole retinae (A, E, and I) from images obtained at 20x objective with the scale bar at 400 µm; enlarged retinal flat-mount images (**b, f**, and **j**) with composite channels of red and green under 20x objective with the scale bar at 100 µm; immunofluorescence of CD68 staining (**c, g,** and **k**) in green under 60x objective with the scale bar at 20 µm; and immunofluorescence images of IBA1 (**d, h,** and **l**) staining in red under 60 x objective. White squares indicate the regions of where the enlarged images shown on the right were taken. **m** and **n.** Bar graphs of CD68^{+} and IBA1^{+} cells number per 0.85 mm^{2} area, respectively. N=4. Bar heights and error bars are means and SDs, respectively. ***, p<0.001 and 0.0001, respectively, analyzed by one-way ANOVA.
