# Supplementary material for: Oral 8-aminoguanine against age-related retinal degeneration
Source: Commun Biol. 2025 May 26;8:812. doi: 10.1038/s42003-025-08242-1 (PMC12106806; doi:10.1038/s42003-025-08242-1)
Supplement: Supplementary file 2 — Description of the Supplementary Data Files [file 42003_2025_8242_MOESM2_ESM.pdf]

Description of Additional Supplementary Files

File Name: Supplementary Data File 1
Description: Spectral domain-optic coherence tomography (SD-OCT) B scans of Fischer 344 rats at baseline and 8 weeks of treatment with 8-AG (5 mg/kg bw) or water only for Figure 1H-J.

File Name: Supplementary Data File 2
Description: Fischer 344 rat retinal histology hematoxylin and eosin (H&E) stain images for Figure 1K-M.

File Name: Supplementary Data File 3
Description: Terminal deoxynucleotidyl transferase dUTP nick-end labeling (TUNEL) staining images of the retinal cryosections from Fischer 344 rats for Figure 2A-D. Green, TUNEL; and blue, Hoechst33342 for nucleus staining.

File Name: Supplementary Data File 4
Description: Immunoblot full scans of intact and cleaved Caspase 3 for Figure 2E-F and immunoblot of rhodopsin for Figure 2K-L. Each lane was loaded with 40 µg of total protein of retinal lysate from Fischer 344 rats.

File Name: Supplementary Data File 5
Description: Immunofluorecence staining of rhodopsin (RHO) on the retinal cryosections from Fischer 344 rats for Figure 2G-J. Red, RHO; and blue, Hoechst33342 for nucleus.

File Name: Supplementary Data File 6
Description: Immunofluorescence staining of Arrestin1 and phosphodiester 6B (PDE6B) on the retinal cryosections from Fischer344 rats for Figure S3. Green, PDE6B, red, Arrestin1; and blue, Hoechst33342 for nucleus.

File Name: Supplementary Data File 7
Description: Immunofluorescence staining of peanut agglutinin lectin (PNA) on the retinal cryosections from Fischer 344 rats for Figure 2M-P. White, PNA staining for cones, and blue, Hoechst33342 stain for nucleus.

File Name: Supplementary Data File 8
Description: Immunofluorescence staining of malondialdehyde (MDA) on the retinal cryosections from Fischer 344 rats for Figure 3A-E. Green, MDA staining; and blue, Hoechst33342 staining for nucleus.

File Name: Supplementary Data File 9
Description: Immunofluorescence staining of 8-Hydroxy-2'-deoxyguanosine (8-OHdG) and Translocase of Outer Mitochondrial Membrane 20 (TOMM20) on the retinal cryosections from Fischer 344 rats for Figure 3F-R. Green, TOMM20; red, 8-OHdG; and blue, Hoechst33342 staining for nucleus.

File Name: Supplementary Data File 10
Description: Immunofluorescence staining of glial fibrillary acidic protein (GFAP) on the Fischer 344 rats for Figure 3S-V. Green, GFAP staining; and blue, Hoechst33342.

File Name: Supplementary Data File 11
Description: Immunofluorescence staining of Ionized calcium-binding adaptor molecule 1 (IBA1) and CD68 on the retinal cryosections Fischer 344 rats for Figure 3W-AB. Red, IBA1; green, CD68; and blue, Hoechst33342 for nucleus staining.

File Name: Supplementary Data File 12
Description: Transmission electronic microscopy images of retina cross-sections from Fischer 344 rats at peripheral and central areas for Figure 4.

File Name: Supplementary Data File 13
Description: Differentially expressed genes comparing the aged (24 months) vs. young retinae (4 months) from Fischer 344 rats. Cut-off for DEGs were slected at fold change >1.5 or <-1.5 and false discovery rate smaller than 0.05.

File Name: Supplementary Data File 14
Description: Differentially expressed genes comparing the aged 8-aminoguanine-treated (24 months) vs. aged water-treated retinae (24 months) from Fischer 344 rats. Cut-off for DEGs were selected at fold change >1.5 or <-1.5 and false discovery rate smaller than 0.05.

File Name: Supplementary Data File 15
Description: Differentially expressed genes comparing the aged (24 months) vs. young (4 months) retinal pigmented epithelium (RPE) from Fischer 344 rats. Cut-off for DEGs were slected at fold change >1.5 or <-1.5 and false discovery rate smaller than 0.05.

File Name: Supplementary Data File 16
Description: Differentially expressed genes comparing the aged 8-aminoguanine-treated (24 months) vs. aged water-treated (24 months) retinal pigmented epithelium (RPE) from Fischer 344 rats. Cut-off for DEGs were selected at fold change >1.5 or <-1.5 and false discovery rate smaller than 0.05.

File Name: Supplementary Data File 17
Description: Immunofluorecence staining of rhodopsin (RHO) on the retinal cryosections from Fischer 344 rats at 27 months of age for Figure 8F-H. Red, RHO; and blue, Hoechst33342 for nucleus.

File Name: Supplementary Data File 18
Description: *RhoP23H/+* mouse retinal histology hematoxylin and eosin (H&E) stain images for Figure S4E-H&N-Q.

File Name: Supplementary Data File 19
Description: Immunofluorecence staining of rhodopsin (RHO) on the retinal cryosections from *RhoP23H/+* mice for Figure S4I-J&R-S. Red, RHO; and blue, Hoechst33342 for nucleus.

File Name: Supplementary Data File 20
Description: Immunofluorecence staining of Ionized calcium-binding adaptor molecule 1 (IBA1) and CD68 on the retinal flat mounts of *RhoP23H/+* mice for Figure S5. Red, IBA1; green, CD68; and blue, Hoechst33342 for nucleus staining.

File Name: Supplementary Data File 21
Description: All raw data values in an Excel fie.
