# Supplementary material for: Oral 8-aminoguanine against age-related retinal degeneration
Source: Commun Biol. 2025 May 26;8:812. doi: 10.1038/s42003-025-08242-1 (PMC12106806; doi:10.1038/s42003-025-08242-1)

OCT in Figure 1

Water treated

- NoTag
- R00
- L0R0
- L00

8-AG treated

- L0
- R0
- L0R00
- L00R0

8-AG treated group

L0-OD basline

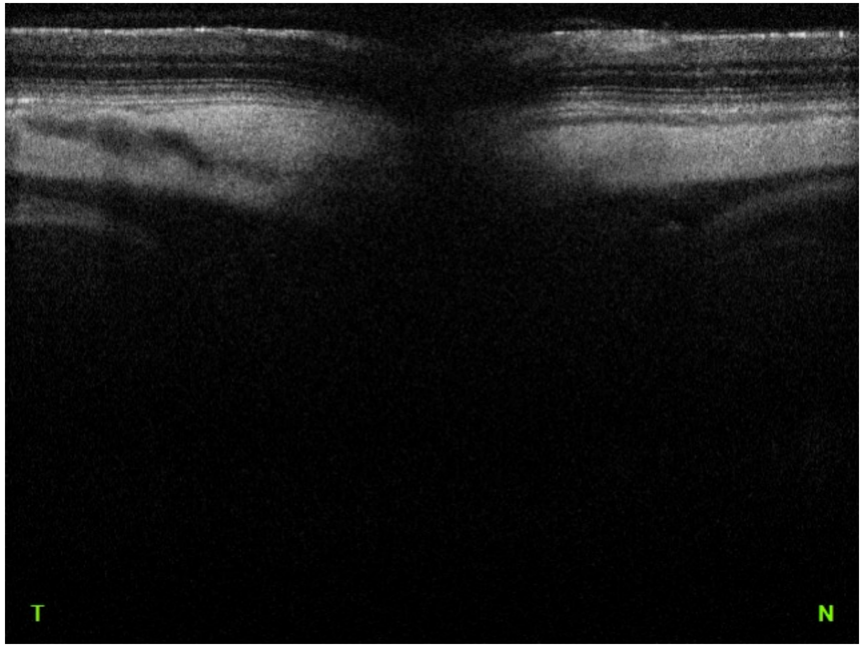

L0-OS basline

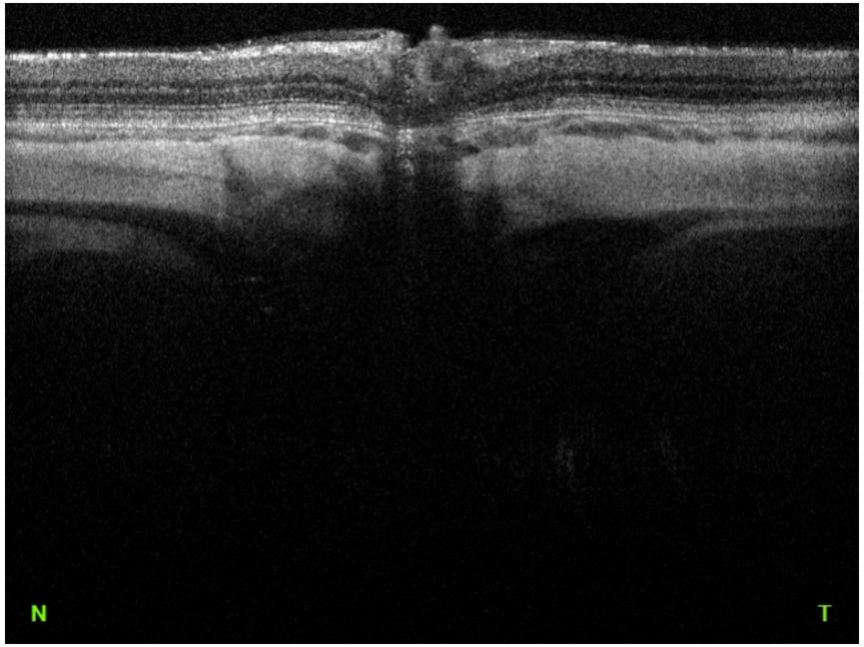

L0 OD 8 weeks

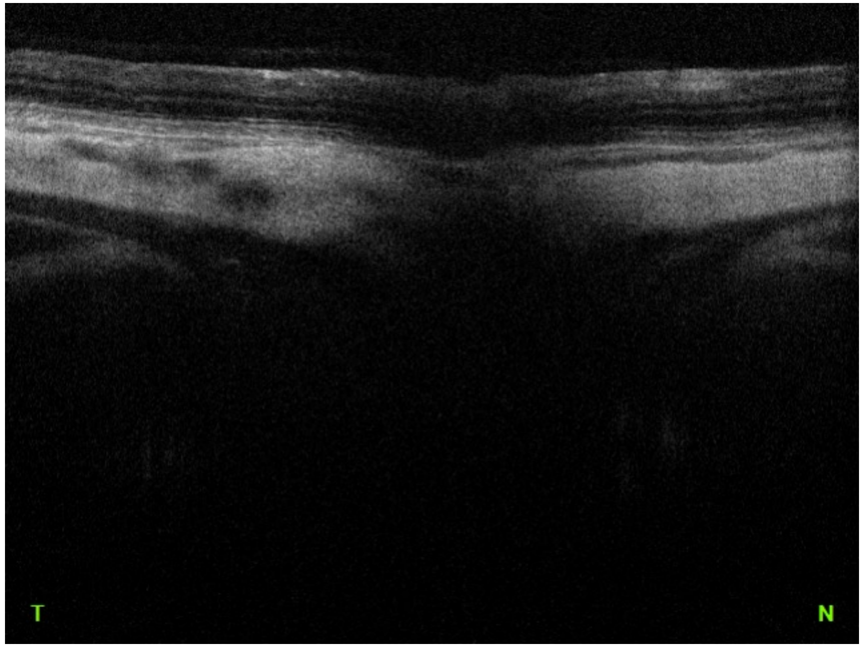

L0 OS 8 weeks

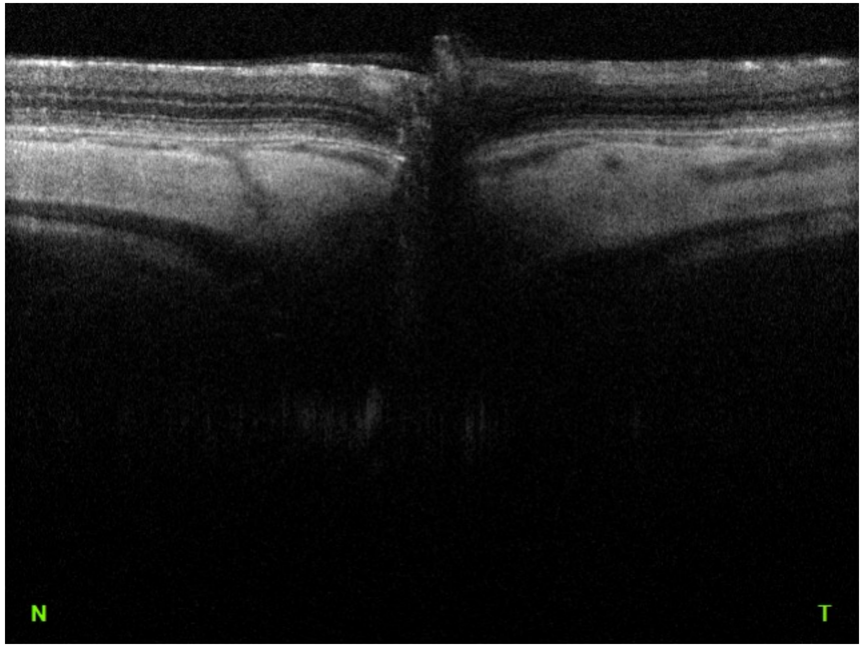

R0 OD baseline

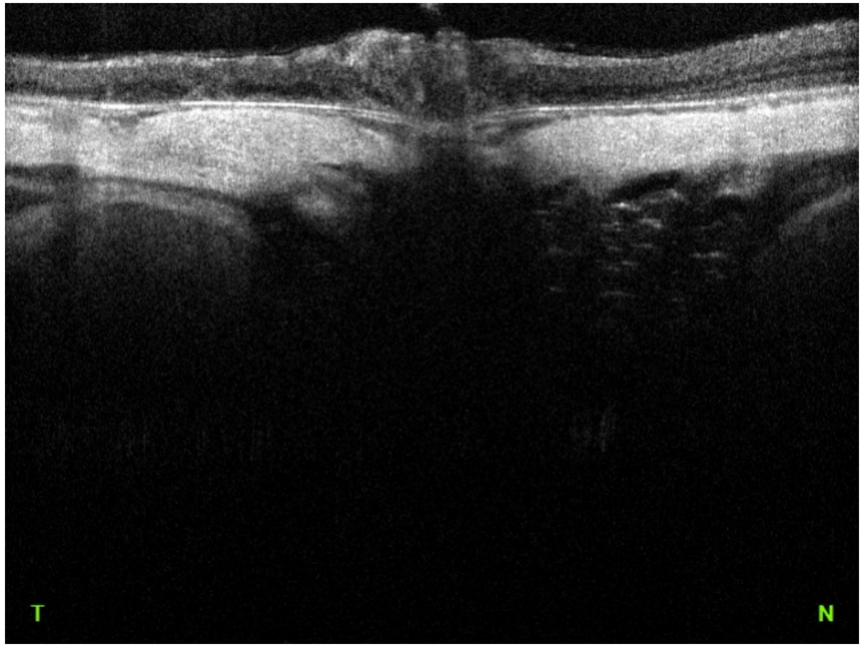

R0 OS baseline

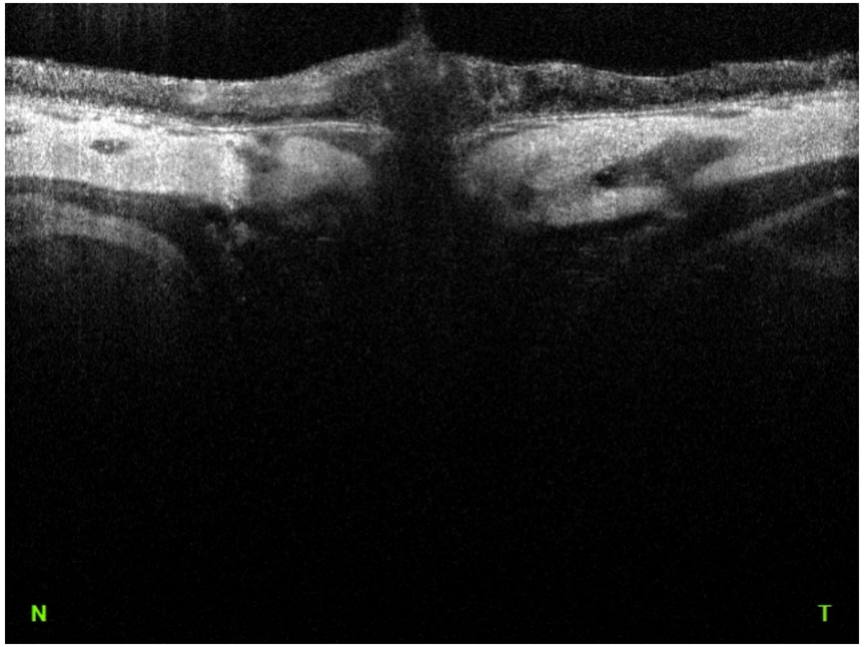

R0 OD 8 weeks

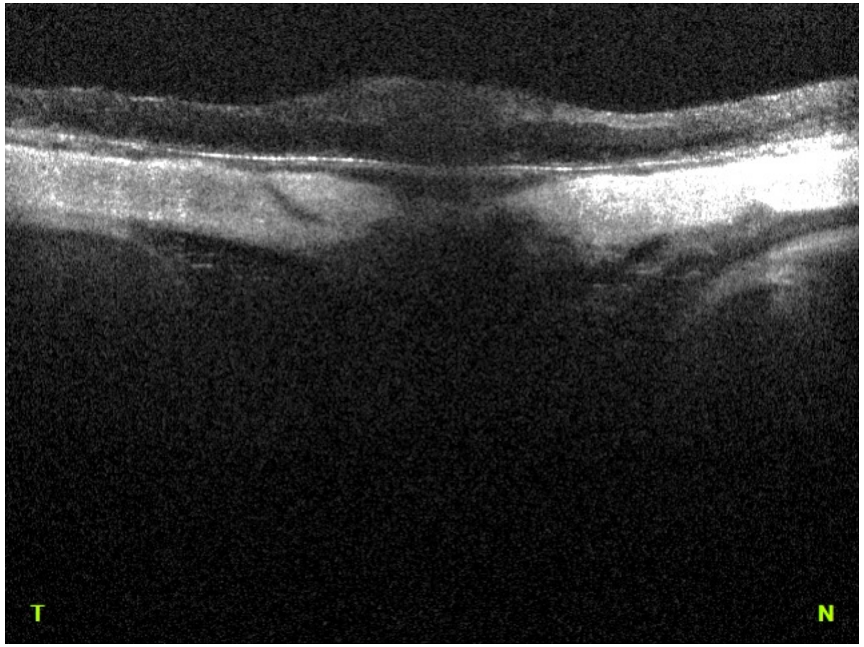

R0 OS 8 weeks

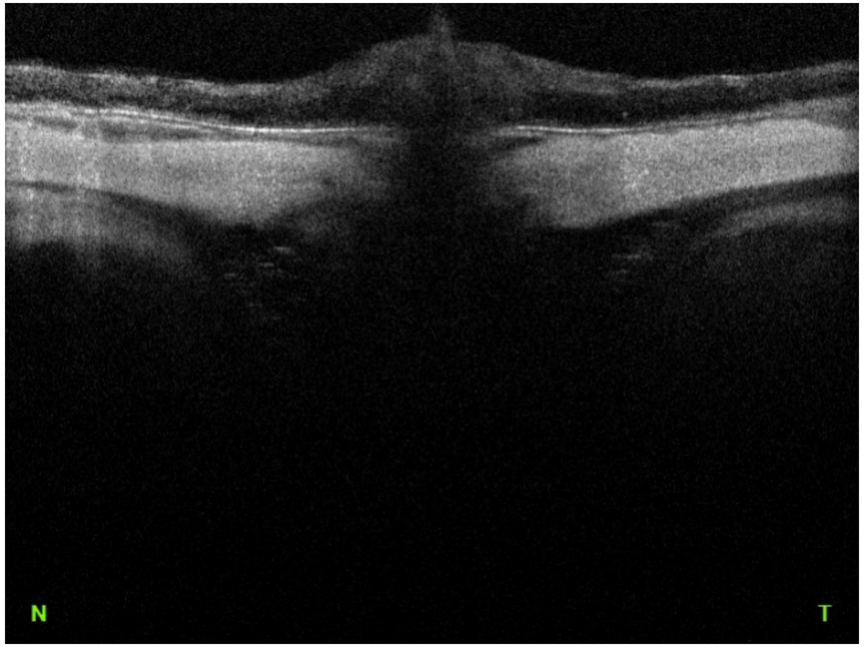

L0R00 OD baseine

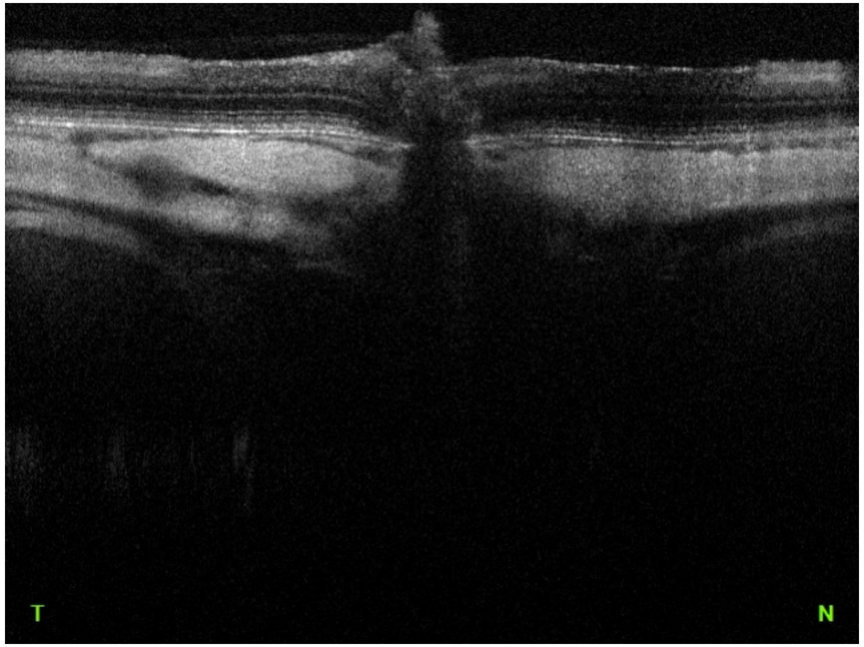

L0R00 OS baseine

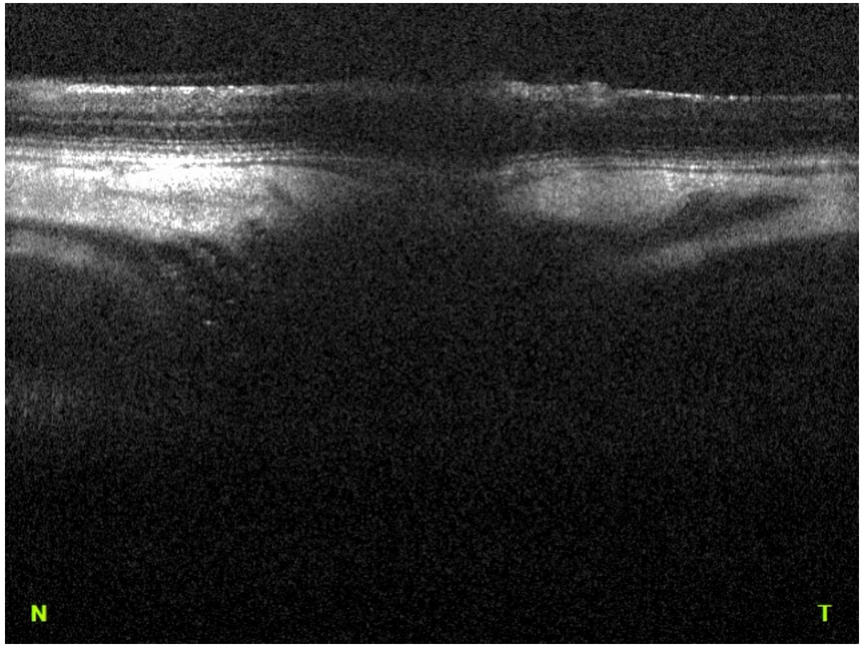

L0R00 OD 8 weeks

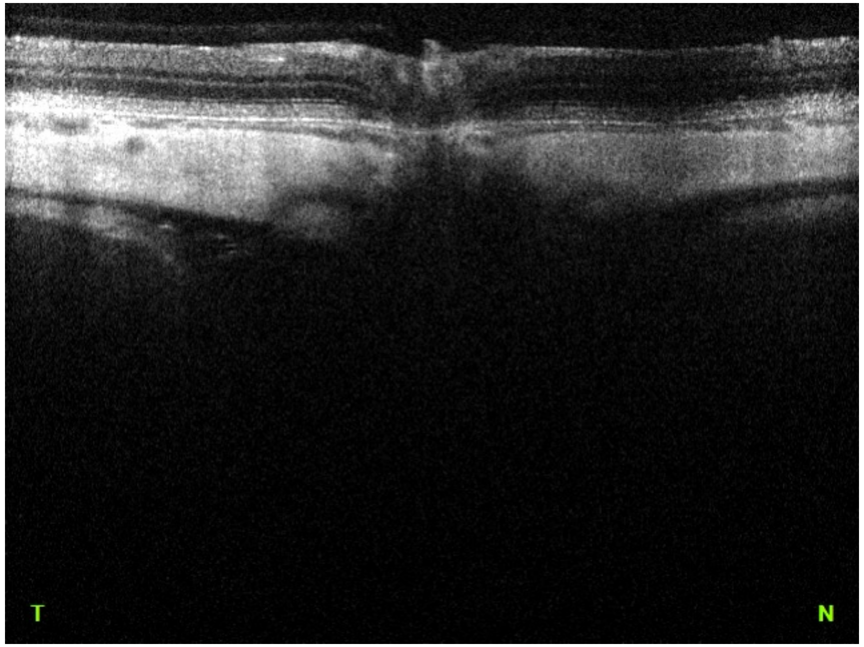

L0R00 OS 8 weeks

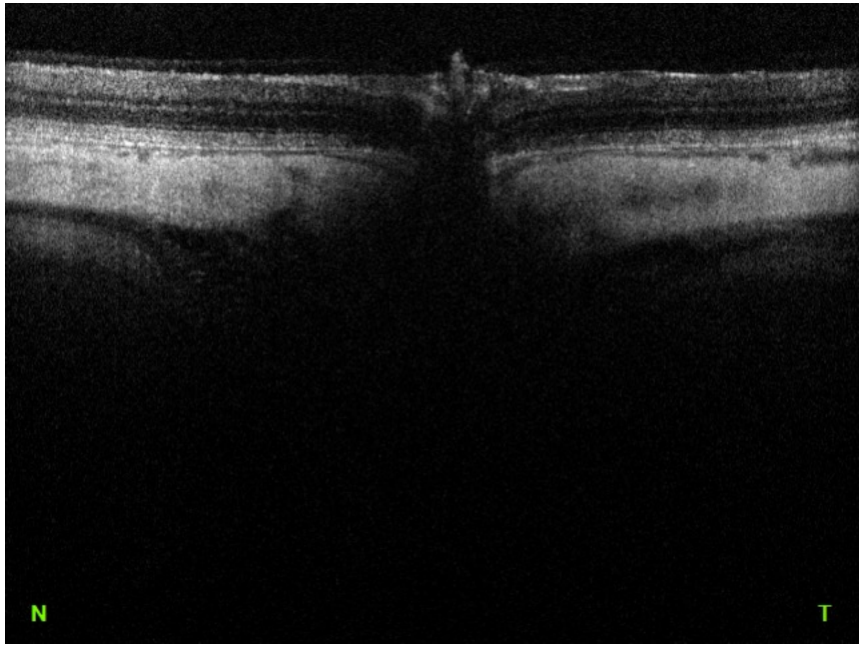

L00R0 OD baseline

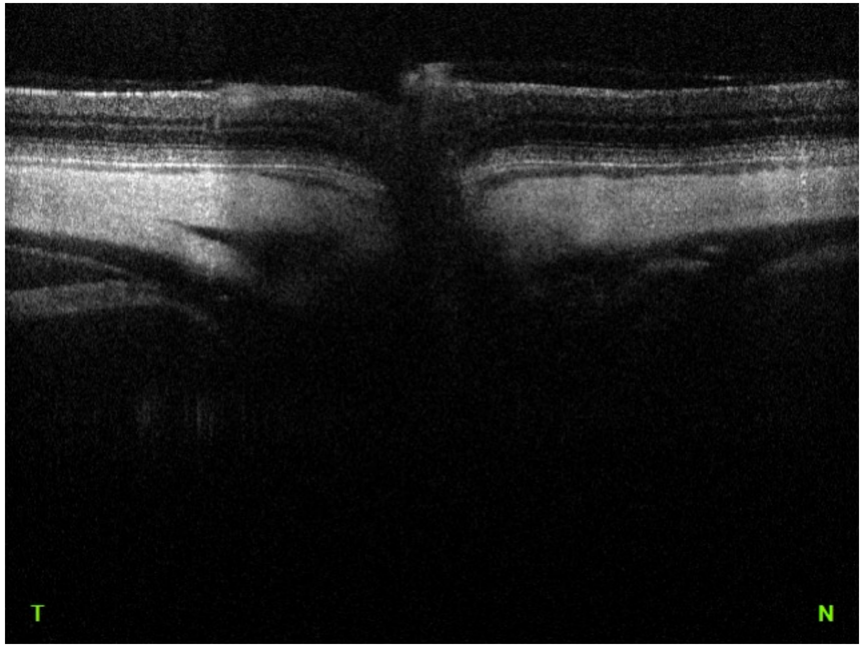

L00R0 OS baseline

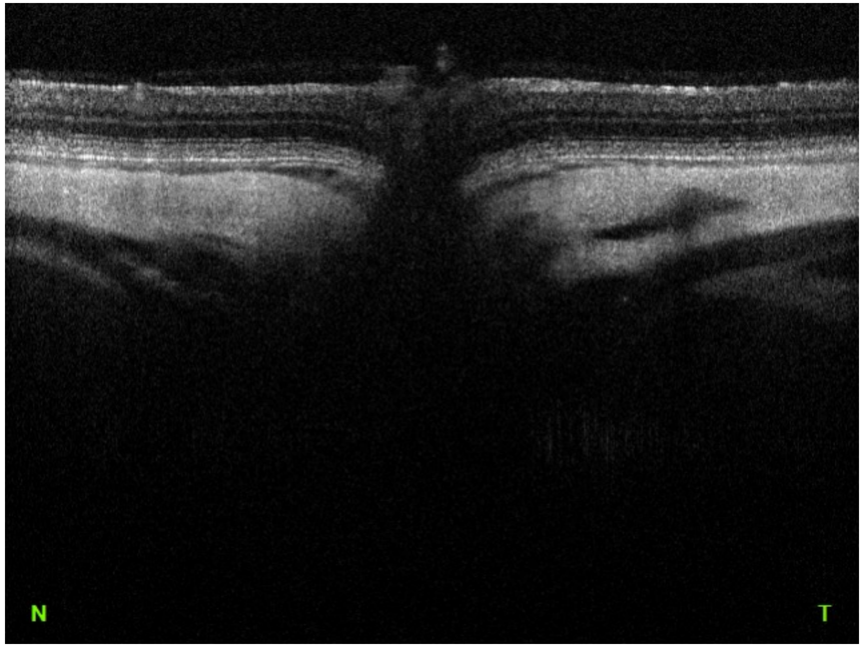

L00R0 OD 8 weeks

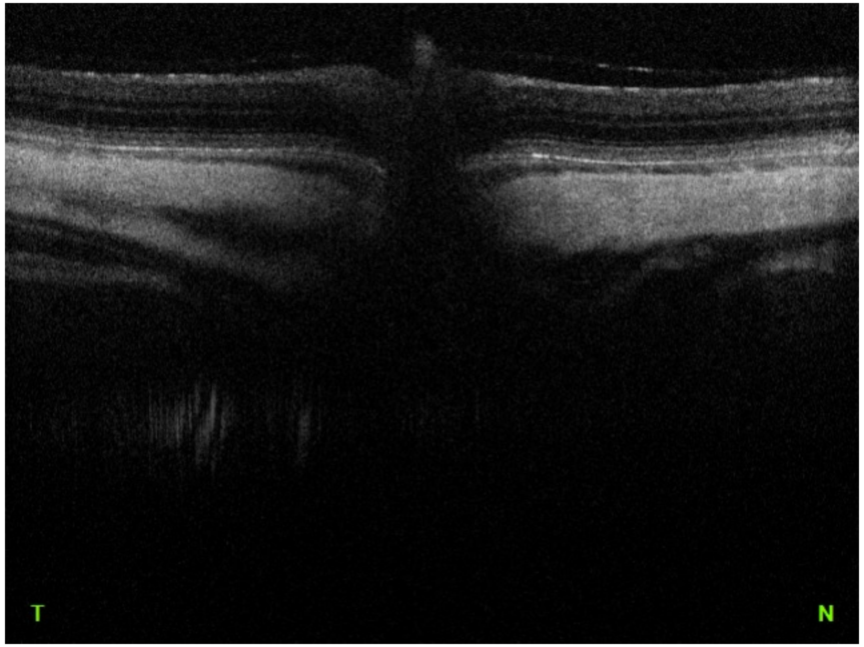

L00R0 OS 8 weeks

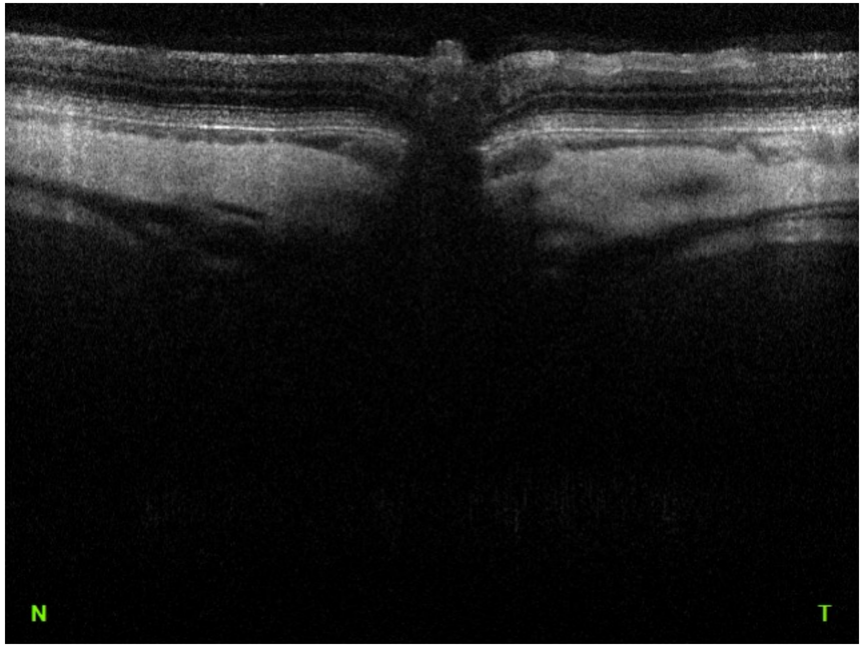

Water-treated group

NoTag OD baseline

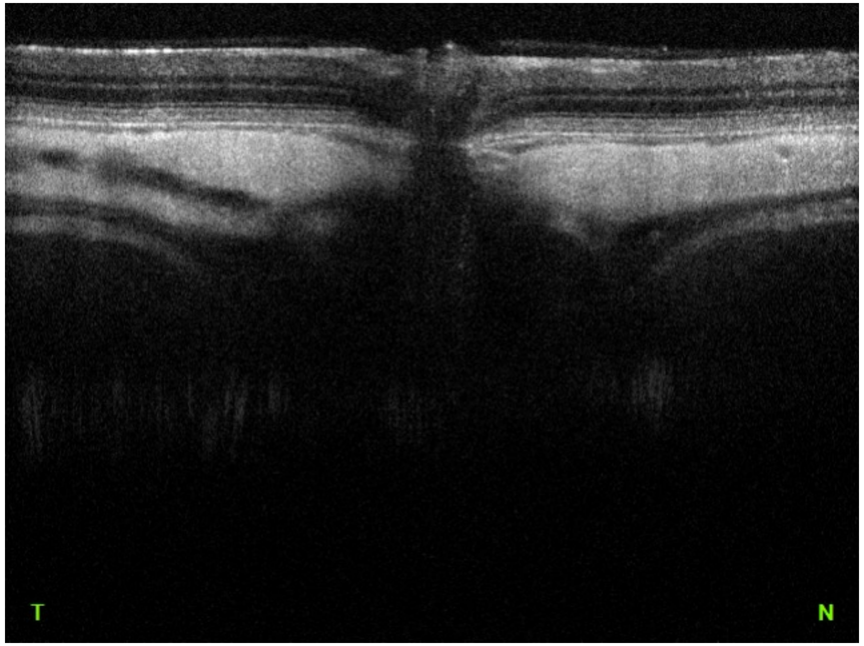

NoTag OS baseline

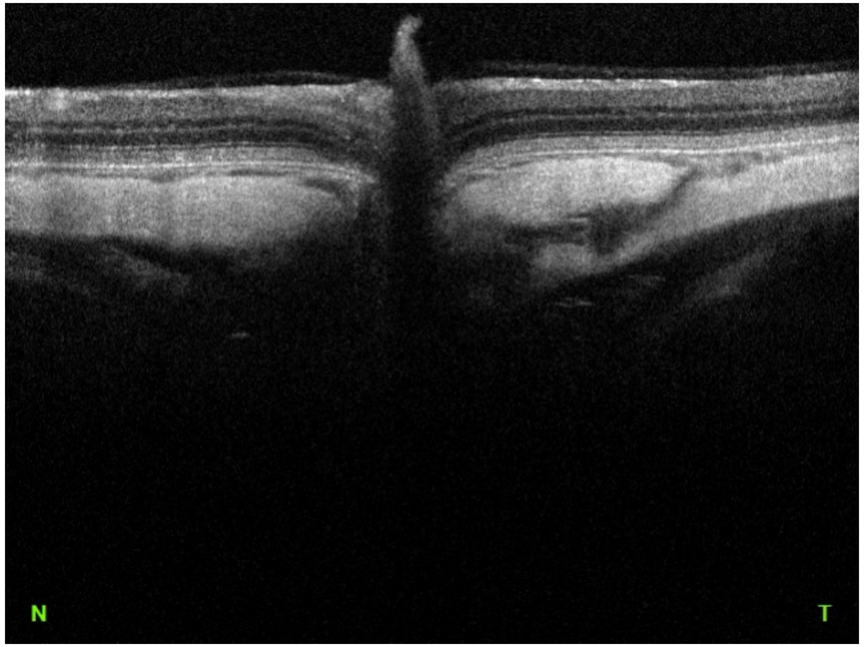

NoTag OD 8 weeks

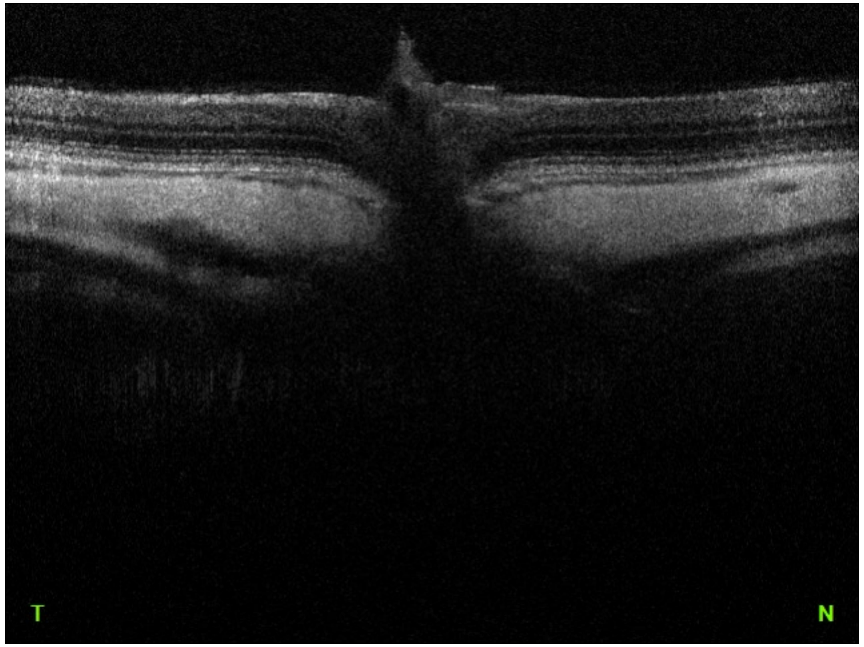

NoTag OS 8 weeks

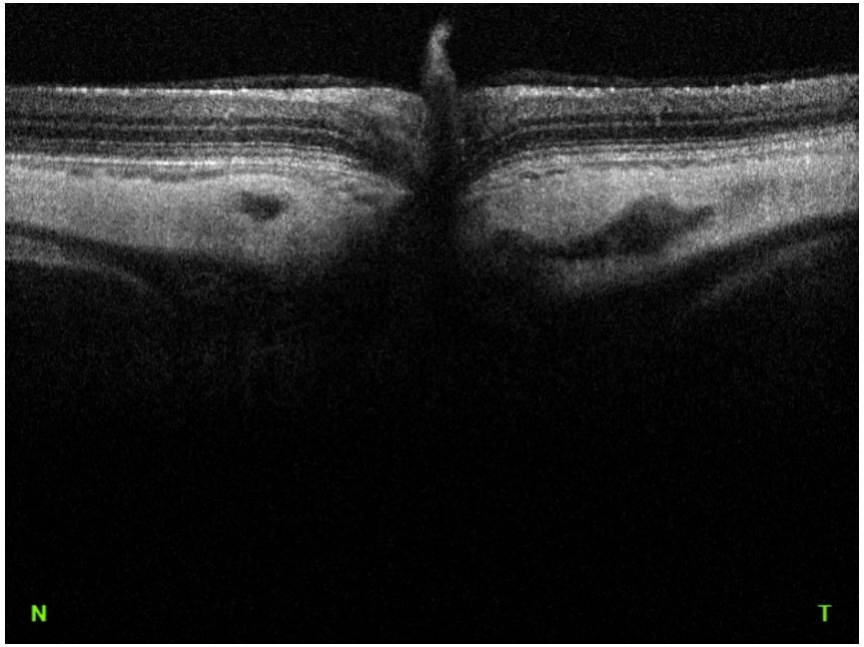

R00 OD baseline

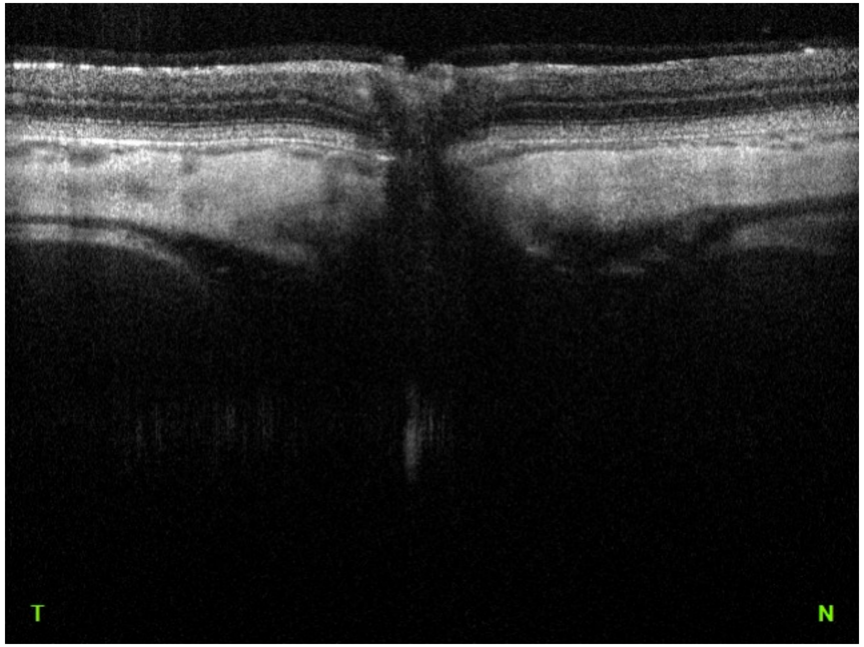

R00 OS baseline

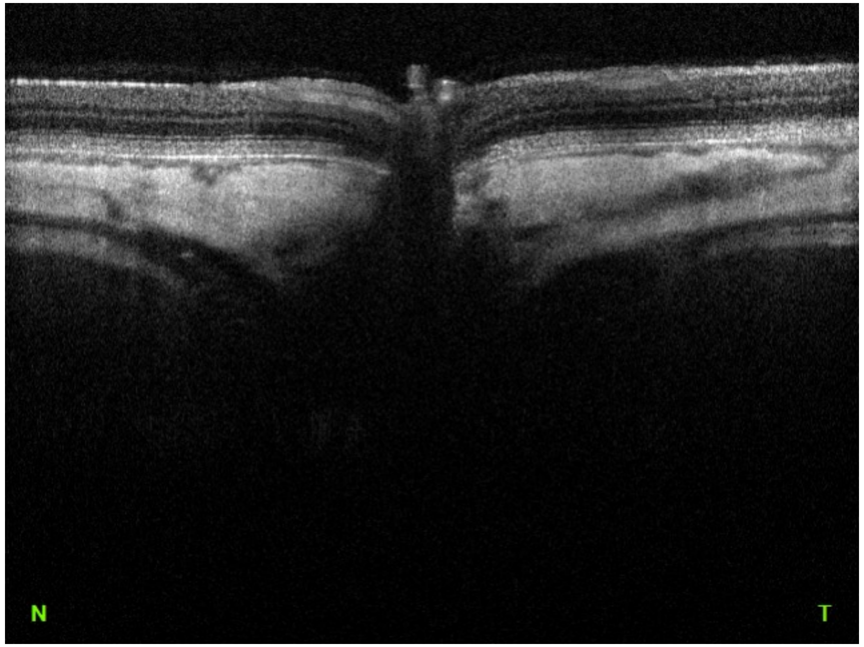

R00 OD 8 weeks

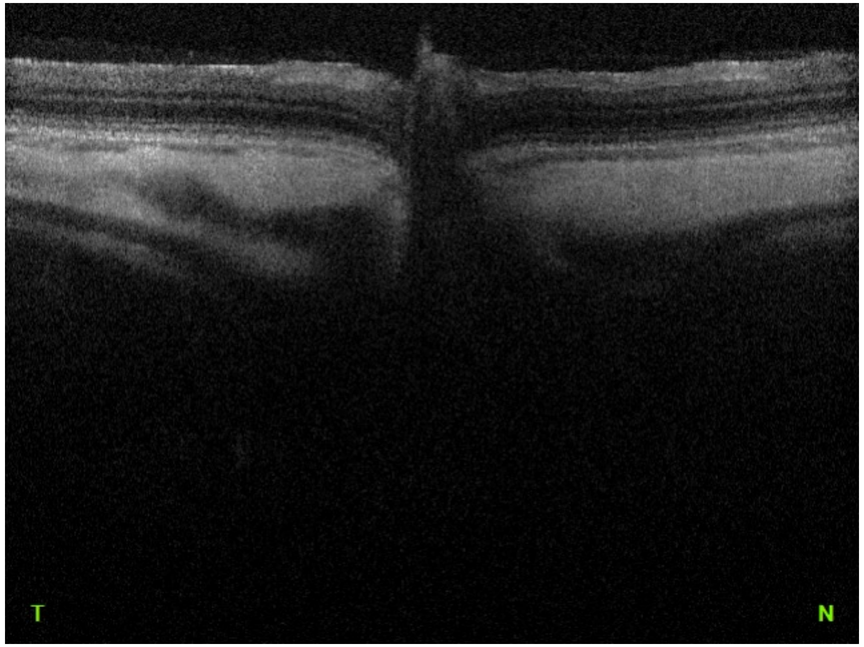

R00 OS 8 weeks

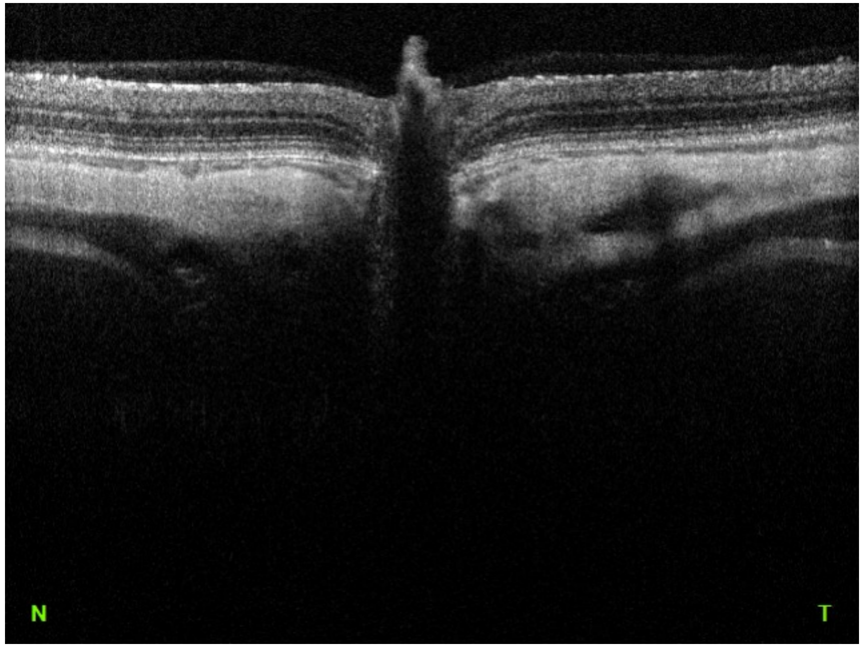

L0R0 OD baseline

L0R0 OS baseline

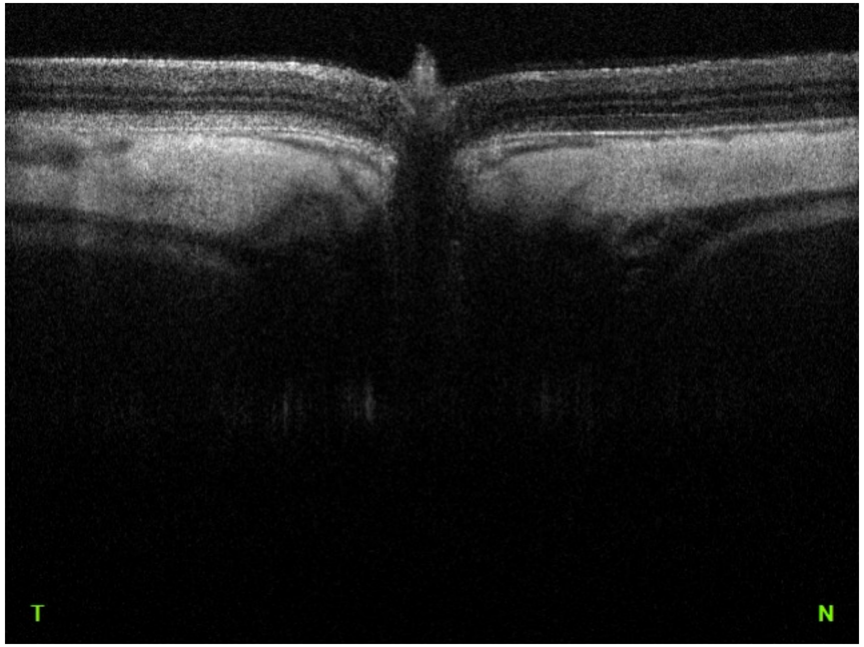

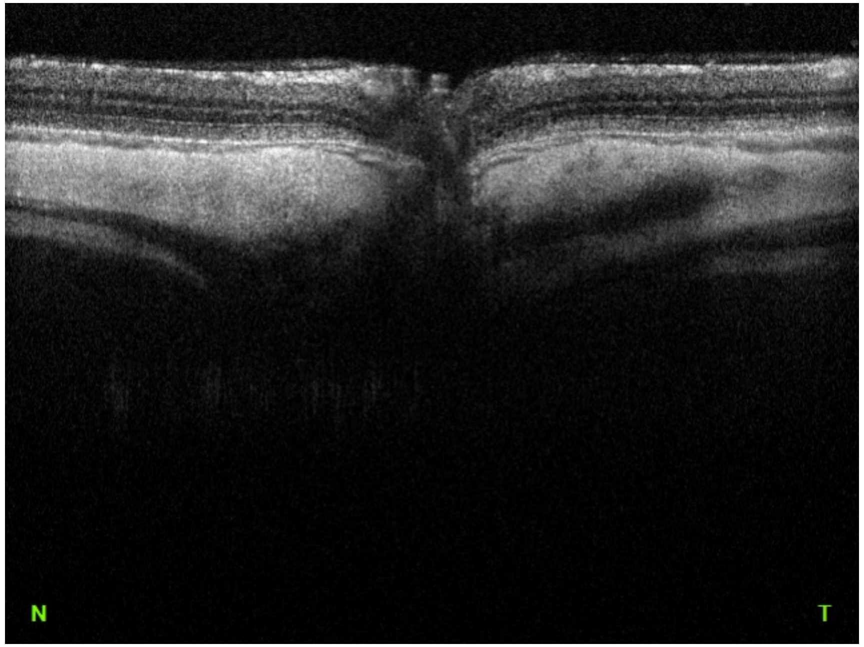

L0R0 OD 8 weeks

L0R0 OS 8 weeks

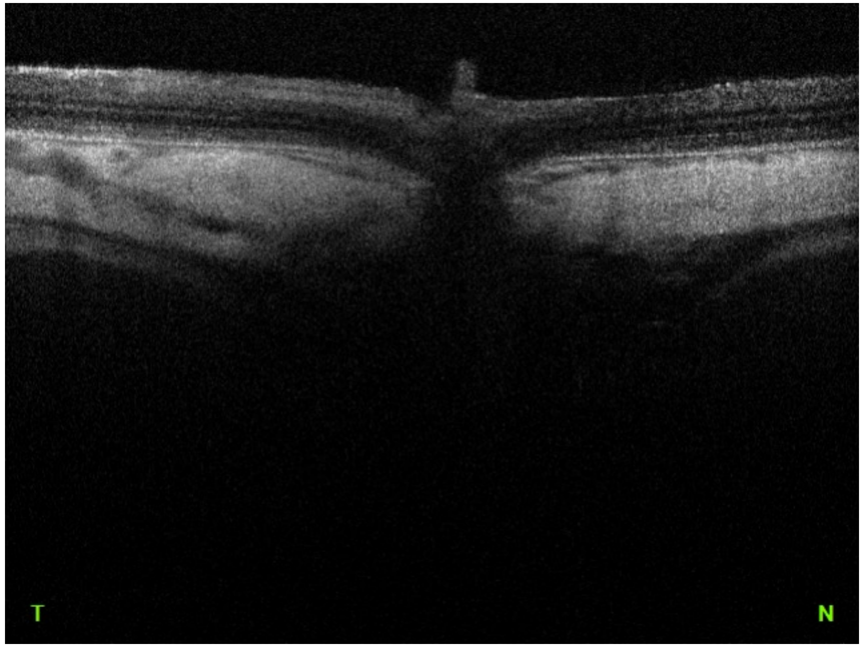

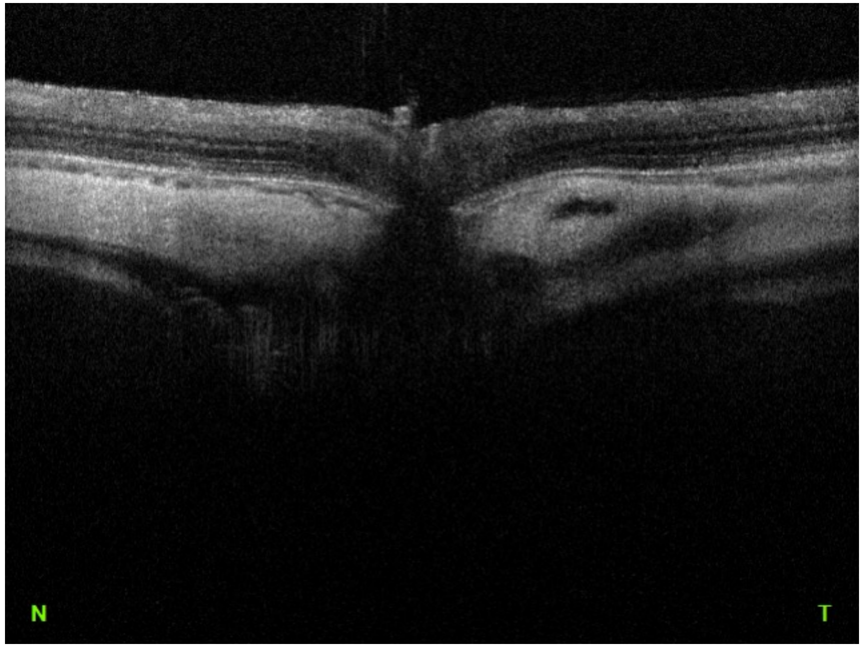

L00-OD Baseline

L00-OS Baseline

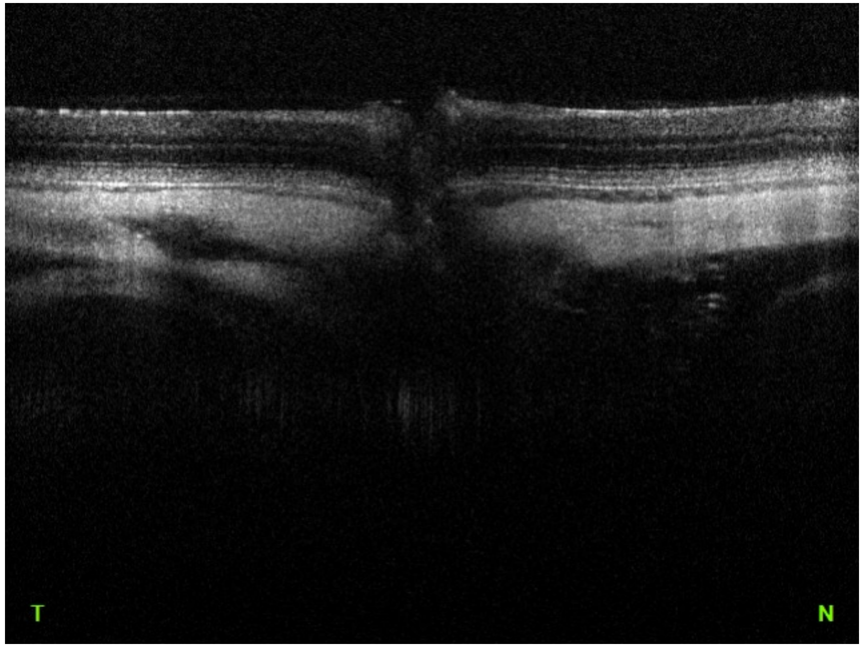

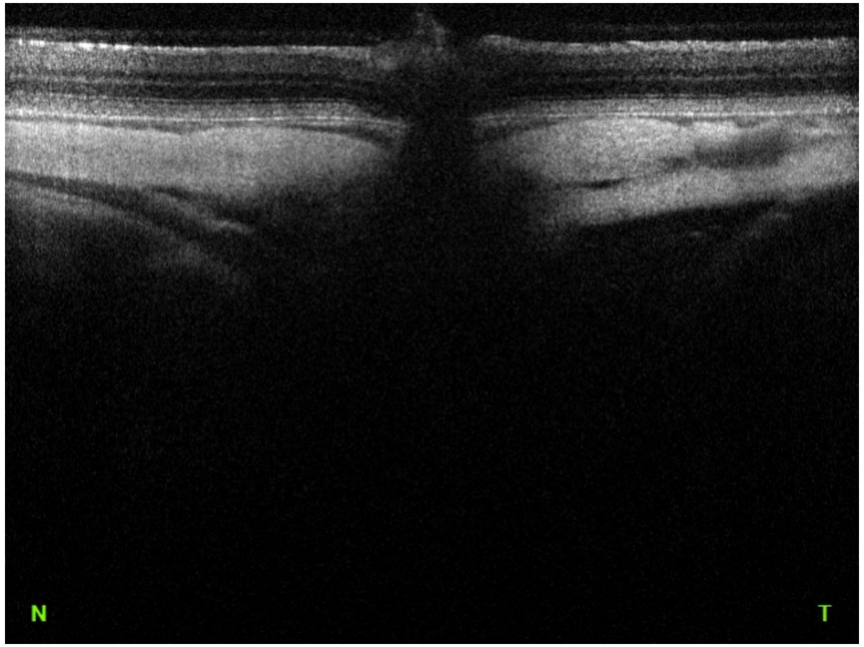

L00-OD 8 weeks

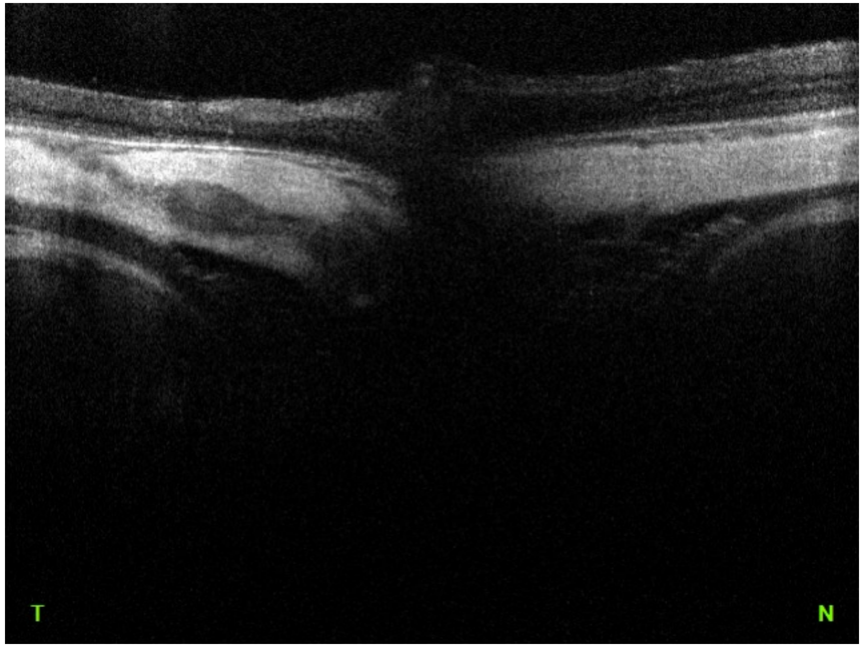

L00-OS 8 weeks

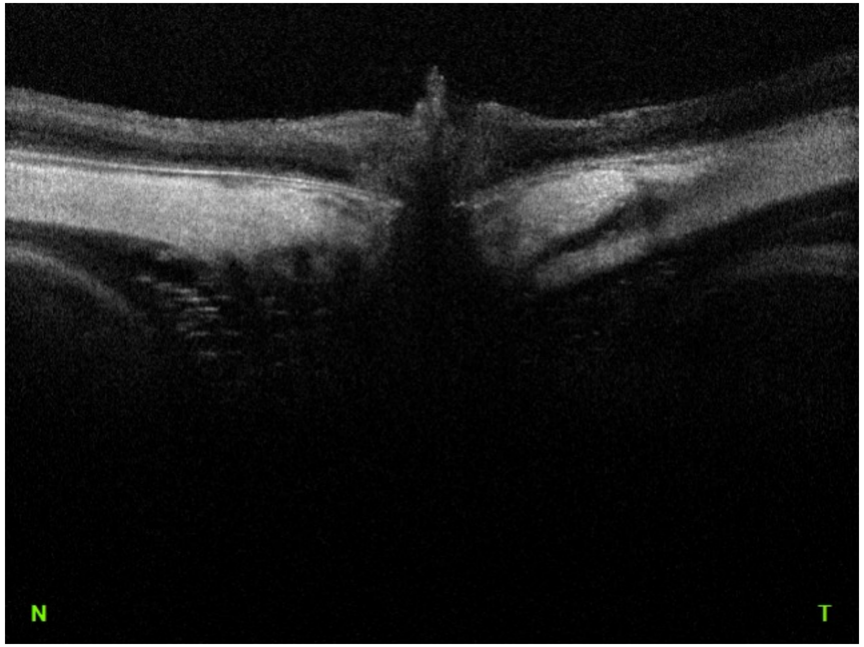

Supplement: Supplementary file 3 — Supplementary Data 1 [file 42003_2025_8242_MOESM3_ESM.pdf]
