# Supplementary material for: Oral 8-aminoguanine against age-related retinal degeneration
Source: Commun Biol. 2025 May 26;8:812. doi: 10.1038/s42003-025-08242-1 (PMC12106806; doi:10.1038/s42003-025-08242-1)

F344 rat retinal histology data for Figure 1K-M

Young rat retinae 1-3

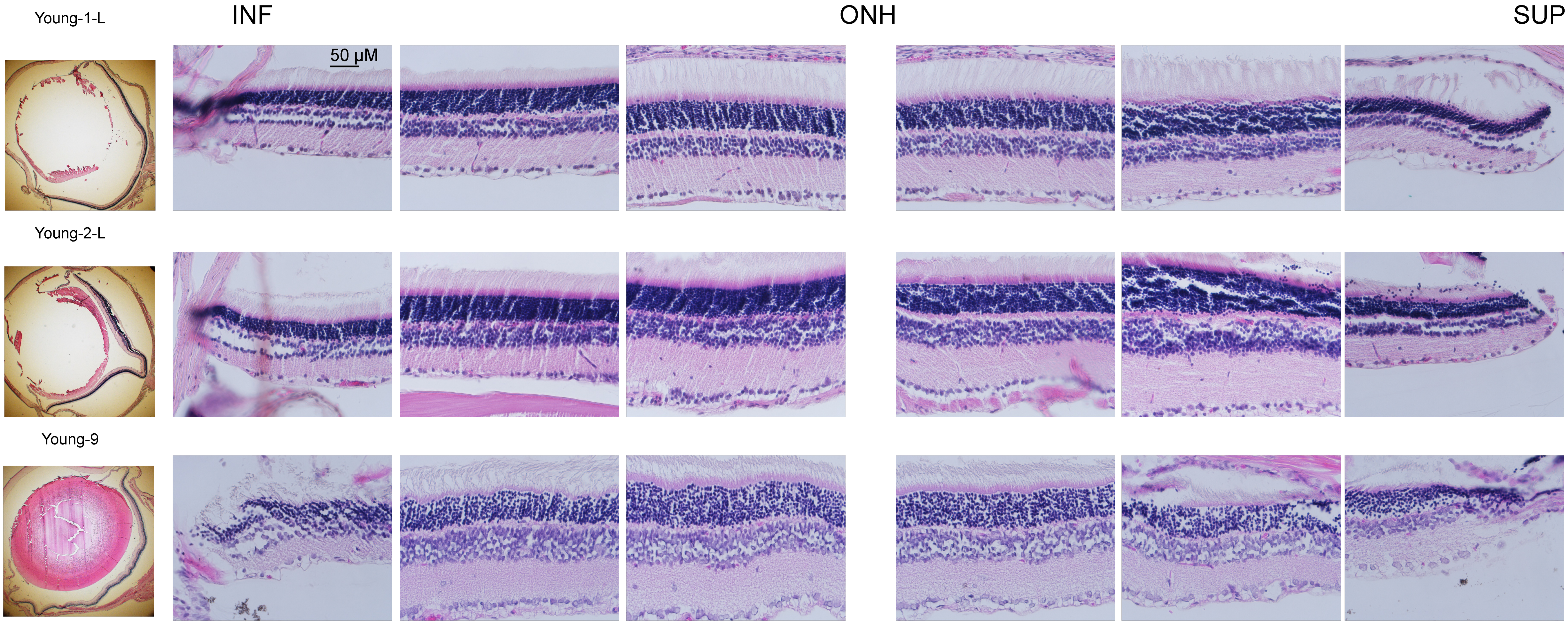

Young rat retinae 4-6

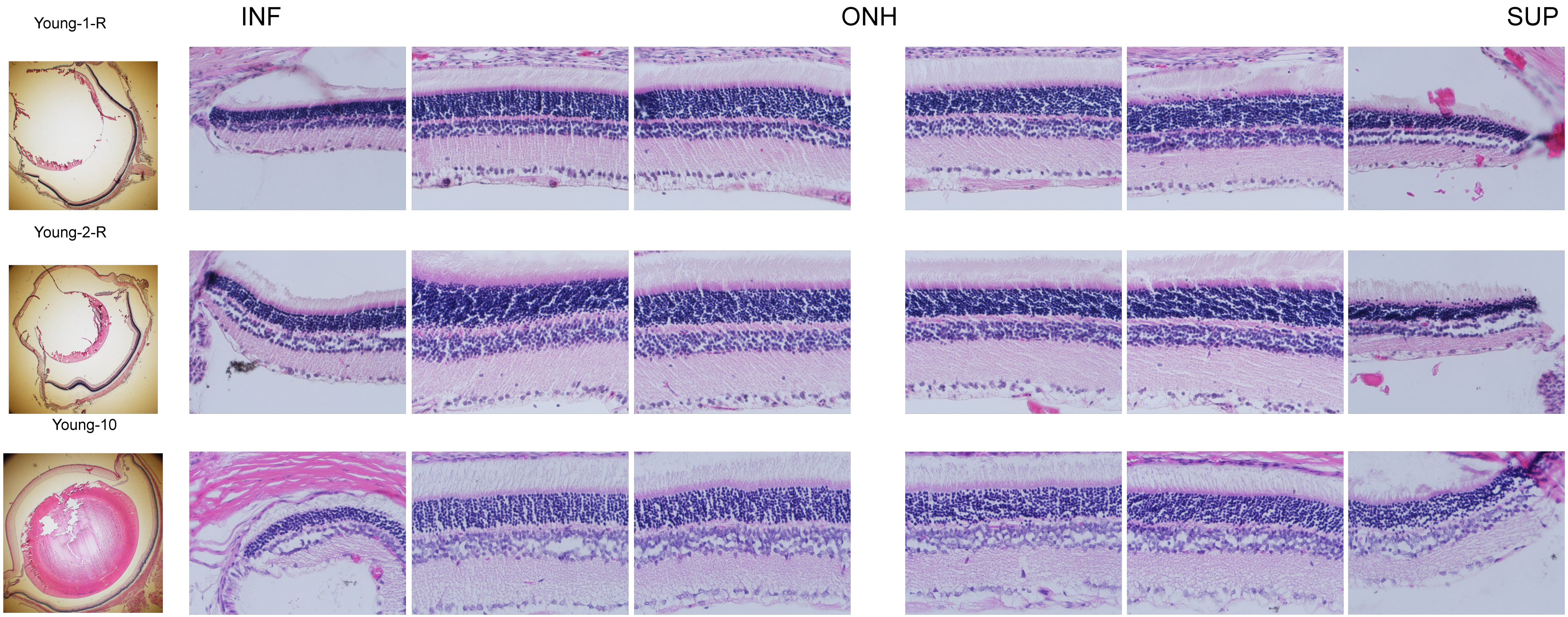

Water-treated aged rat retinae 1-3

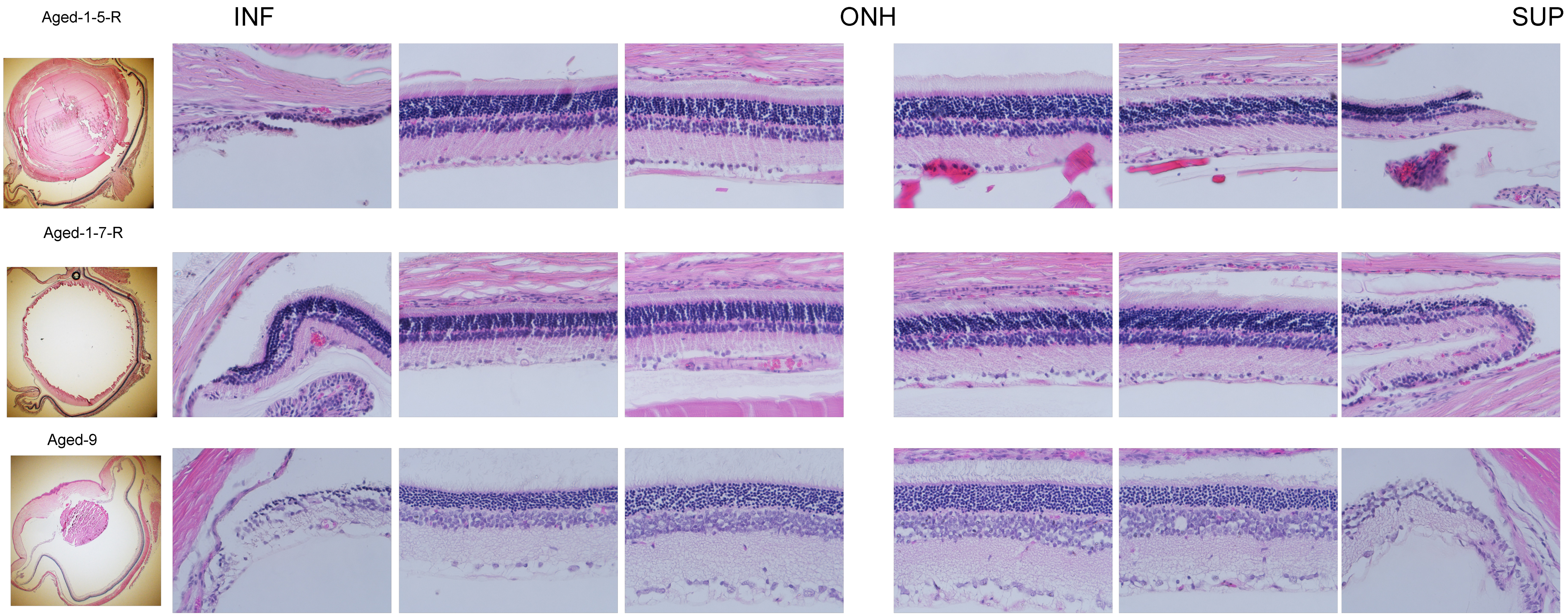

Water-treated aged rat retinae 4-6

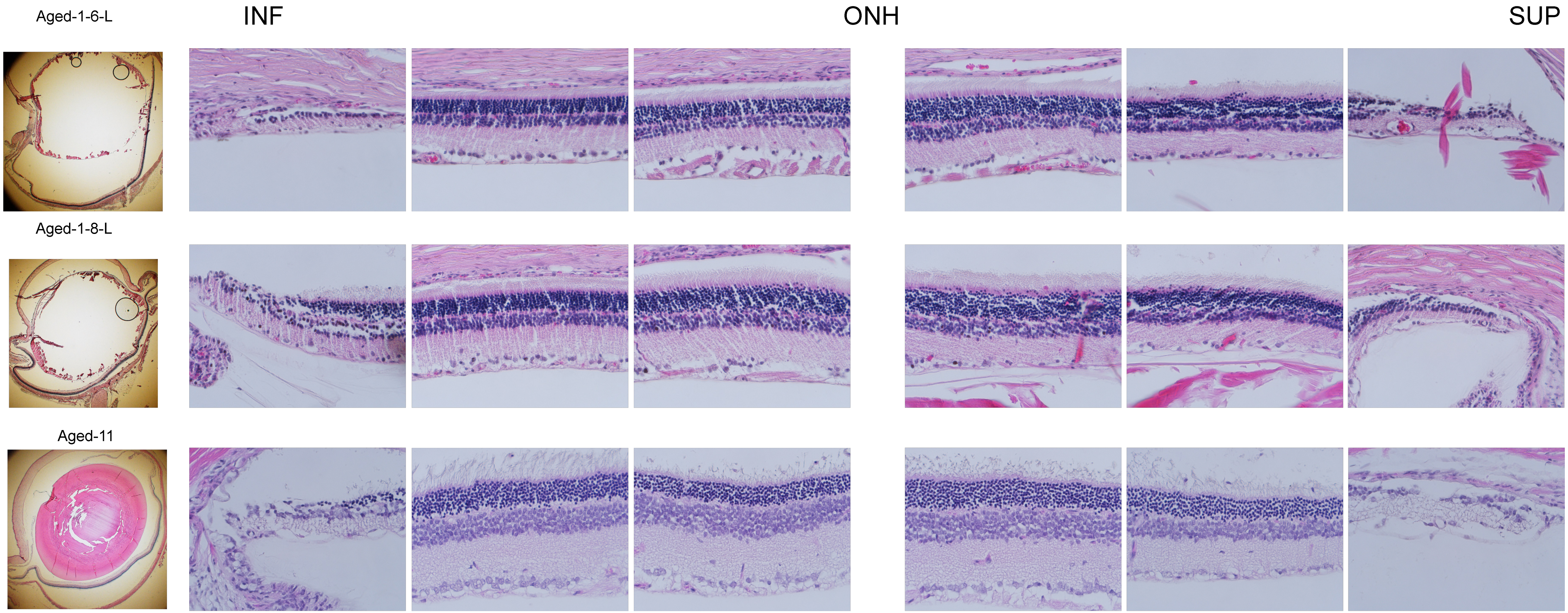

8-AG-treated aged rat retinae 1-3

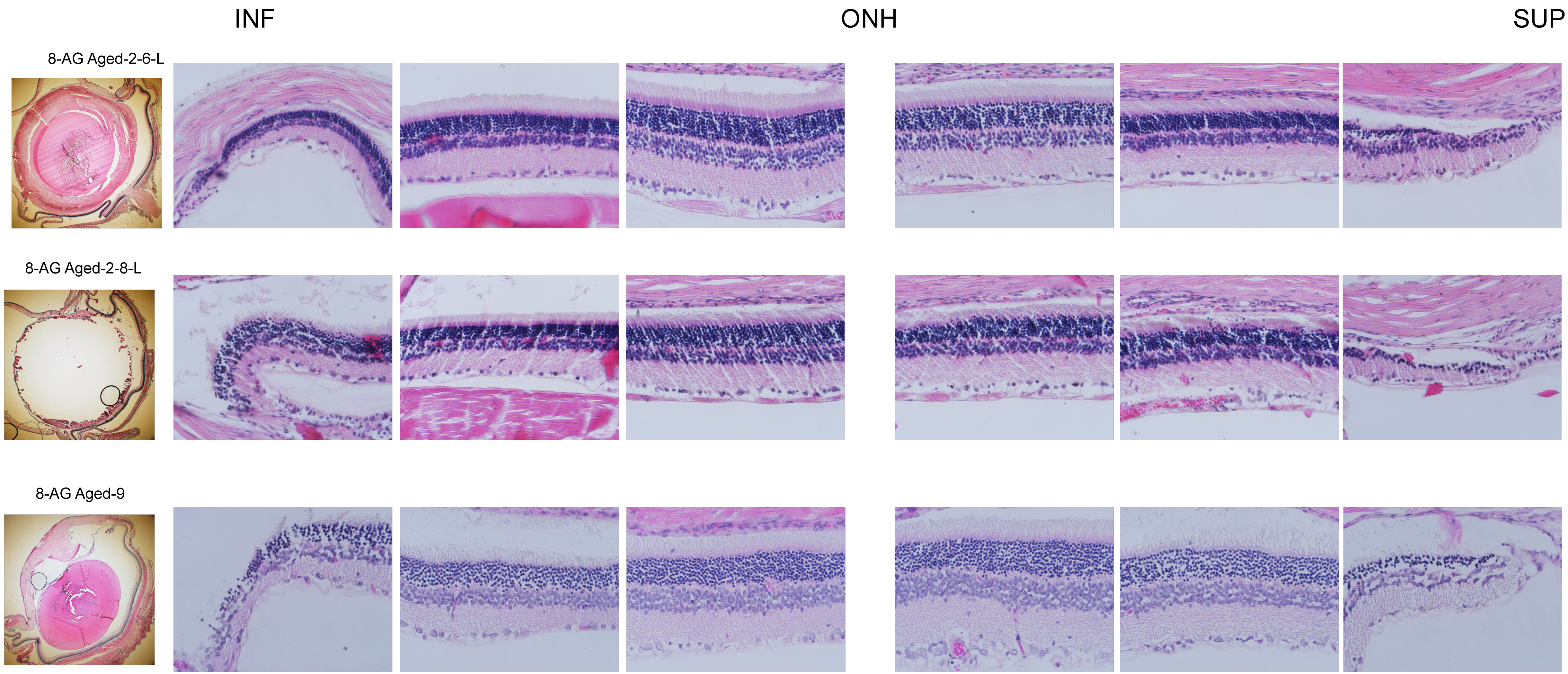

8-AG-treated aged rat retinae 4-6

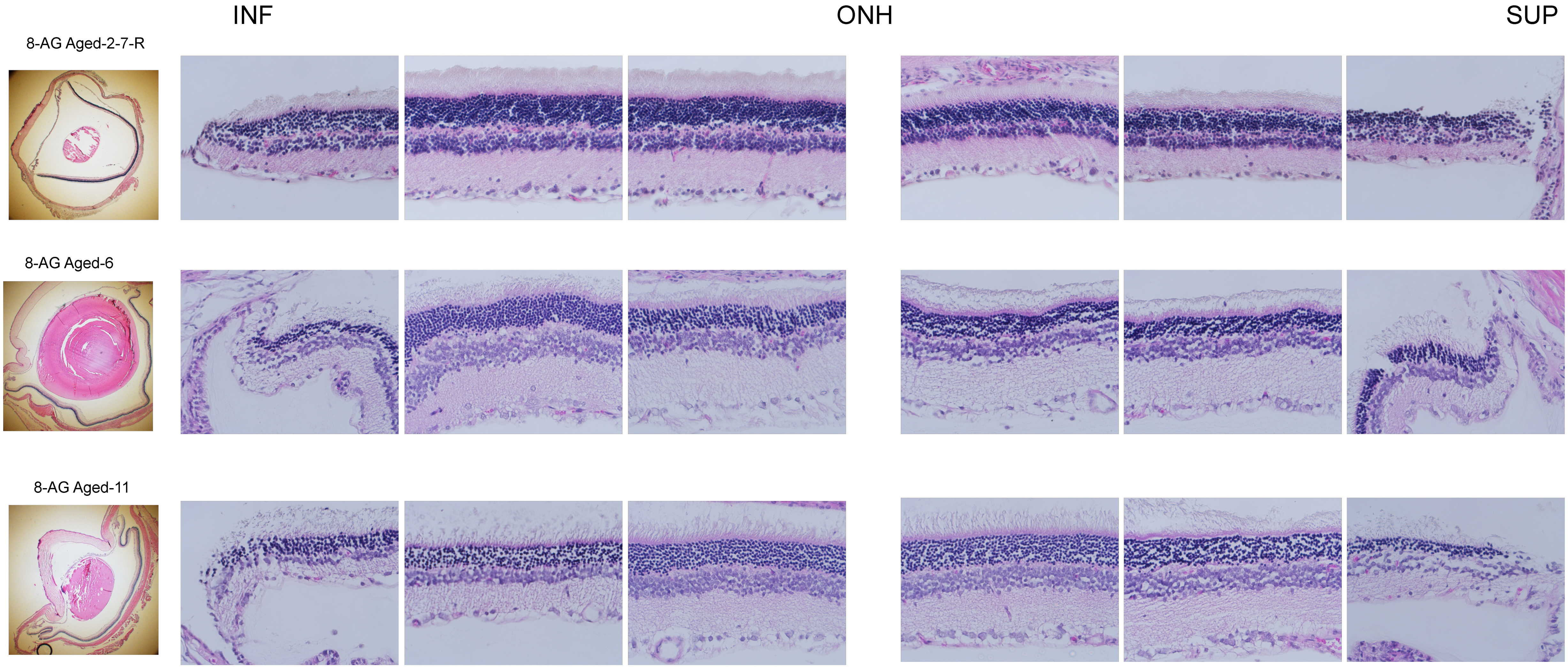

Supplement: Supplementary file 4 — Supplementary Data 2 [file 42003_2025_8242_MOESM4_ESM.pdf]
