# Supplementary material for: Oral 8-aminoguanine against age-related retinal degeneration
Source: Commun Biol. 2025 May 26;8:812. doi: 10.1038/s42003-025-08242-1 (PMC12106806; doi:10.1038/s42003-025-08242-1)

TUNEL staining for Figure 2A-D

Controls for TUNEL staine

Green, TUNEL stain; blue, Hoechst33342

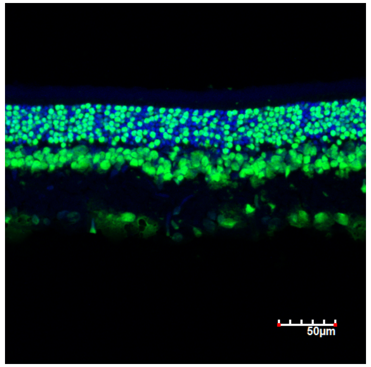

Positive control

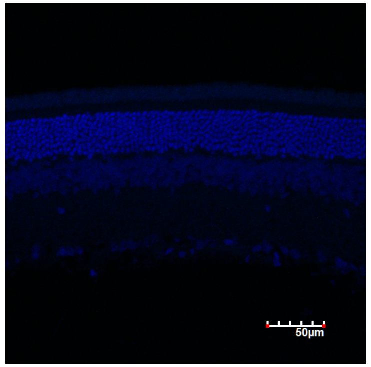

Secondary only control

Young F344 rat retinae (Biol. repeat 1-2)

Green, TUNEL stain; blue, Hoechst33342

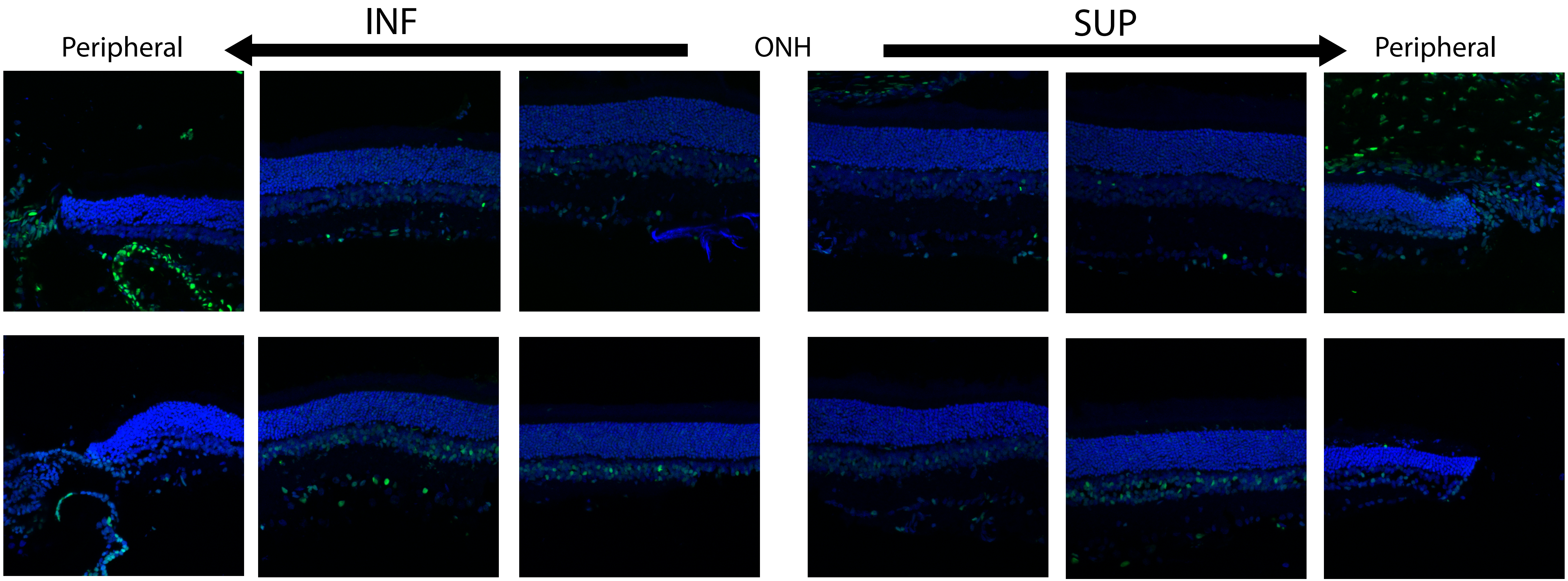

Young F344 rat retinae (Biol. repeat 3-4)

Green, TUNEL stain; blue, Hoechst33342

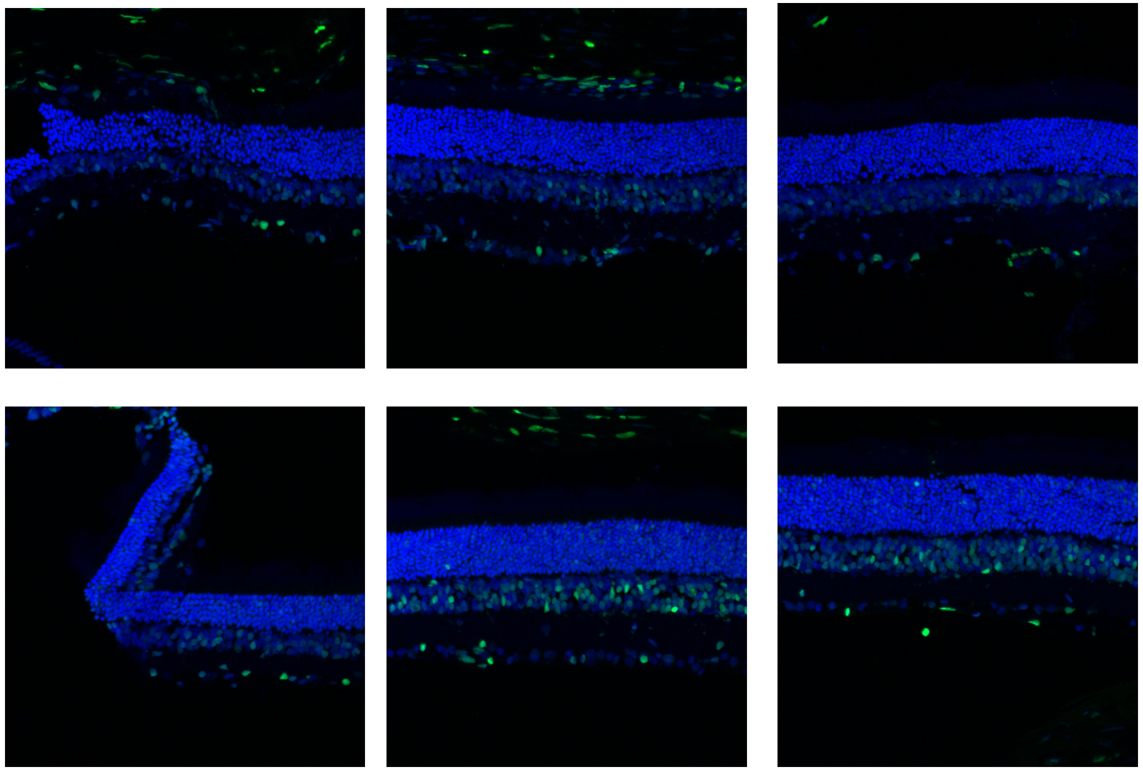

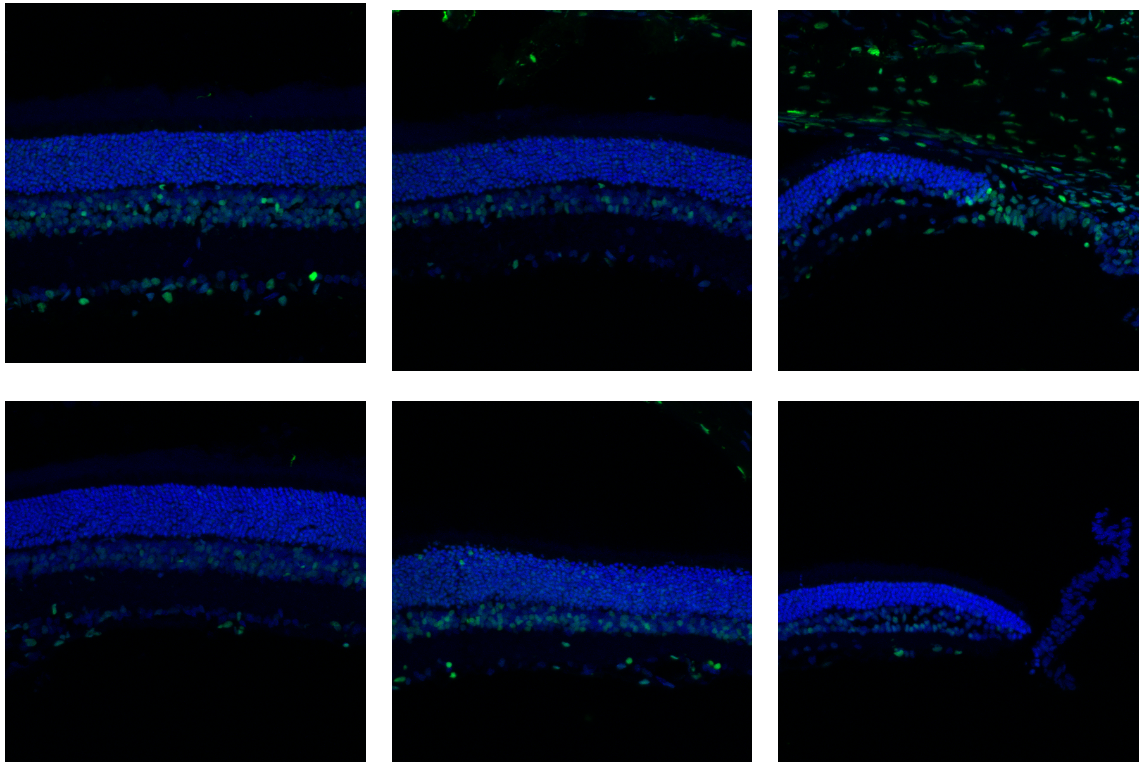

Aged water-treated F344 rat retinae (Biol. Repeat 1-2)

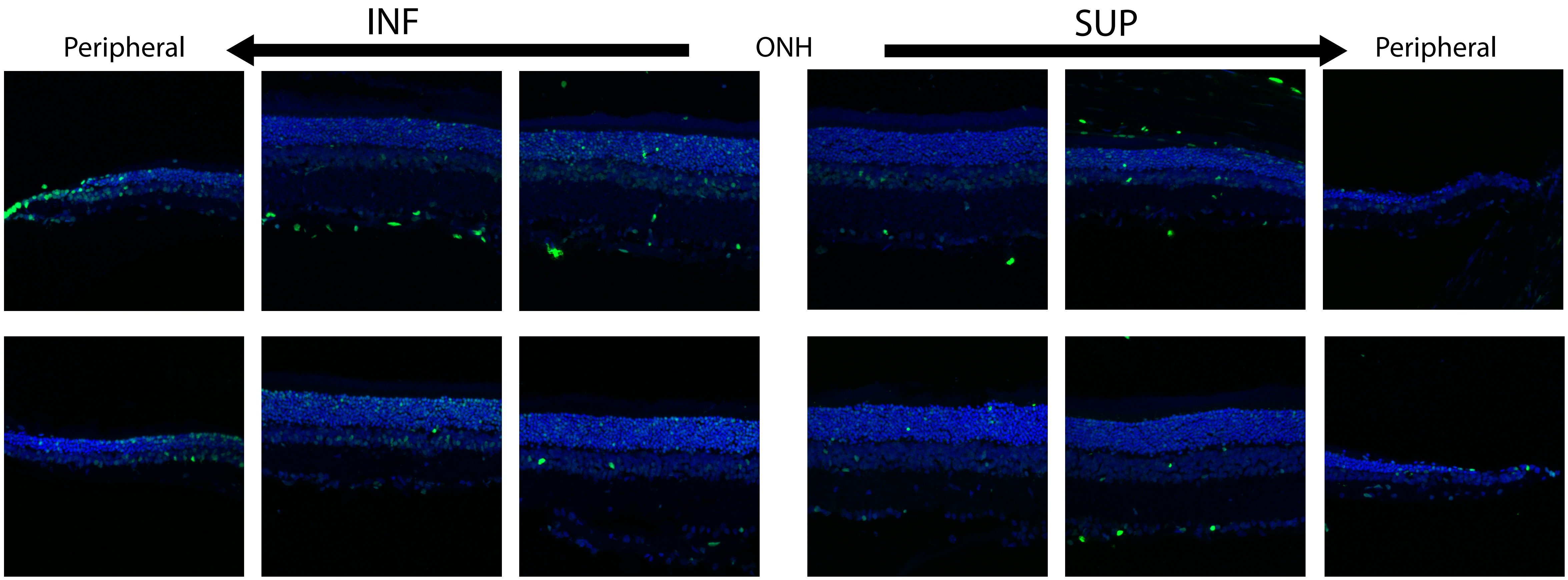

Aged water-treated F344 rat retinae (Biol. Repeat 3-4)

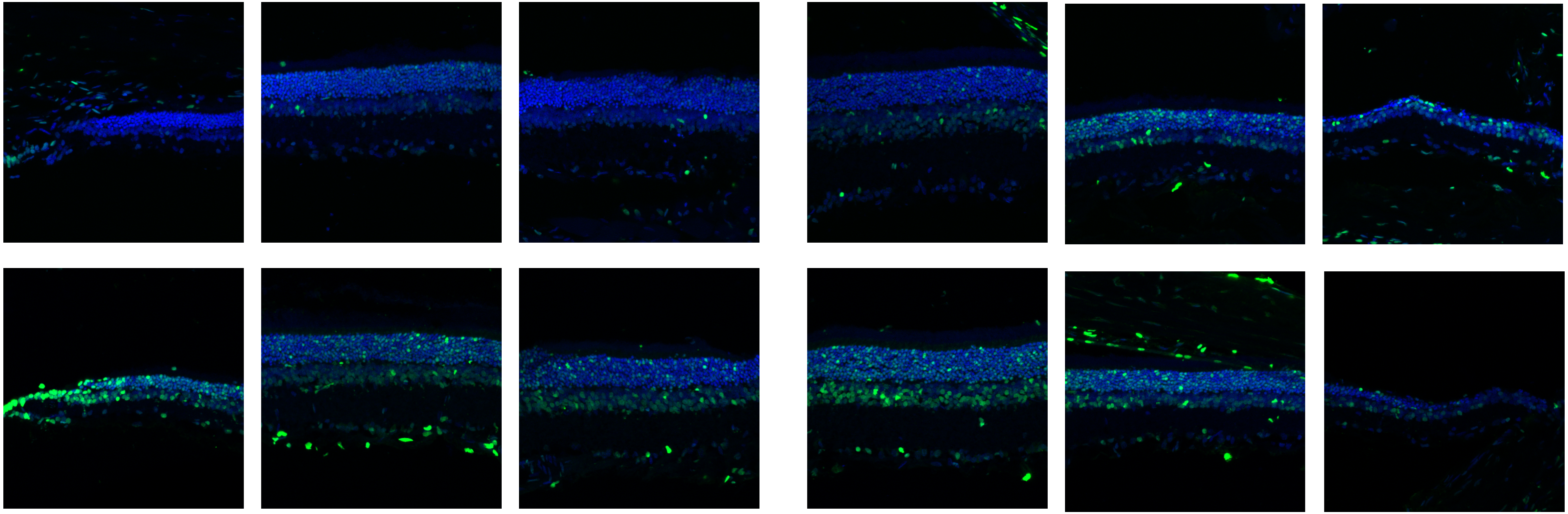

Aged 8AG-treated F344 rat retinae (Biol. Repeat 1-3)

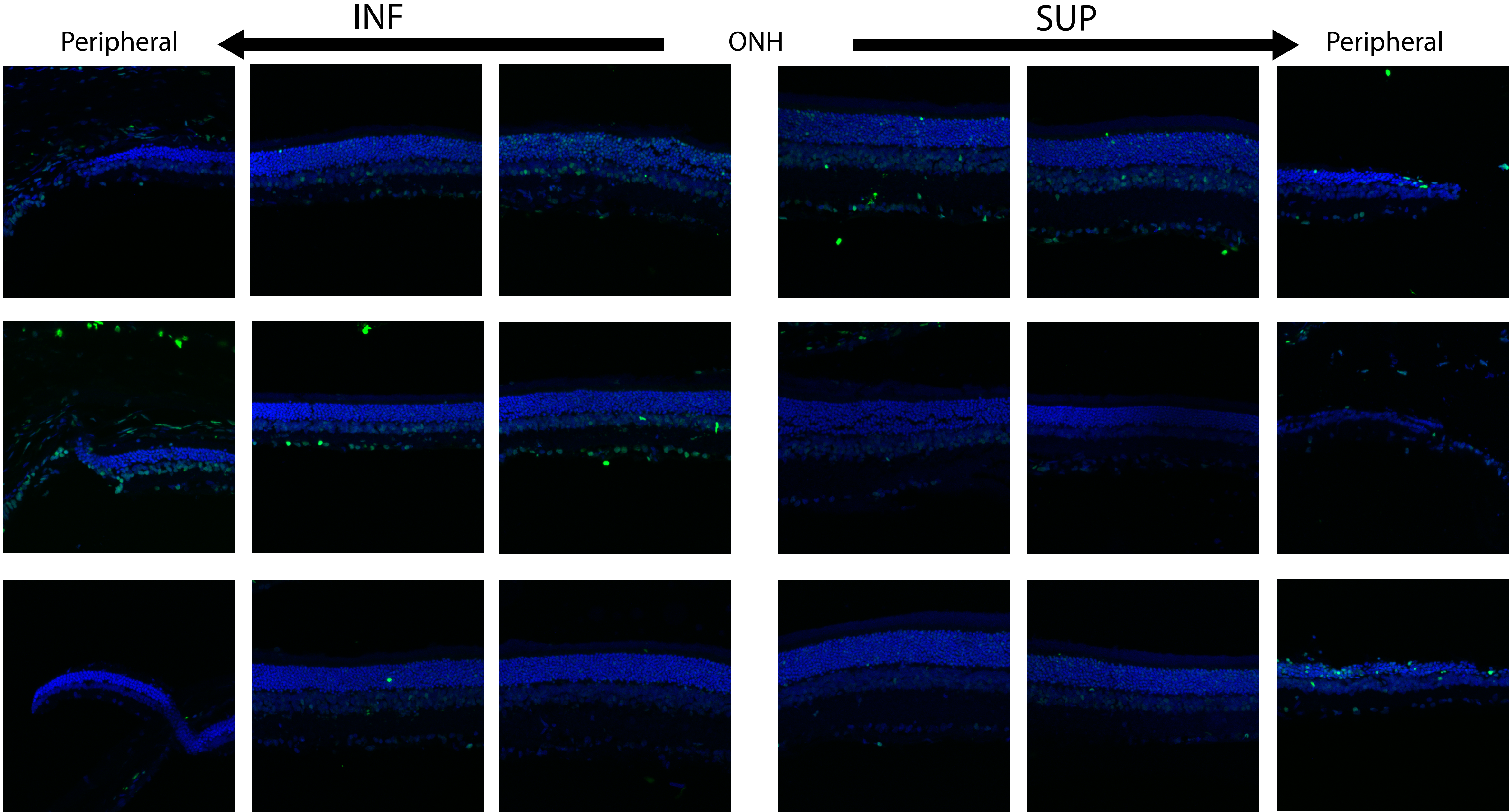

Supplement: Supplementary file 5 — Supplementary Data 3 [file 42003_2025_8242_MOESM5_ESM.pdf]
