# Supplementary material for: Oral 8-aminoguanine against age-related retinal degeneration
Source: Commun Biol. 2025 May 26;8:812. doi: 10.1038/s42003-025-08242-1 (PMC12106806; doi:10.1038/s42003-025-08242-1)
Supplement: Supplementary file 6 — Supplementary Data 4 [file 42003_2025_8242_MOESM6_ESM.pdf]

Immunoblots for Fig2E-F and K-L

Immunoblot of intact and cleaved caspase-3 for Figure 2E-F

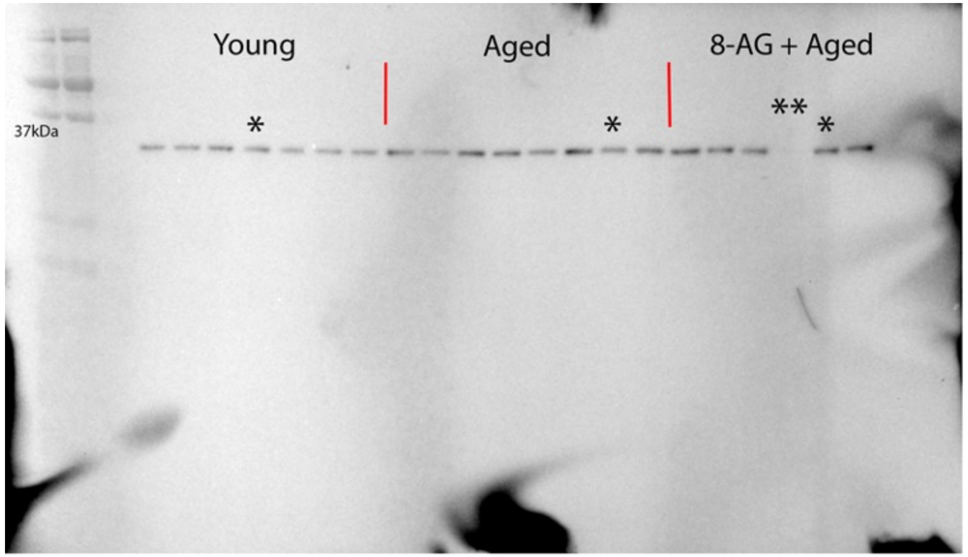

Uncleaved caspase 3; 35kDa
Cell Signaling 9662, Rabbit 1:5000

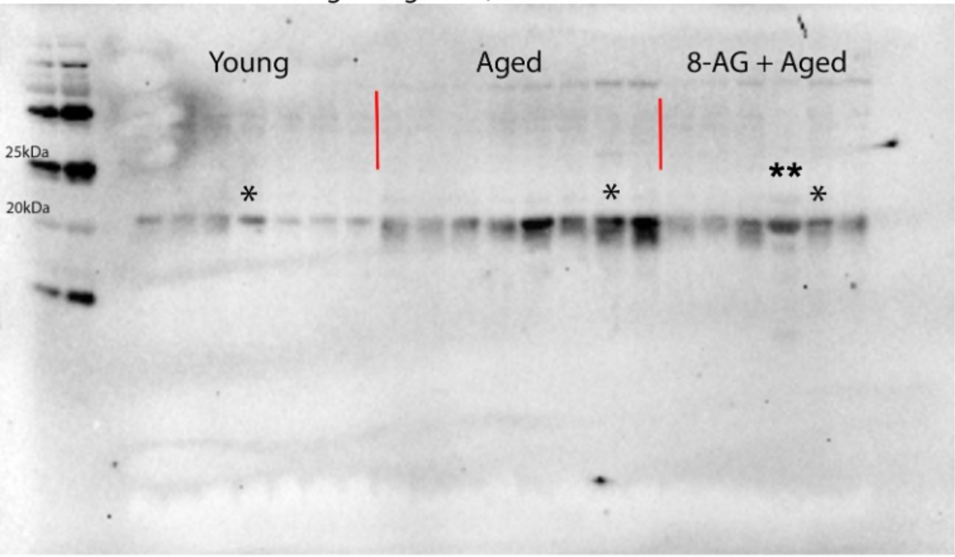

Cleaved caspase 3; 17,19kDa
Cell Signaling 9664 Rabbit 1:1000
[illegible]

Ponsau total protein stain

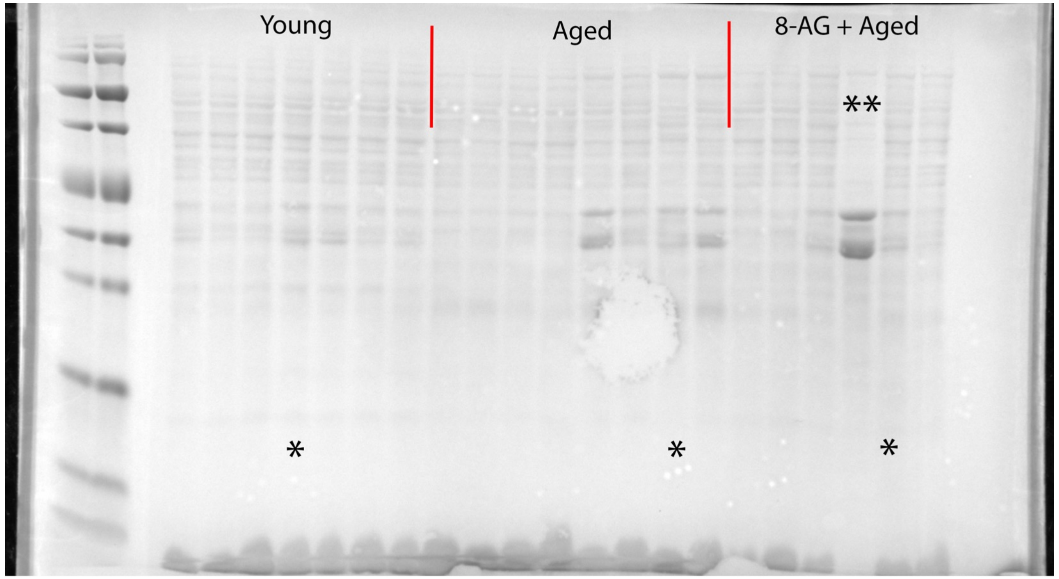

Protein stain for caspase 3
* = representative bands
** = dropped

Immunoblot of rhodopsin for Figure 2K-L

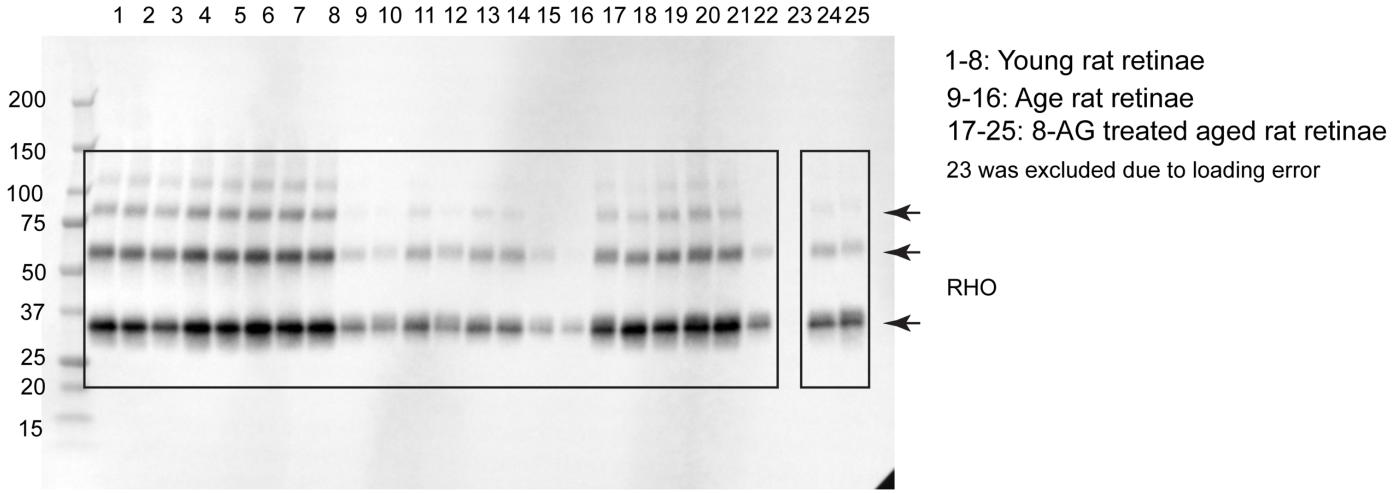

1-8: Young rat retinae

9-16: Age rat retinae

17-25: 8-AG treated aged rat retinae

23 was excluded due to loading error

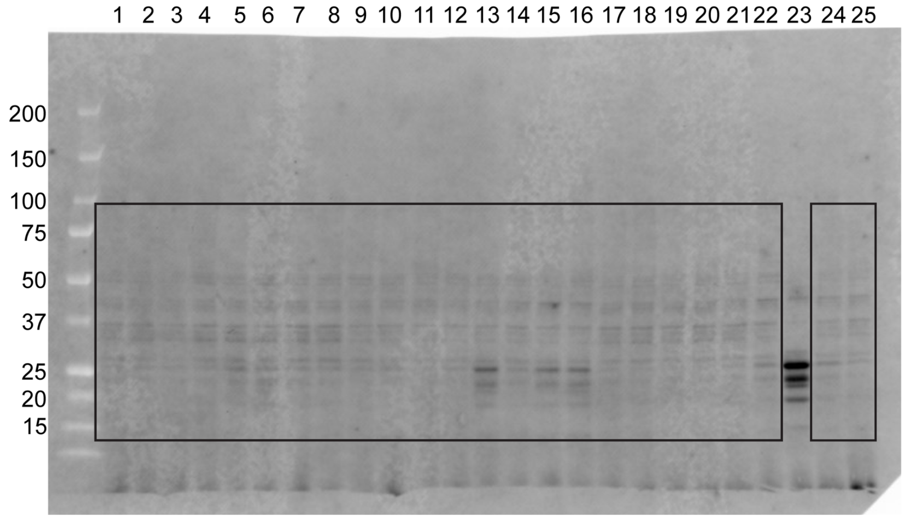

Ponsau S loading control
