# Supplementary material for: Oral 8-aminoguanine against age-related retinal degeneration
Source: Commun Biol. 2025 May 26;8:812. doi: 10.1038/s42003-025-08242-1 (PMC12106806; doi:10.1038/s42003-025-08242-1)

Rhodopsin Immunofluorescence for Figure 2G-J

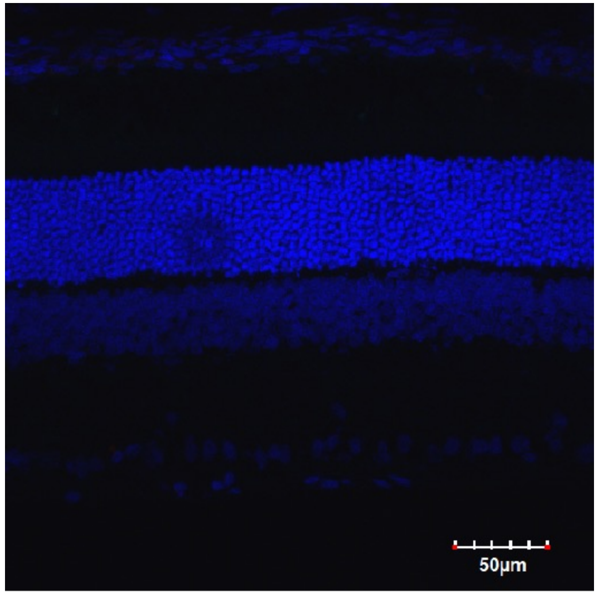

Secondary only control

Young rat retinae (biological repeat 1-2)

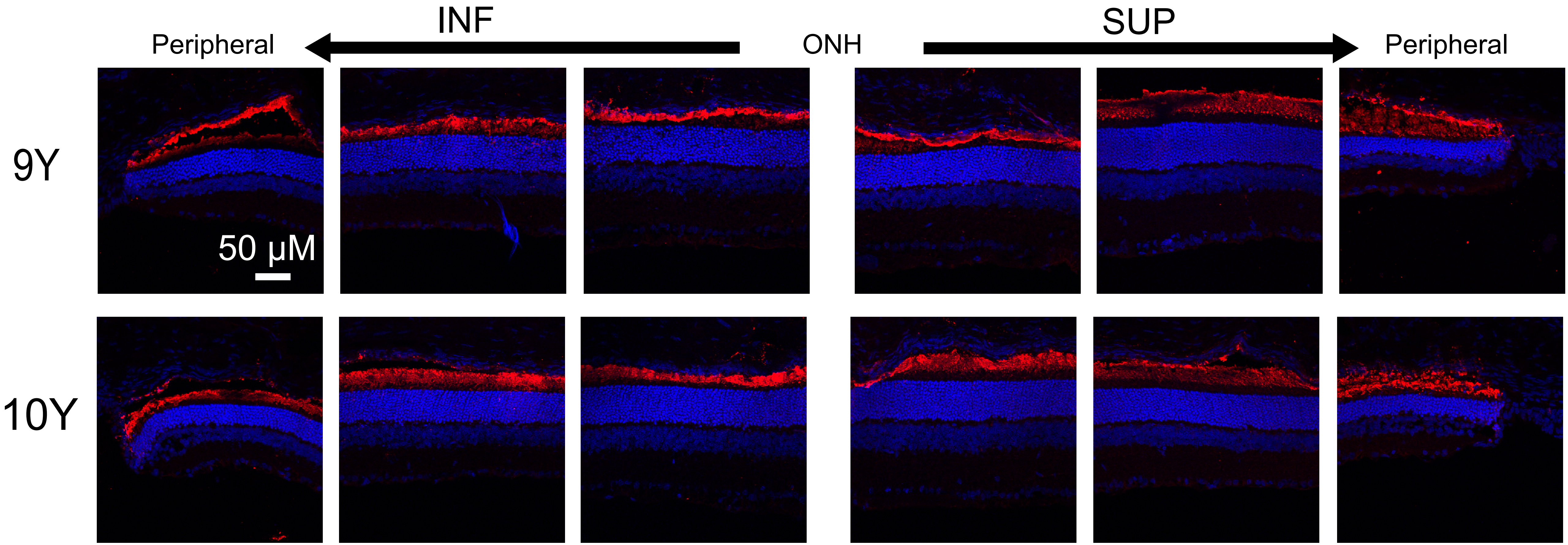

Young rat retinae (biological repeat 3-4)

11Y

12Y

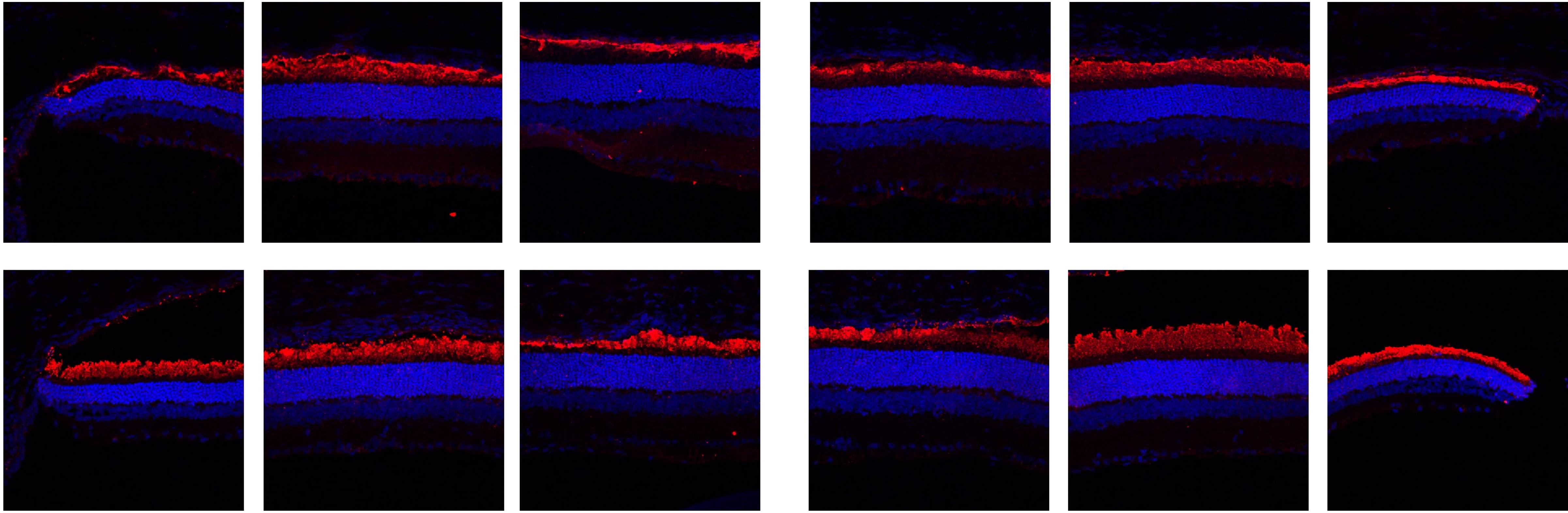

Young rat retinae (biological repeat 5-6)

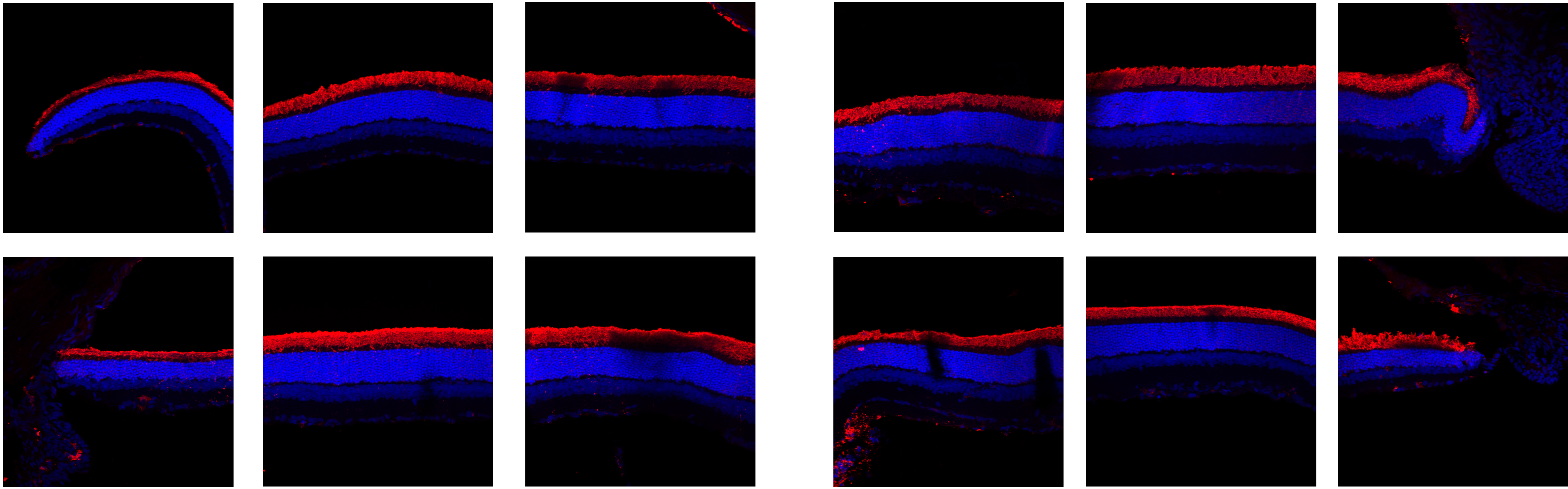

Young rat retinae (biological repeat 5-6)

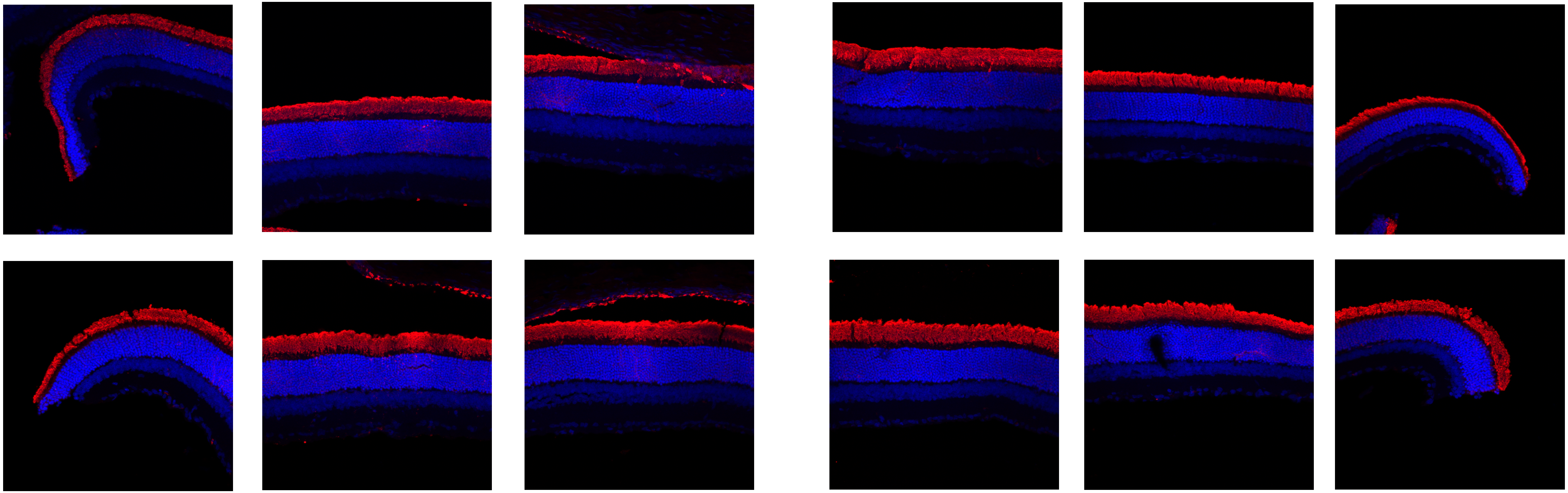

Aged water-treated F344 rat retinae (Biological repeat 1-2)

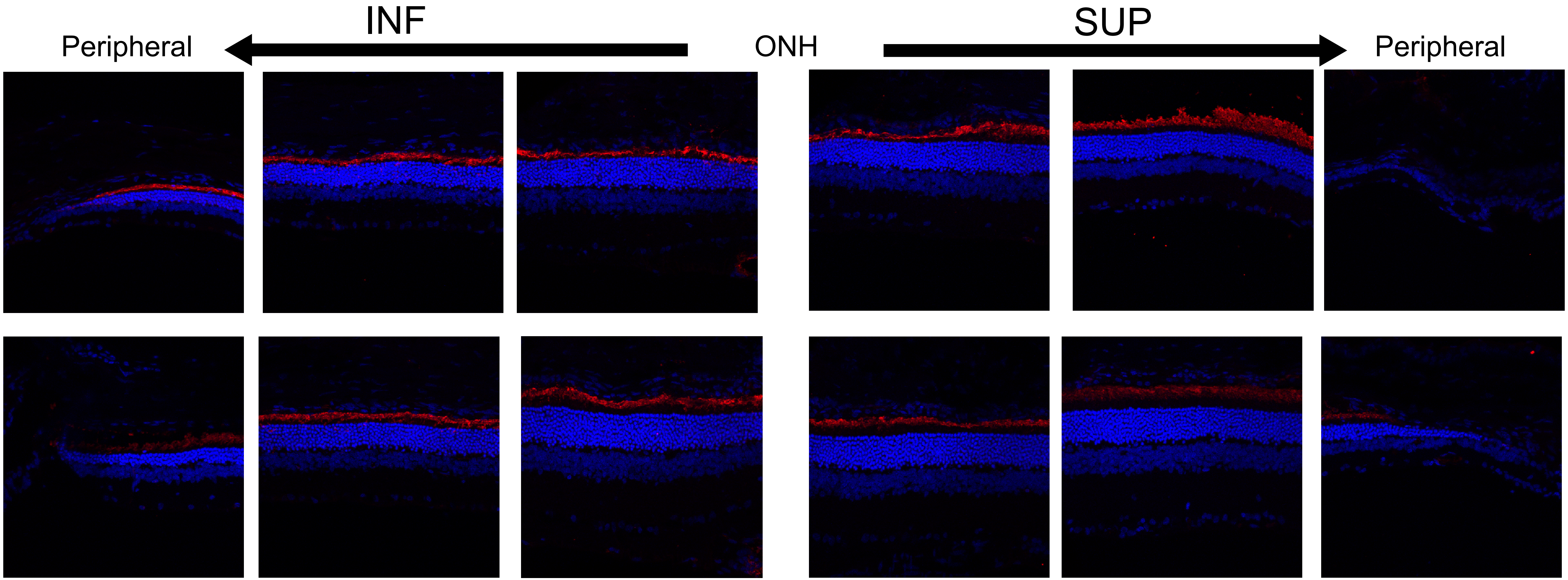

Aged water-treated F344 rat retinae (biological repeat 3-4)

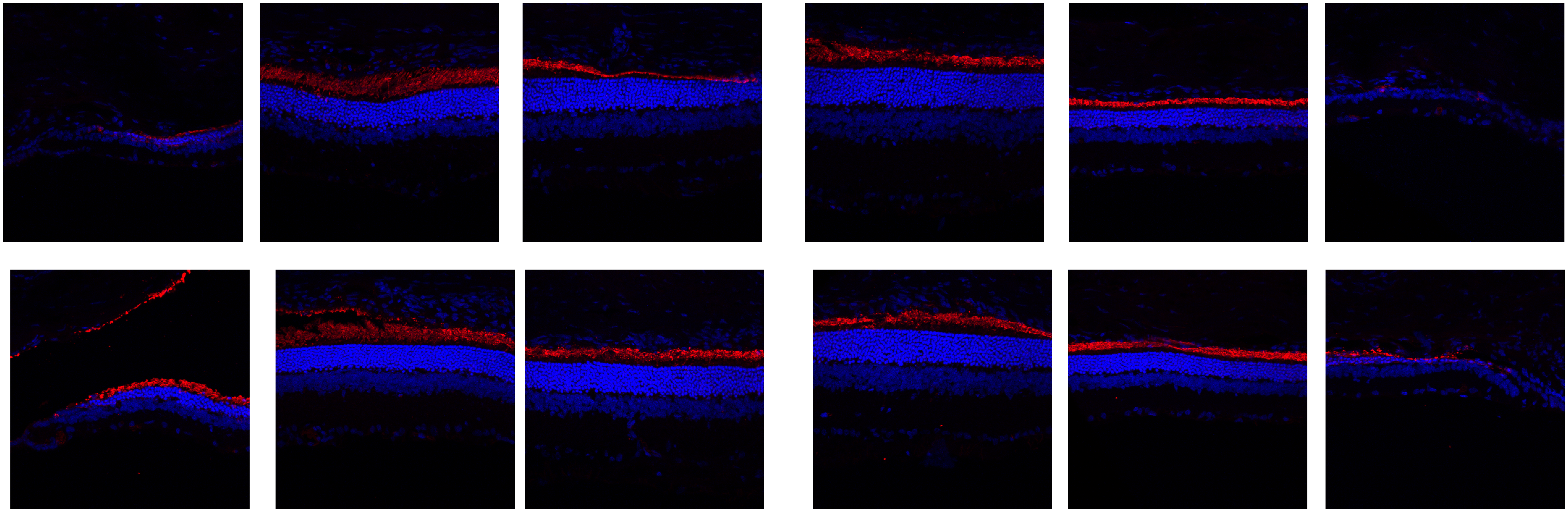

Aged water-treated F344 rat retinae (biological repeat 5-6)

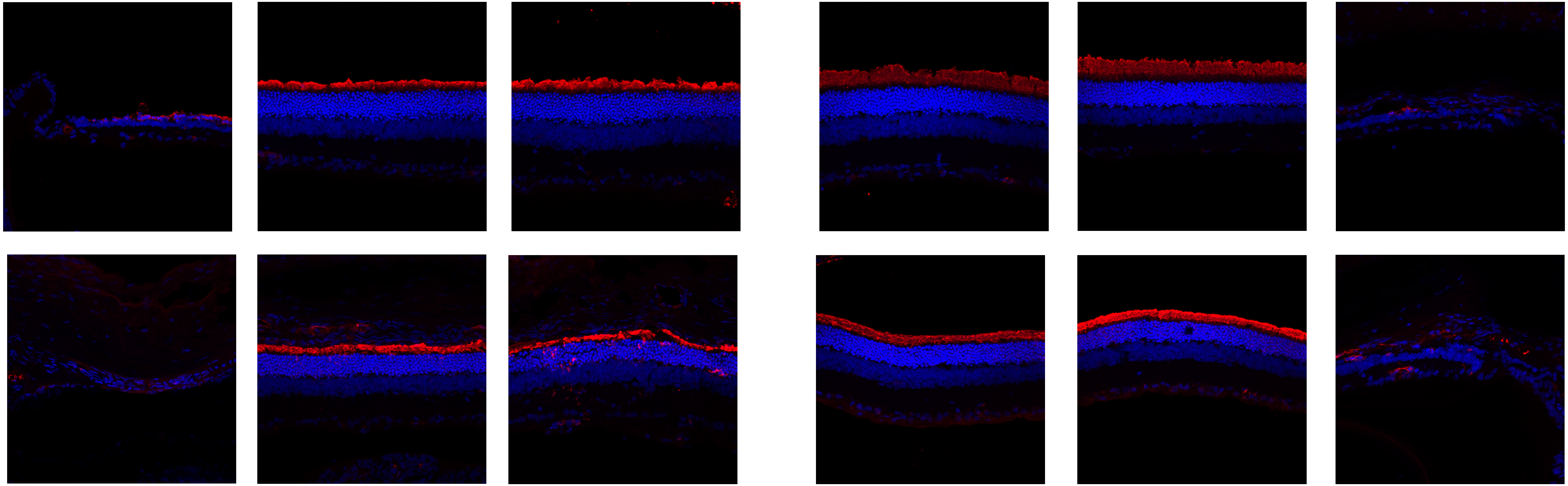

Aged water-treated F344 rat retinae (biological repeat 7-8)

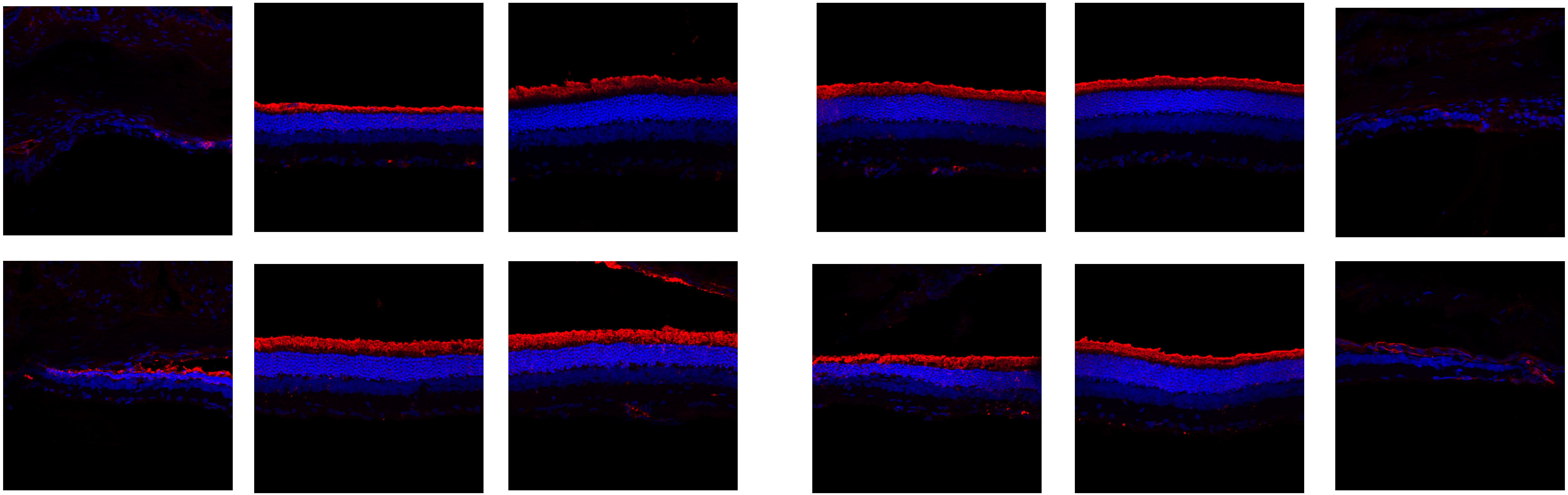

Aged 8AG-treated F344 rat retinae (biol. Repeat 1-3)

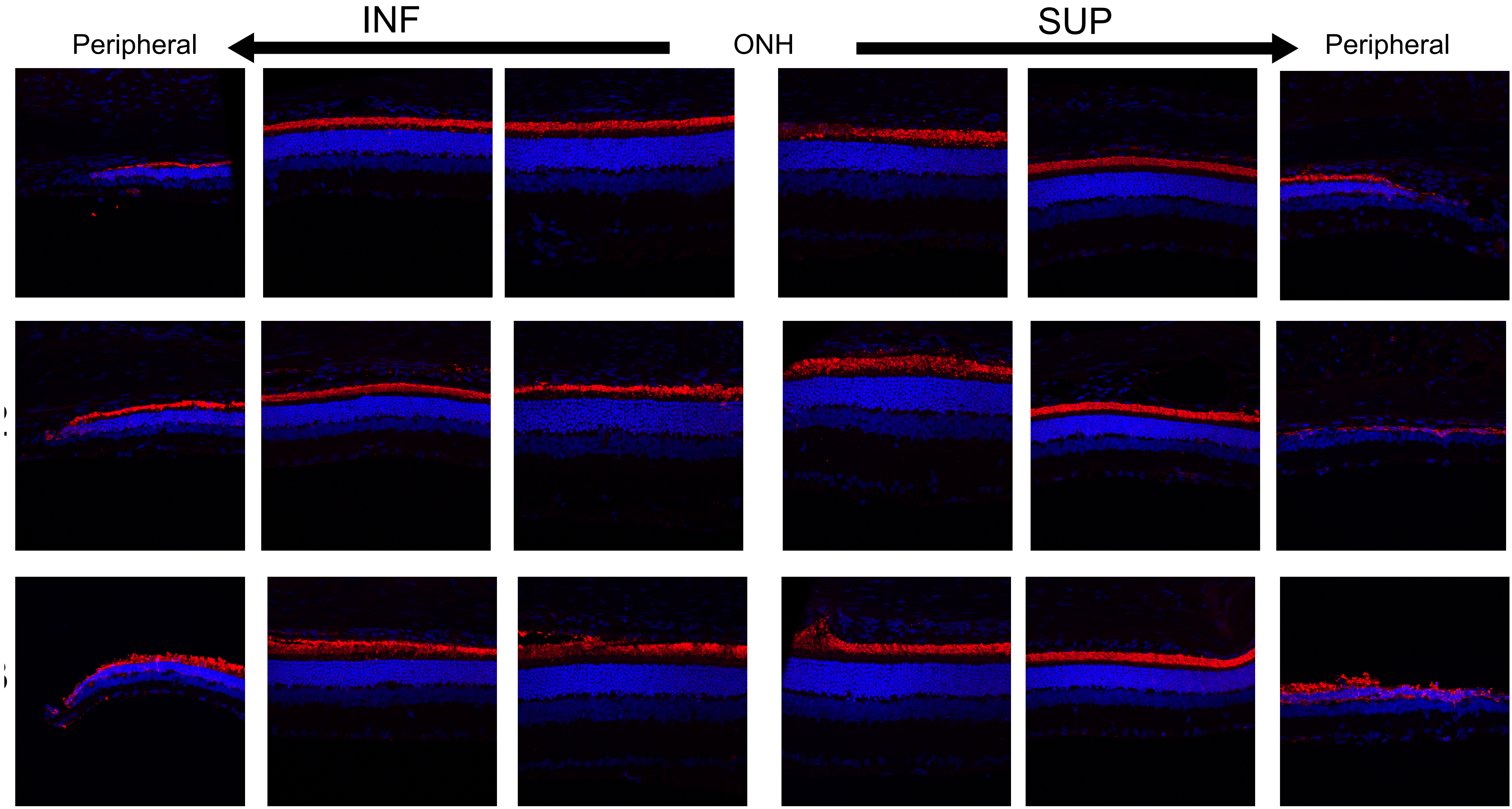

Aged 8AG-treated F344 rat retinae (biol. Repeat 4-5)

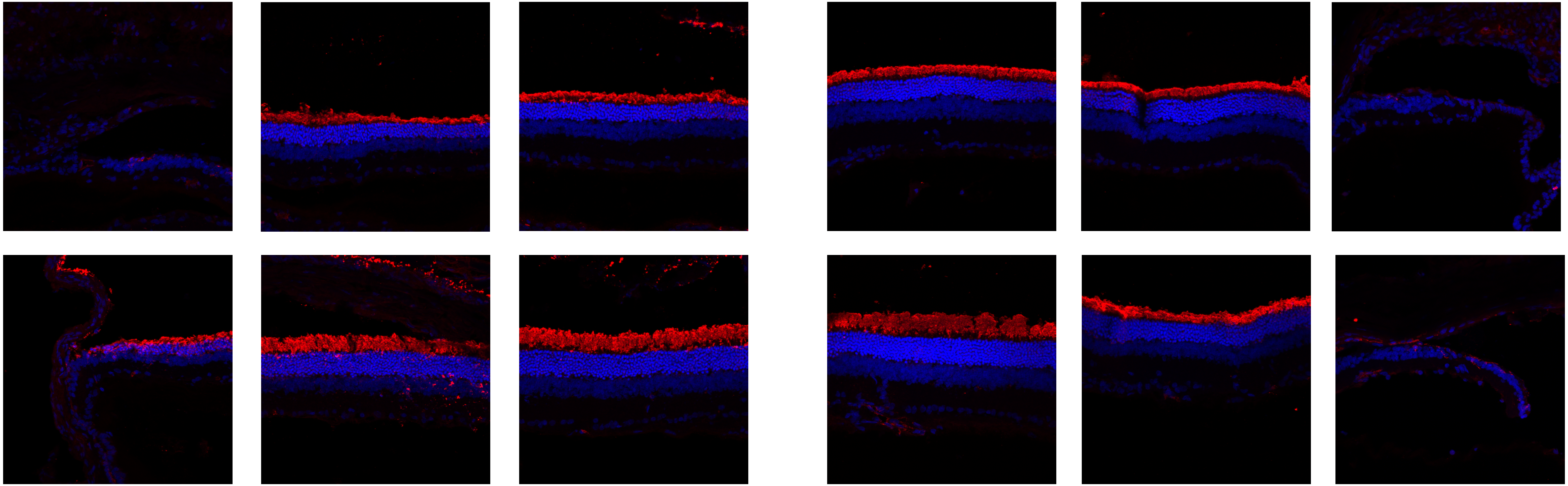

Aged 8AG-treated F344 rat retinae (biol. Repeat 6-7)

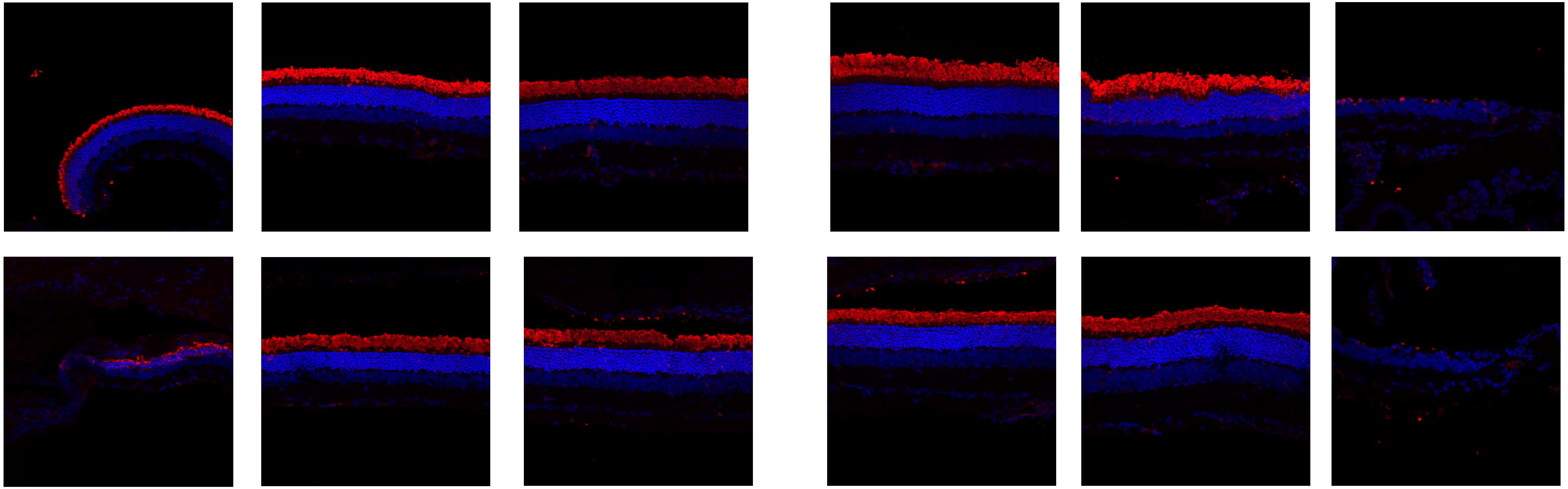

Supplement: Supplementary file 7 — Supplementary Data 5 [file 42003_2025_8242_MOESM7_ESM.pdf]
