# Supplementary material for: Oral 8-aminoguanine against age-related retinal degeneration
Source: Commun Biol. 2025 May 26;8:812. doi: 10.1038/s42003-025-08242-1 (PMC12106806; doi:10.1038/s42003-025-08242-1)

IHC of Arrestin1 and PDE6 for Figure S3

Young Rat 1_Arrestin+PDE6_INF_1

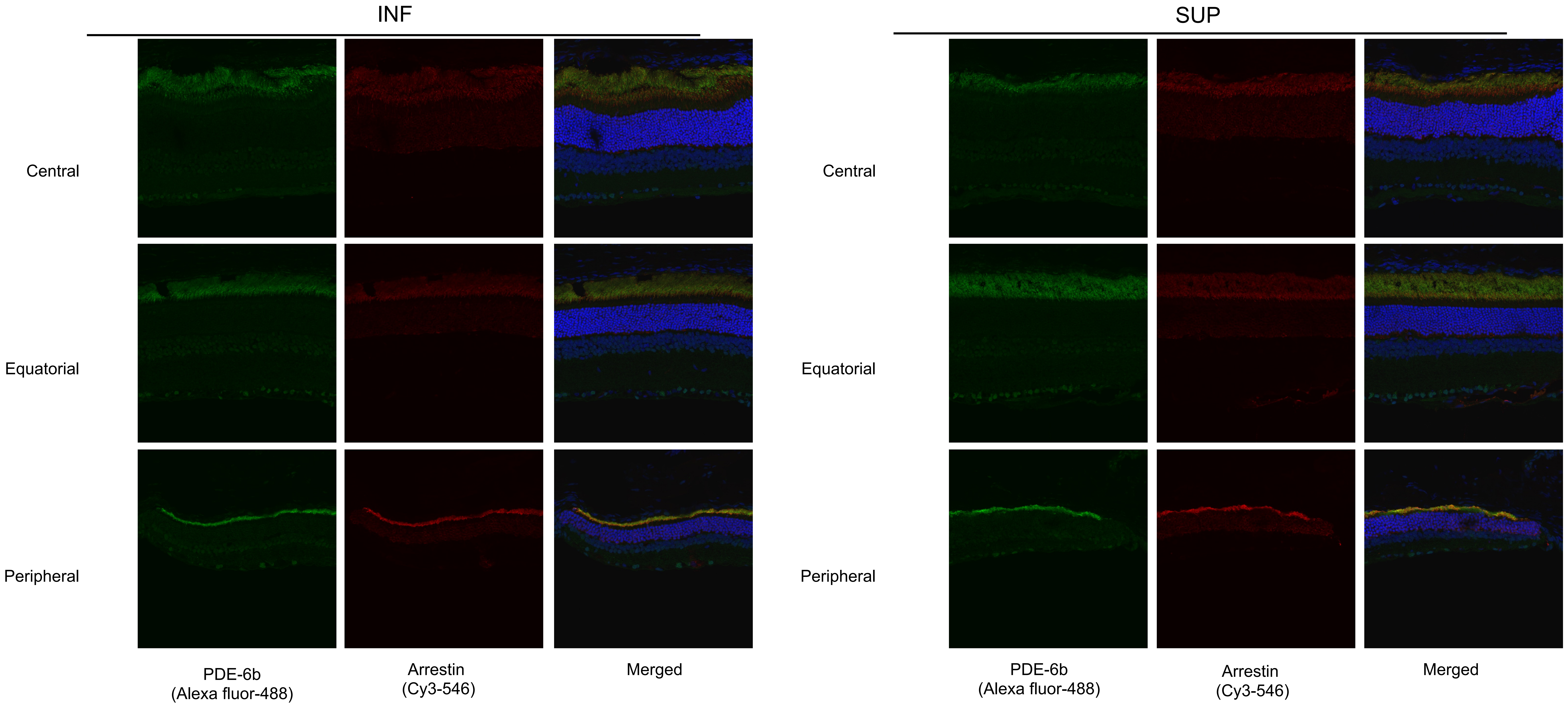

Young Rat 2_Arrestin+PDE6_INF_1

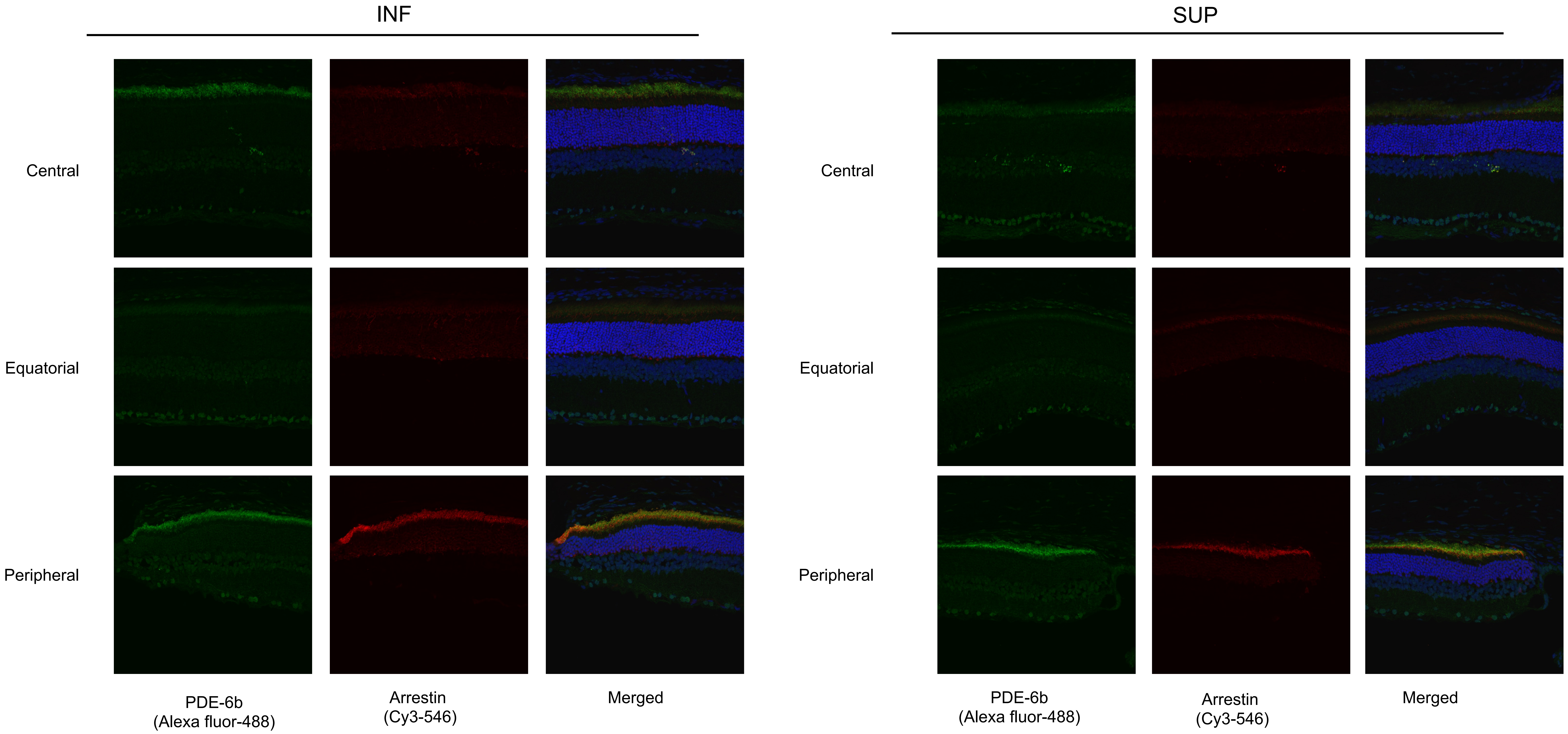

Young Rat 3_Arrestin+PDE6_INF_1

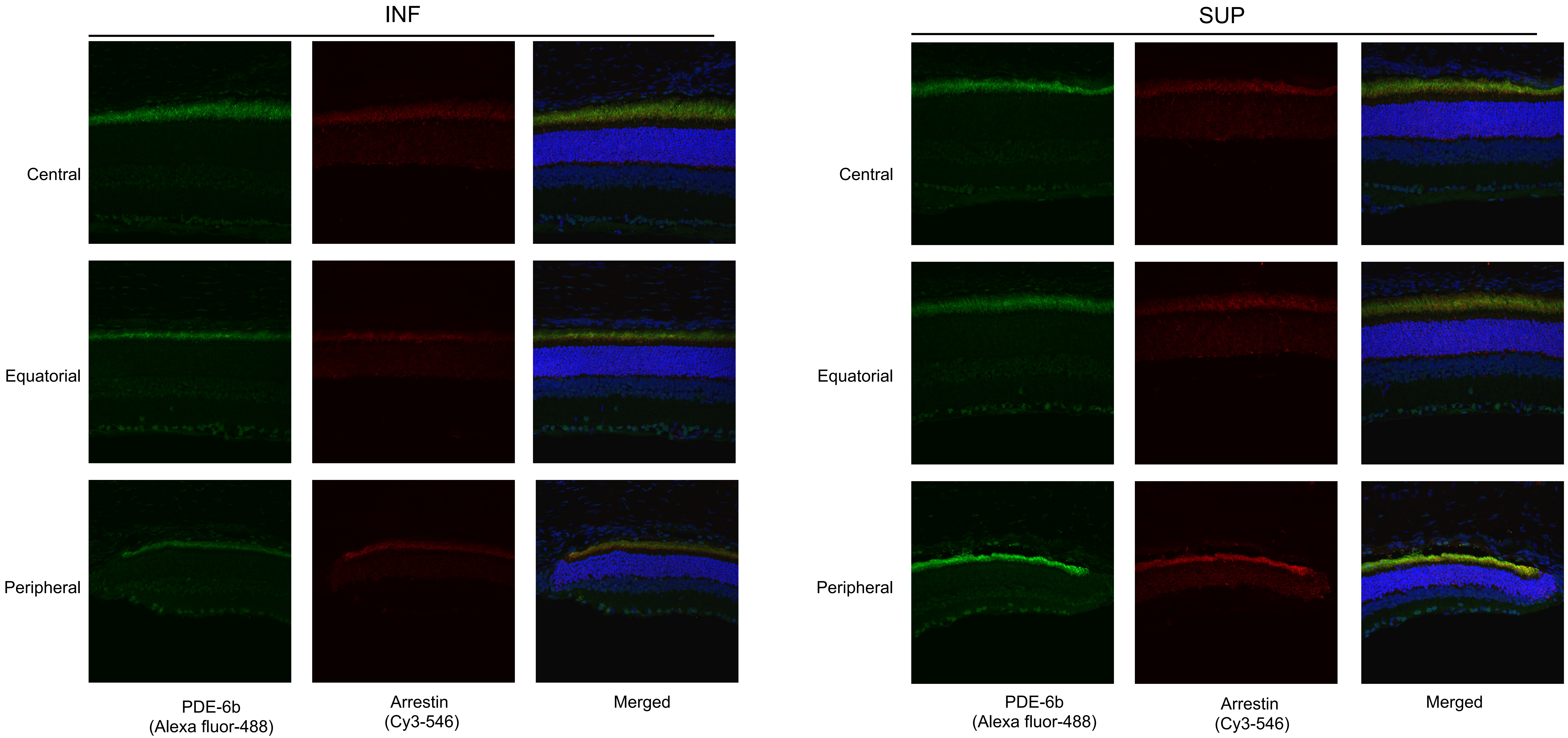

Young Rat 4_Arrestin+PDE6_INF_1

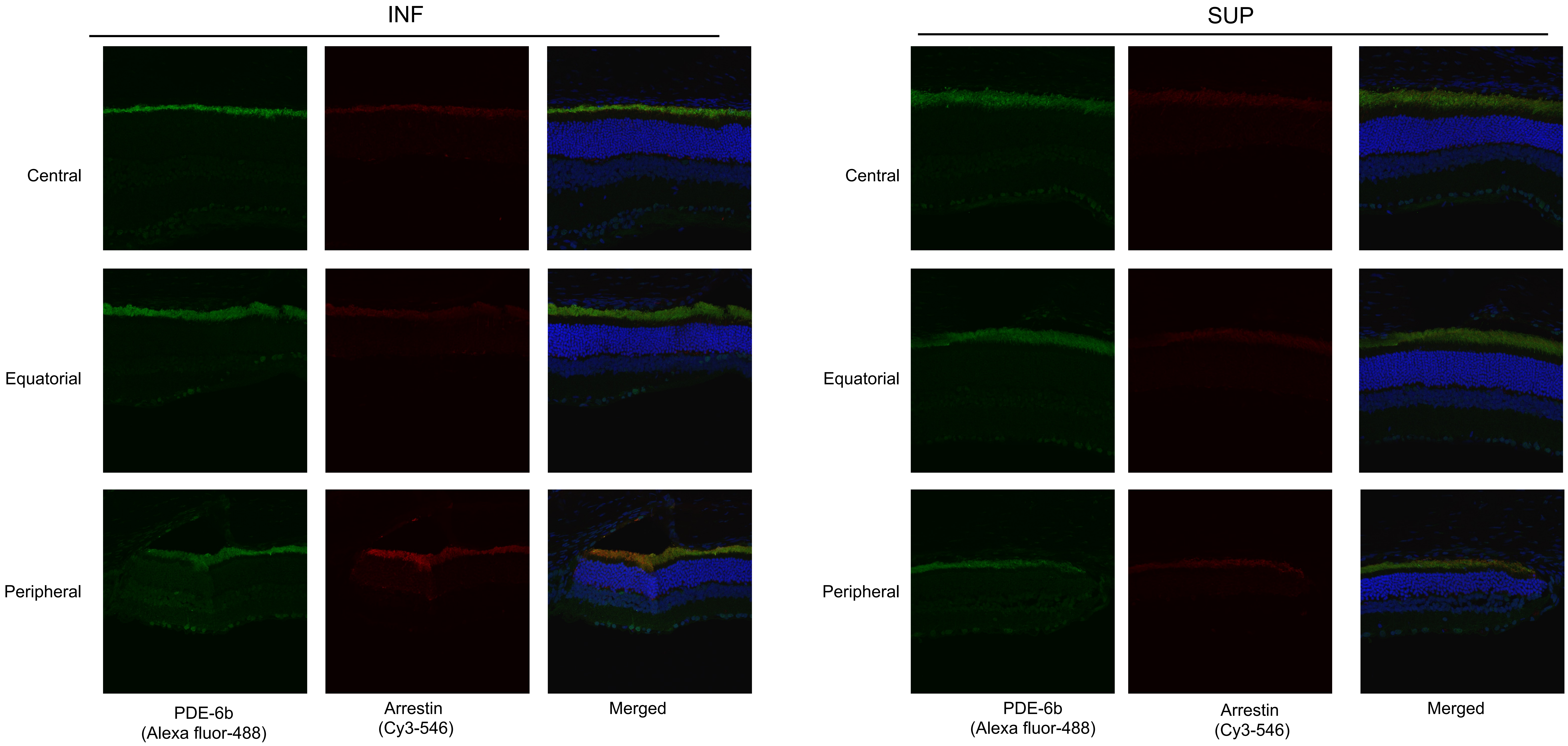

AGED UNTREATED RAT 5_Arrestin+PDE6_INF_A

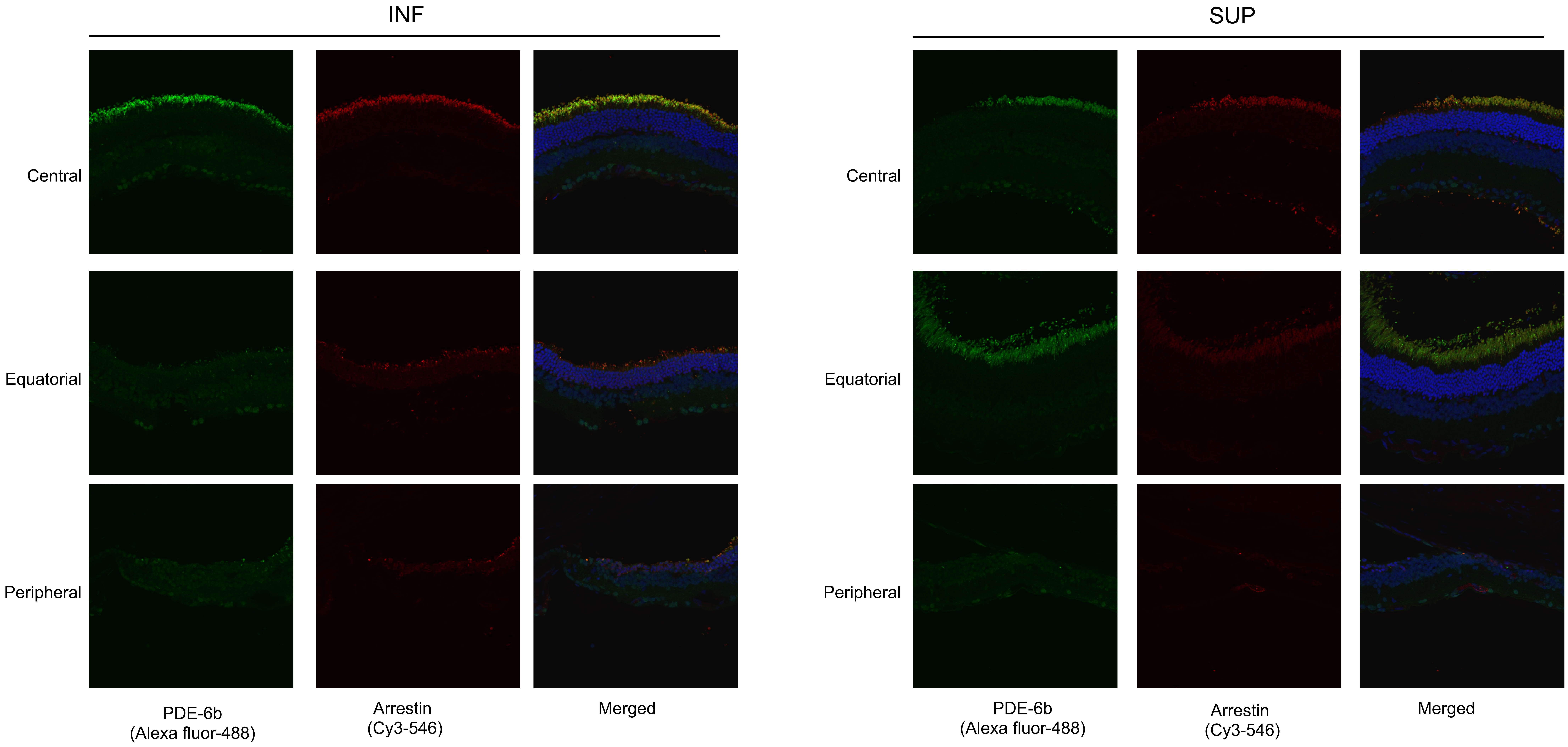

AGED UNTREATED RAT 6_Arrestin+PDE6_INF_A

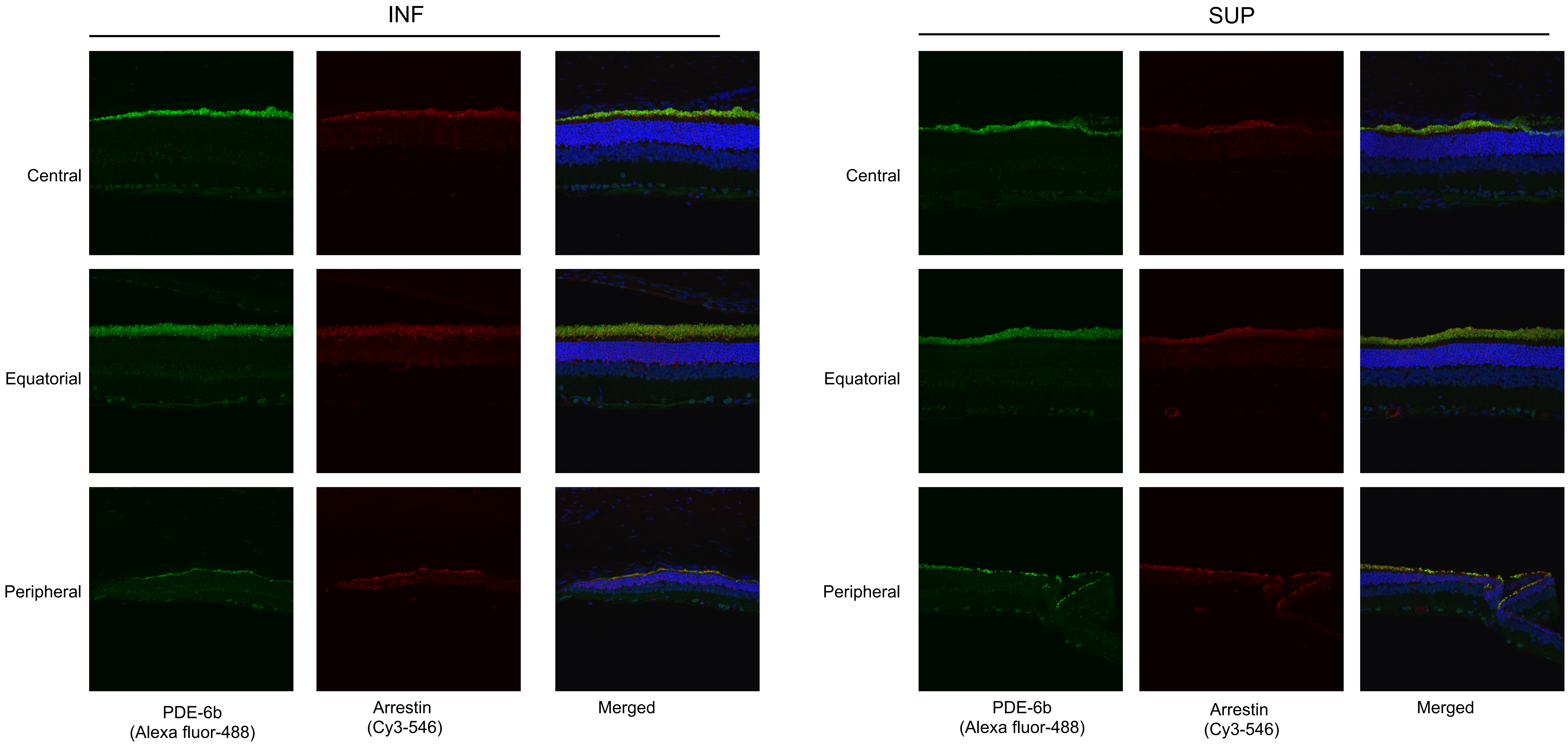

AGED UNTREATED RAT 7_Arrestin+PDE6_INF_A

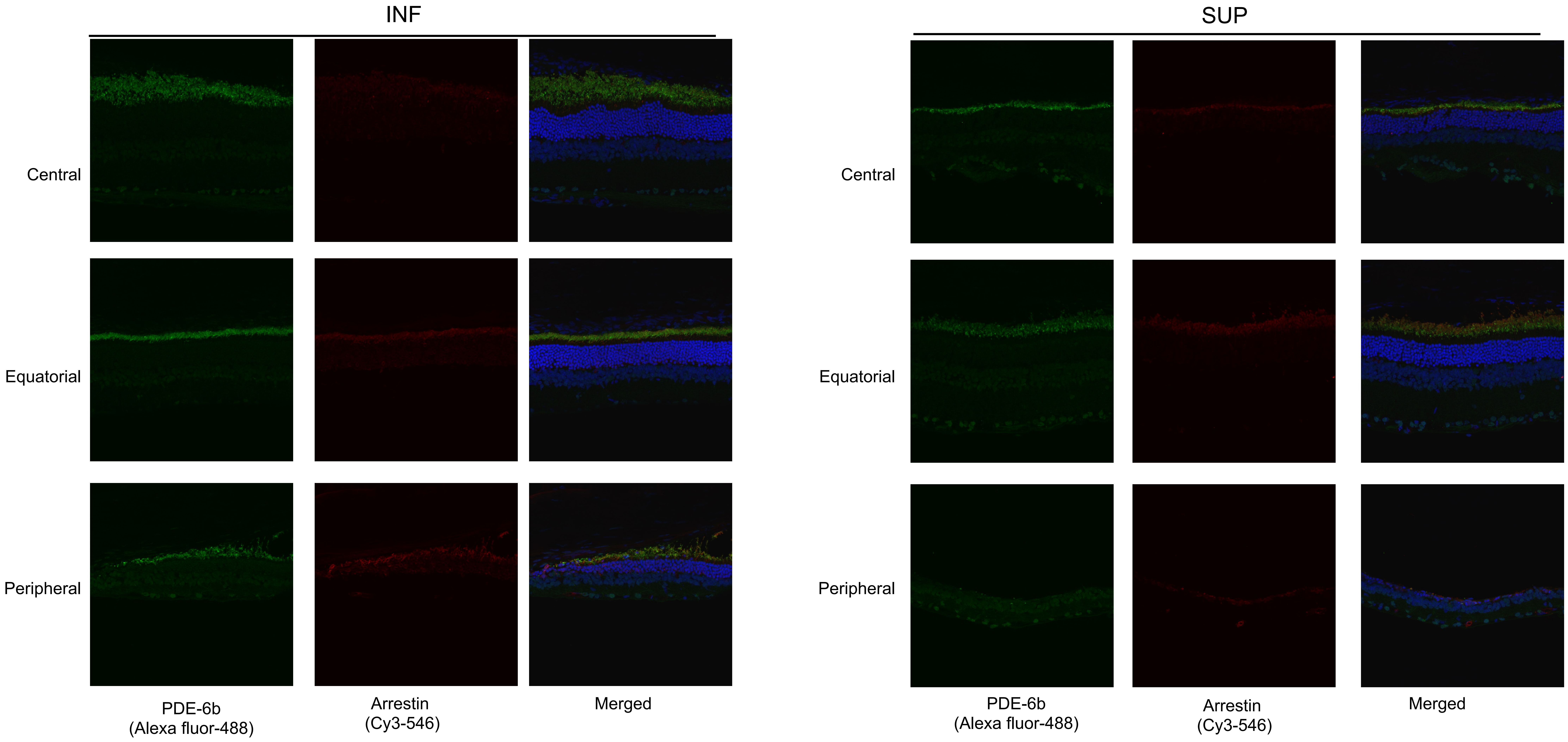

AGED UNTREATED RAT 10_Arrestin+PDE6_INF_A

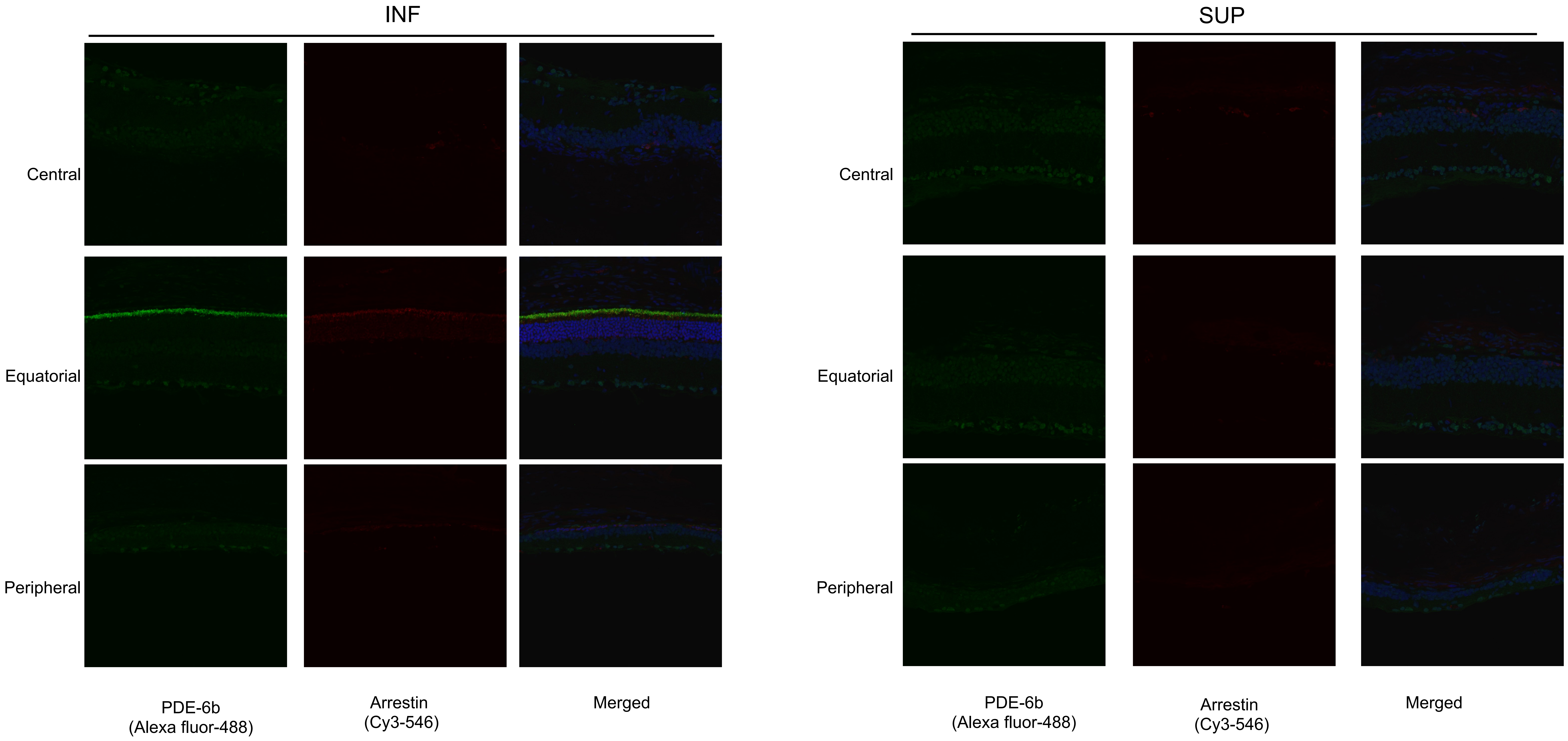

AGED UNTREATED RAT 11_Arrestin+PDE6_INF_A

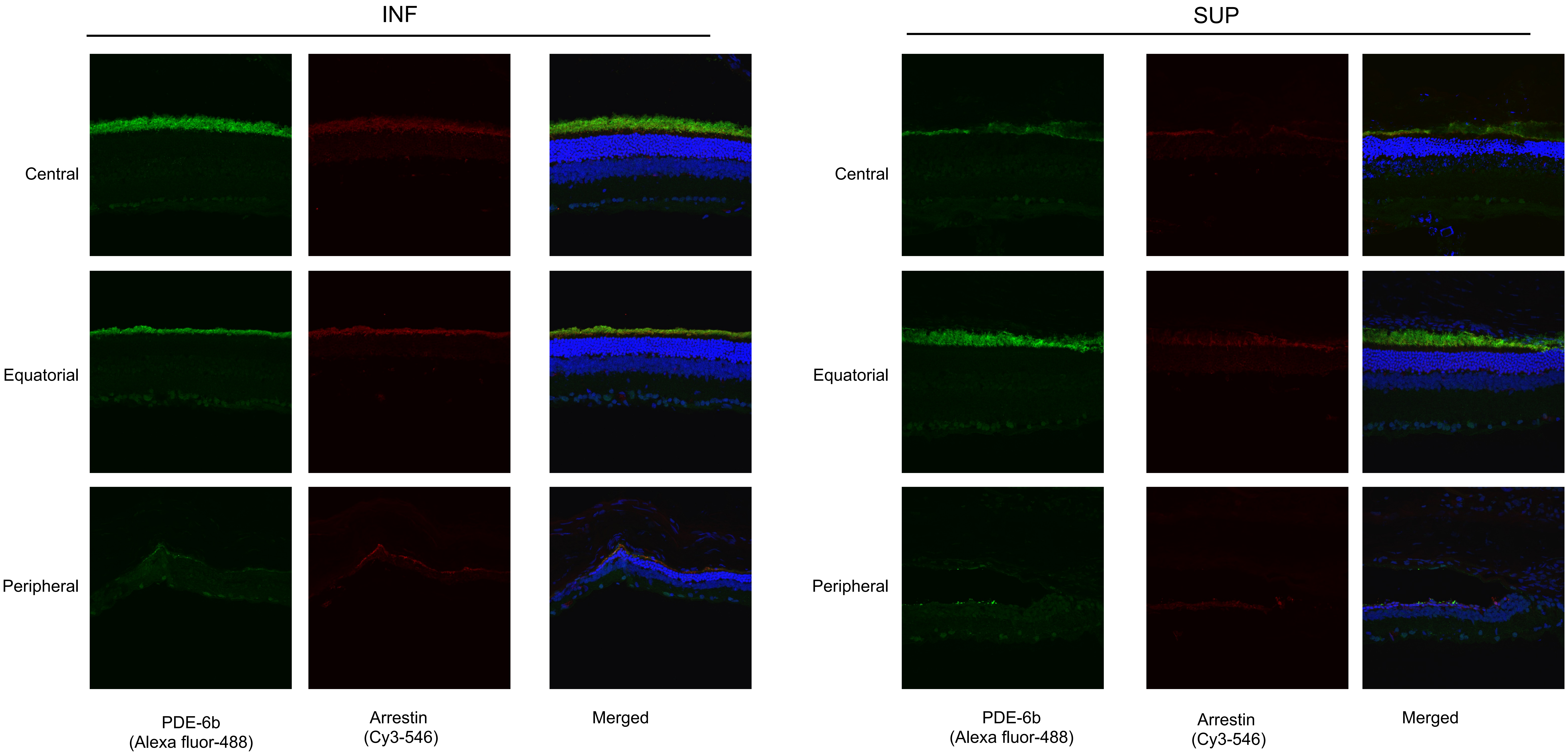

AGED 8-AG TREATED RAT 8_Arrestin+PDE6_INF_1

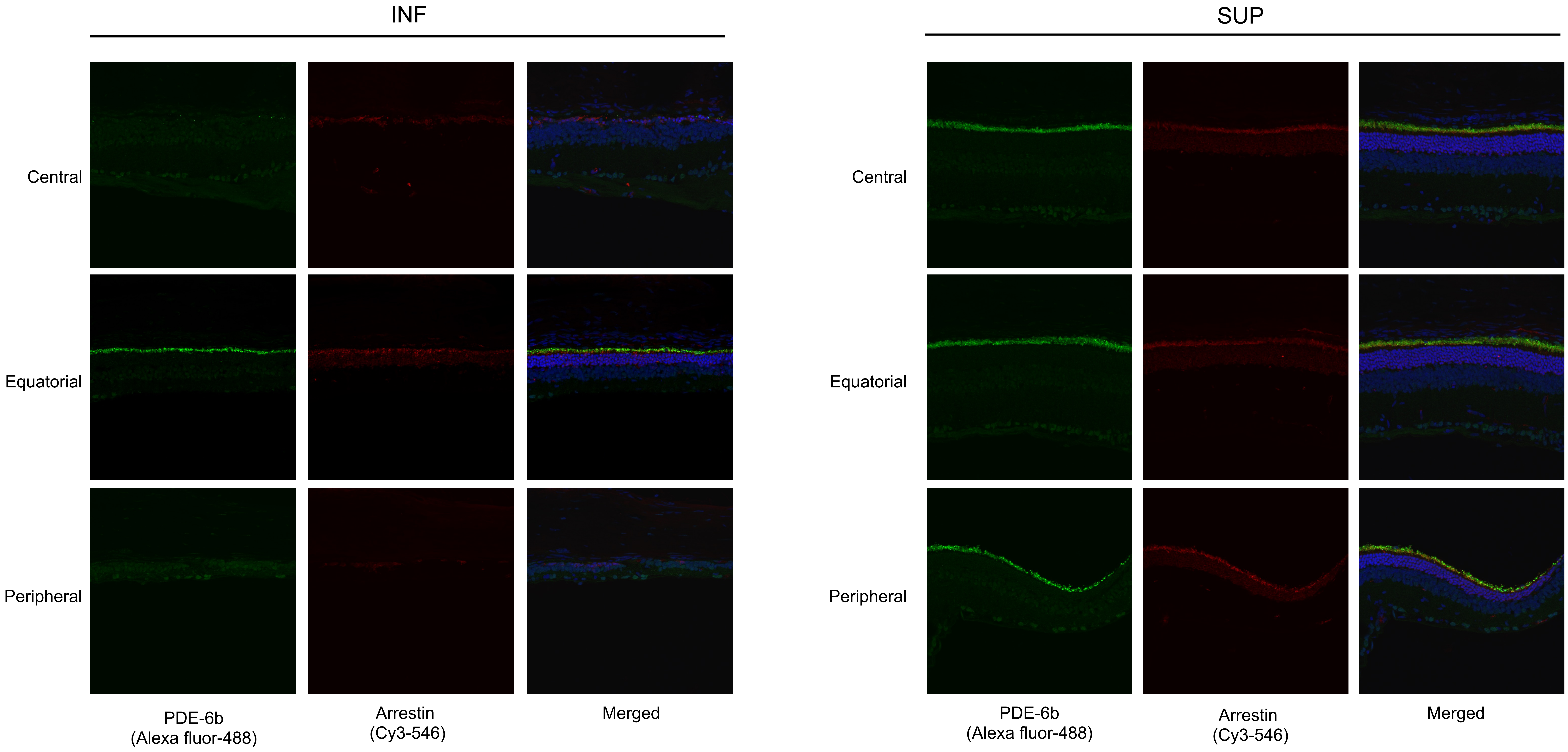

AGED 8-AG TREATED RAT 9_Arrestin+PDE6_INF_1

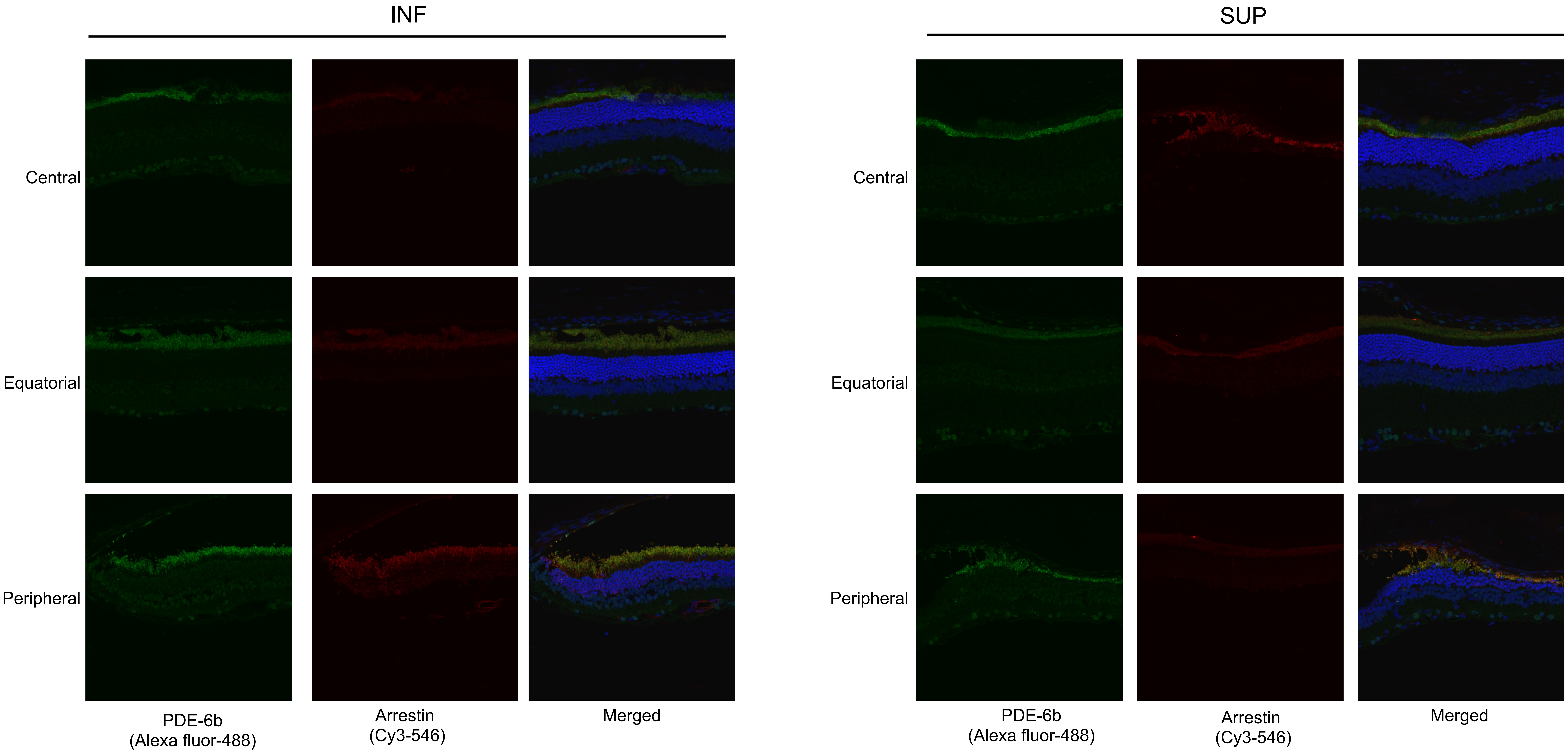

AGED 8-AG TREATED RAT 12_Arrestin+PDE6_INF_1

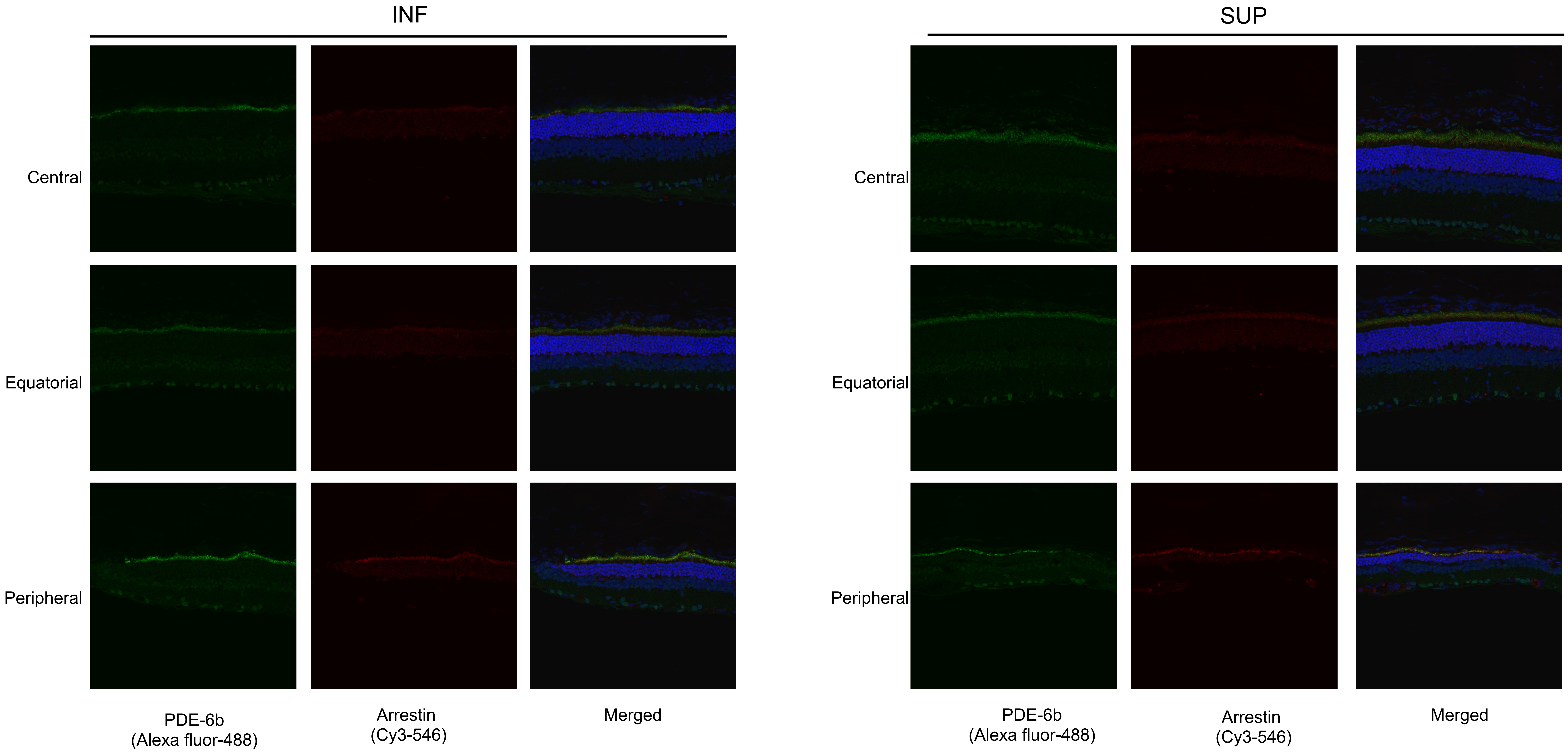

AGED 8-AG TREATED RAT 13_Arrestin+PDE6_INF_1

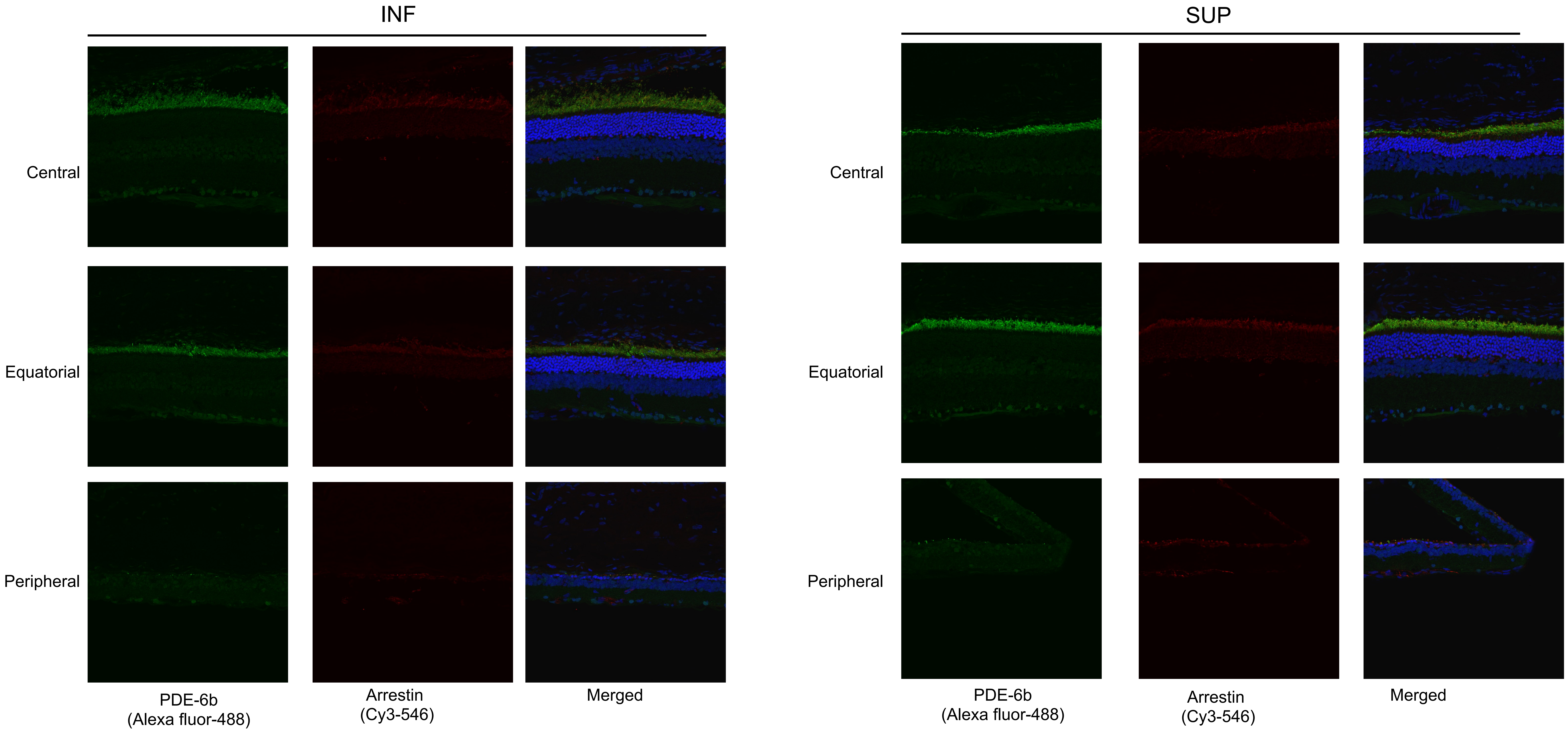

Supplement: Supplementary file 8 — Supplementary Data 6 [file 42003_2025_8242_MOESM8_ESM.pdf]
