# Supplementary material for: Oral 8-aminoguanine against age-related retinal degeneration
Source: Commun Biol. 2025 May 26;8:812. doi: 10.1038/s42003-025-08242-1 (PMC12106806; doi:10.1038/s42003-025-08242-1)

PNA staining

For Fig. 2M-P

Young Rat 1

INF

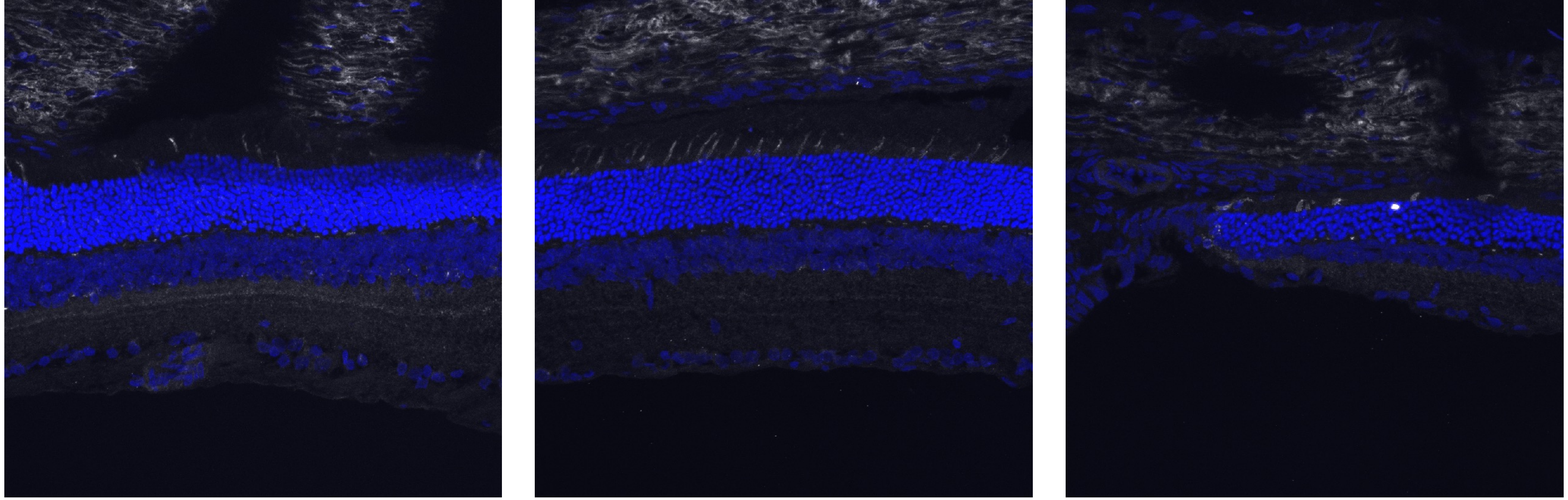

Central | Equatorial | Peripheral

SUP

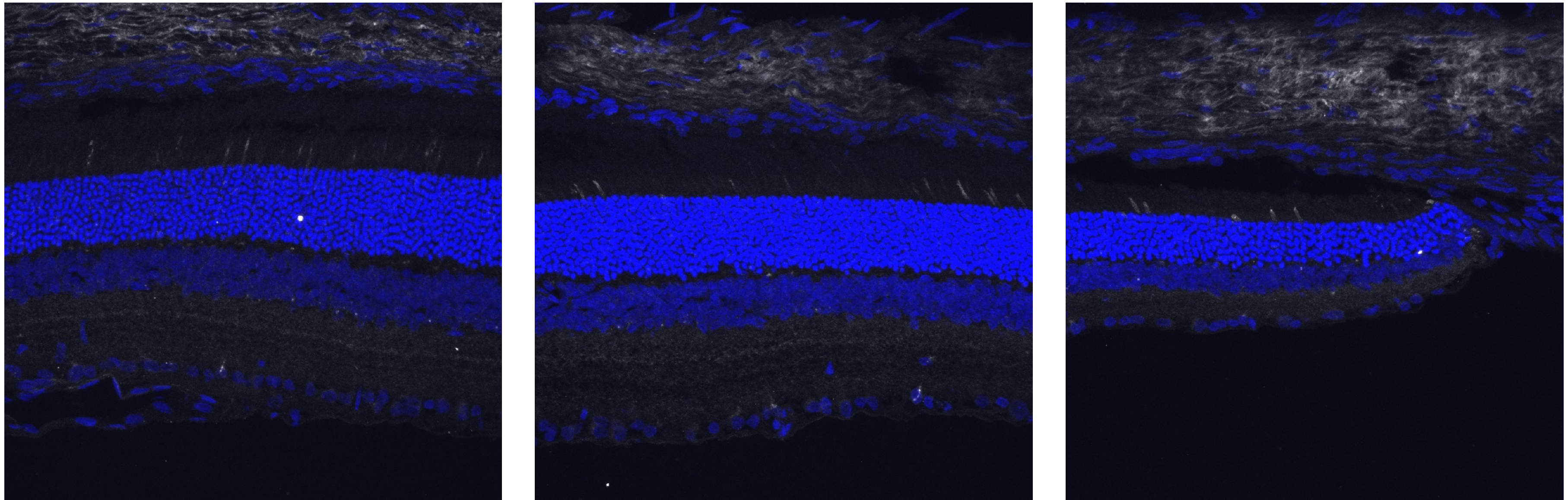

Young Rat 2

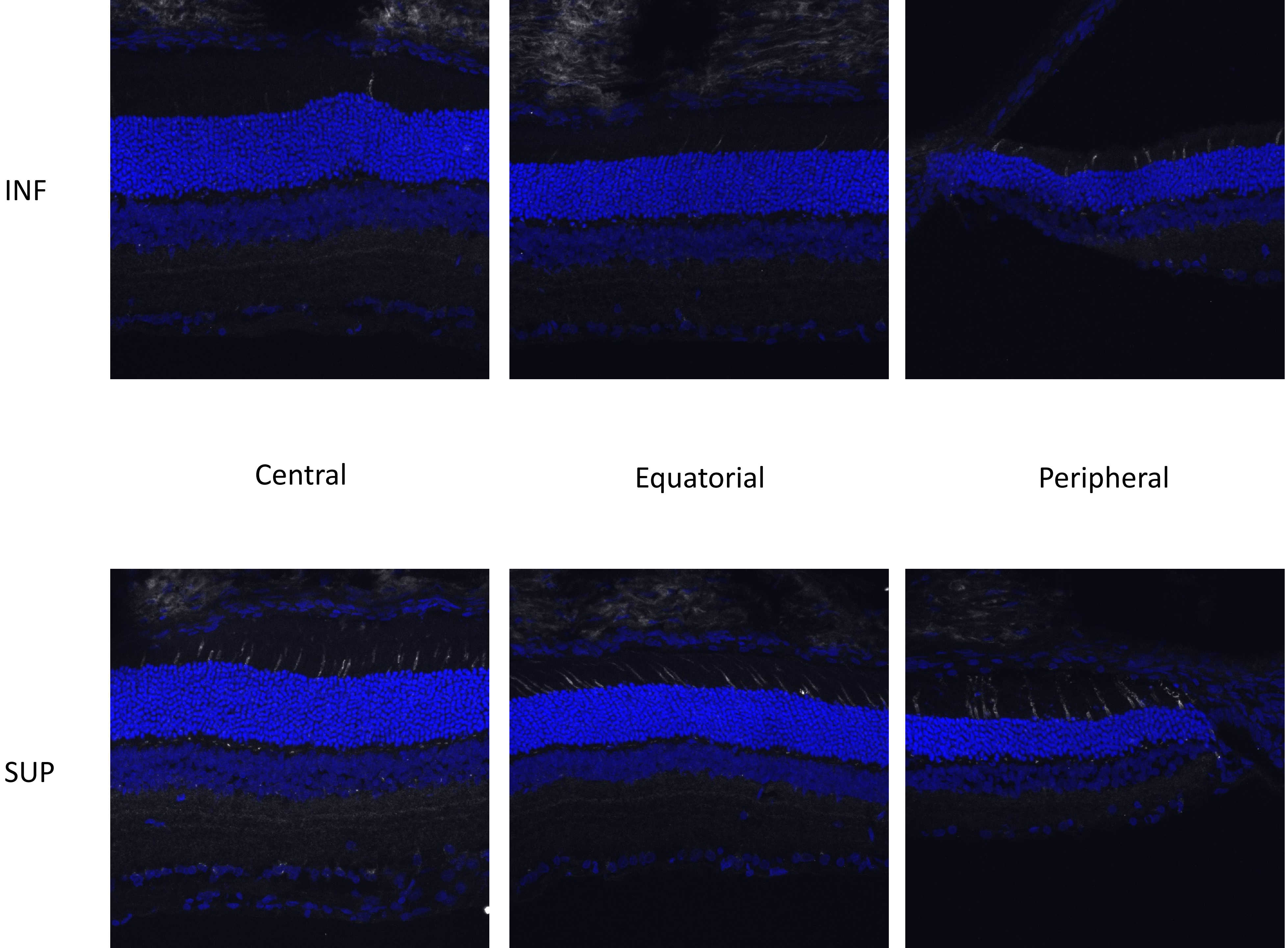

Young Rat 3

INF

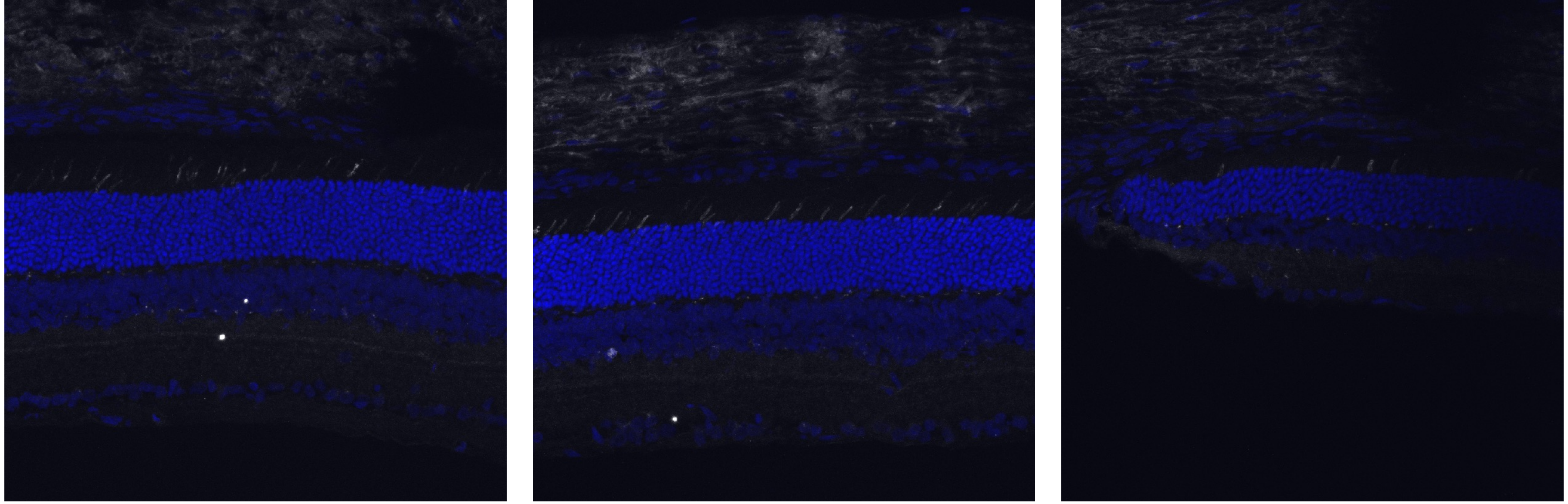

Central   Equatorial   Peripheral

SUP

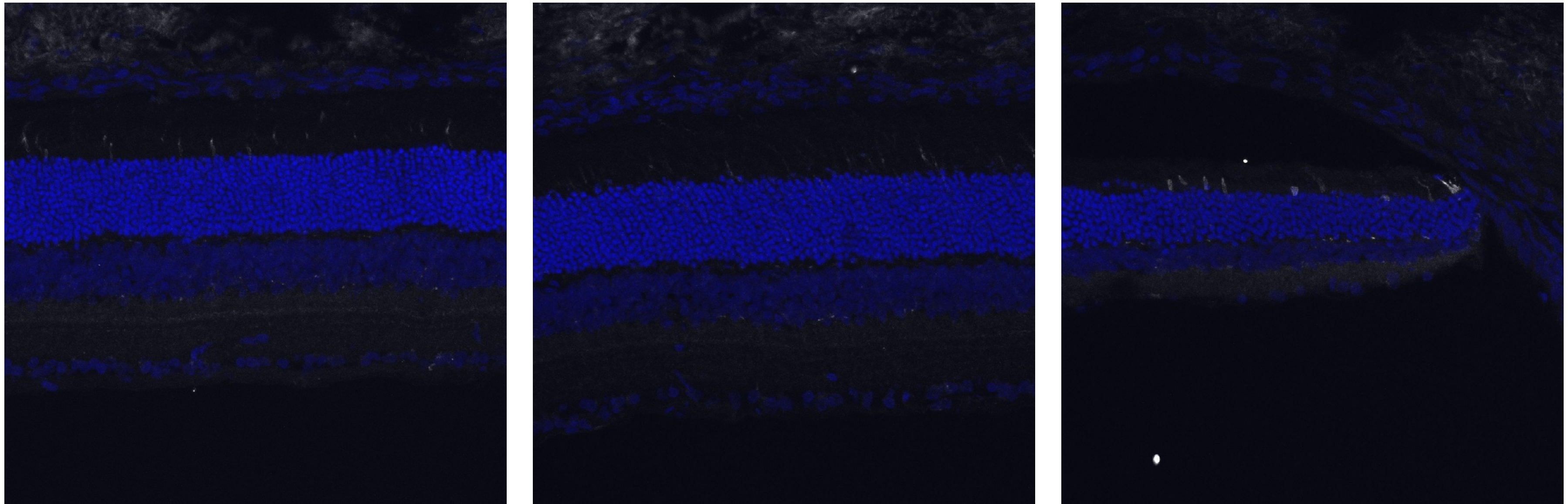

Young Rat 4

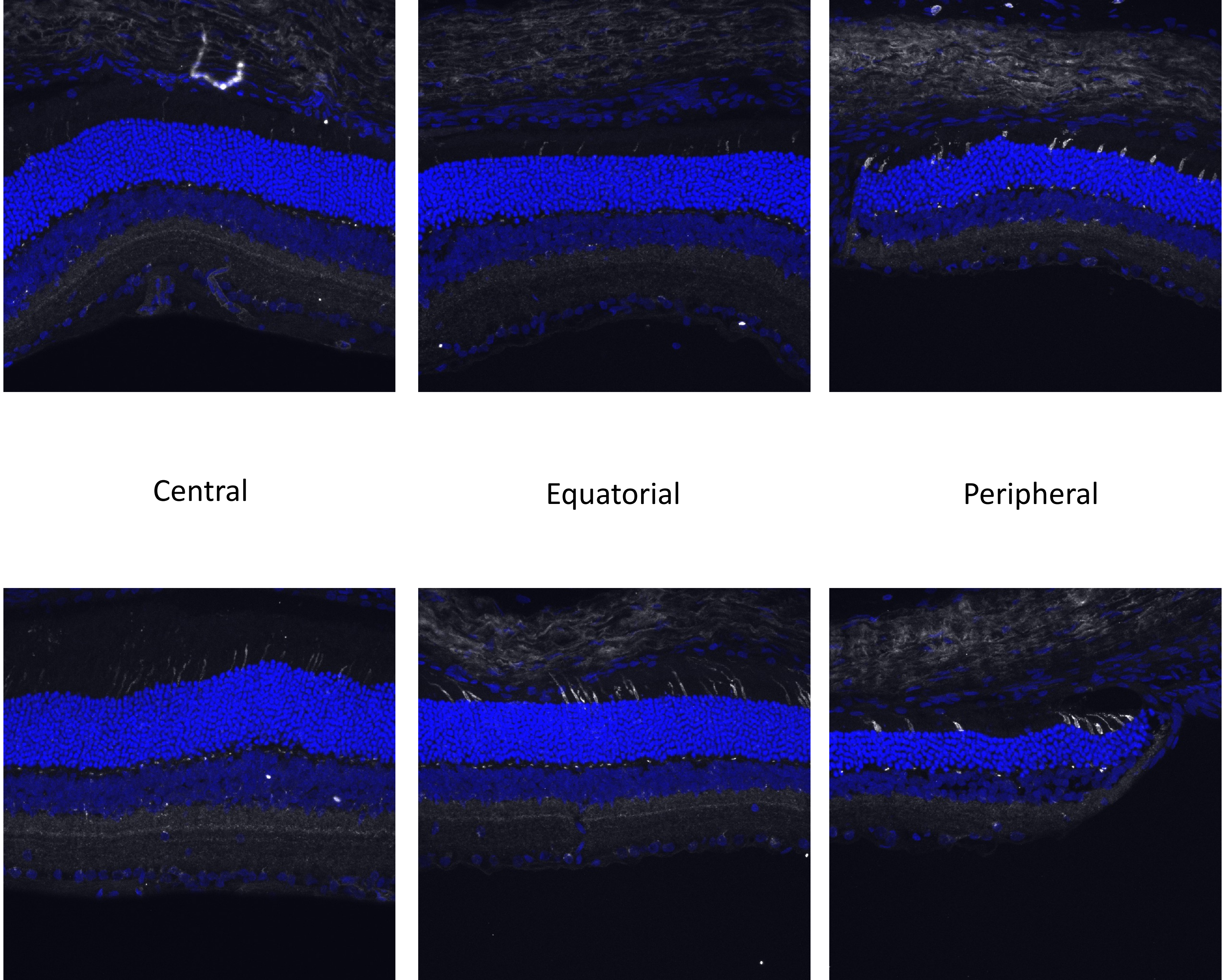

Young5

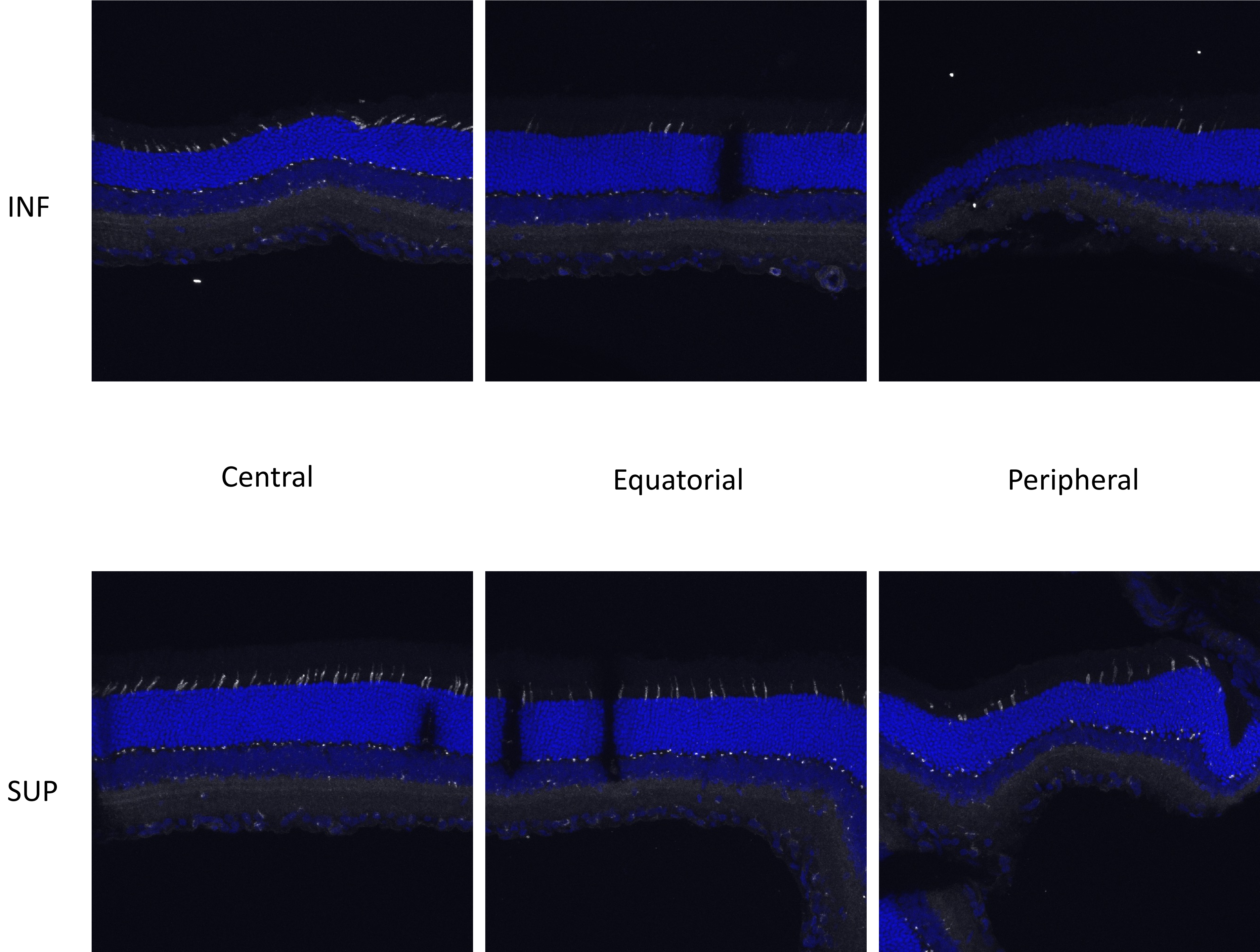

Young6

INF

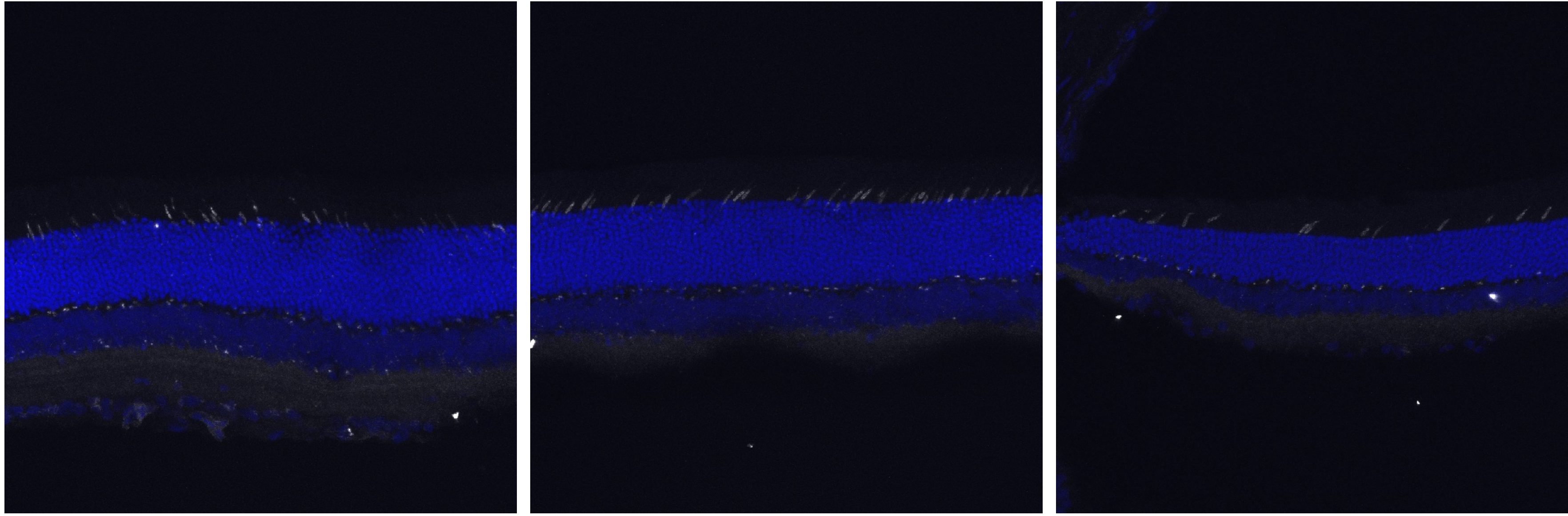

Central Equatorial Peripheral

SUP

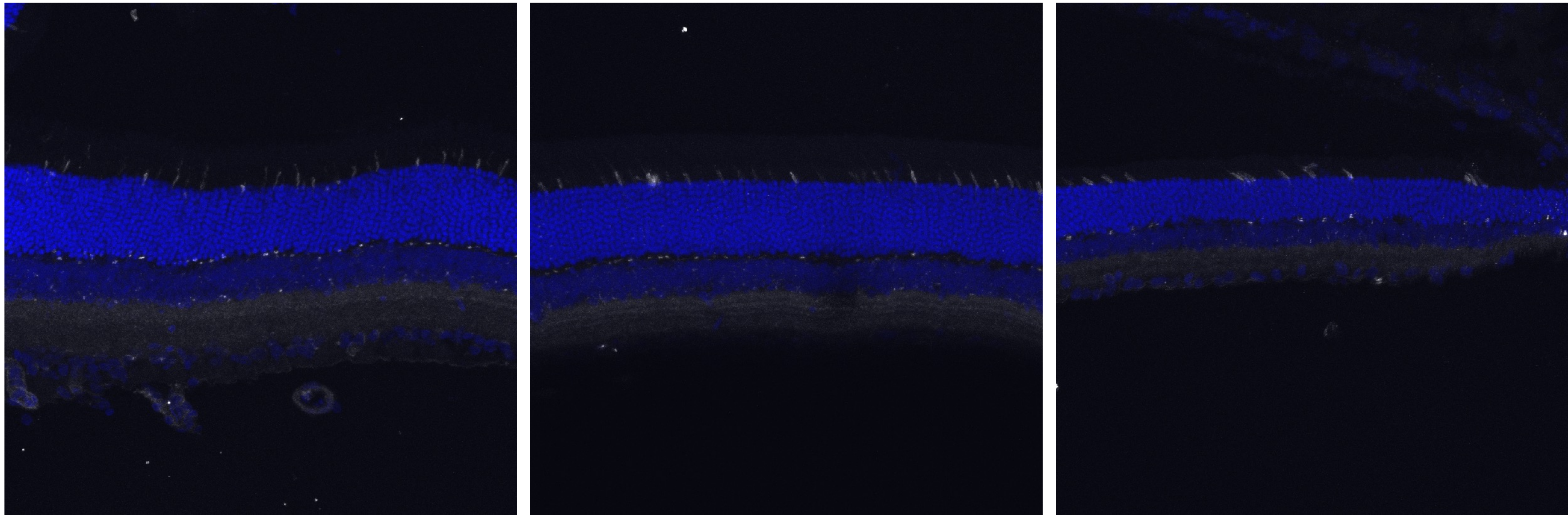

Young7

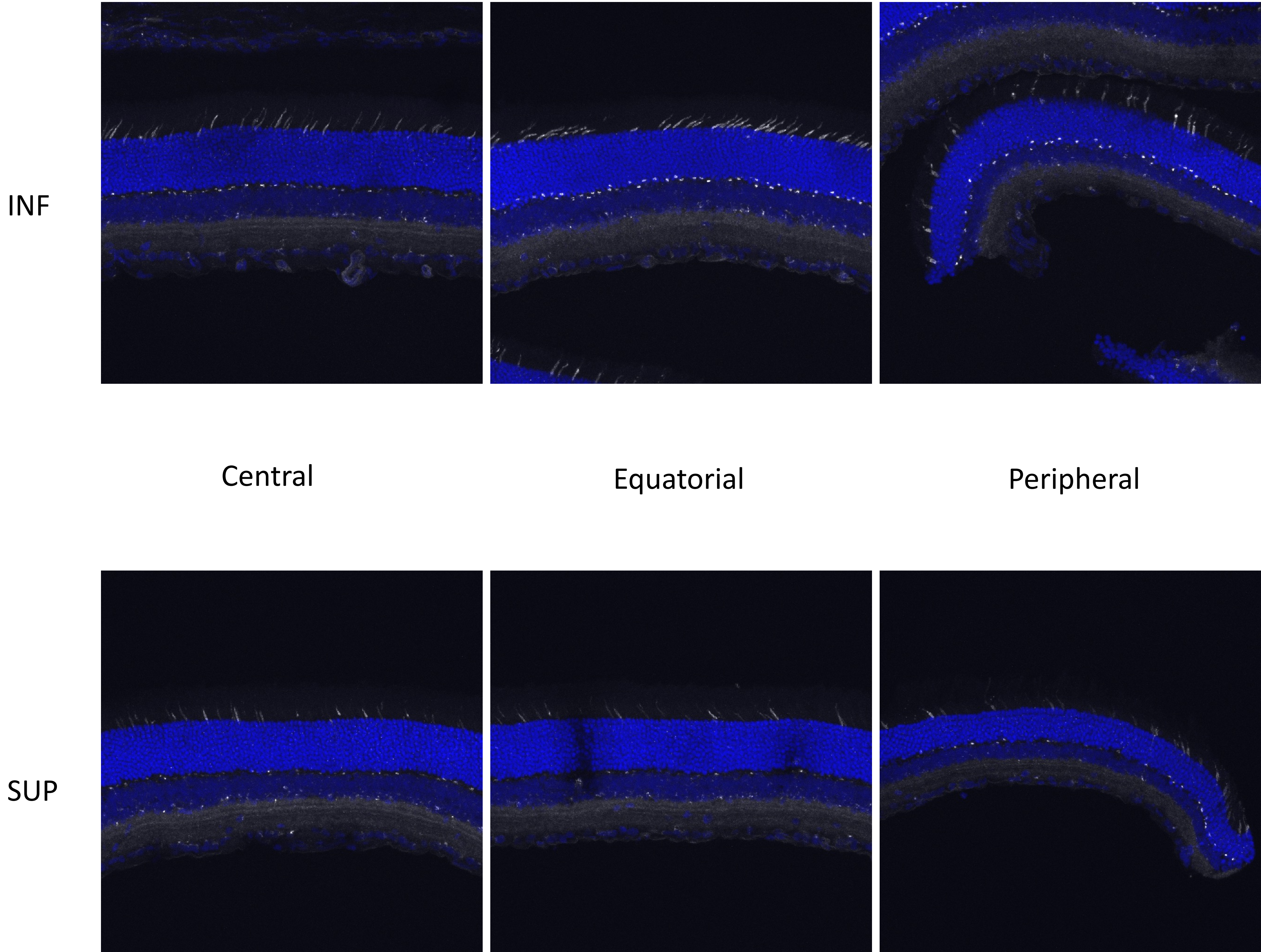

Young8

INF

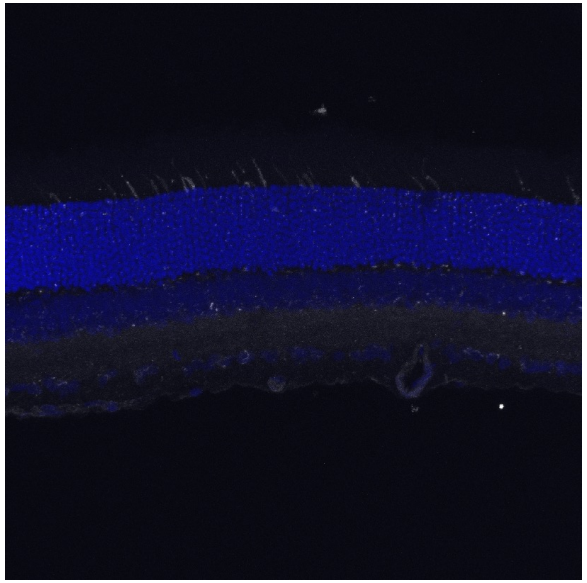

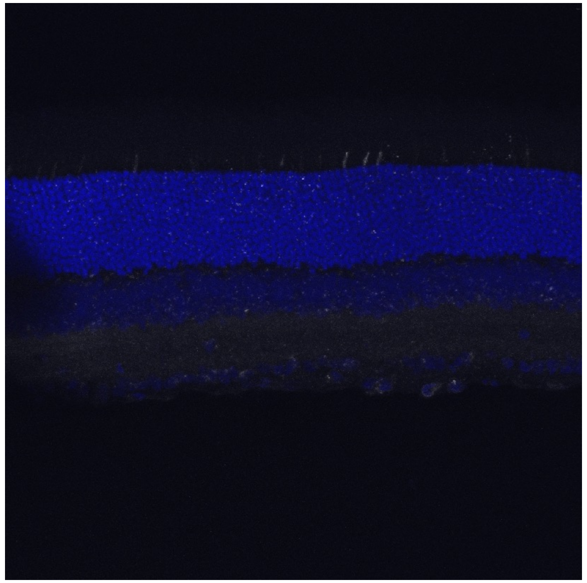

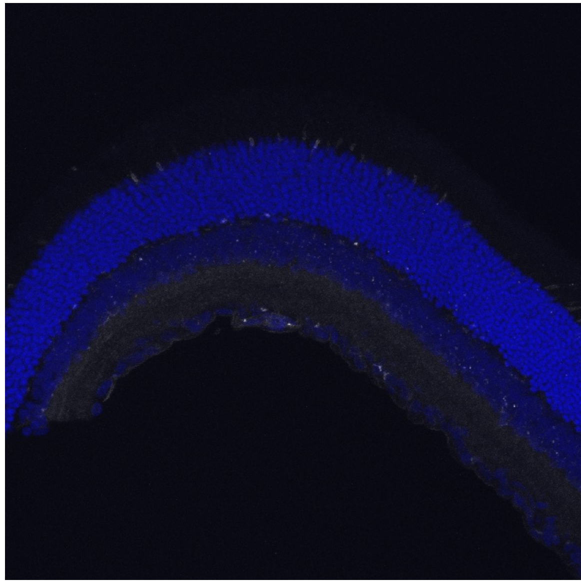

Central

Equatorial

Peripheral

SUP

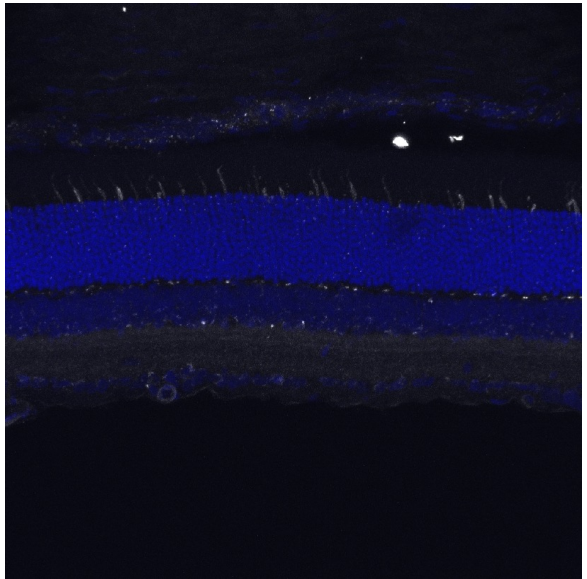

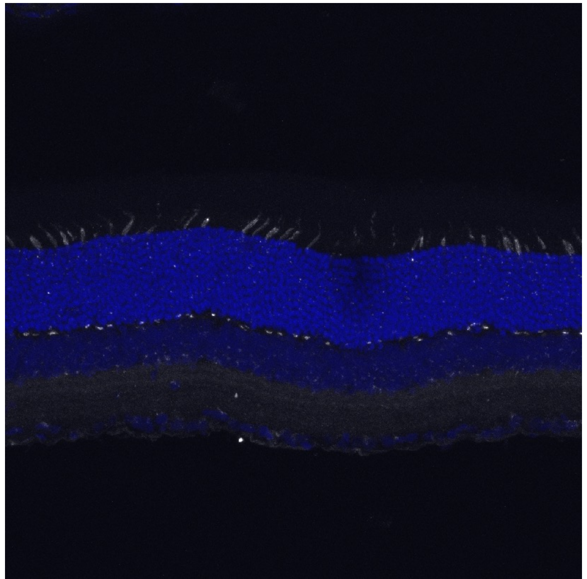

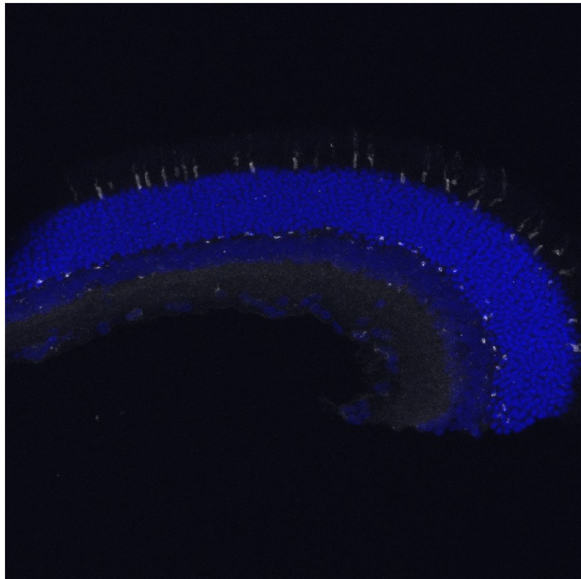

Aged water-treated 1

INF

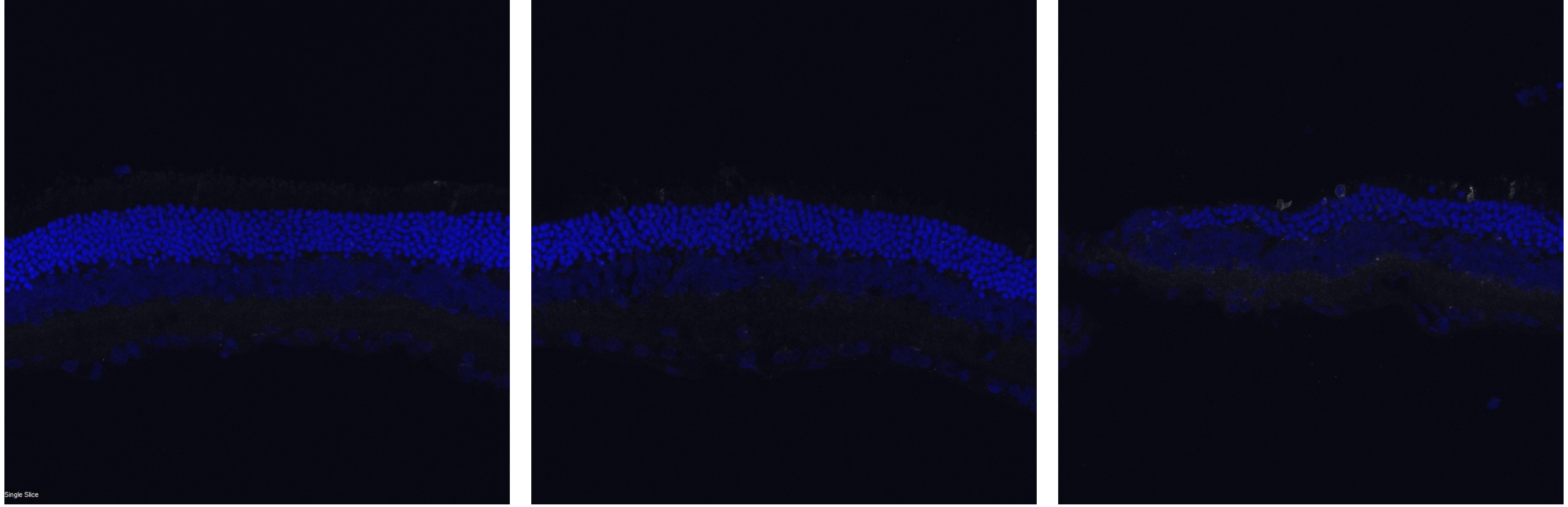

Central

Equatorial

Peripheral

SUP

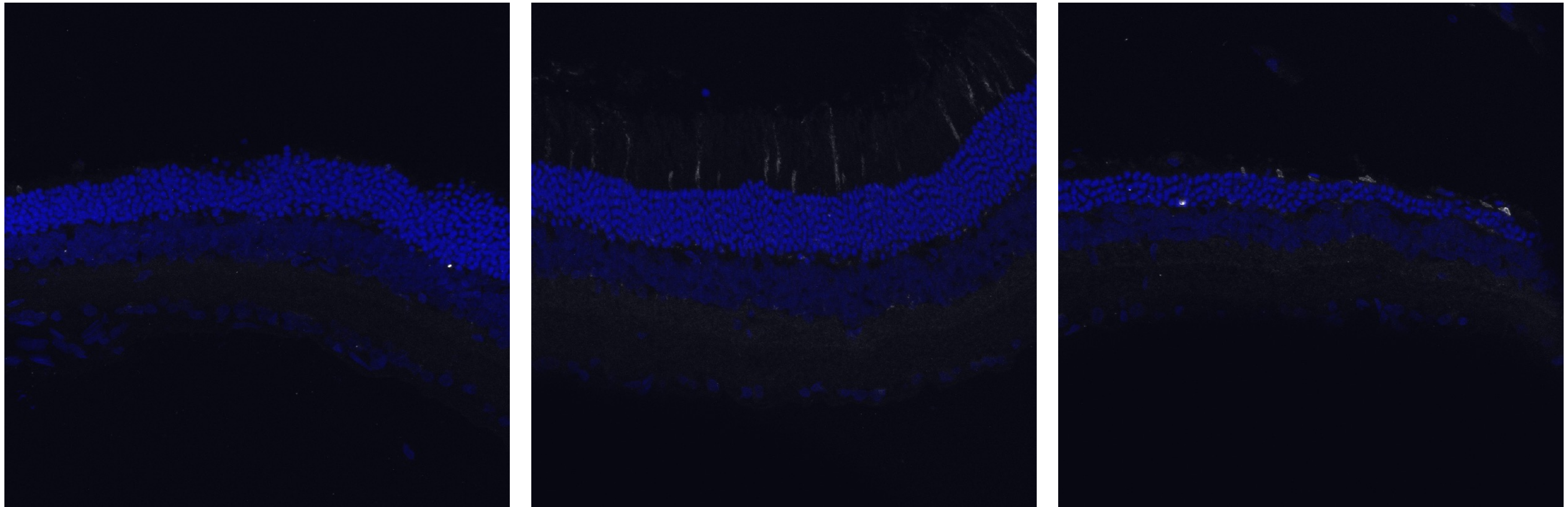

Aged water-treated 2

INF

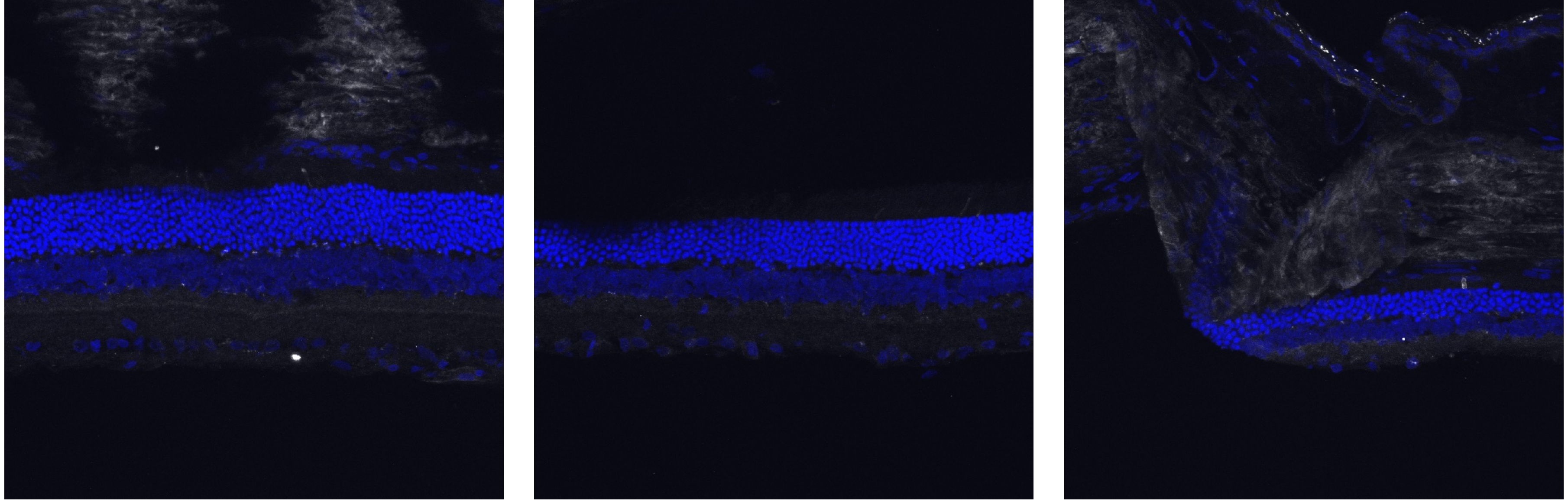

Central | Equatorial | Peripheral

SUP

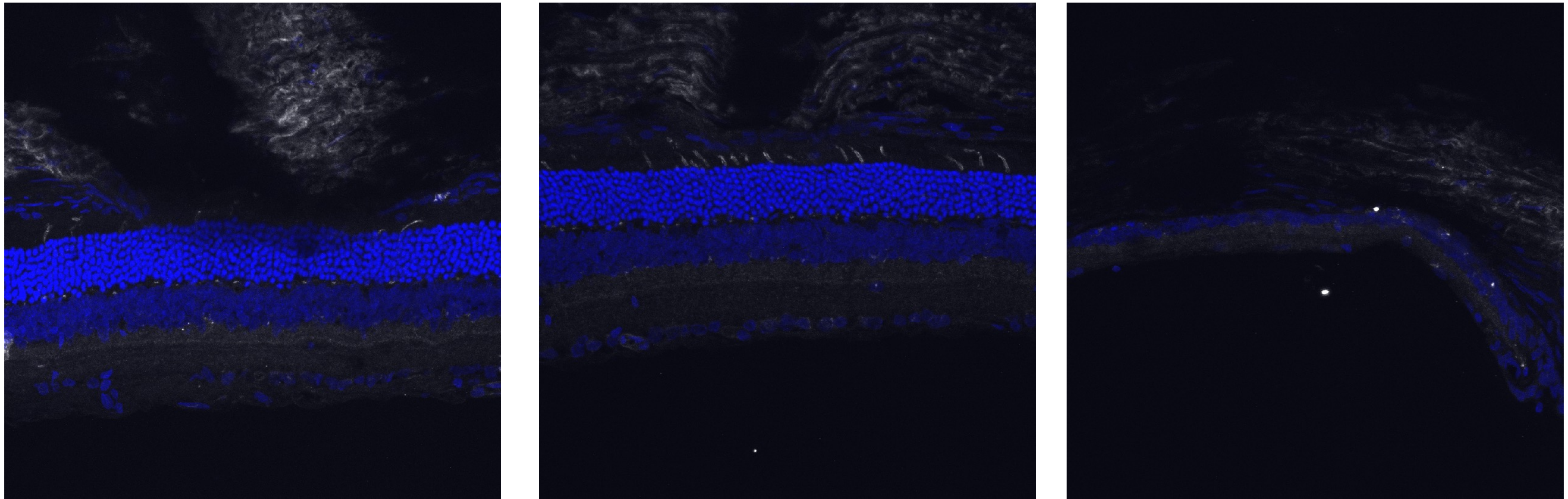

Aged water-treated 3

INF

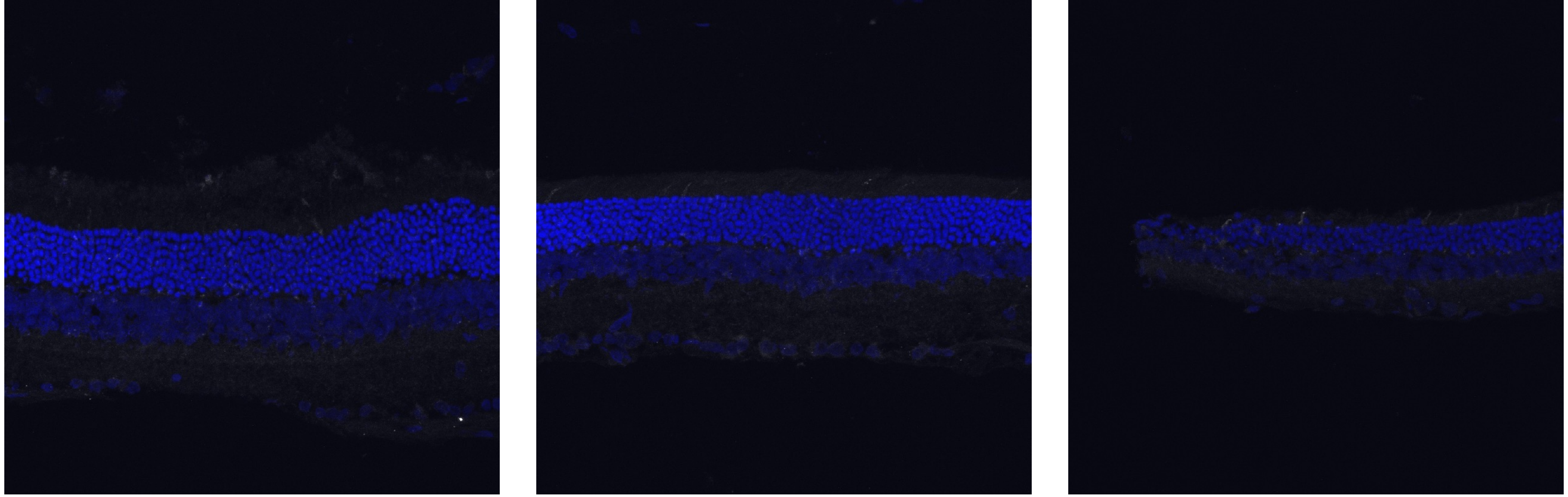

Central Equatorial Peripheral

SUP

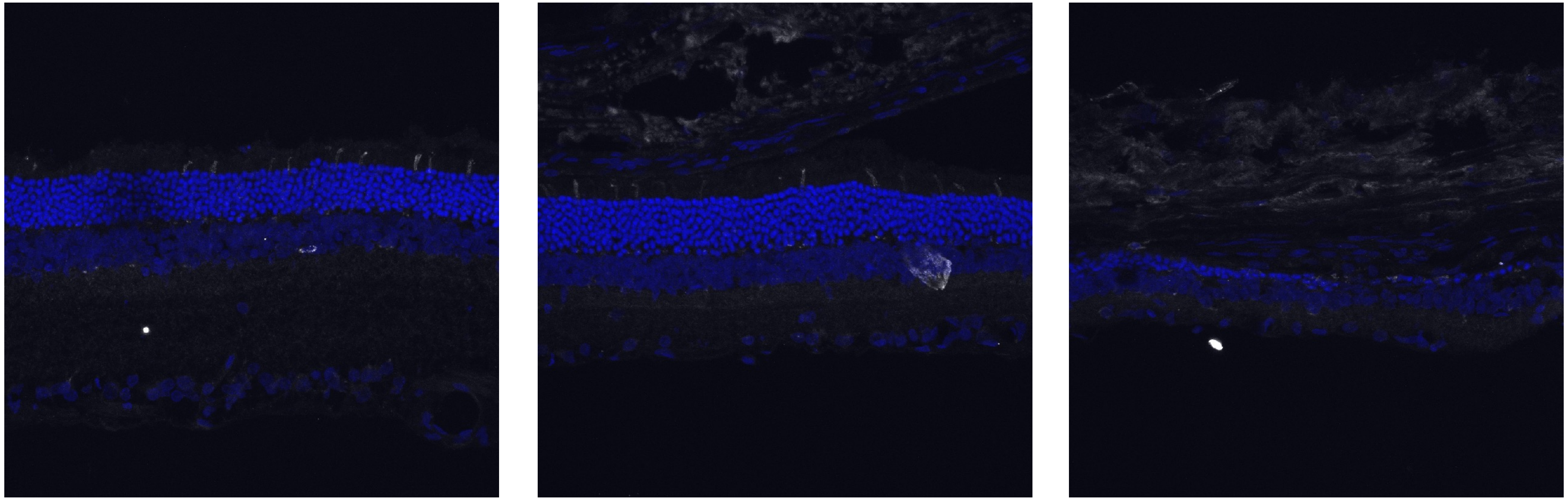

Aged water-treated 4

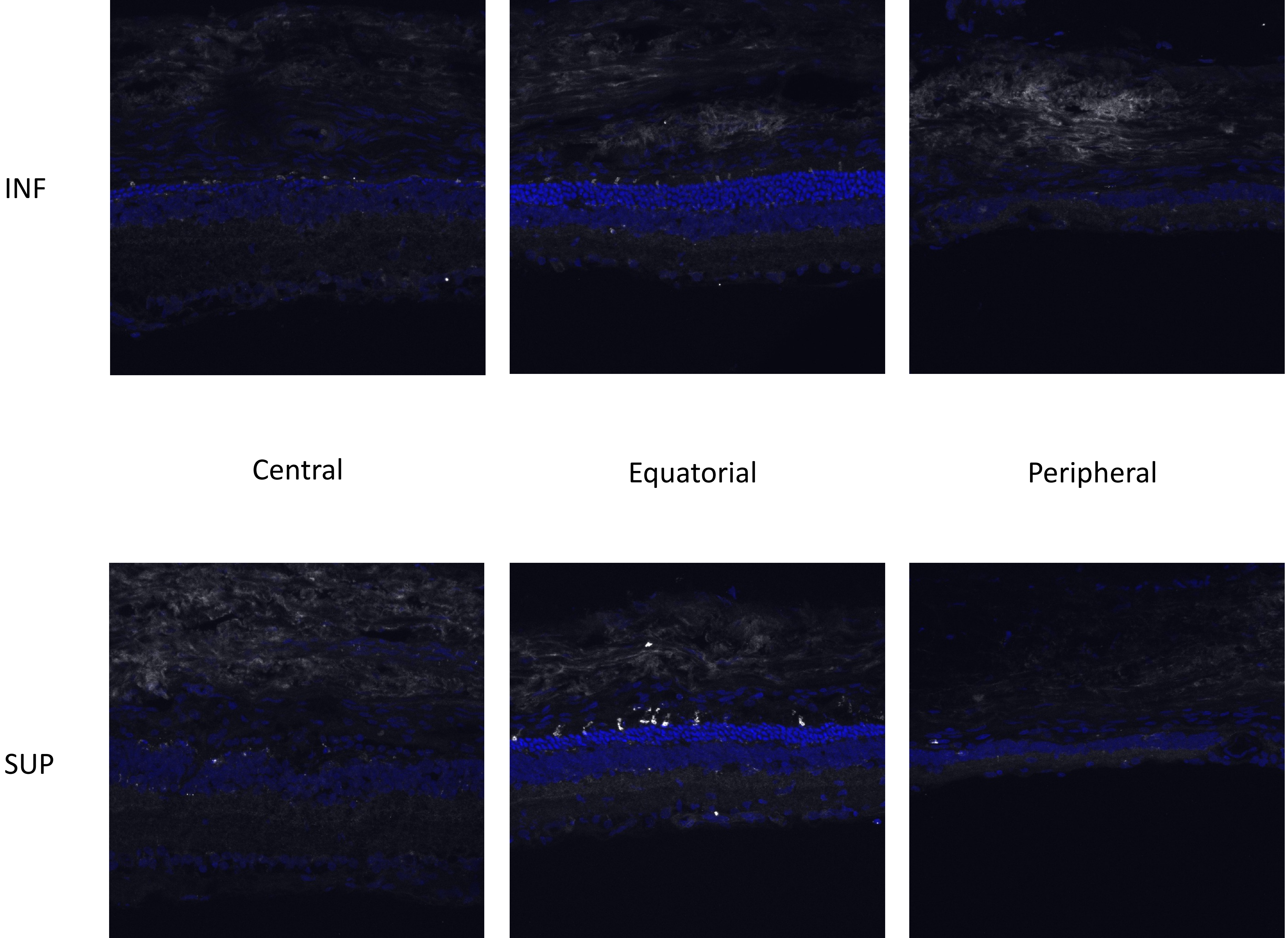

Aged water-treated 5

INF

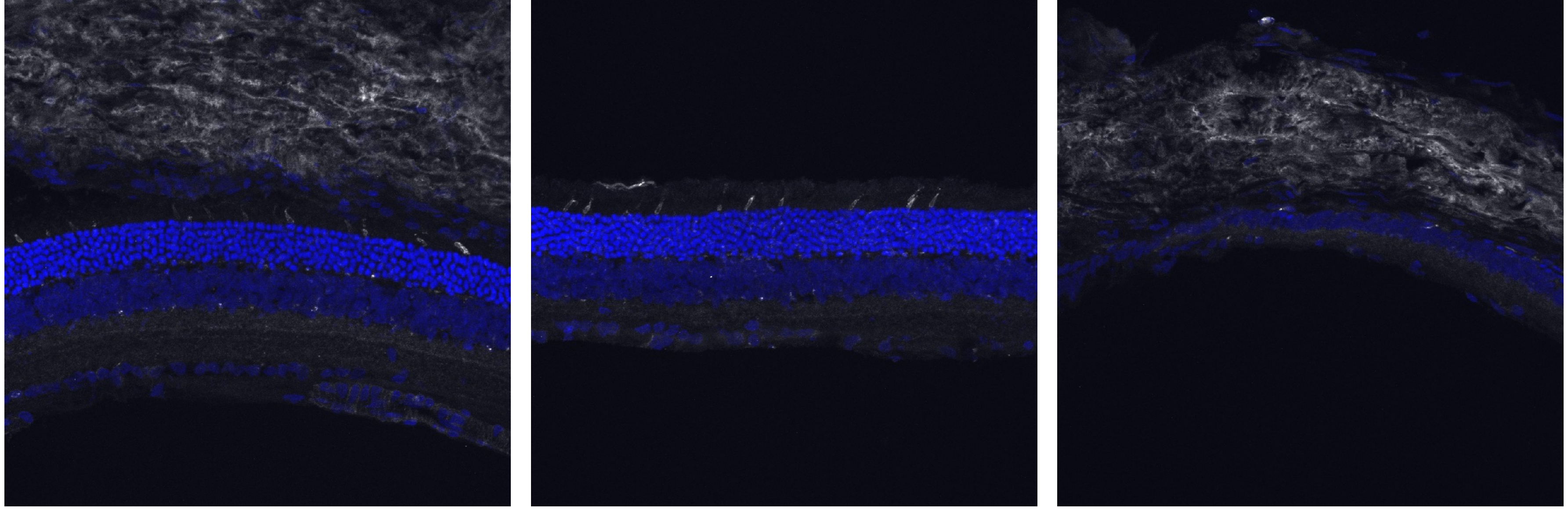

Central

Equatorial

Peripheral

SUP

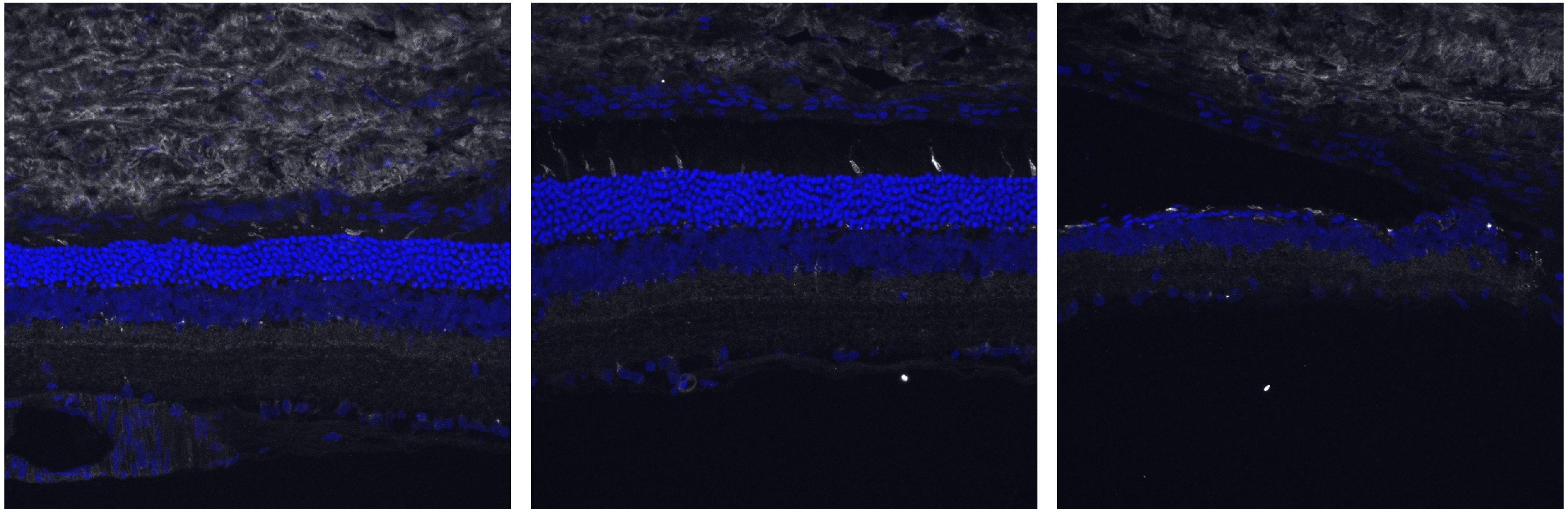

Aged water-treated 6

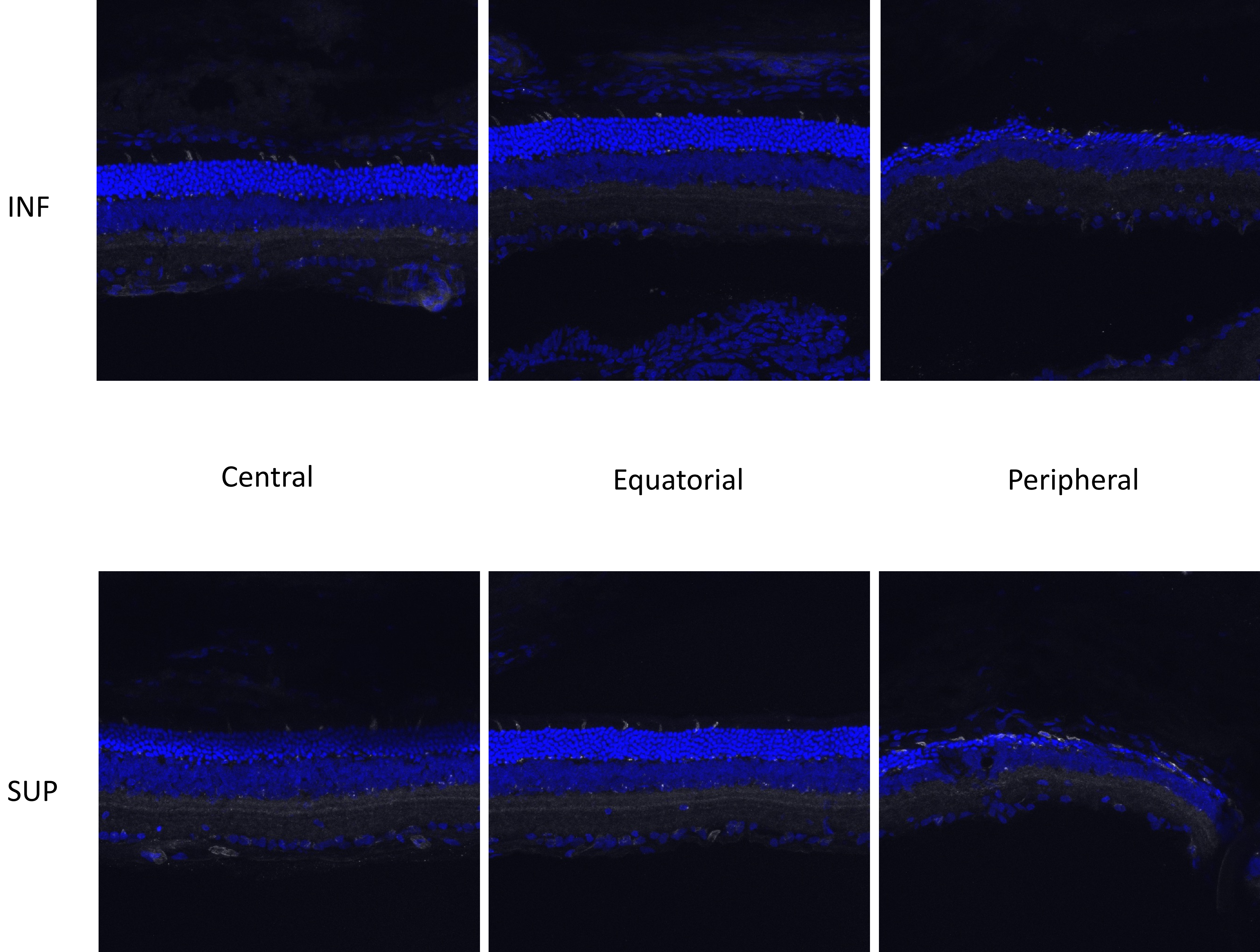

Aged water-treated 7

INF

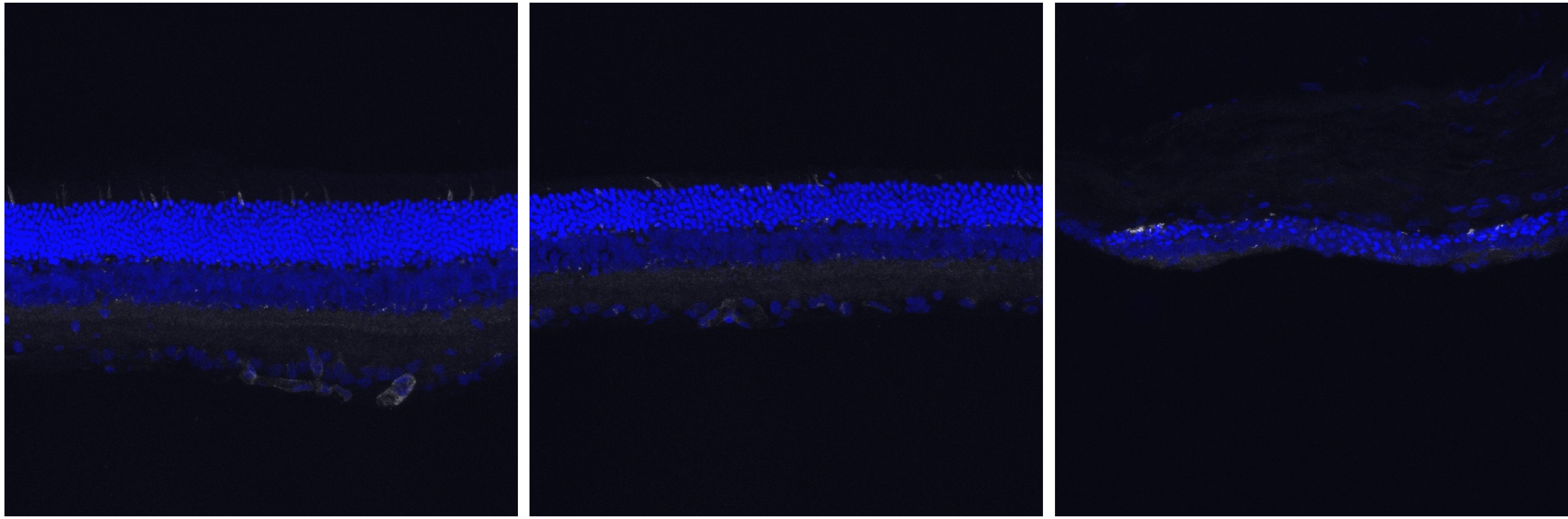

Central

Equatorial

Peripheral

SUP

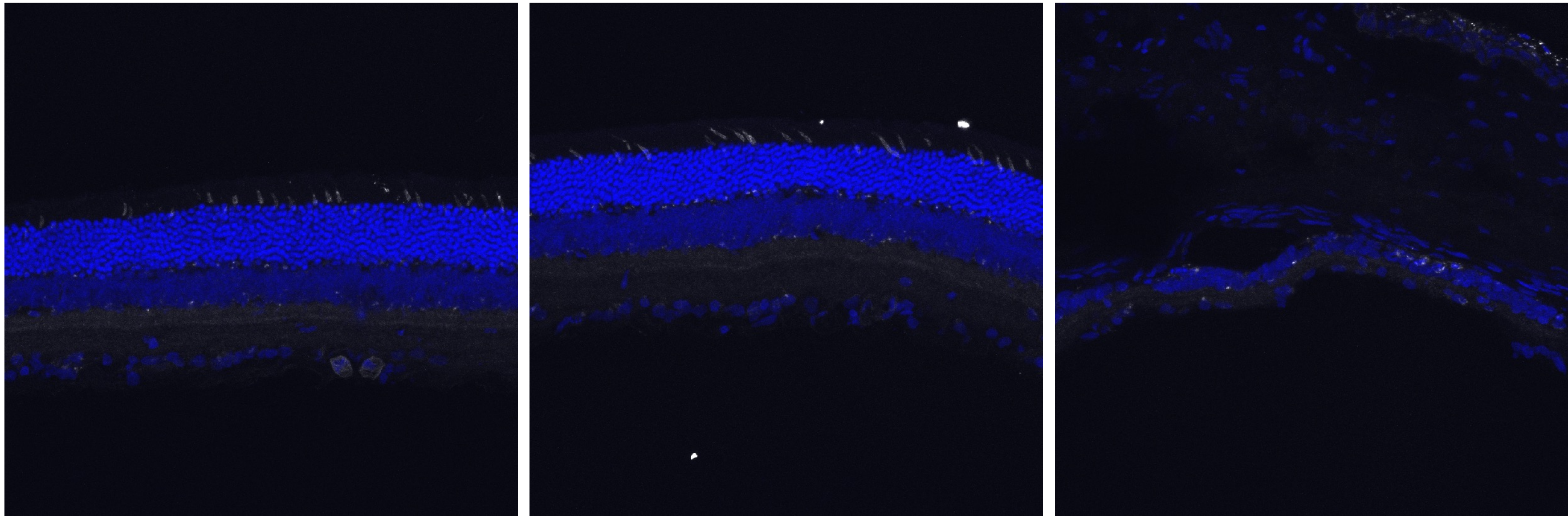

Aged water-treated 8

INF

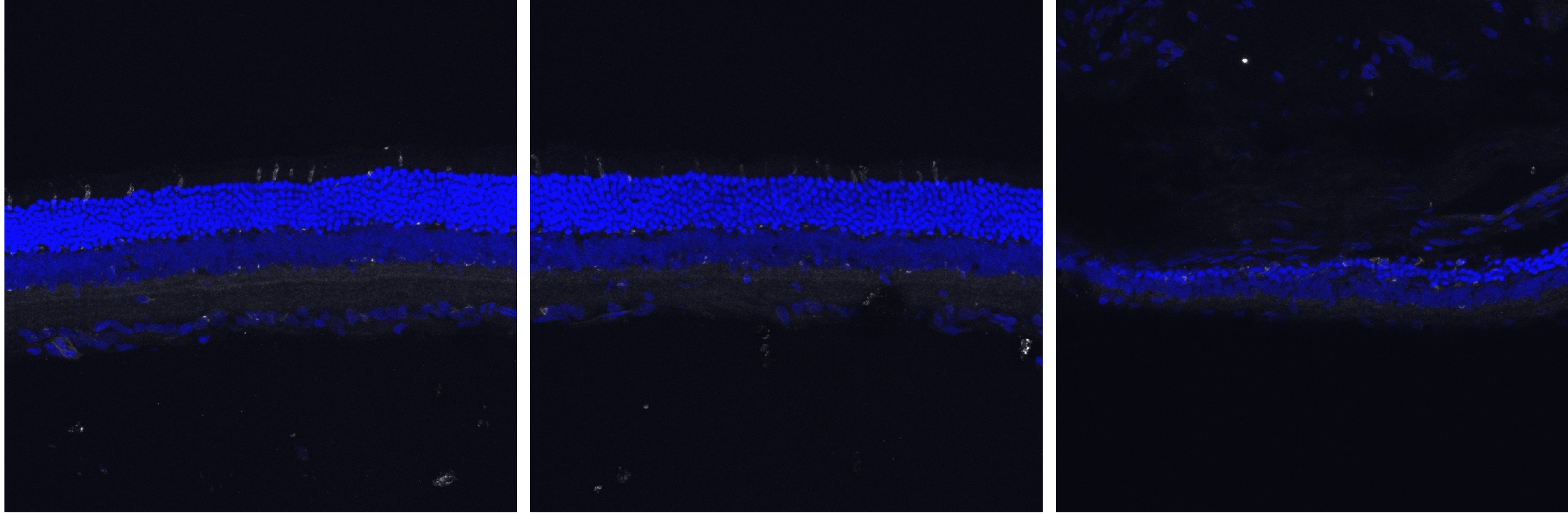

Central Equatorial Peripheral

SUP

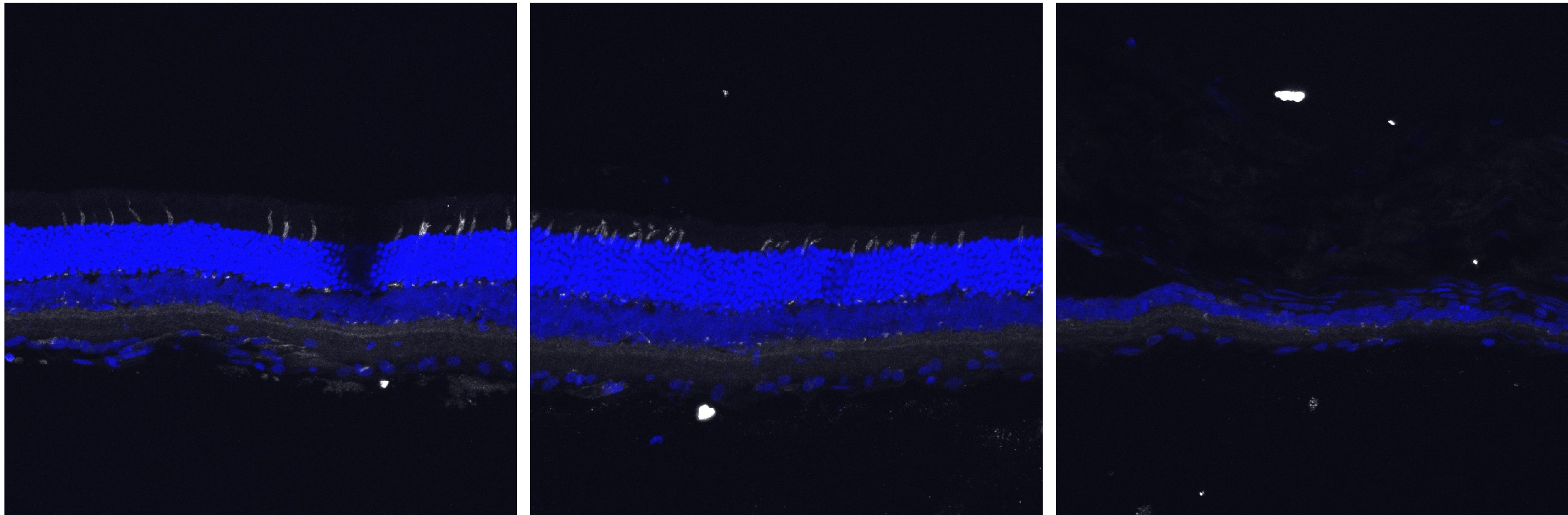

Aged 8AG-treated 1

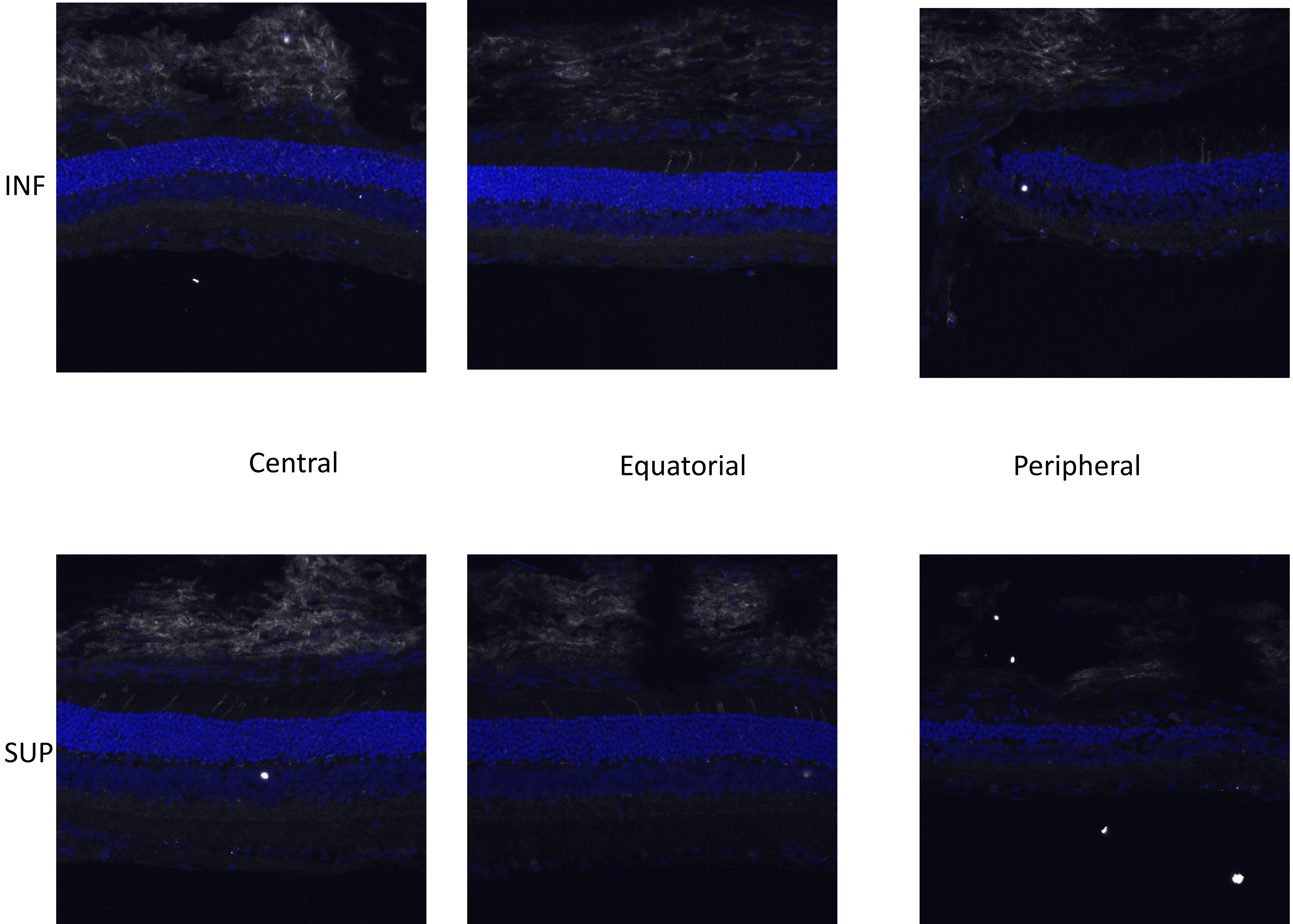

Aged 8AG-treated 2

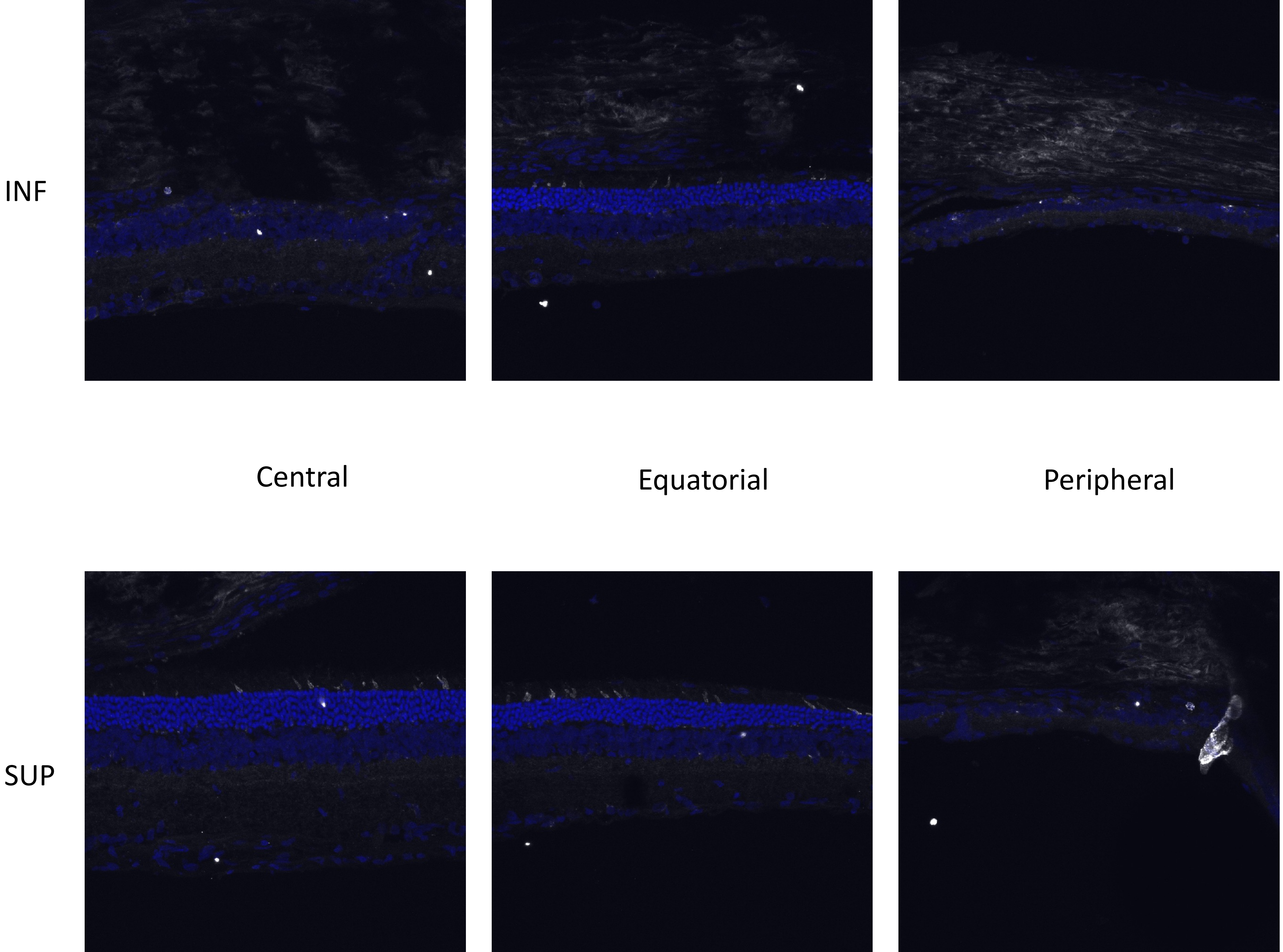

Aged 8AG-treated 3

INF

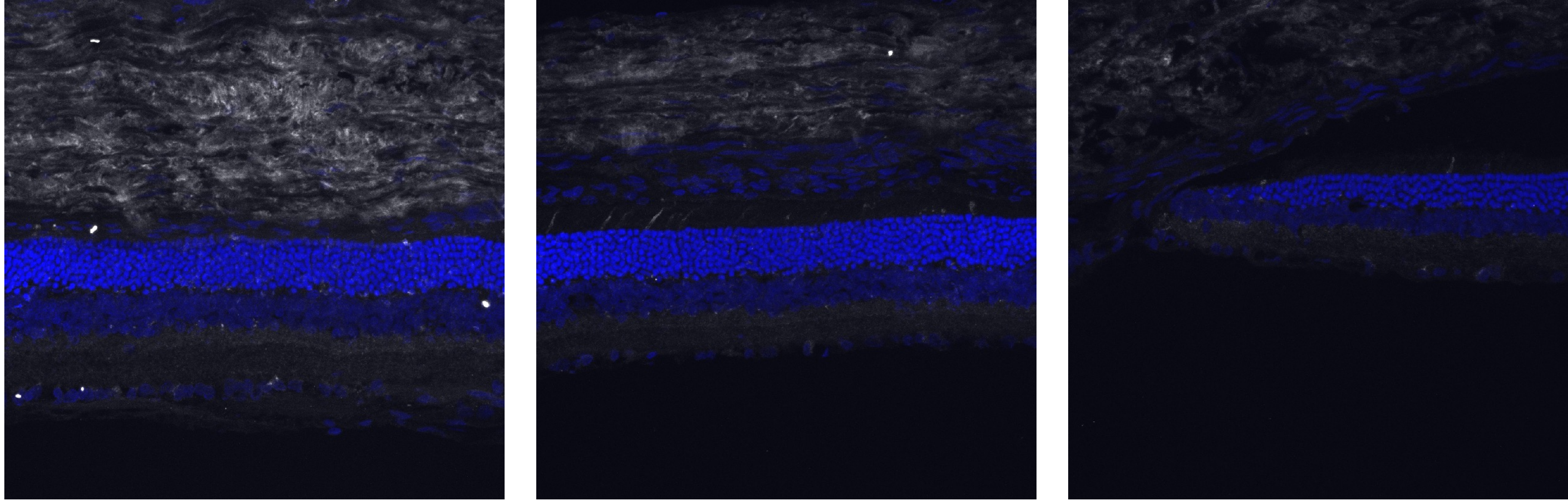

Central

Equatorial

Peripheral

SUP

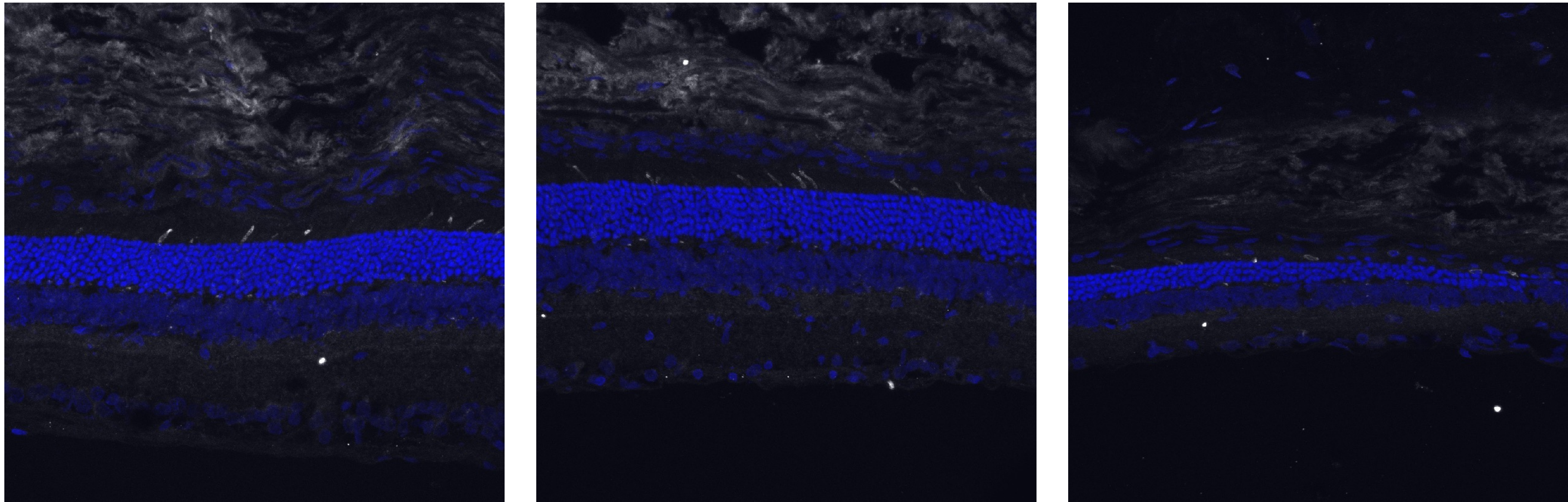

Aged 8AG-treated 4

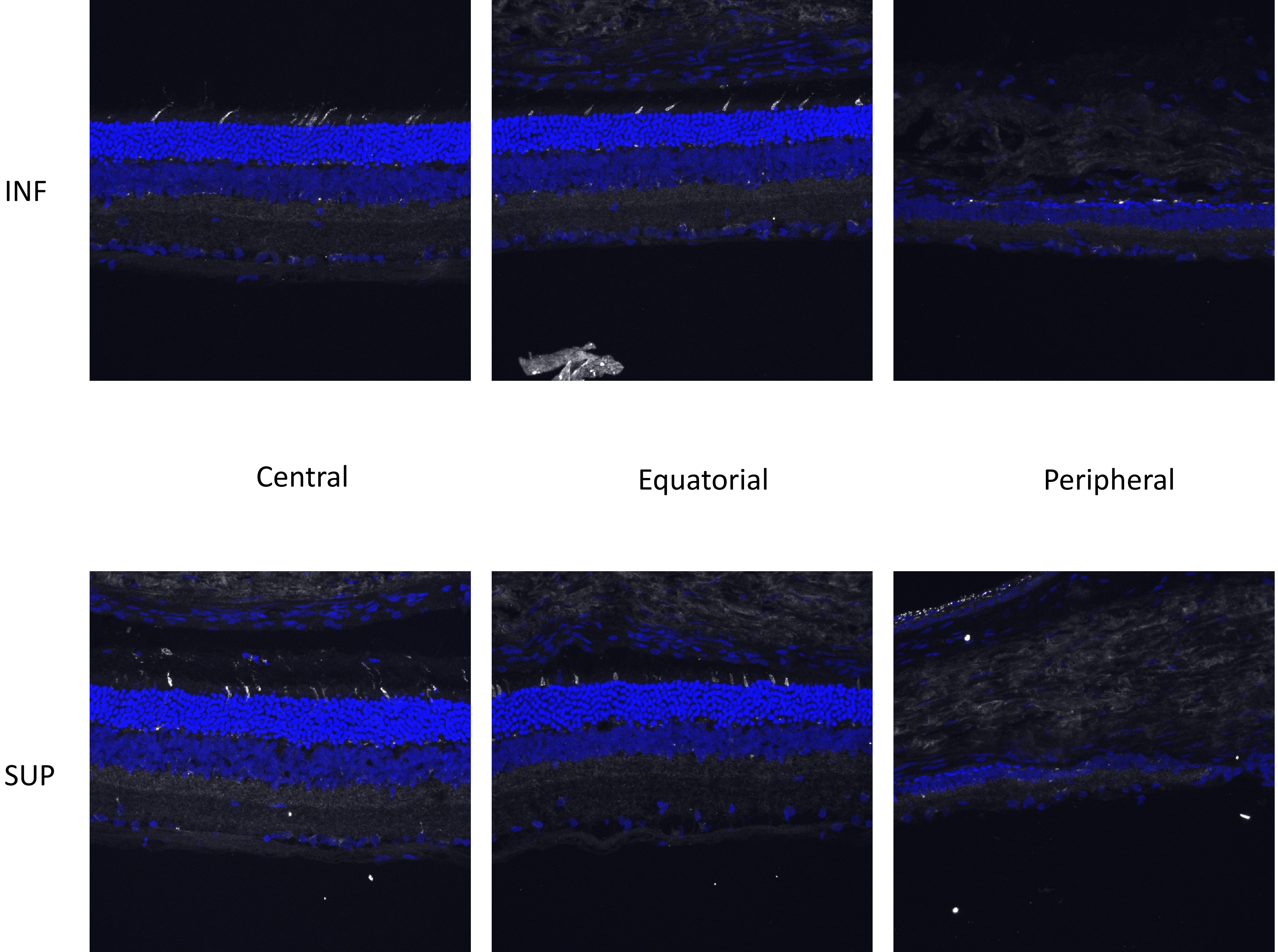

Aged 8AG-treated 5

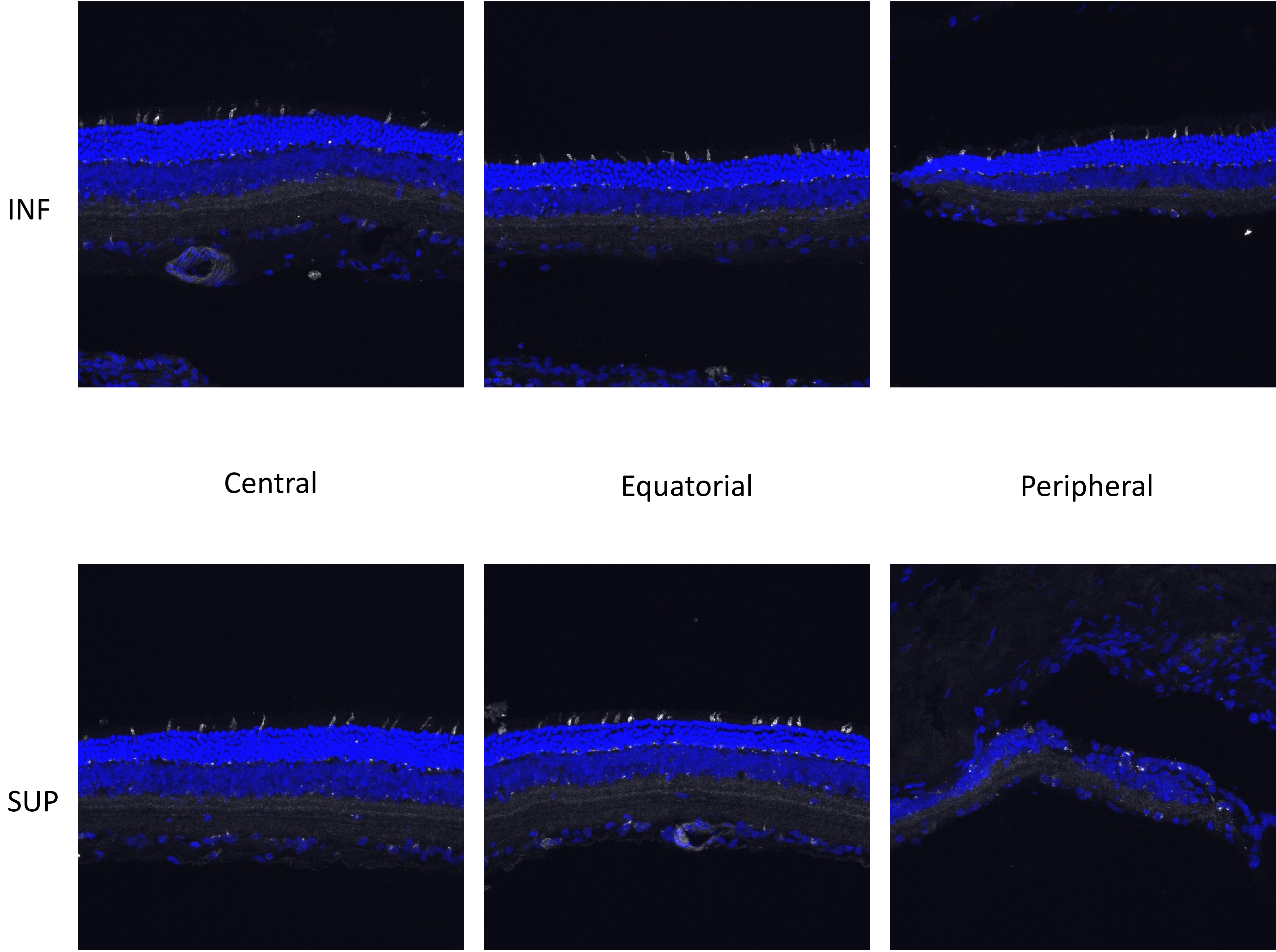

Aged 8AG-treated 6

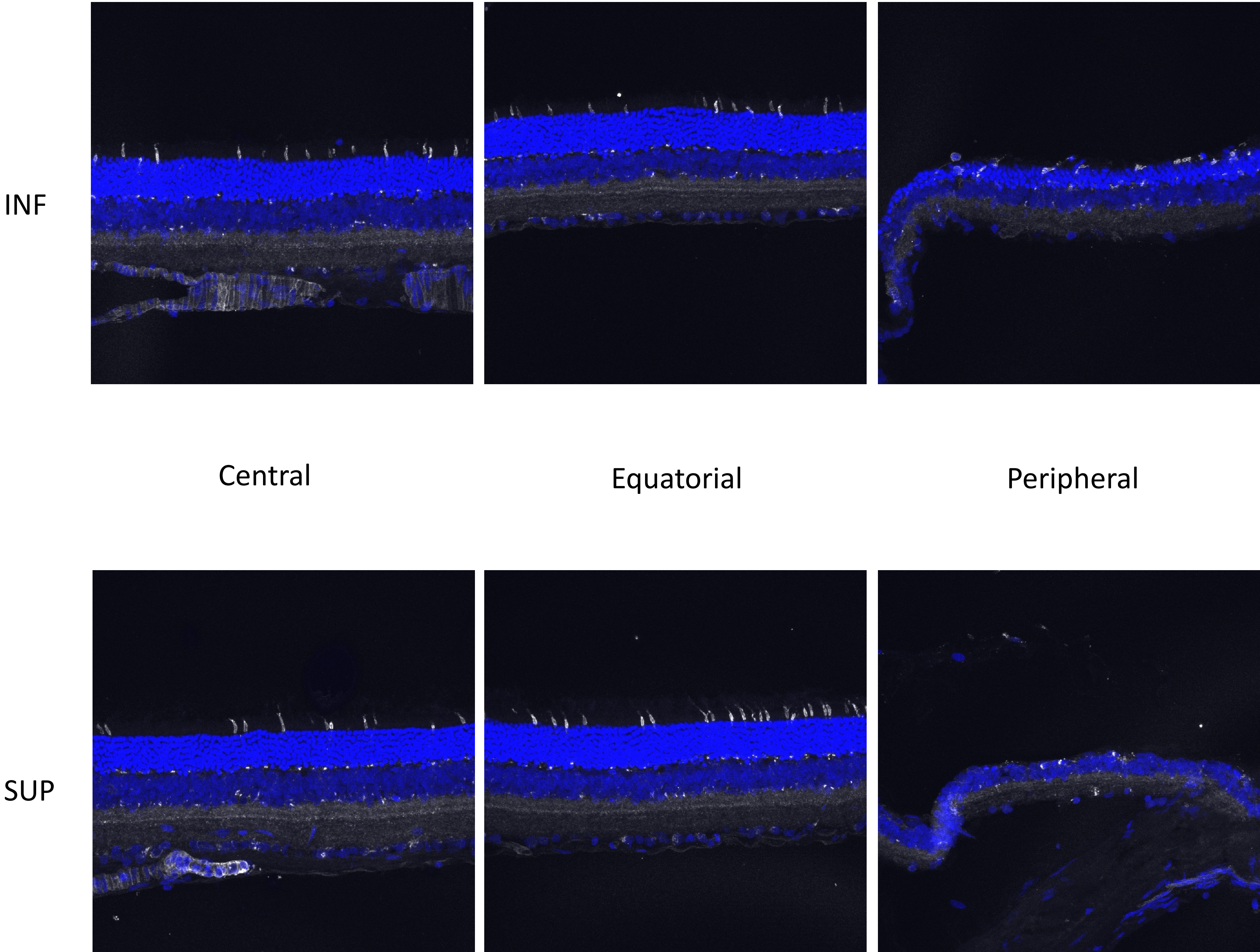

Aged 8AG-treated 7

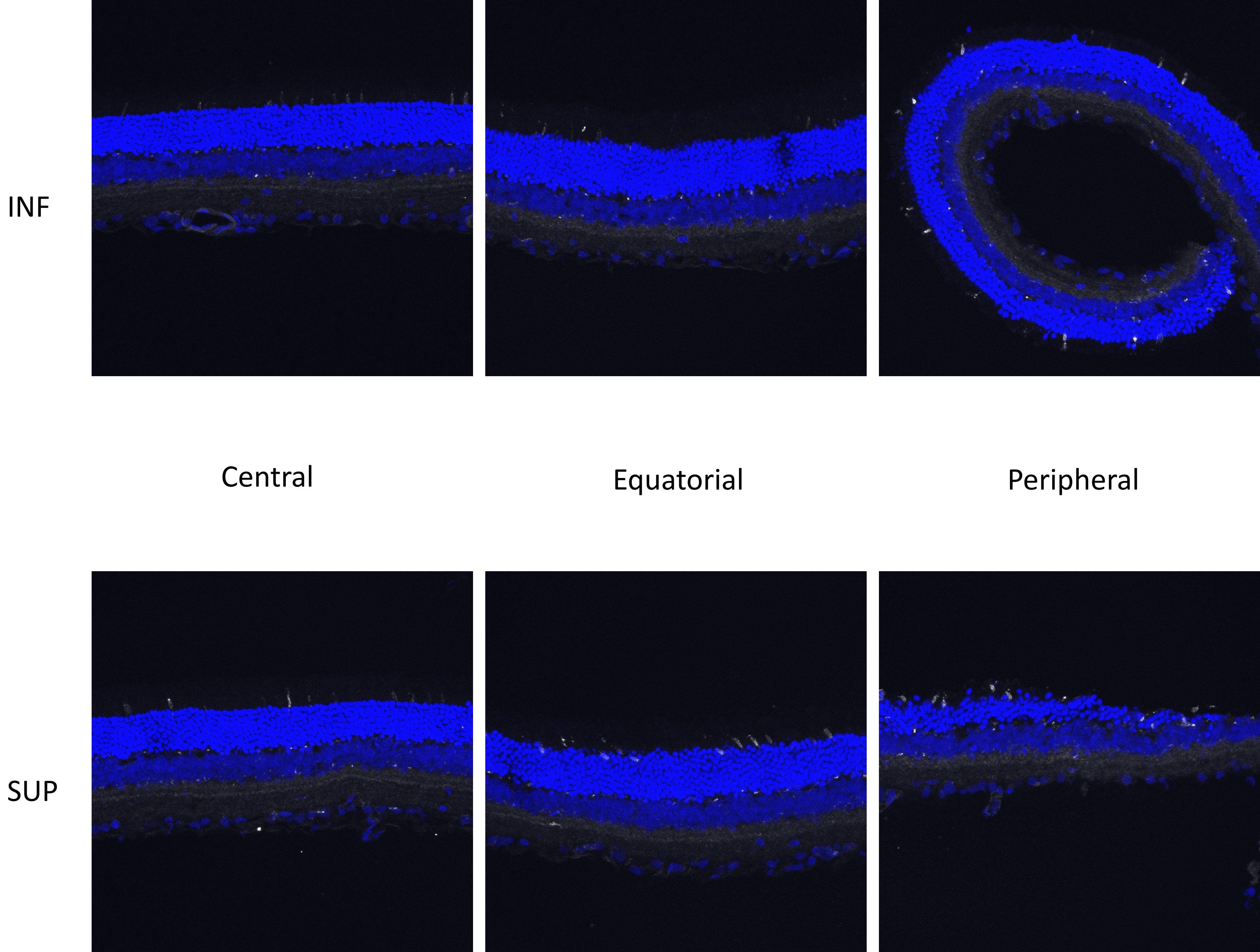

Aged 8AG-treated 8

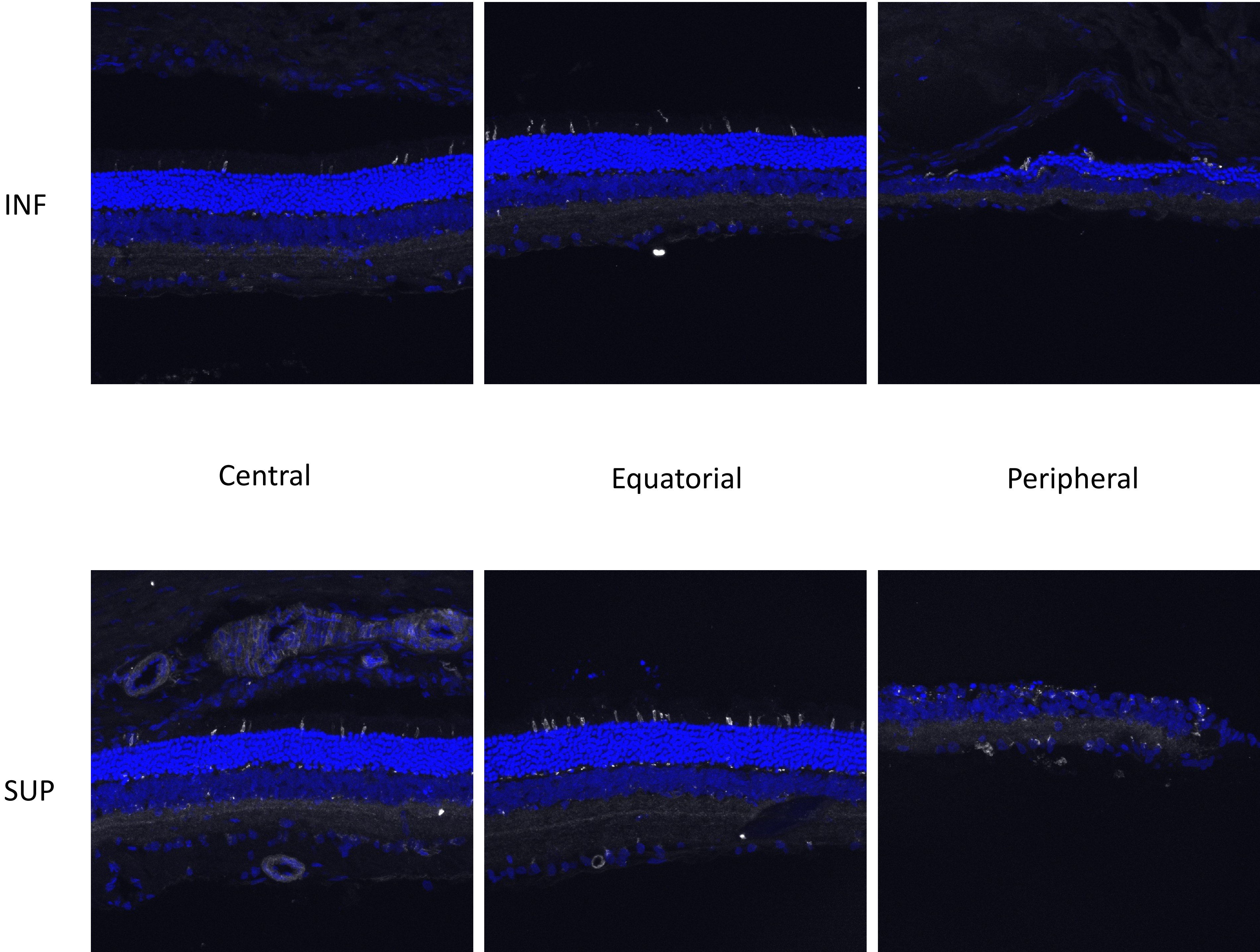

Supplement: Supplementary file 9 — Supplementary Data 7 [file 42003_2025_8242_MOESM9_ESM.pdf]
