# Supplementary material for: Oral 8-aminoguanine against age-related retinal degeneration
Source: Commun Biol. 2025 May 26;8:812. doi: 10.1038/s42003-025-08242-1 (PMC12106806; doi:10.1038/s42003-025-08242-1)

MDA staining for Figure 3A-E

Secondary only control

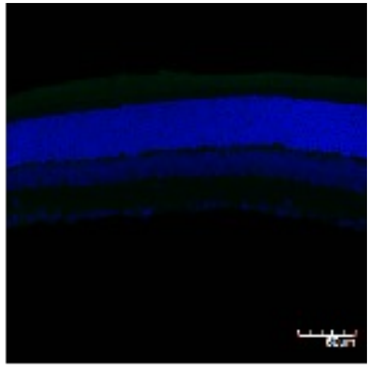

Secondary
only control

Young F344 rat retinae (Biol. repeat 1-3)

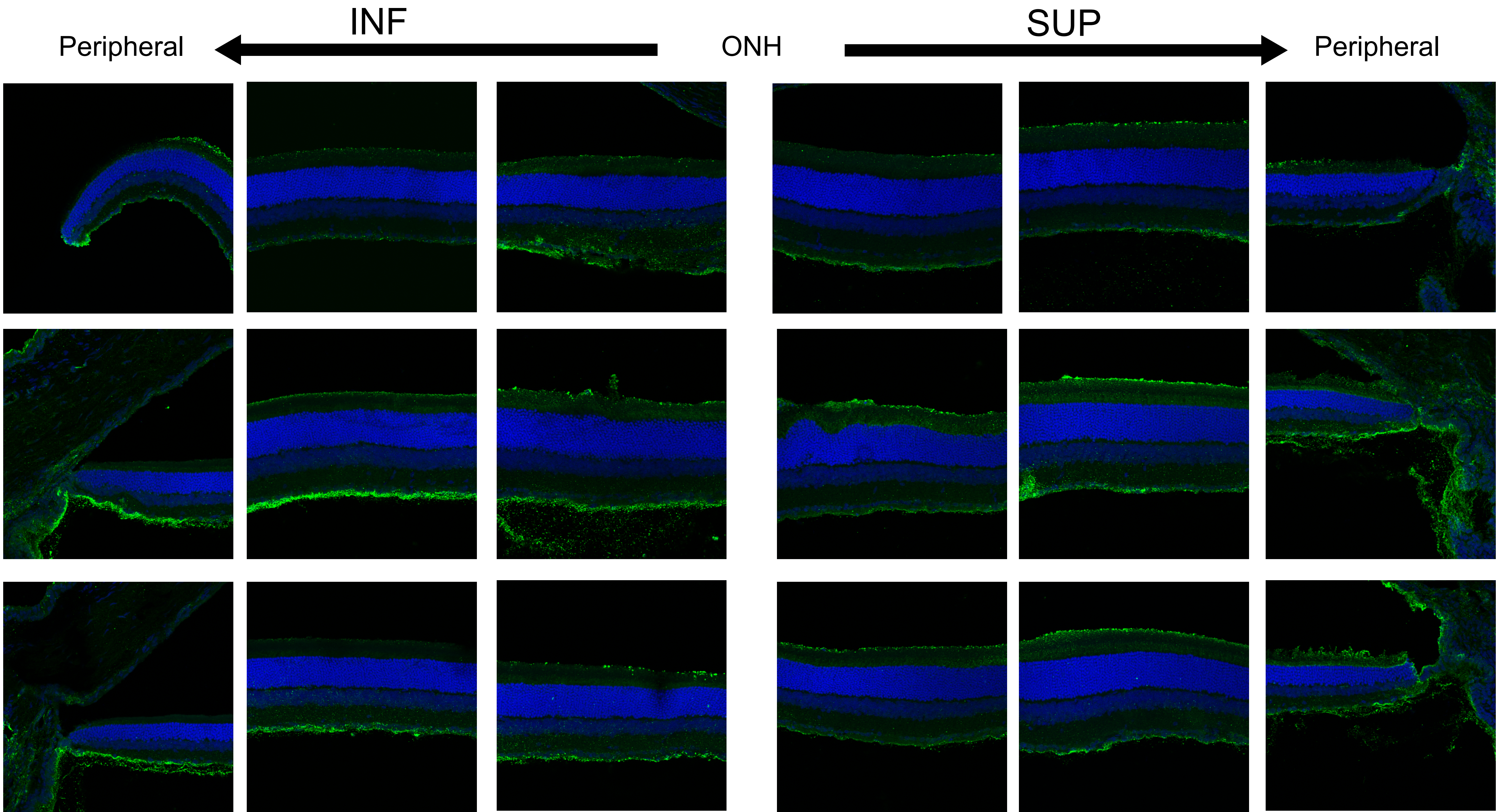

Aged water-treated F344 rat retinae (Biol. Repeat 1-3)

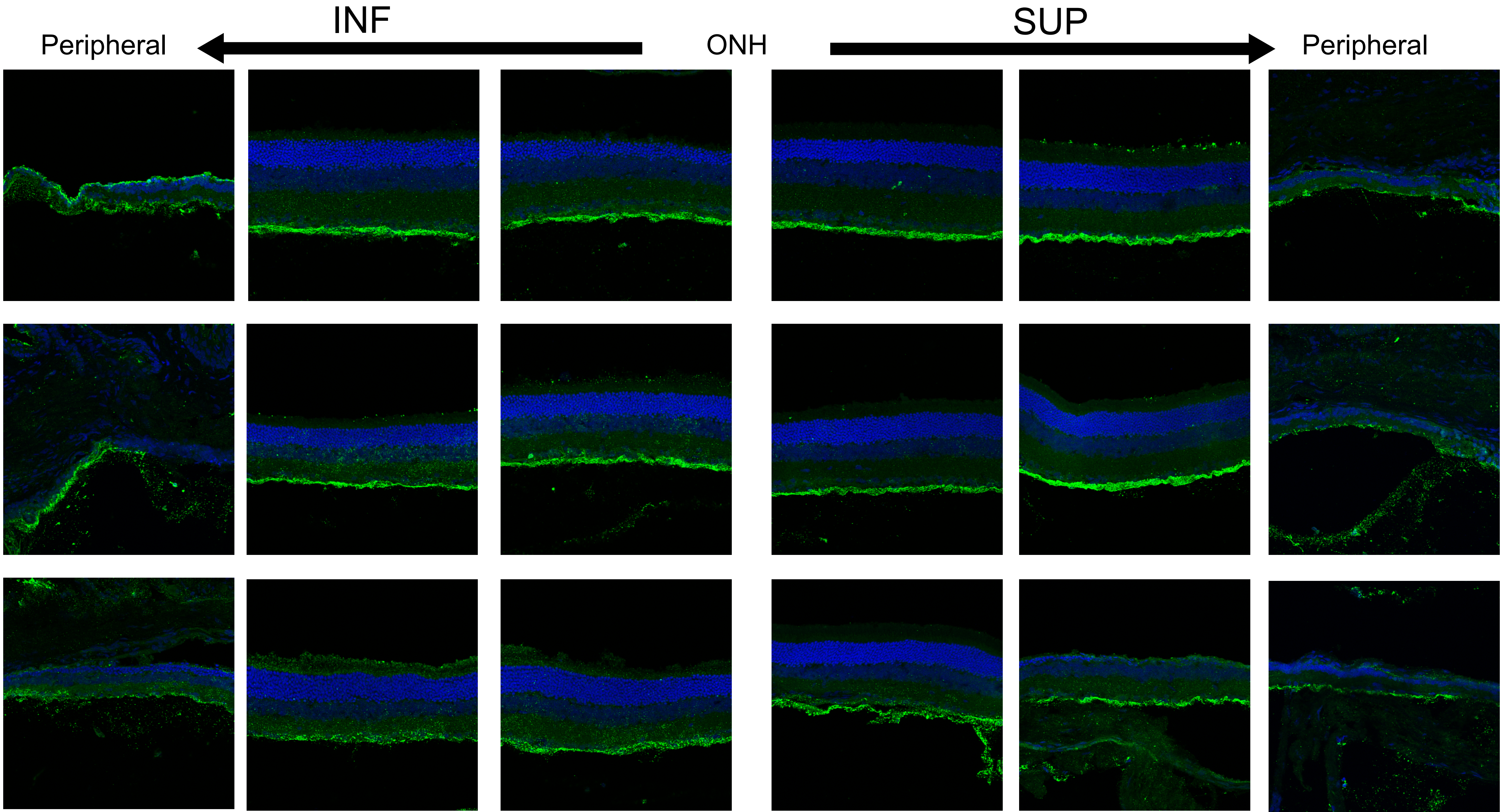

Aged 8AG-treated F344 rat retinae (Biol. Repeat 1-3)

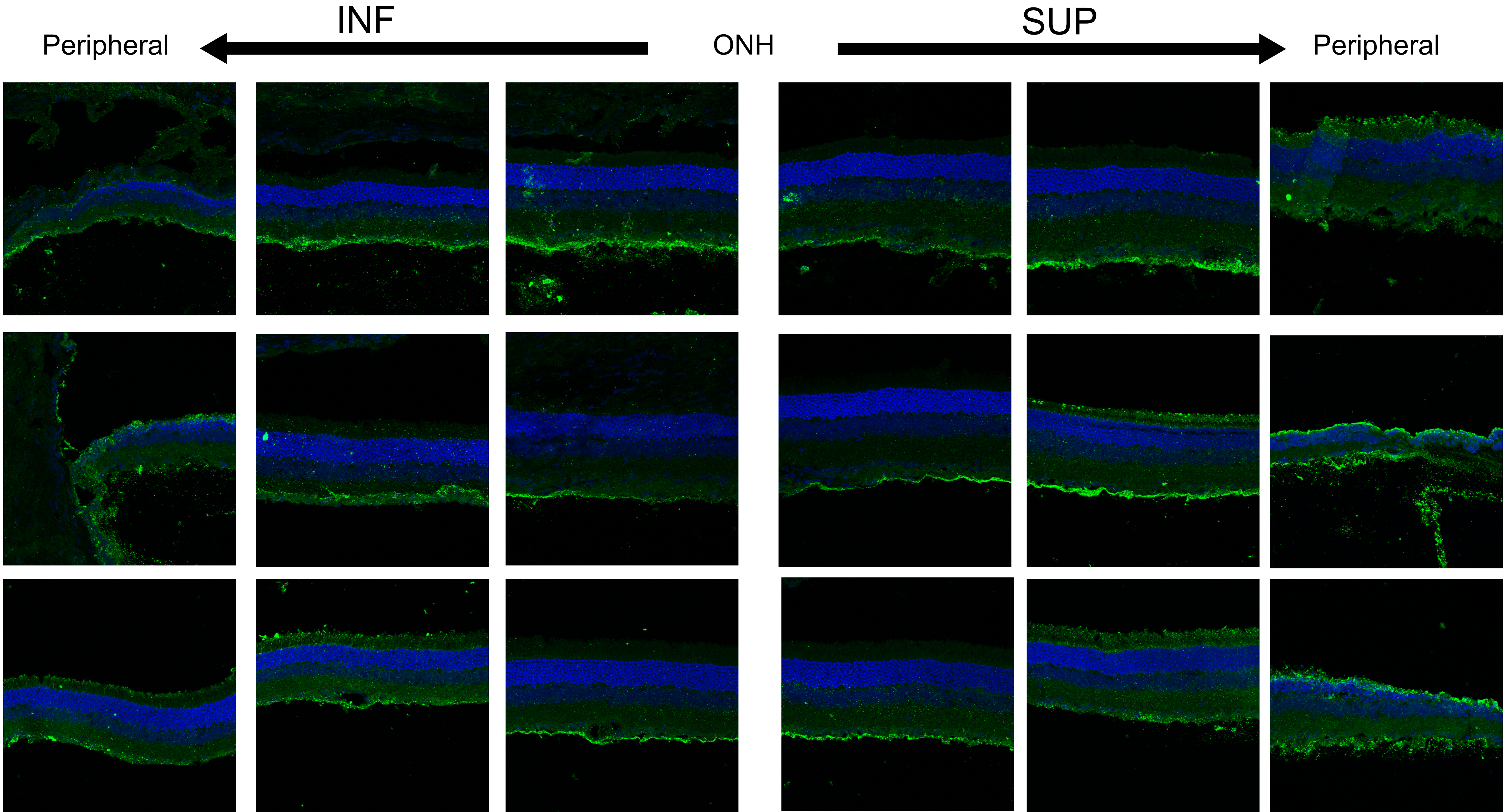

Supplement: Supplementary file 10 — Supplementary Data 8 [file 42003_2025_8242_MOESM10_ESM.pdf]
