# Supplementary material for: Oral 8-aminoguanine against age-related retinal degeneration
Source: Commun Biol. 2025 May 26;8:812. doi: 10.1038/s42003-025-08242-1 (PMC12106806; doi:10.1038/s42003-025-08242-1)
Supplement: Supplementary file 11 — Supplementary Data 9 [file 42003_2025_8242_MOESM11_ESM.pdf]

IHC of 8OH dG and TOMM20 for Figure 3F-R

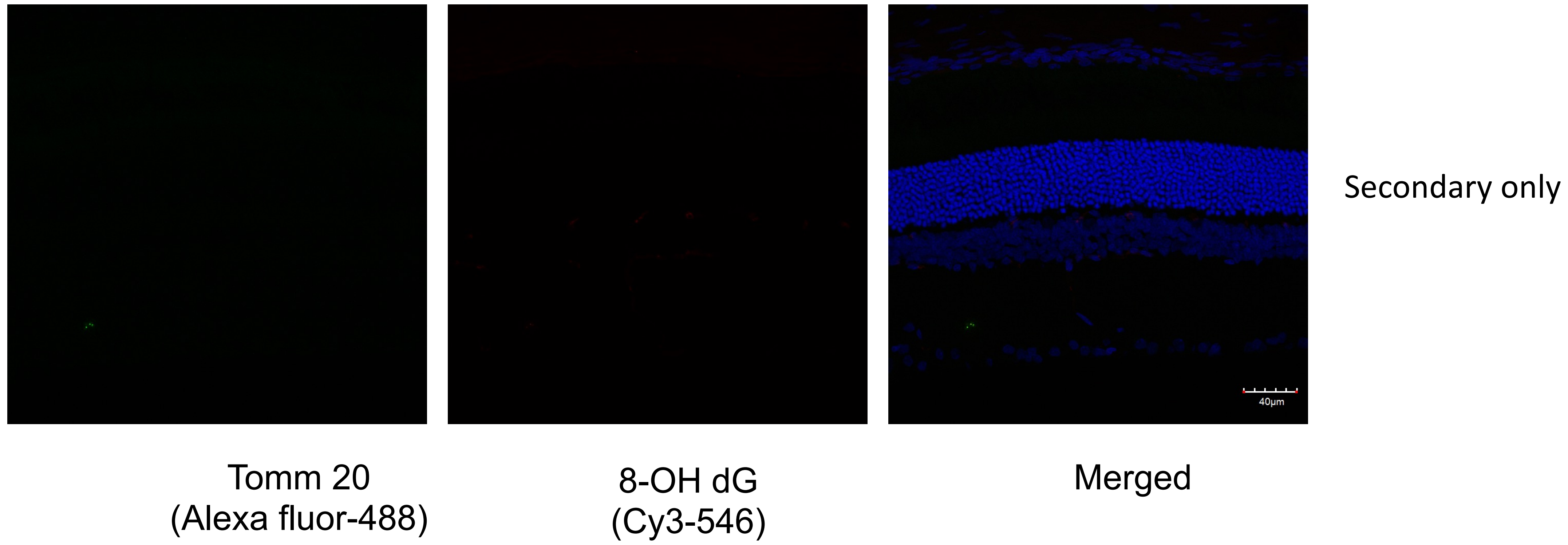

AGED 8-AG TREATED RAT 8_8-OH dG

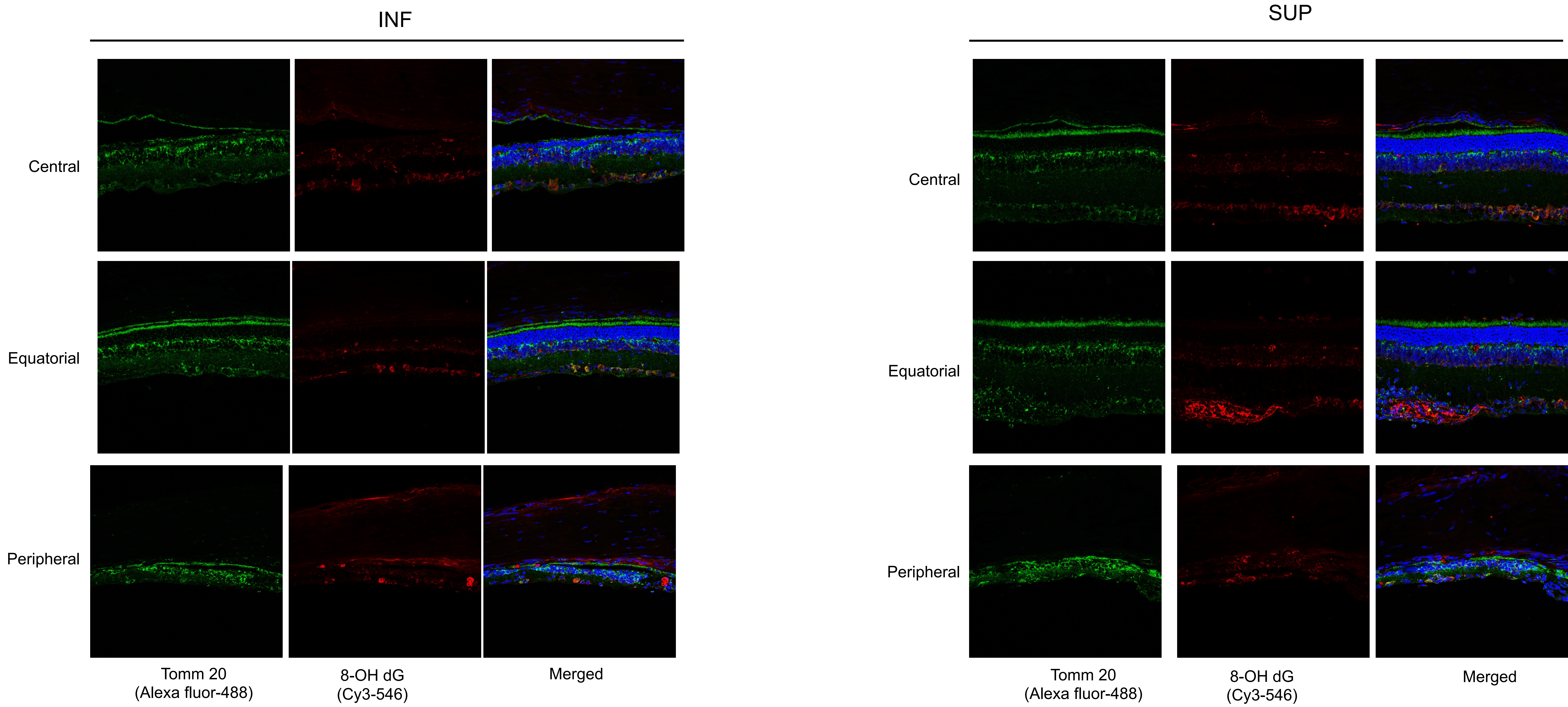

AGED 8-AG TREATED RAT 9_8-OH dG

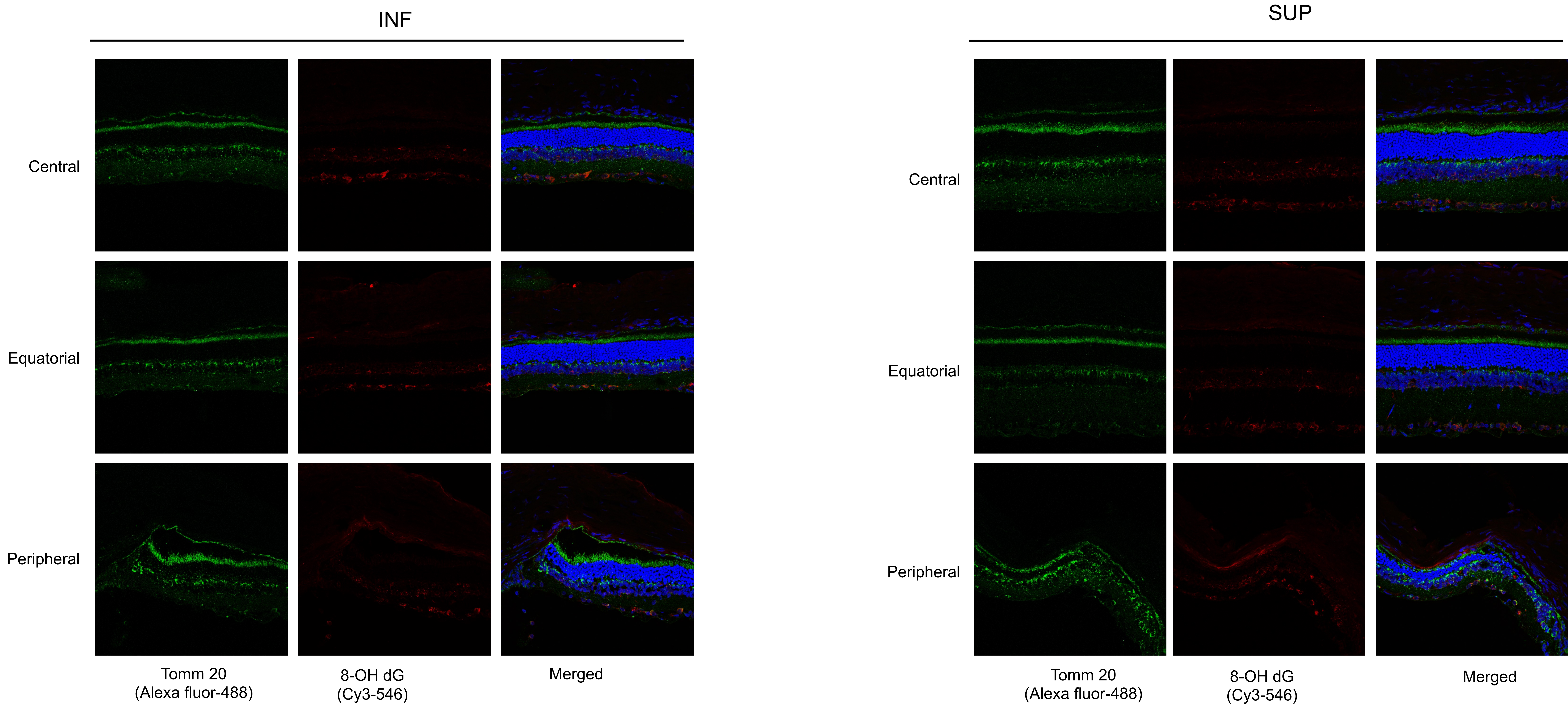

AGED 8-AG TREATED RAT 12_8-OH dG

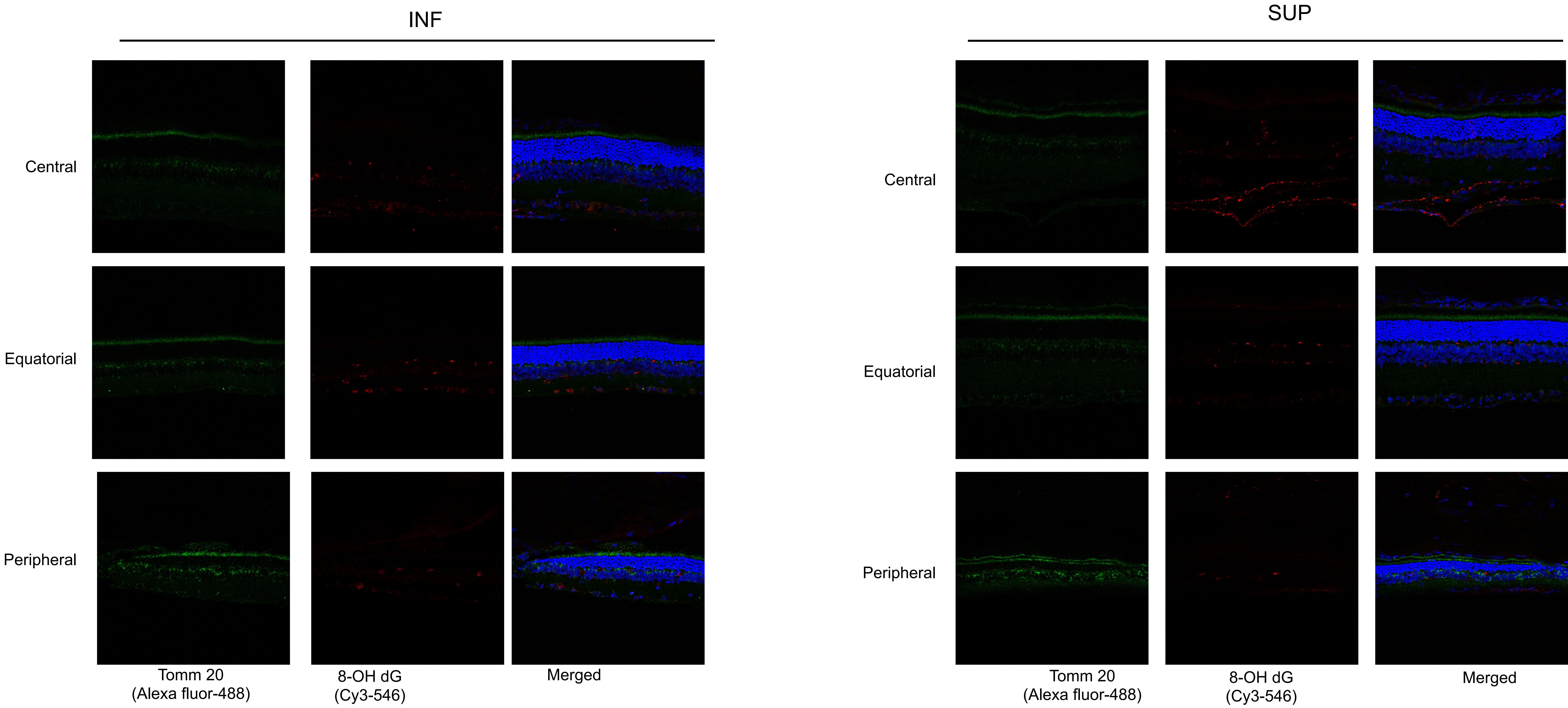

AGED 8-AG TREATED RAT 13_8-OH dG

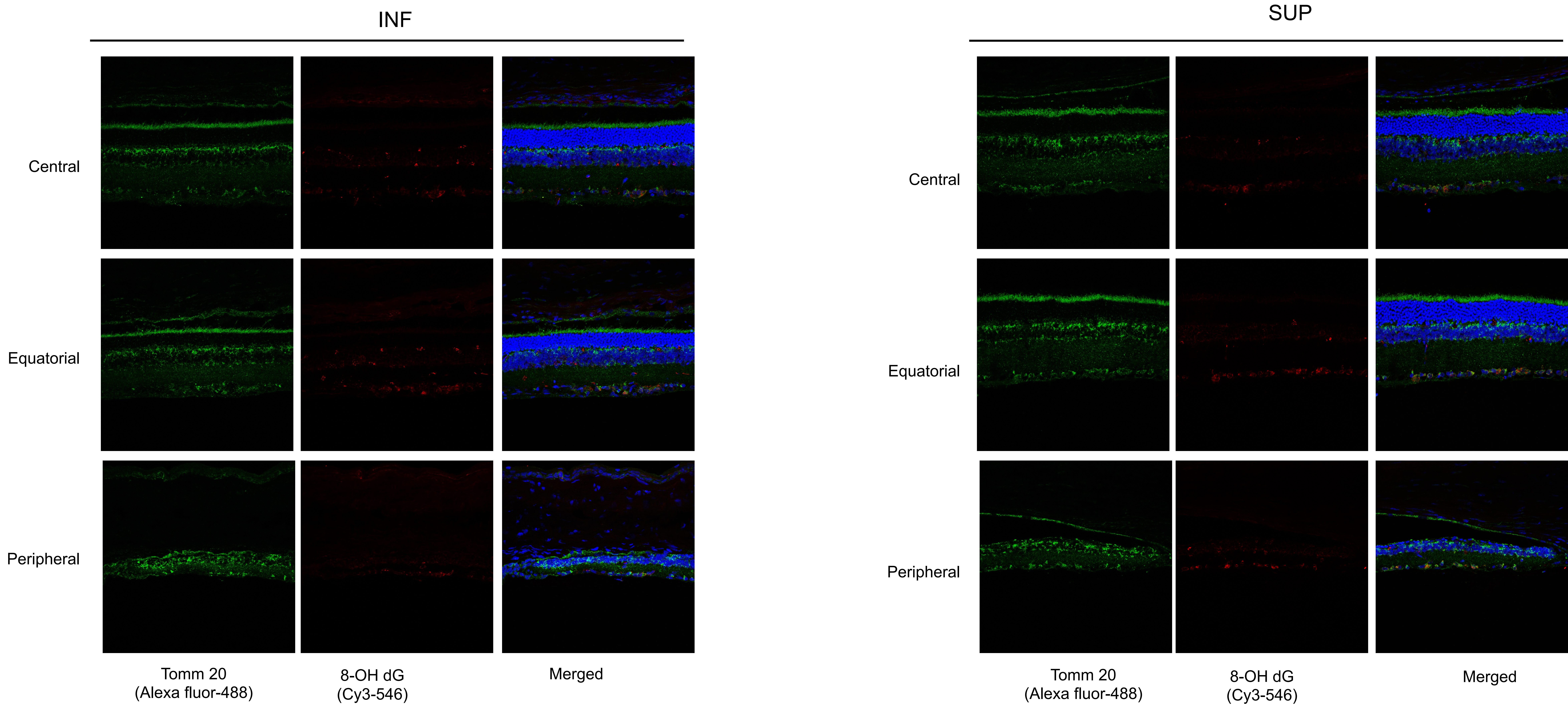

AGED water-treated RAT 5_8-OH dG

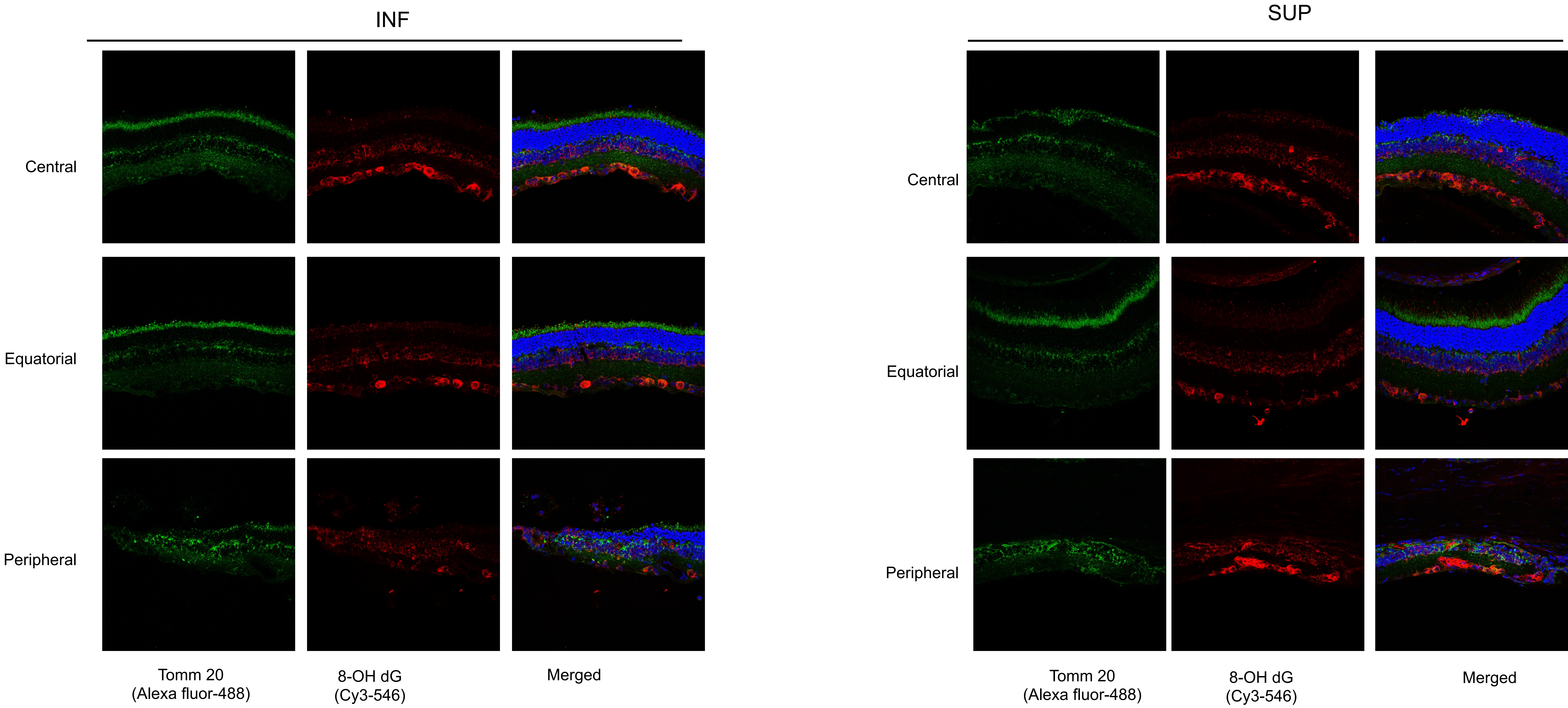

AGED water-treated RAT 6_8-OH dG

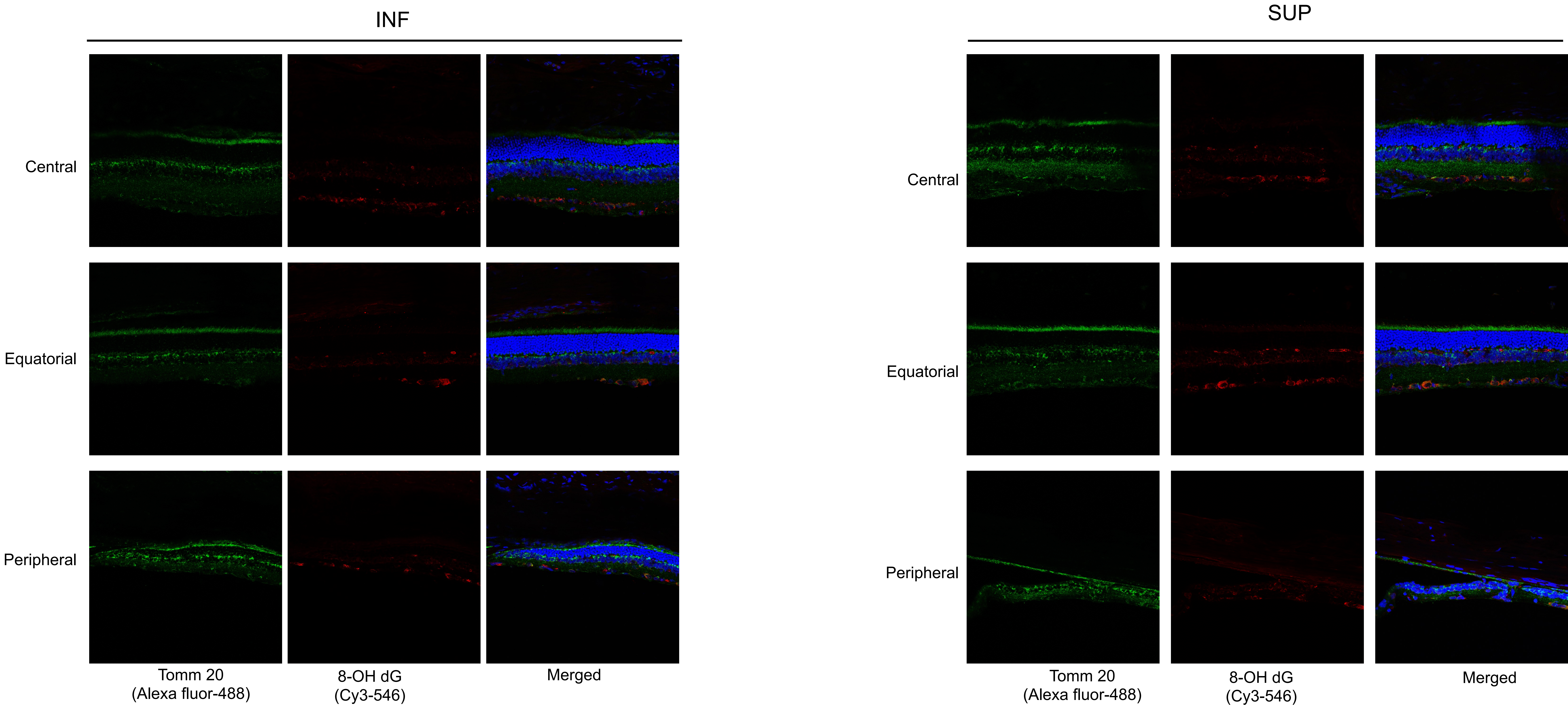

AGED water-treated RAT 7_8-OH dG

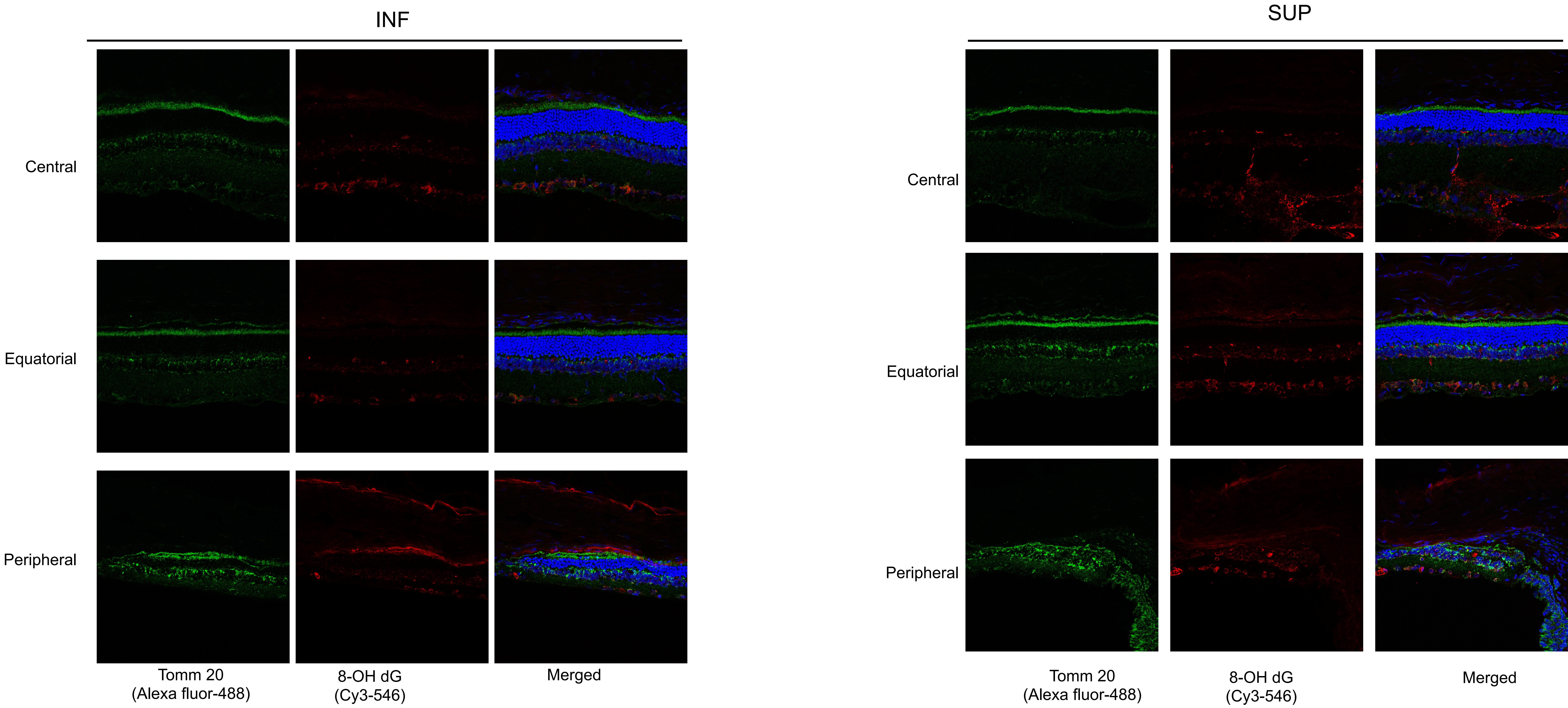

AGED water-treated RAT 10_8-OH dG

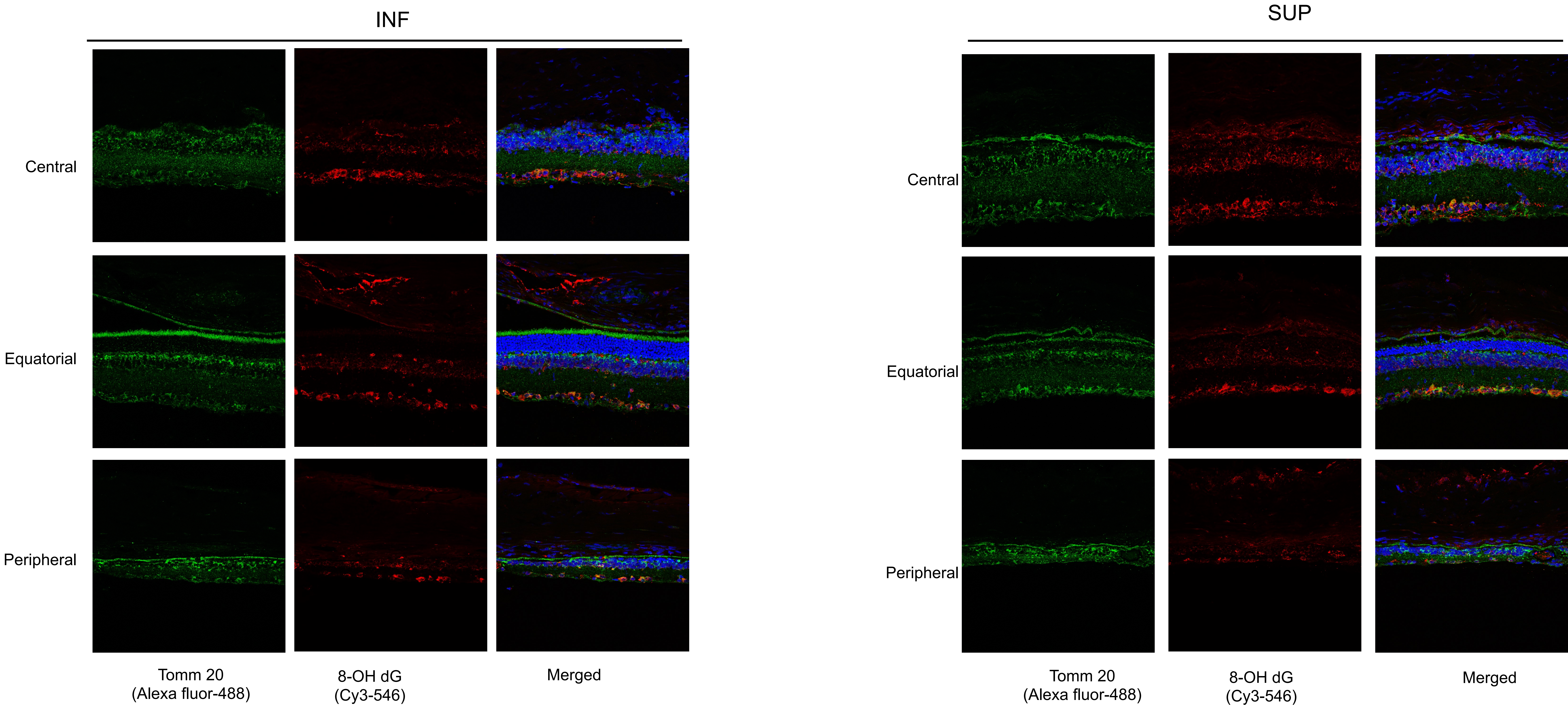

AGED water-treated RAT 11_8-OH dG

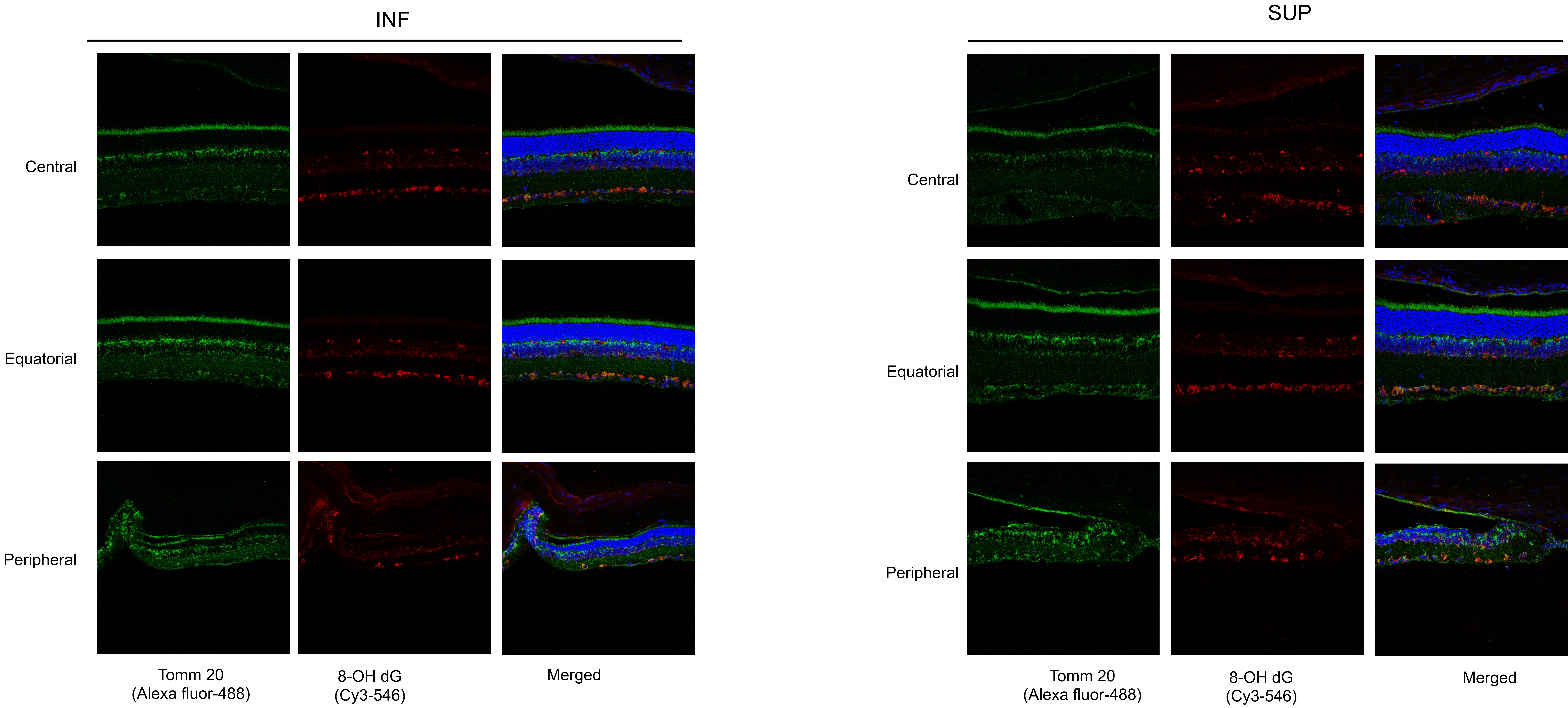

Young Rat 1_8-OH dG

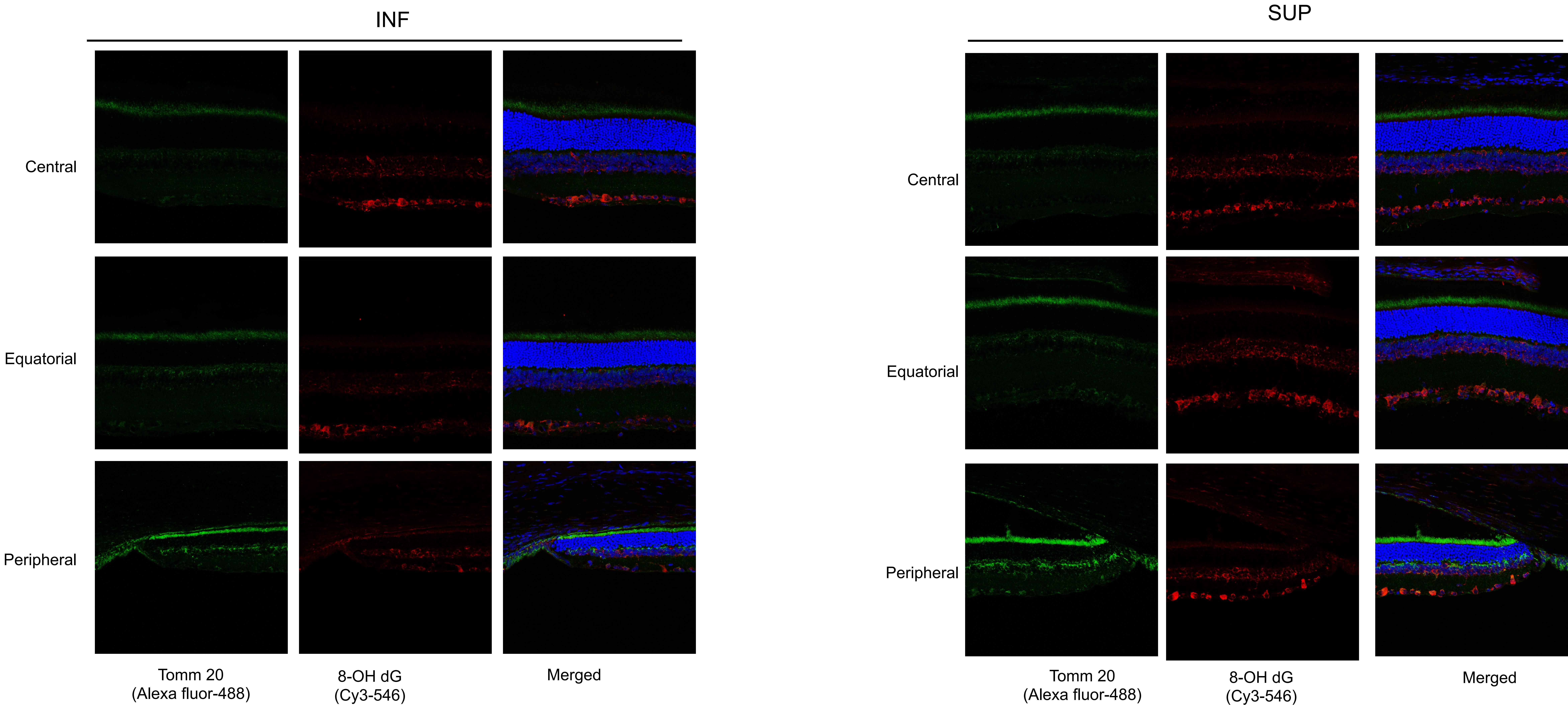

Young Rat 2_8-OH dG

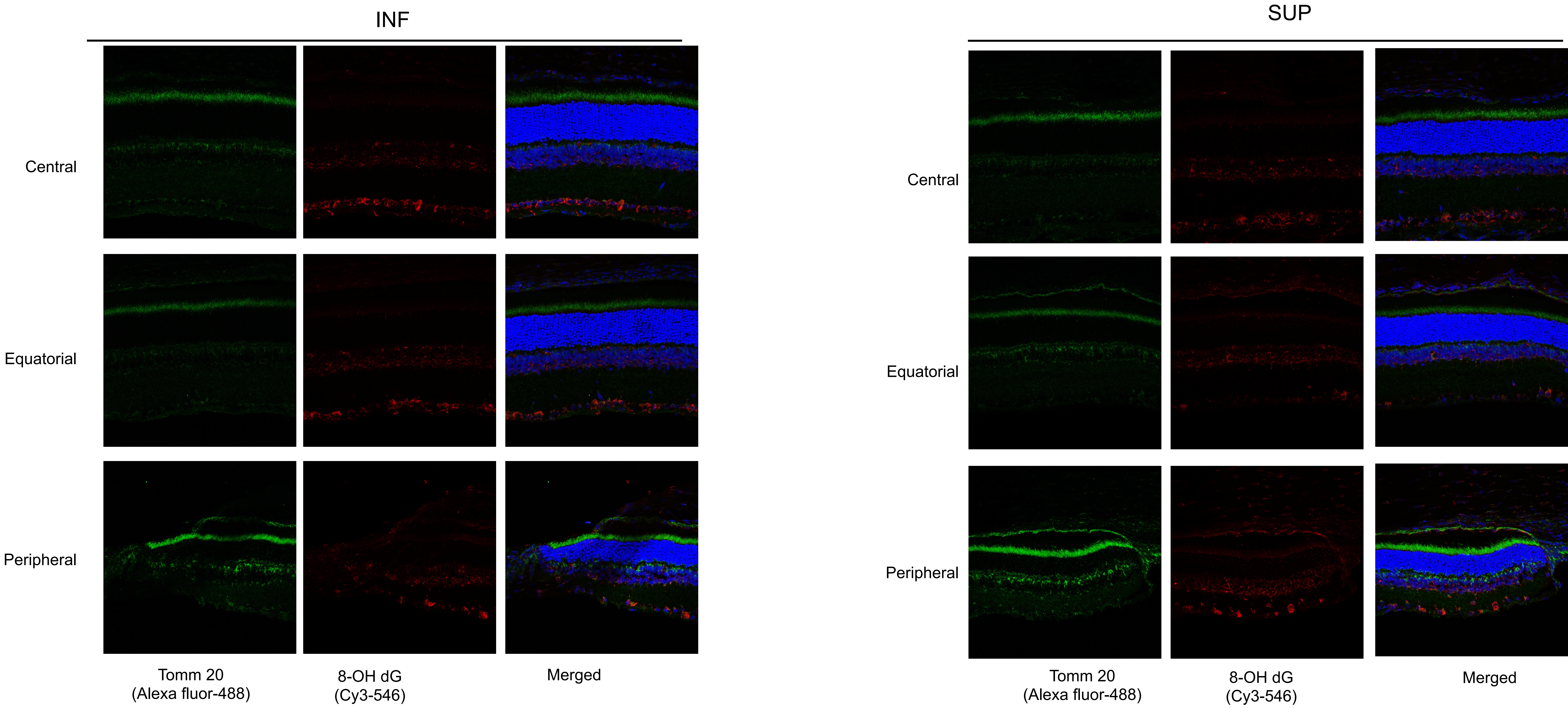

Young Rat 3_8-OH dG

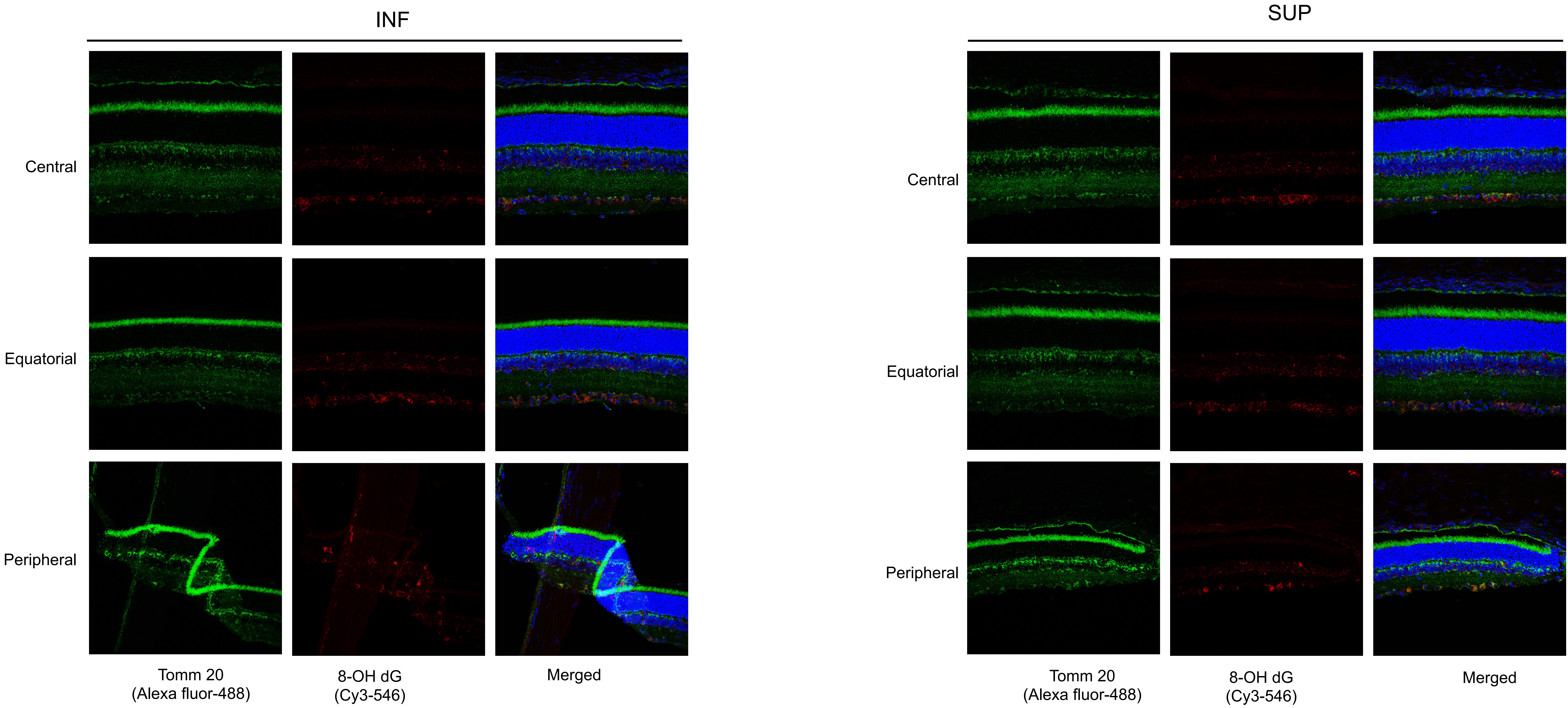

Young Rat 4_8-OH dG

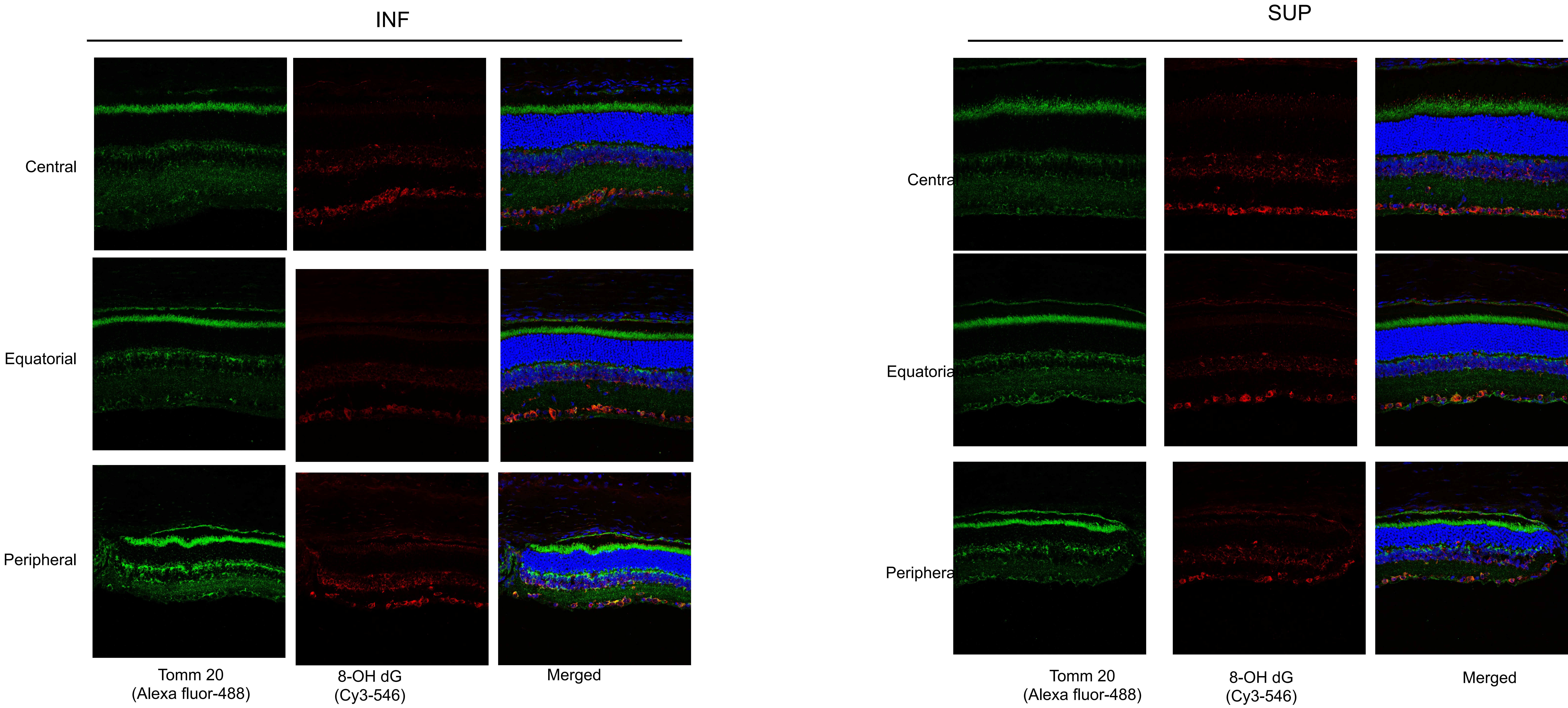

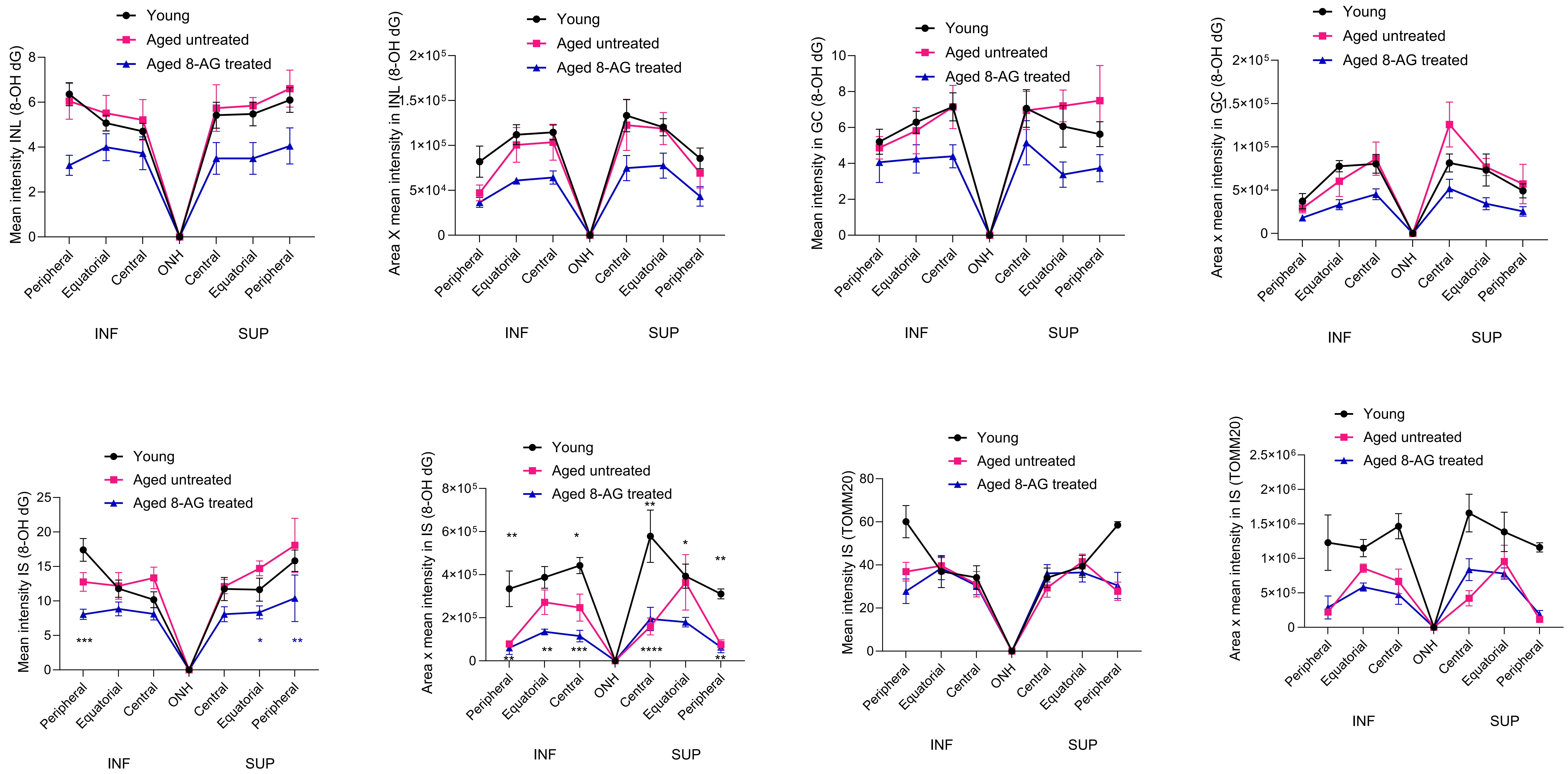

Young
Aged untreated
Aged 8-AG treated
Mean intensity INL (8-OH dG)
Area X mean intensity in INL (8-OH dG)
Mean intensity in GC (8-OH dG)
Area x mean intensity in GC (8-OH dG)
Mean intensity IS (8-OH dG)
Area x mean intensity in IS (8-OH dG)
Mean intensity IS (TOMM20)
Area x mean intensity in IS (TOMM20)
Peripheral
Equatorial
Central
ONH
Central
Equatorial
Peripheral
INF
SUP
