# Supplementary material for: Oral 8-aminoguanine against age-related retinal degeneration
Source: Commun Biol. 2025 May 26;8:812. doi: 10.1038/s42003-025-08242-1 (PMC12106806; doi:10.1038/s42003-025-08242-1)

GFAP staining for Figure 3S-V

Secondary only control

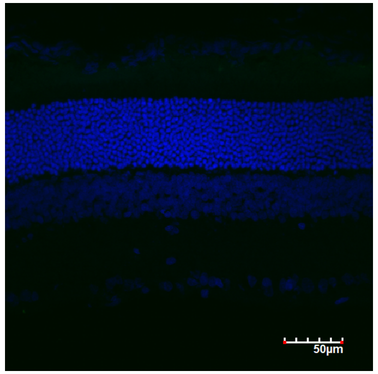

Young F344 rat retinae (Biol. repeat 1-2)

Green, GFAP; blue, Hoechst33342

Superior | Inferior

Peripheral | ONH | Peripheral

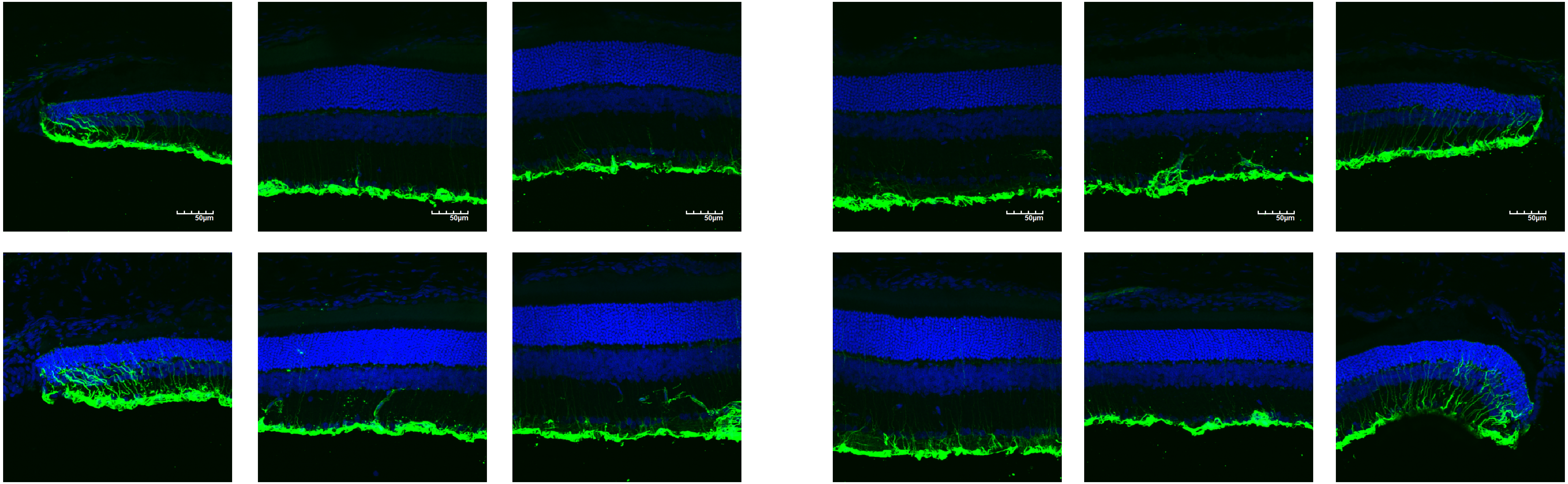

Young F344 rat retinae (Biol. repeat 3-4)

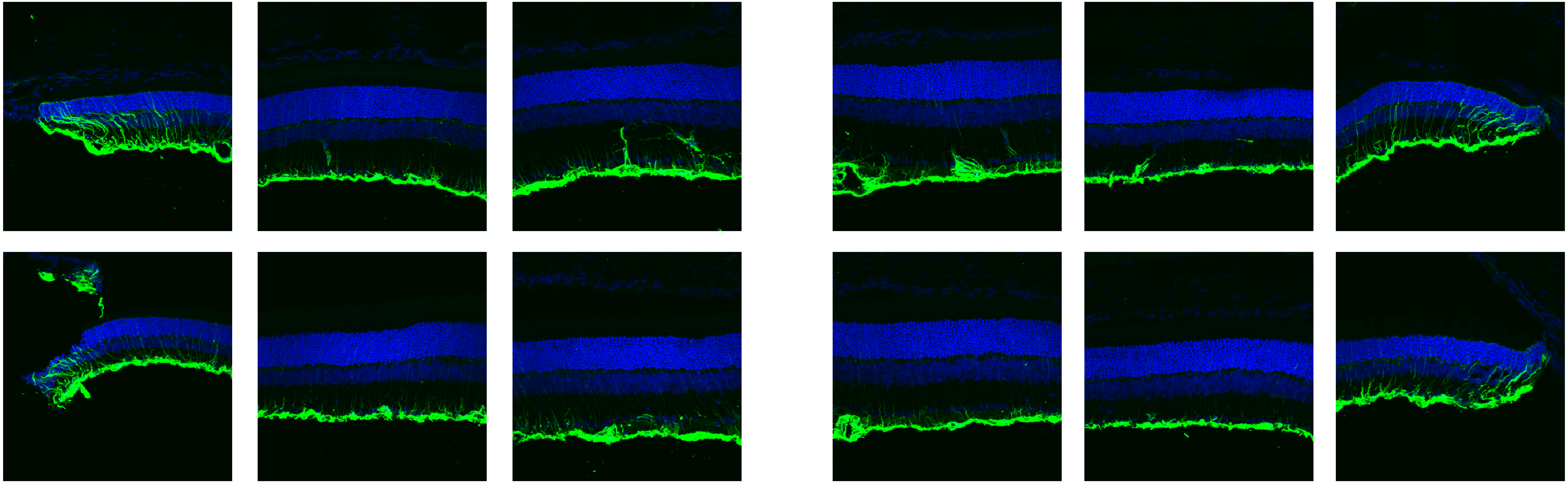

Aged water-treated F344 rat retinae (Biol. Repeat 1-2)

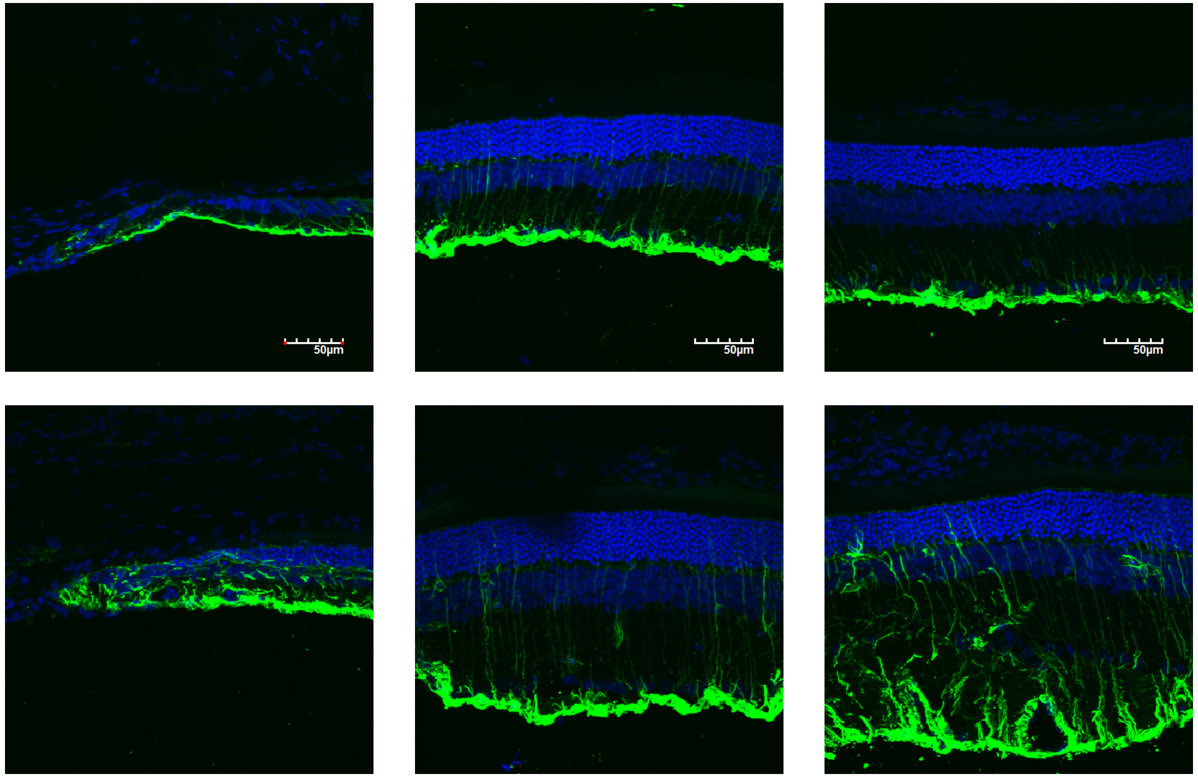

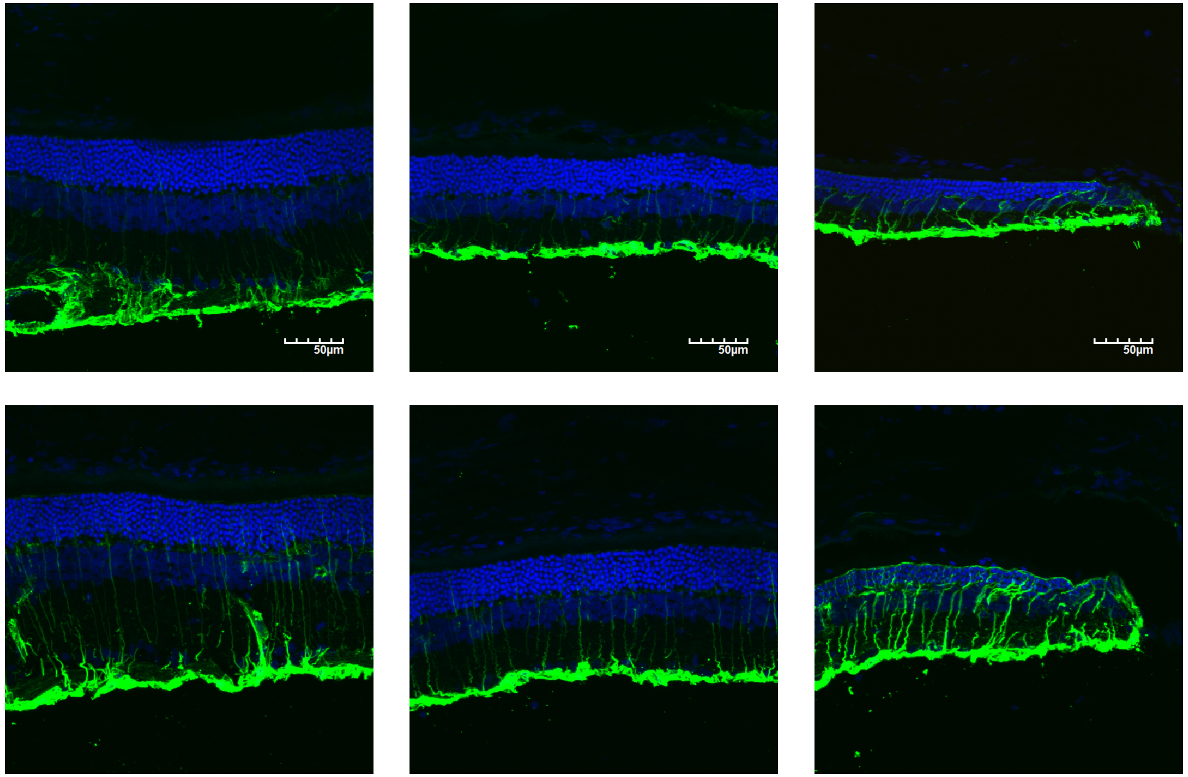

Aged water-treated F344 rat retinae (Biol. Repeat 3-4)

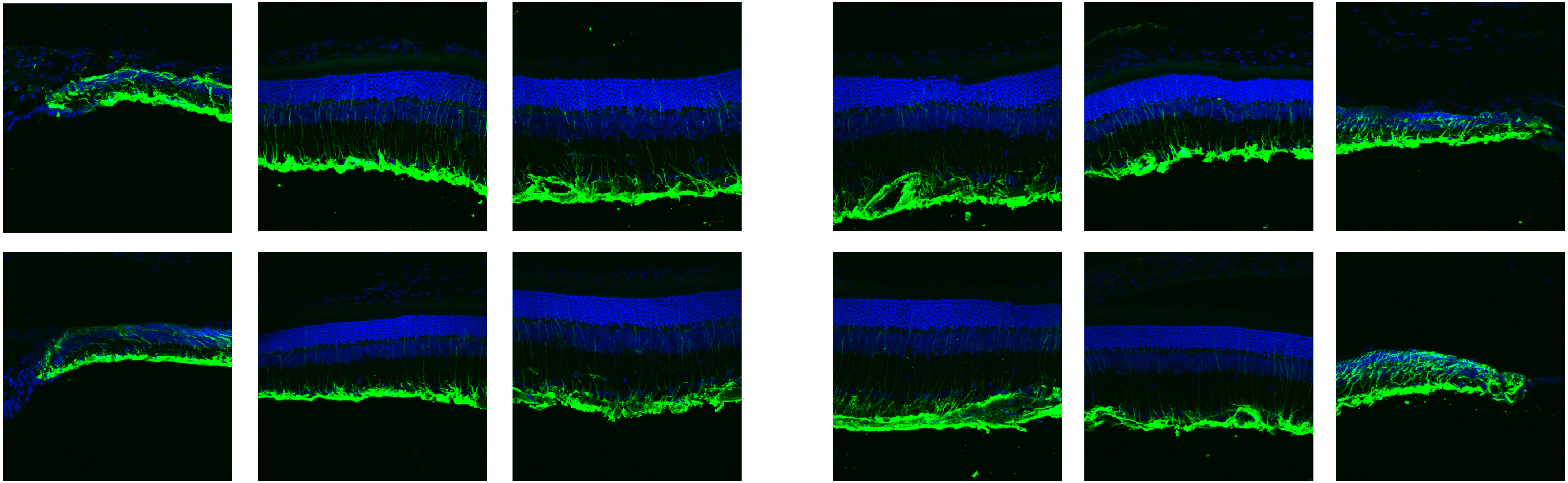

Aged 8AG-treated F344 rat retinae (Biol. Repeat 1-3)

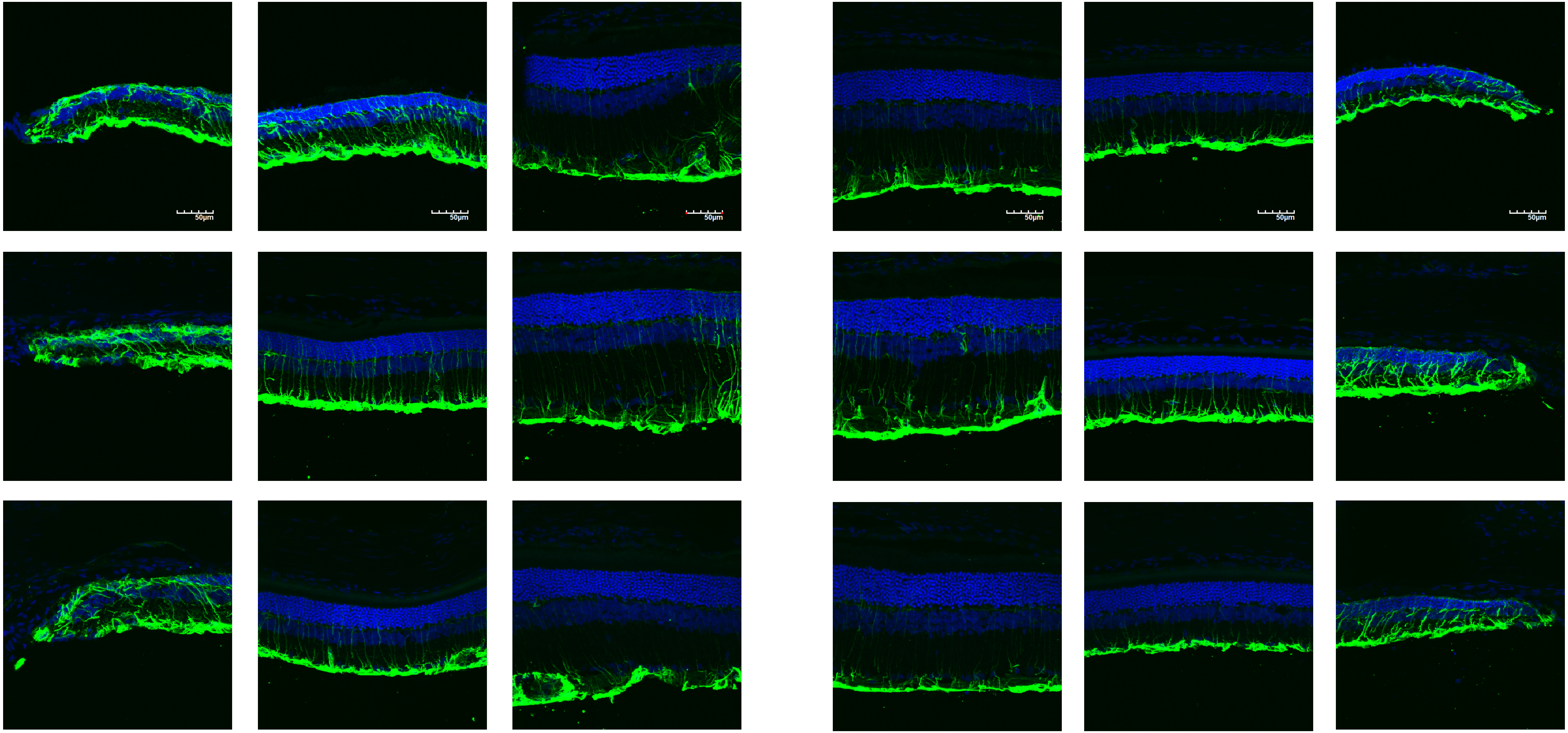

Supplement: Supplementary file 12 — Supplementary Data 10 [file 42003_2025_8242_MOESM12_ESM.pdf]
