# Supplementary material for: Oral 8-aminoguanine against age-related retinal degeneration
Source: Commun Biol. 2025 May 26;8:812. doi: 10.1038/s42003-025-08242-1 (PMC12106806; doi:10.1038/s42003-025-08242-1)

IBA1 and CD68 staining for Figure 3W-AB

Secondary Only

Red, IBA1

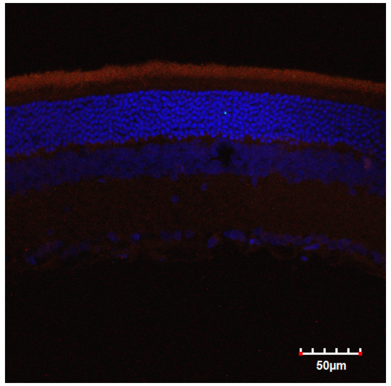

Young F344 rat retinae (Biol. repeat 1-2)

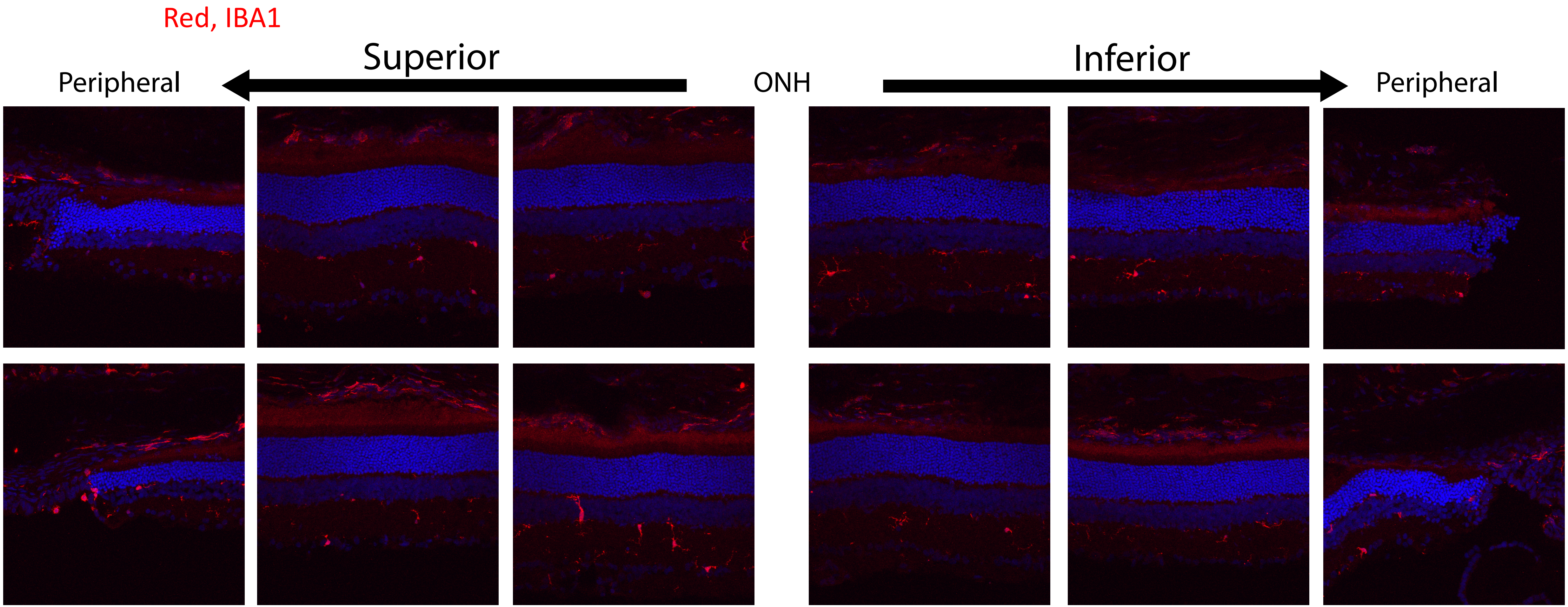

Young F344 rat retinae (Biol. repeat 3-4)

Red, IBA1

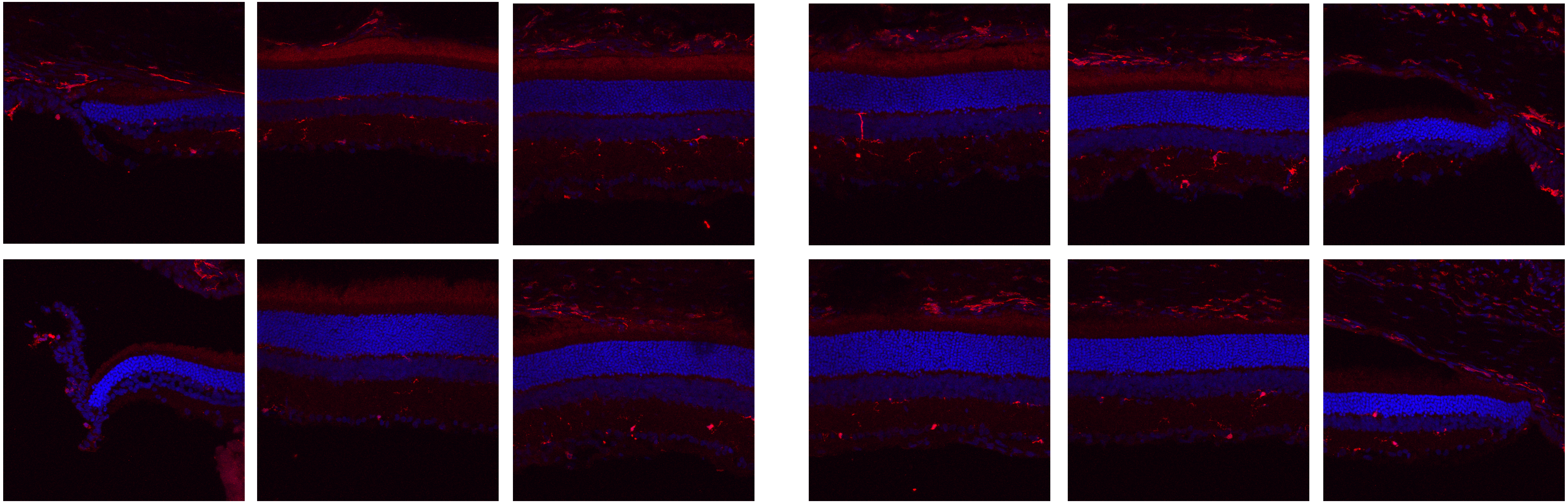

Aged water-treated F344 rat retinae (Biol. Repeat 1-2)

Red, IBA1

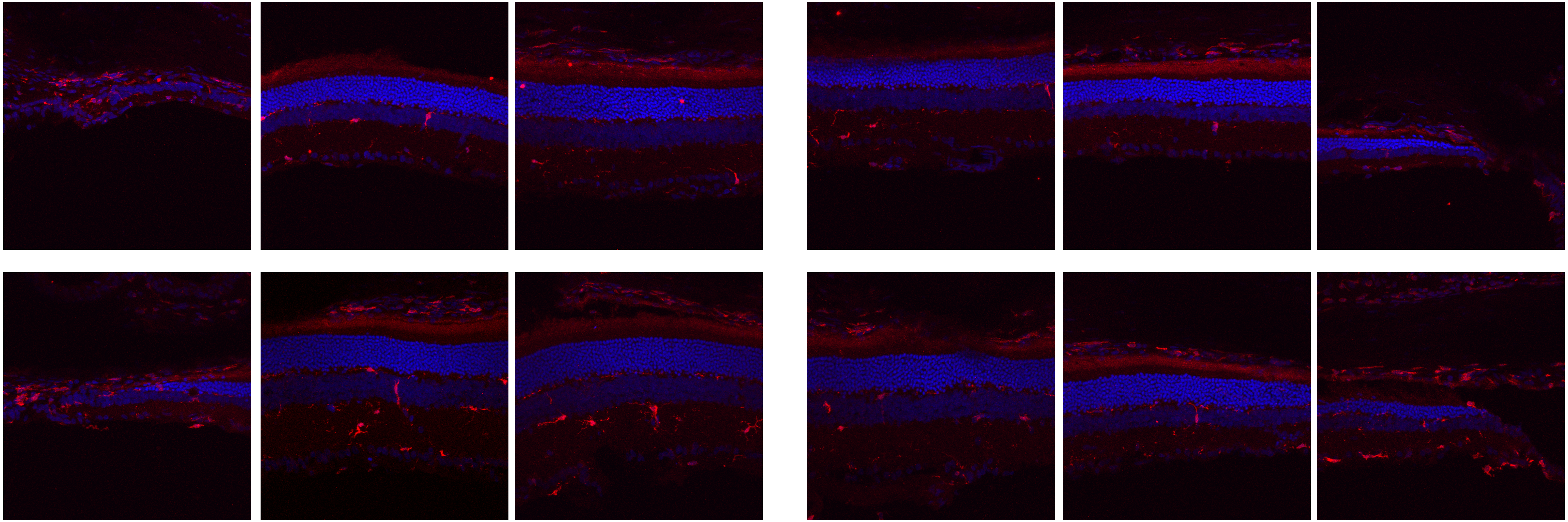

Aged water-treated F344 rat retinae (Biol. Repeat 3-4)

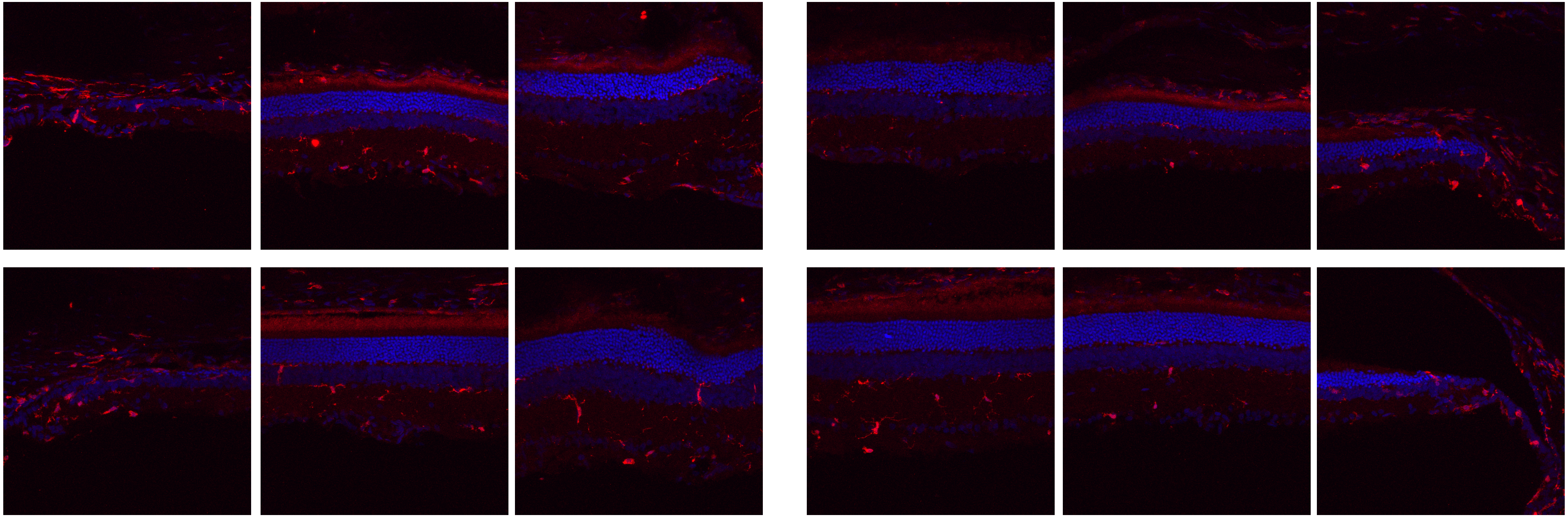

Aged 8AG-treated F344 rat retinae (Biol. Repeat 1-3)

Red, IBA1

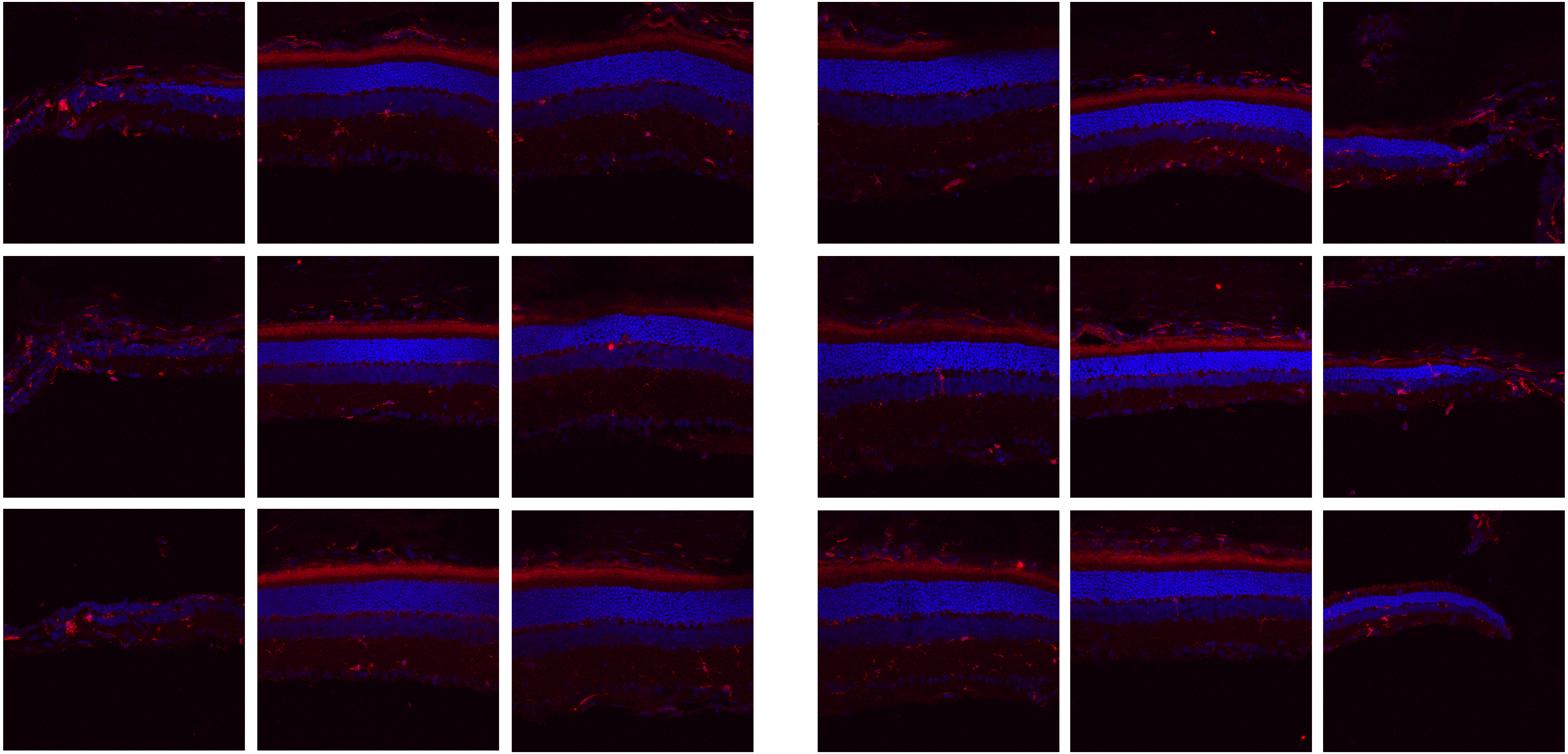

Secondary Only

Green, CD68

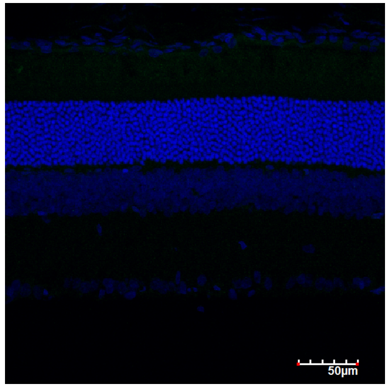

Young F344 rat retinae (Biol. repeat 1-2)

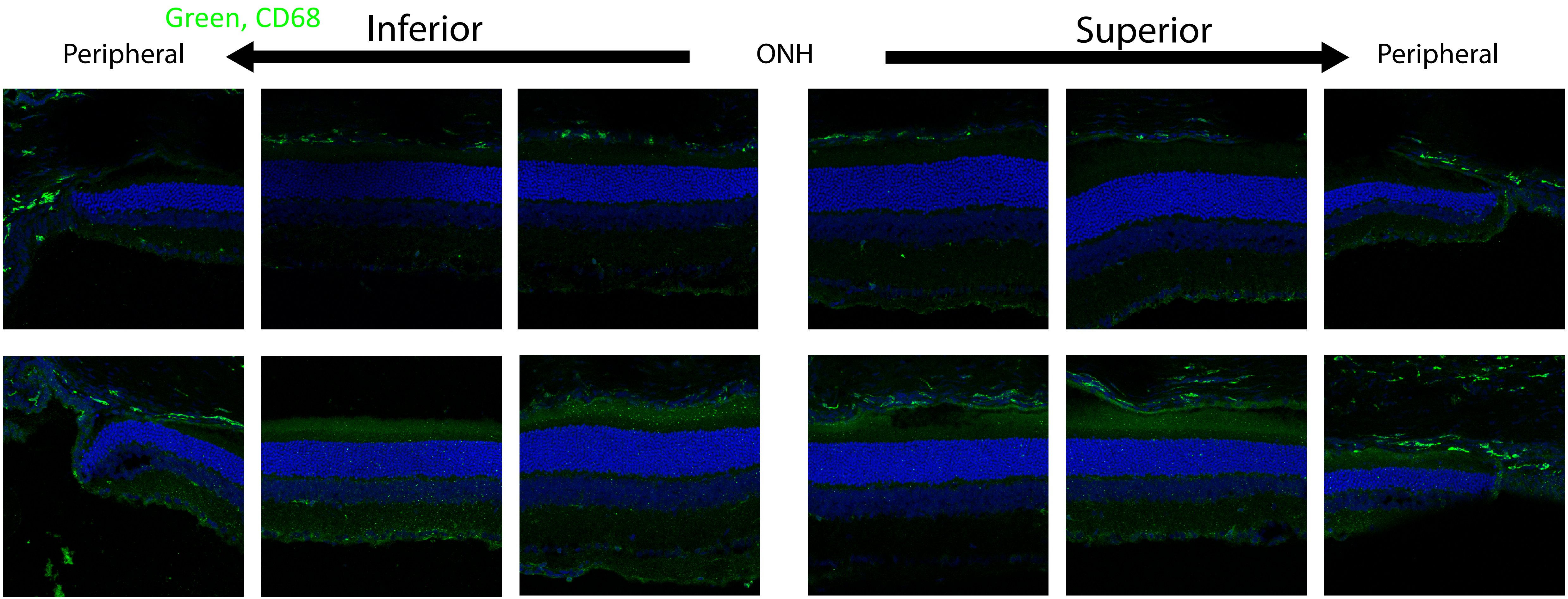

Young F344 rat retinae (Biol. repeat 3-4)

Green, CD68

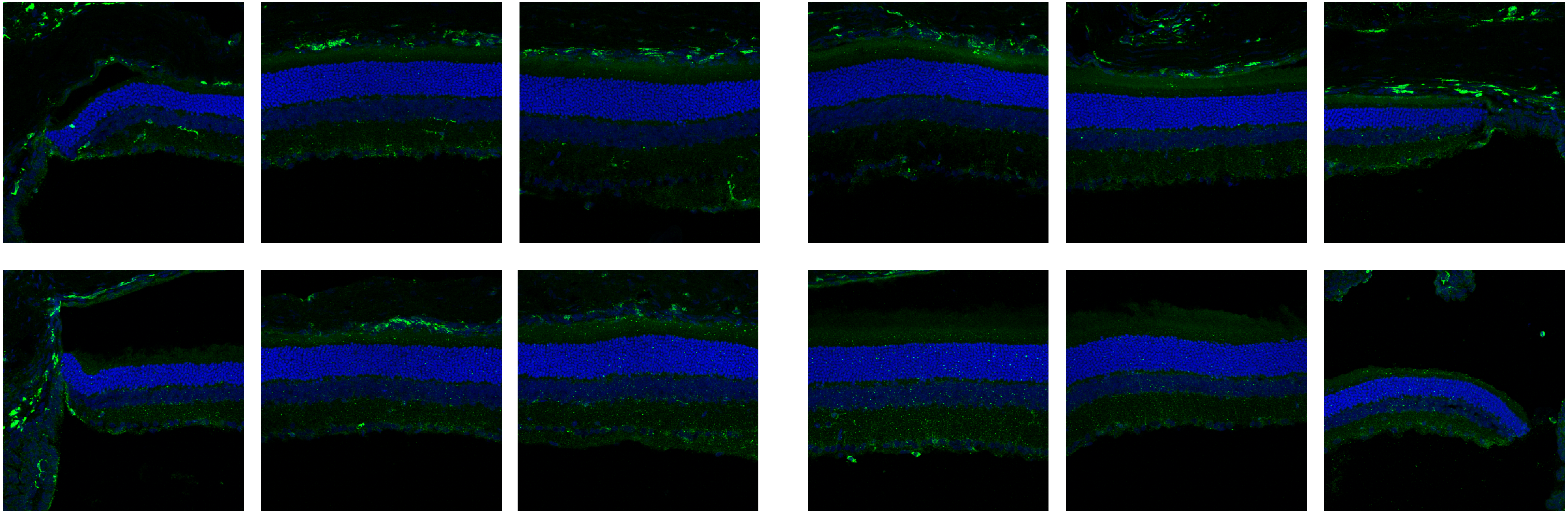

Aged water-treated F344 rat retinae (Biol. Repeat 1-2)

Green, CD68

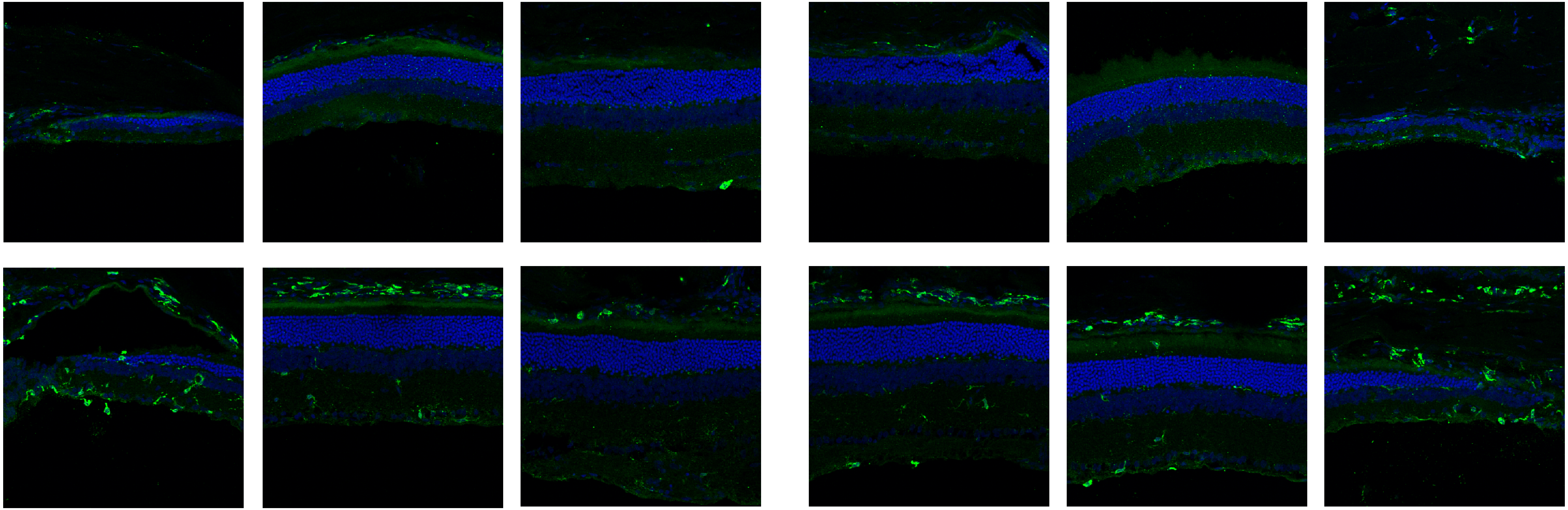

Aged water-treated F344 rat retinae (Biol. Repeat 3-4)

Green, CD68

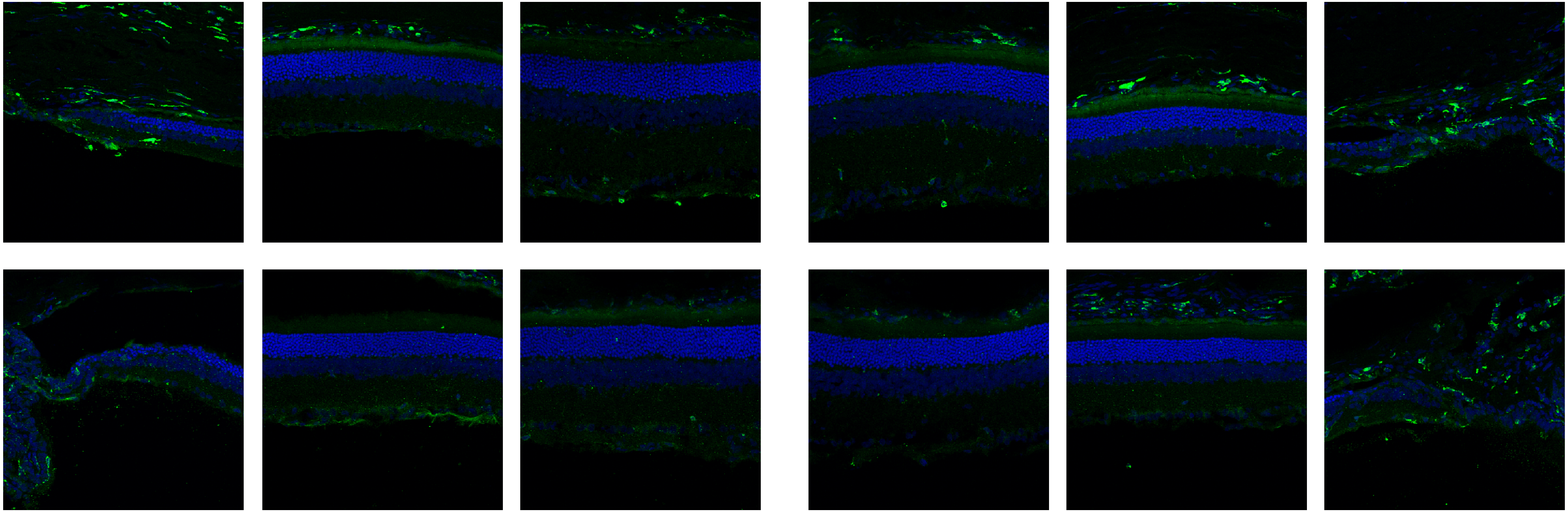

Aged 8AG-treated F344 rat retinae (Biol. Repeat 1-3)

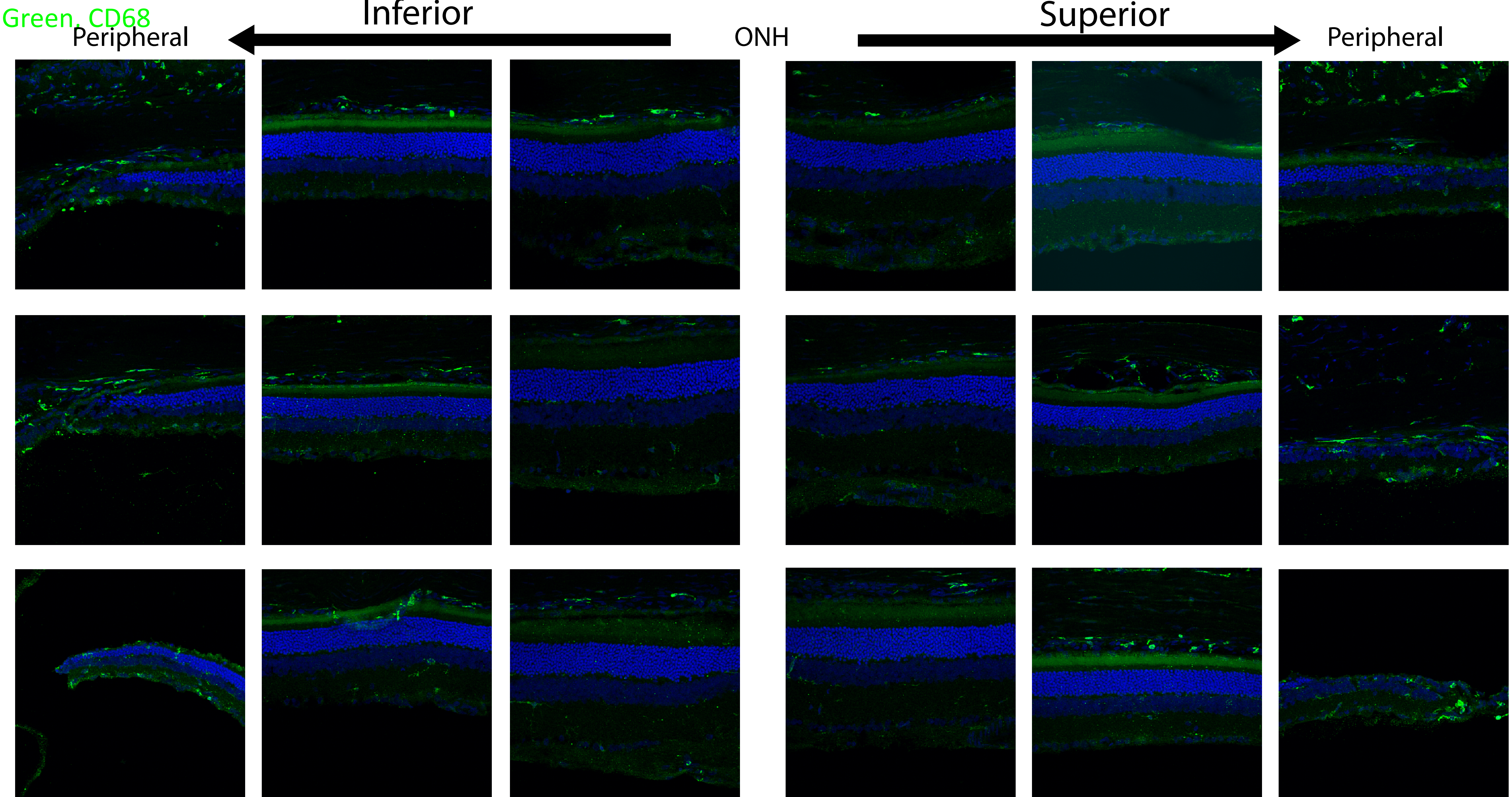

Supplement: Supplementary file 13 — Supplementary Data 11 [file 42003_2025_8242_MOESM13_ESM.pdf]
