# Supplementary material for: Oral 8-aminoguanine against age-related retinal degeneration
Source: Commun Biol. 2025 May 26;8:812. doi: 10.1038/s42003-025-08242-1 (PMC12106806; doi:10.1038/s42003-025-08242-1)
Supplement: Supplementary file 19 — Supplementary Data 17 [file 42003_2025_8242_MOESM19_ESM.pdf]

IHC of RHO of 27 m rats for figure 8F-H

Untreated rats 27M_1L

INF

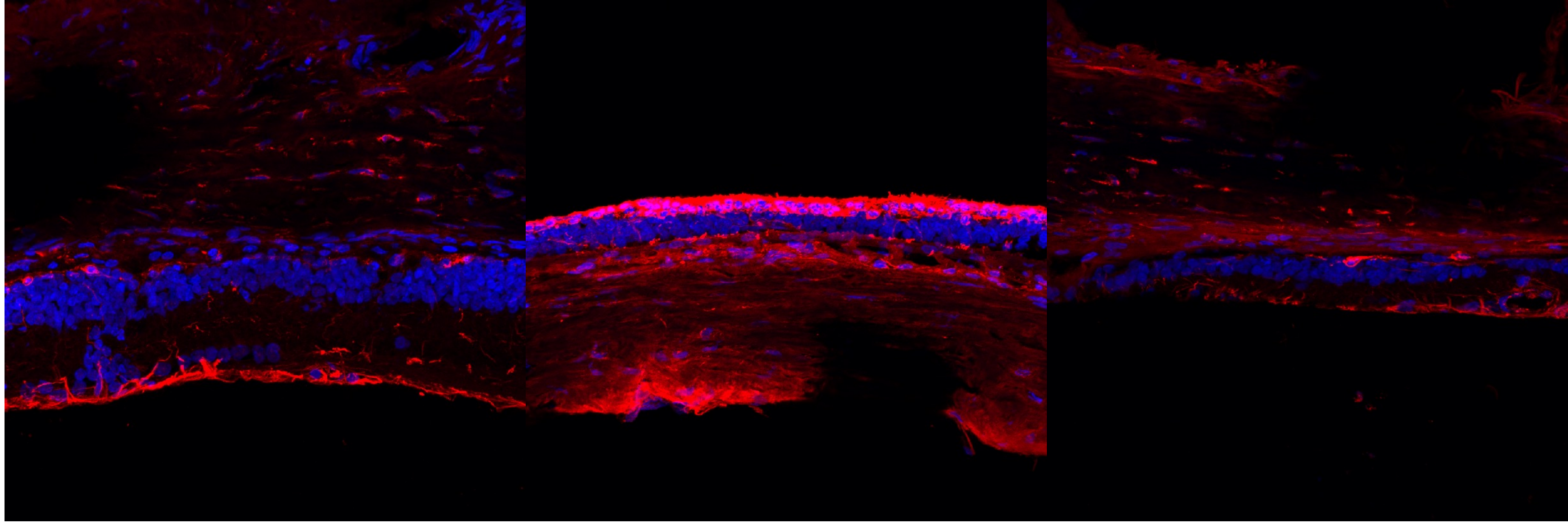

Central Equatorial Peripheral

SUP

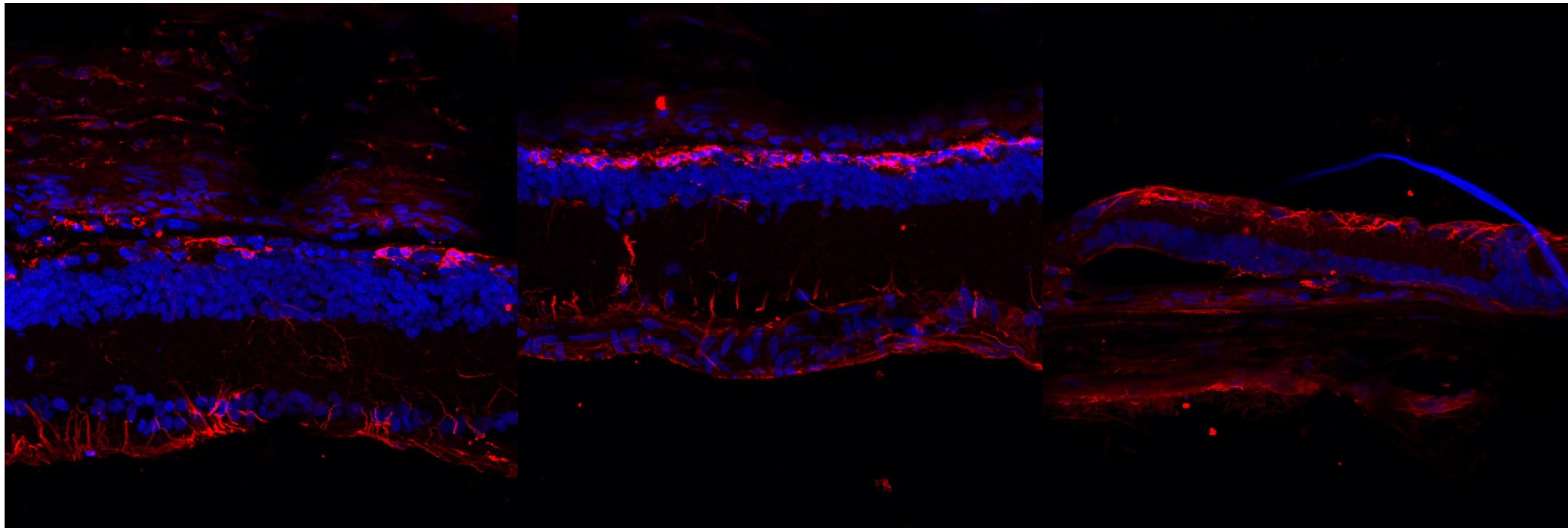

Untreated rats 27M_1R

INF

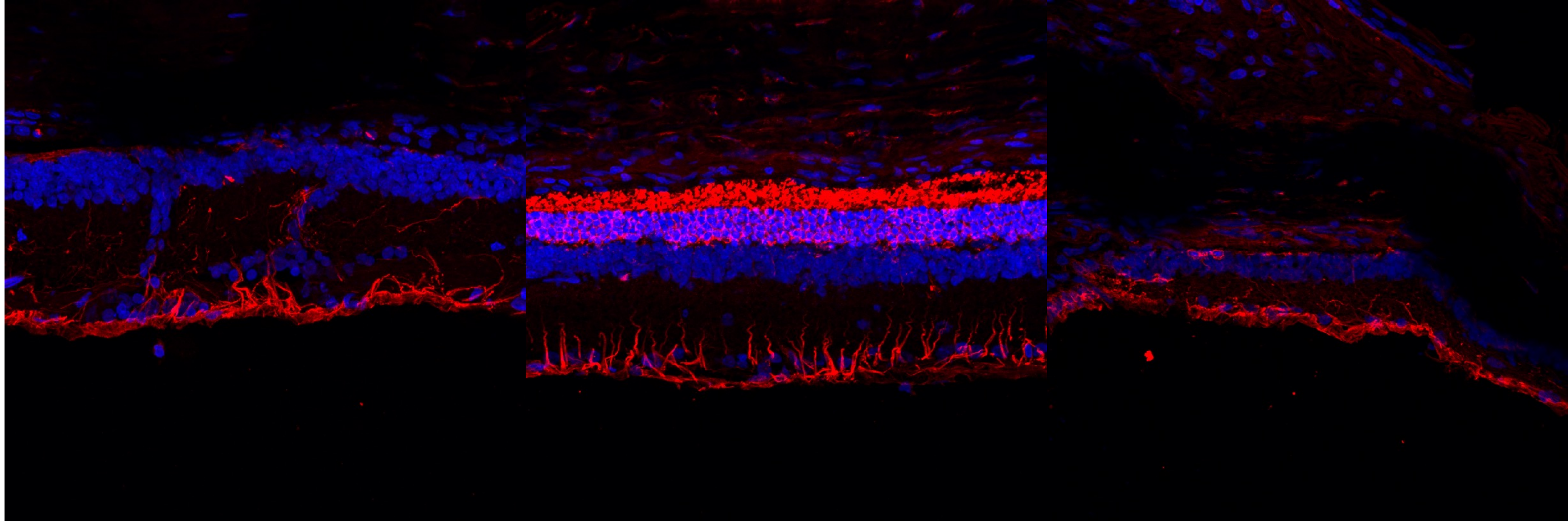

Central | Equatorial | Peripheral

SUP

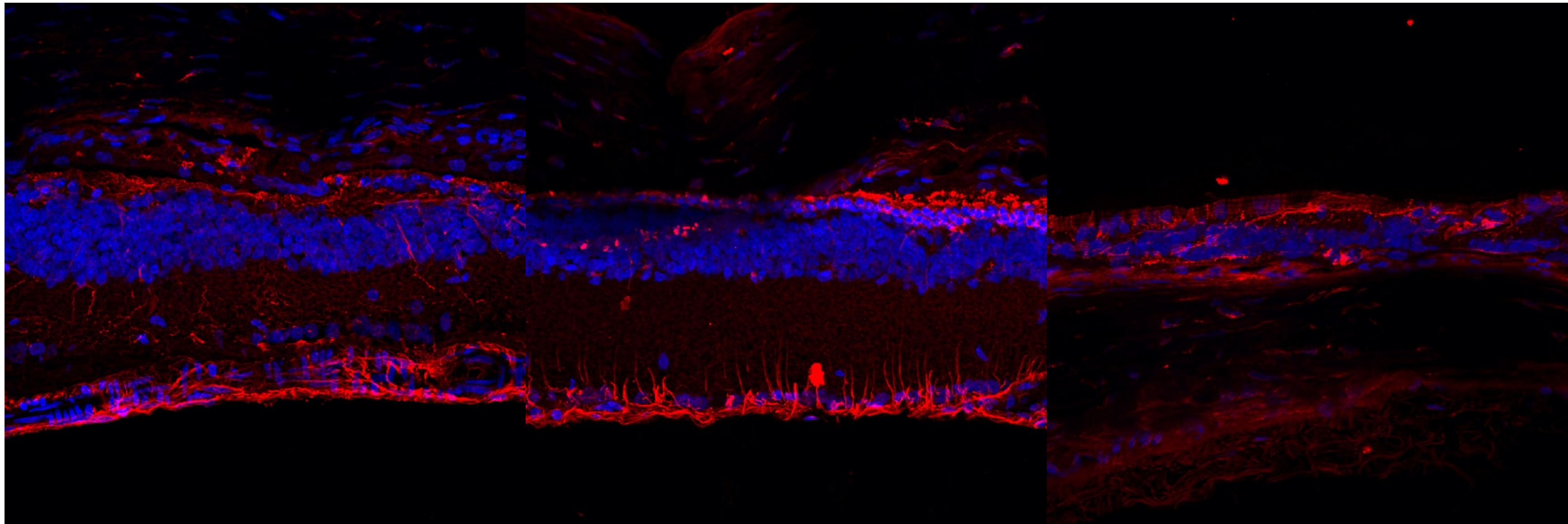

8-AG TREATED rats 27M_2L

INF

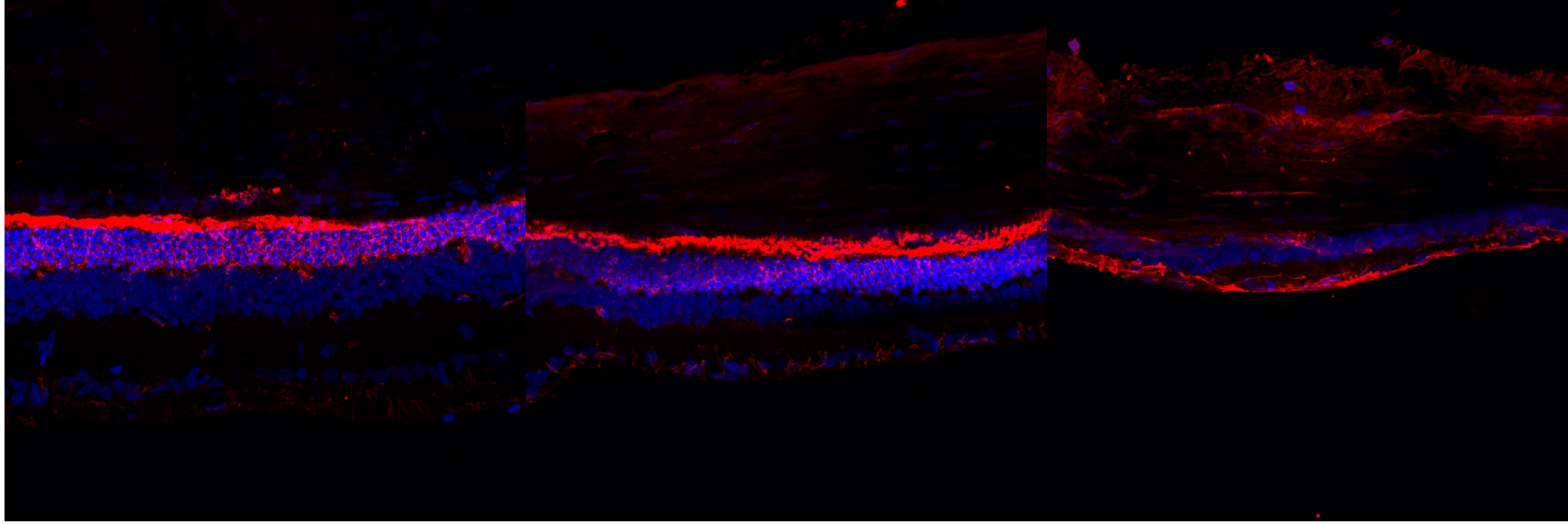

Central Equatorial Peripheral

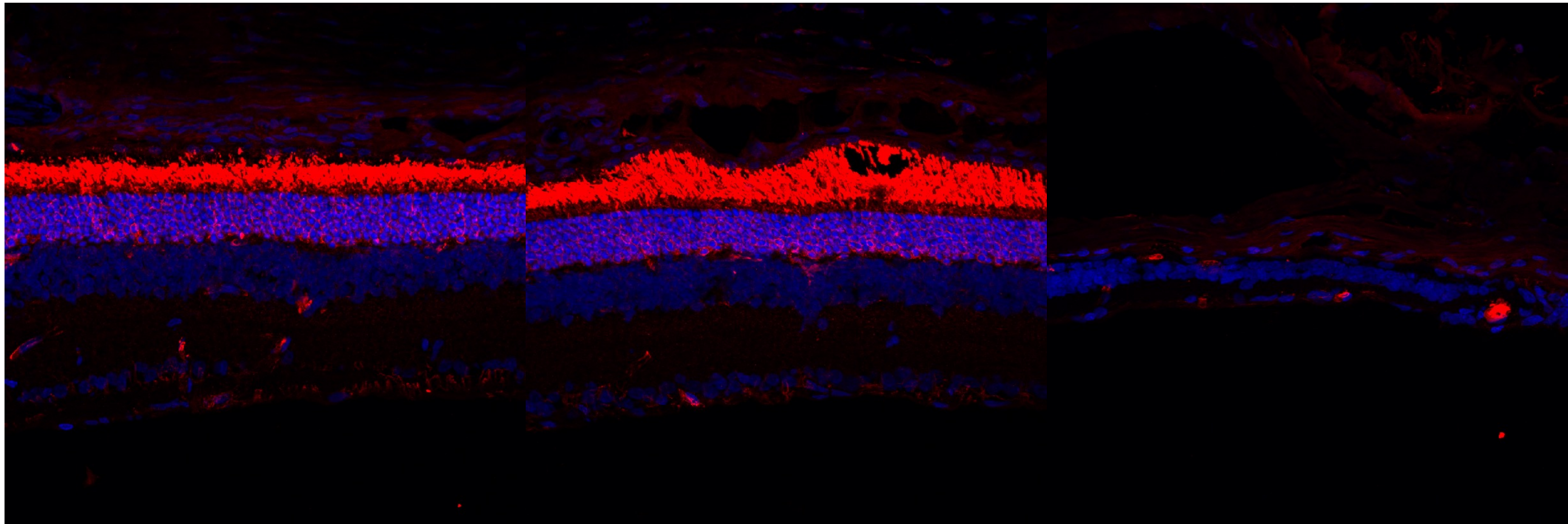

SUP

8-AG TREATED rats 27M_2R

INF

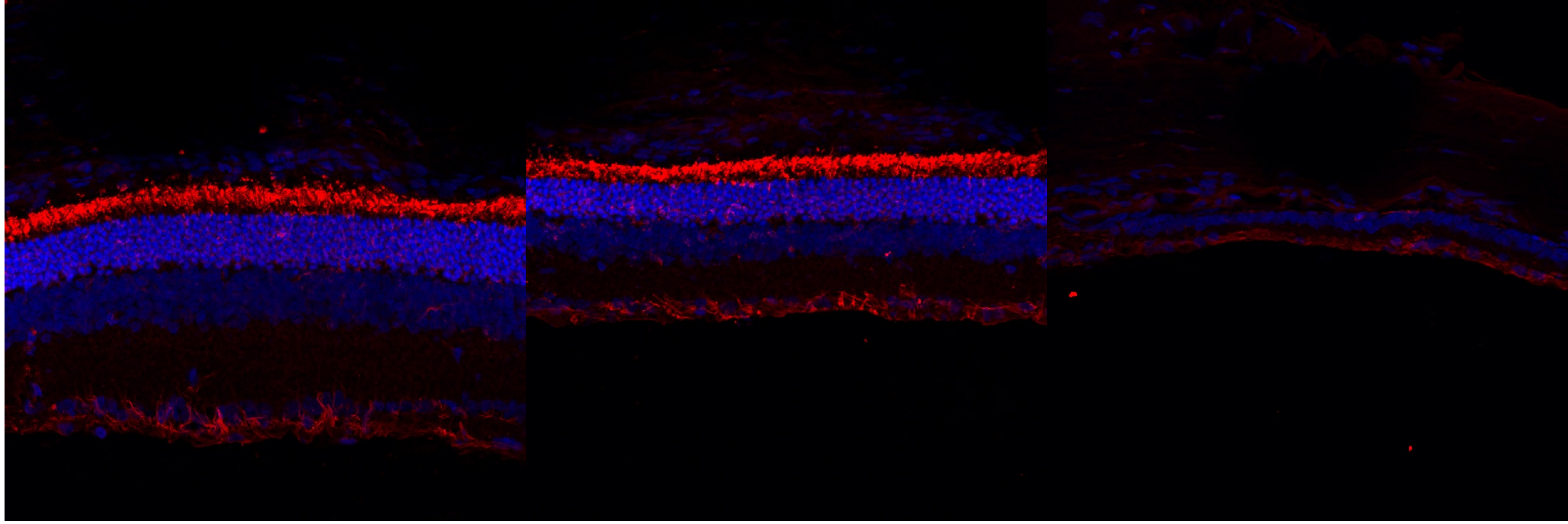

Central Equatorial Peripheral

SUP

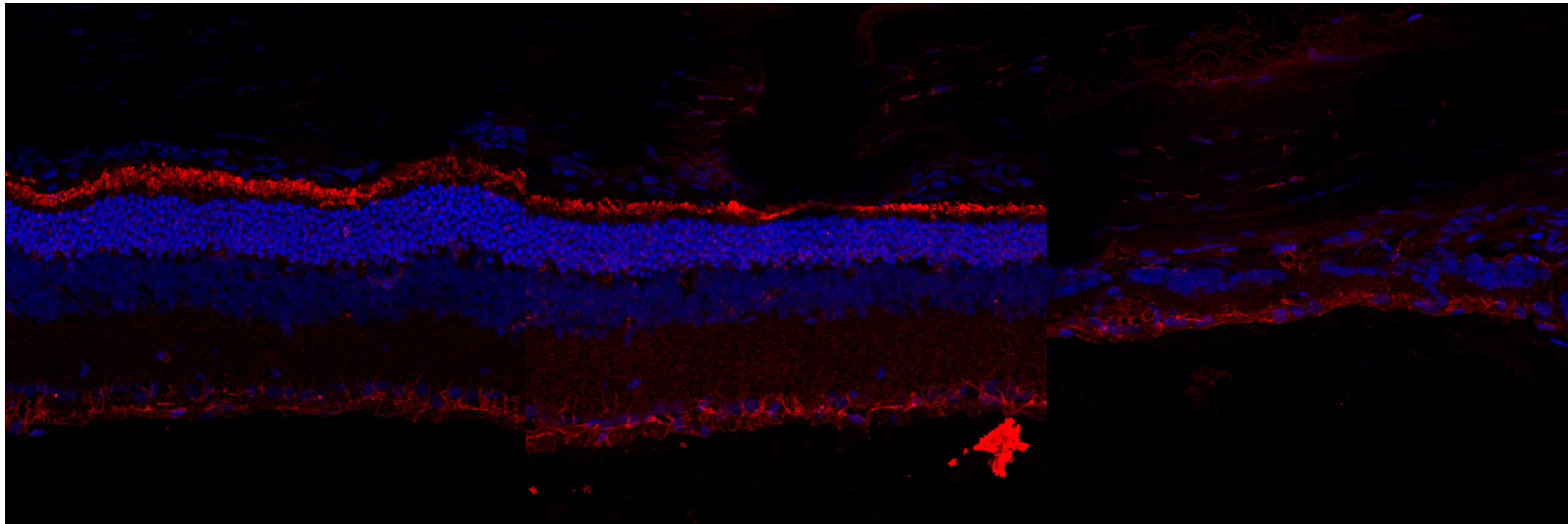

8-AG TREATED rats 27M_3L

INF

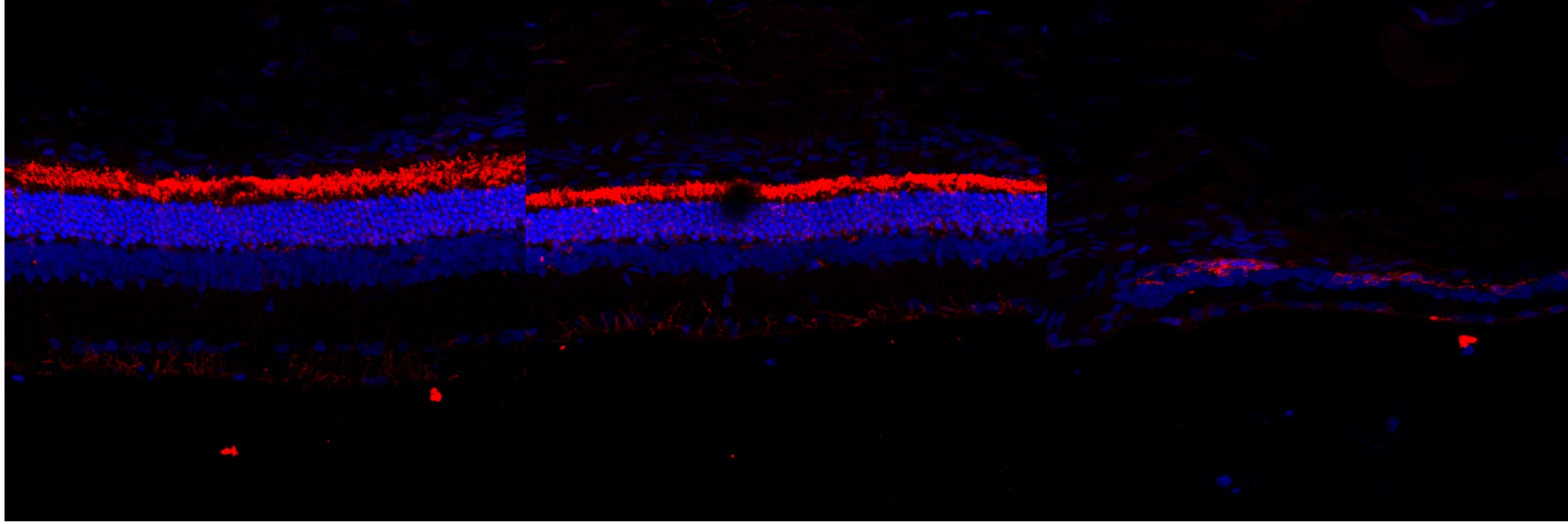

Central Equatorial Peripheral

SUP

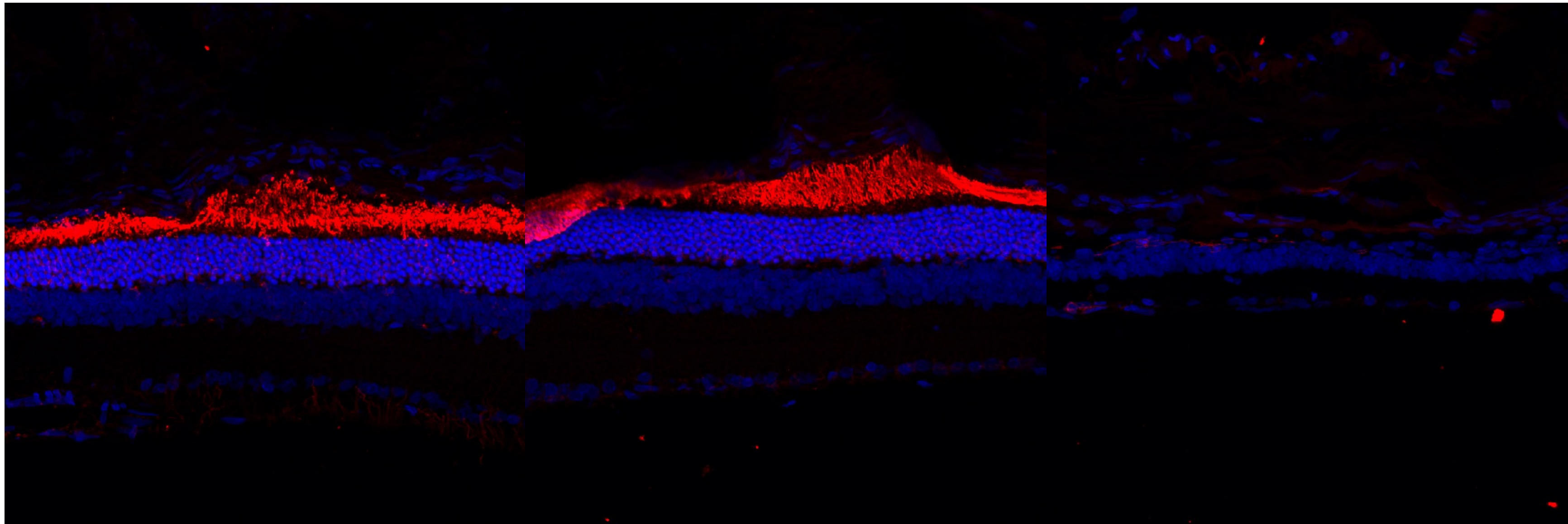

8-AG TREATED rats 27M_3R

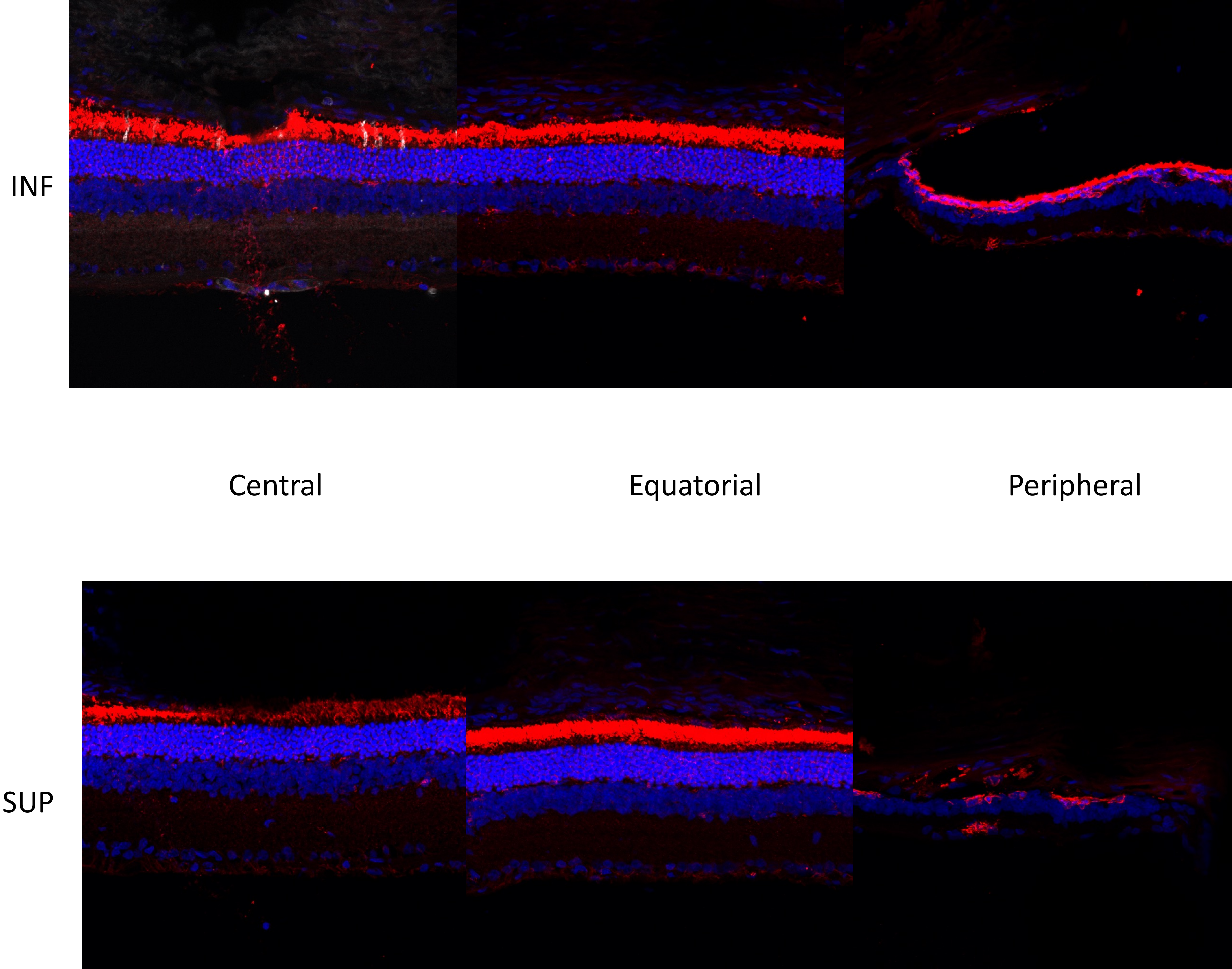

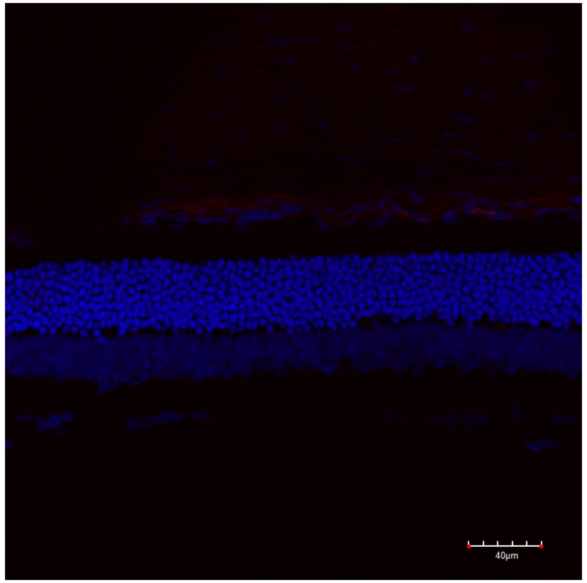
40μm
