# Supplementary material for: Oral 8-aminoguanine against age-related retinal degeneration
Source: Commun Biol. 2025 May 26;8:812. doi: 10.1038/s42003-025-08242-1 (PMC12106806; doi:10.1038/s42003-025-08242-1)

Trial1+Trial5 mouse HE-staining data for Figure S4E-H and N-Q

Trial-1 IP injection of 8AG in $Rho^{P23H/+}$ mice

- IP injection
  - 1.1 mg/ml slurry
  - pH 7.2
  - 11 mg/kg bw—10ul/g bw
- Test groups:
  - DOB 06/05/2020 (first injection: PND15)
- Injection—L0 for PBS; R0 for 8AG
  - 662187: 5 pups—11 mg/kg bw; 2XPBS, 3X8AG
  - 714762: 7 pups—11 mg/kg bw; 3XPBS, 4X8AG
  - 607740: 5 pups—5.5 mg/kg bw; 3XPBS, 2X8AG
- OCT date: PND33/49
- ERG date: PND36/50
- IHC/H&E date: PND53

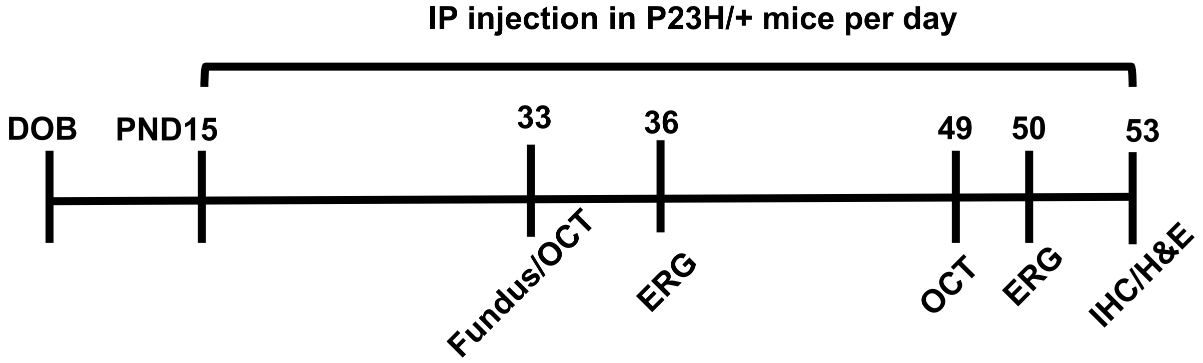

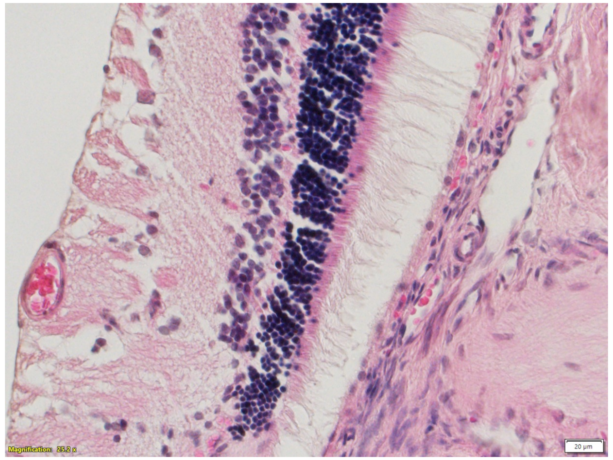

Trial1 mouse HE staining data PND53 20X—Rho$^{+/+}$

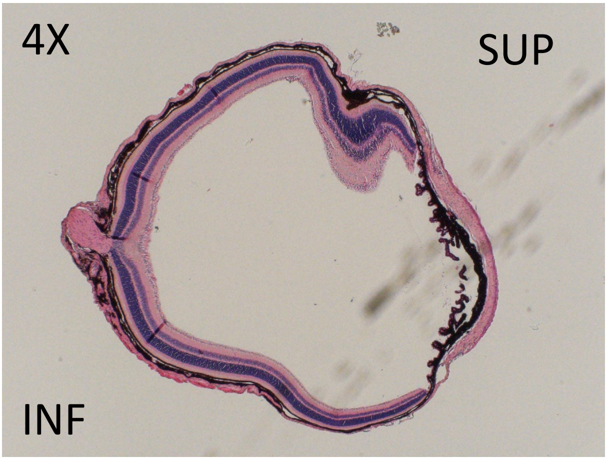

IC

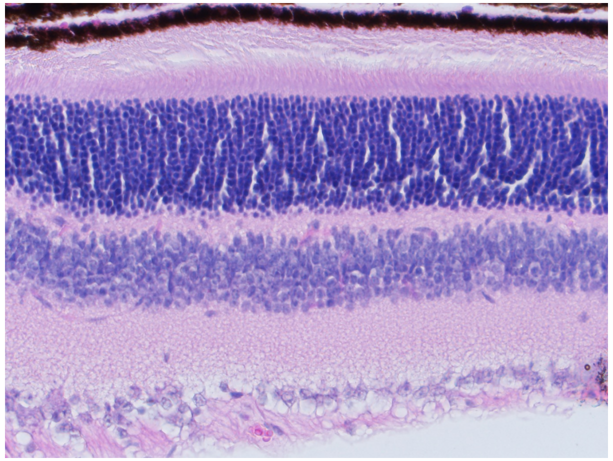

IE

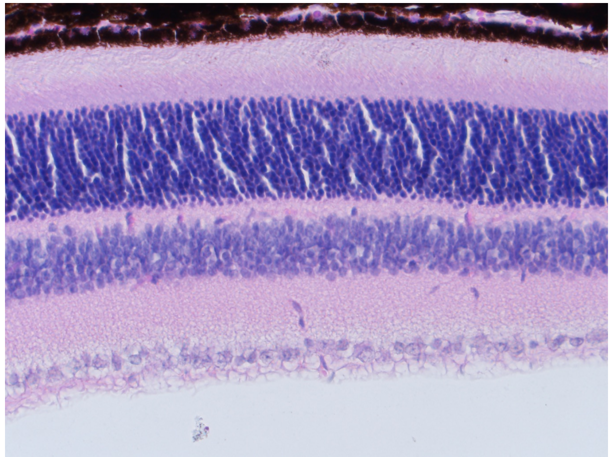

IP

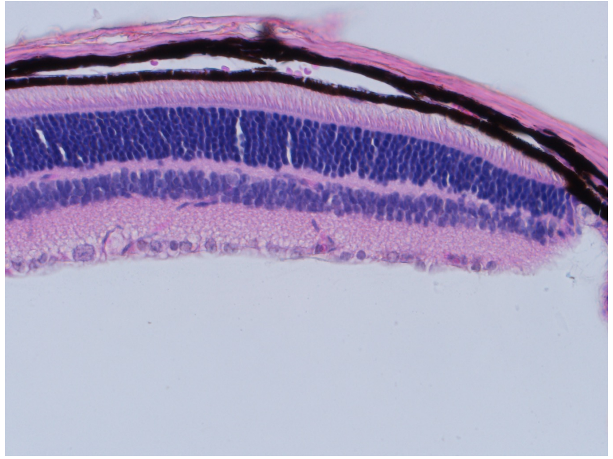

SP

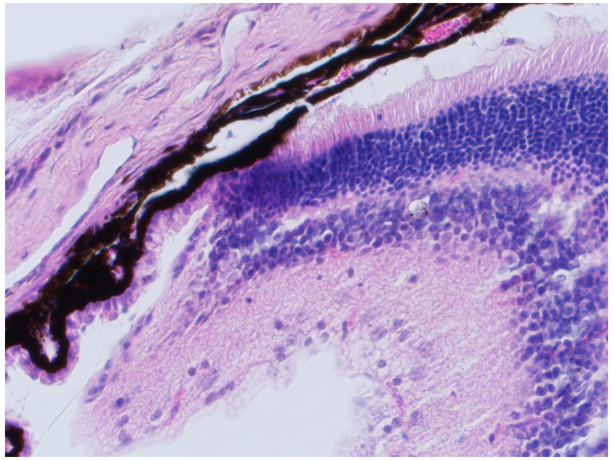

SE

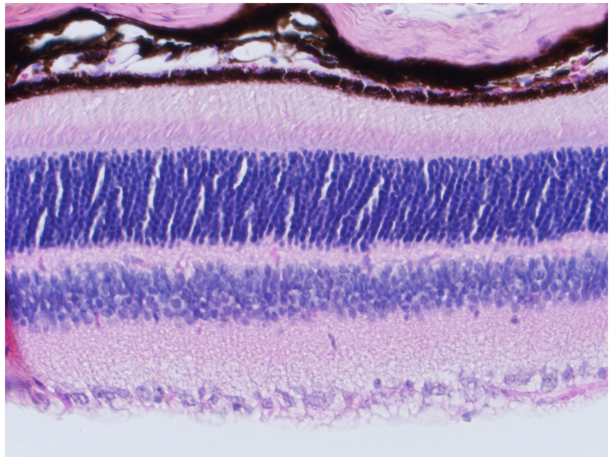

SC

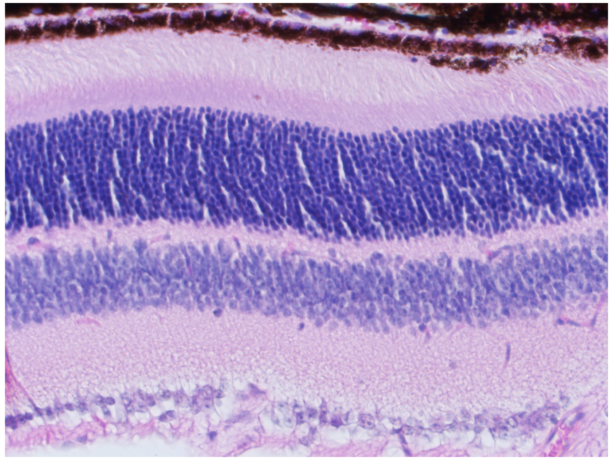

Trial1 mouse HE staining data PND53 20X—Rho$^{P23H/+}$ PBS-2

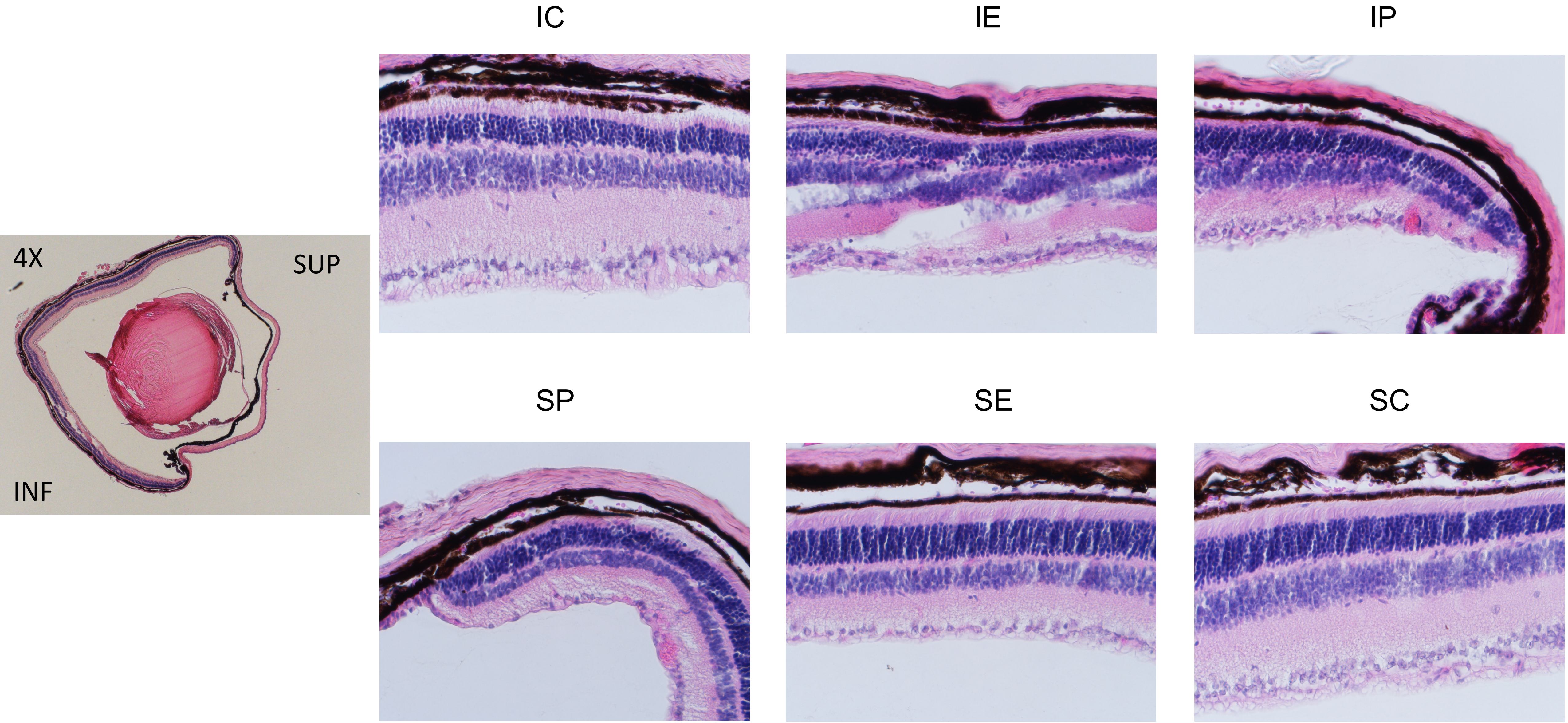

Trial1 mouse HE staining data PND53 20X—Rho$^{P23H/+}$ PBS-3

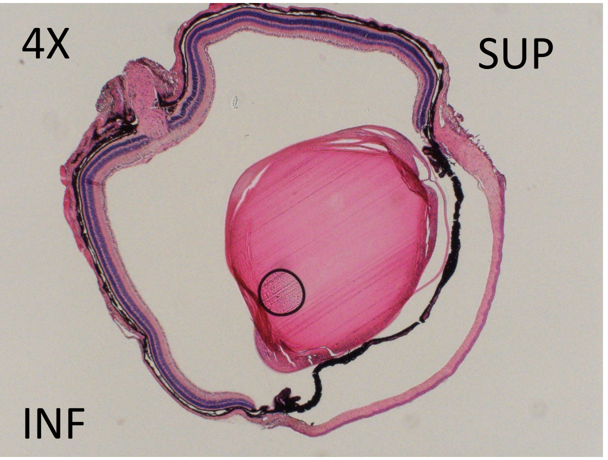

IC

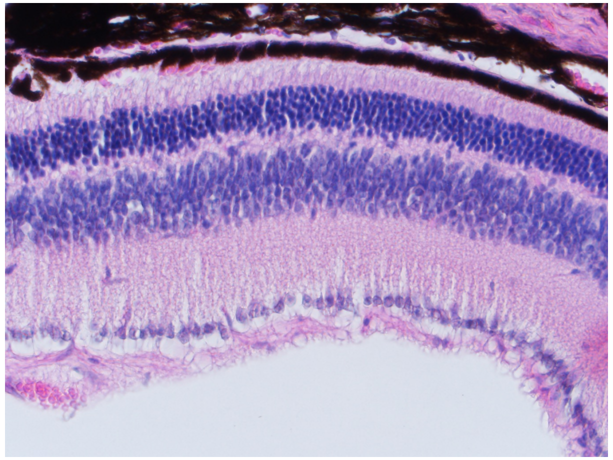

IE

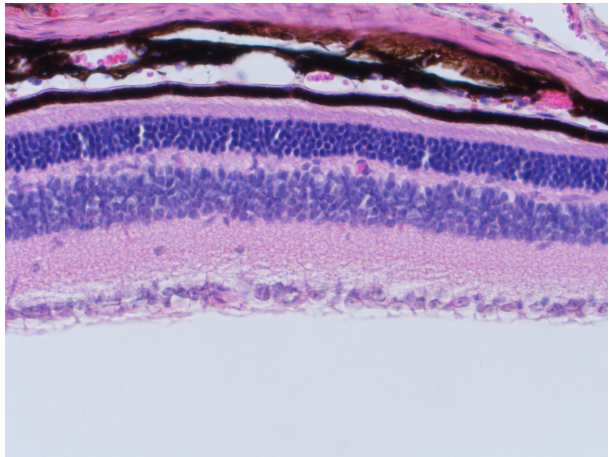

IP

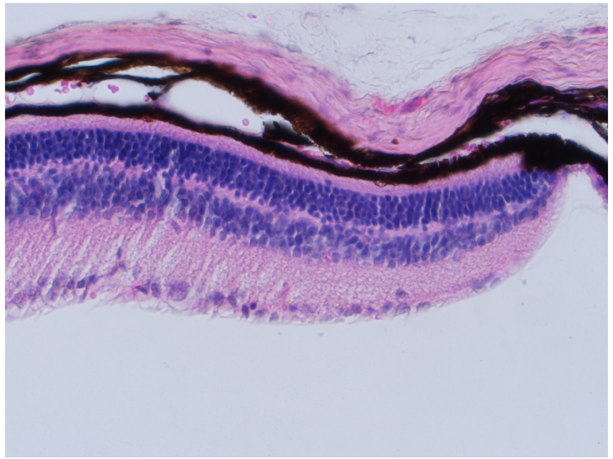

SP

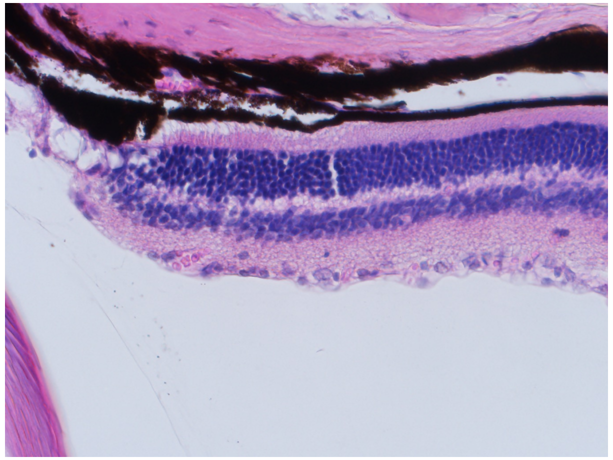

SE

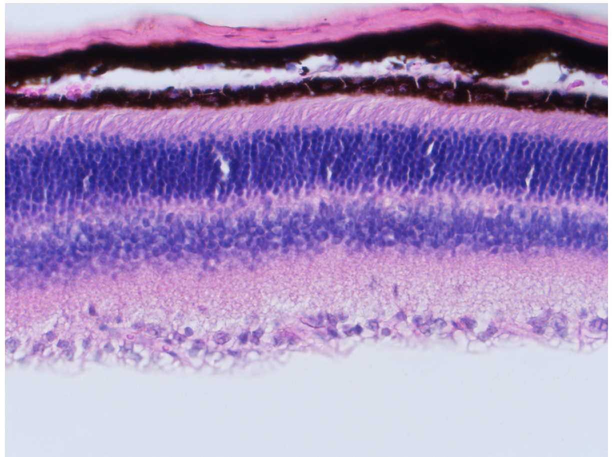

SC

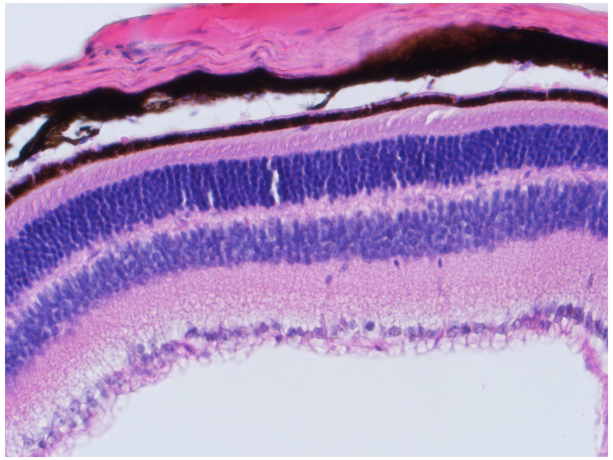

Trial1 mouse HE staining data PND53 20X—Rho$^{P23H/+}$ PBS-4

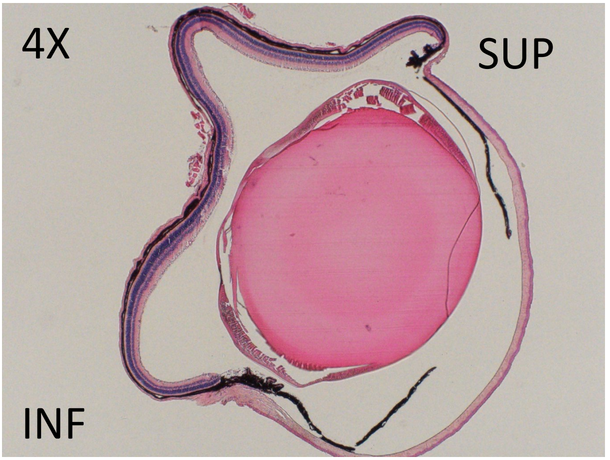

IC

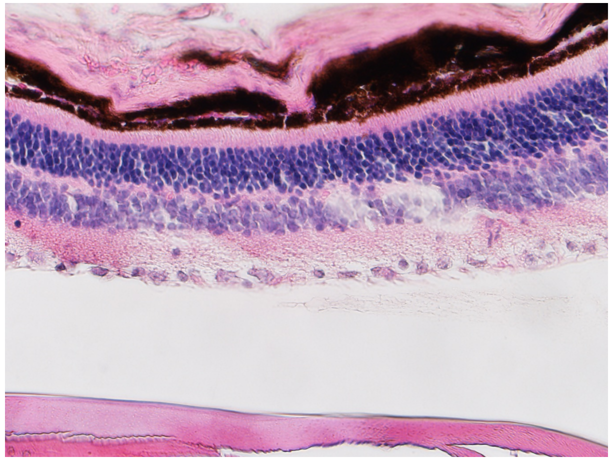

IE

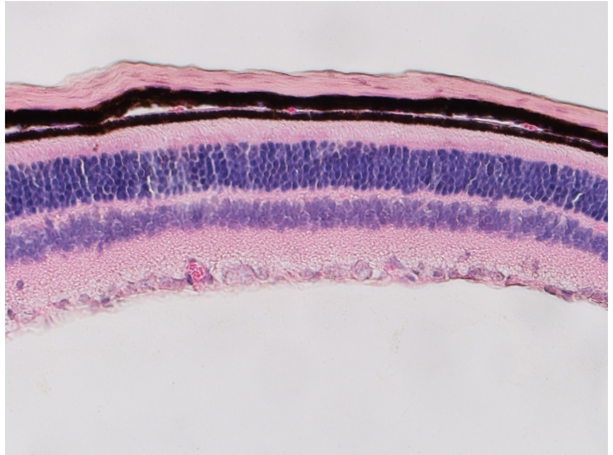

IP

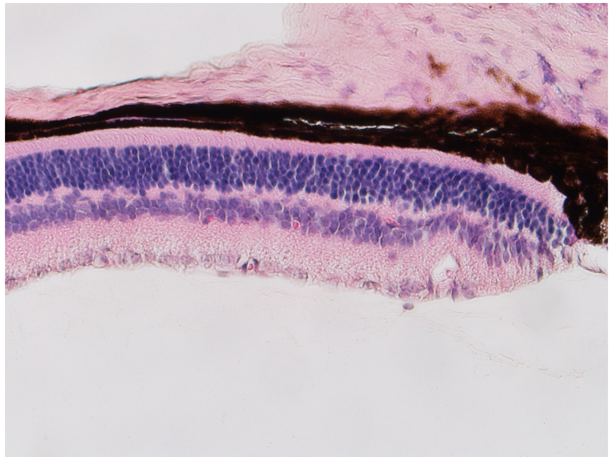

SP

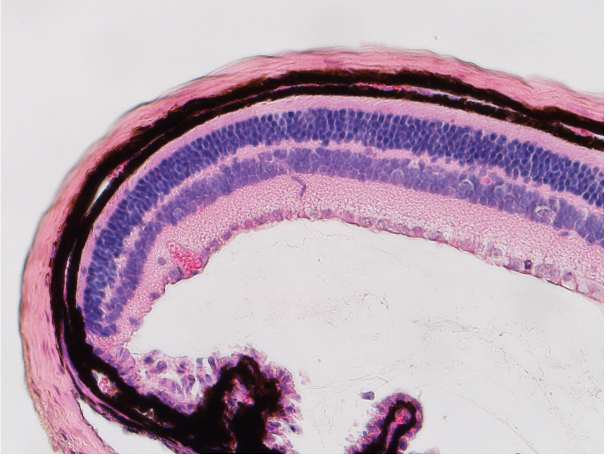

SE

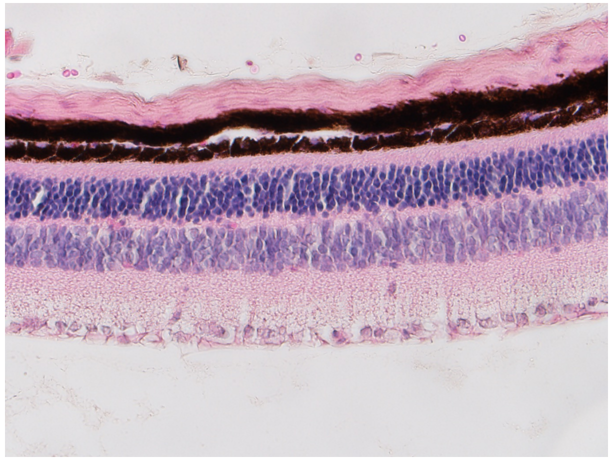

SC

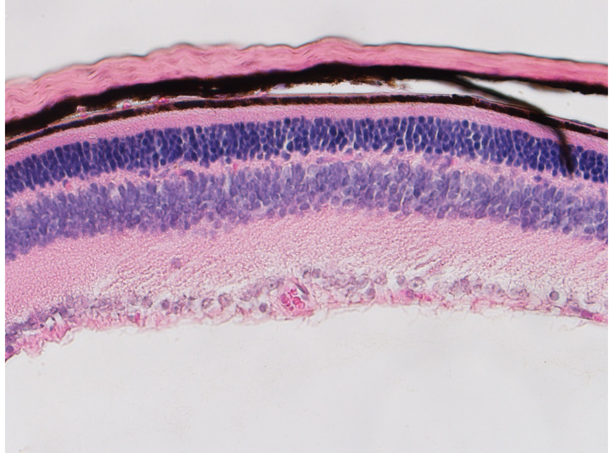

Trial1 mouse HE staining data PND53 20X—Rho$^{P23H/+}$ PBS-5

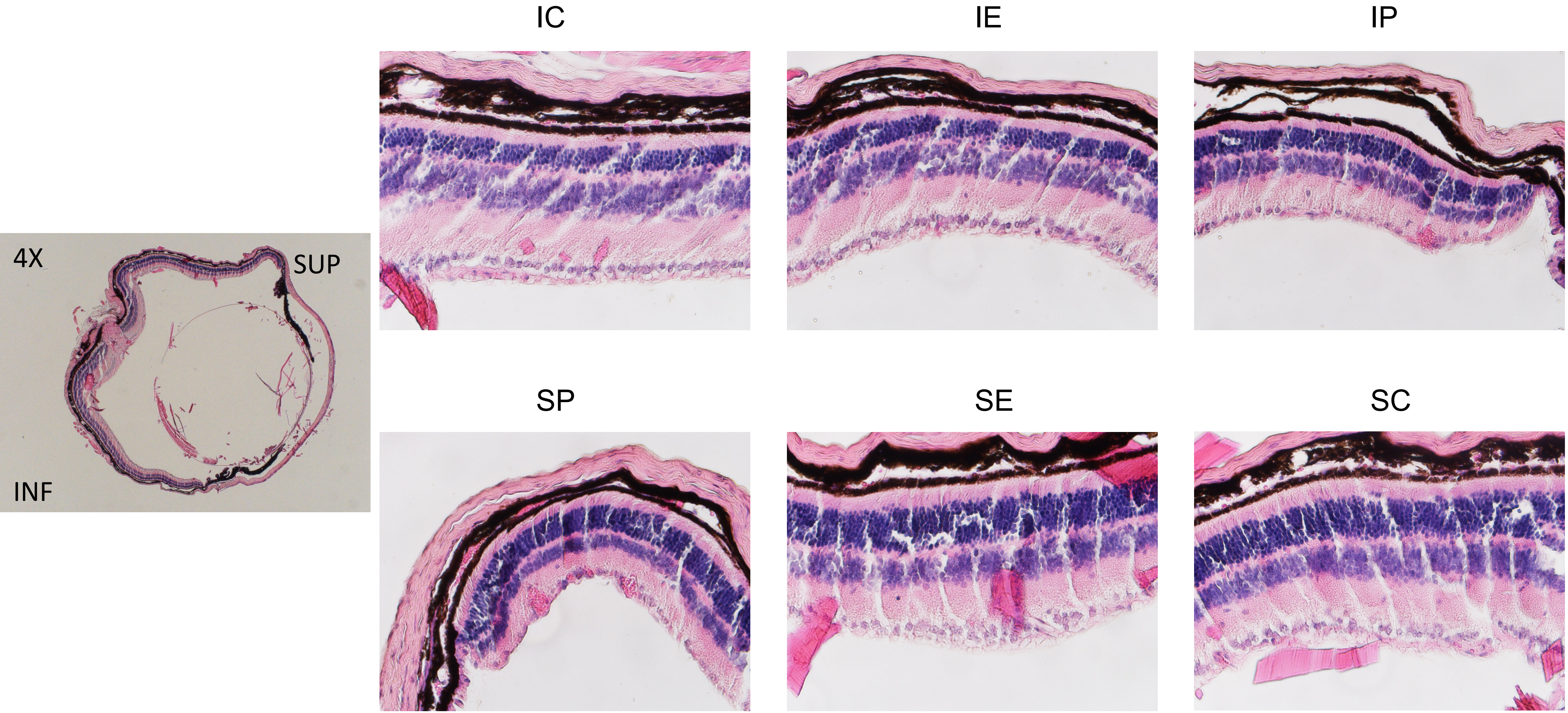

Trial1 mouse HE staining data PND53 20X—$Rho^{P23H/+}$ 8-AG-7

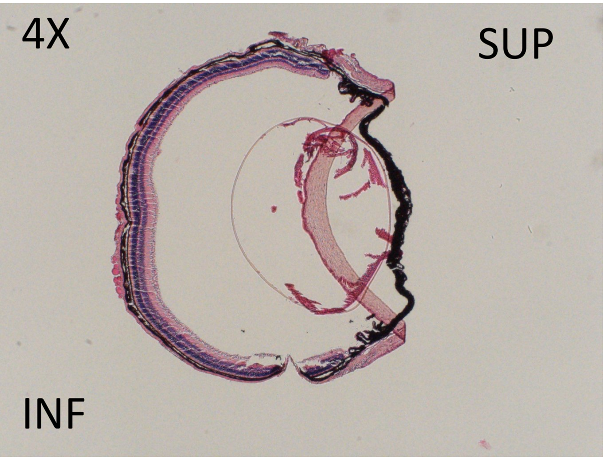

IC

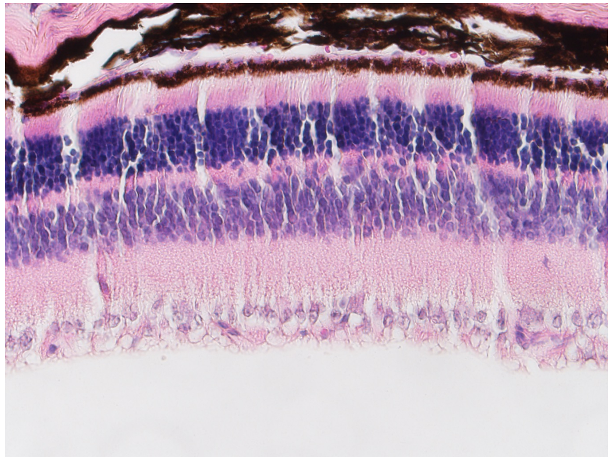

IE

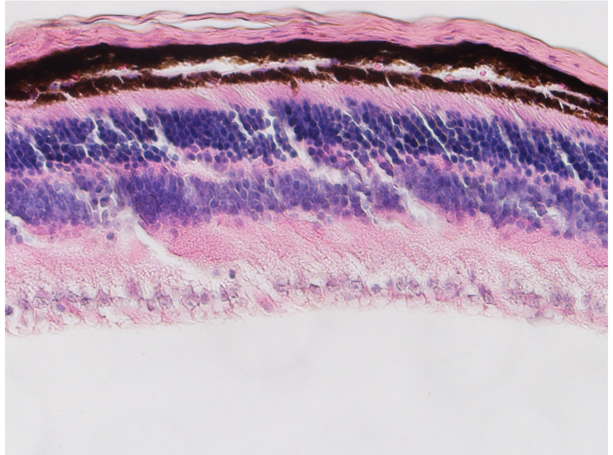

IP

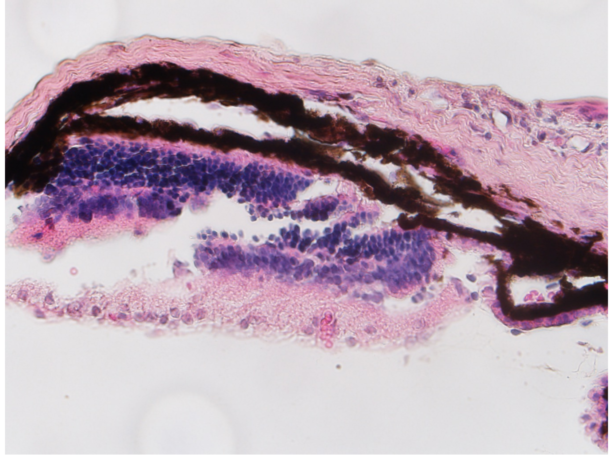

SP

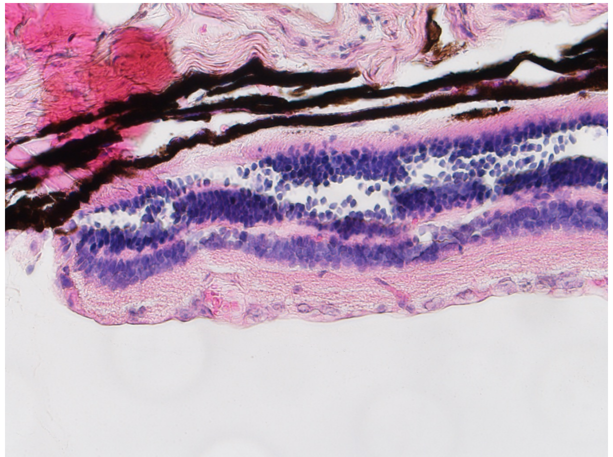

SE

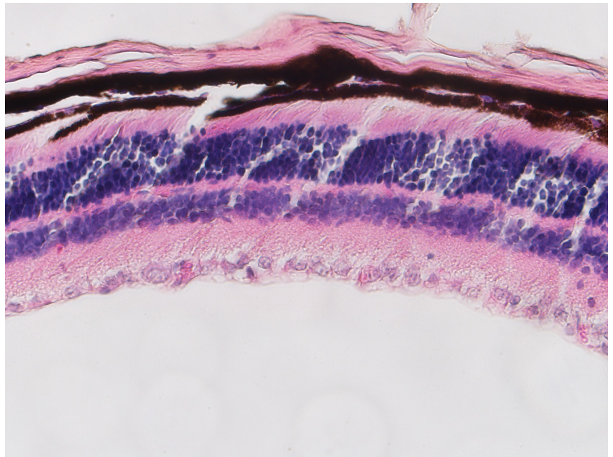

SC

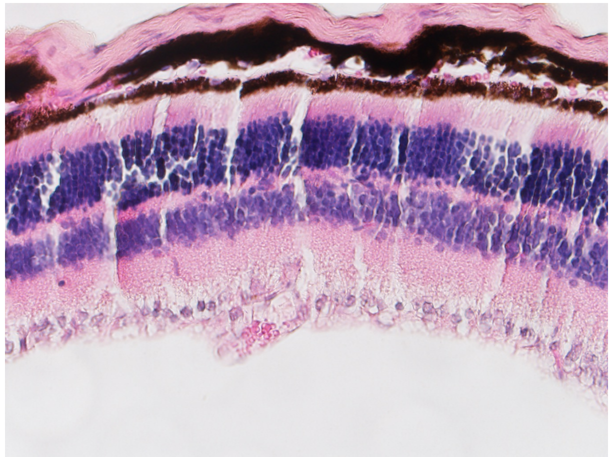

Trial1 mouse HE staining data PND53 20X—Rho$^{P23H/+}$ 8-AG-8

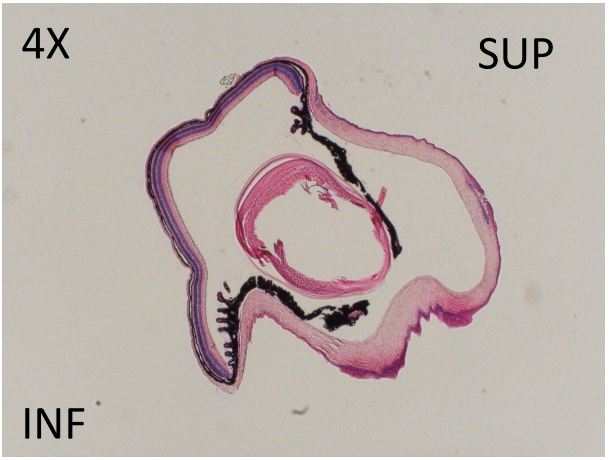

IC IE IP

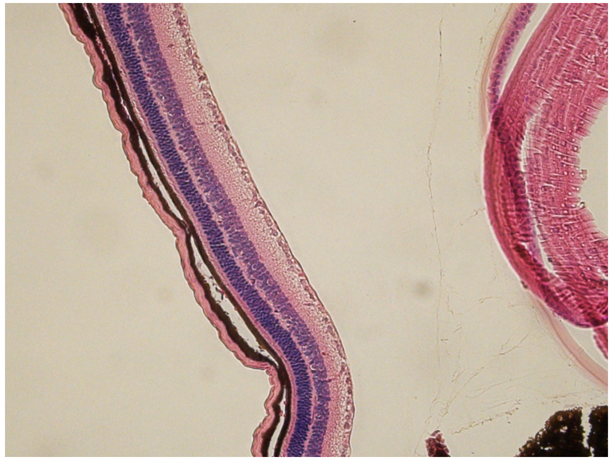
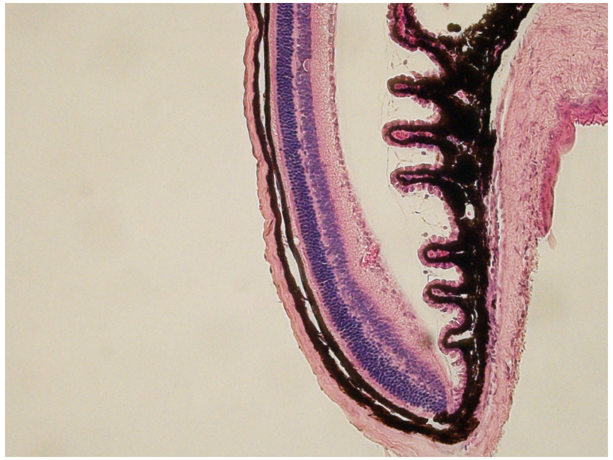

SC SE SP

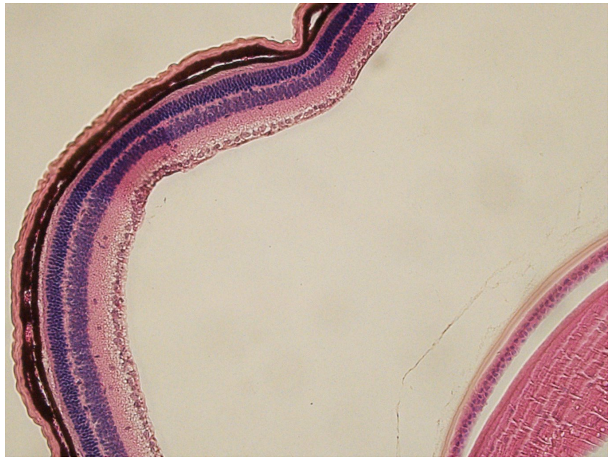
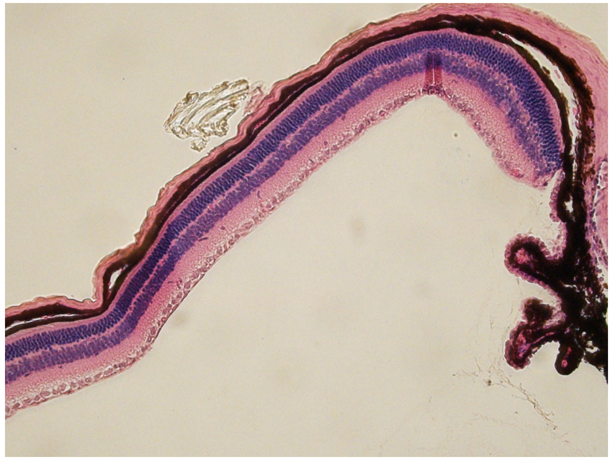

Trial1 mouse HE staining data PND53 20X—Rho$^{P23H/+}$ 8-AG-9

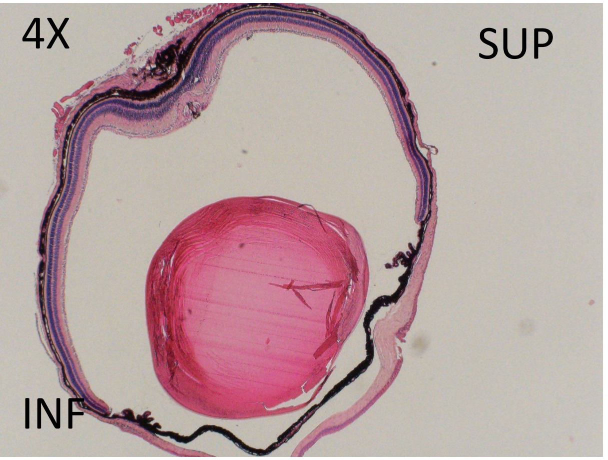

IC

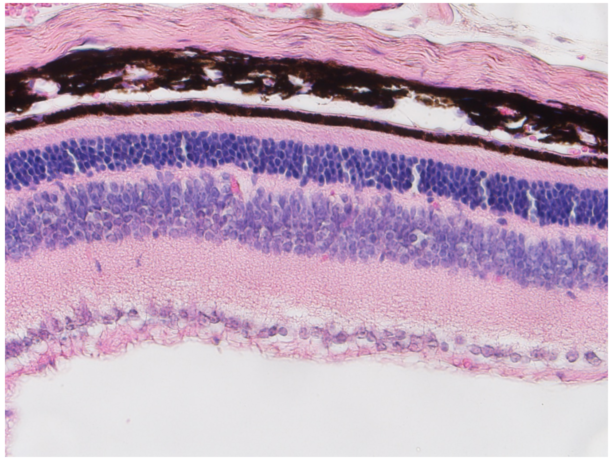

IE

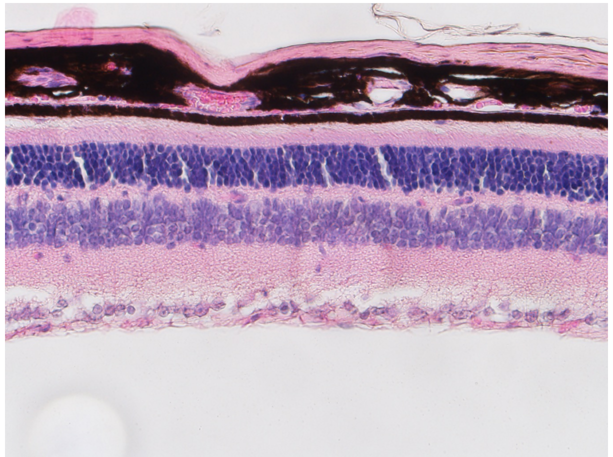

IP

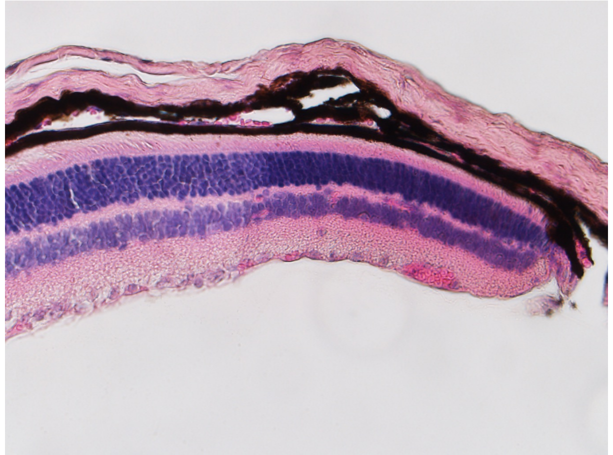

SP

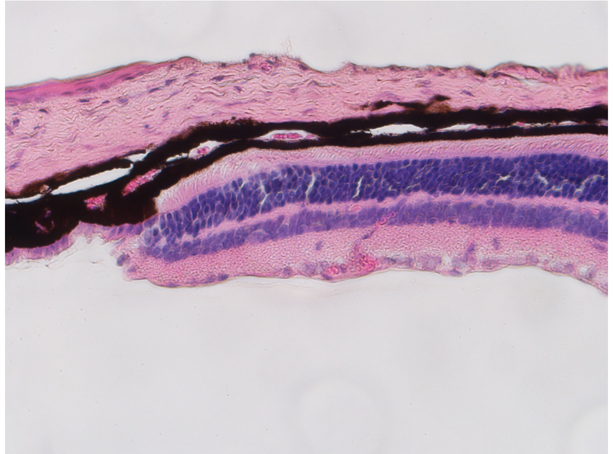

SE

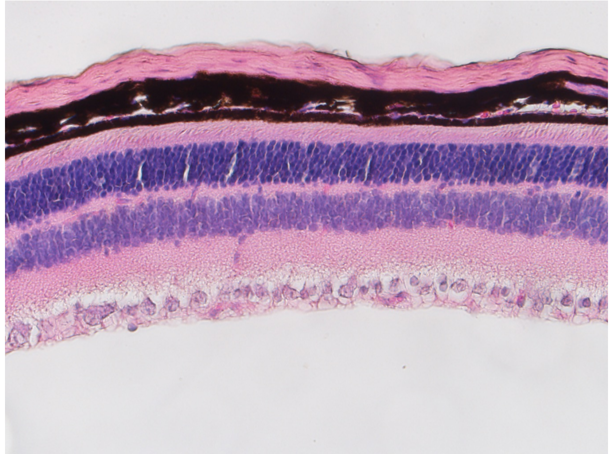

SC

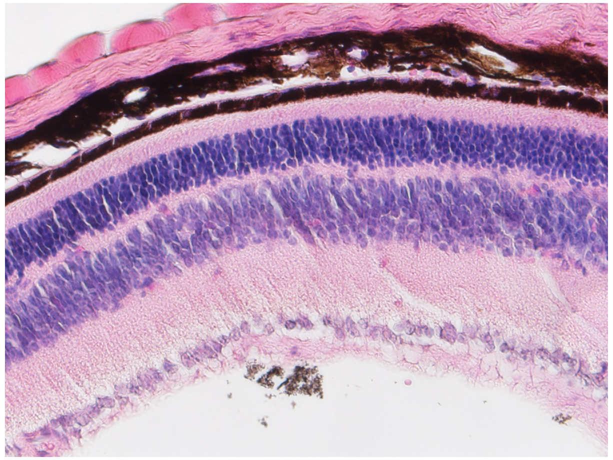

Trial1 mouse HE staining data PND53 20X—Rho$^{P23H/+}$ 8-AG-11

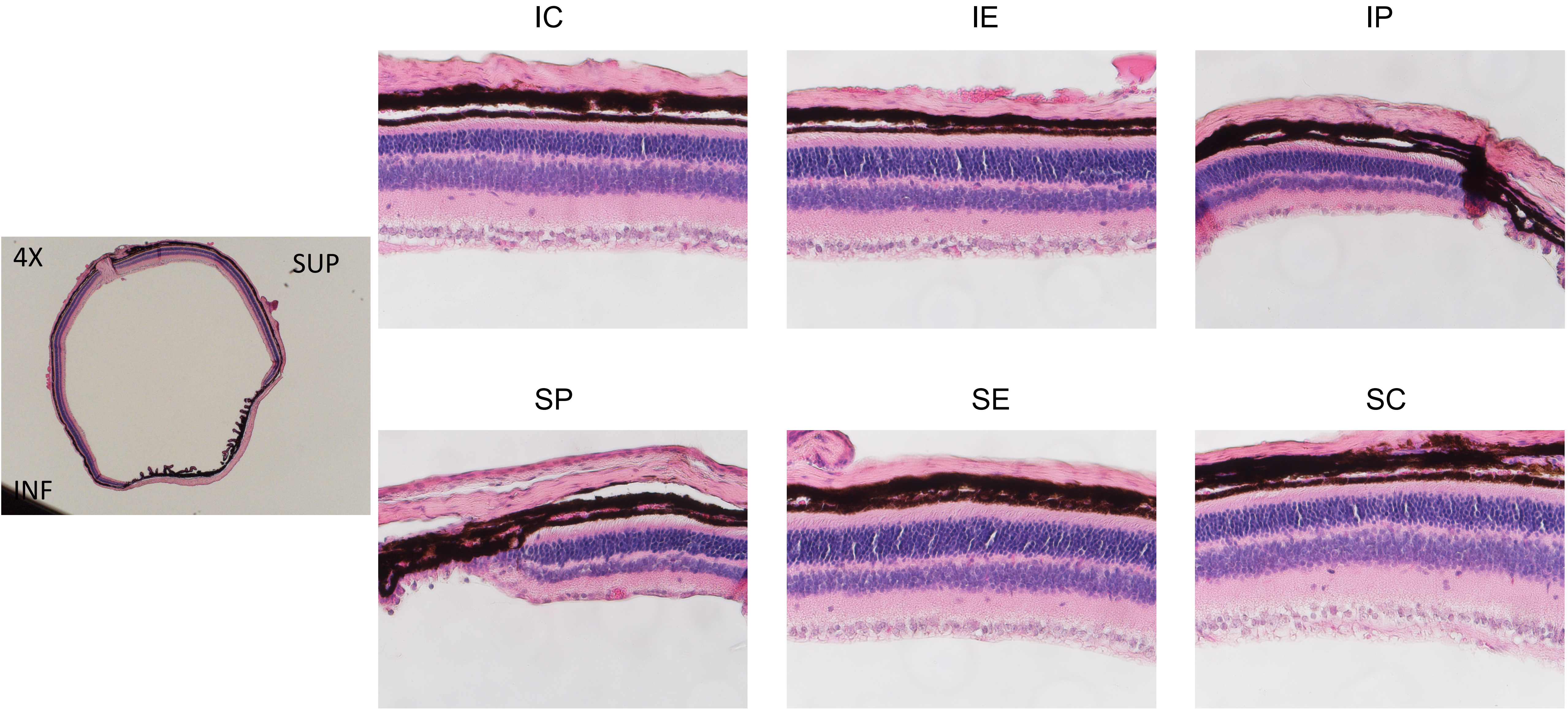

Tria-5 Oral feeding & IP injection of 8AG in Rho$^{P23H/+}$ mice

- Trial-5 P23H/+ mice 8AG test(IP & Oral start from PND10, test OCT/ERG on PND28/29 and PND35/36)
  - DOB:12/28/2020—two cages,6 pups per cage, one for water, one for 8AG
  - PND 10=01/07/2021 start the treatment, 11 mg/kg.bw IP & 11 mg/kg.bw oral
  - PND 28/29 OCT/ERG =01/25/2021 and 01/26/2021
  - 6X2=12 eyes per group, 4 for cryo-section, 4 for H&E staining, 4 for flat mount staining

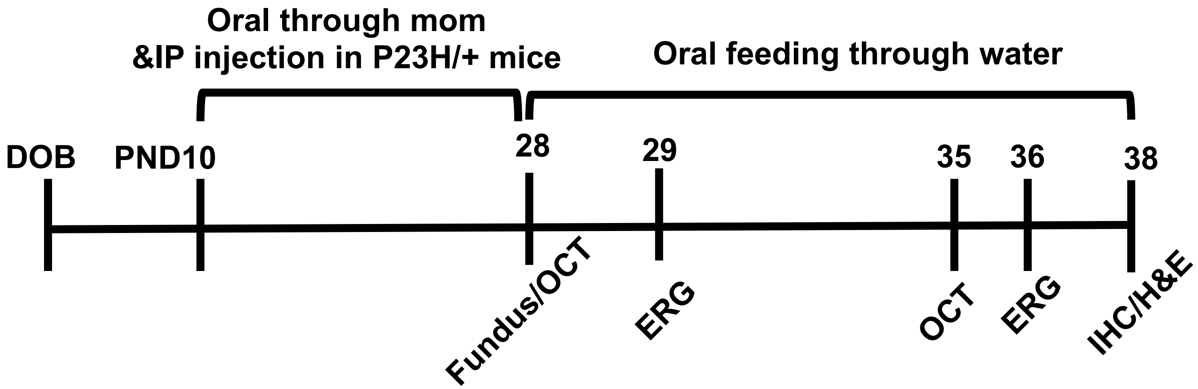

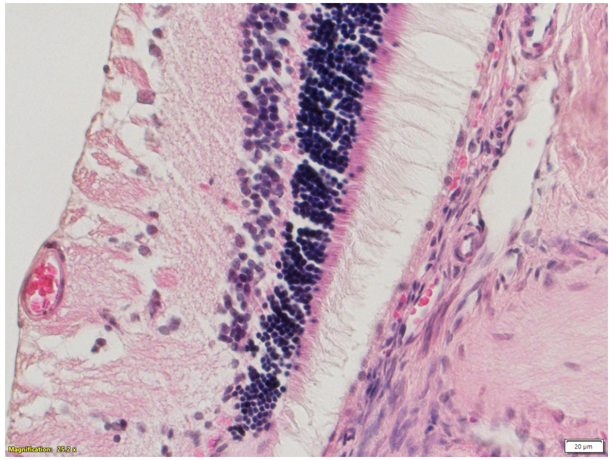

Trial5 mouse HE staining data PND38 40X—$Rho^{+/+}$ 1

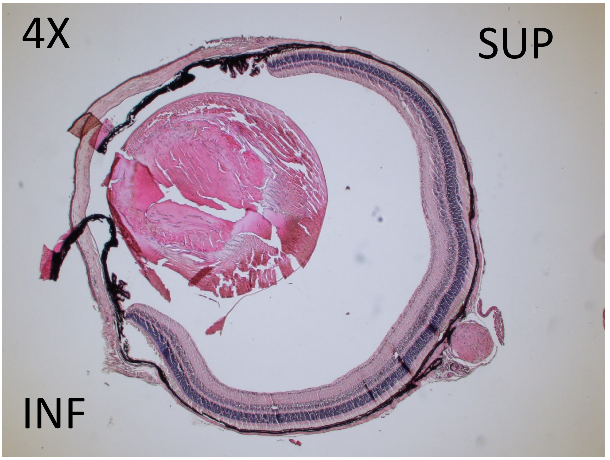

IP

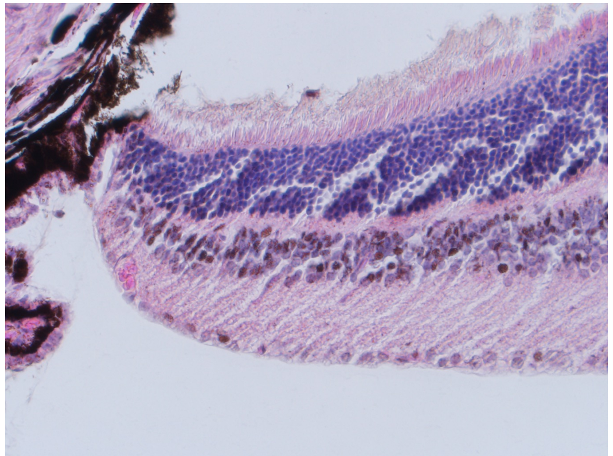

IE

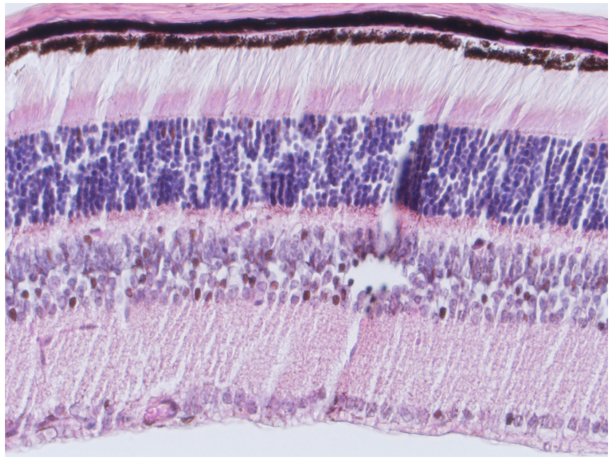

IC

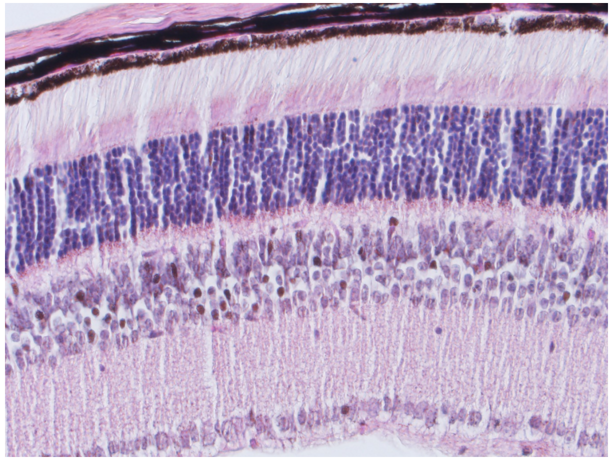

SC

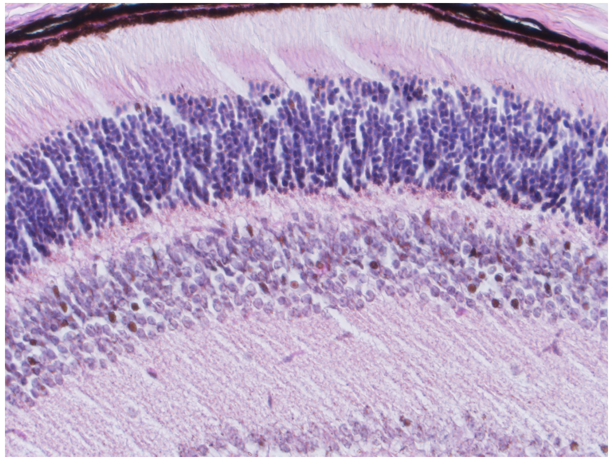

SE

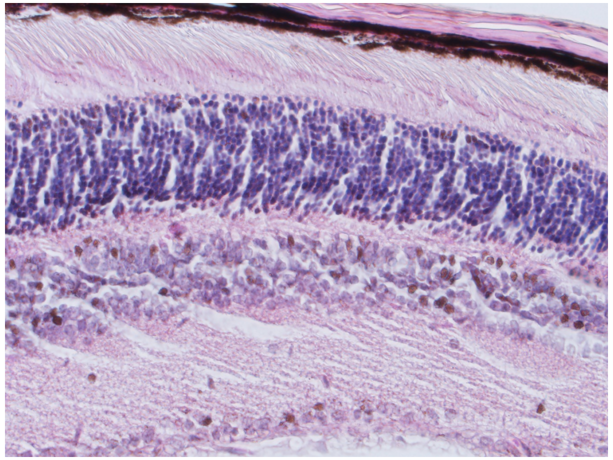

SP

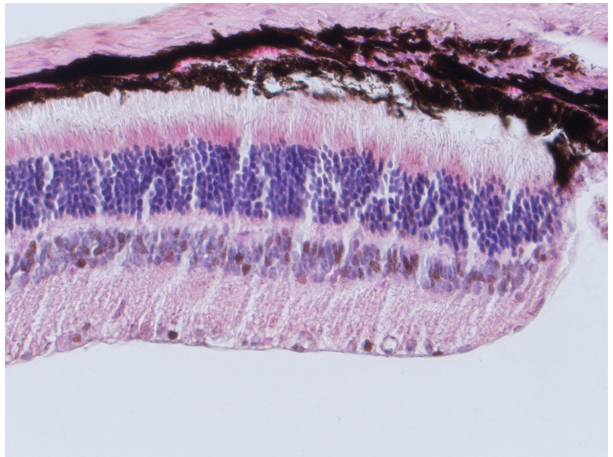

Trial5 mouse HE staining data PND38 40X—Rho$^{+/+}$ 2

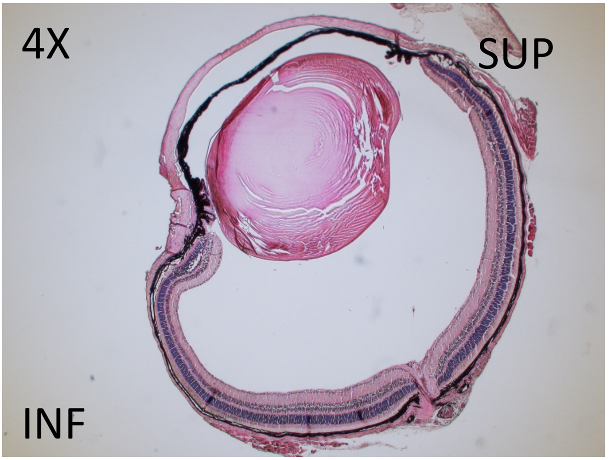

IP

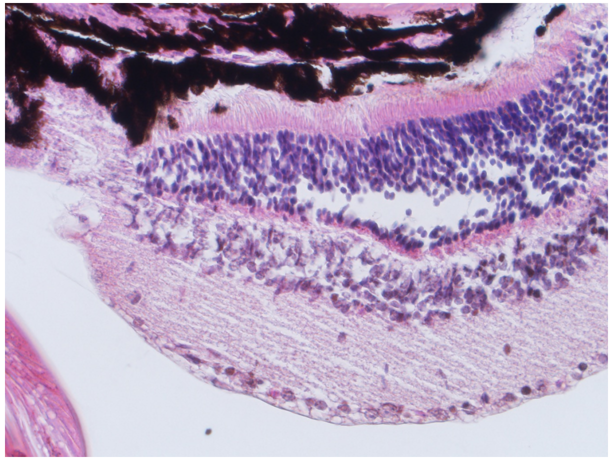

IE

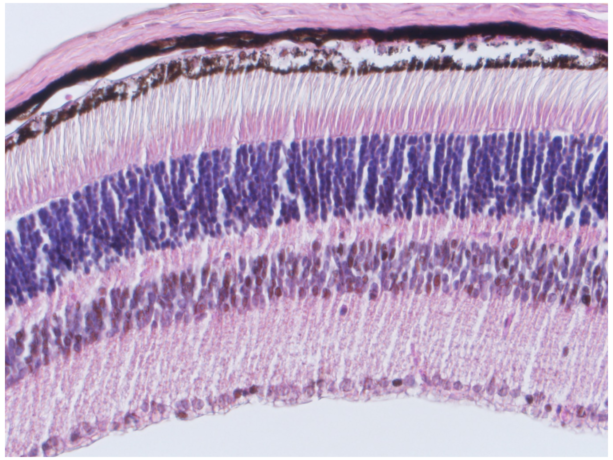

IC

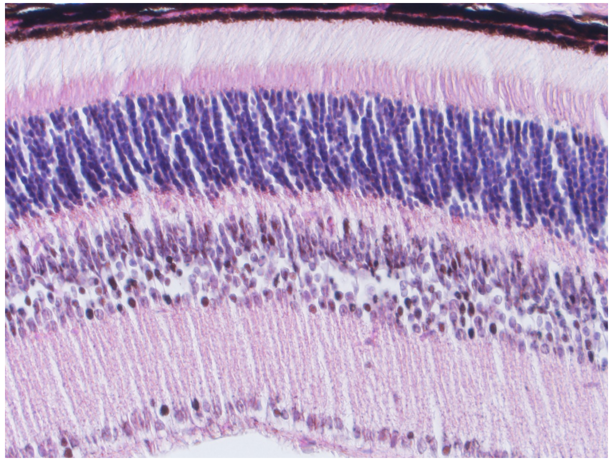

SC

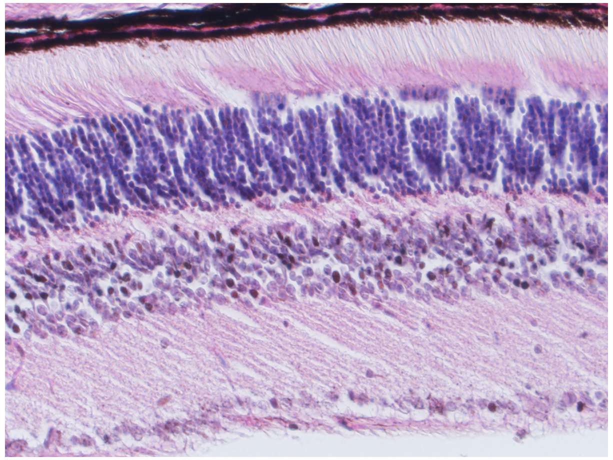

SE

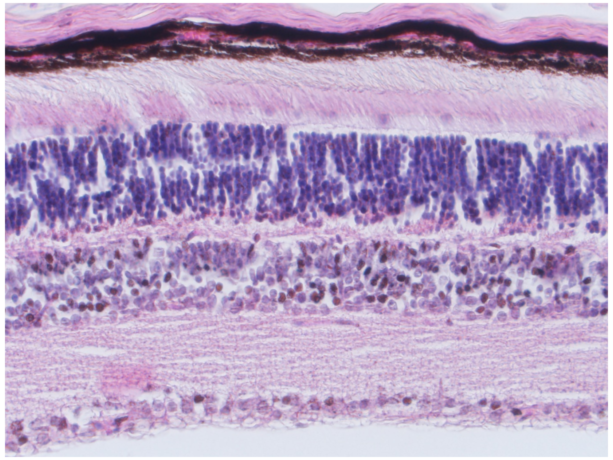

SP

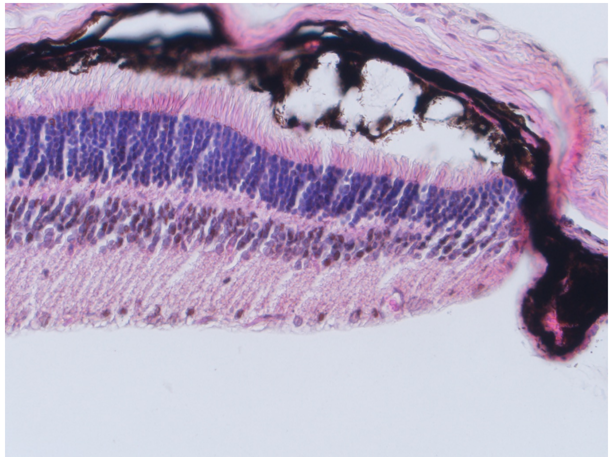

Trial5 mouse HE staining data PND38 40X—Rho$^{+/+}$ 3

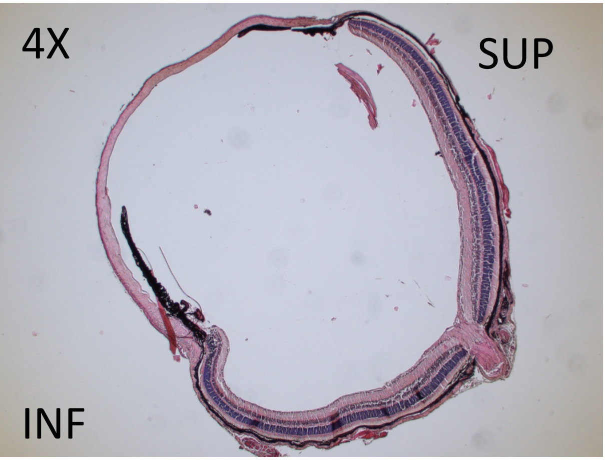

IP

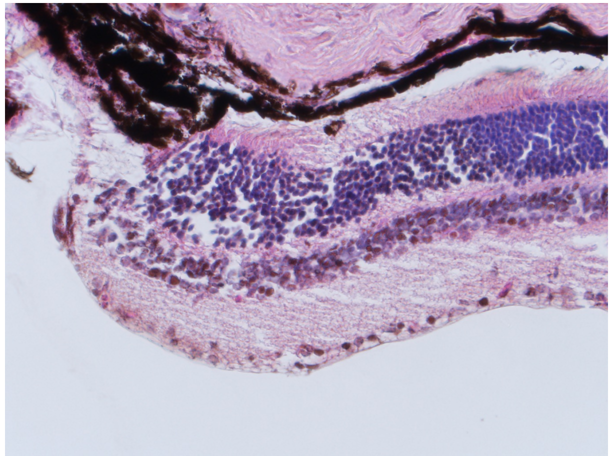

IE

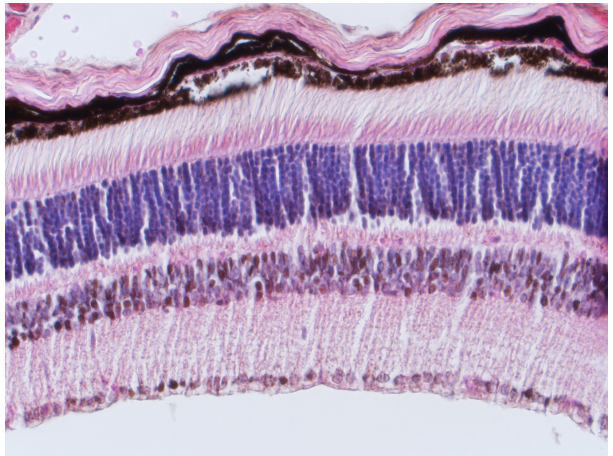

IC

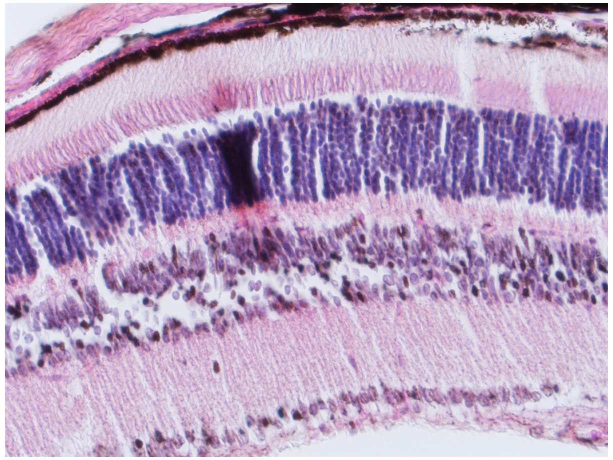

SC

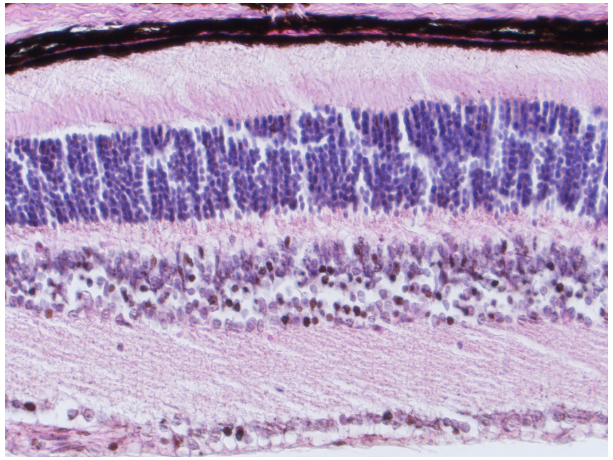

SE

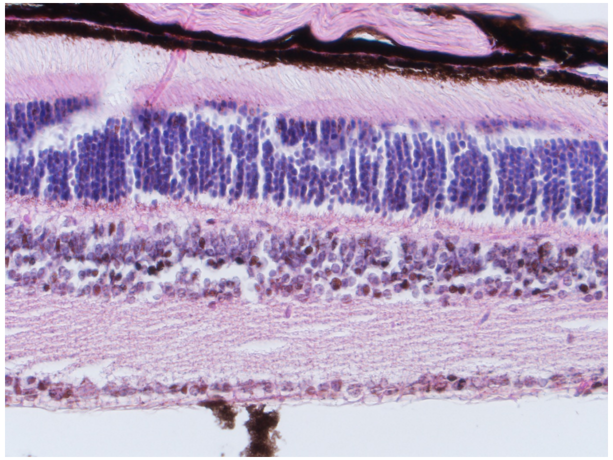

SP

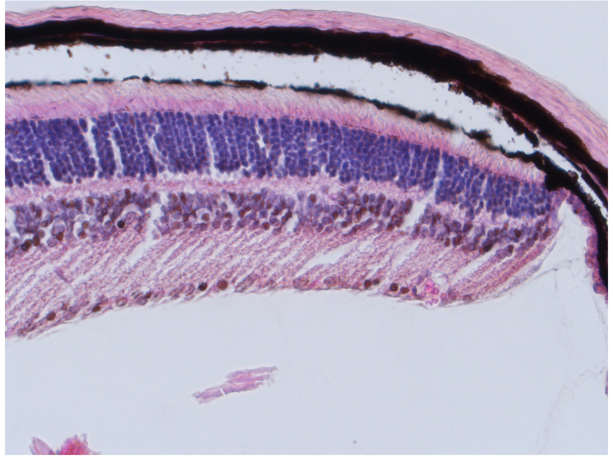

Trial5 mouse HE staining data PND38 40X—Rho$^{+/+}$ 4

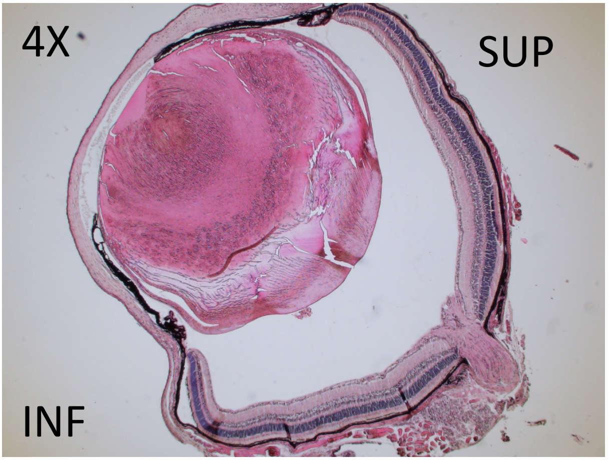

IP

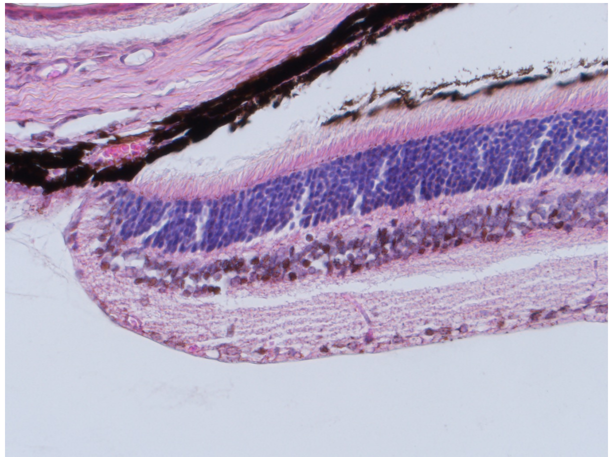

IE

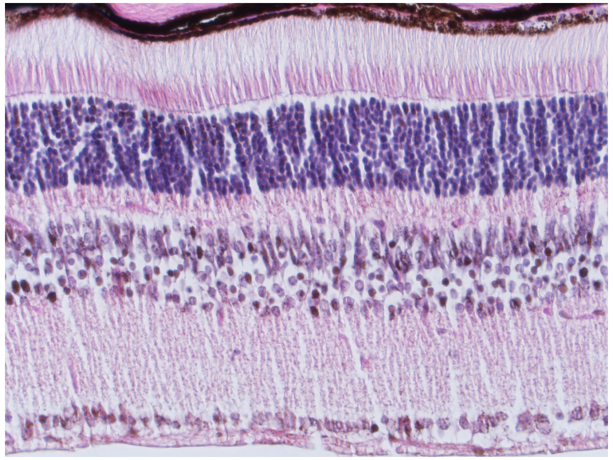

IC

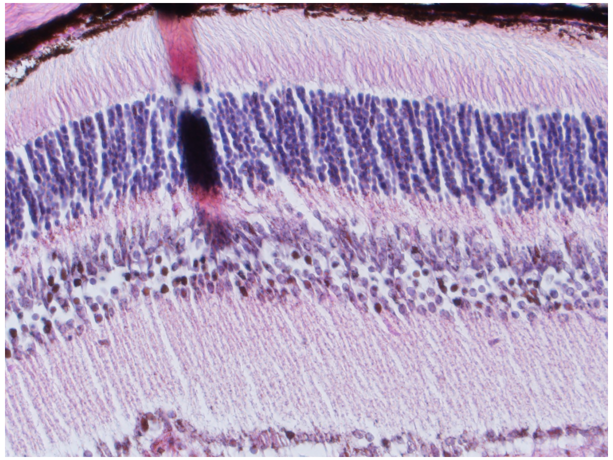

SC

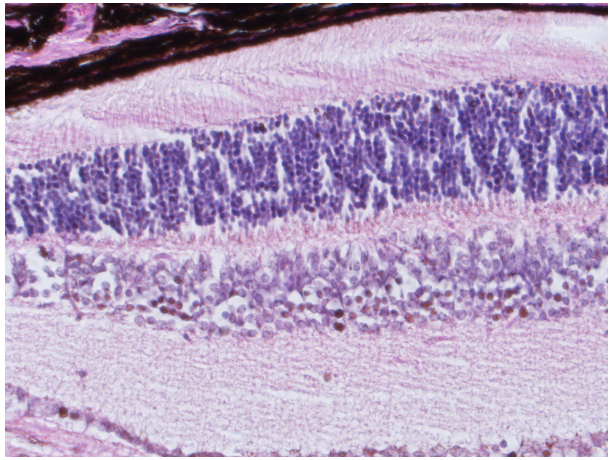

SE

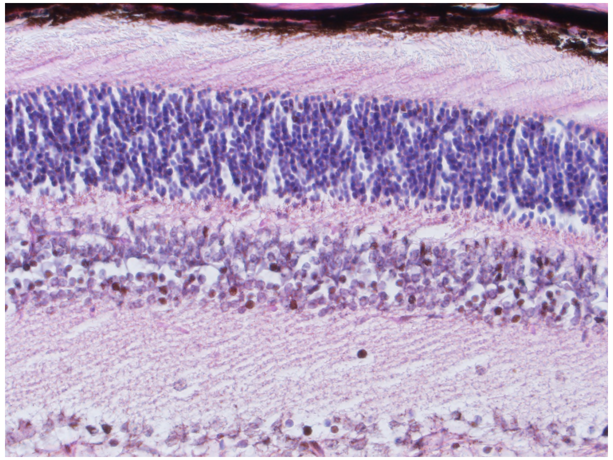

SP

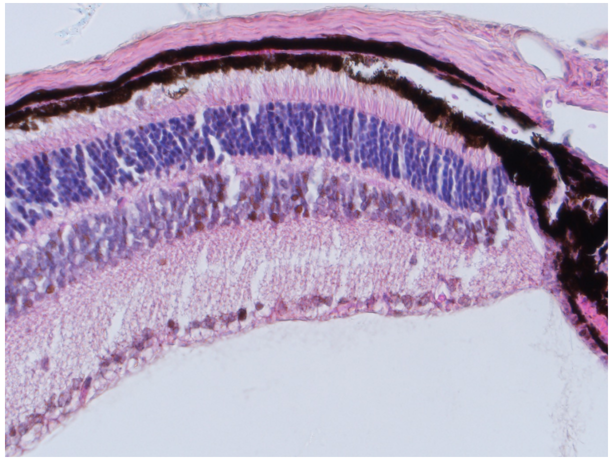

Trial5 mouse HE staining data PND38 40X—Rho$^{P23H/+}$ PBS-1

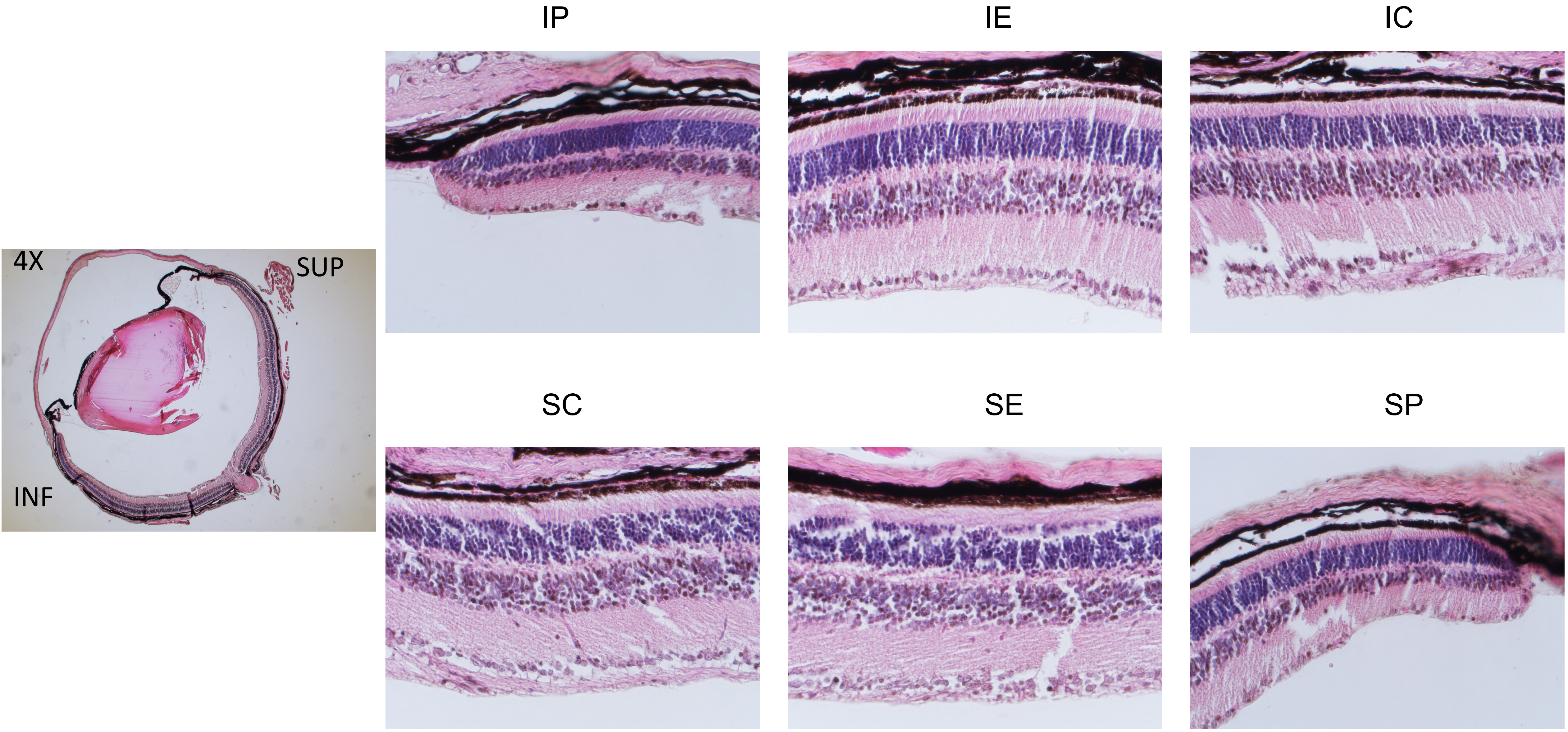

Trial5 mouse HE staining data PND38 40X—Rho$^{P23H/+}$ PBS-2

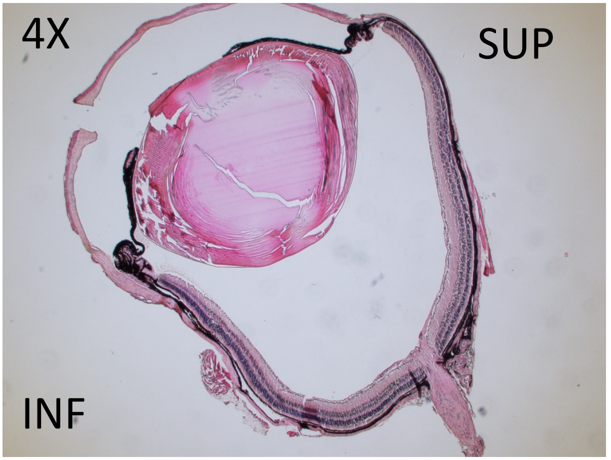

IP

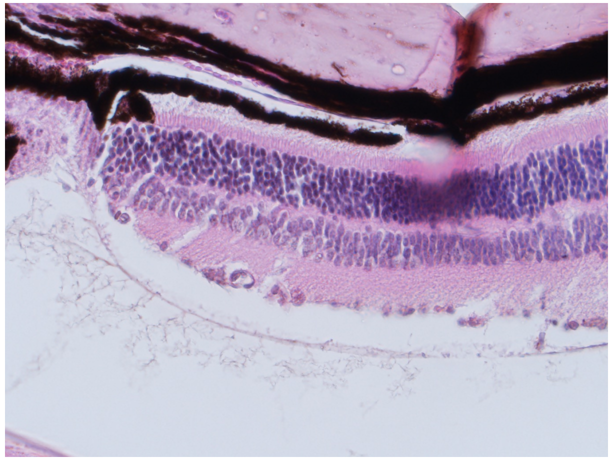

IE

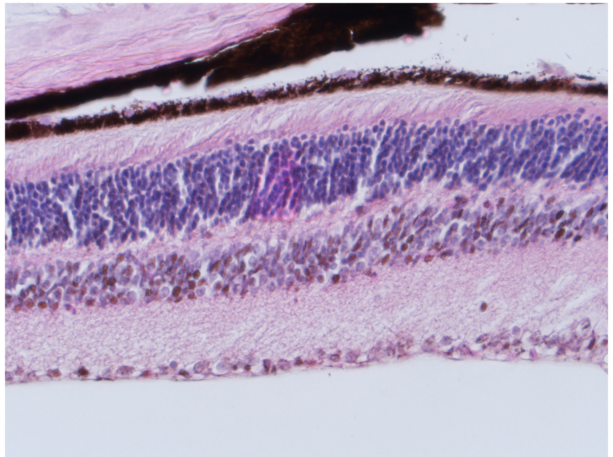

IC

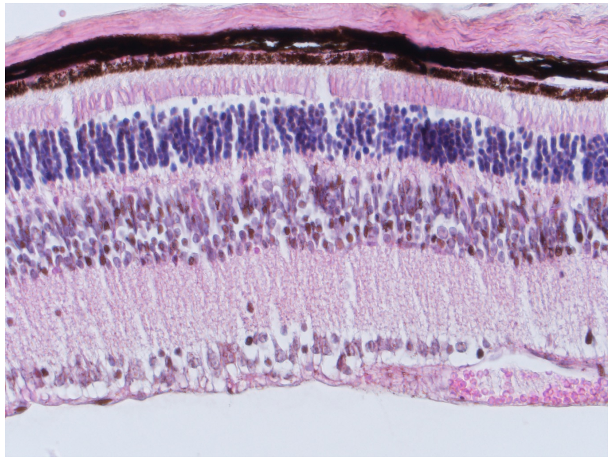

SC

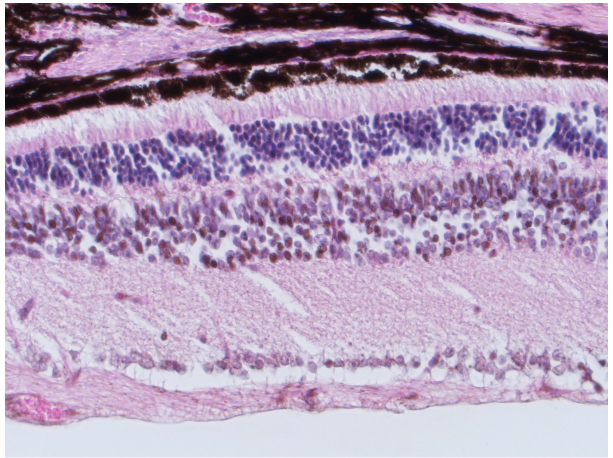

SE

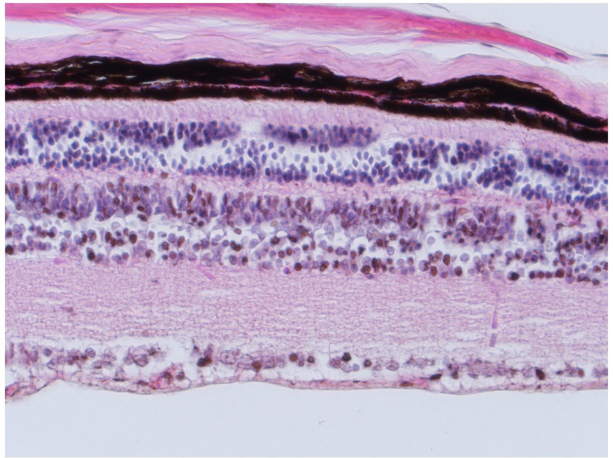

SP

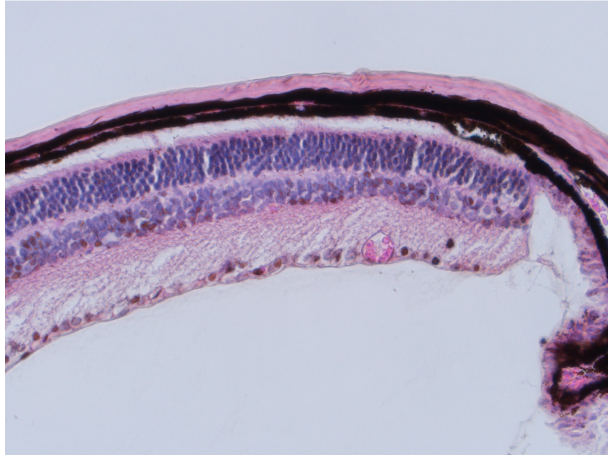

Trial5 mouse HE staining data PND38 40X—Rho$^{P23H/+}$ PBS-3

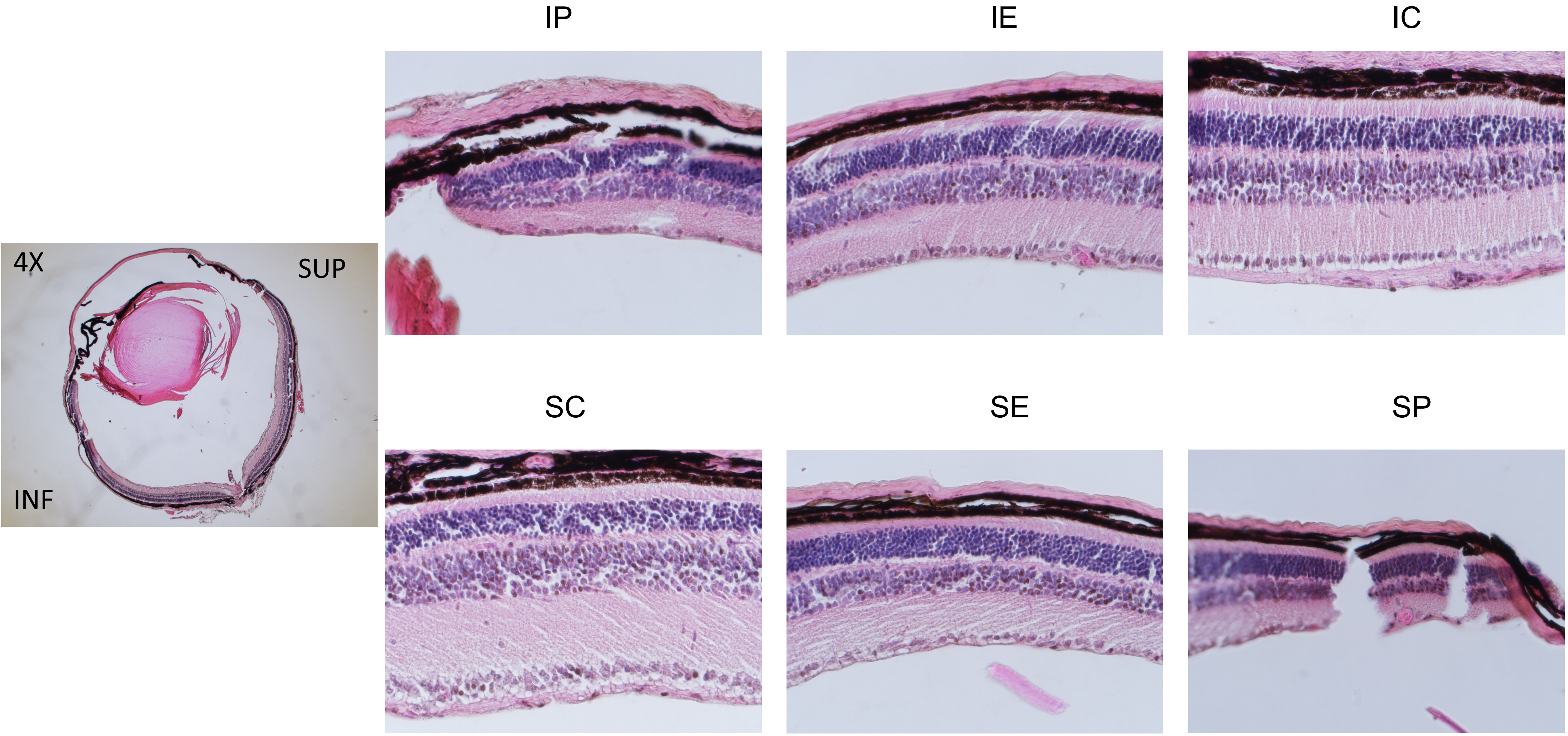

Trial5 mouse HE staining data PND38 40X—Rho$^{P23H/+}$ PBS-4

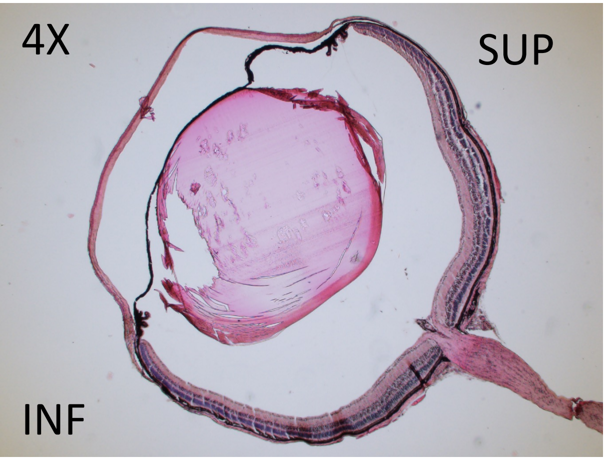

IP

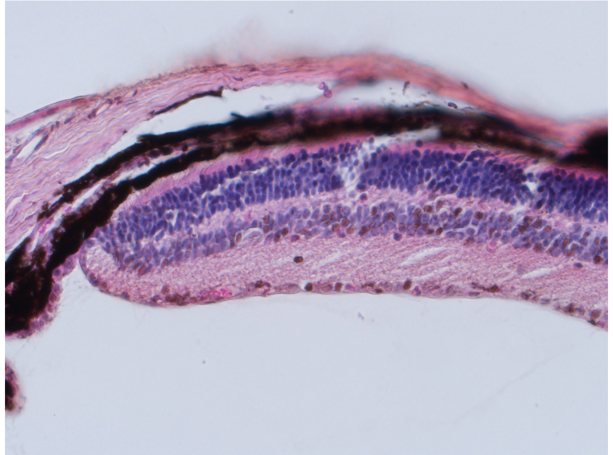

IE

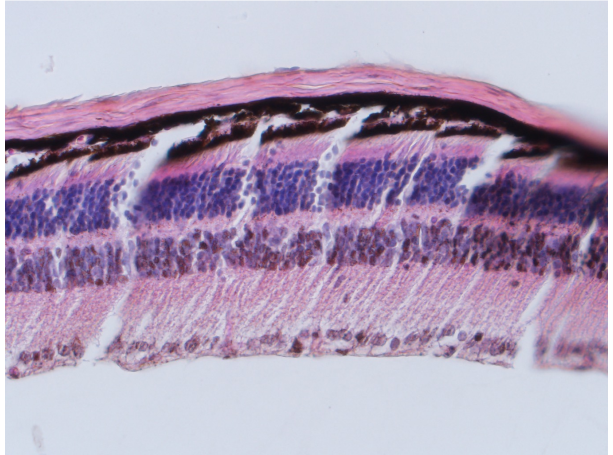

IC

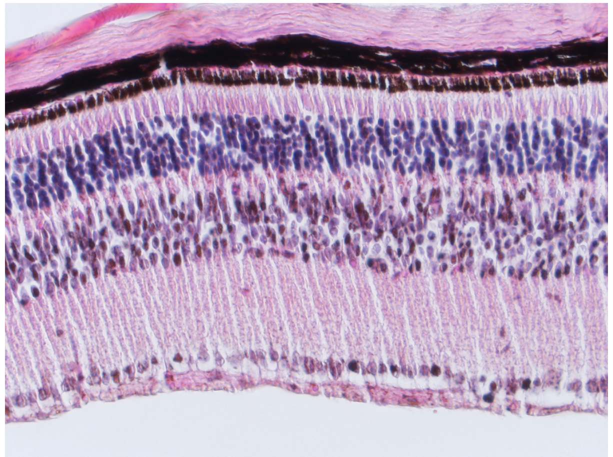

SC

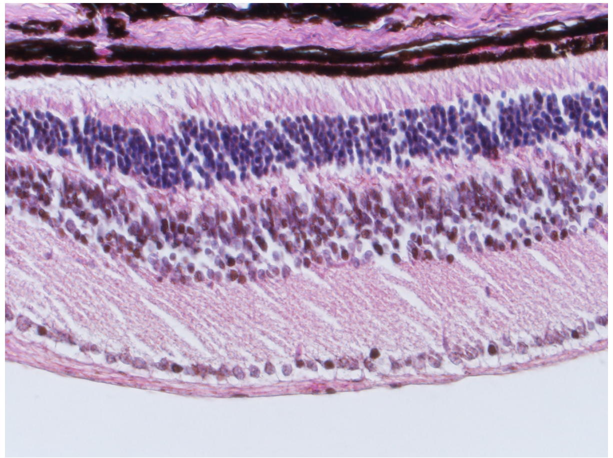

SE

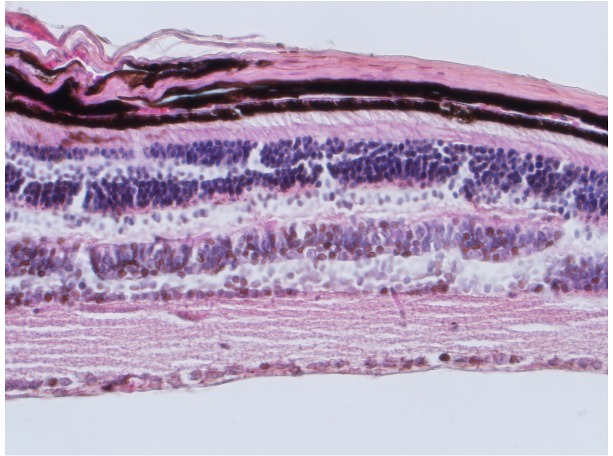

SP

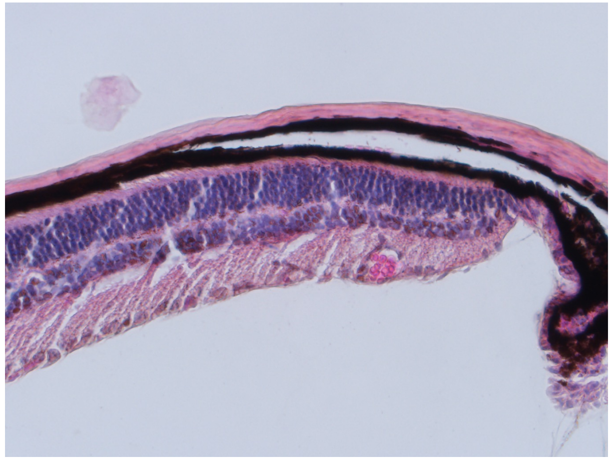

Trial5 mouse HE staining data PND38 40X—$Rho^{P23H/+}$ 8-AG-3

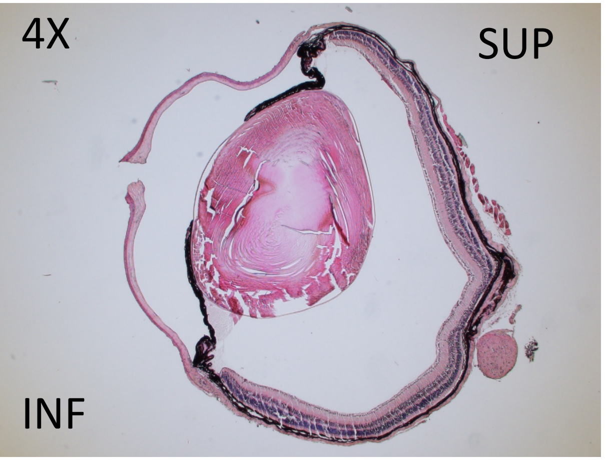

IP

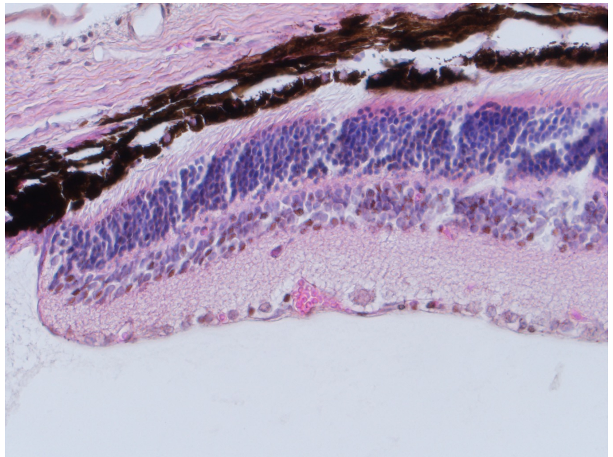

IE

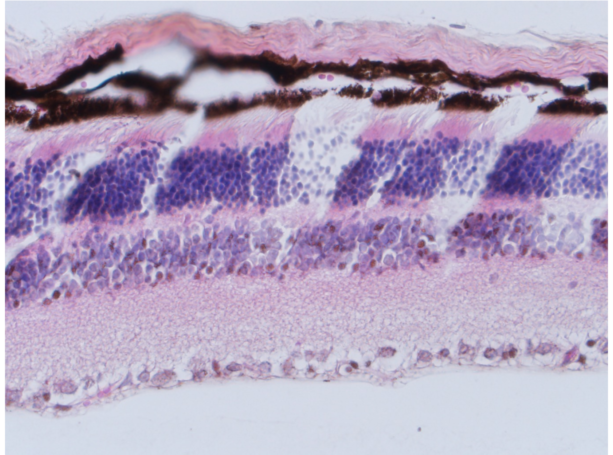

IC

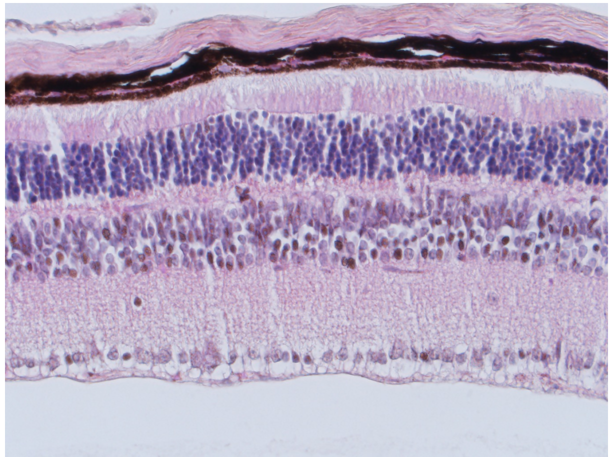

SC

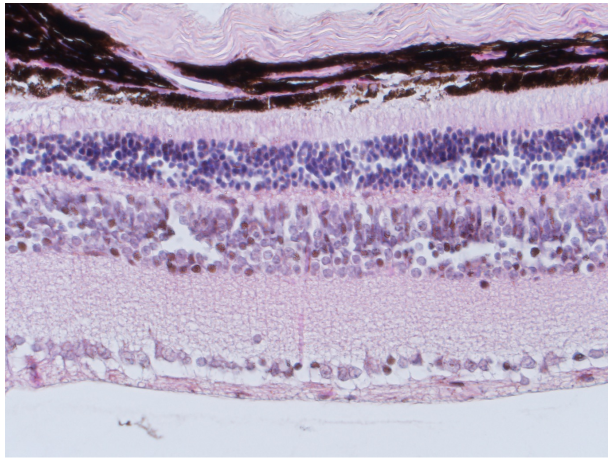

SE

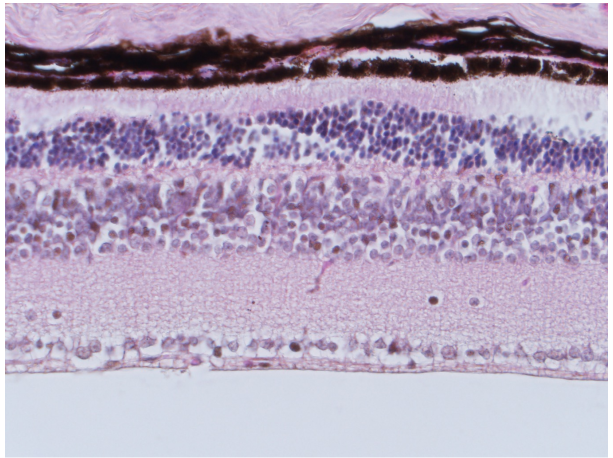

SP

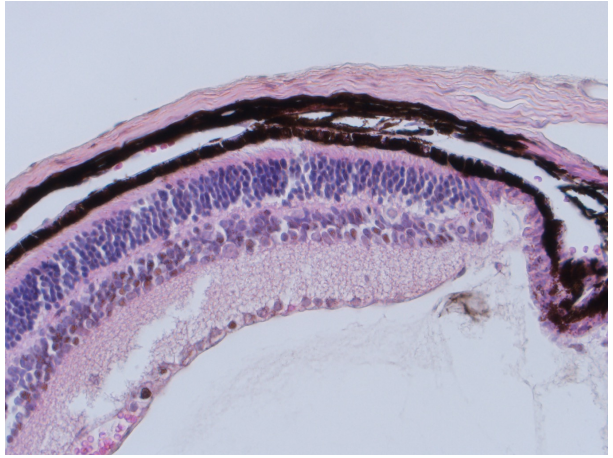

Trial5 mouse HE staining data PND38 40X—Rho$^{P23H/+}$ 8-AG-4

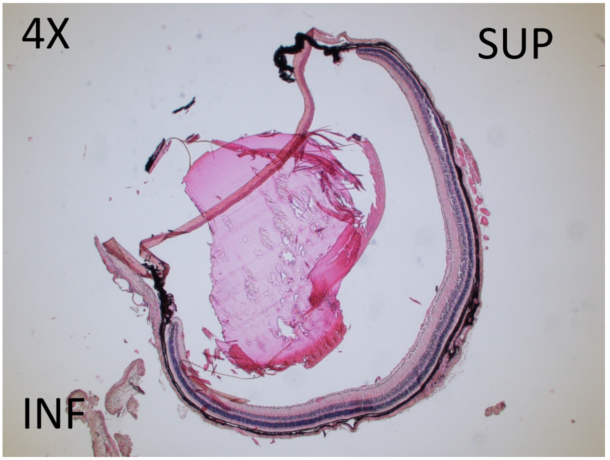

IP

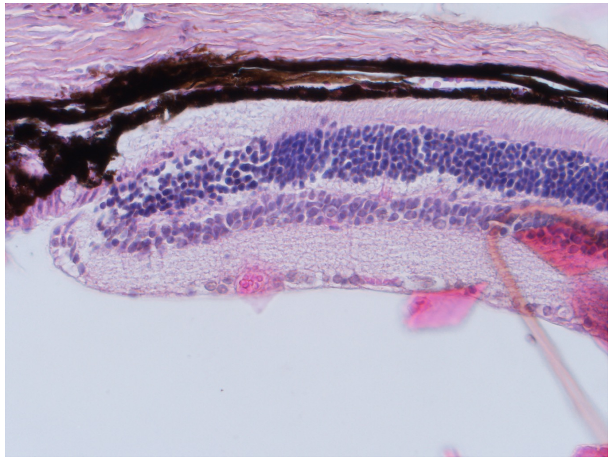

IE

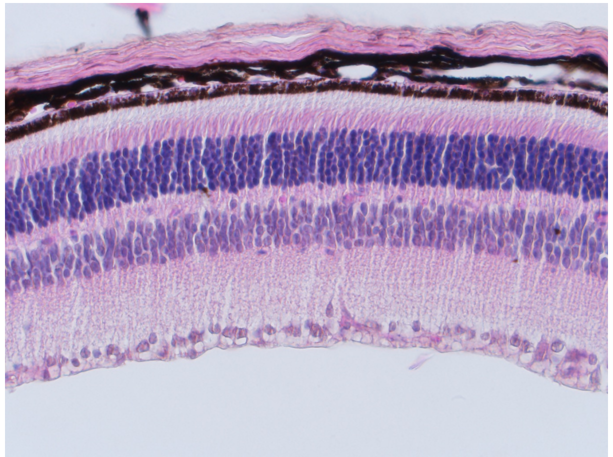

IC

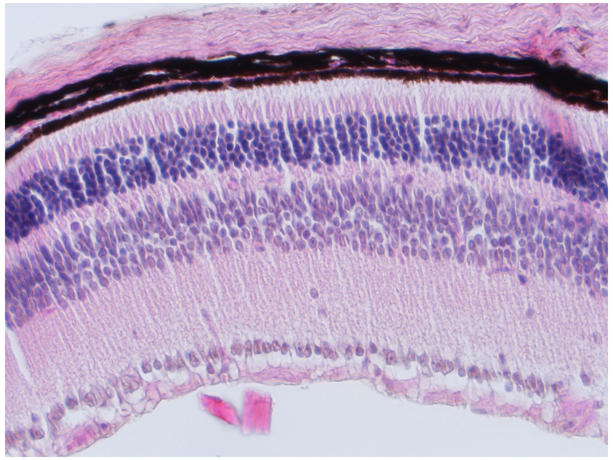

SC

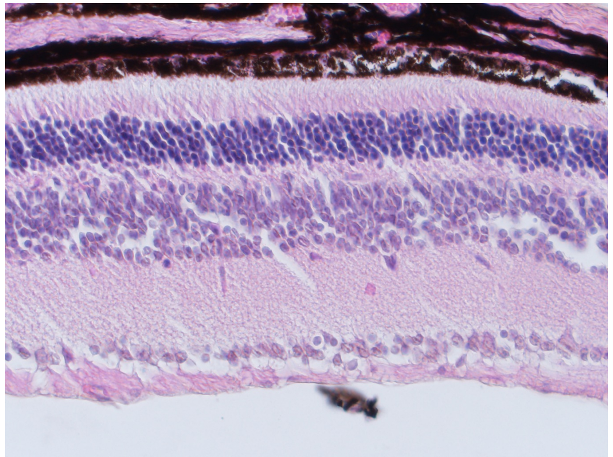

SE

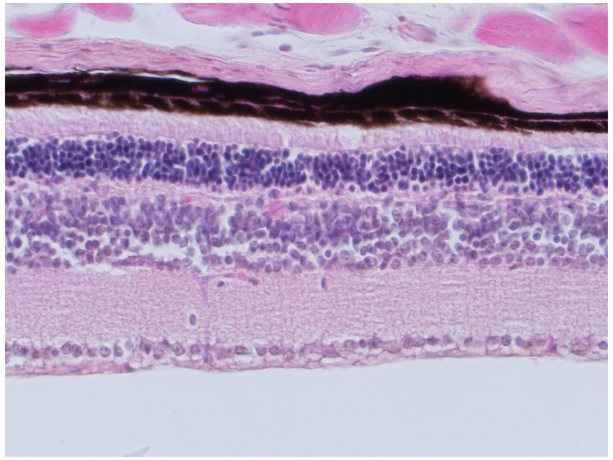

SP

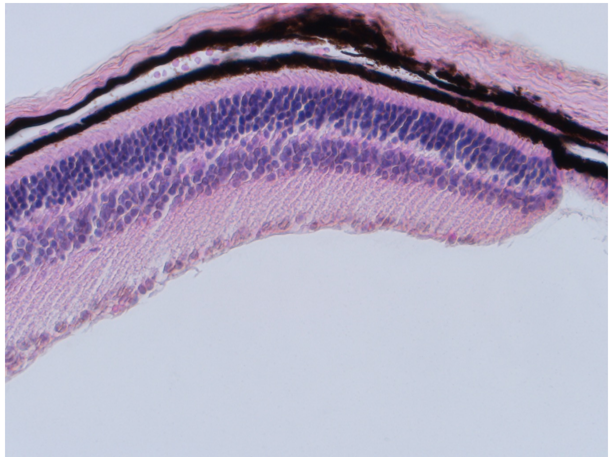

Trial5 mouse HE staining data PND38 40X—Rho$^{P23H/+}$ 8-AG-5

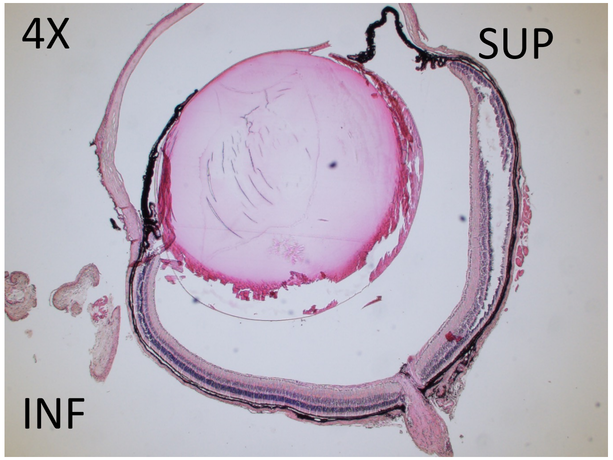

IP

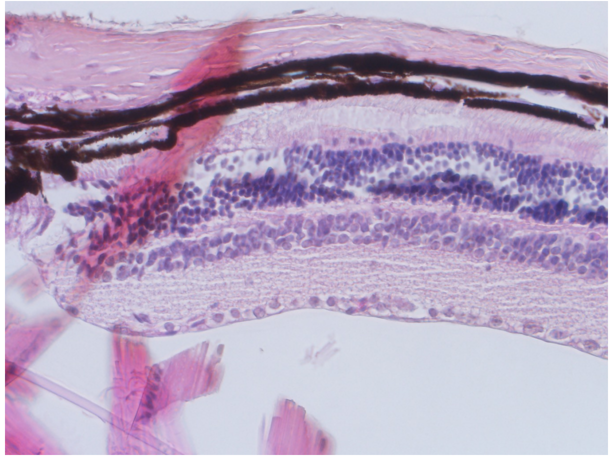

IE

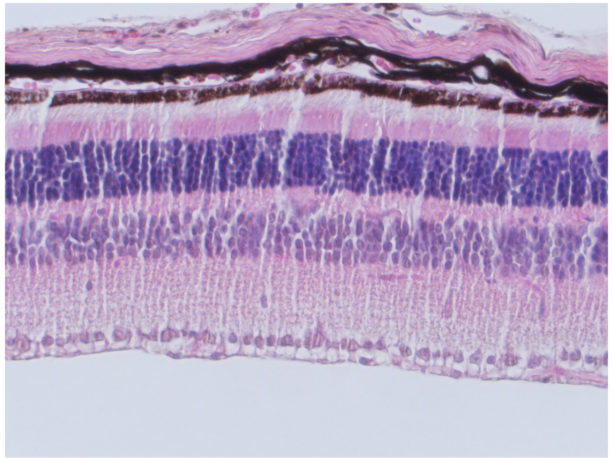

IC

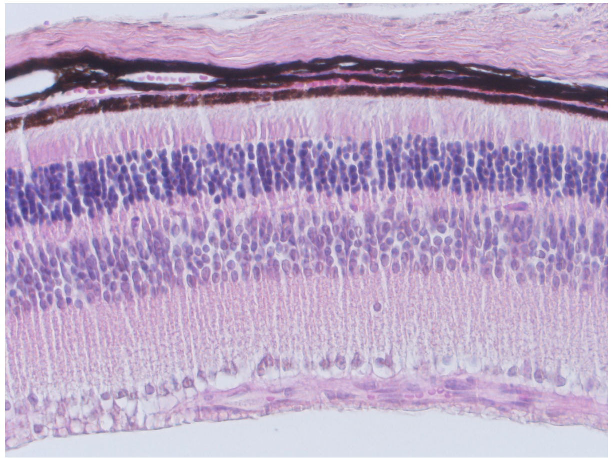

SC

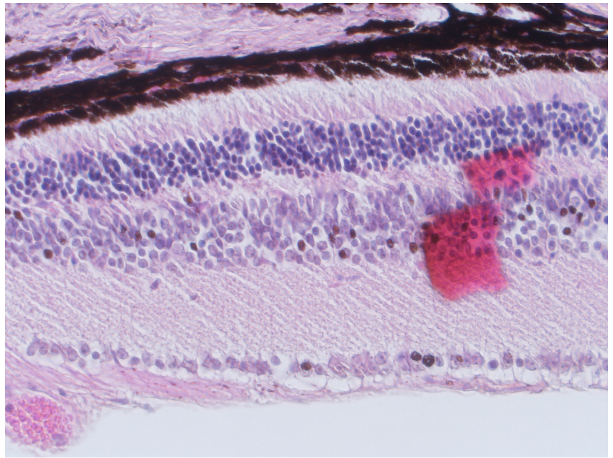

SE

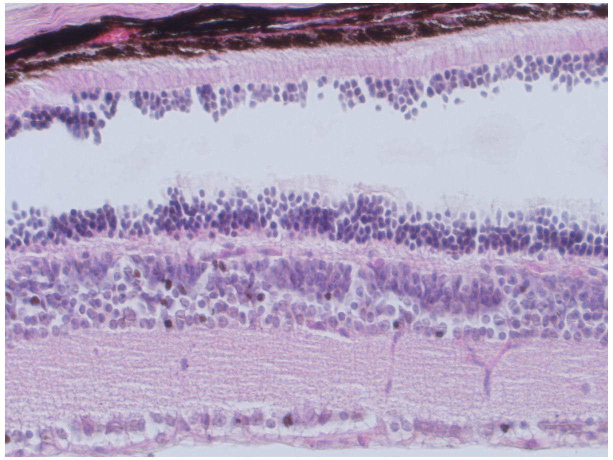

SP

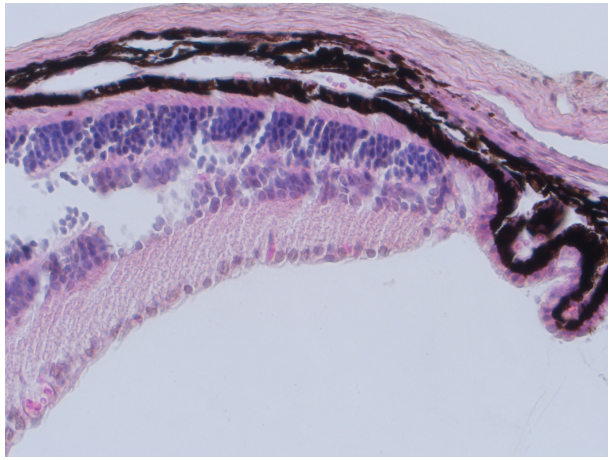

Trial5 mouse HE staining data PND38 40X—Rho$^{P23H/+}$ 8-AG-6

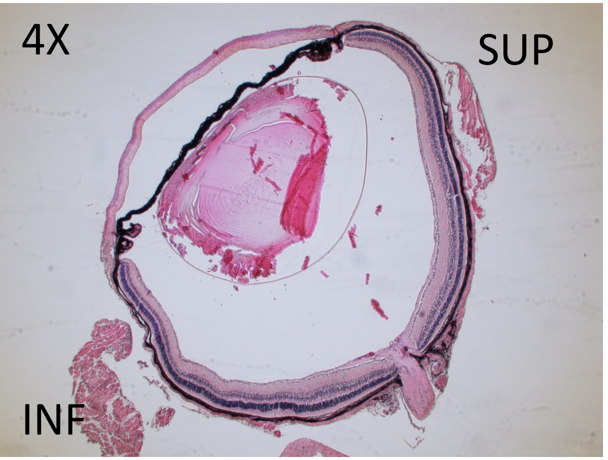

IP

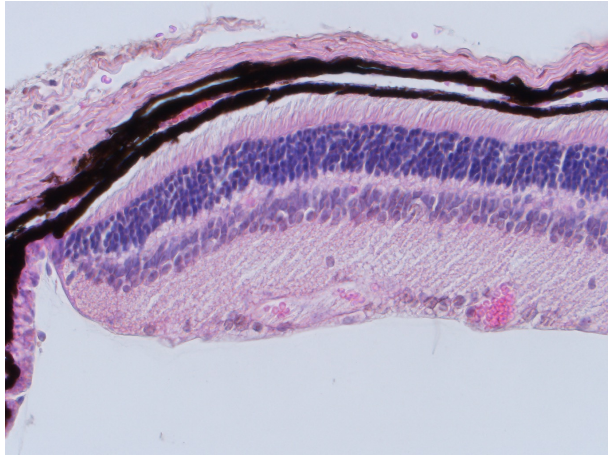

IE

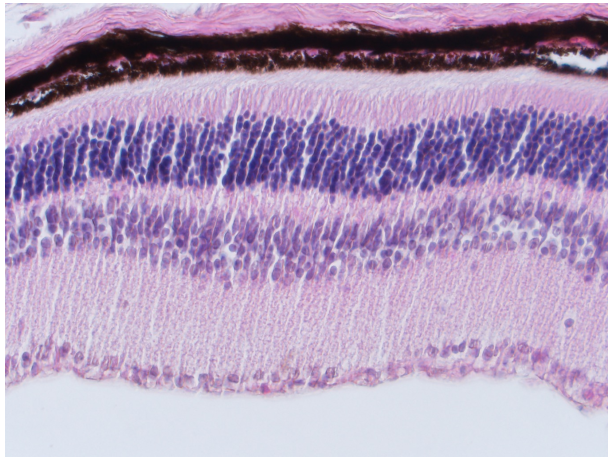

IC

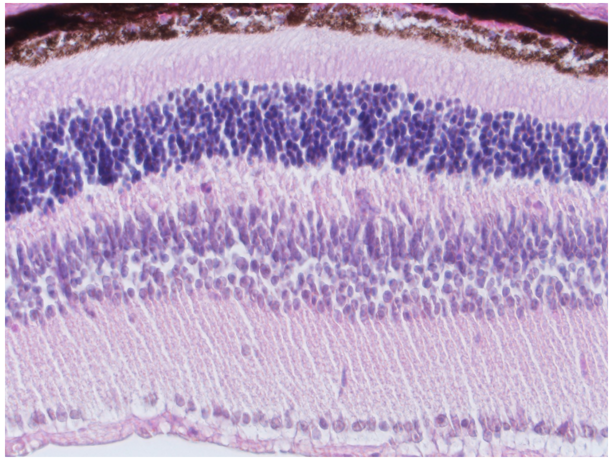

SC

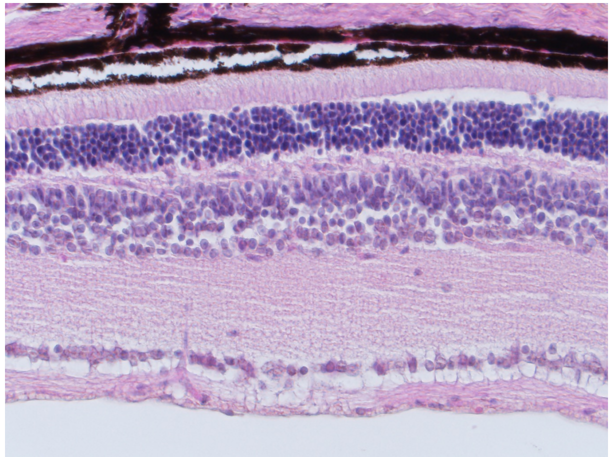

SE

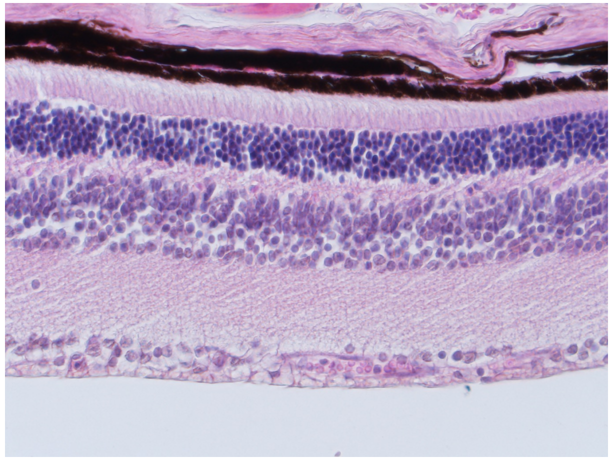

SP

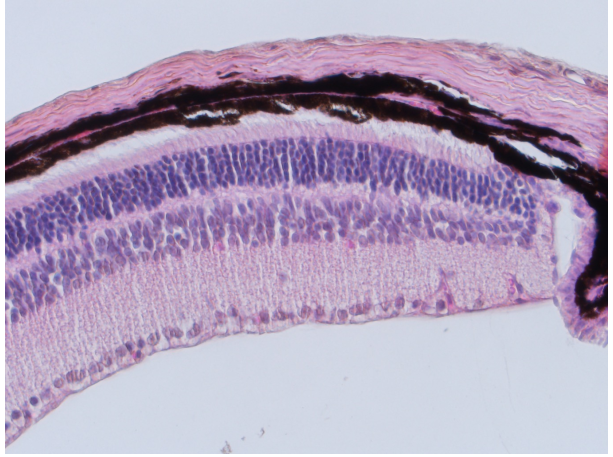

Supplement: Supplementary file 20 — Supplementary Data 18 [file 42003_2025_8242_MOESM20_ESM.pdf]
