# Supplementary material for: Oral 8-aminoguanine against age-related retinal degeneration
Source: Commun Biol. 2025 May 26;8:812. doi: 10.1038/s42003-025-08242-1 (PMC12106806; doi:10.1038/s42003-025-08242-1)

RHO staining for Figure S4

Secondary only control

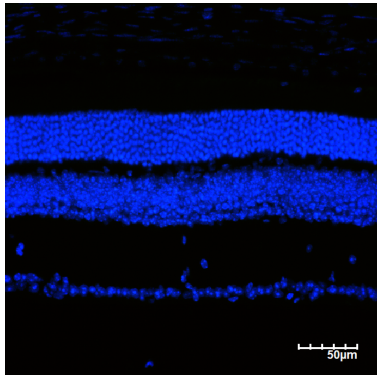

Rho$^{P23H/+}$ mouse retinae-VC(Biol. repeat 1-2)

Red, RHO; blue, Hoechst33342

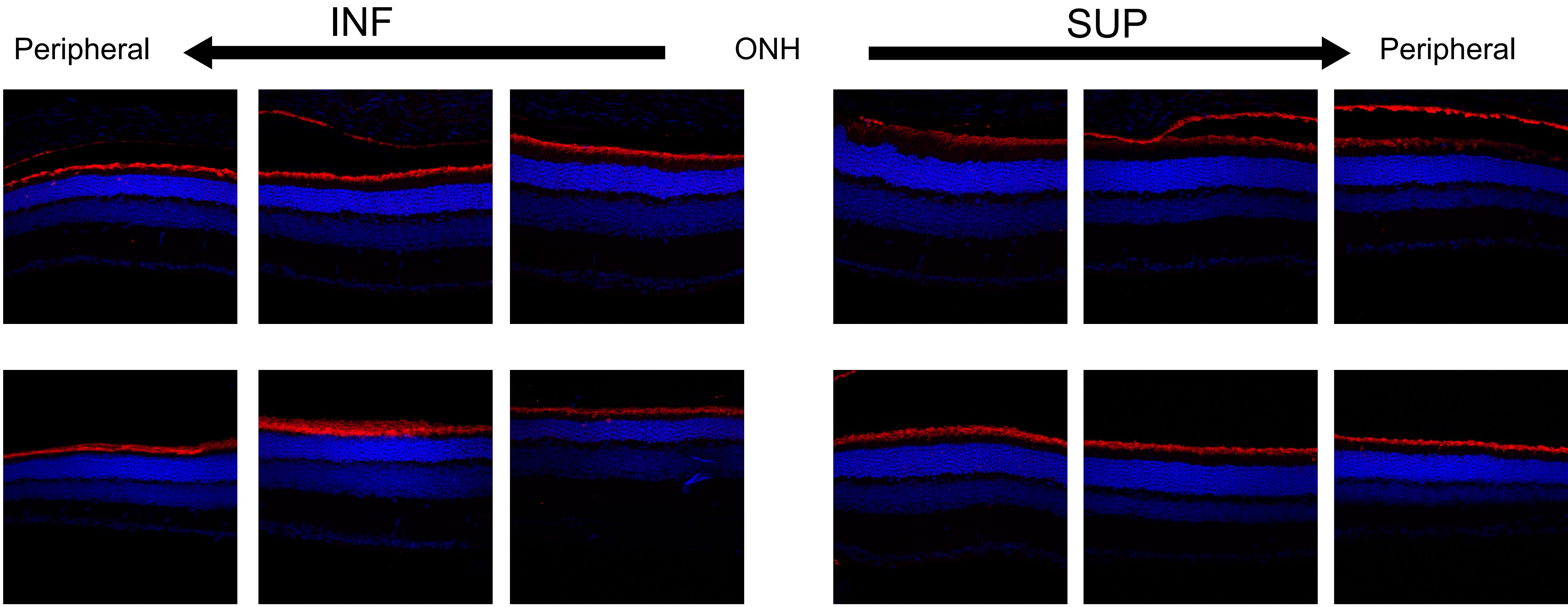

Rho$^{P23H/+}$ mouse retinae-VC(Biol. repeat 3-5)

Red, RHO; blue, Hoechst33342

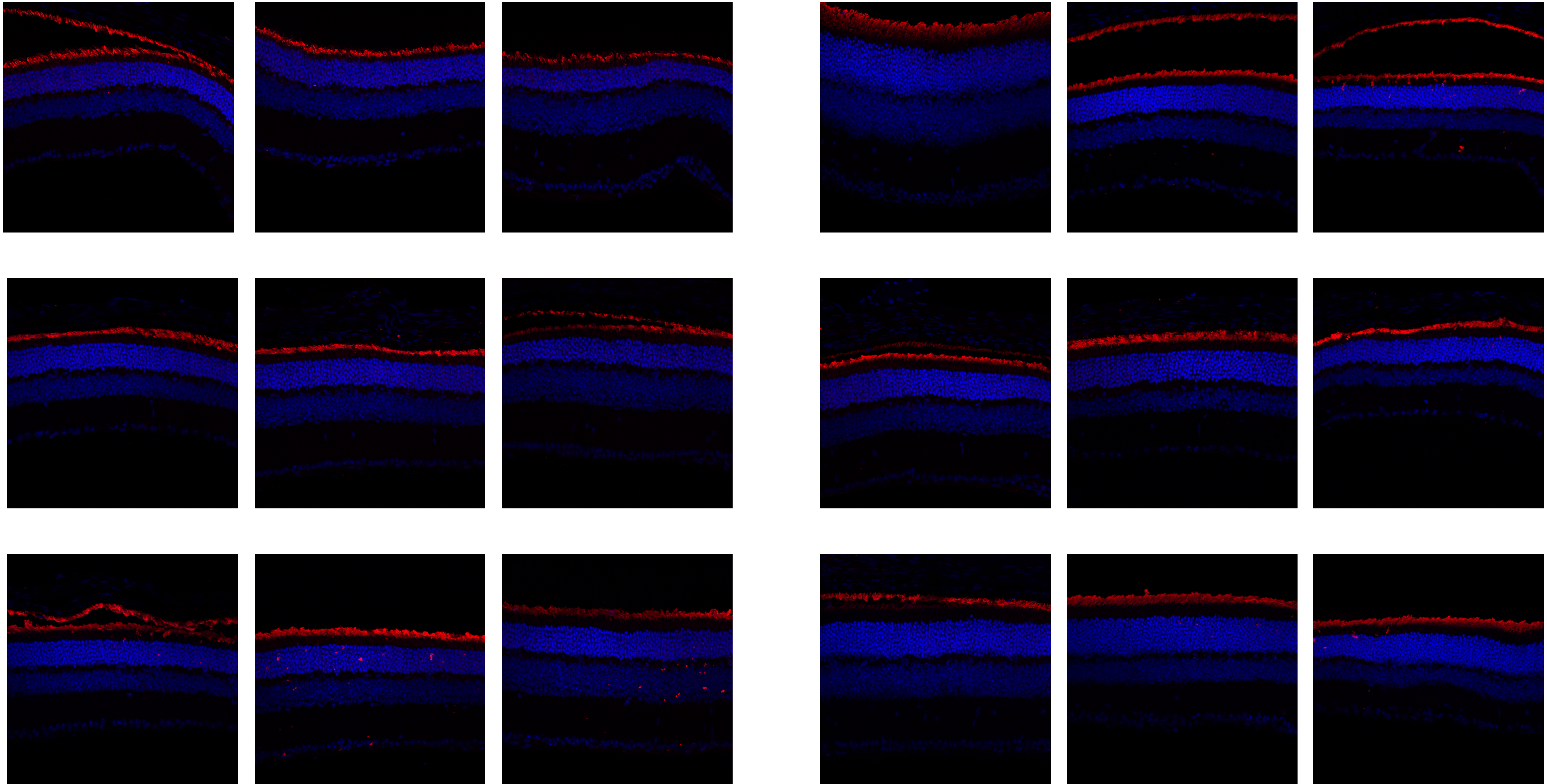

Rho$^{P23H/+}$ mouse retinae-8AG (Biol. repeat 1-2)

Red, RHO; blue, Hoechst33342

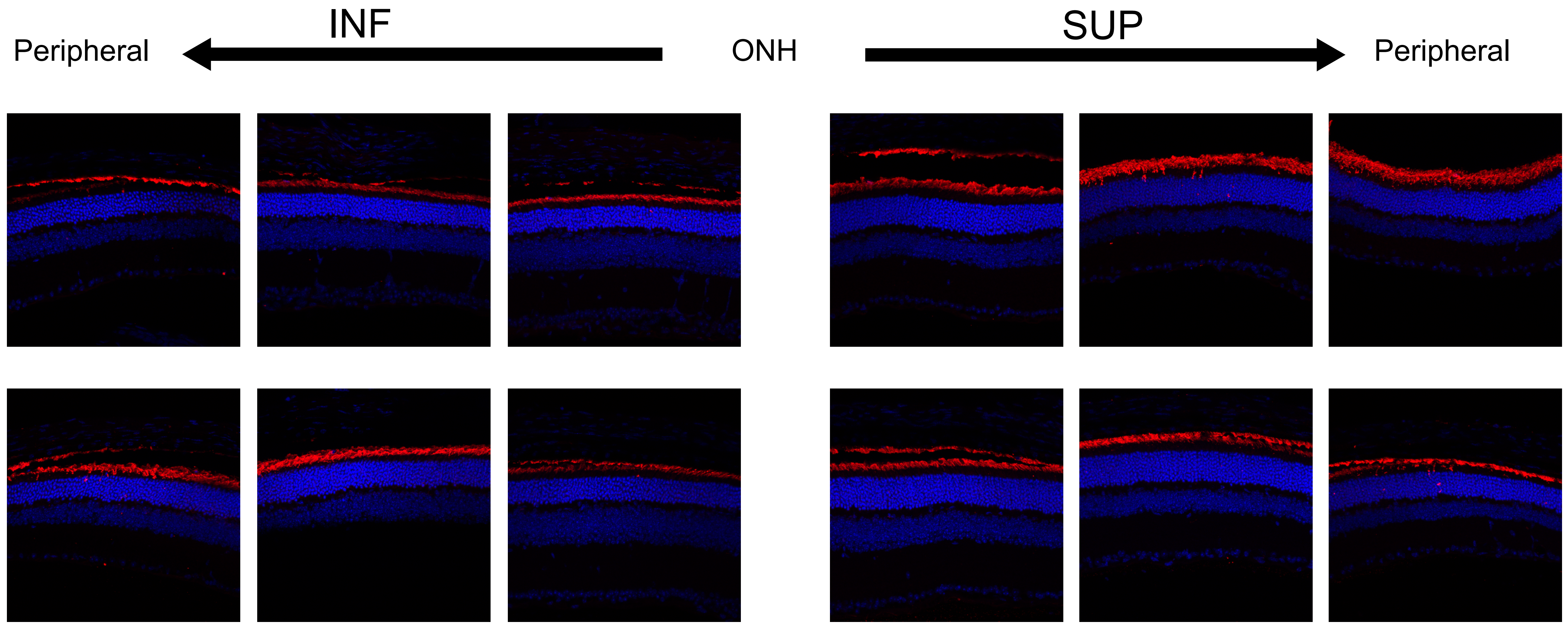

Rho$^{P23H/+}$ mouse retinae-8AG (Biol. repeat 3-5)

Red, RHO; blue, Hoechst33342

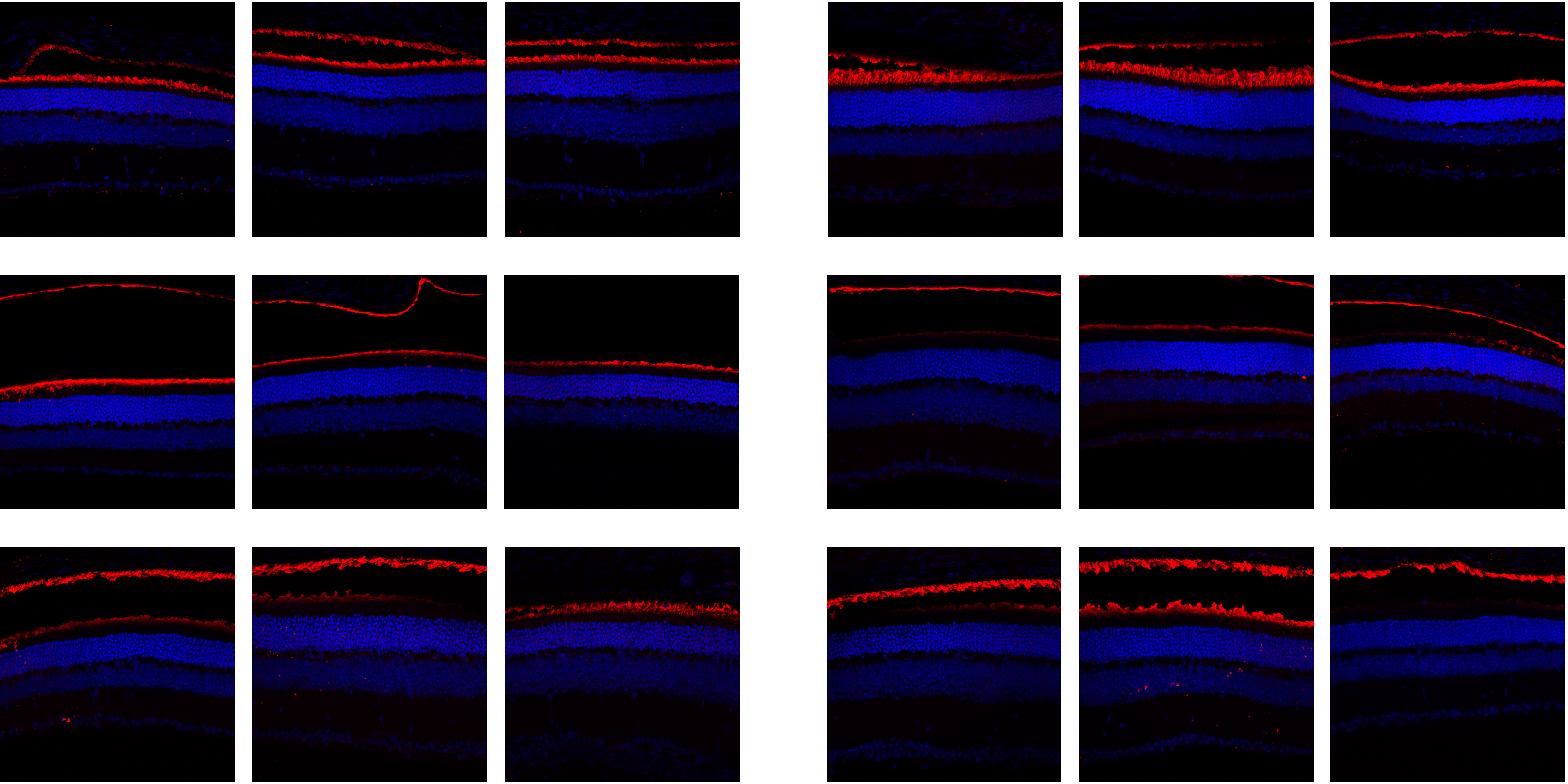

Supplement: Supplementary file 21 — Supplementary Data 19 [file 42003_2025_8242_MOESM21_ESM.pdf]
