# Supplementary material for: Oral 8-aminoguanine against age-related retinal degeneration
Source: Commun Biol. 2025 May 26;8:812. doi: 10.1038/s42003-025-08242-1 (PMC12106806; doi:10.1038/s42003-025-08242-1)

Immunofluorescence of IBA1 and CD68 on RhoP23H/+ mice for Figure S5

WT control

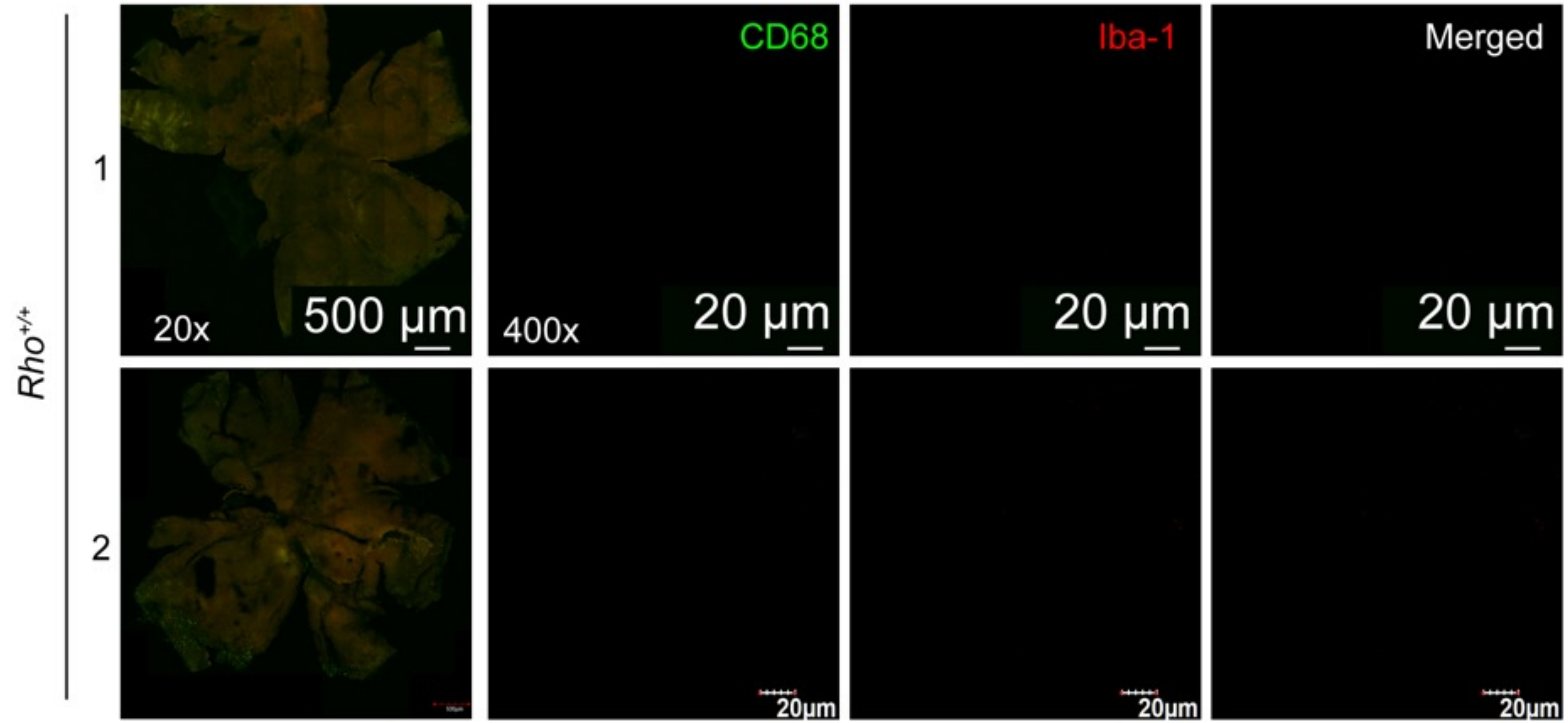
CD68
Iba-1
Merged
1
2
$Rho^{+/+}$
20x
500 µm
400x
20 µm
20 µm
20 µm
20µm
20µm
20µm

$Rho^{P23H/+}$ mouse retina treated with PBS

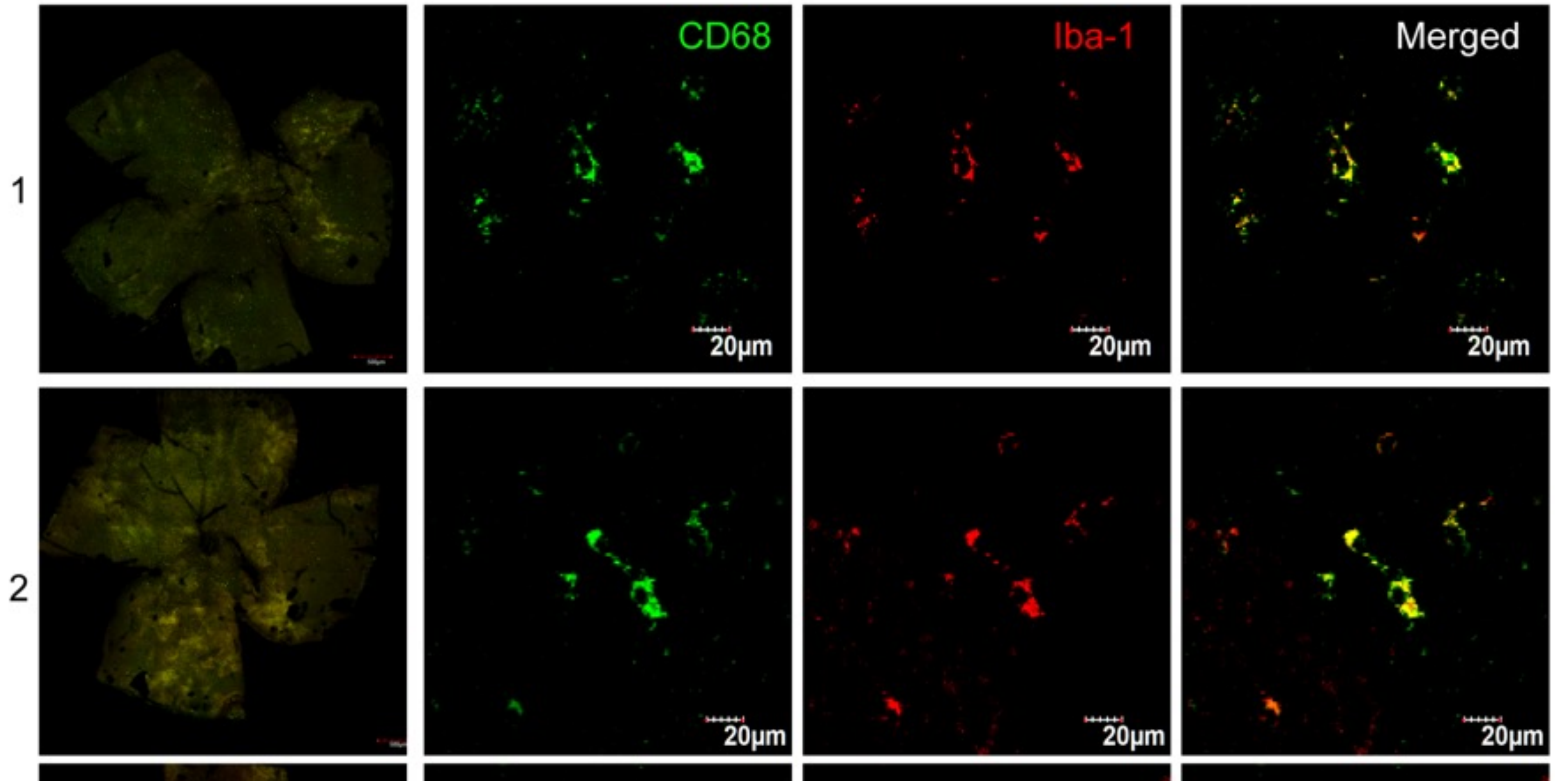

Rho$^{P23H/+}$ mouse retina treated with PBS

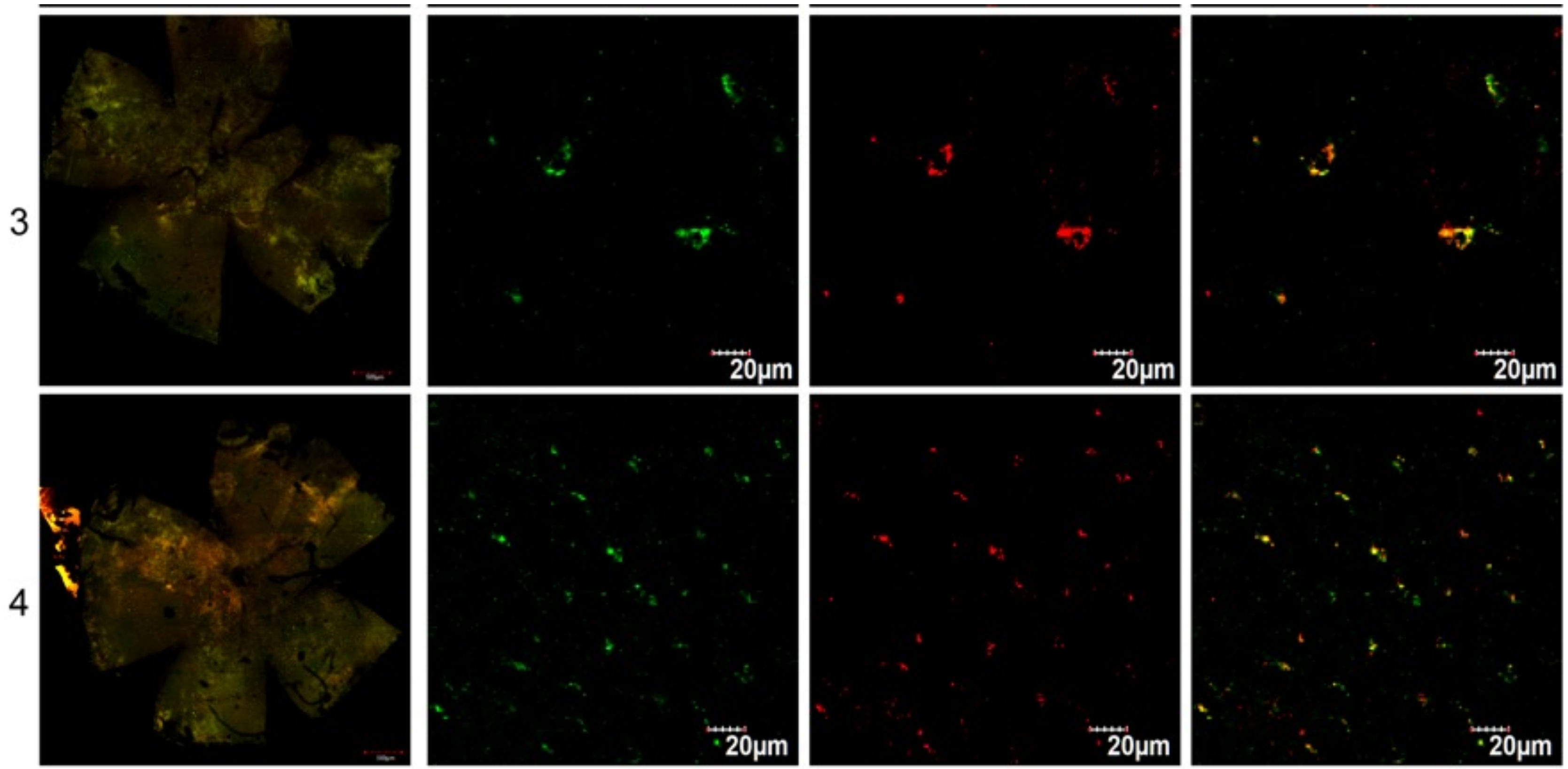

Rho$^{P23H/+}$ mouse retina treated with 8-AG

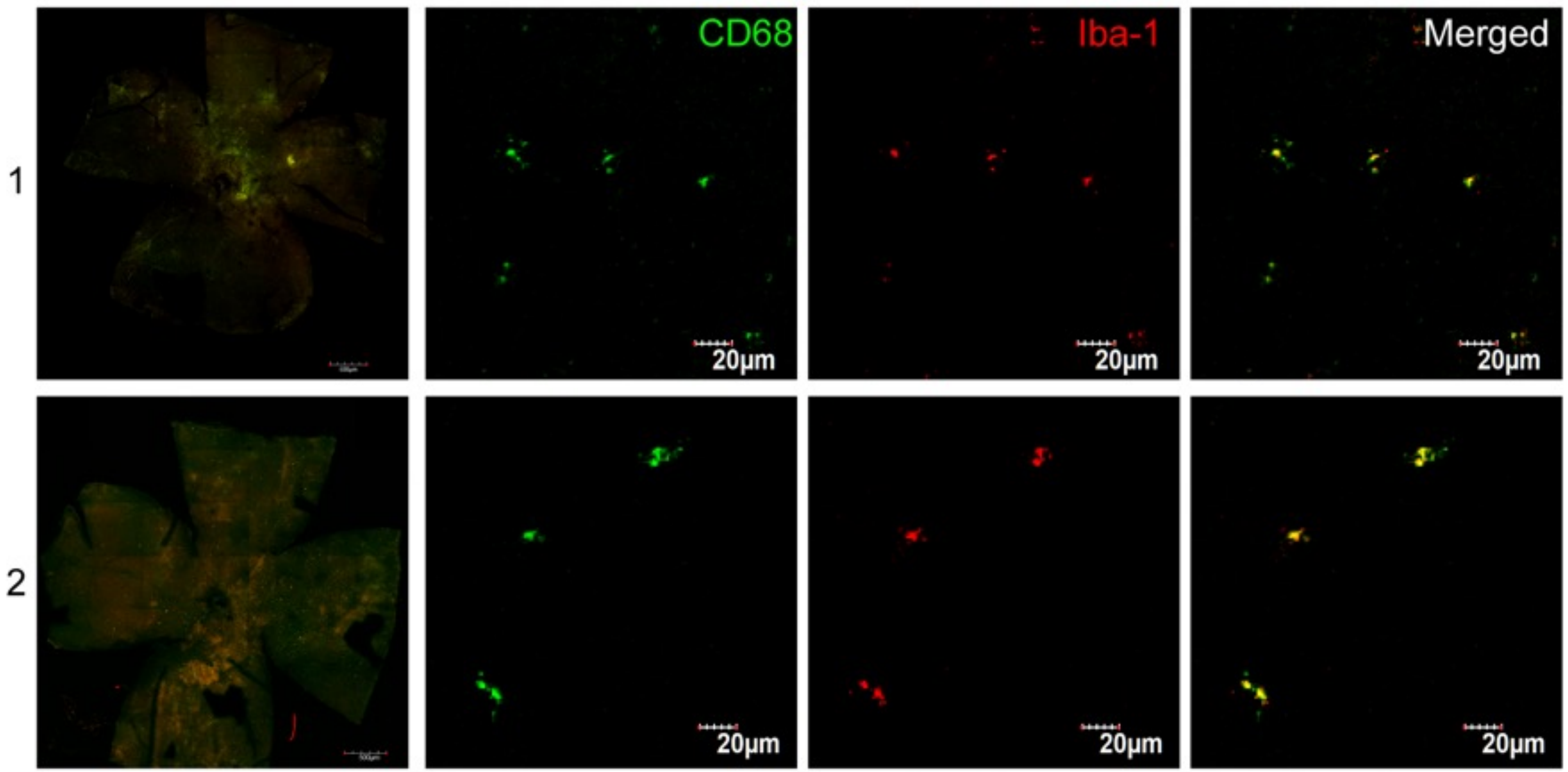

Rho$^{P23H/+}$ mouse retina treated with 8-AG

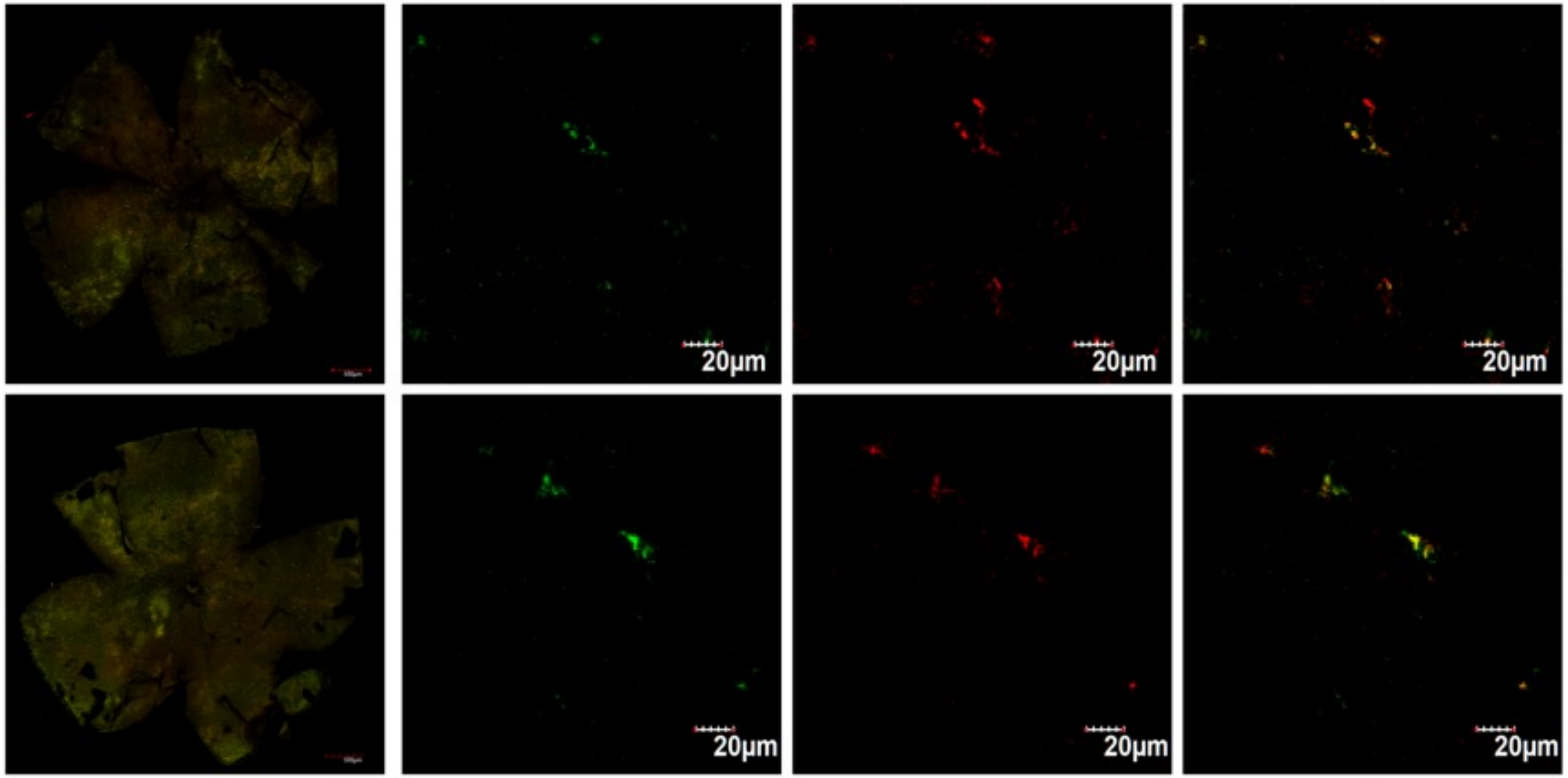

Supplement: Supplementary file 22 — Supplementary Data 20 [file 42003_2025_8242_MOESM22_ESM.pdf]
