# Supplementary material for: Oral 8-aminoguanine against age-related retinal degeneration
Source: Commun Biol. 2025 May 26;8:812. doi: 10.1038/s42003-025-08242-1 (PMC12106806; doi:10.1038/s42003-025-08242-1)

TEM-F344 rats

Peripheral retina

- Young 4 months
- Old 24 months
- Old_8-AG 24 month, (supplemented 5 mg/kg bw 8-AG in water starting from 22 months of age for 8 weeks)

Young peripheral

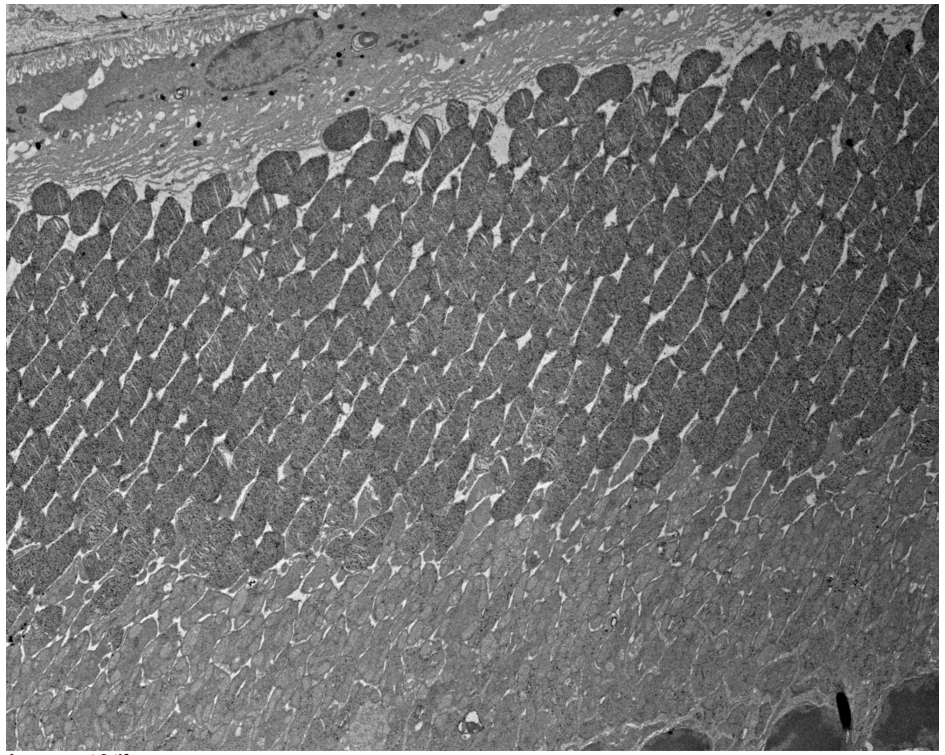

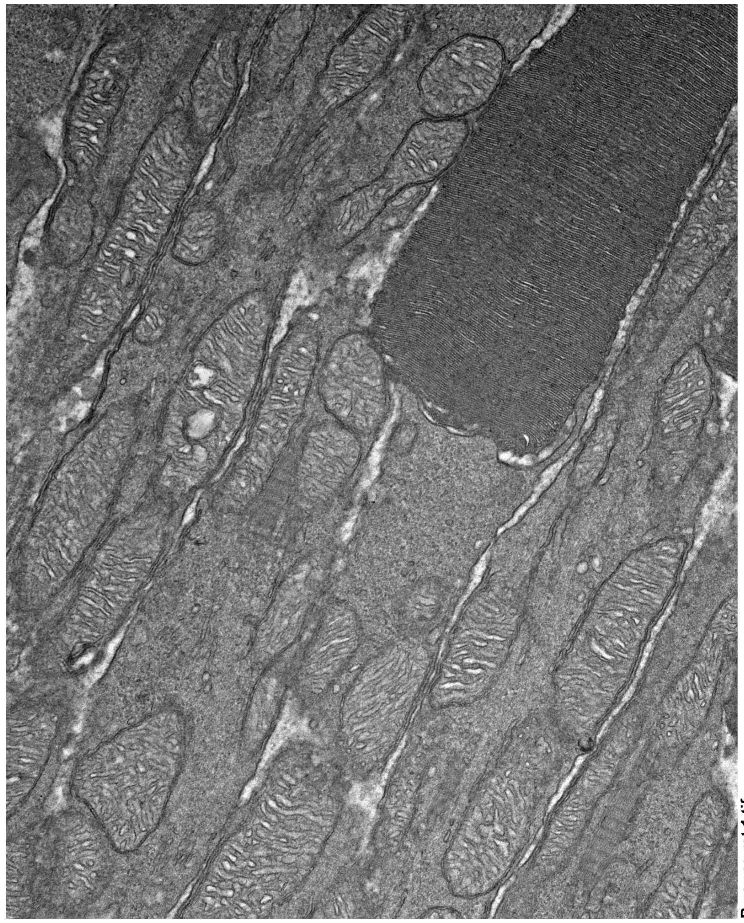

Young IS+RPE

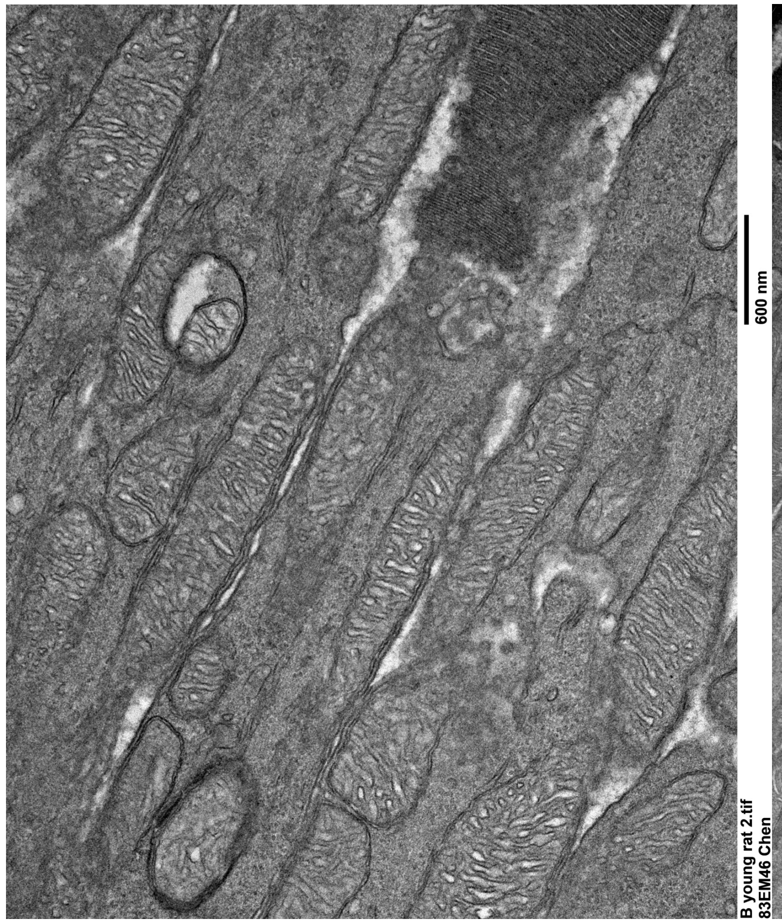
600 nm
B young rat 2.tif
83EM46 Chen

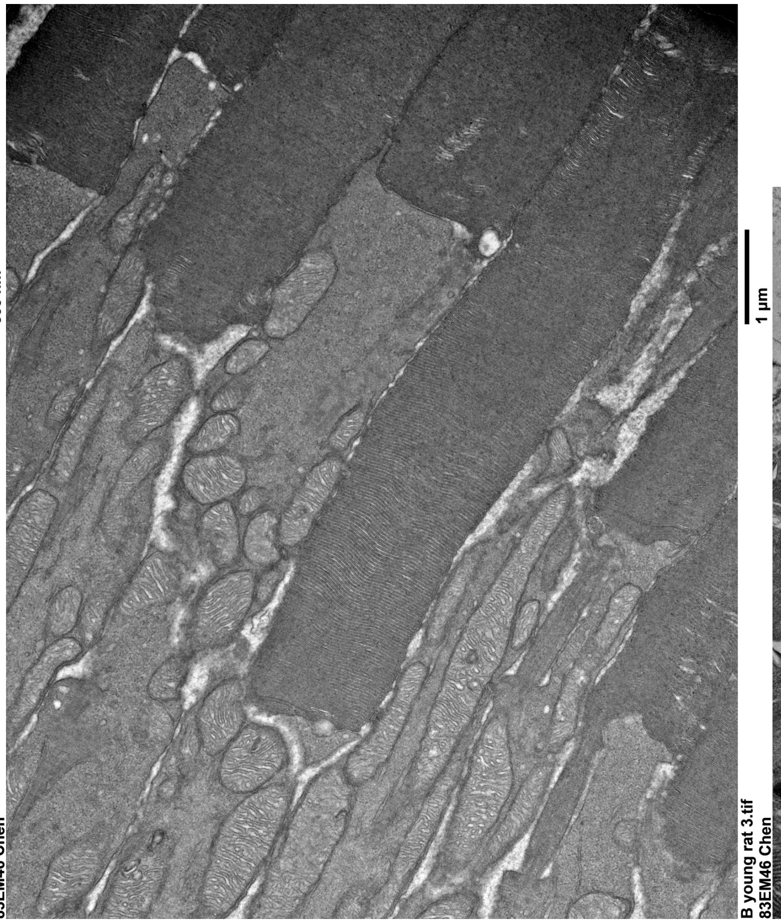
1 µm
B young rat 3.tif
83EM46 Chen

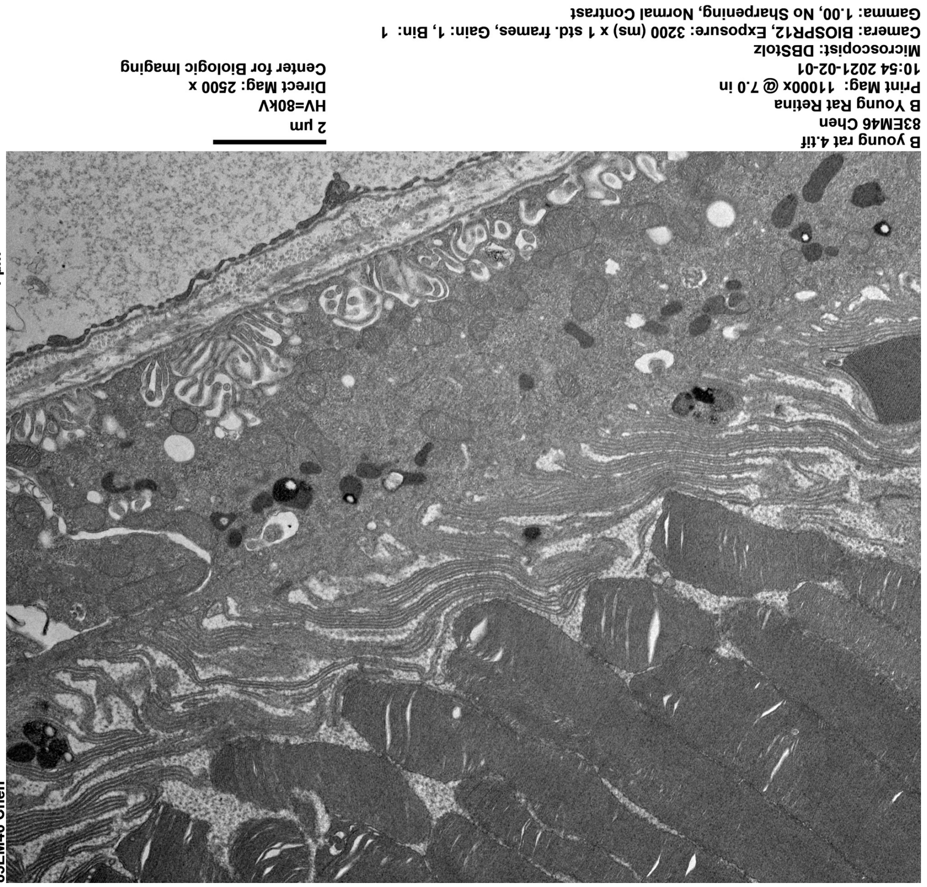
B young rat 4.tif
83EM46 Chen
B Young Rat Retina
Print Mag: 11000x @ 7.0 in
10:54 2021-02-01
Microscopist: DBStolz
Camera: BIOSPR12, Exposure: 3200 (ms) x 1 std. frames, Gain: 1, Bin: 1
Gamma: 1.00, No Sharpening, Normal Contrast
2 µm
HV=80kV
Direct Mag: 2500 x
Center for Biologic Imaging

Young RPE, IS,IS

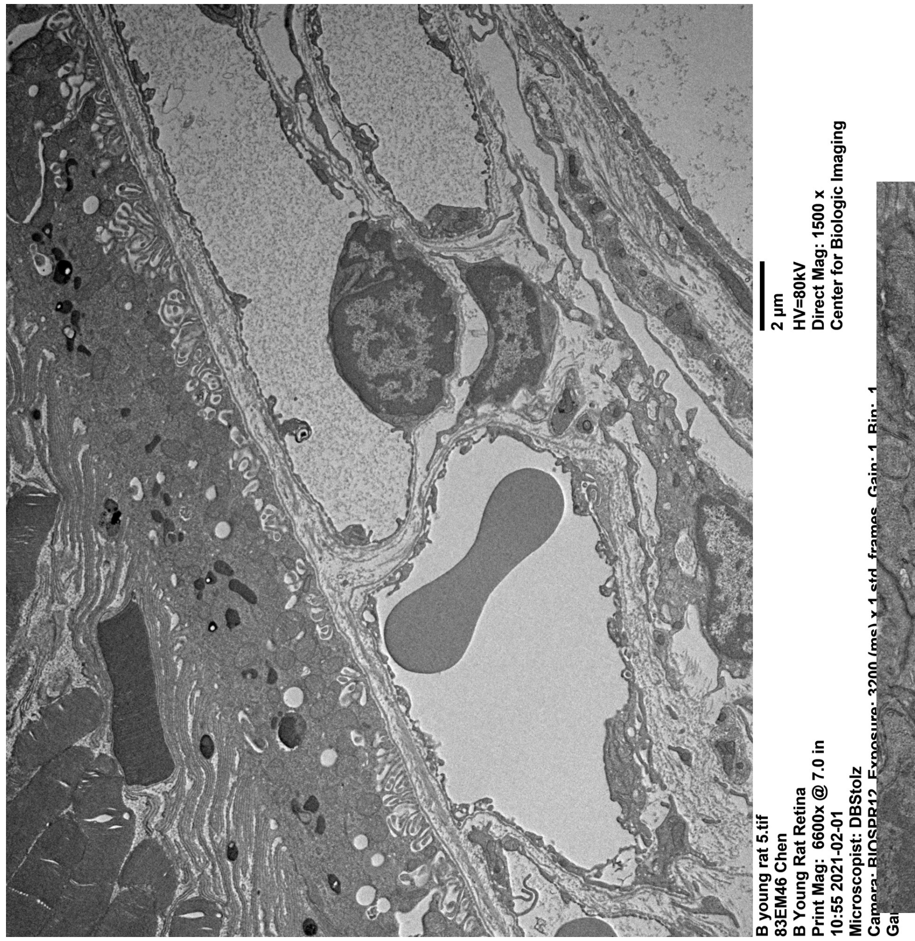

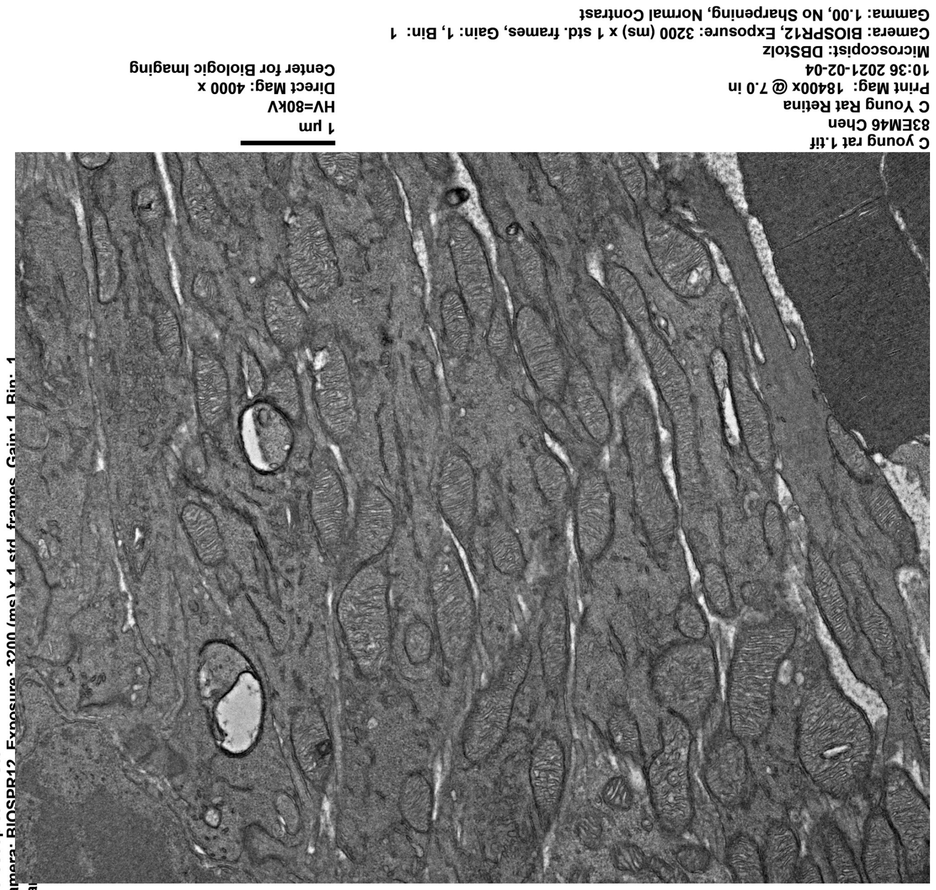

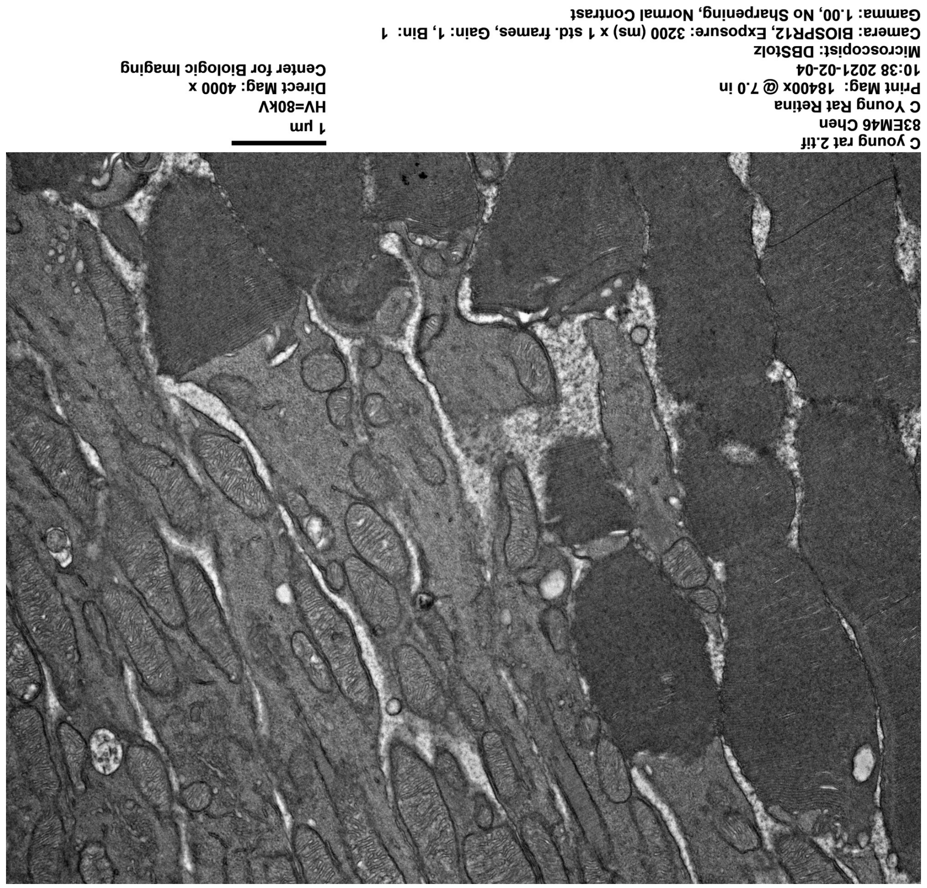

OS

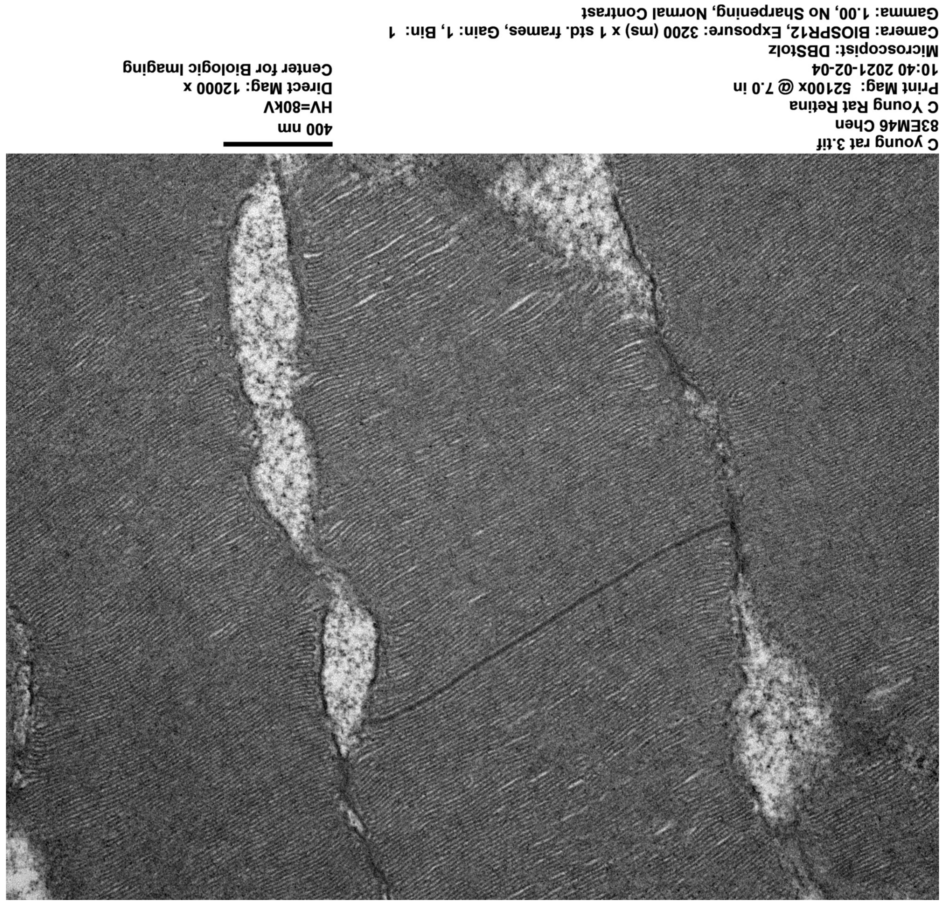
C young rat 3.tif
83EM46 Chen
C Young Rat Retina
Print Mag: 52100x @ 7.0 in
10:40 2021-02-04
Microscopist: DBStolz
Camera: BIOSPR12, Exposure: 3200 (ms) x 1 std. frames, Gain: 1, Bin: 1
Gamma: 1.00, No Sharpening, Normal Contrast
400 nm
HV=80kV
Direct Mag: 12000 x
Center for Biologic Imaging

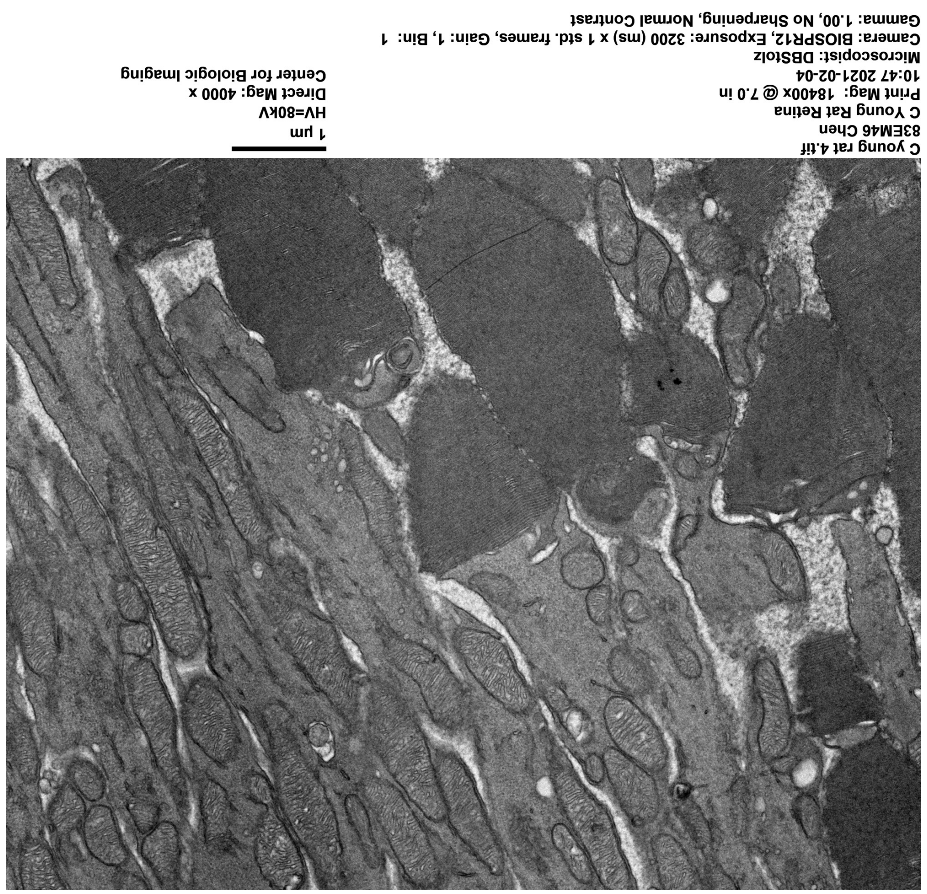
C young rat 4.tif
83EM46 Chen
C Young Rat Retina
Print Mag: 18400x @ 7.0 in
10:47 2021-02-04
Microscopist: DBStolz
Camera: BIOSPR12, Exposure: 3200 (ms) x 1 std. frames, Gain: 1, Bin: 1
Gamma: 1.00, No Sharpening, Normal Contrast
1 µm
HV=80kV
Direct Mag: 4000 x
Center for Biologic Imaging

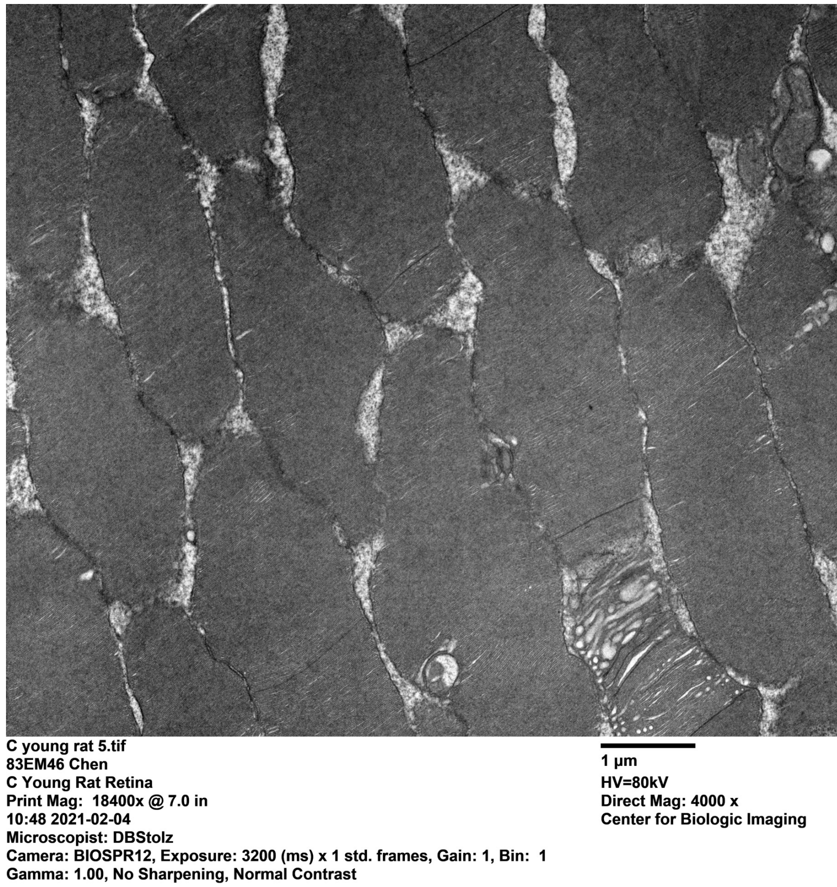
C young rat 5.tif
83EM46 Chen
C Young Rat Retina
Print Mag: 18400x @ 7.0 in
10:48 2021-02-04
Microscopist: DBStolz
Camera: BIOSPR12, Exposure: 3200 (ms) x 1 std. frames, Gain: 1, Bin: 1
Gamma: 1.00, No Sharpening, Normal Contrast
1 µm
HV=80kV
Direct Mag: 4000 x
Center for Biologic Imaging

Young OS

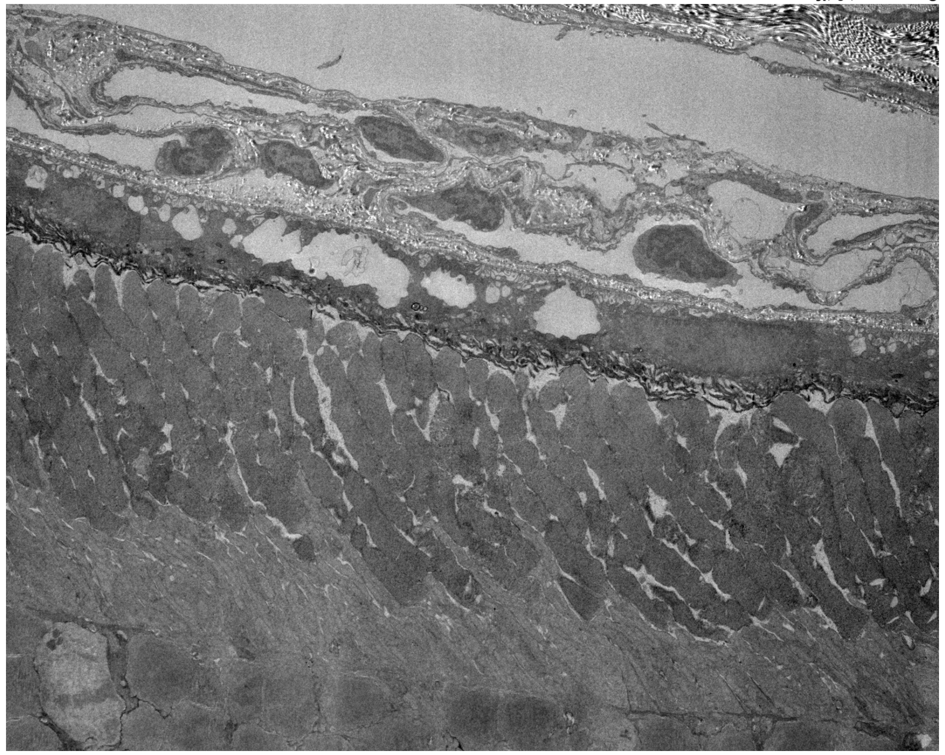

C young rat 6.tif
83EM46 Chen
C Young Rat Retina
Print Mag: 2640x @ 7.0 in
10:51 2021-02-04
Microscopist: DBStolz
Camera: BIOSPR12, Exposure: 3200 (ms) x 1 std. frames, Gain: 1, Bin: 1
Gamma: 1.00, No Sharpening, Normal Contrast

8 µm
HV=80kV
Direct Mag: 600 x
Center for Biologic Imaging

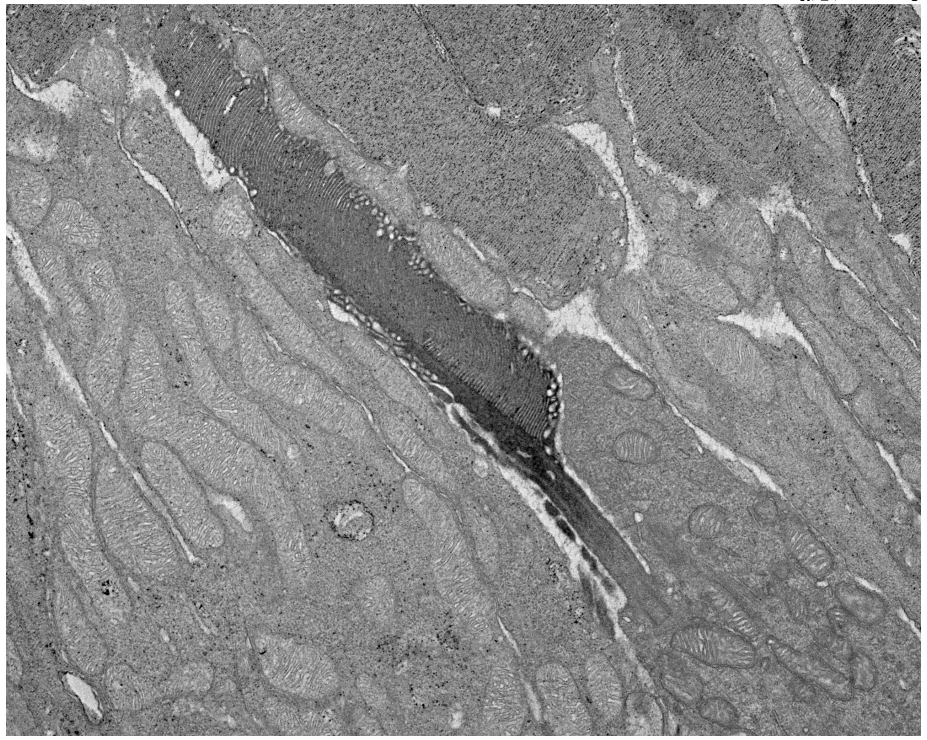

C young rat 7.tif
83EM46 Chen
C Young Rat Retina
Print Mag: 18400x @ 7.0 in
10:53 2021-02-04
Microscopist: DBStolz
Camera: BIOSPR12, Exposure: 3200 (ms) x 1 std. frames, Gain: 1, Bin: 1
Gamma: 1.00, No Sharpening, Normal Contrast

1 µm
HV=80kV
Direct Mag: 4000 x
Center for Biologic Imaging

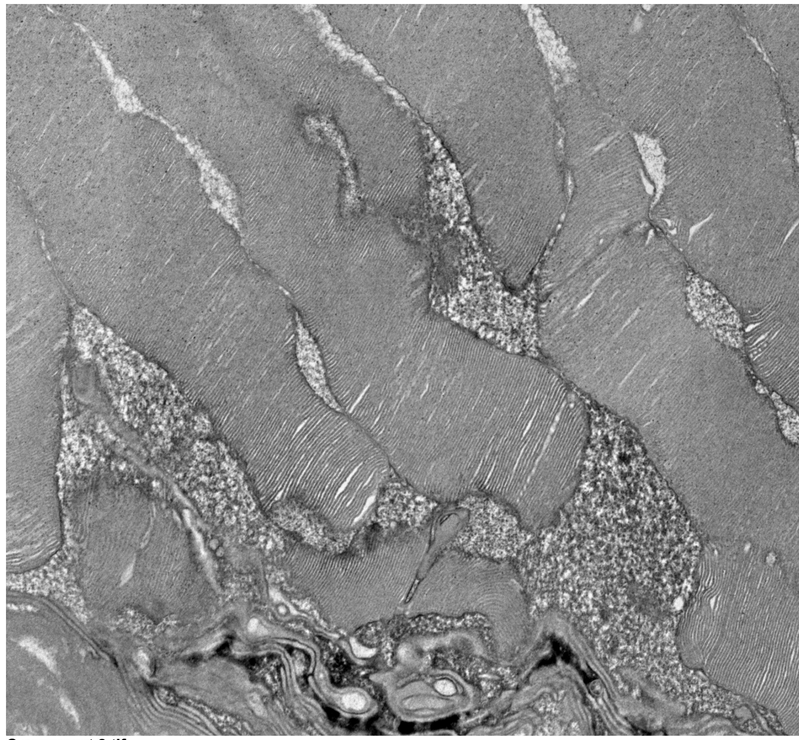

C young rat 8.tif
83EM46 Chen
C Young Rat Retina
Print Mag: 18400x @ 7.0 in
10:55 2021-02-04
Microscopist: DBStolz
Camera: BIOSPR12, Exposure: 3200 (ms) x 1 std. frames, Gain: 1, Bin: 1
Gamma: 1.00, No Sharpening, Normal Contrast

1 µm
HV=80kV
Direct Mag: 4000 x
Center for Biologic Imagin

Young OS/IS

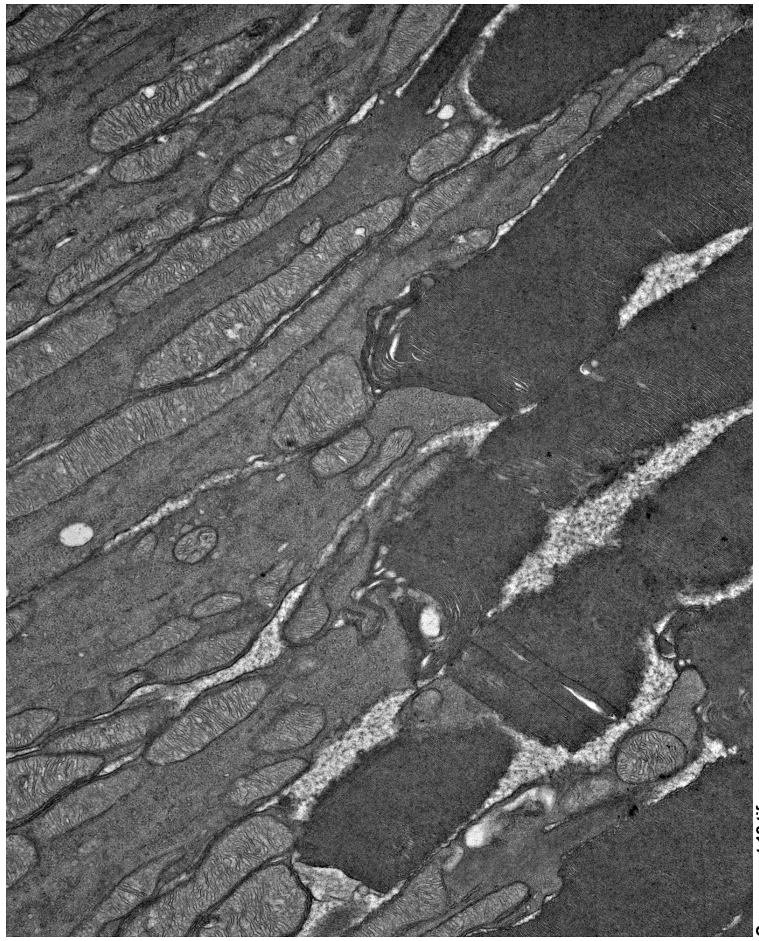

1 µm
HV=80kV
Direct Mag: 4000 x
C young rat 12.tif
83EM46 Chen
C Young Rat Retina
Print Mag: 18400x @ 7.0 in

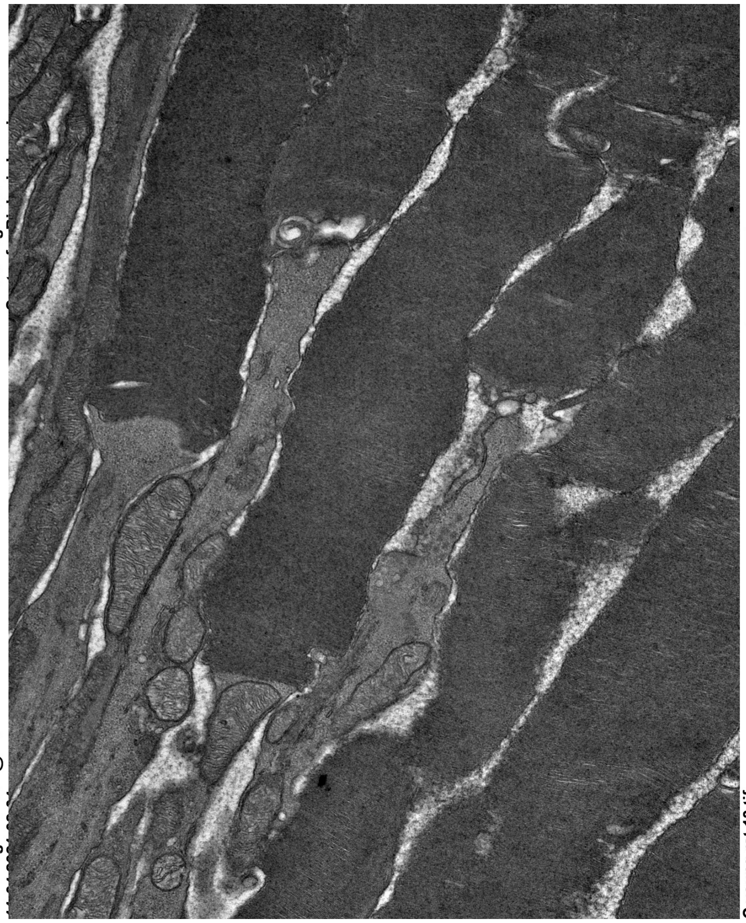

1 µm
HV=80kV
Direct Mag: 4000 x
Center for Biologic Imaging
C young rat 13.tif
83EM46 Chen
C Young Rat Retina
Print Mag: 18400x @ 7.0 in
11:06 2021-02-04
Microscopist: DBStolz
Camera: BIOSPR12, Exposure: 3200 (ms) x 1 std. frames, Gain: 1, Bin: 1
Gamma: 1.00, No Sharpening, Normal Contrast

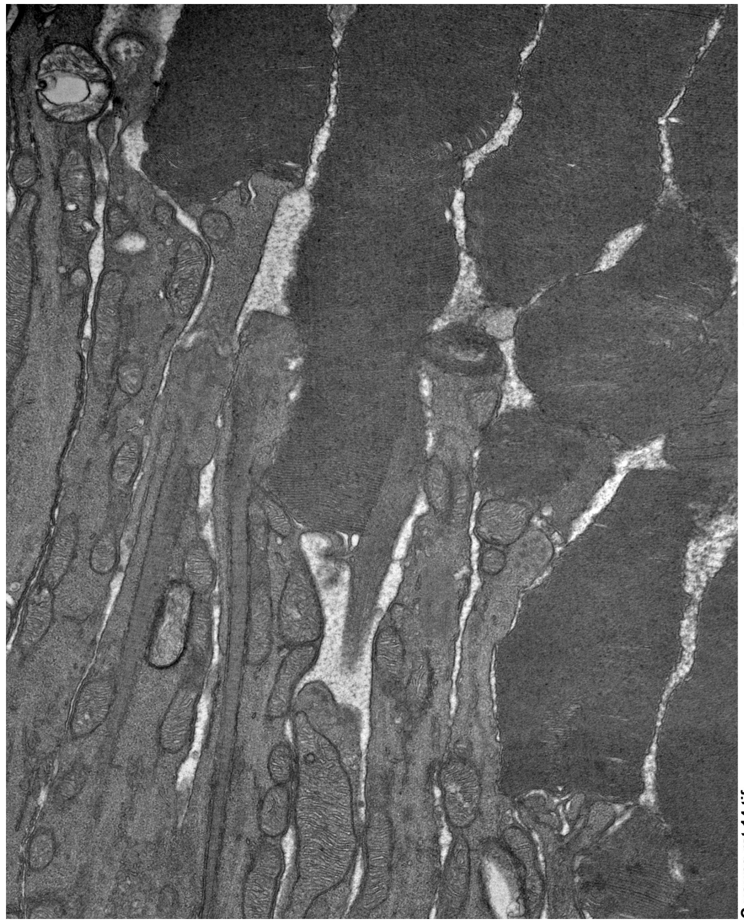

1 µm
HV=80kV
Direct Mag: 4000 x
Center for Biologic Imaging
C young rat 14.tif
83EM46 Chen
C Young Rat Retina
Print Mag: 18400x @ 7.0 in
11:07 2021-02-04

Young Chroid/RPE, IS

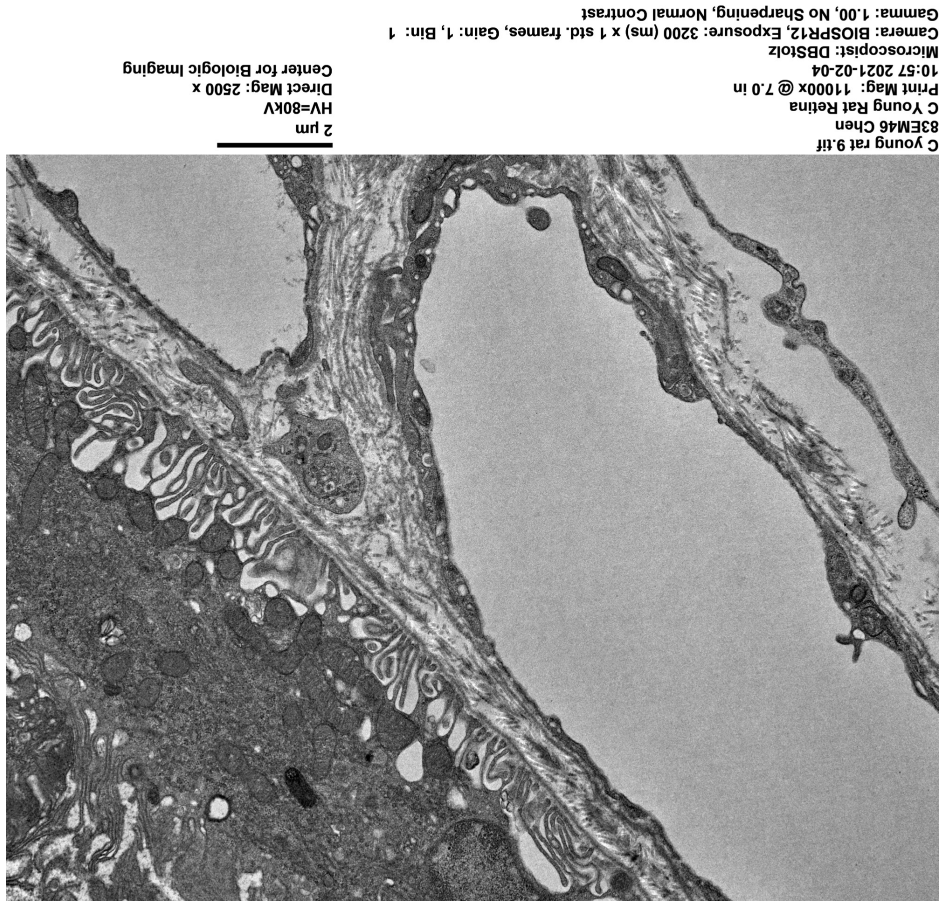

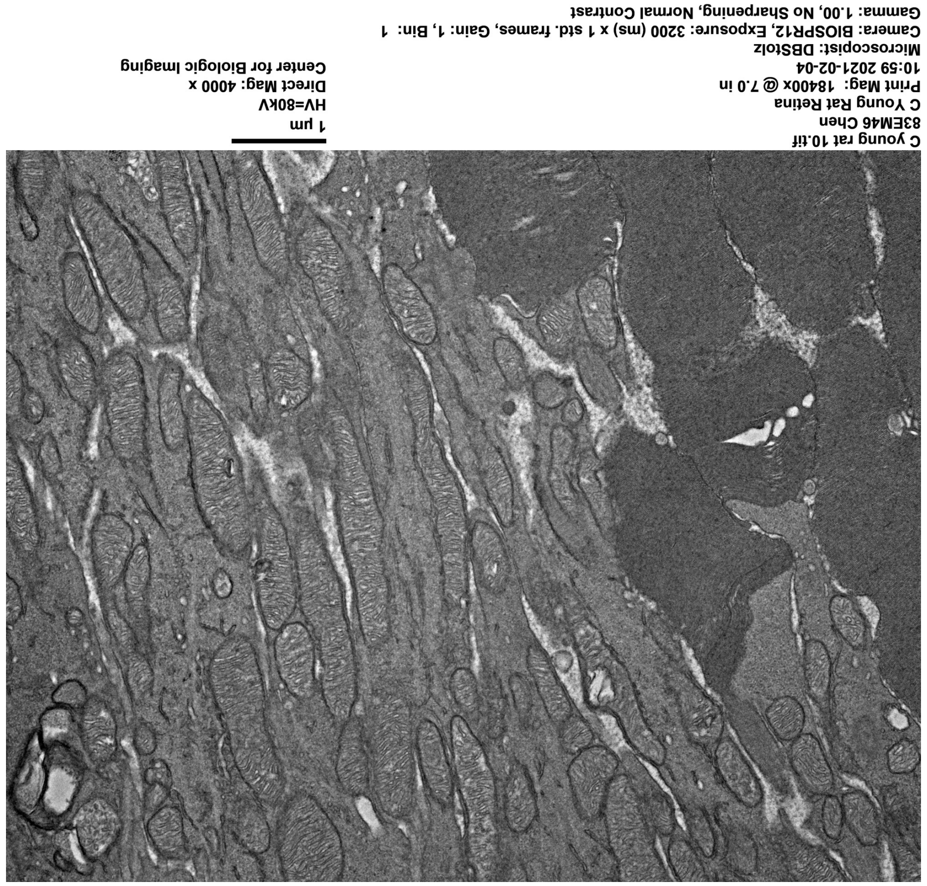

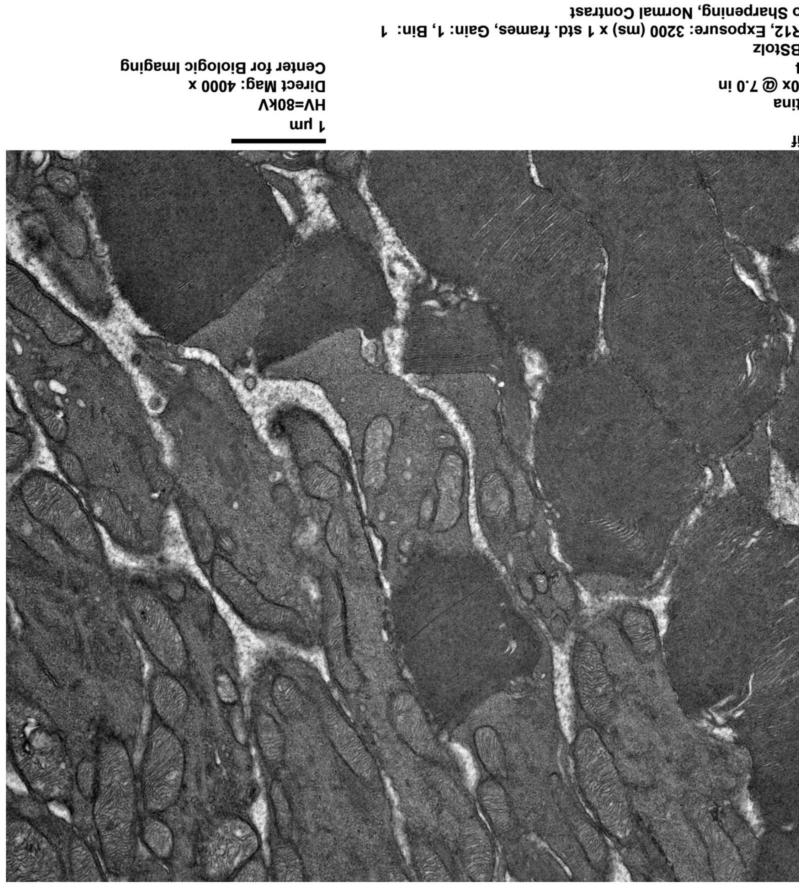

Young RPE

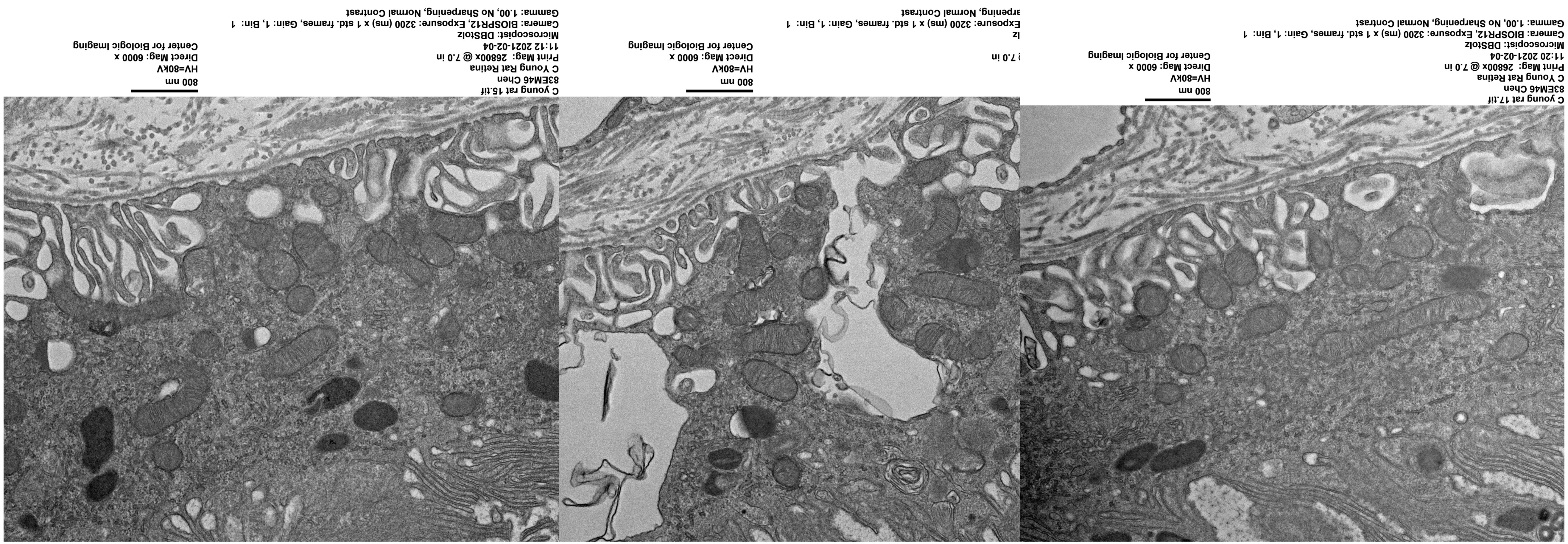

C young rat 15.tif
83EM46 Chen
C Young Rat Retina
Print Mag: 26800x @ 7.0 in
11:12 2021-02-04
Microscopist: DBStolz
Camera: BIOSPR12, Exposure: 3200 (ms) x 1 std. frames, Gain: 1, Bin: 1
Gamma: 1.00, No Sharpening, Normal Contrast
800 nm
HV=80kV
Direct Mag: 6000 x
Center for Biologic Imaging
800 nm
HV=80kV
Direct Mag: 6000 x
Center for Biologic Imaging
C young rat 17.tif
83EM46 Chen
C Young Rat Retina
Print Mag: 26800x @ 7.0 in
11:20 2021-02-04
Microscopist: DBStolz
Camera: BIOSPR12, Exposure: 3200 (ms) x 1 std. frames, Gain: 1, Bin: 1
Gamma: 1.00, No Sharpening, Normal Contrast
800 nm
HV=80kV
Direct Mag: 6000 x
Center for Biologic Imaging

Young RPE

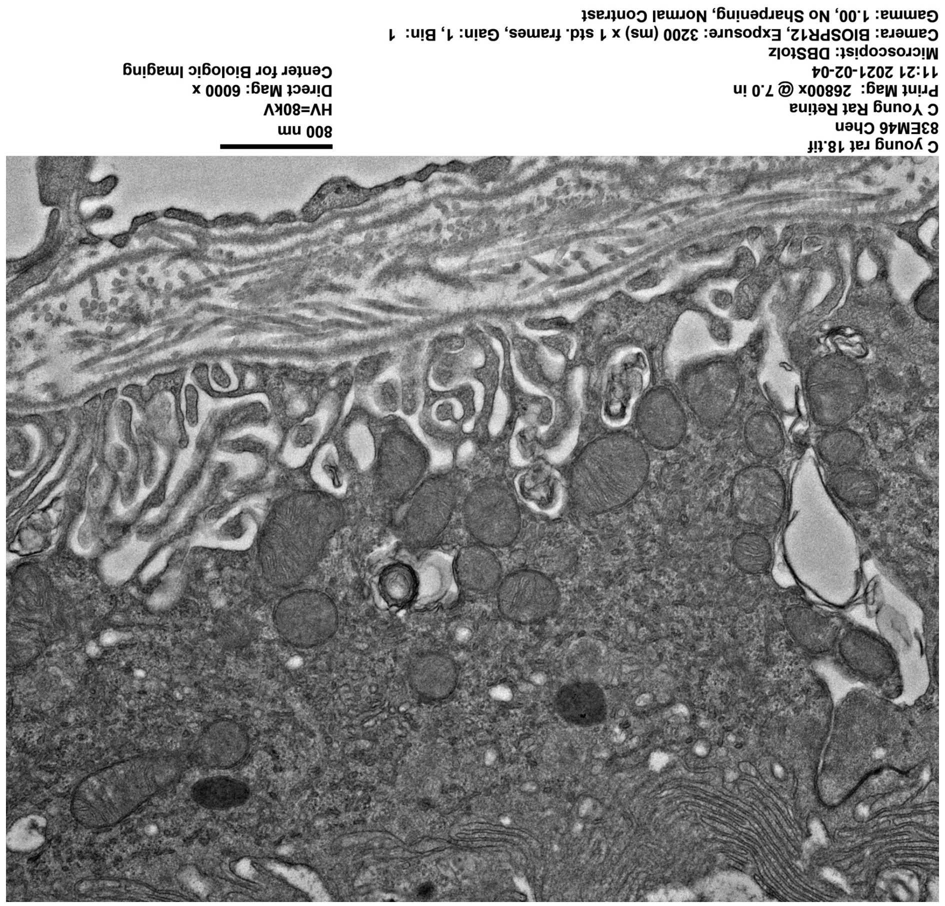
C young rat 18.tif
83EM46 Chen
C Young Rat Retina
Print Mag: 26800x @ 7.0 in
11:21 2021-02-04
Microscopist: DBStolz
Camera: BIOSPR12, Exposure: 3200 (ms) x 1 std. frames, Gain: 1, Bin: 1
Gamma: 1.00, No Sharpening, Normal Contrast
800 nm
HV=80kV
Direct Mag: 6000 x
Center for Biologic Imaging

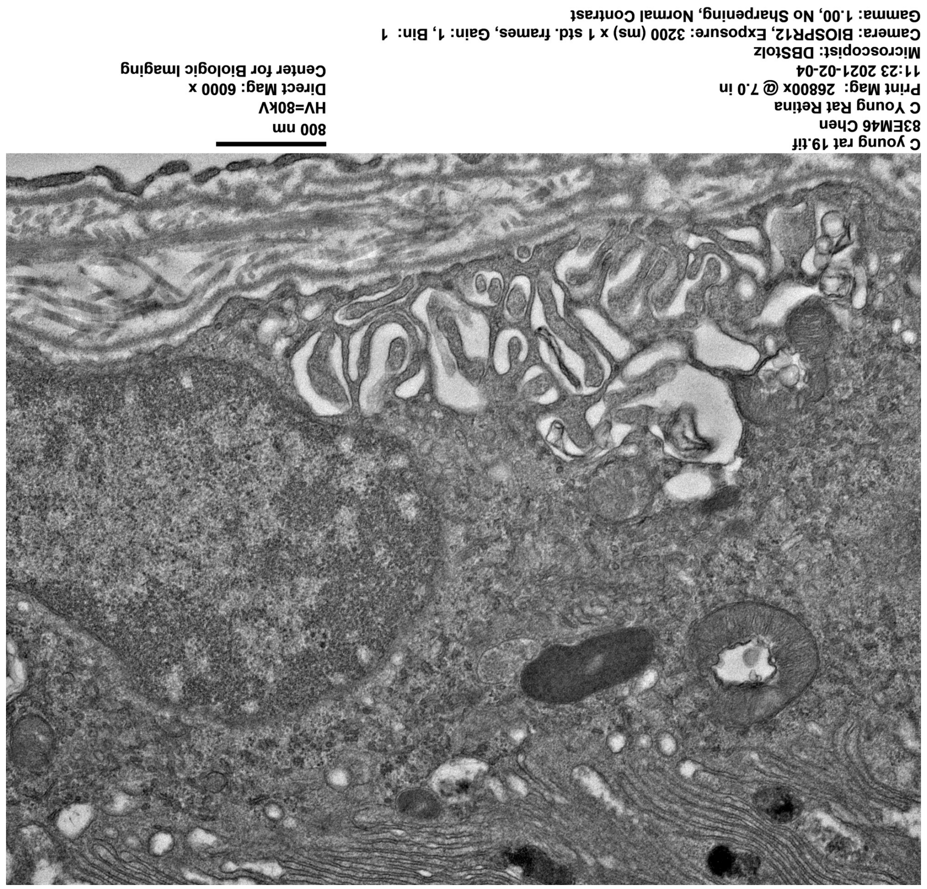
C young rat 19.tif
83EM46 Chen
C Young Rat Retina
Print Mag: 26800x @ 7.0 in
11:23 2021-02-04
Microscopist: DBStolz
Camera: BIOSPR12, Exposure: 3200 (ms) x 1 std. frames, Gain: 1, Bin: 1
Gamma: 1.00, No Sharpening, Normal Contrast
800 nm
HV=80kV
Direct Mag: 6000 x
Center for Biologic Imaging

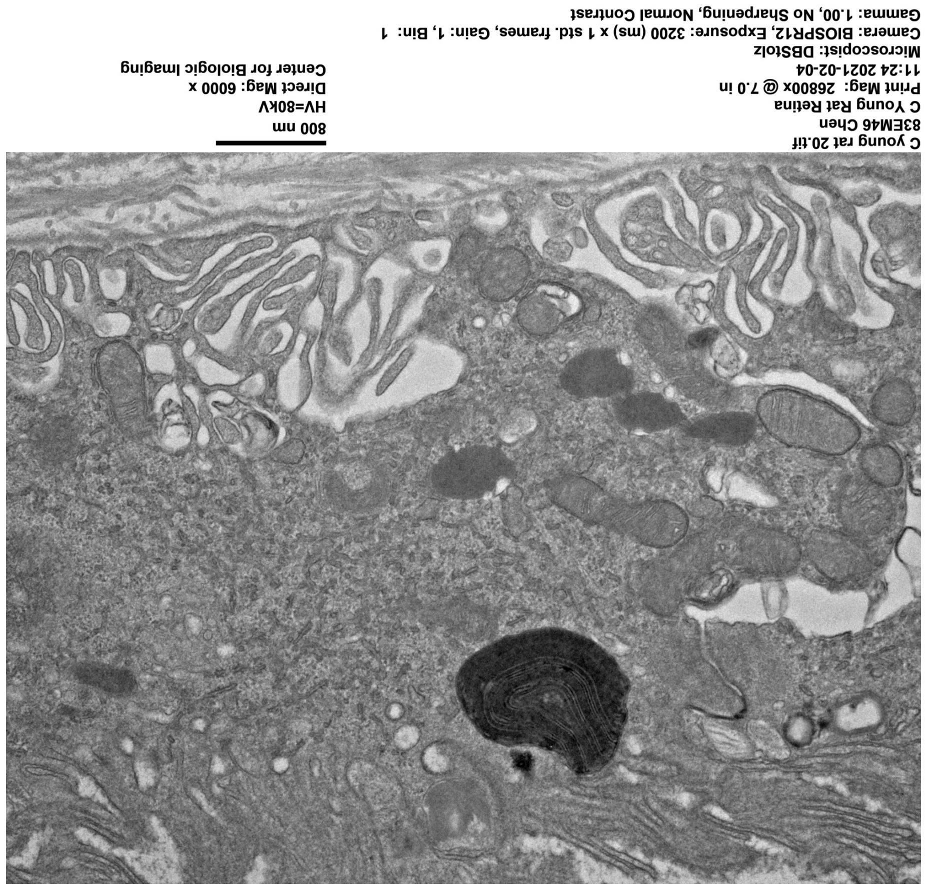
C young rat 20.tif
83EM46 Chen
C Young Rat Retina
Print Mag: 26800x @ 7.0 in
11:24 2021-02-04
Microscopist: DBStolz
Camera: BIOSPR12, Exposure: 3200 (ms) x 1 std. frames, Gain: 1, Bin: 1
Gamma: 1.00, No Sharpening, Normal Contrast
800 nm
HV=80kV
Direct Mag: 6000 x
Center for Biologic Imaging

Young IS, RPE, INL

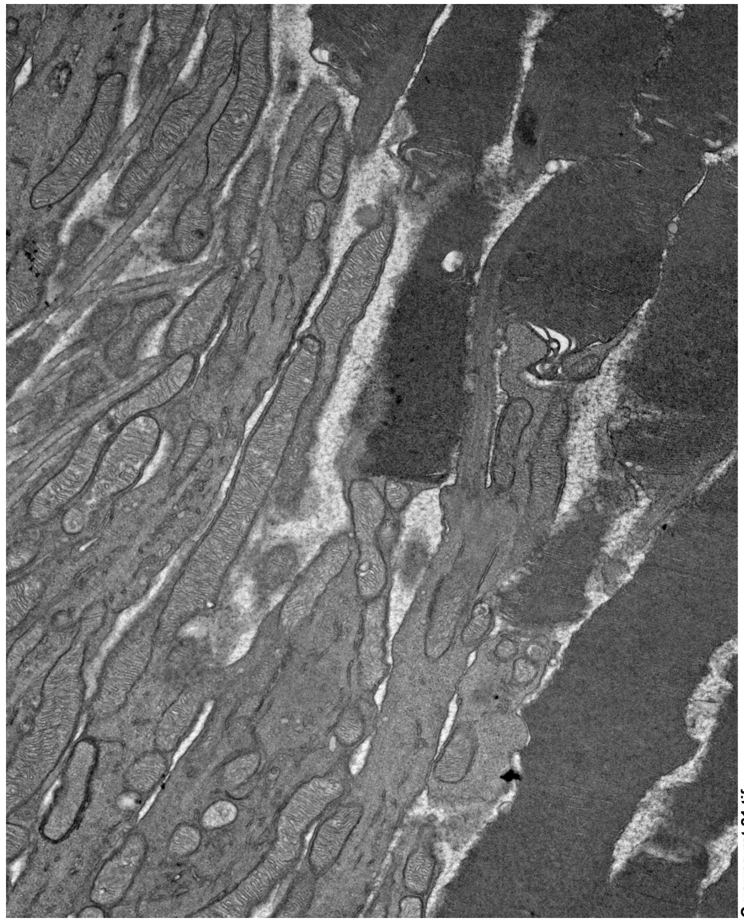

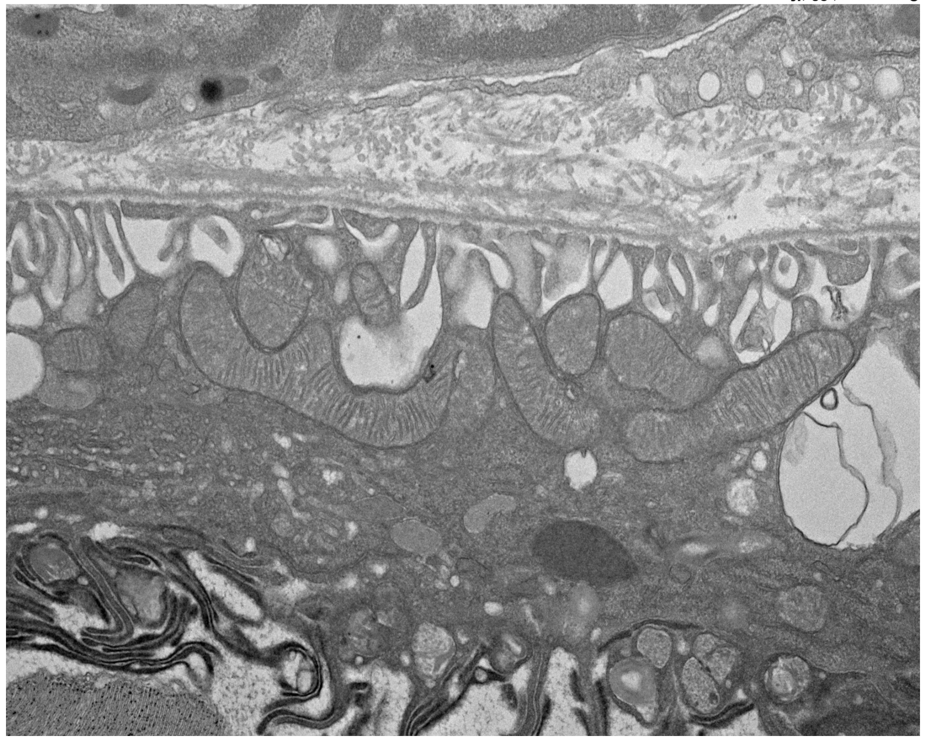

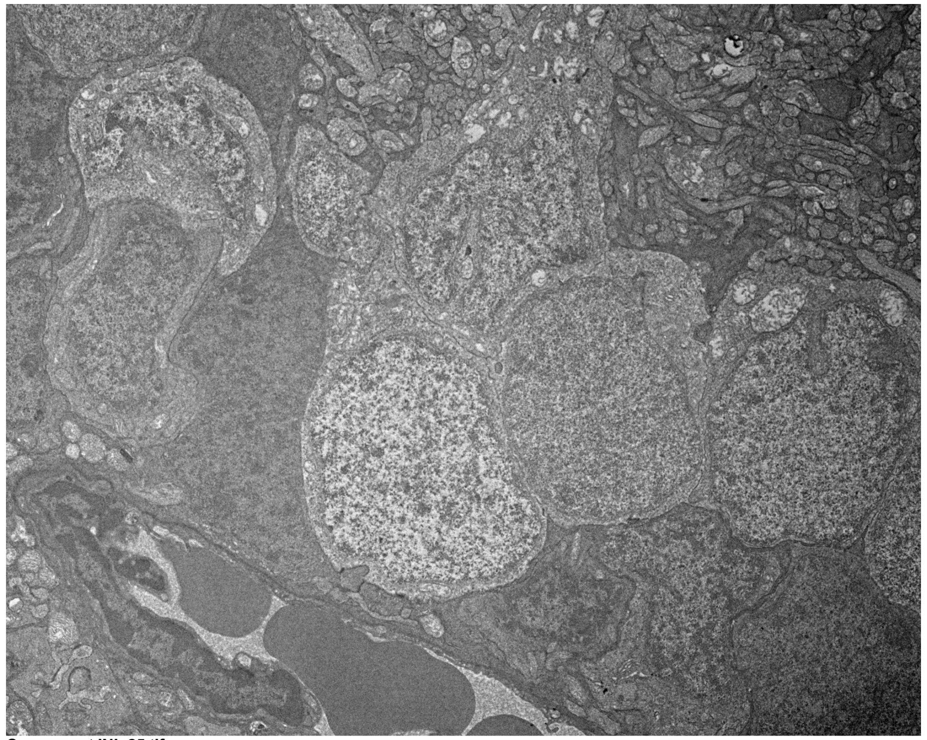

Young INL, IPL, ONL

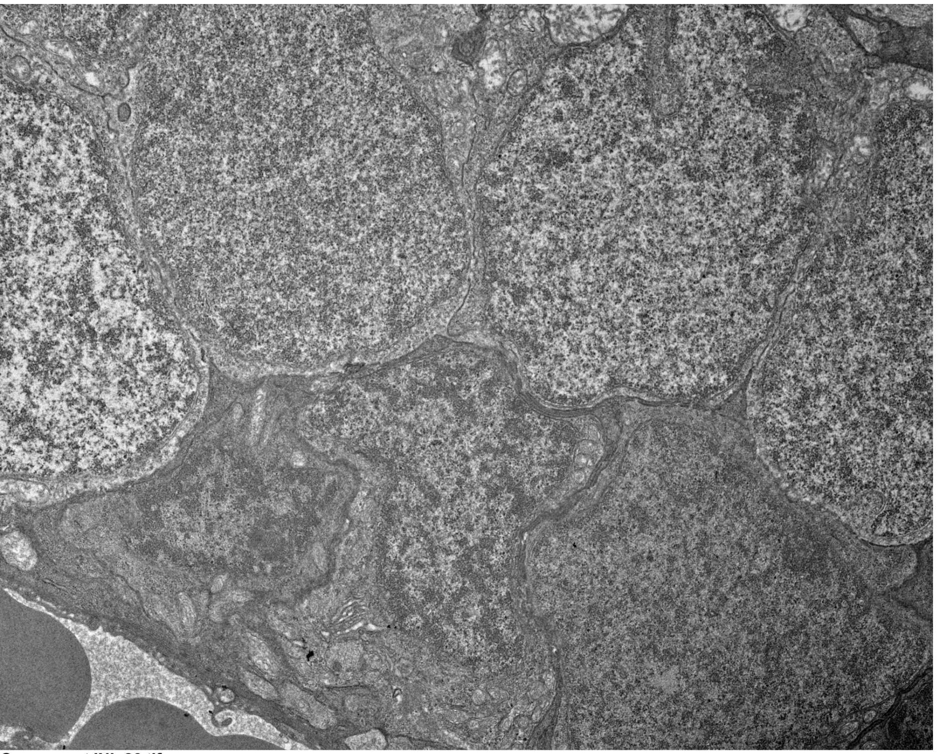

C young rat INL 26.tif
83EM46 Chen
C Young Rat Retina
Print Mag: 11000x @ 7.0 in
11:35 2021-02-04
Microscopist: DBStolz
Camera: BIOSPR12, Exposure: 3200 (ms) x 1 std. frames, Gain: 1, Bin: 1
Gamma: 1.00, No Sharpening, Normal Contrast

2 µm
HV=80kV
Direct Mag: 2500 x
Center for Biologic Imaging

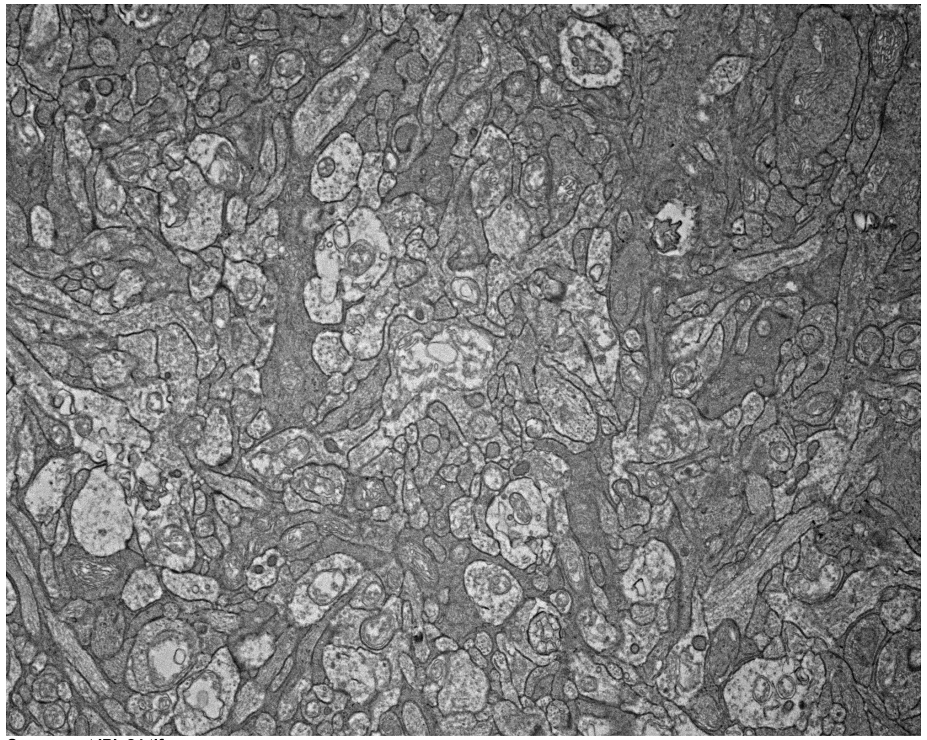

C young rat IPL 24.tif
83EM46 Chen
C Young Rat Retina
Print Mag: 11000x @ 7.0 in
11:30 2021-02-04
Microscopist: DBStolz
Camera: BIOSPR12, Exposure: 3200 (ms) x 1 std. frames, Gain: 1, Bin: 1
Gamma: 1.00, No Sharpening, Normal Contrast

2 µm
HV=80kV
Direct Mag: 2500 x
Center for Biologic Imaging

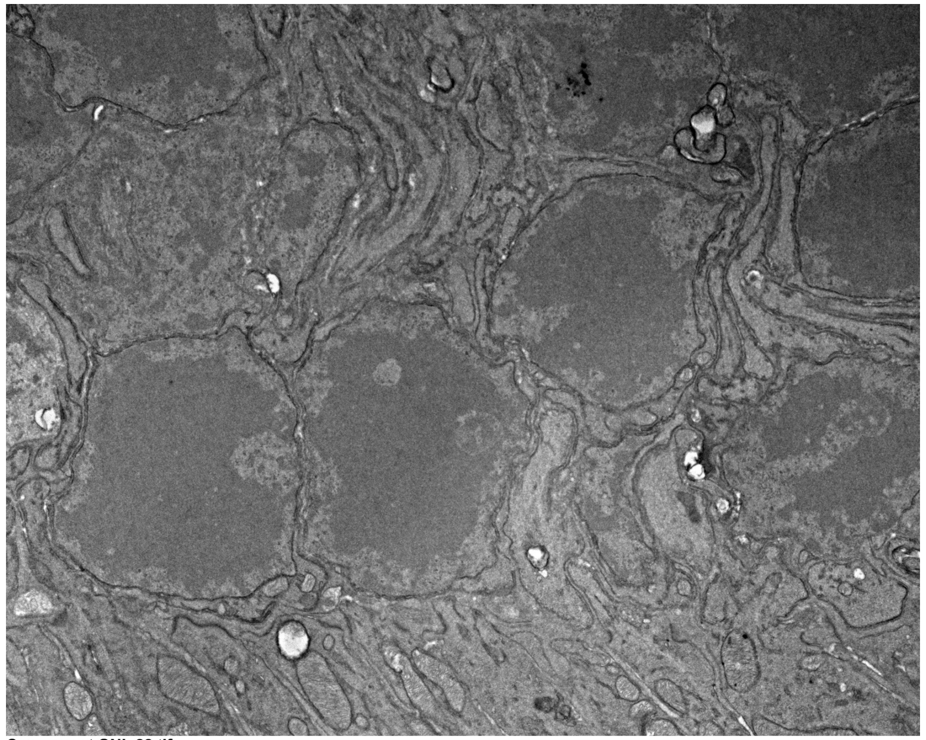

C young rat ONL 22.tif
83EM46 Chen
C Young Rat Retina
Print Mag: 11000x @ 7.0 in
11:27 2021-02-04
Microscopist: DBStolz
Camera: BIOSPR12, Exposure: 3200 (ms) x 1 std. frames, Gain: 1, Bin: 1
Gamma: 1.00, No Sharpening, Normal Contrast

2 µm
HV=80kV
Direct Mag: 2500 x
Center for Biologic Imaging

Young OPL

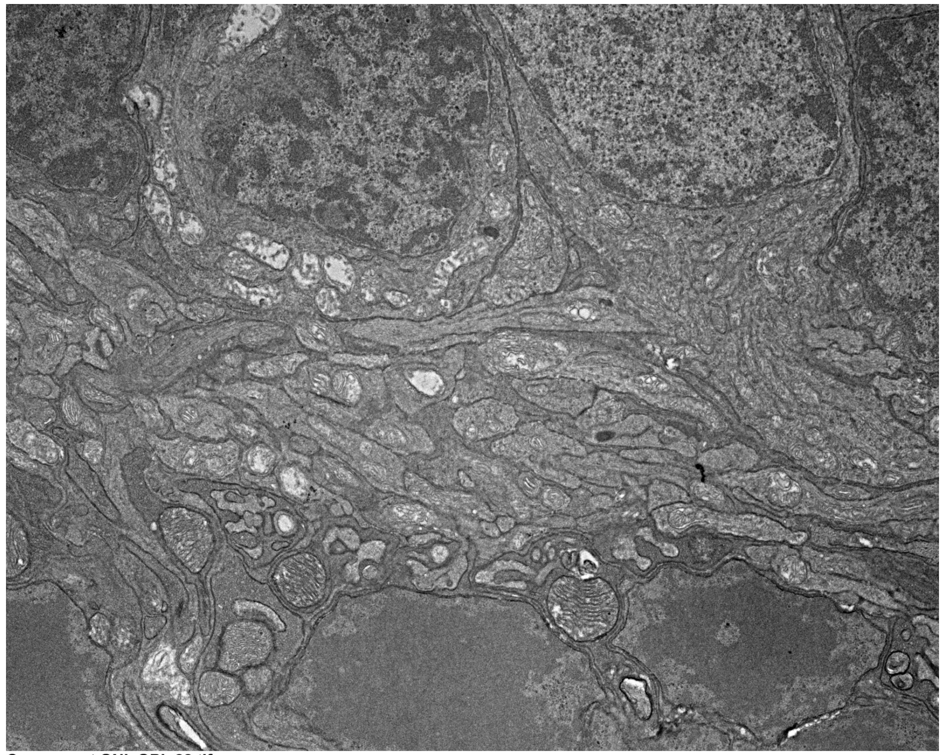

C young rat ONI_OPL 22.tif
83EM46 Chen
C Young Rat Retina
Print Mag: 11000x @ 7.0 in
11:29 2021-02-04
Microscopist: DBStolz
Camera: BIOSPR12, Exposure: 3200 (ms) x 1 std. frames, Gain: 1, Bin: 1
Gamma: 1.00, No Sharpening, Normal Contrast

2 µm
HV=80kV
Direct Mag: 2500 x
Center for Biologic Imaging

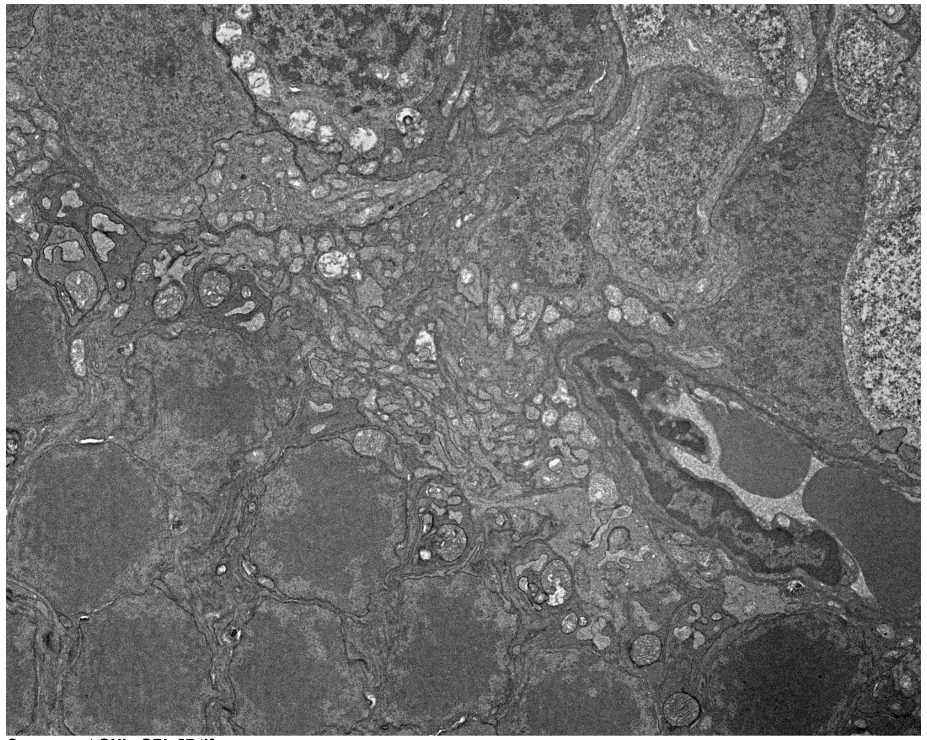

C young rat ONL_OPL 27.tif
83EM46 Chen
C Young Rat Retina
Print Mag: 6600x @ 7.0 in
11:37 2021-02-04
Microscopist: DBStolz
Camera: BIOSPR12, Exposure: 3200 (ms) x 1 std. frames, Gain: 1, Bin: 1
Gamma: 1.00, No Sharpening, Normal Contrast

2 µm
HV=80kV
Direct Mag: 1500 x
Center for Biologic Imaging

Old peripheral retina RPE_OS_IS, IS,OS_IS

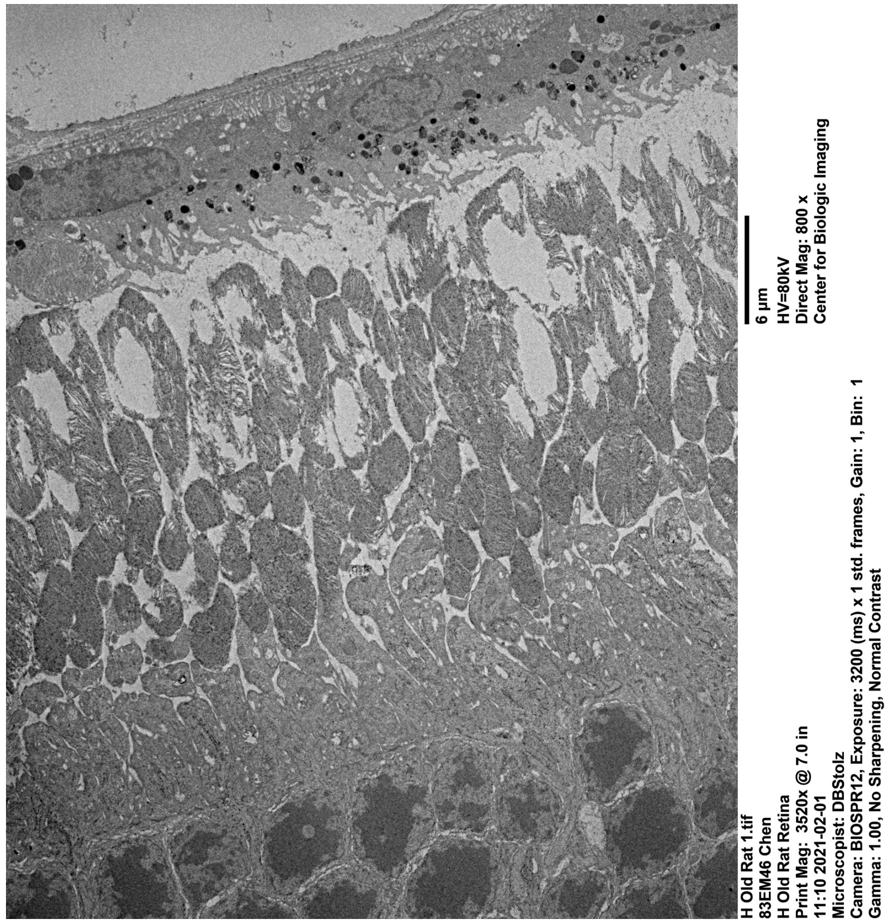

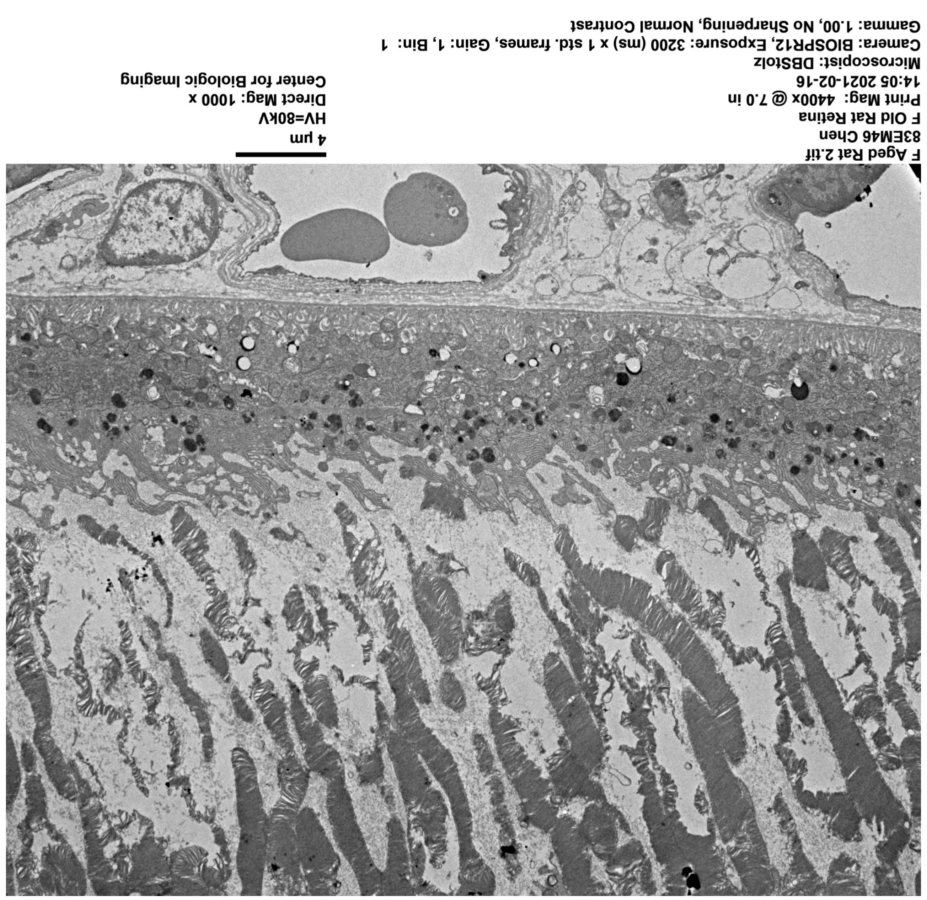

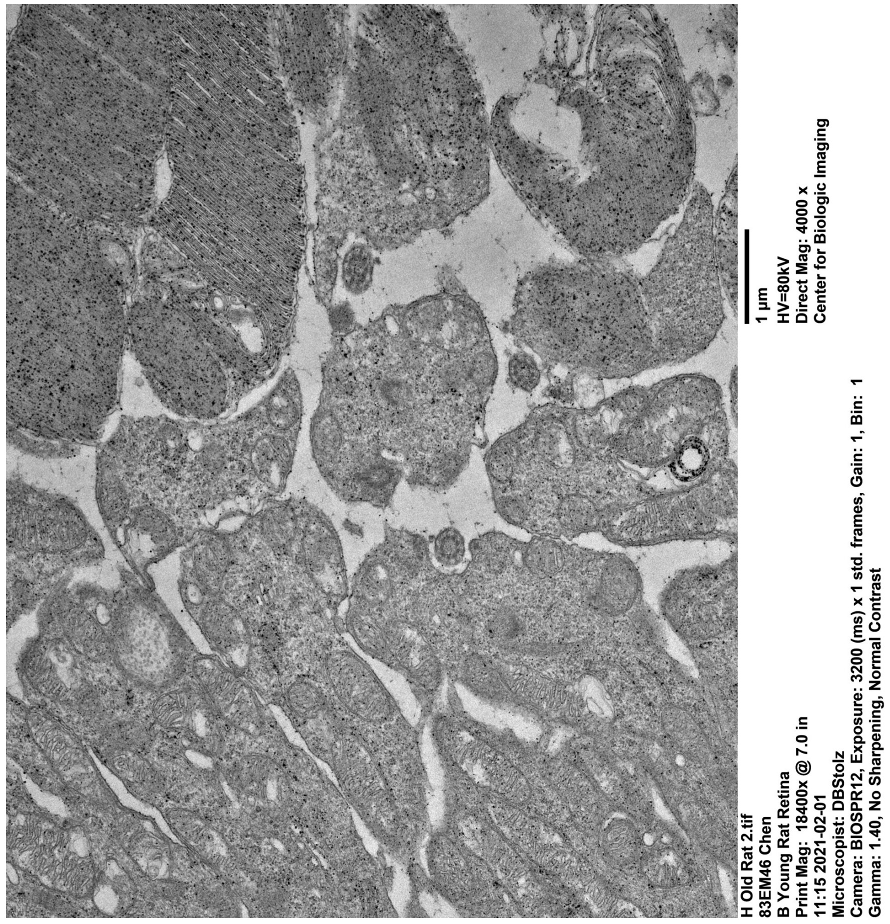

Old RPE

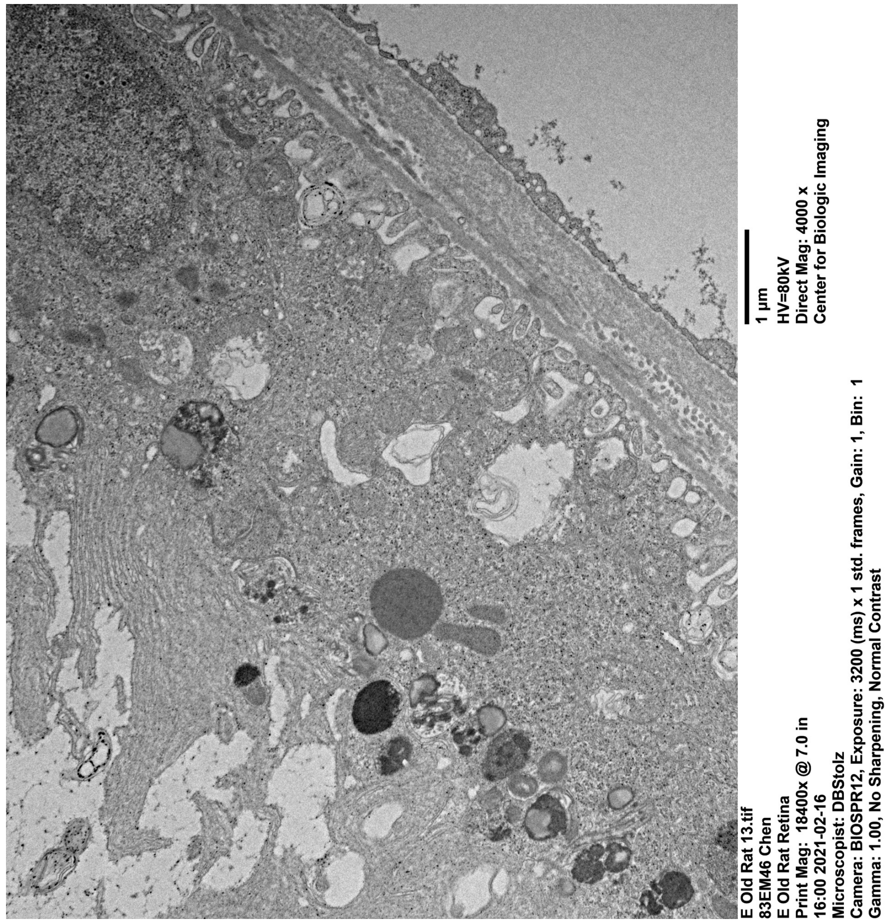
1 µm
HV=80kV
Direct Mag: 4000 x
Center for Biologic Imaging
E Old Rat 13.tif
83EM46 Chen
E Old Rat Retina
Print Mag: 18400x @ 7.0 in
16:00 2021-02-16
Microscopist: DBStolz
Camera: BIOSPR12, Exposure: 3200 (ms) x 1 std. frames, Gain: 1, Bin: 1
Gamma: 1.00, No Sharpening, Normal Contrast

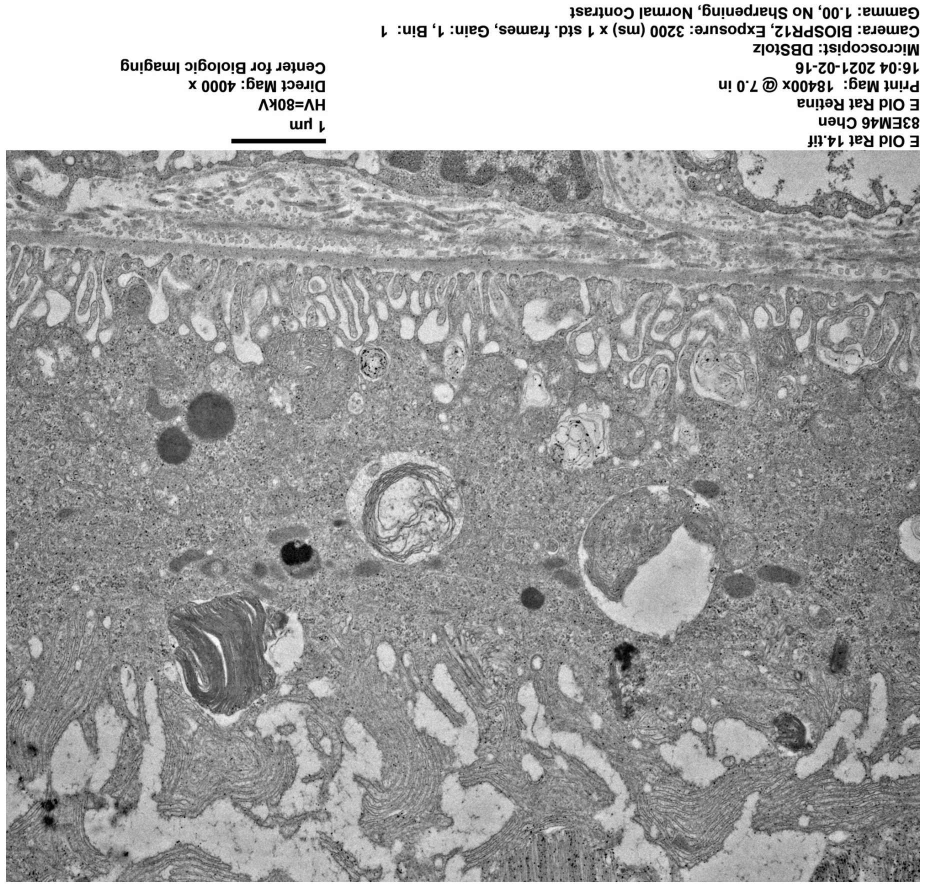
E Old Rat 14.tif
83EM46 Chen
E Old Rat Retina
Print Mag: 18400x @ 7.0 in
16:04 2021-02-16
Microscopist: DBStolz
Camera: BIOSPR12, Exposure: 3200 (ms) x 1 std. frames, Gain: 1, Bin: 1
Gamma: 1.00, No Sharpening, Normal Contrast
1 µm
HV=80kV
Direct Mag: 4000 x
Center for Biologic Imaging

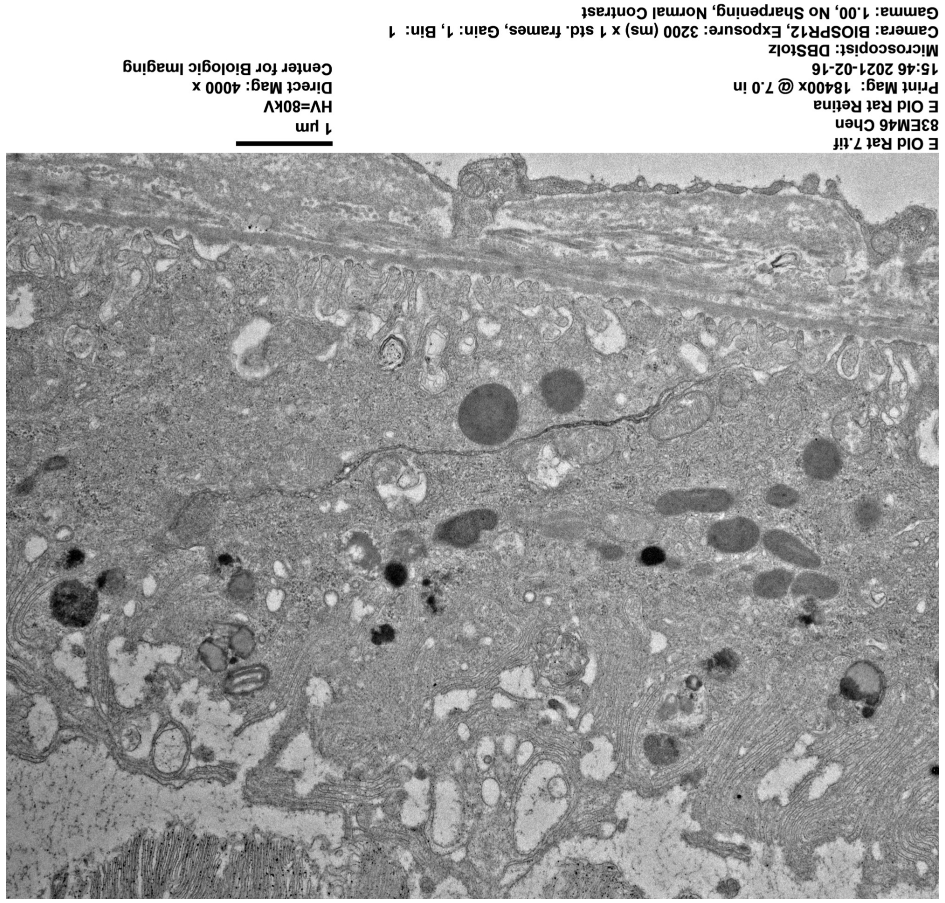
E Old Rat 7.tif
83EM46 Chen
E Old Rat Retina
Print Mag: 18400x @ 7.0 in
15:46 2021-02-16
Microscopist: DBStolz
Camera: BIOSPR12, Exposure: 3200 (ms) x 1 std. frames, Gain: 1, Bin: 1
Gamma: 1.00, No Sharpening, Normal Contrast
1 µm
HV=80kV
Direct Mag: 4000 x
Center for Biologic Imaging

Old RPE

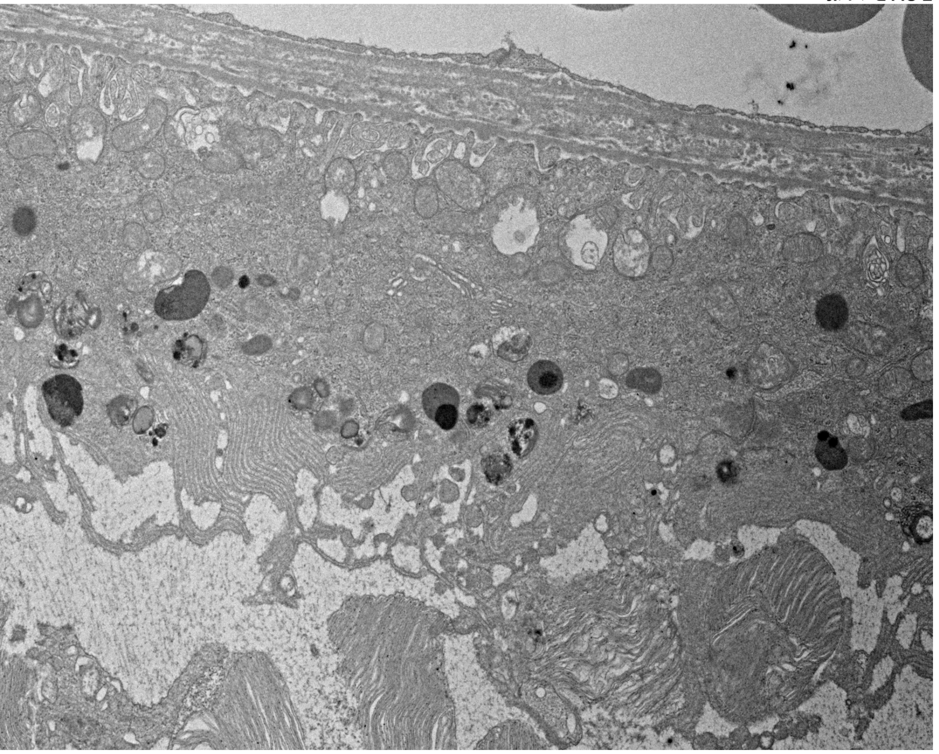
E Old Rat 4.tif
83EM46 Chen
E Old Rat Retina
Print Mag: 13500x @ 7.0 in
15:41 2021-02-16
Microscopist: DBStolz
Camera: BIOSPR12, Exposure: 3200 (ms) x 1 std. frames, Gain: 1, Bin: 1
Gamma: 1.00, No Sharpening, Normal Contrast
1 µm
HV=80kV
Direct Mag: 3000 x
Center for Biologic Imaging

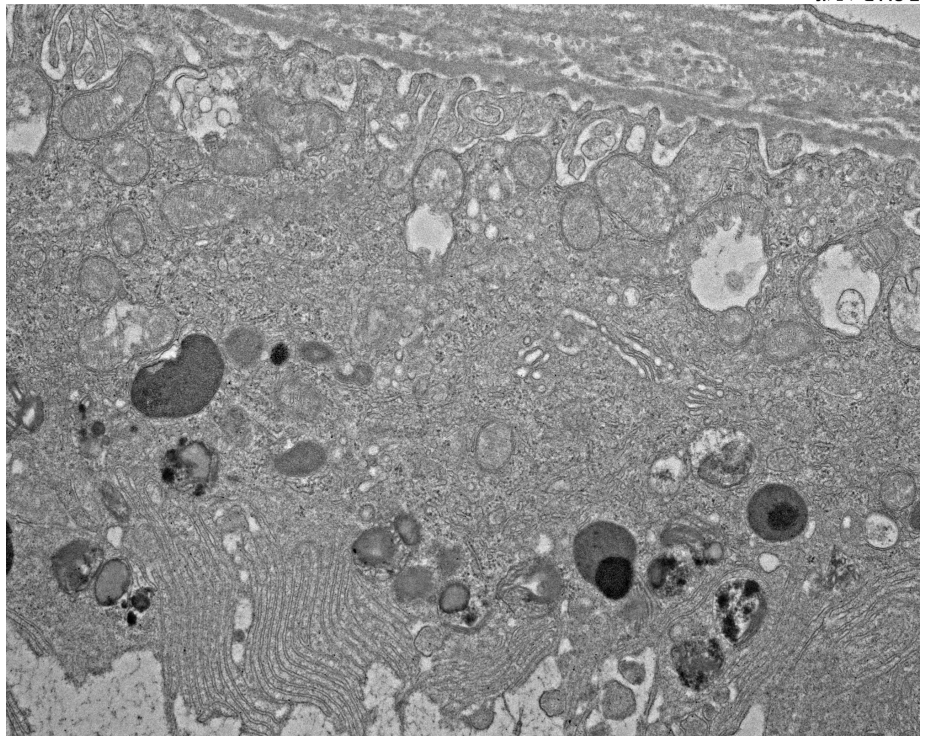
E Old Rat 5.tif
83EM46 Chen
E Old Rat Retina
Print Mag: 22700x @ 7.0 in
15:42 2021-02-16
Microscopist: DBStolz
Camera: BIOSPR12, Exposure: 3200 (ms) x 1 std. frames, Gain: 1, Bin: 1
Gamma: 1.00, No Sharpening, Normal Contrast
800 nm
HV=80kV
Direct Mag: 5000 x
Center for Biologic Imaging

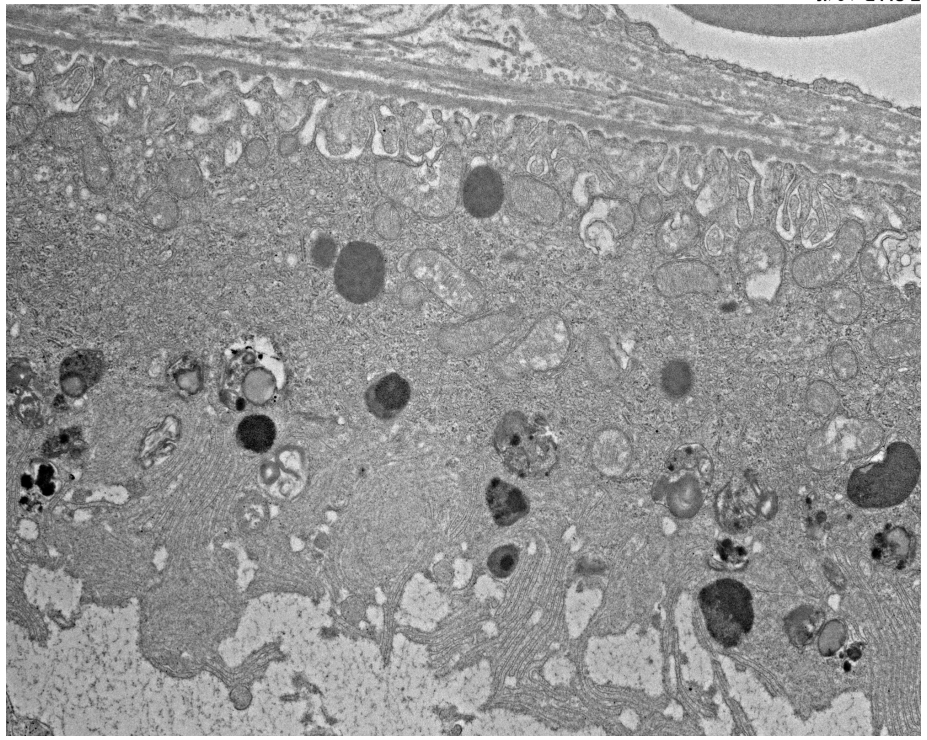
E Old Rat 6.tif
83EM46 Chen
E Old Rat Retina
Print Mag: 18400x @ 7.0 in
15:43 2021-02-16
Microscopist: DBStolz
Camera: BIOSPR12, Exposure: 3200 (ms) x 1 std. frames, Gain: 1, Bin: 1
Gamma: 1.00, No Sharpening, Normal Contrast
1 µm
HV=80kV
Direct Mag: 4000 x
Center for Biologic Imaging

Old RPE

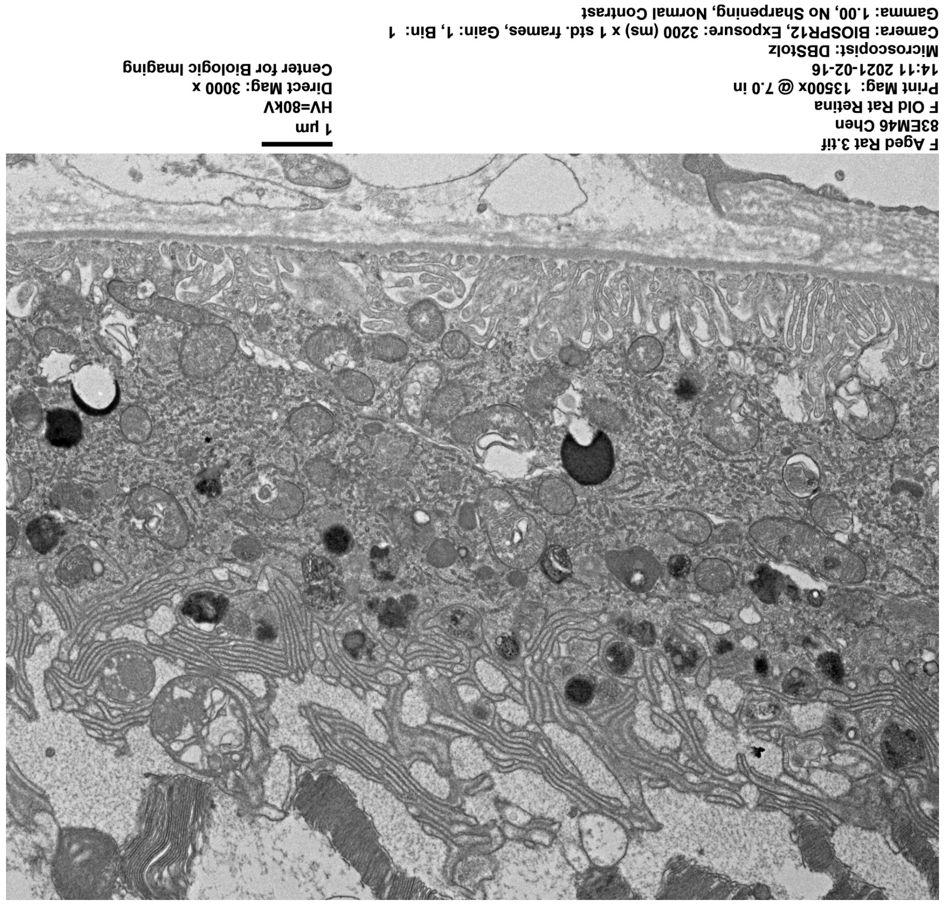
F Aged Rat 3.tif
83EM46 Chen
F Old Rat Retina
Print Mag: 13500x @ 7.0 in
14:11 2021-02-16
Microscopist: DBStolz
Camera: BIOSPR12, Exposure: 3200 (ms) x 1 std. frames, Gain: 1, Bin: 1
Gamma: 1.00, No Sharpening, Normal Contrast
1 µm
HV=80kV
Direct Mag: 3000 x
Center for Biologic Imaging

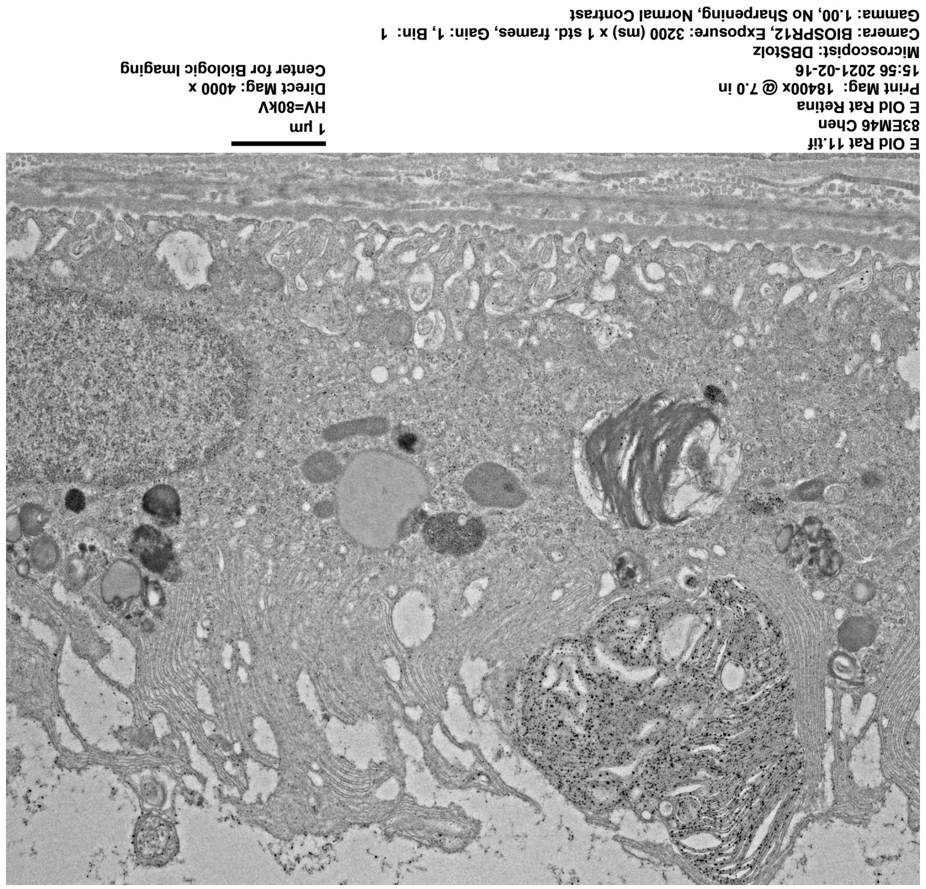
E Old Rat 11.tif
83EM46 Chen
E Old Rat Retina
Print Mag: 18400x @ 7.0 in
15:56 2021-02-16
Microscopist: DBStolz
Camera: BIOSPR12, Exposure: 3200 (ms) x 1 std. frames, Gain: 1, Bin: 1
Gamma: 1.00, No Sharpening, Normal Contrast
1 µm
HV=80kV
Direct Mag: 4000 x
Center for Biologic Imaging

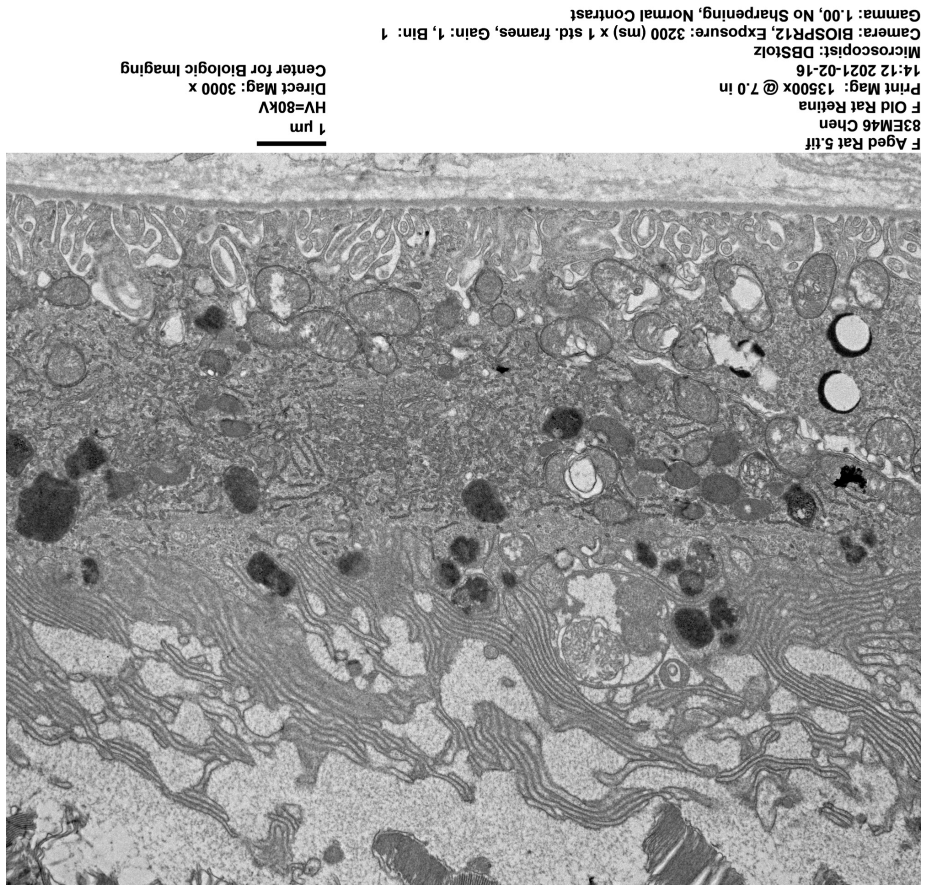
F Aged Rat 5.tif
83EM46 Chen
F Old Rat Retina
Print Mag: 13500x @ 7.0 in
14:12 2021-02-16
Microscopist: DBStolz
Camera: BIOSPR12, Exposure: 3200 (ms) x 1 std. frames, Gain: 1, Bin: 1
Gamma: 1.00, No Sharpening, Normal Contrast
1 µm
HV=80kV
Direct Mag: 3000 x
Center for Biologic Imaging

Old RPE

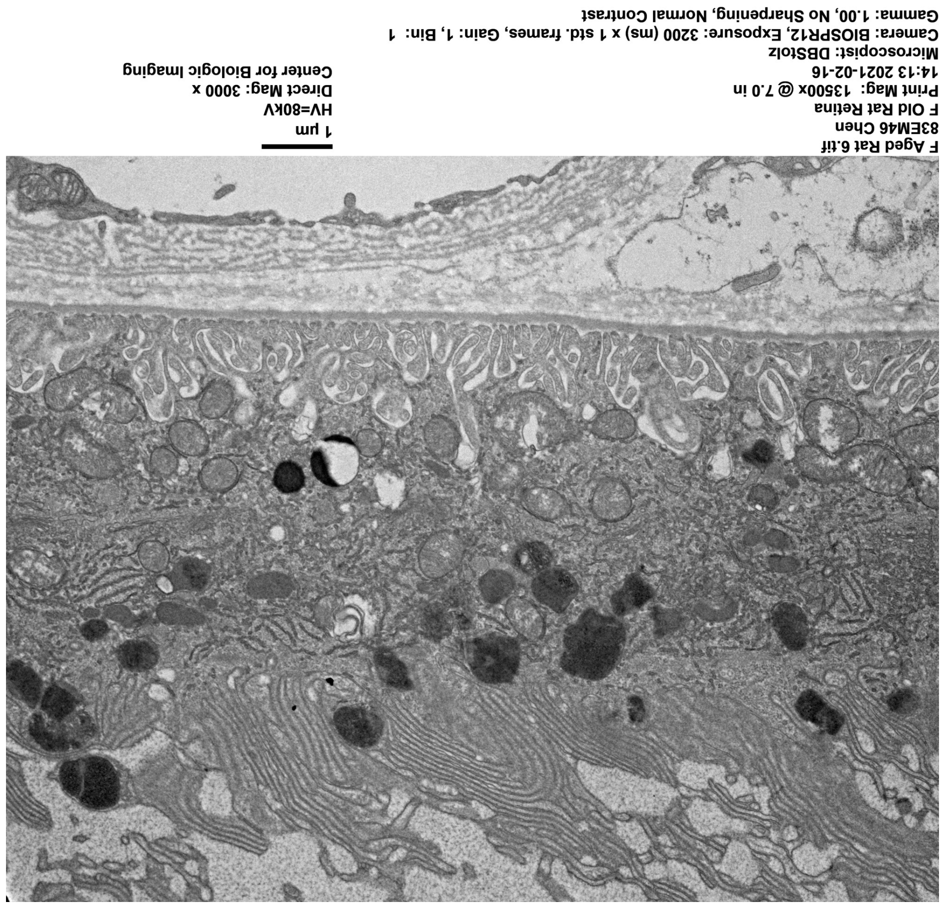

F Aged Rat 6.tif
83EM46 Chen
F Old Rat Retina
Print Mag: 13500x @ 7.0 in
14:13 2021-02-16
Microscopist: DBStolz
Camera: BIOSPR12, Exposure: 3200 (ms) x 1 std. frames, Gain: 1, Bin: 1
Gamma: 1.00, No Sharpening, Normal Contrast
1 µm
HV=80kV
Direct Mag: 3000 x
Center for Biologic Imaging

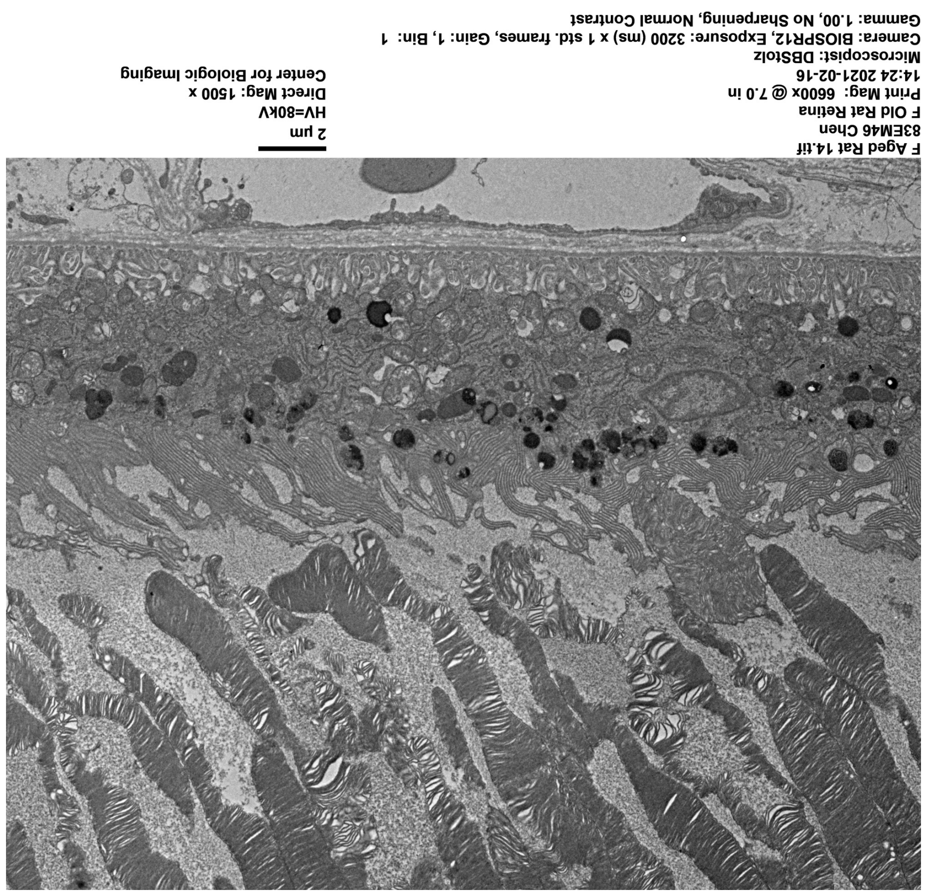

F Aged Rat 14.tif
83EM46 Chen
F Old Rat Retina
Print Mag: 6600x @ 7.0 in
14:24 2021-02-16
Microscopist: DBStolz
Camera: BIOSPR12, Exposure: 3200 (ms) x 1 std. frames, Gain: 1, Bin: 1
Gamma: 1.00, No Sharpening, Normal Contrast
2 µm
HV=80kV
Direct Mag: 1500 x
Center for Biologic Imaging

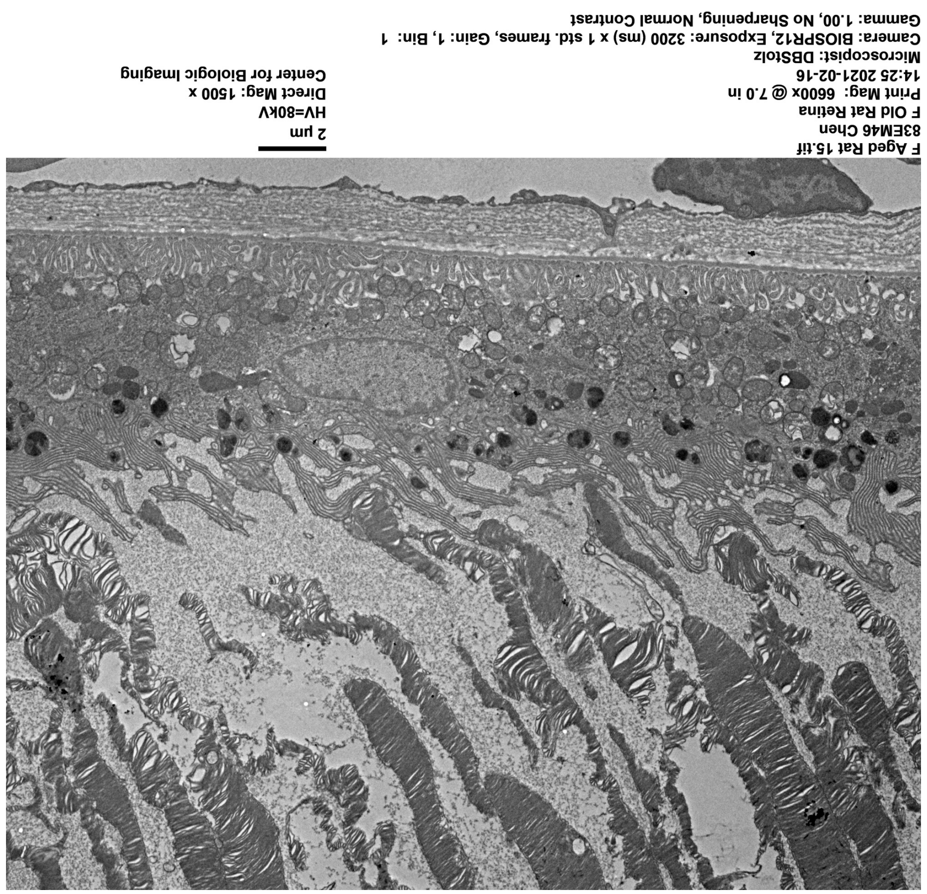

F Aged Rat 15.tif
83EM46 Chen
F Old Rat Retina
Print Mag: 6600x @ 7.0 in
14:25 2021-02-16
Microscopist: DBStolz
Camera: BIOSPR12, Exposure: 3200 (ms) x 1 std. frames, Gain: 1, Bin: 1
Gamma: 1.00, No Sharpening, Normal Contrast
2 µm
HV=80kV
Direct Mag: 1500 x
Center for Biologic Imaging

Old RPE

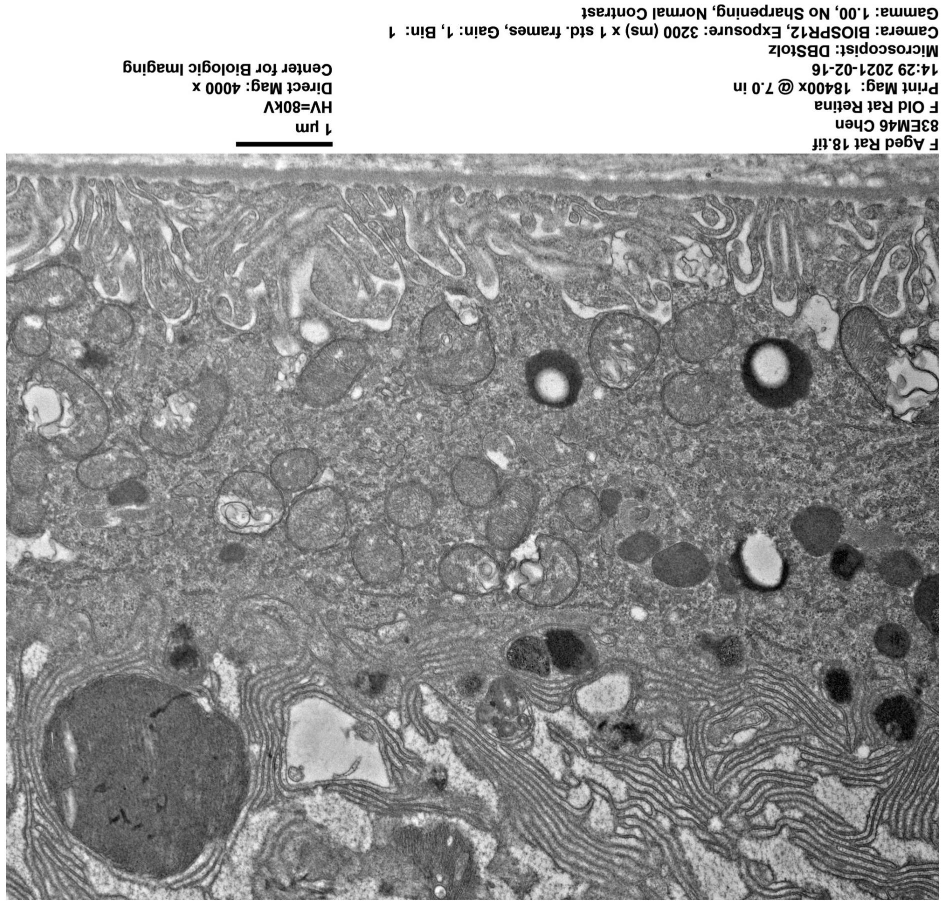
F Aged Rat 18.tif
83EM46 Chen
F Old Rat Retina
Print Mag: 18400x @ 7.0 in
14:29 2021-02-16
Microscopist: DBStolz
Camera: BIOSPR12, Exposure: 3200 (ms) x 1 std. frames, Gain: 1, Bin: 1
Gamma: 1.00, No Sharpening, Normal Contrast
1 µm
HV=80kV
Direct Mag: 4000 x
Center for Biologic Imaging

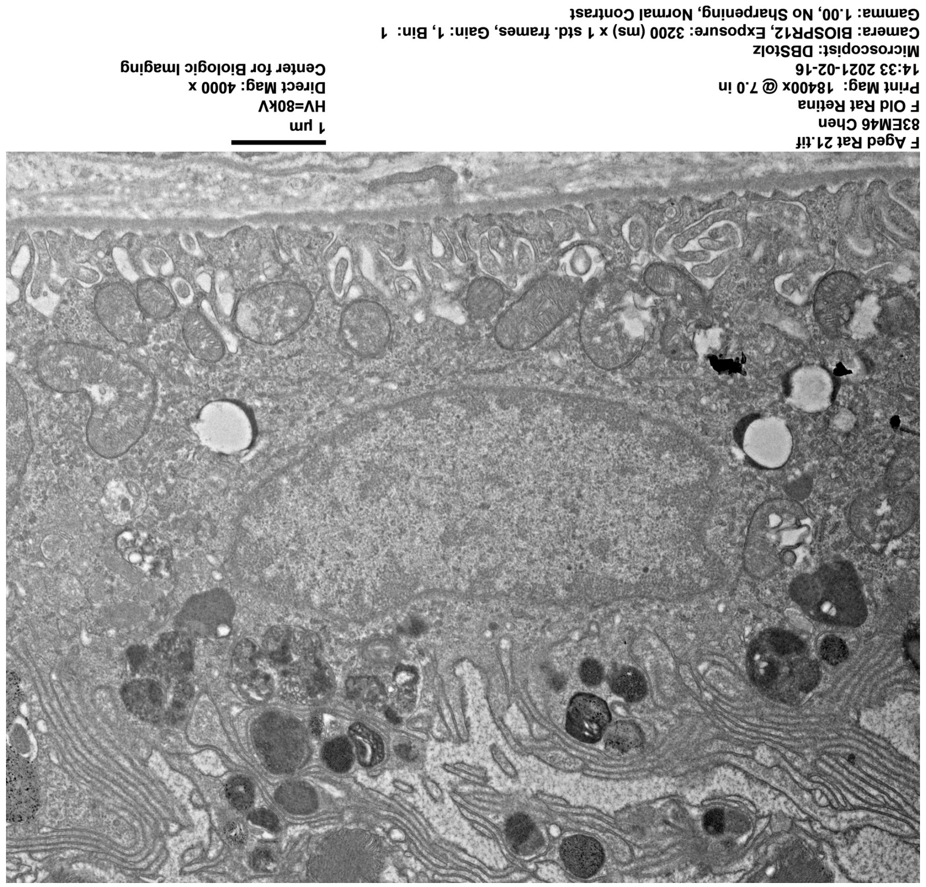
F Aged Rat 21.tif
83EM46 Chen
F Old Rat Retina
Print Mag: 18400x @ 7.0 in
14:33 2021-02-16
Microscopist: DBStolz
Camera: BIOSPR12, Exposure: 3200 (ms) x 1 std. frames, Gain: 1, Bin: 1
Gamma: 1.00, No Sharpening, Normal Contrast
1 µm
HV=80kV
Direct Mag: 4000 x
Center for Biologic Imaging

Old OS

F Aged Rat 4.tif
83EM46 Chen
F Old Rat Retina
Print Mag: 11000x @ 7.0 in
14:09 2021-02-16
Microscopist: DBStolz
Camera: BIOSPR12, Exposure: 3200 (ms) x 1 std. frames, Gain: 1, Bin: 1
Gamma: 1.00, No Sharpening, Normal Contrast

2 µm
HV=80kV
Direct Mag: 2500 x
Center for Biologic Imaging

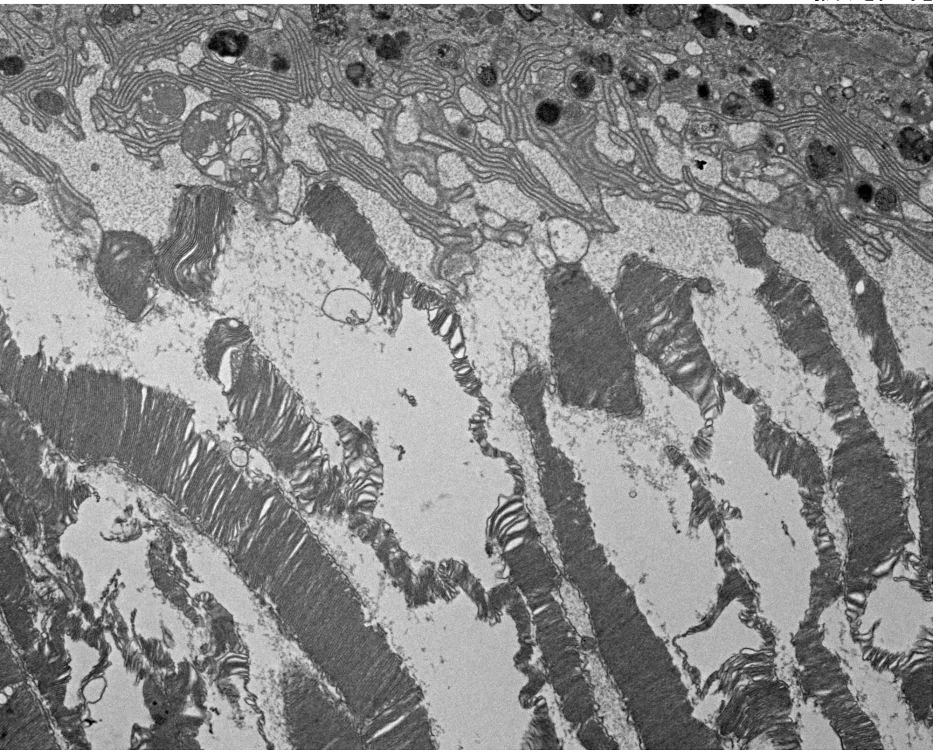

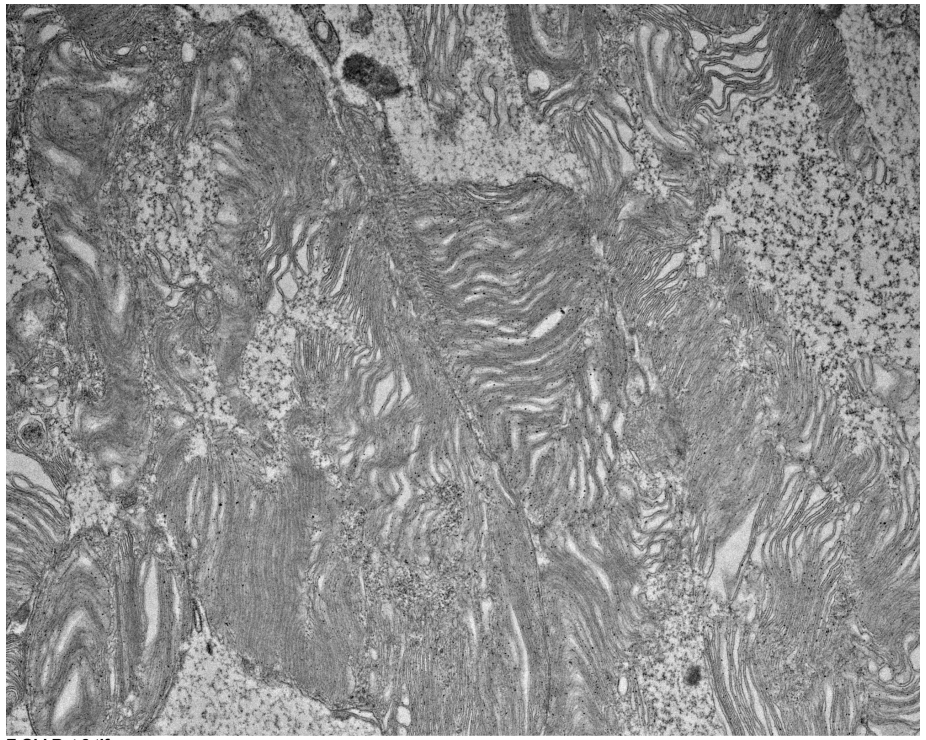

E Old Rat 8.tif
83EM46 Chen
E Old Rat Retina
Print Mag: 18400x @ 7.0 in
15:47 2021-02-16
Microscopist: DBStolz
Camera: BIOSPR12, Exposure: 3200 (ms) x 1 std. frames, Gain: 1, Bin: 1
Gamma: 1.00, No Sharpening, Normal Contrast

1 µm
HV=80kV
Direct Mag: 4000 x
Center for Biologic Imaging

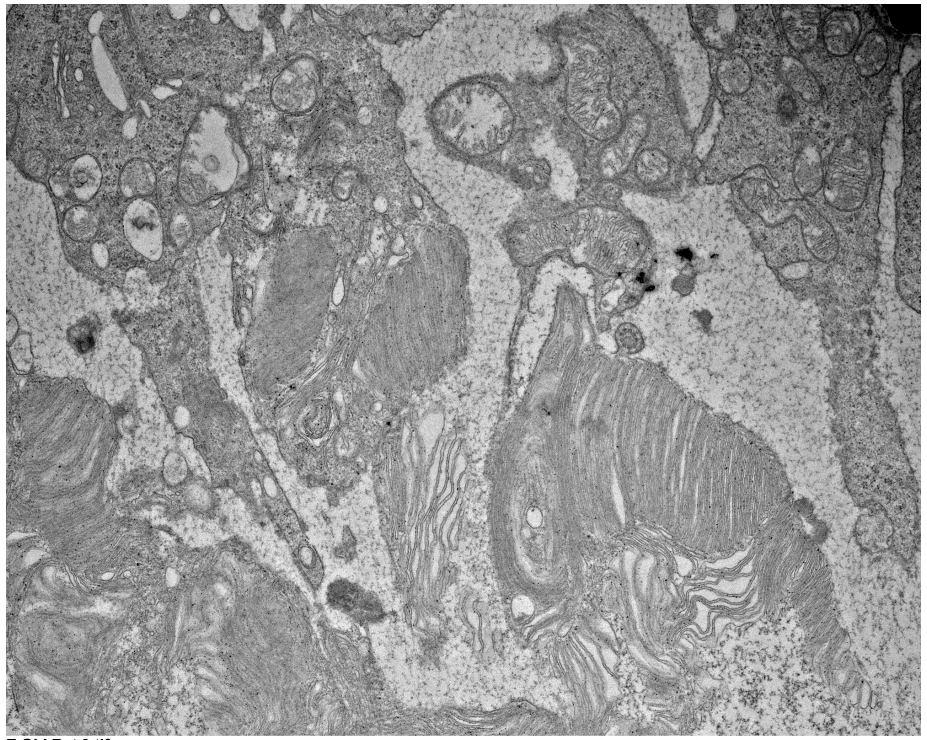

E Old Rat 9.tif
83EM46 Chen
E Old Rat Retina
Print Mag: 18400x @ 7.0 in
15:48 2021-02-16
Microscopist: DBStolz
Camera: BIOSPR12, Exposure: 3200 (ms) x 1 std. frames, Gain: 1, Bin: 1
Gamma: 1.00, No Sharpening, Normal Contrast

1 µm
HV=80kV
Direct Mag: 4000 x
Center for Biologic Imaging

Old OS

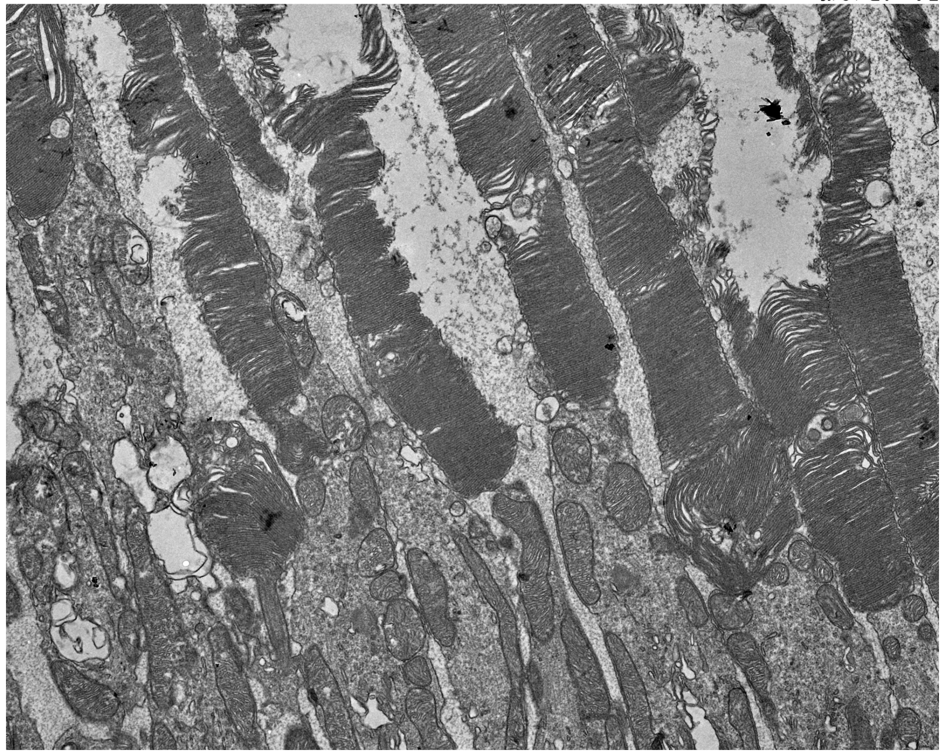

F Aged Rat 9.tif
83EM46 Chen
F Old Rat Retina
Print Mag: 11000x @ 7.0 in
14:17 2021-02-16
Microscopist: DBStolz
Camera: BIOSPR12, Exposure: 3200 (ms) x 1 std. frames, Gain: 1, Bin: 1
Gamma: 1.00, No Sharpening, Normal Contrast

2 µm
HV=80kV
Direct Mag: 2500 x
Center for Biologic Imaging

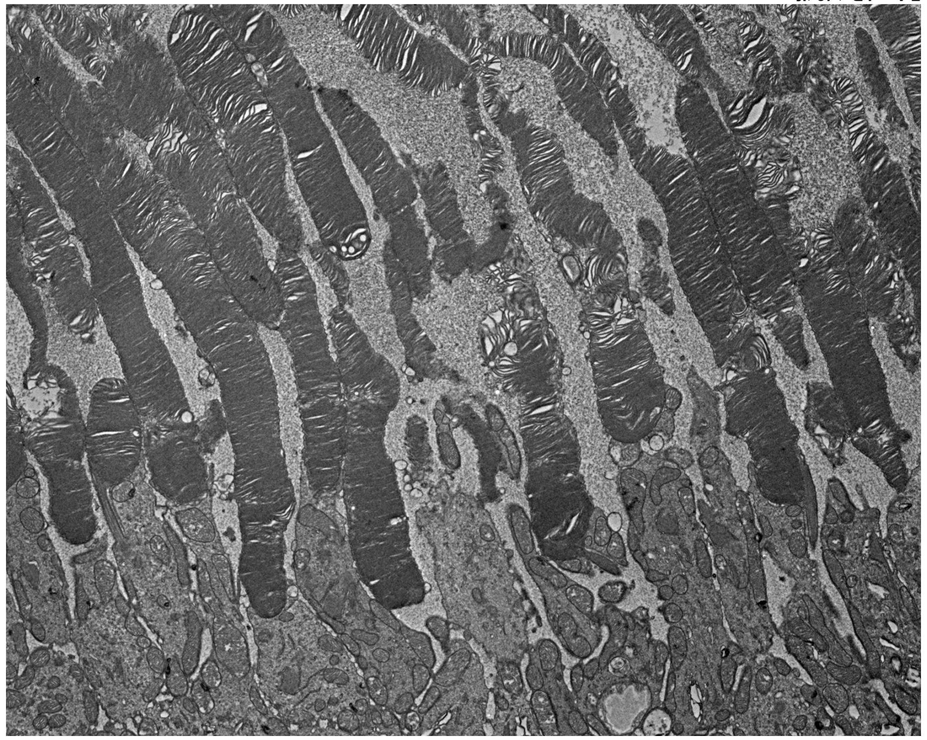

F Aged Rat 13.tif
83EM46 Chen
F Old Rat Retina
Print Mag: 6600x @ 7.0 in
14:23 2021-02-16
Microscopist: DBStolz
Camera: BIOSPR12, Exposure: 3200 (ms) x 1 std. frames, Gain: 1, Bin: 1
Gamma: 1.00, No Sharpening, Normal Contrast

2 µm
HV=80kV
Direct Mag: 1500 x
Center for Biologic Imaging

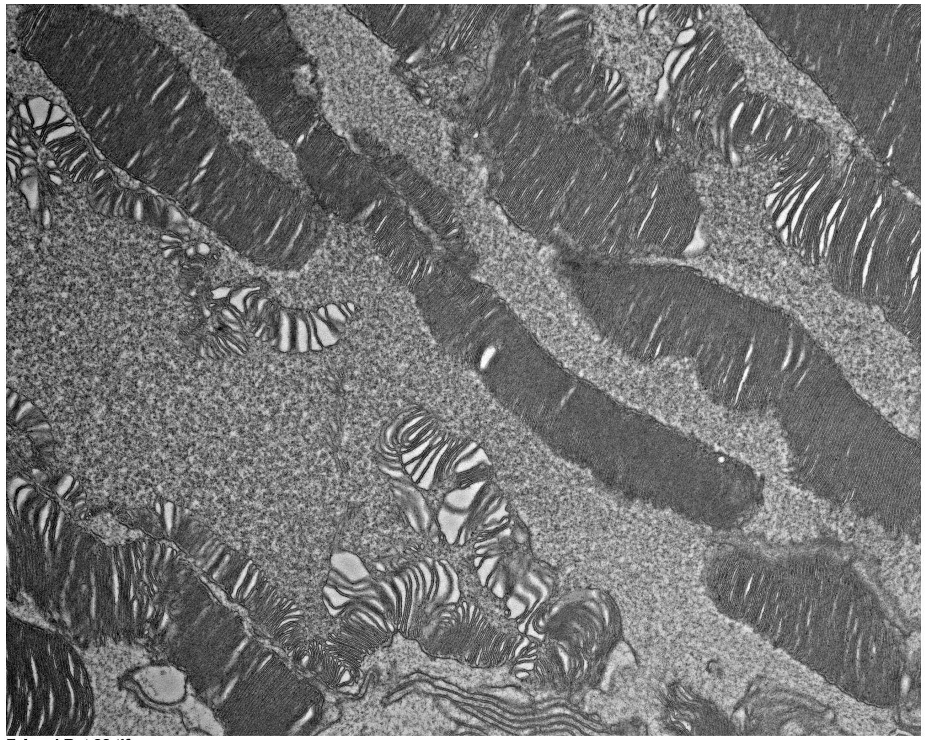

F Aged Rat 22.tif
83EM46 Chen
F Old Rat Retina
Print Mag: 18400x @ 7.0 in
14:33 2021-02-16
Microscopist: DBStolz
Camera: BIOSPR12, Exposure: 3200 (ms) x 1 std. frames, Gain: 1, Bin: 1
Gamma: 1.00, No Sharpening, Normal Contrast

1 µm
HV=80kV
Direct Mag: 4000 x
Center for Biologic Imaging

Old OS

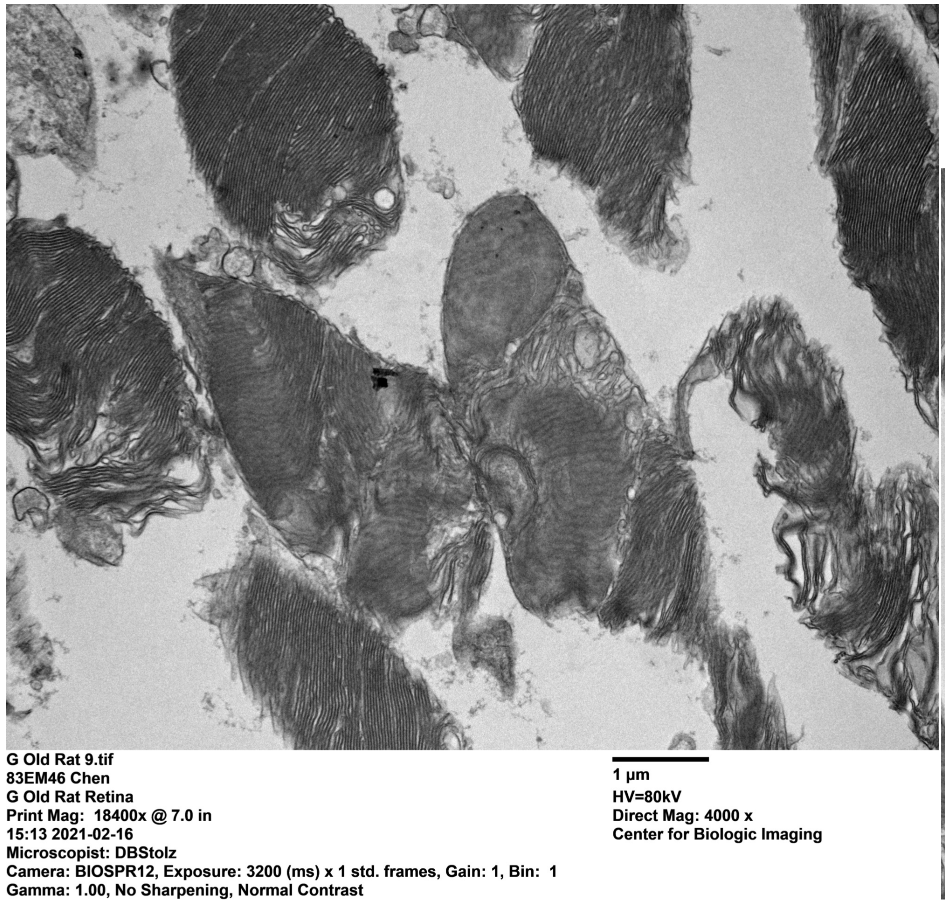

G Old Rat 9.tif
83EM46 Chen
G Old Rat Retina
Print Mag: 18400x @ 7.0 in
15:13 2021-02-16
Microscopist: DBStolz
Camera: BIOSPR12, Exposure: 3200 (ms) x 1 std. frames, Gain: 1, Bin: 1
Gamma: 1.00, No Sharpening, Normal Contrast
1 µm
HV=80kV
Direct Mag: 4000 x
Center for Biologic Imaging

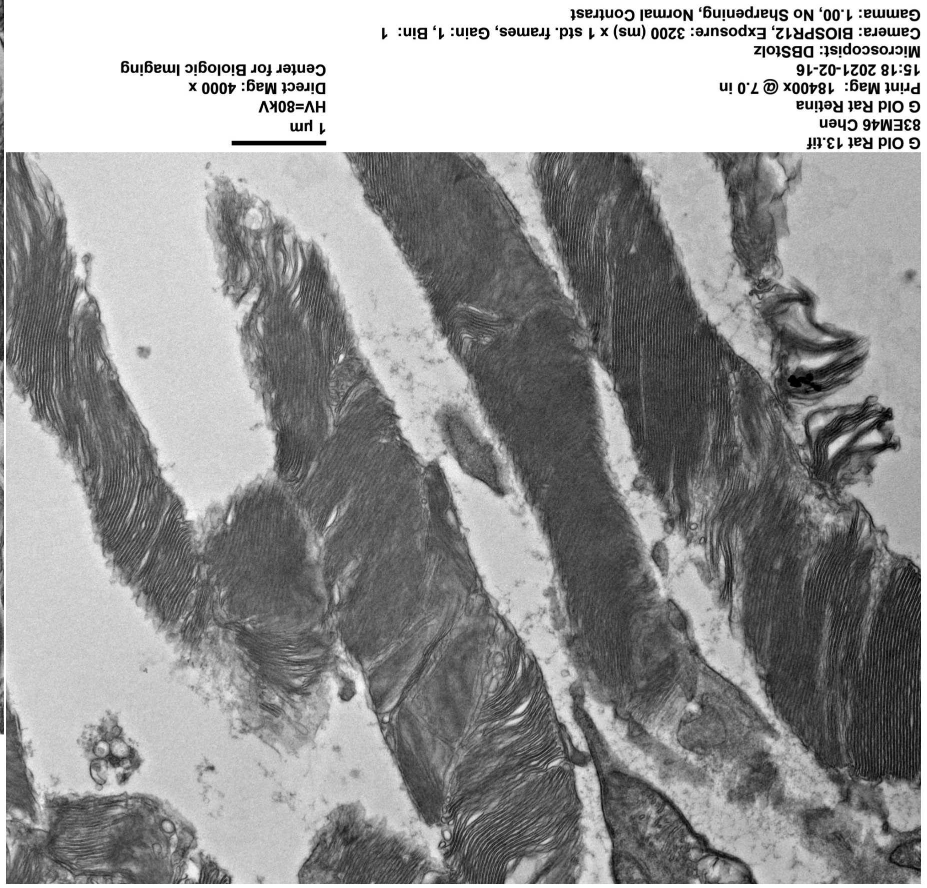

G Old Rat 13.tif
83EM46 Chen
G Old Rat Retina
Print Mag: 18400x @ 7.0 in
15:18 2021-02-16
Microscopist: DBStolz
Camera: BIOSPR12, Exposure: 3200 (ms) x 1 std. frames, Gain: 1, Bin: 1
Gamma: 1.00, No Sharpening, Normal Contrast
1 µm
HV=80kV
Direct Mag: 4000 x
Center for Biologic Imaging

Old IS

E Old Rat 10.tif
83EM46 Chen
E Old Rat Retina
Print Mag: 18400x @ 7.0 in
15:50 2021-02-16
Microscopist: DBStolz
Camera: BIOSPR12, Exposure: 3200 (ms) x 1 std. frames, Gain: 1, Bin: 1
Gamma: 1.00, No Sharpening, Normal Contrast

1 µm
HV=80kV
Direct Mag: 4000 x
Center for Biologic Imaging

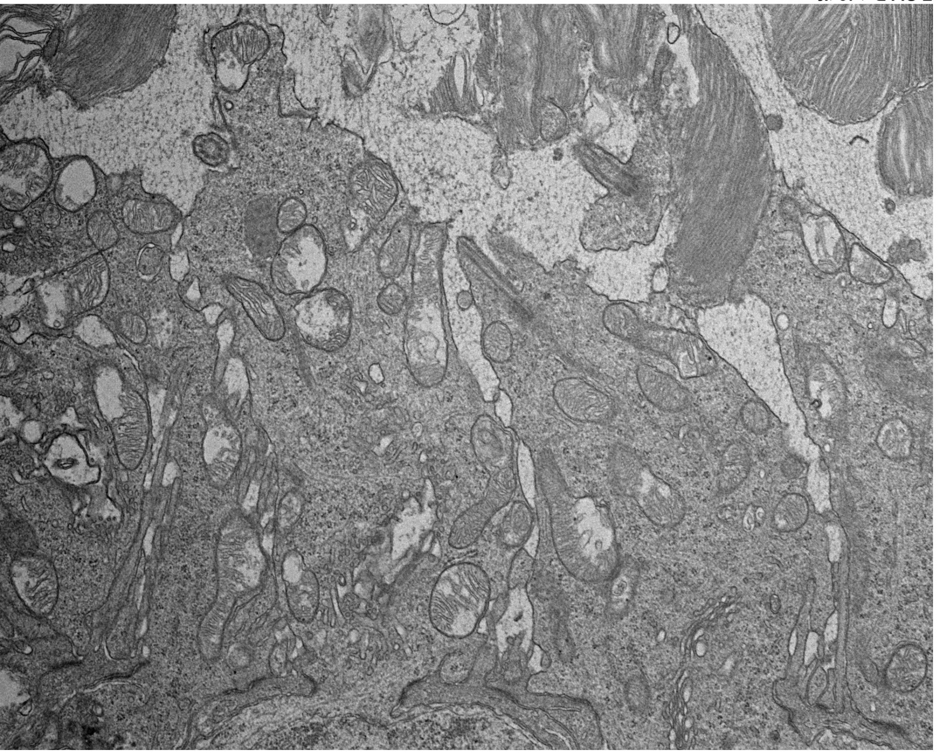

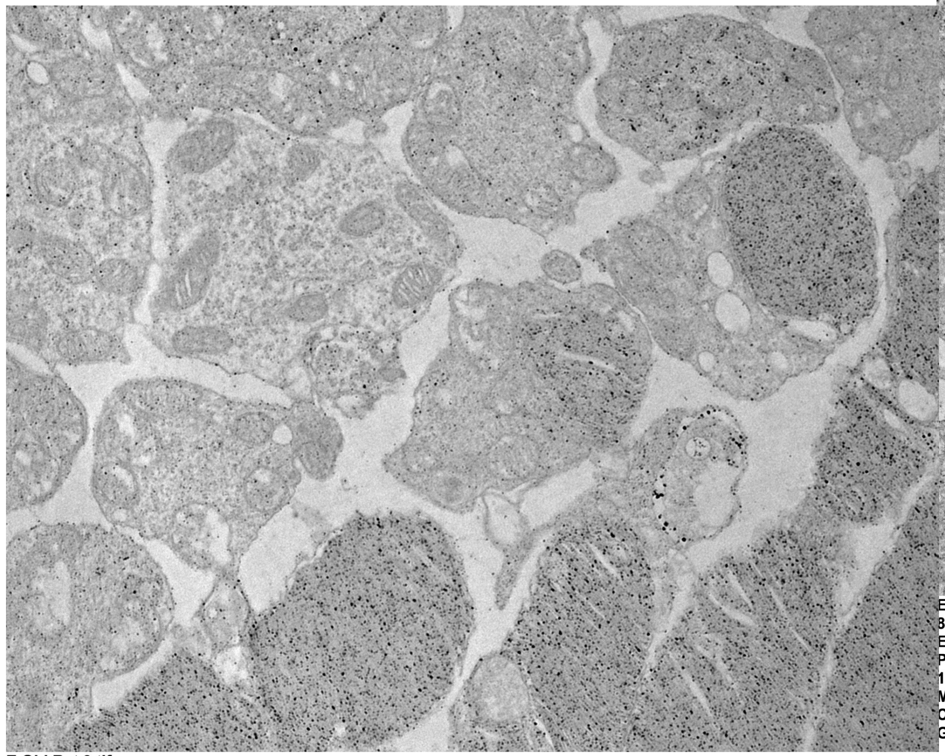

E Old Rat 3.tif
83EM46 Chen
E Old Rat Retina
Print Mag: 18400x @ 7.0 in
12:24 2021-02-01
Microscopist: DBStolz
Camera: BIOSPR12, Exposure: 3200 (ms) x 1 std. frames, Gain: 1, Bin: 1
Gamma: 1.00, No Sharpening, Normal Contrast

1 µm
HV=80kV
Direct Mag: 4000 x
Center for Biologic Imaging

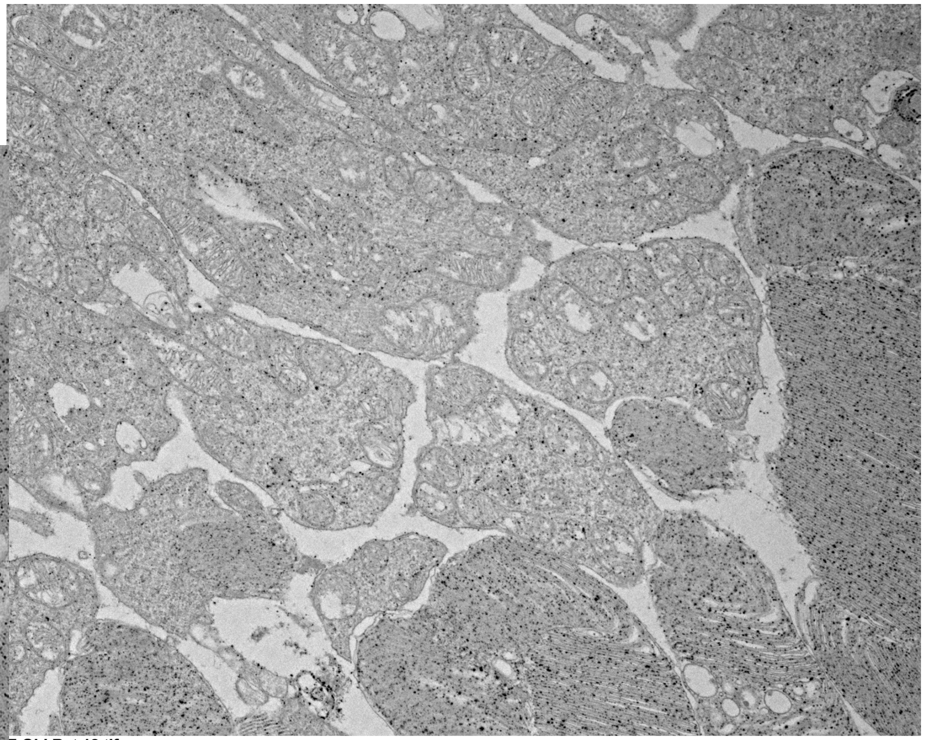

E Old Rat 12.tif
83EM46 Chen
E Old Rat Retina
Print Mag: 18400x @ 7.0 in
15:59 2021-02-16
Microscopist: DBStolz
Camera: BIOSPR12, Exposure: 3200 (ms) x 1 std. frames, Gain: 1, Bin: 1
Gamma: 1.00, No Sharpening, Normal Contrast

1 µm
HV=80kV
Direct Mag: 4000 x
Center for Biologic Imaging

Old IS

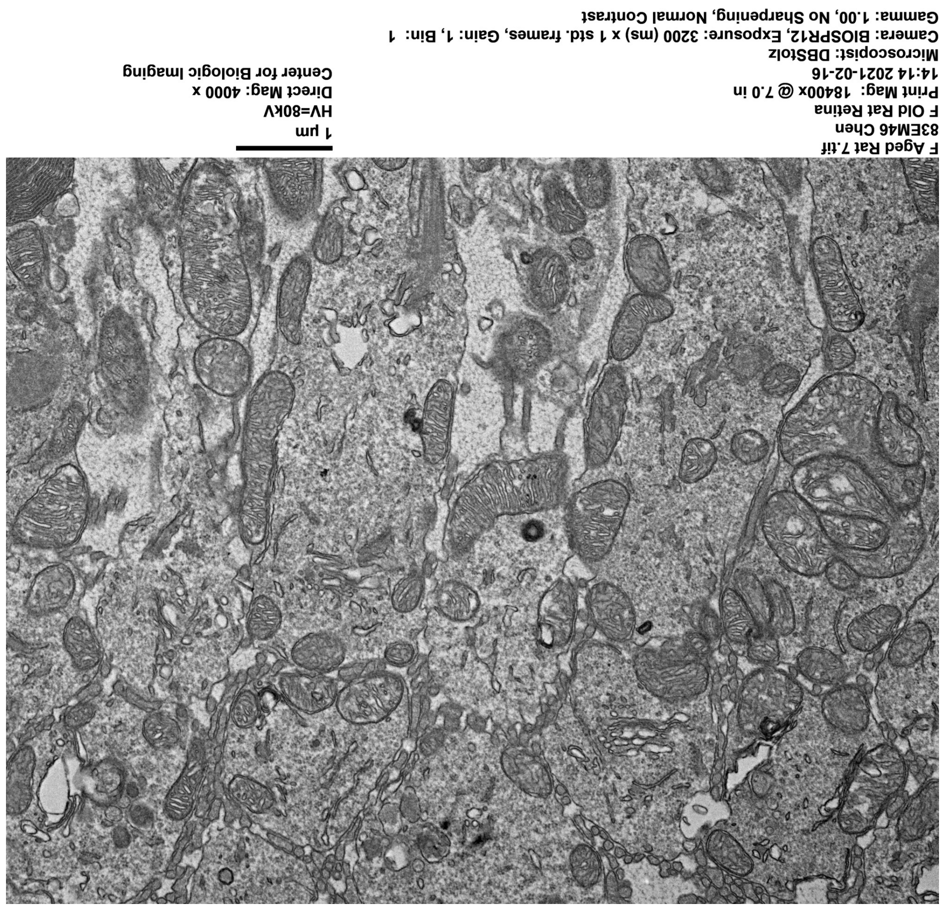
F Aged Rat 7.tif
83EM46 Chen
F Old Rat Retina
Print Mag: 18400x @ 7.0 in
14:14 2021-02-16
Microscopist: DBStolz
Camera: BIOSPR12, Exposure: 3200 (ms) x 1 std. frames, Gain: 1, Bin: 1
Gamma: 1.00, No Sharpening, Normal Contrast
1 µm
HV=80kV
Direct Mag: 4000 x
Center for Biologic Imaging

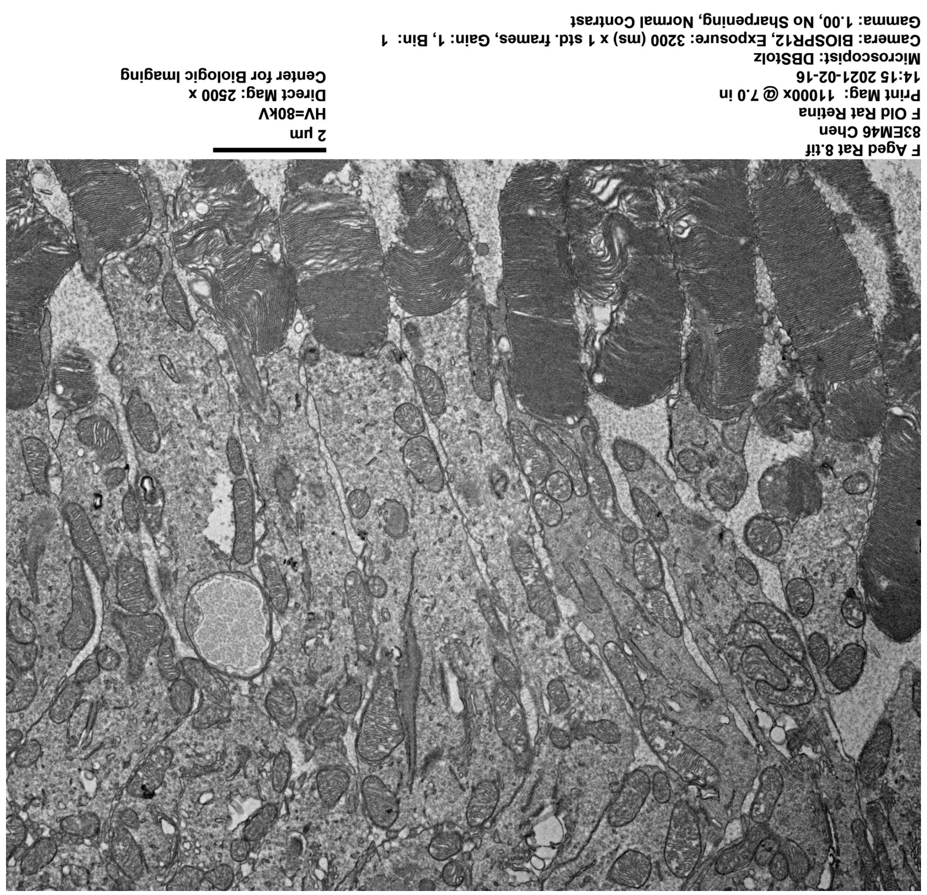
F Aged Rat 8.tif
83EM46 Chen
F Old Rat Retina
Print Mag: 11000x @ 7.0 in
14:15 2021-02-16
Microscopist: DBStolz
Camera: BIOSPR12, Exposure: 3200 (ms) x 1 std. frames, Gain: 1, Bin: 1
Gamma: 1.00, No Sharpening, Normal Contrast
2 µm
HV=80kV
Direct Mag: 2500 x
Center for Biologic Imaging

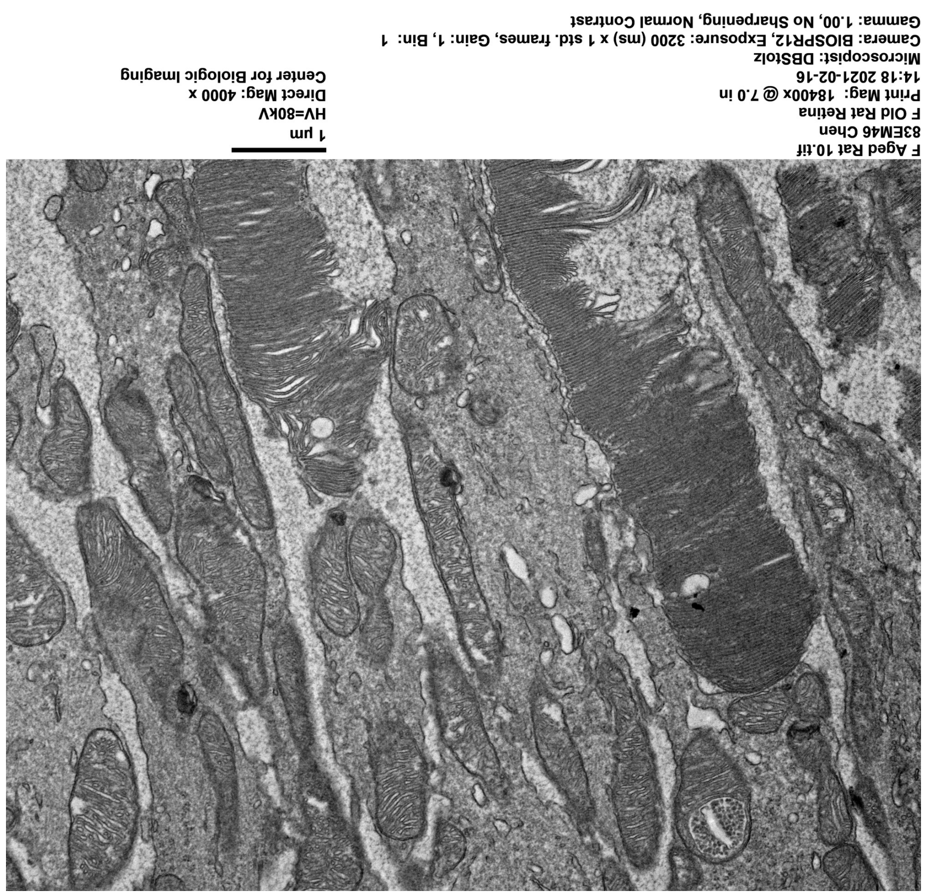
F Aged Rat 10.tif
83EM46 Chen
F Old Rat Retina
Print Mag: 18400x @ 7.0 in
14:18 2021-02-16
Microscopist: DBStolz
Camera: BIOSPR12, Exposure: 3200 (ms) x 1 std. frames, Gain: 1, Bin: 1
Gamma: 1.00, No Sharpening, Normal Contrast
1 µm
HV=80kV
Direct Mag: 4000 x
Center for Biologic Imaging

Old IS

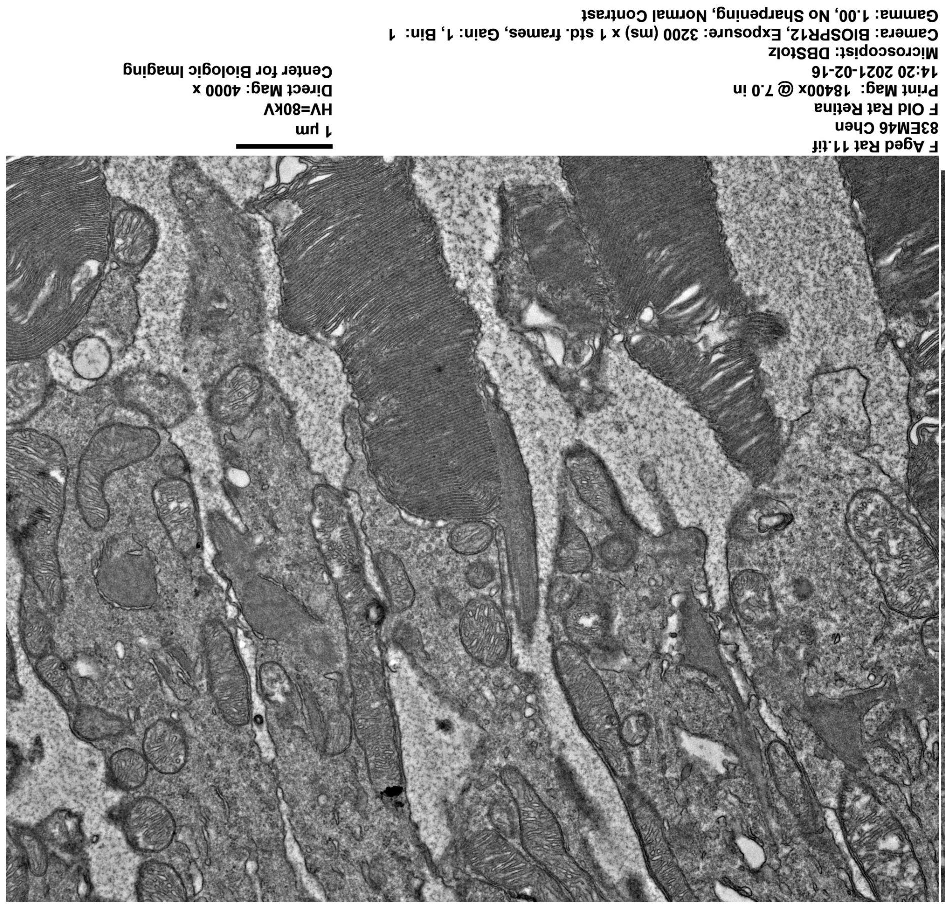
F Aged Rat 11.tif
83EM46 Chen
F Old Rat Retina
Print Mag: 18400x @ 7.0 in
14:20 2021-02-16
Microscopist: DBStolz
Camera: BIOSPR12, Exposure: 3200 (ms) x 1 std. frames, Gain: 1, Bin: 1
Gamma: 1.00, No Sharpening, Normal Contrast
1 µm
HV=80kV
Direct Mag: 4000 x
Center for Biologic Imaging

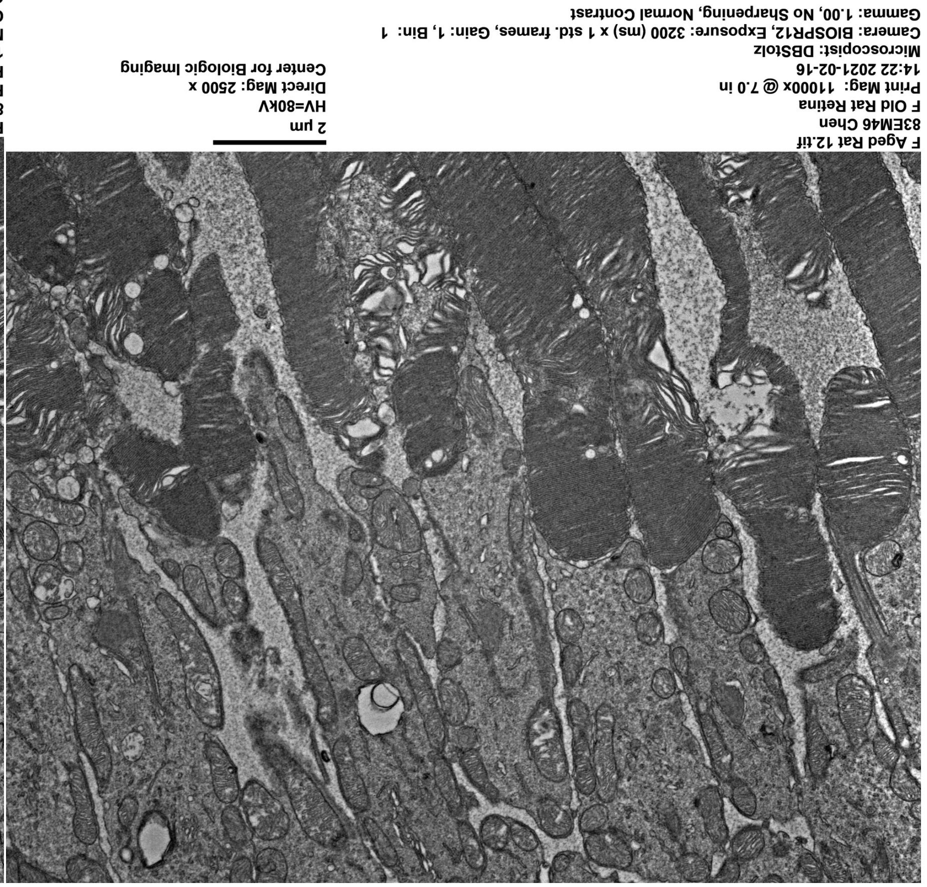
F Aged Rat 12.tif
83EM46 Chen
F Old Rat Retina
Print Mag: 11000x @ 7.0 in
14:22 2021-02-16
Microscopist: DBStolz
Camera: BIOSPR12, Exposure: 3200 (ms) x 1 std. frames, Gain: 1, Bin: 1
Gamma: 1.00, No Sharpening, Normal Contrast
2 µm
HV=80kV
Direct Mag: 2500 x
Center for Biologic Imaging

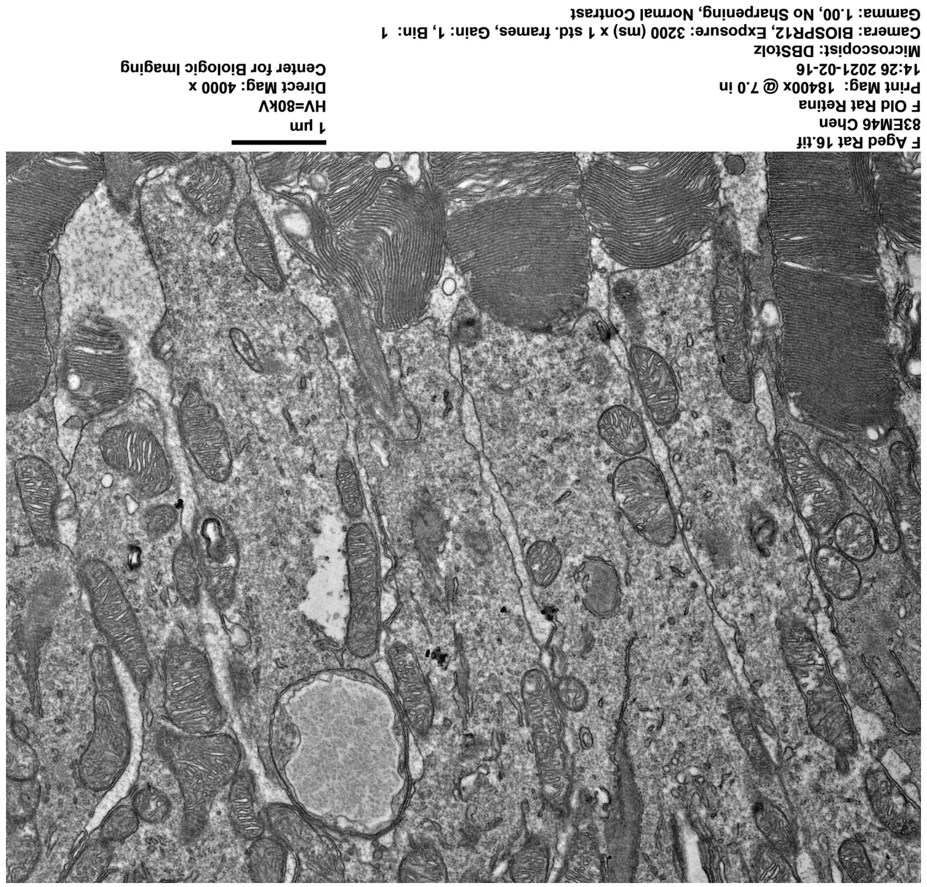
F Aged Rat 16.tif
83EM46 Chen
F Old Rat Retina
Print Mag: 18400x @ 7.0 in
14:26 2021-02-16
Microscopist: DBStolz
Camera: BIOSPR12, Exposure: 3200 (ms) x 1 std. frames, Gain: 1, Bin: 1
Gamma: 1.00, No Sharpening, Normal Contrast
1 µm
HV=80kV
Direct Mag: 4000 x
Center for Biologic Imaging

Old OS_IS

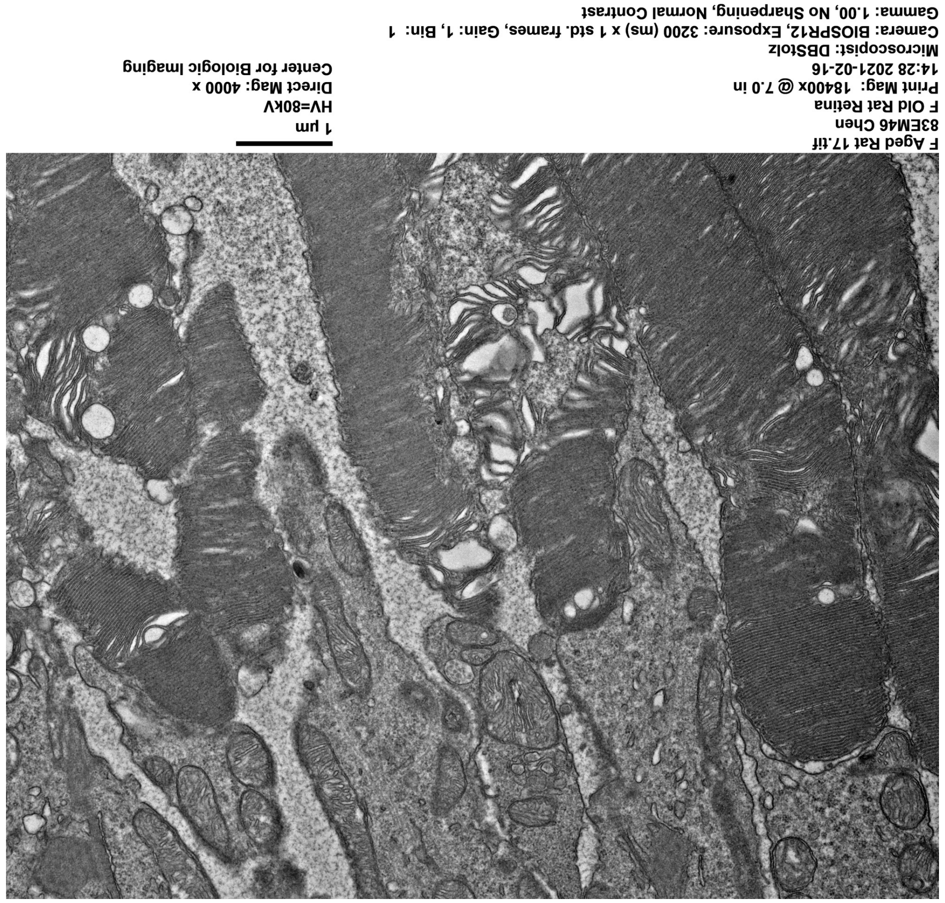
F Aged Rat 17.tif
83EM46 Chen
F Old Rat Retina
Print Mag: 18400x @ 7.0 in
14:28 2021-02-16
Microscopist: DBStolz
Camera: BIOSPR12, Exposure: 3200 (ms) x 1 std. frames, Gain: 1, Bin: 1
Gamma: 1.00, No Sharpening, Normal Contrast
1 µm
HV=80kV
Direct Mag: 4000 x
Center for Biologic Imaging

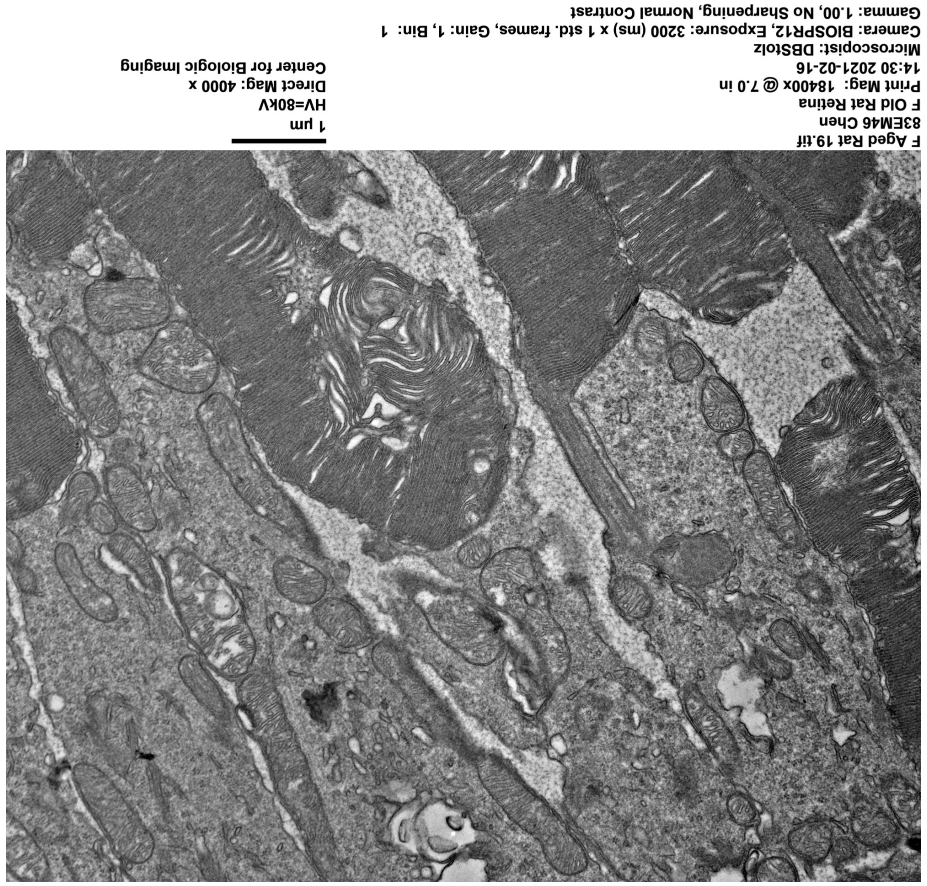
F Aged Rat 19.tif
83EM46 Chen
F Old Rat Retina
Print Mag: 18400x @ 7.0 in
14:30 2021-02-16
Microscopist: DBStolz
Camera: BIOSPR12, Exposure: 3200 (ms) x 1 std. frames, Gain: 1, Bin: 1
Gamma: 1.00, No Sharpening, Normal Contrast
1 µm
HV=80kV
Direct Mag: 4000 x
Center for Biologic Imaging

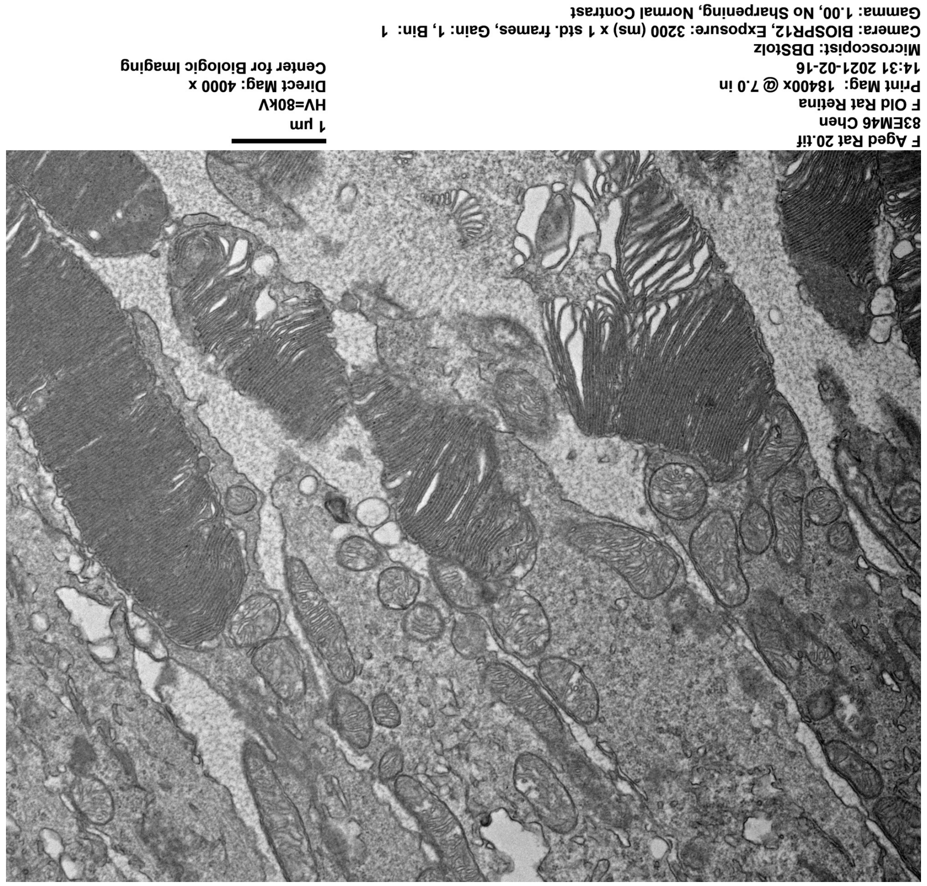
F Aged Rat 20.tif
83EM46 Chen
F Old Rat Retina
Print Mag: 18400x @ 7.0 in
14:31 2021-02-16
Microscopist: DBStolz
Camera: BIOSPR12, Exposure: 3200 (ms) x 1 std. frames, Gain: 1, Bin: 1
Gamma: 1.00, No Sharpening, Normal Contrast
1 µm
HV=80kV
Direct Mag: 4000 x
Center for Biologic Imaging

Old IS

F Aged Rat 23.tif
83EM46 Chen
F Old Rat Retina
Print Mag: 11000x @ 7.0 in
14:35 2021-02-16
Microscopist: DBStolz
Camera: BIOSPR12, Exposure: 3200 (ms) x 1 std. frames, Gain: 1, Bin: 1
Gamma: 1.00, No Sharpening, Normal Contrast

2 µm
HV=80kV
Direct Mag: 2500 x
Center for Biologic Imaging

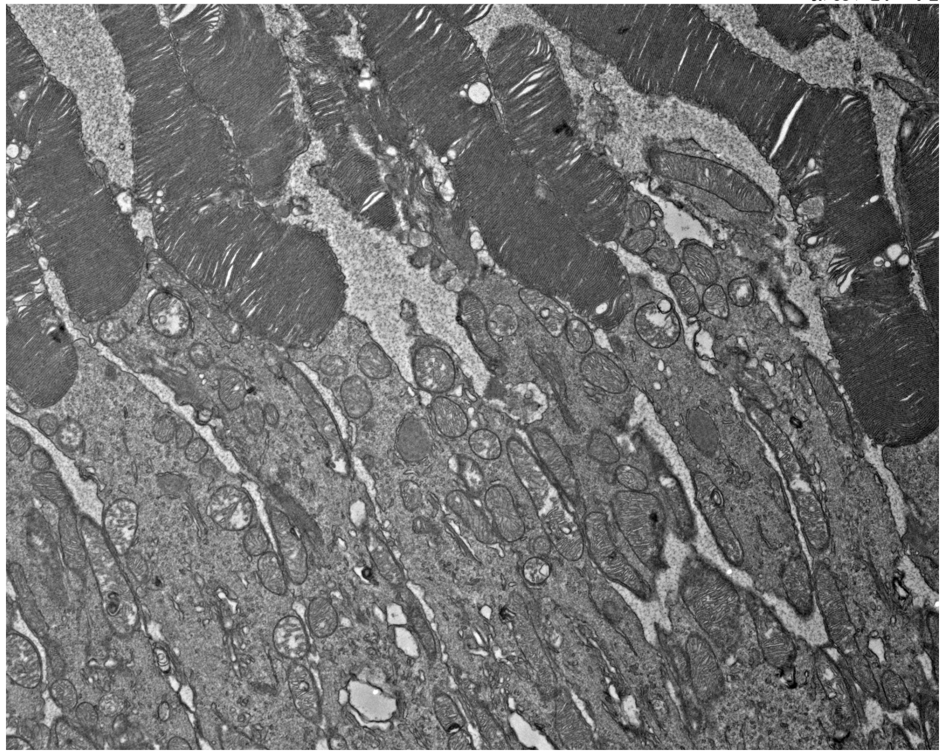

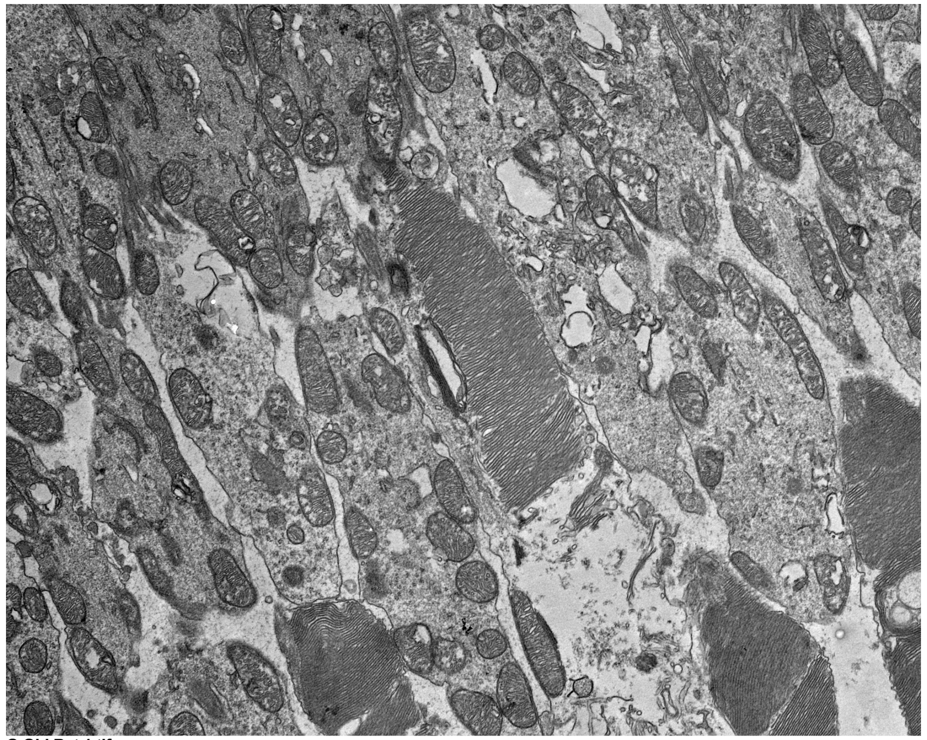

G Old Rat 1.tif
83EM46 Chen
G Old Rat Retina
Print Mag: 11000x @ 7.0 in
15:01 2021-02-16
Microscopist: DBStolz
Camera: BIOSPR12, Exposure: 3200 (ms) x 1 std. frames, Gain: 1, Bin: 1
Gamma: 1.00, No Sharpening, Normal Contrast

2 µm
HV=80kV
Direct Mag: 2500 x
Center for Biologic Imaging

G Old Rat 2.tif
83EM46 Chen
G Old Rat Retina
Print Mag: 18400x @ 7.0 in
15:05 2021-02-16
Microscopist: DBStolz
Camera: BIOSPR12, Exposure: 3200 (ms) x 1 std. frames, Gain: 1, Bin: 1
Gamma: 1.00, No Sharpening, Normal Contrast

1 µm
HV=80kV
Direct Mag: 4000 x
Center for Biologic Imaging

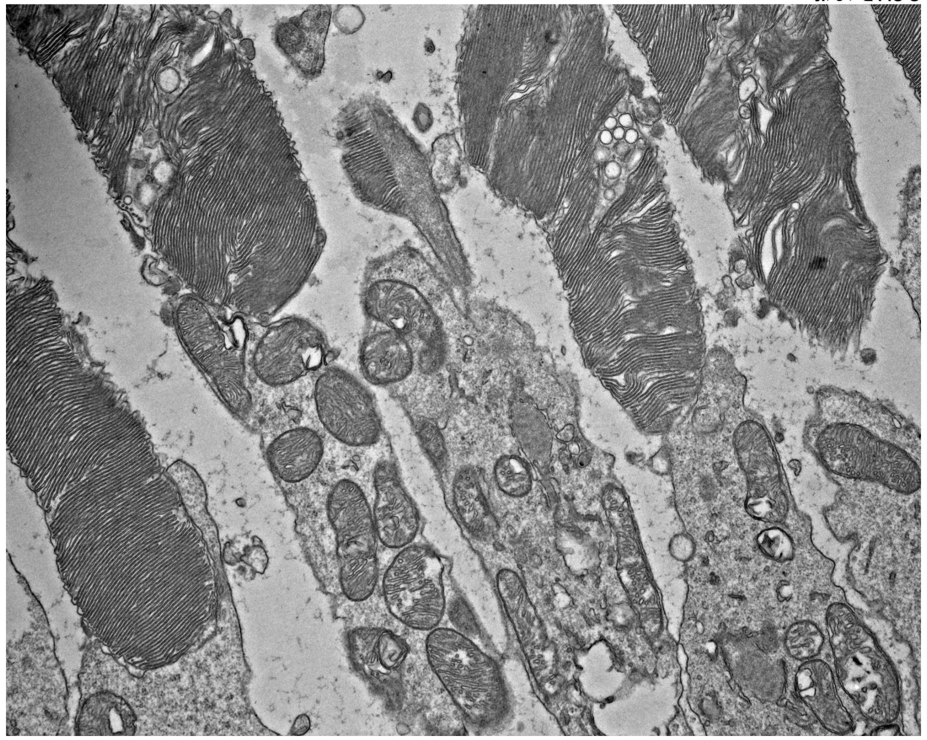

Old IS

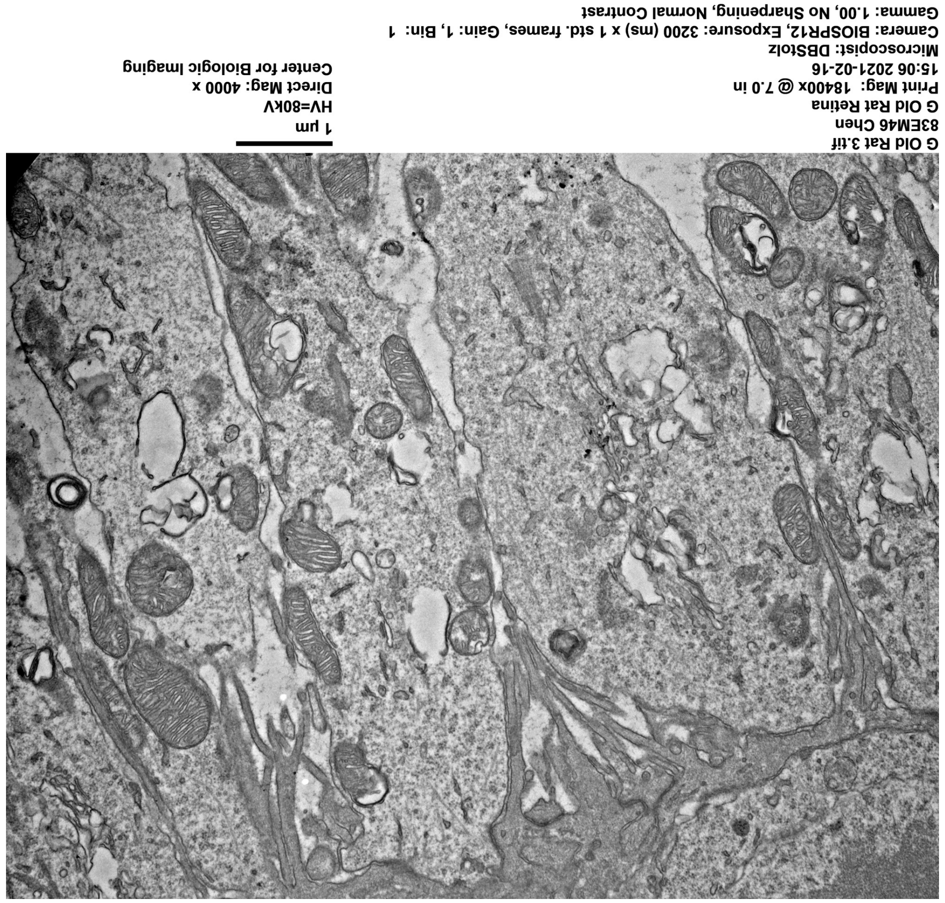

G Old Rat 3.tif
83EM46 Chen
G Old Rat Retina
Print Mag: 18400x @ 7.0 in
15:06 2021-02-16
Microscopist: DBStolz
Camera: BIOSPR12, Exposure: 3200 (ms) x 1 std. frames, Gain: 1, Bin: 1
Gamma: 1.00, No Sharpening, Normal Contrast
1 µm
HV=80kV
Direct Mag: 4000 x
Center for Biologic Imaging

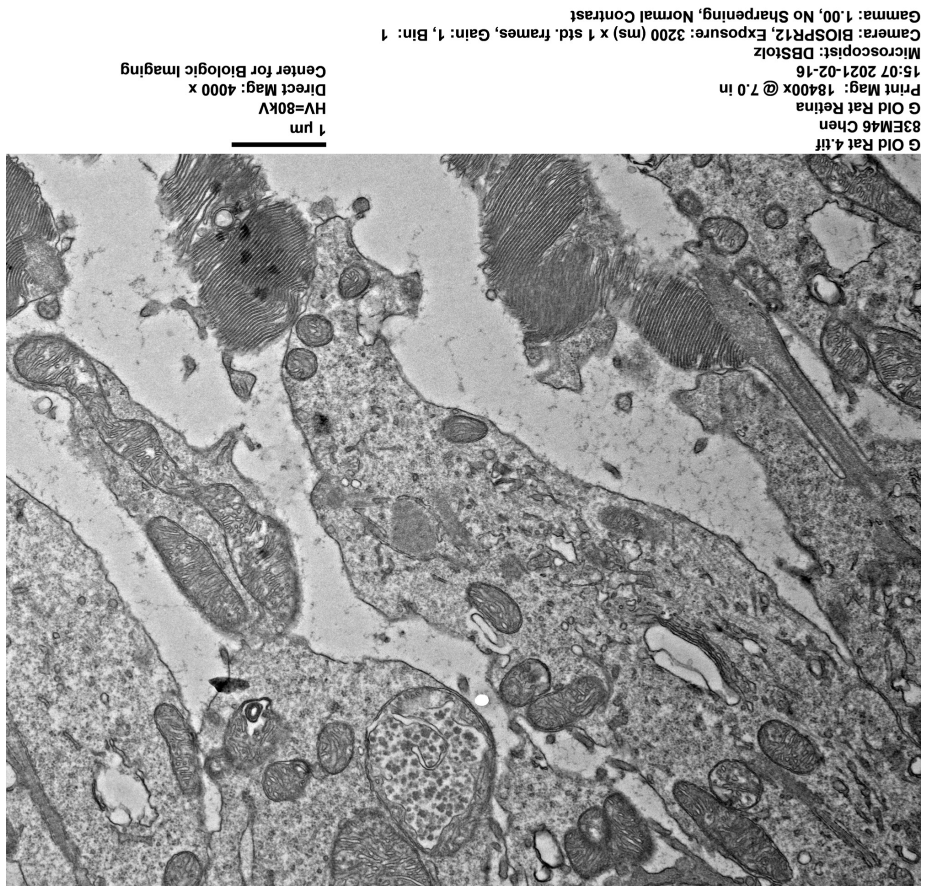

G Old Rat 4.tif
83EM46 Chen
G Old Rat Retina
Print Mag: 18400x @ 7.0 in
15:07 2021-02-16
Microscopist: DBStolz
Camera: BIOSPR12, Exposure: 3200 (ms) x 1 std. frames, Gain: 1, Bin: 1
Gamma: 1.00, No Sharpening, Normal Contrast
1 µm
HV=80kV
Direct Mag: 4000 x
Center for Biologic Imaging

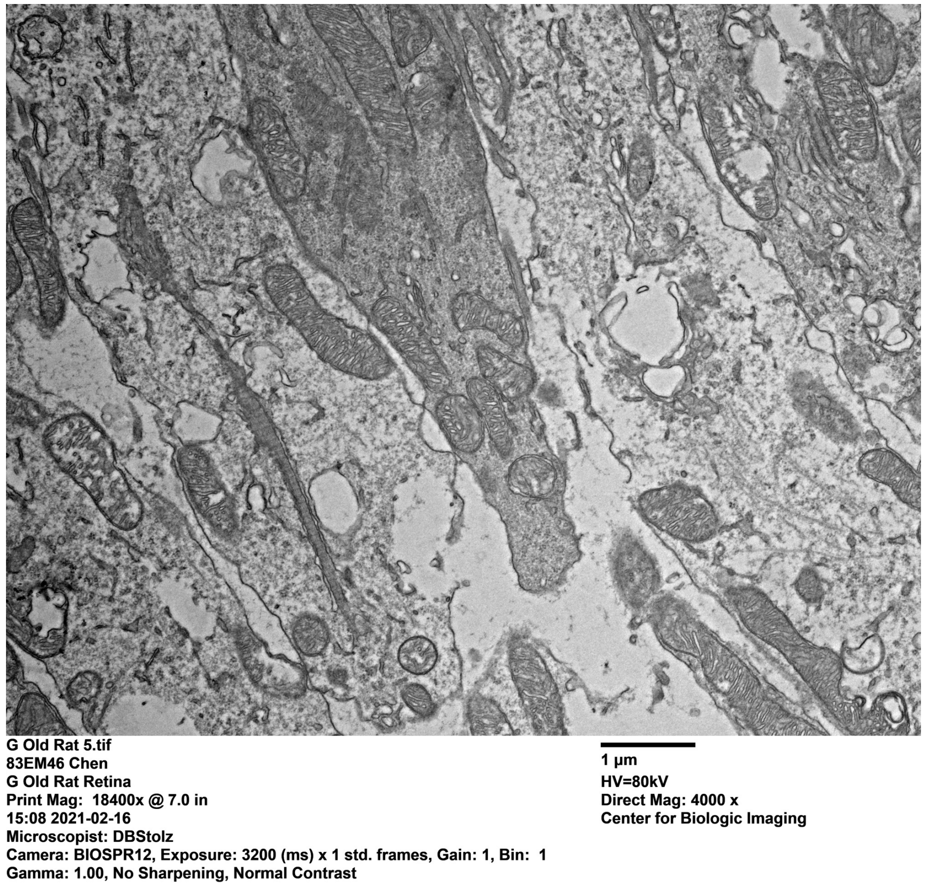

G Old Rat 5.tif
83EM46 Chen
G Old Rat Retina
Print Mag: 18400x @ 7.0 in
15:08 2021-02-16
Microscopist: DBStolz
Camera: BIOSPR12, Exposure: 3200 (ms) x 1 std. frames, Gain: 1, Bin: 1
Gamma: 1.00, No Sharpening, Normal Contrast
1 µm
HV=80kV
Direct Mag: 4000 x
Center for Biologic Imaging

Old IS

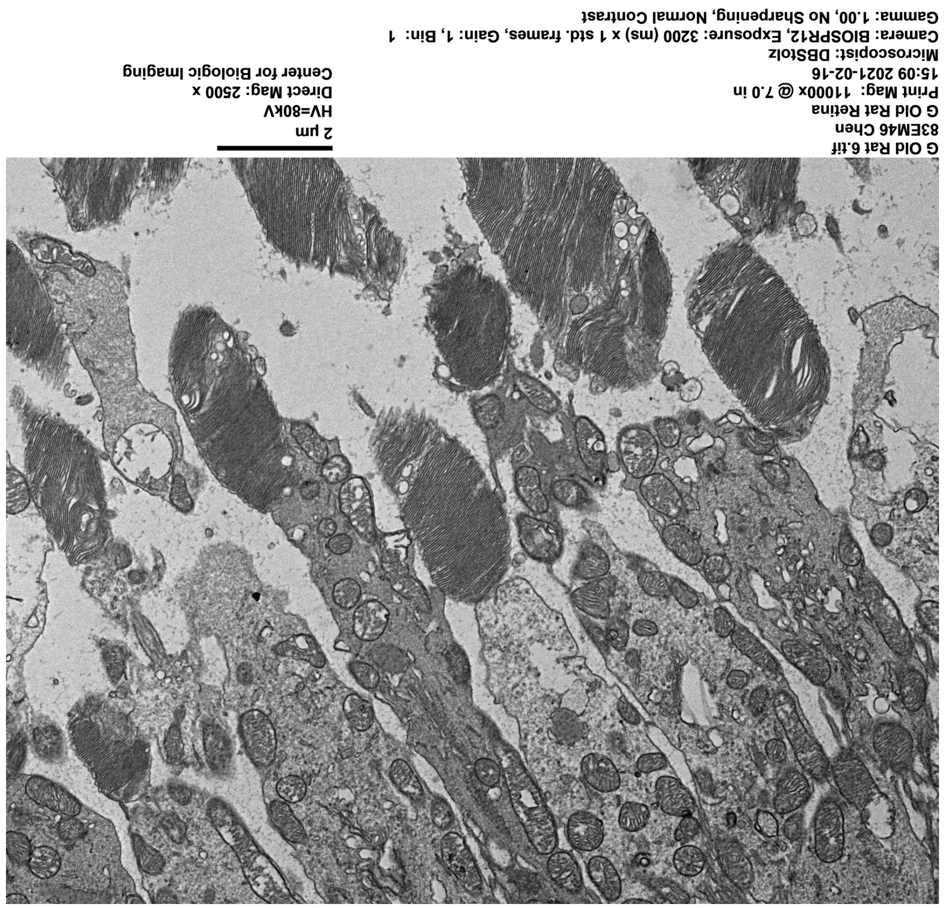
G Old Rat 6.tif
83EM46 Chen
G Old Rat Retina
Print Mag: 11000x @ 7.0 in
15:09 2021-02-16
Microscopist: DBStolz
Camera: BIOSPR12, Exposure: 3200 (ms) x 1 std. frames, Gain: 1, Bin: 1
Gamma: 1.00, No Sharpening, Normal Contrast
2 µm
HV=80kV
Direct Mag: 2500 x
Center for Biologic Imaging

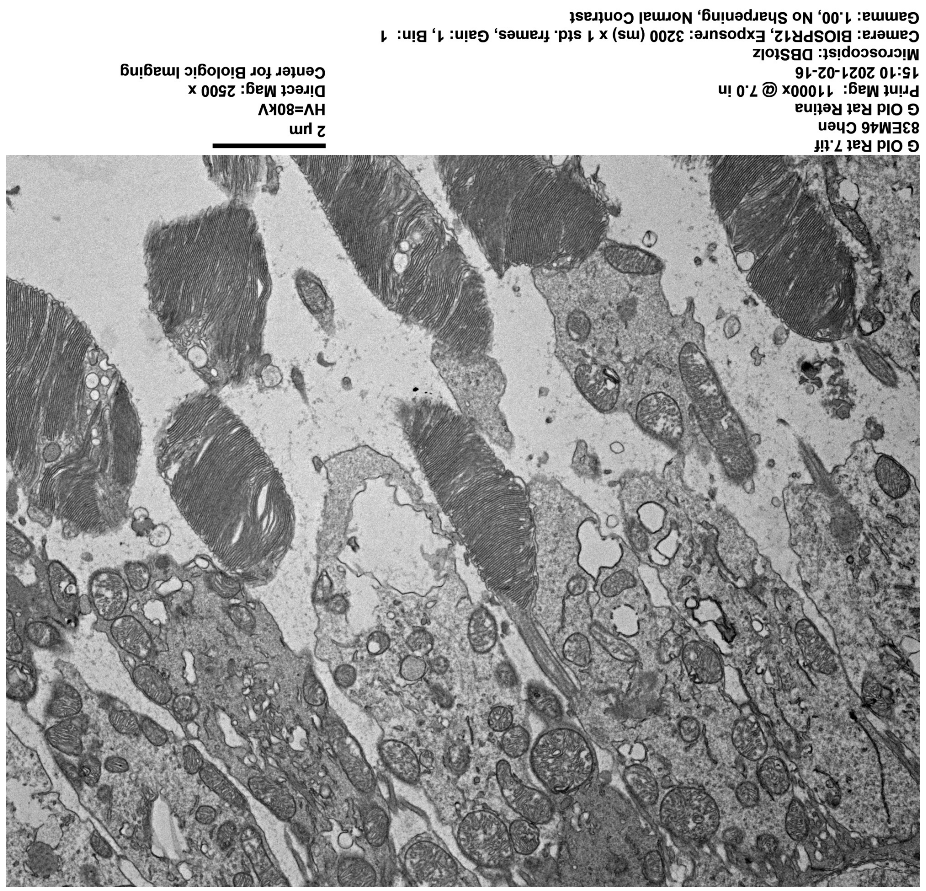
G Old Rat 7.tif
83EM46 Chen
G Old Rat Retina
Print Mag: 11000x @ 7.0 in
15:10 2021-02-16
Microscopist: DBStolz
Camera: BIOSPR12, Exposure: 3200 (ms) x 1 std. frames, Gain: 1, Bin: 1
Gamma: 1.00, No Sharpening, Normal Contrast
2 µm
HV=80kV
Direct Mag: 2500 x
Center for Biologic Imaging

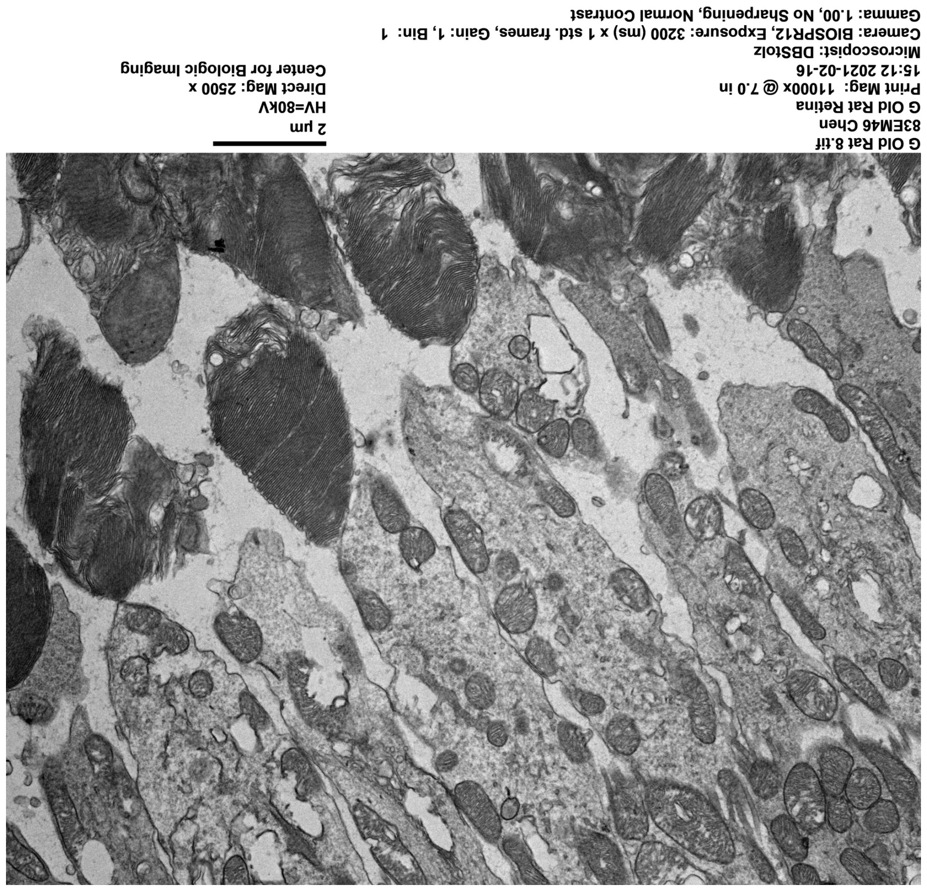
G Old Rat 8.tif
83EM46 Chen
G Old Rat Retina
Print Mag: 11000x @ 7.0 in
15:12 2021-02-16
Microscopist: DBStolz
Camera: BIOSPR12, Exposure: 3200 (ms) x 1 std. frames, Gain: 1, Bin: 1
Gamma: 1.00, No Sharpening, Normal Contrast
2 µm
HV=80kV
Direct Mag: 2500 x
Center for Biologic Imaging

Old IS

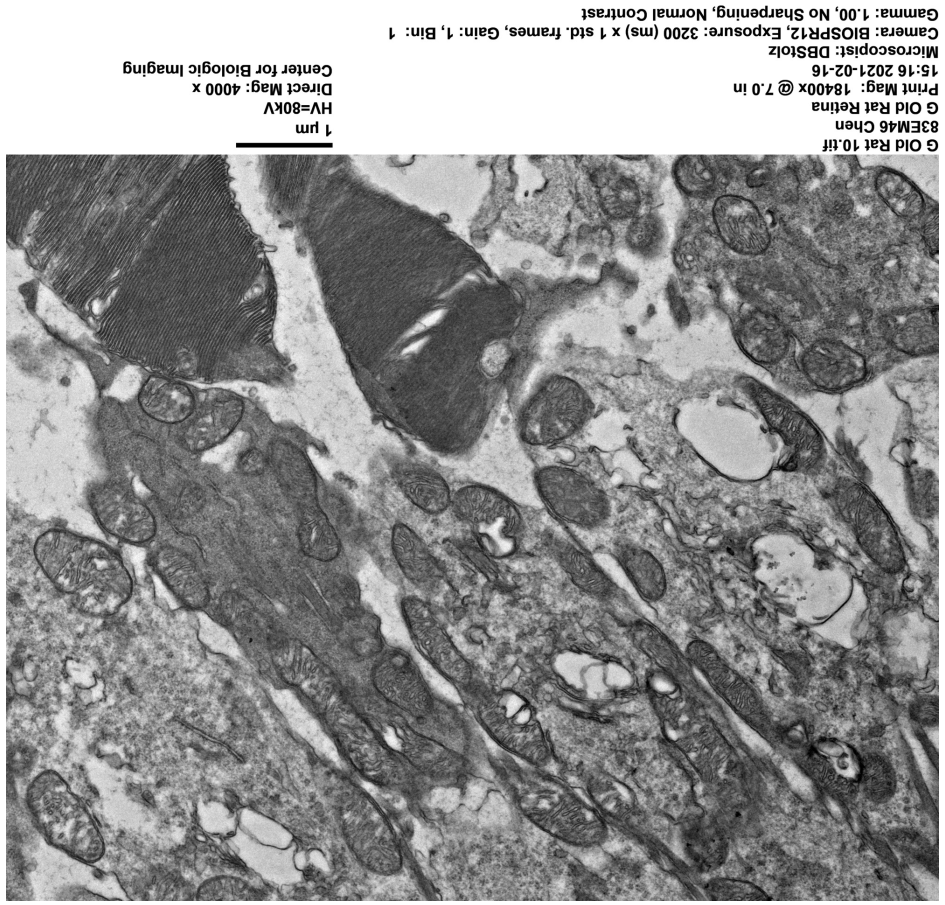
G Old Rat 10.tif
83EM46 Chen
G Old Rat Retina
Print Mag: 18400x @ 7.0 in
15:16 2021-02-16
Microscopist: DBStolz
Camera: BIOSPR12, Exposure: 3200 (ms) x 1 std. frames, Gain: 1, Bin: 1
Gamma: 1.00, No Sharpening, Normal Contrast
1 µm
HV=80kV
Direct Mag: 4000 x
Center for Biologic Imaging

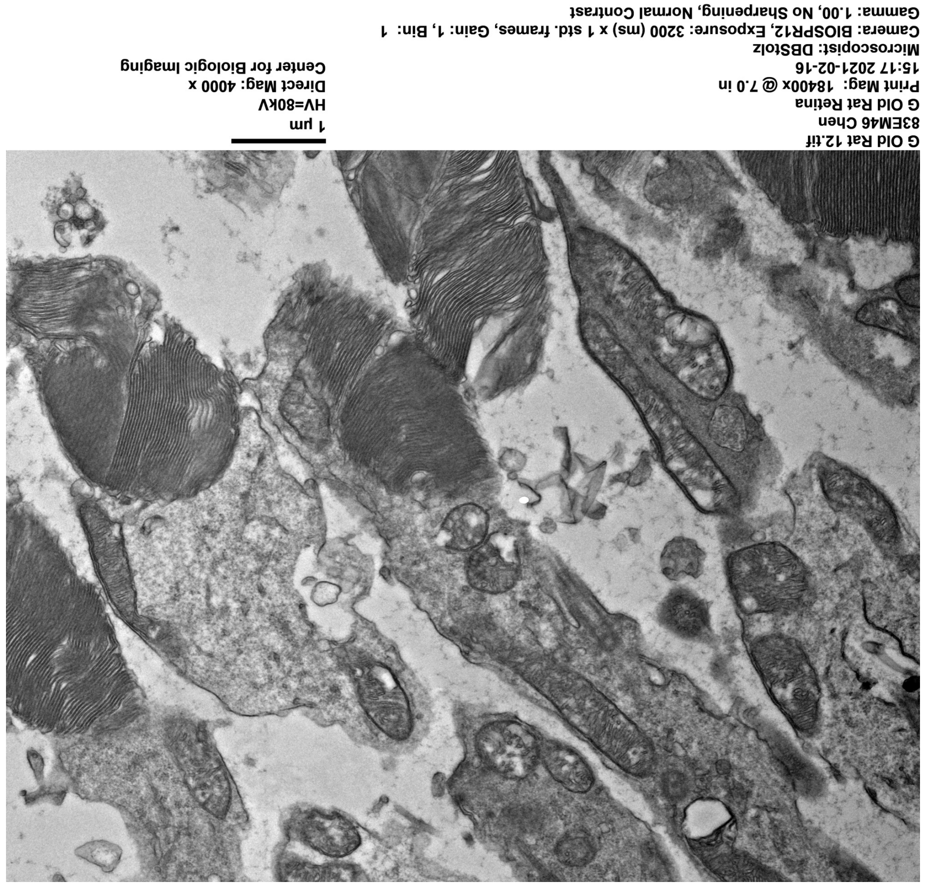
G Old Rat 12.tif
83EM46 Chen
G Old Rat Retina
Print Mag: 18400x @ 7.0 in
15:17 2021-02-16
Microscopist: DBStolz
Camera: BIOSPR12, Exposure: 3200 (ms) x 1 std. frames, Gain: 1, Bin: 1
Gamma: 1.00, No Sharpening, Normal Contrast
1 µm
HV=80kV
Direct Mag: 4000 x
Center for Biologic Imaging

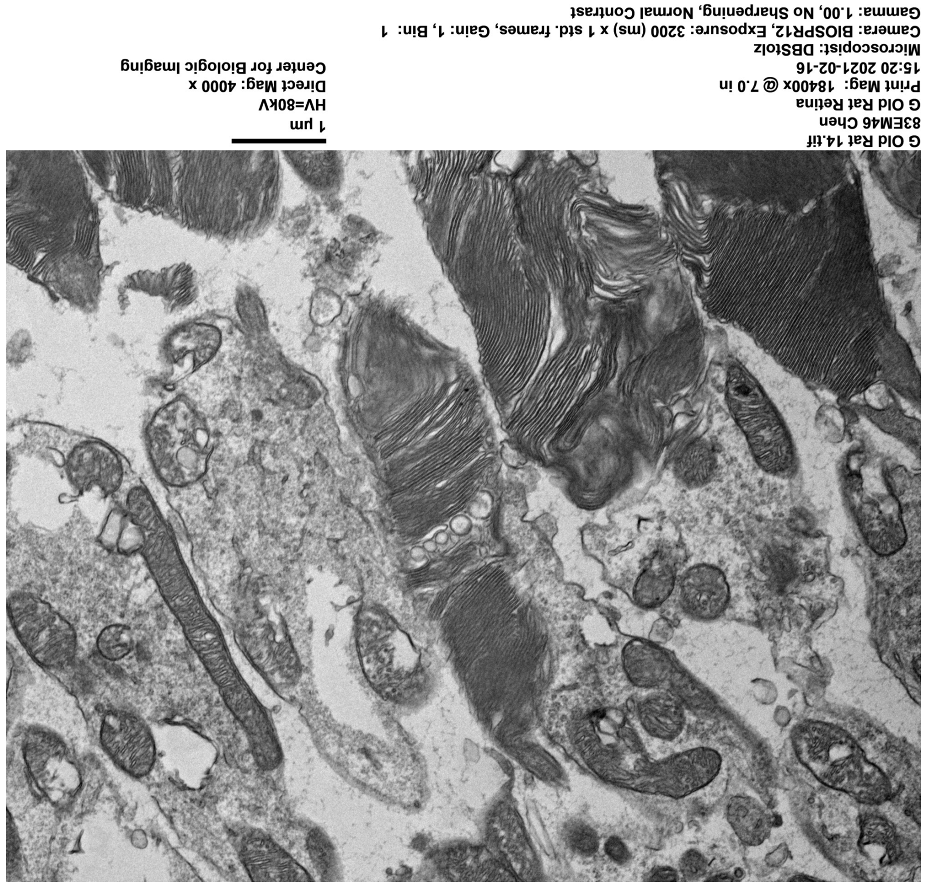
G Old Rat 14.tif
83EM46 Chen
G Old Rat Retina
Print Mag: 18400x @ 7.0 in
15:20 2021-02-16
Microscopist: DBStolz
Camera: BIOSPR12, Exposure: 3200 (ms) x 1 std. frames, Gain: 1, Bin: 1
Gamma: 1.00, No Sharpening, Normal Contrast
1 µm
HV=80kV
Direct Mag: 4000 x
Center for Biologic Imaging

Old IS

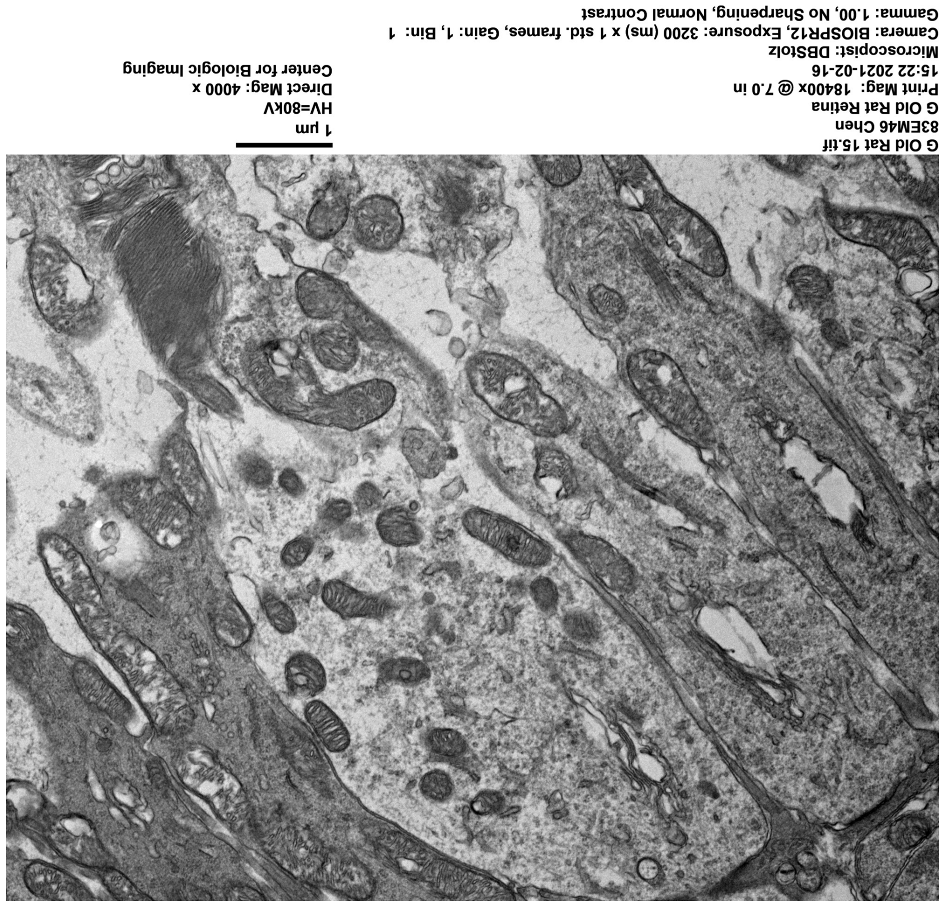
G Old Rat 15.tif
83EM46 Chen
G Old Rat Retina
Print Mag: 18400x @ 7.0 in
15:22 2021-02-16
Microscopist: DBStolz
Camera: BIOSPR12, Exposure: 3200 (ms) x 1 std. frames, Gain: 1, Bin: 1
Gamma: 1.00, No Sharpening, Normal Contrast
1 µm
HV=80kV
Direct Mag: 4000 x
Center for Biologic Imaging

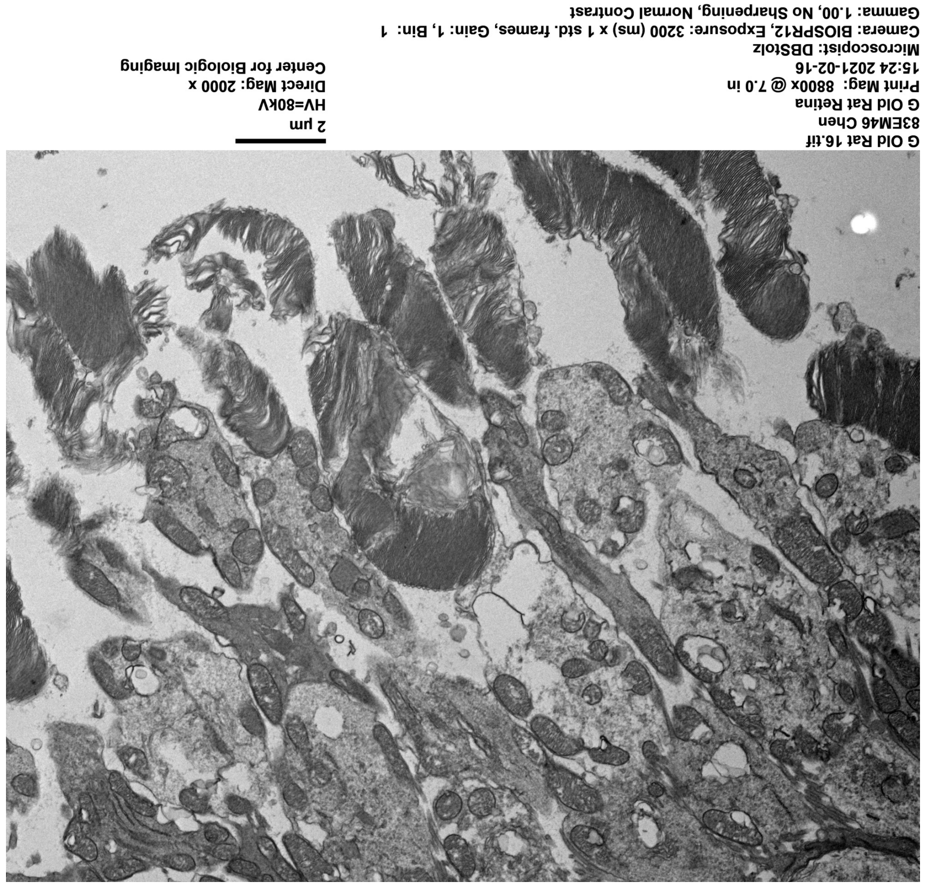
G Old Rat 16.tif
83EM46 Chen
G Old Rat Retina
Print Mag: 8800x @ 7.0 in
15:24 2021-02-16
Microscopist: DBStolz
Camera: BIOSPR12, Exposure: 3200 (ms) x 1 std. frames, Gain: 1, Bin: 1
Gamma: 1.00, No Sharpening, Normal Contrast
2 µm
HV=80kV
Direct Mag: 2000 x
Center for Biologic Imaging

8-AG peripheral RPE low mag

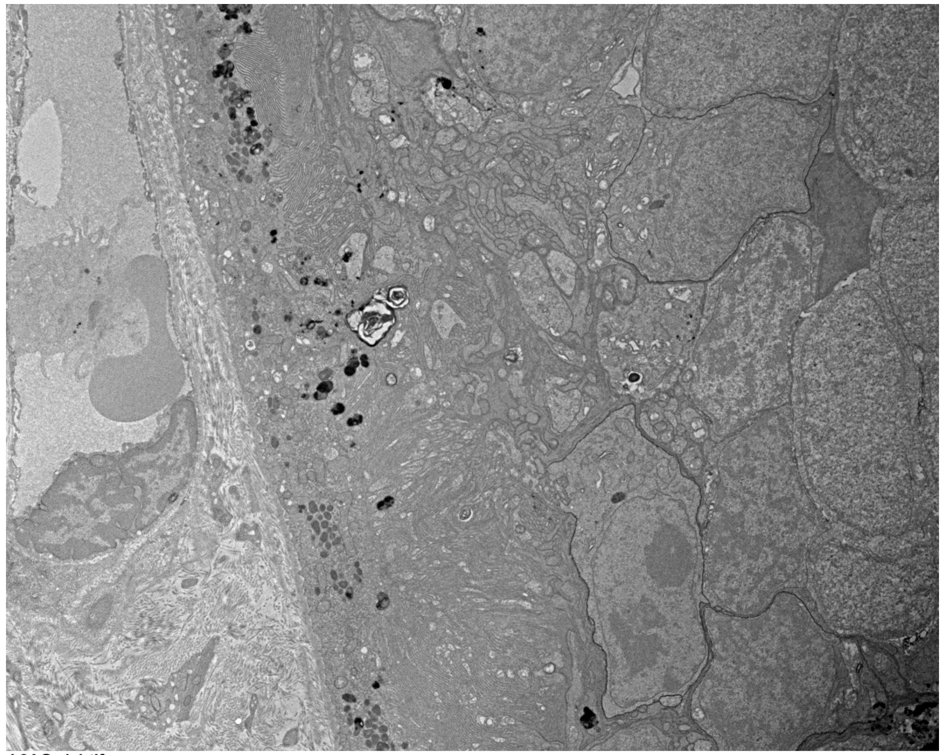

J 8AG -1 1.tif
83EM46 Chen
J 8AG 2 Rat Retina
Print Mag: 5280x @ 7.0 in
10:18 2021-02-08
Microscopist: DBStolz
Camera: BIOSPR12, Exposure: 3200 (ms) x 1 std. frames, Gain: 1, Bin: 1
Gamma: 1.15, No Sharpening, Normal Contrast

4 µm
HV=80kV
Direct Mag: 1200 x
Center for Biologic Imaging

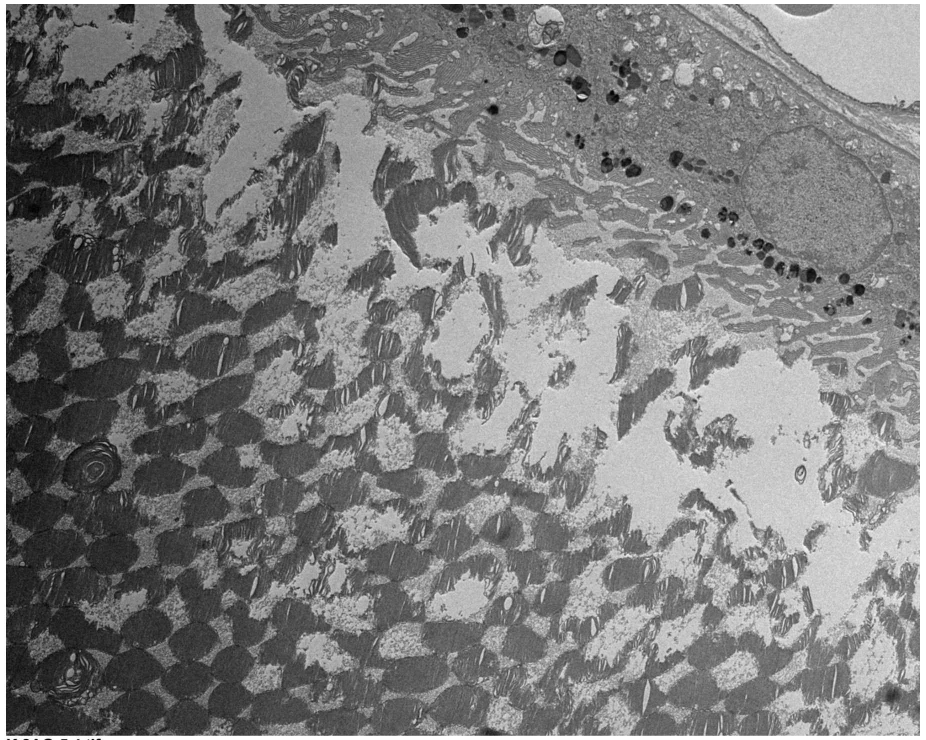

K 8AG-5 4.tif
83EM46 Chen
K 8AG-5
Print Mag: 4400x @ 7.0 in
10:50 2021-02-08
Microscopist: DBStolz
Camera: BIOSPR12, Exposure: 3200 (ms) x 1 std. frames, Gain: 1, Bin: 1
Gamma: 1.15, No Sharpening, Normal Contrast

4 µm
HV=80kV
Direct Mag: 1000 x
Center for Biologic Imaging

8-AG RPE

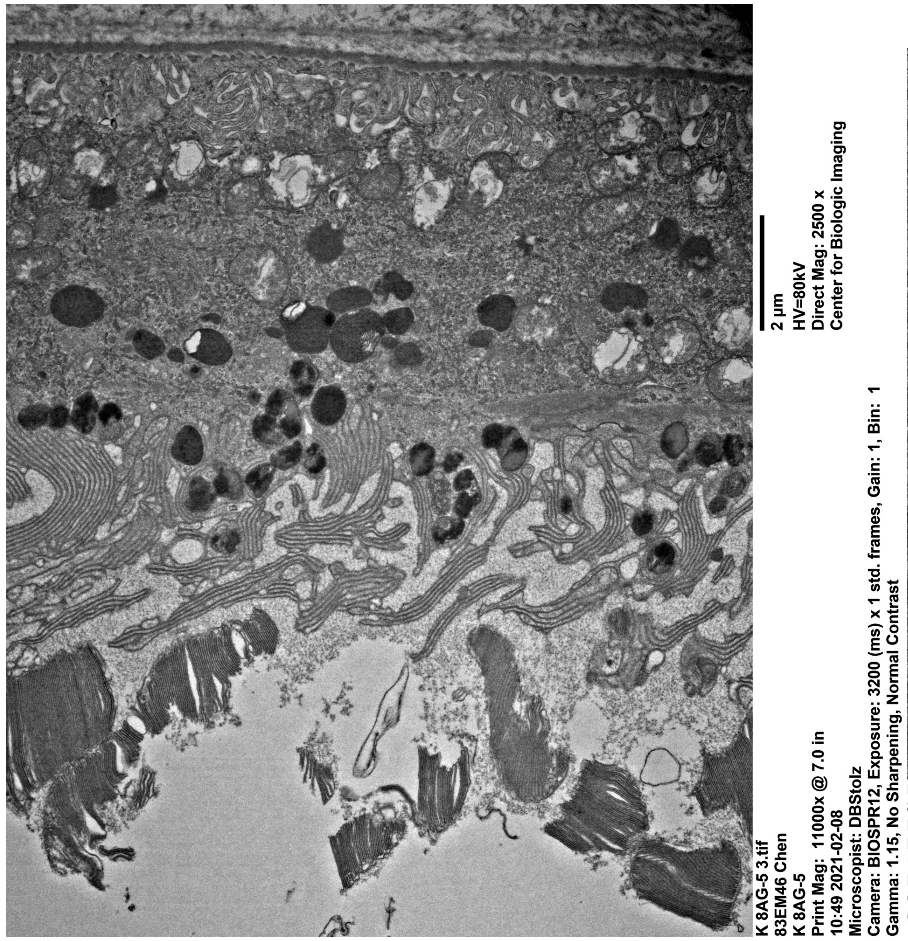
2 µm
HV=80kV
Direct Mag: 2500 x
Center for Biologic Imaging
K 8AG-5 3.tif
83EM46 Chen
K 8AG-5
Print Mag: 11000x @ 7.0 in
10:49 2021-02-08
Microscopist: DBStolz
Camera: BIOSPR12, Exposure: 3200 (ms) x 1 std. frames, Gain: 1, Bin: 1
Gamma: 1.15, No Sharpening, Normal Contrast

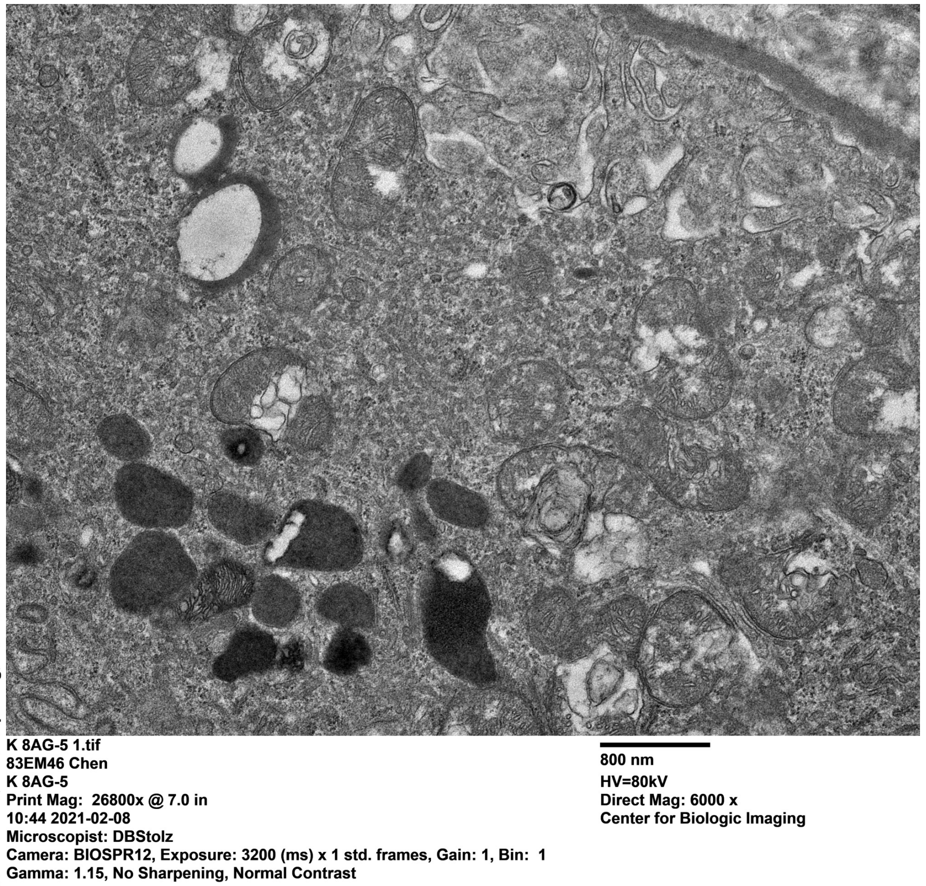
K 8AG-5 1.tif
83EM46 Chen
K 8AG-5
Print Mag: 26800x @ 7.0 in
10:44 2021-02-08
Microscopist: DBStolz
Camera: BIOSPR12, Exposure: 3200 (ms) x 1 std. frames, Gain: 1, Bin: 1
Gamma: 1.15, No Sharpening, Normal Contrast
800 nm
HV=80kV
Direct Mag: 6000 x
Center for Biologic Imaging

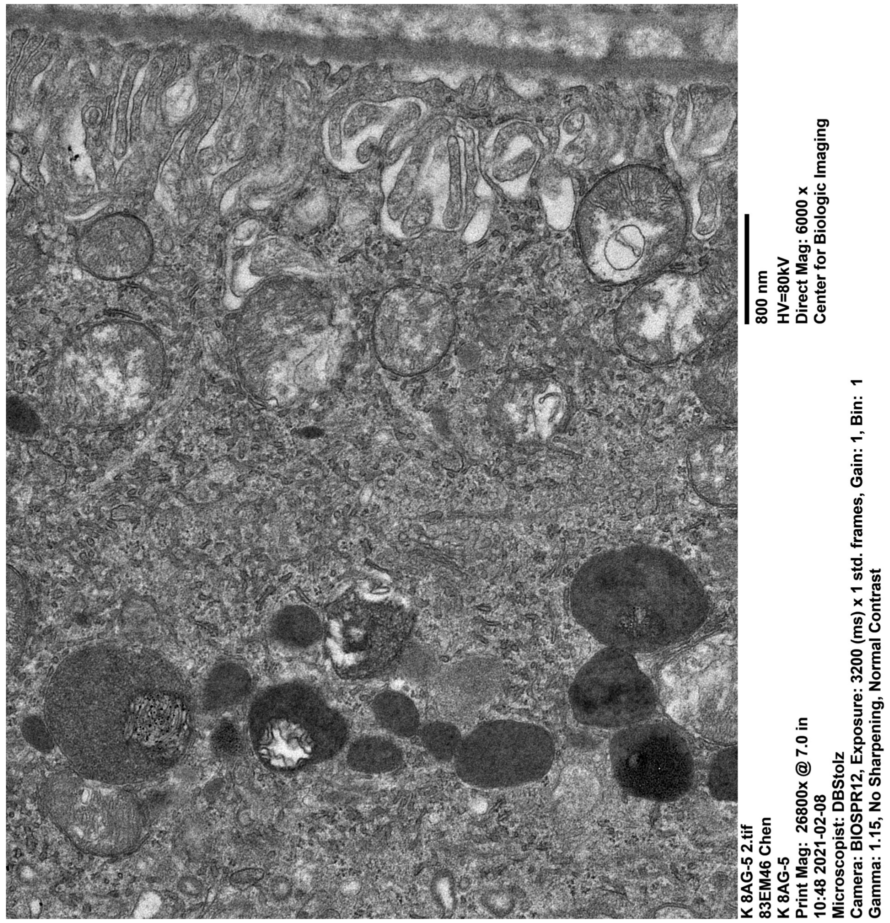
800 nm
HV=80kV
Direct Mag: 6000 x
Center for Biologic Imaging
K 8AG-5 2.tif
83EM46 Chen
K 8AG-5
Print Mag: 26800x @ 7.0 in
10:48 2021-02-08
Microscopist: DBStolz
Camera: BIOSPR12, Exposure: 3200 (ms) x 1 std. frames, Gain: 1, Bin: 1
Gamma: 1.15, No Sharpening, Normal Contrast

8-AG RPE

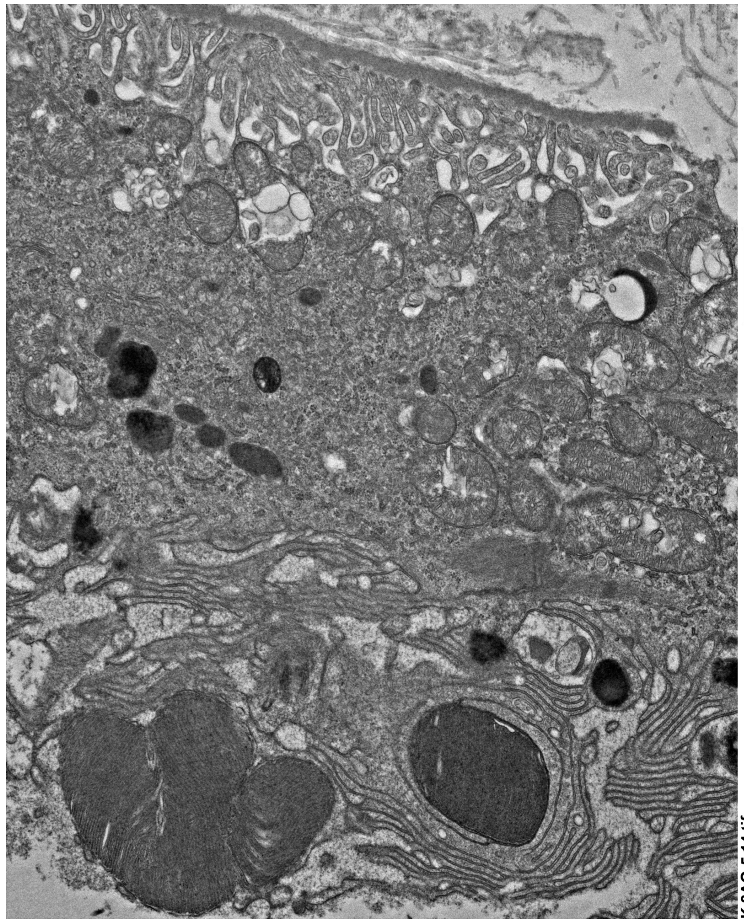
1 µm
HV=80kV
Direct Mag: 4000 x
Center for Biologic Imaging
K 8AG-5 14.tif
83EM46 Chen
K 8AG-5
Print Mag: 18400x @ 7.0 in
11:10 2021-02-08
Microscopist: DBStolz
Camera: BIOSPR12, Exposure: 3200 (ms) x 1 std. frames, Gain: 1, Bin: 1
Gamma: 1.15, No Sharpening, Normal Contrast

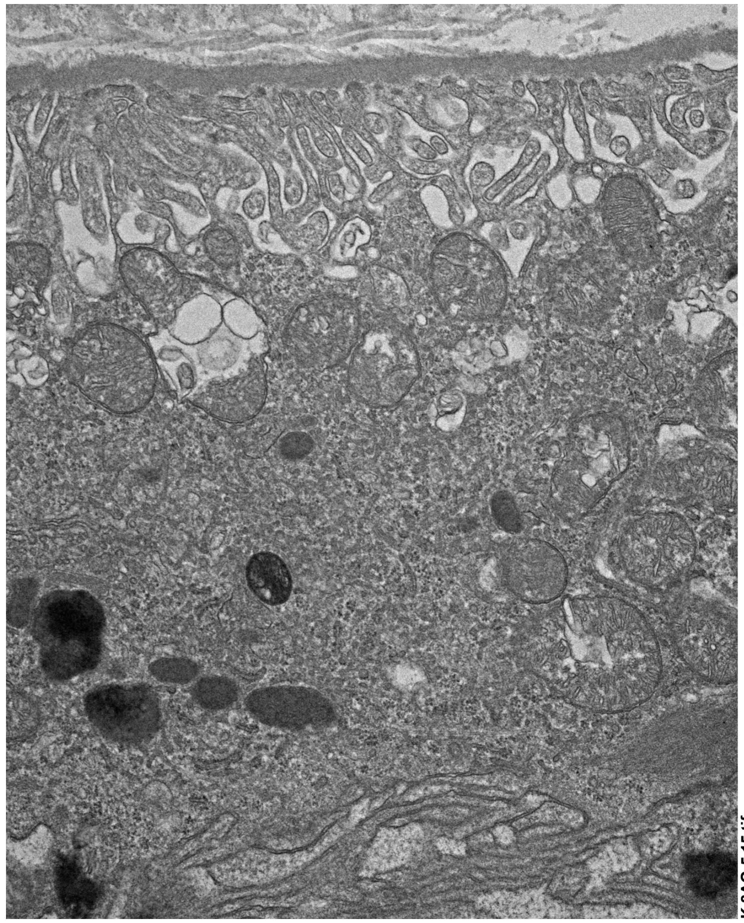
800 nm
HV=80kV
Direct Mag: 6000 x
Center for Biologic Imaging
K 8AG-5 15.tif
83EM46 Chen
K 8AG-5
Print Mag: 26800x @ 7.0 in
11:11 2021-02-08
Microscopist: DBStolz
Camera: BIOSPR12, Exposure: 3200 (ms) x 1 std. frames, Gain: 1, Bin: 1
Gamma: 1.15, No Sharpening, Normal Contrast

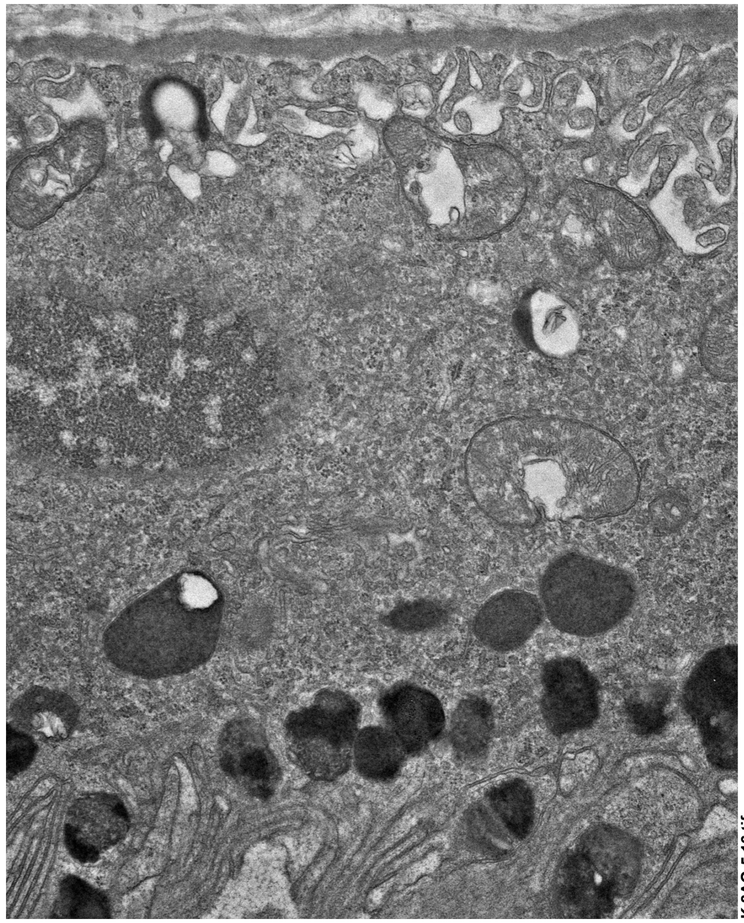
800 nm
HV=80kV
Direct Mag: 6000 x
Center for Biologic Imaging
K 8AG-5 16.tif
83EM46 Chen
K 8AG-5
Print Mag: 26800x @ 7.0 in
11:13 2021-02-08
Microscopist: DBStolz
Camera: BIOSPR12, Exposure: 3200 (ms) x 1 std. frames, Gain: 1, Bin: 1
Gamma: 1.15, No Sharpening, Normal Contrast

8-AG OS

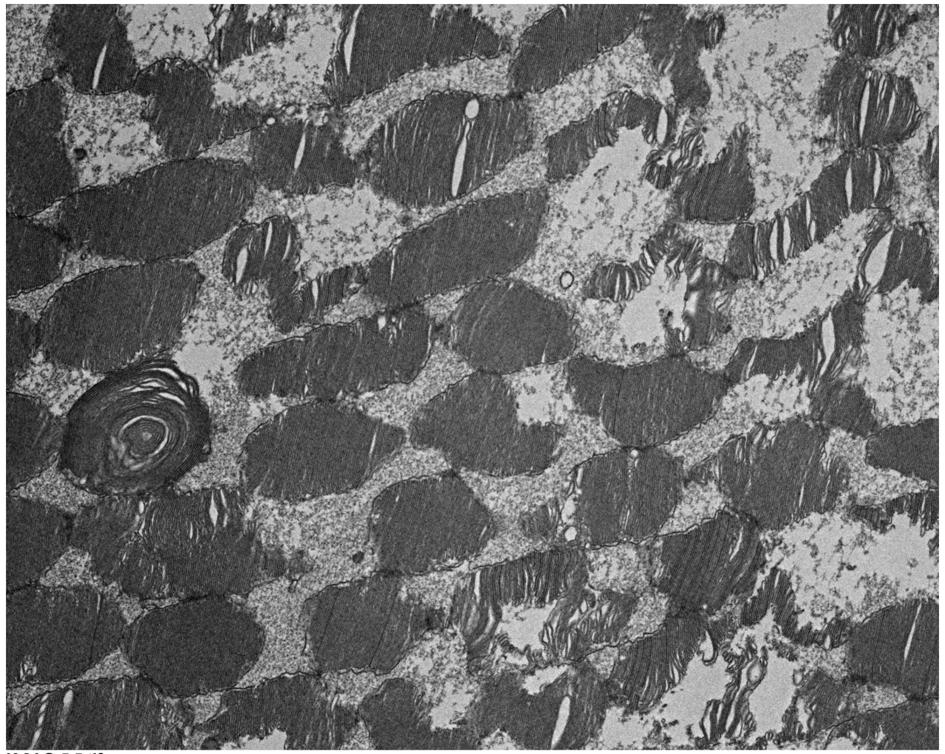

K 8AG-5 5.tif
83EM46 Chen
K 8AG-5
Print Mag: 11000x @ 7.0 in
10:53 2021-02-08
Microscopist: DBStolz
Camera: BIOSPR12, Exposure: 3200 (ms) x 1 std. frames, Gain: 1, Bin: 1
Gamma: 1.15, No Sharpening, Normal Contrast

2 μm
HV=80kV
Direct Mag: 2500 x
Center for Biologic Imaging

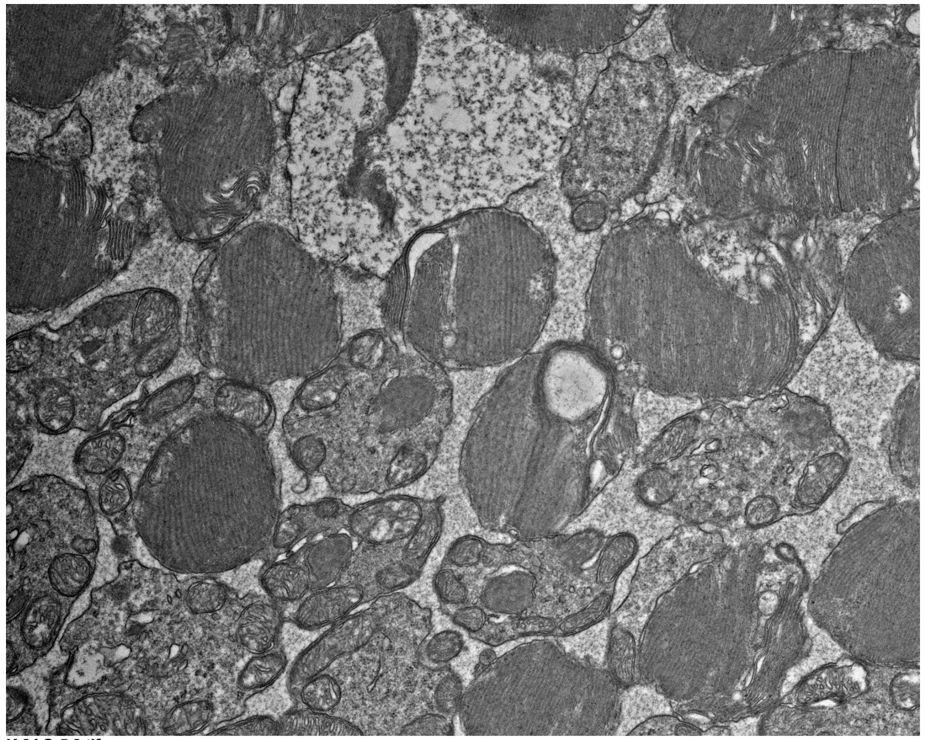

K 8AG-5 8.tif
83EM46 Chen
K 8AG-5
Print Mag: 18400x @ 7.0 in
10:56 2021-02-08
Microscopist: DBStolz
Camera: BIOSPR12, Exposure: 3200 (ms) x 1 std. frames, Gain: 1, Bin: 1
Gamma: 1.15, No Sharpening, Normal Contrast

1 μm
HV=80kV
Direct Mag: 4000 x
Center for Biologic Imaging

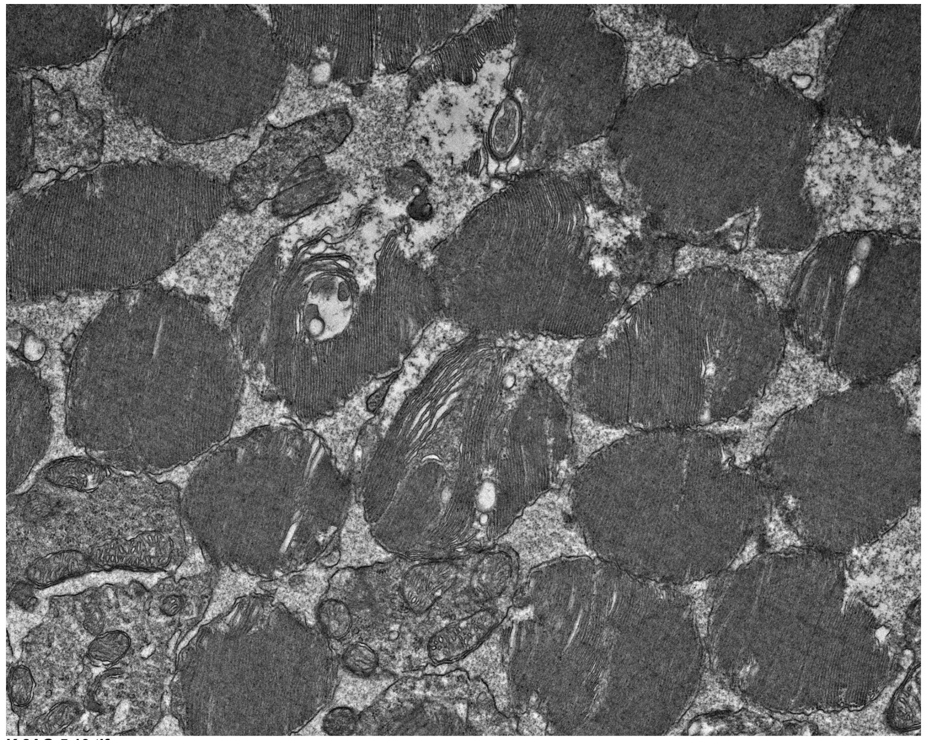

K 8AG-5 10.tif
83EM46 Chen
K 8AG-5
Print Mag: 18400x @ 7.0 in
11:00 2021-02-08
Microscopist: DBStolz
Camera: BIOSPR12, Exposure: 3200 (ms) x 1 std. frames, Gain: 1, Bin: 1
Gamma: 1.15, No Sharpening, Normal Contrast

1 μm
HV=80kV
Direct Mag: 4000 x
Center for Biologic Imaging

8-AG IS

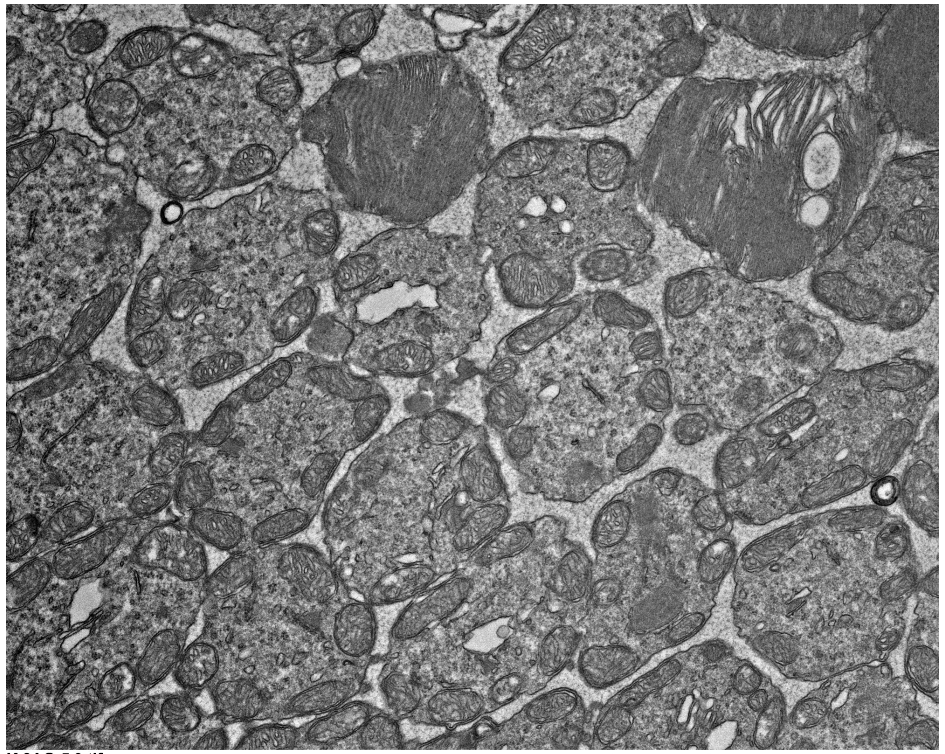

K 8AG-5 9.tif
83EM46 Chen
K 8AG-5
Print Mag: 18400x @ 7.0 in
10:57 2021-02-08
Microscopist: DBStolz
Camera: BIOSPR12, Exposure: 3200 (ms) x 1 std. frames, Gain: 1, Bin: 1
Gamma: 1.15, No Sharpening, Normal Contrast

1 µm
HV=80kV
Direct Mag: 4000 x
Center for Biologic Imaging

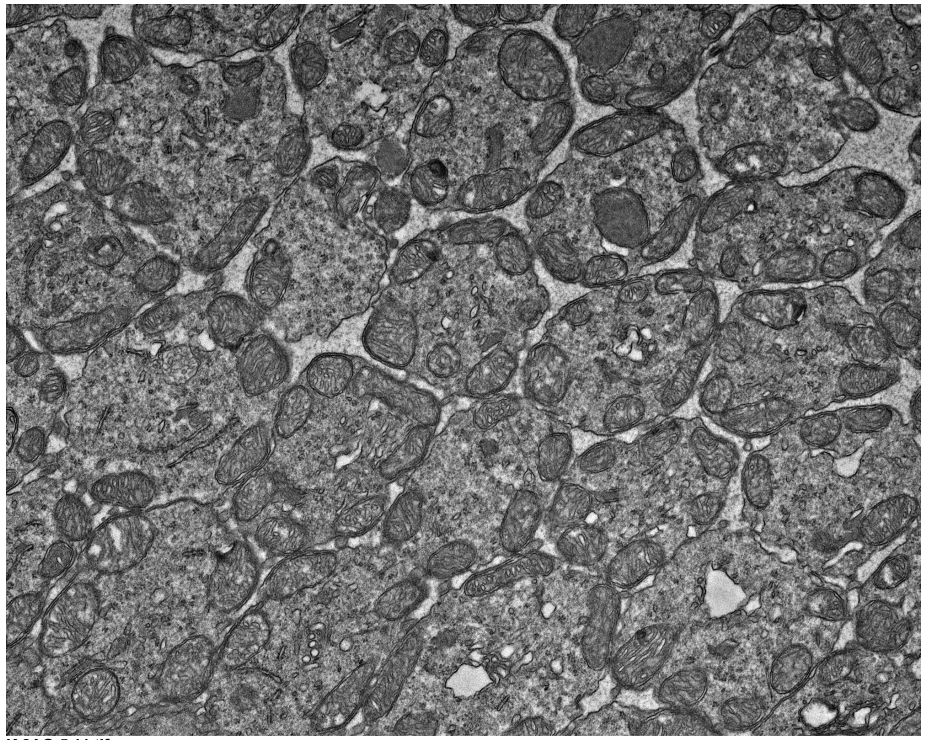

K 8AG-5 11.tif
83EM46 Chen
K 8AG-5
Print Mag: 18400x @ 7.0 in
11:02 2021-02-08
Microscopist: DBStolz
Camera: BIOSPR12, Exposure: 3200 (ms) x 1 std. frames, Gain: 1, Bin: 1
Gamma: 1.15, No Sharpening, Normal Contrast

1 µm
HV=80kV
Direct Mag: 4000 x
Center for Biologic Imaging

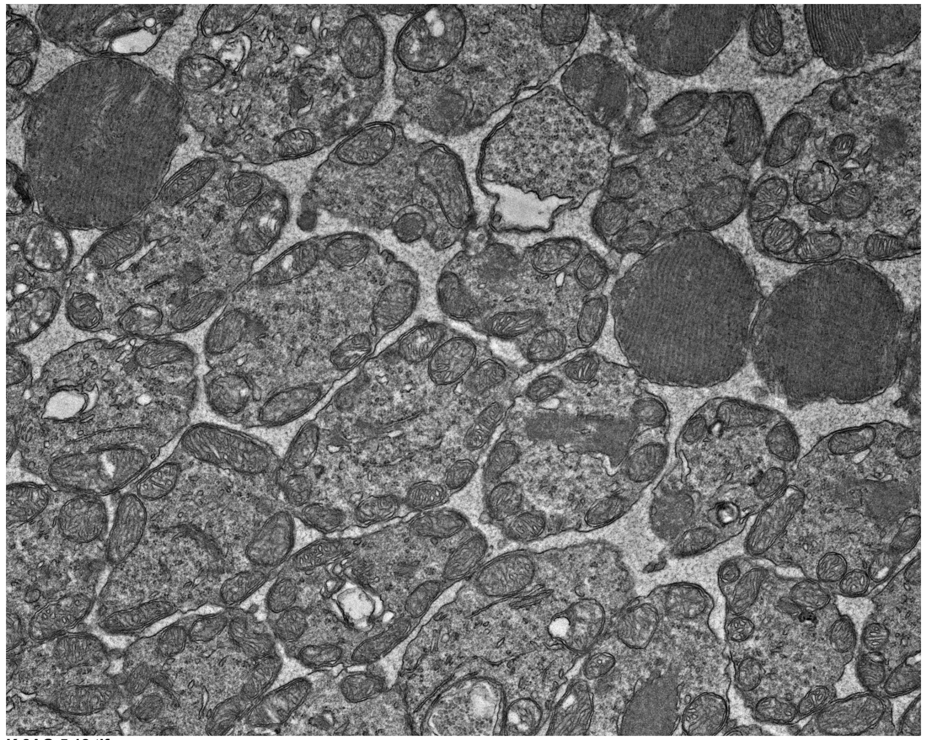

K 8AG-5 12.tif
83EM46 Chen
K 8AG-5
Print Mag: 18400x @ 7.0 in
11:03 2021-02-08
Microscopist: DBStolz
Camera: BIOSPR12, Exposure: 3200 (ms) x 1 std. frames, Gain: 1, Bin: 1
Gamma: 1.15, No Sharpening, Normal Contrast

1 µm
HV=80kV
Direct Mag: 4000 x
Center for Biologic Imaging

8-AG IS

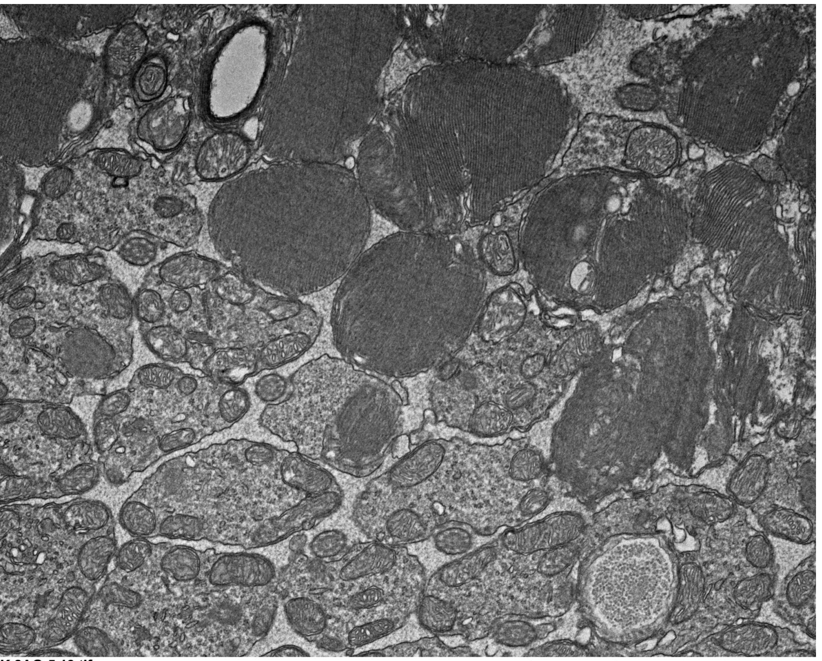

K 8AG-5 13.tif
83EM46 Chen
K 8AG-5
Print Mag: 18400x @ 7.0 in
11:07 2021-02-08
Microscopist: DBStolz
Camera: BIOSPR12, Exposure: 3200 (ms) x 1 std. frames, Gain: 1, Bin: 1
Gamma: 1.15, No Sharpening, Normal Contrast

1 µm
HV=80kV
Direct Mag: 4000 x
Center for Biologic Imaging

Central retina TEM

- Young N=6
- Aged untreated N=7
- Aged 8-AG N=7

Young central retina INL

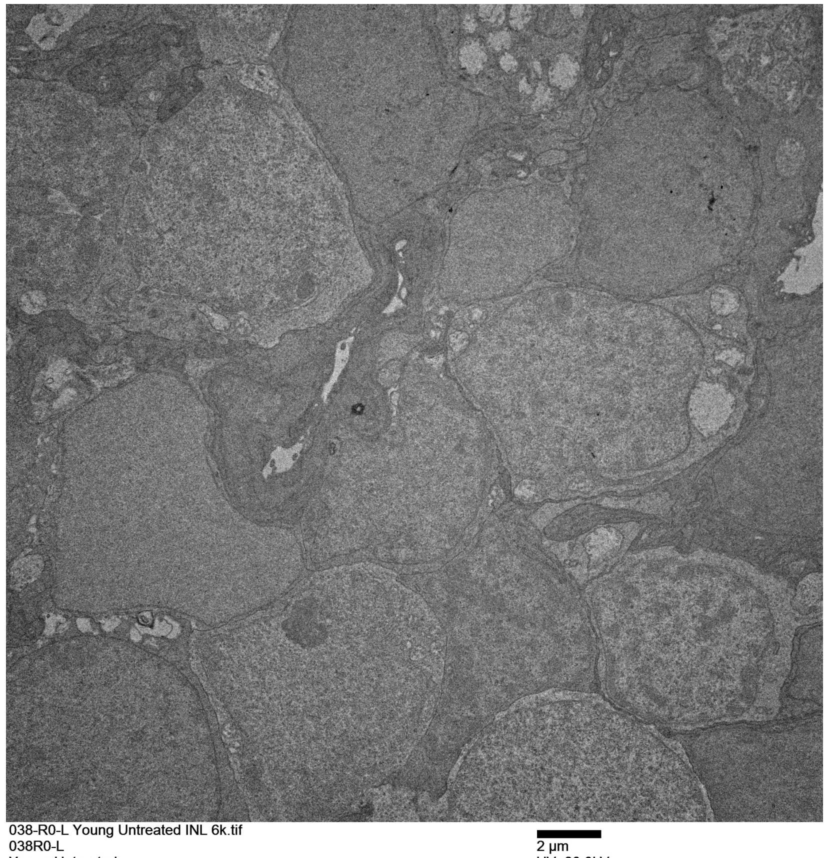

038-R0-L Young Untreated INL 6k.tif
038R0-L
Young Untreated
Print Mag: 7020x @ 7.0 in
11:51:36 2/16/2023

2 µm
HV=80.0kV
Direct Mag: 6000x
Center for Biologic Imaging

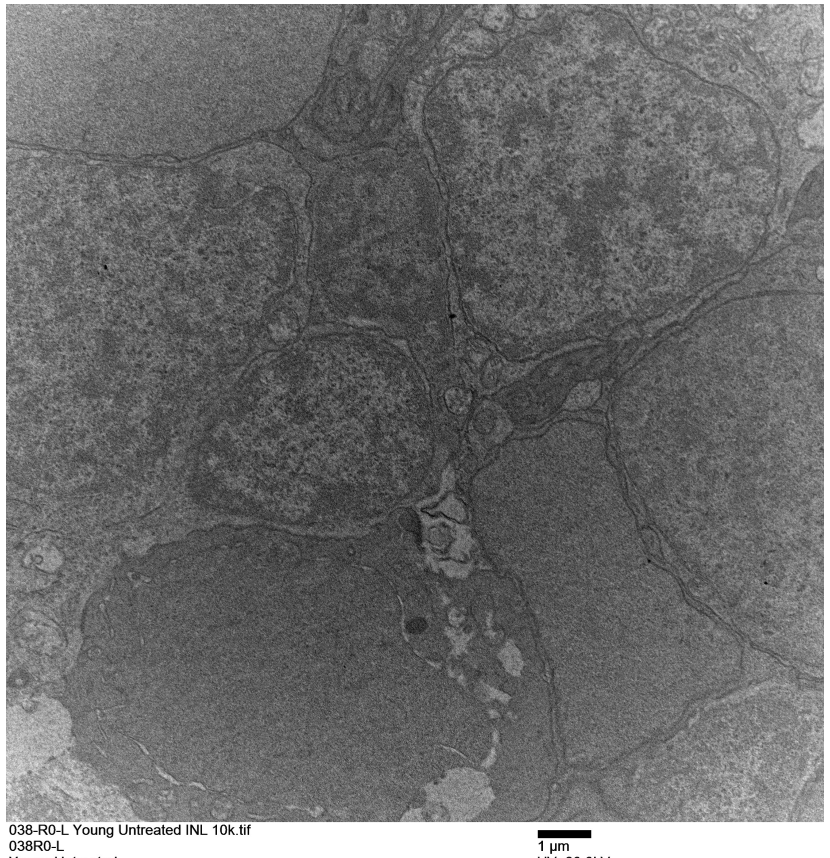

038-R0-L Young Untreated INL 10k.tif
038R0-L
Young Untreated
Print Mag: 11700x @ 7.0 in
11:52:34 2/16/2023

1 µm
HV=80.0kV
Direct Mag: 10000x
Center for Biologic Imaging

Young central IPL

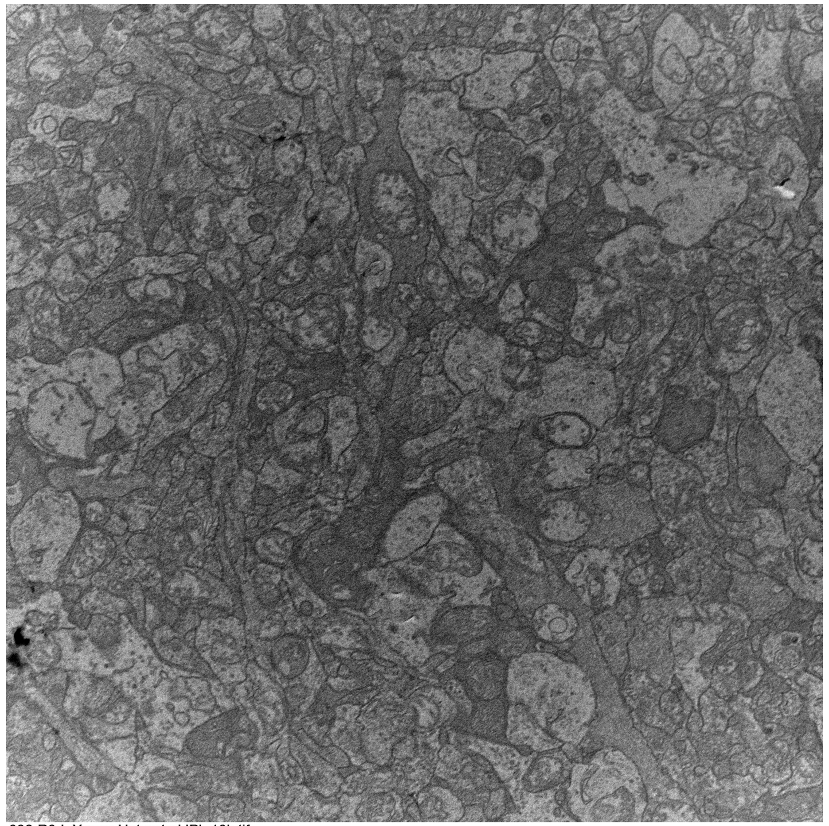

038-R0-L Young Untreated IPL 10k.tif
038R0-L
Young Untreated
Print Mag: 11700x @ 7.0 in
11:49:44 2/16/2023

1 µm
HV=80.0kV
Direct Mag: 10000x
Center for Biologic Imaging

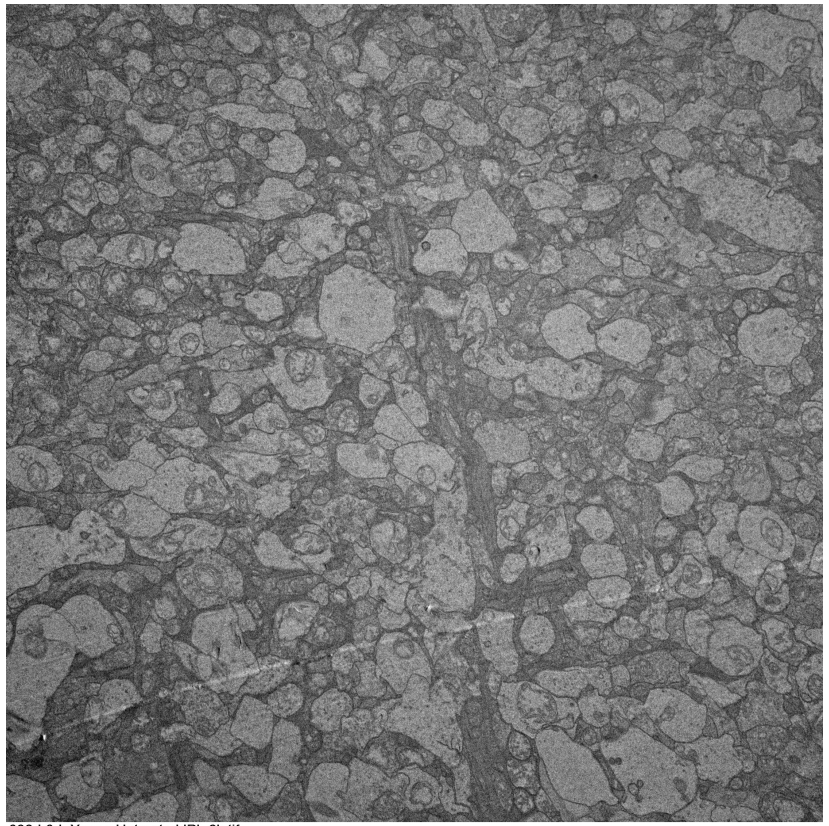

038-L0-L Young Untreated IPL 6k.tif
038L0-L
Young Untreated
Print Mag: 7020x @ 7.0 in
10:43:33 2/16/2023

2 µm
HV=80.0kV
Direct Mag: 6000x
Center for Biologic Imaging

Young IS

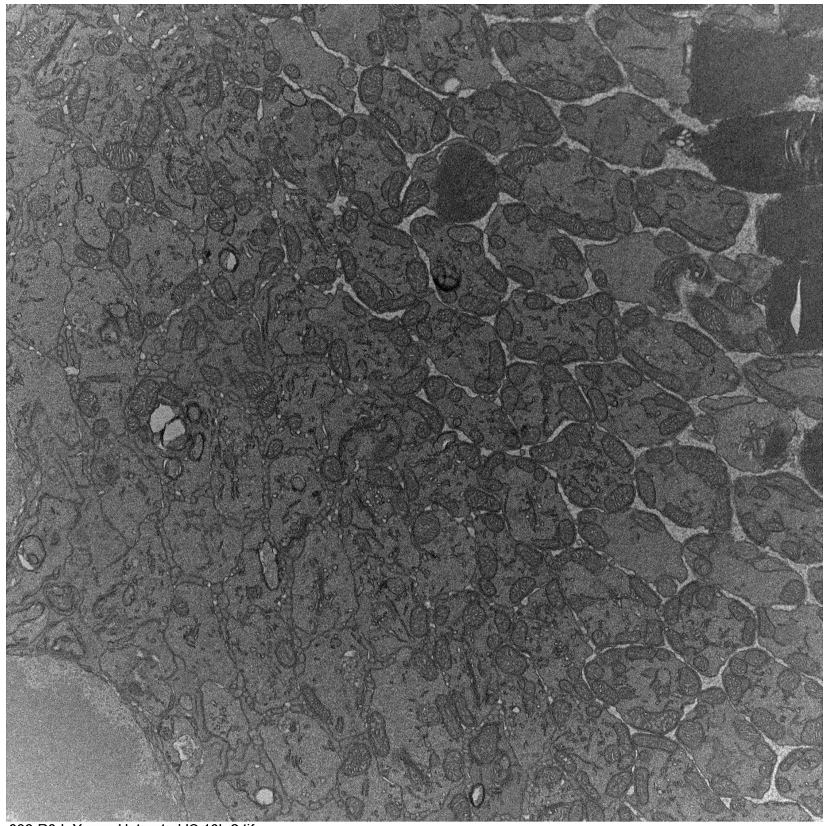

038-R0-L Young Untreated IS 10k-2.tif
038R0-L
Young Untreated
Print Mag: 11700x @ 7.0 in
11:27:16 2/16/2023

1 μm
HV=80.0kV
Direct Mag: 10000x
Center for Biologic Imaging

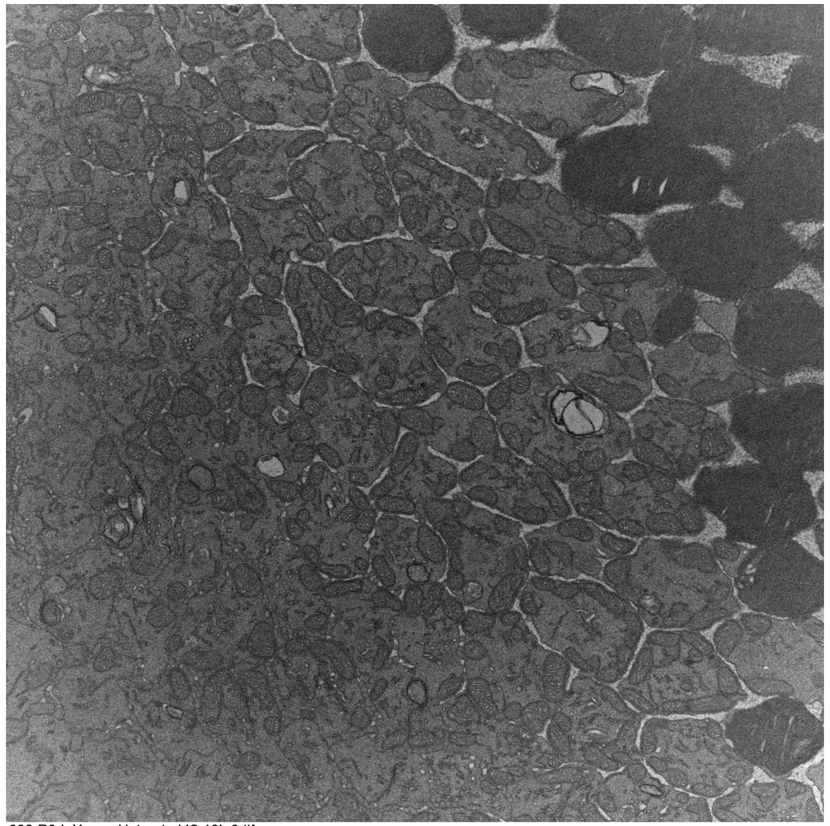

038-R0-L Young Untreated IS 10k-3.tif
038R0-L
Young Untreated
Print Mag: 11700x @ 7.0 in
12:01:09 2/16/2023

1 μm
HV=80.0kV
Direct Mag: 10000x
Center for Biologic Imaging

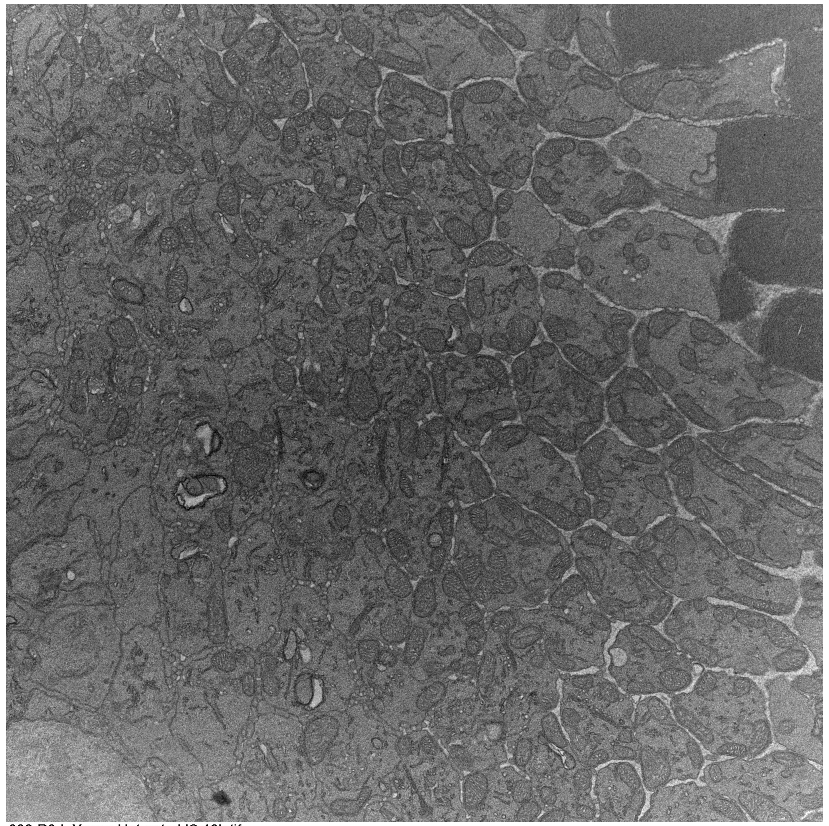

038-R0-L Young Untreated IS 10k.tif
038R0-L
Young Untreated
Print Mag: 11700x @ 7.0 in
11:26:23 2/16/2023

1 μm
HV=80.0kV
Direct Mag: 10000x
Center for Biologic Imaging

Young IS

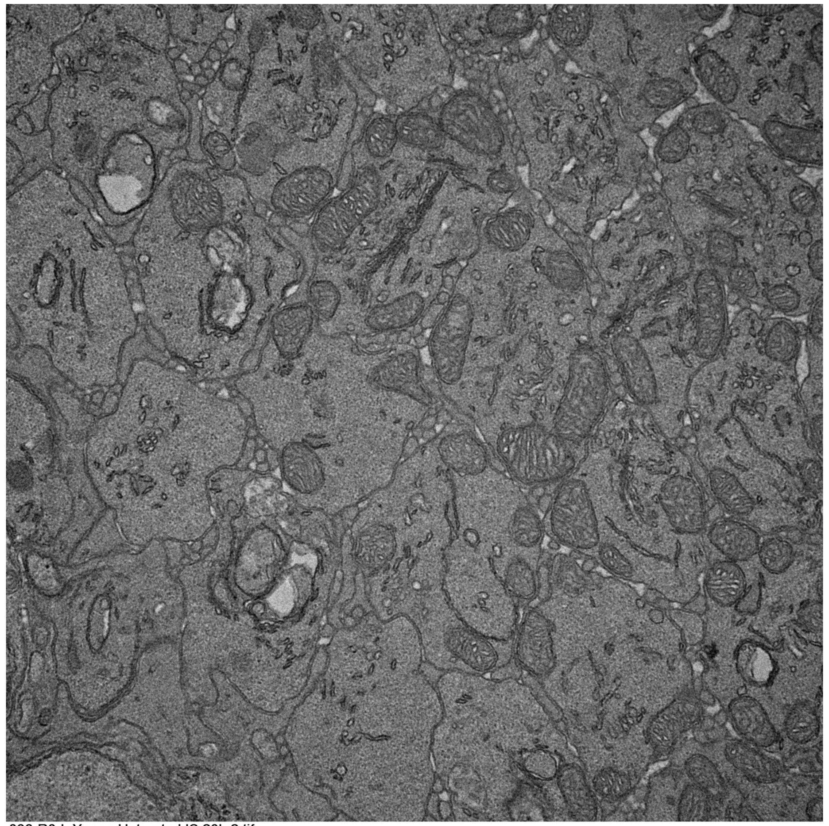

038-R0-L Young Untreated IS 20k-2.tif
038R0-L
Young Untreated
Print Mag: 23400x @ 7.0 in
11:37:27 2/16/2023

800 nm
HV=80.0kV
Direct Mag: 20000x
Center for Biologic Imaging

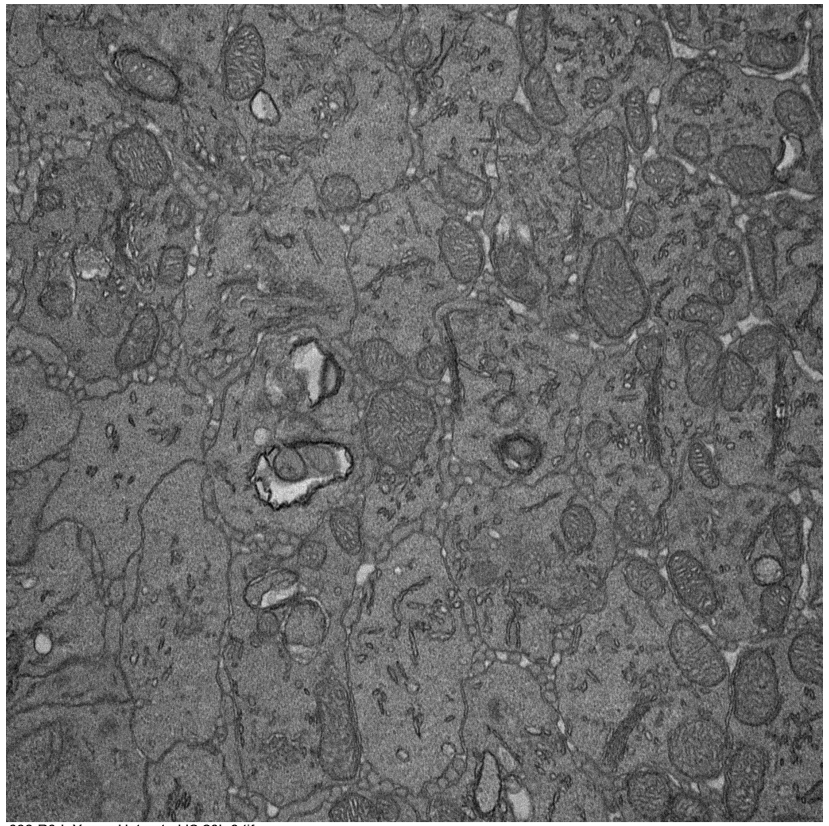

038-R0-L Young Untreated IS 20k-3.tif
038R0-L
Young Untreated
Print Mag: 23400x @ 7.0 in
11:38:10 2/16/2023

800 nm
HV=80.0kV
Direct Mag: 20000x
Center for Biologic Imaging

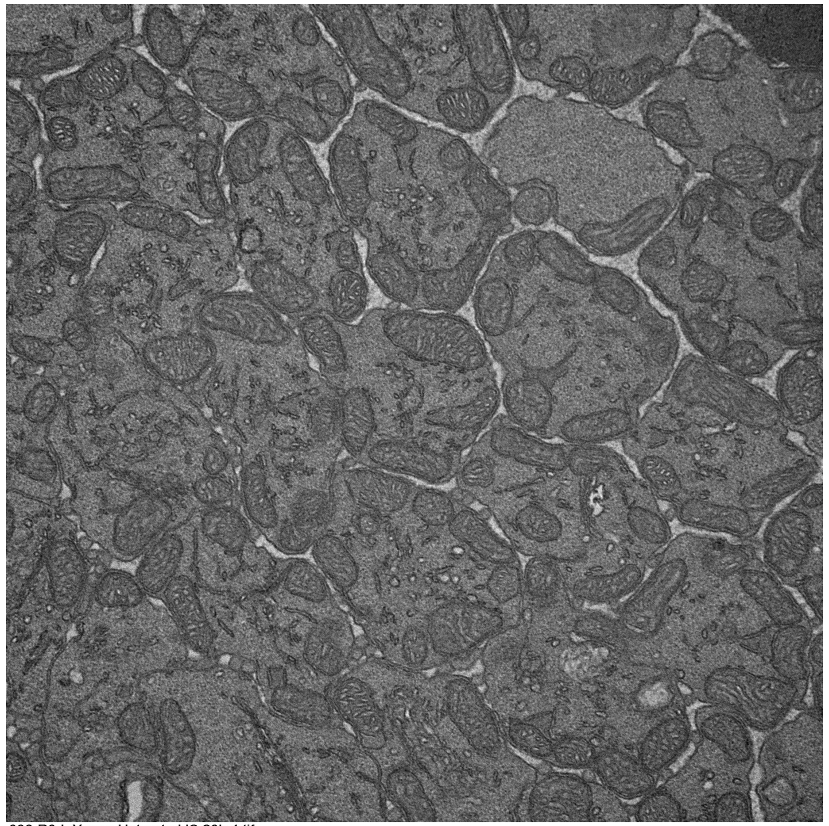

038-R0-L Young Untreated IS 20k-4.tif
038R0-L
Young Untreated
Print Mag: 23400x @ 7.0 in
12:03:29 2/16/2023

800 nm
HV=80.0kV
Direct Mag: 20000x
Center for Biologic Imaging

Young OS

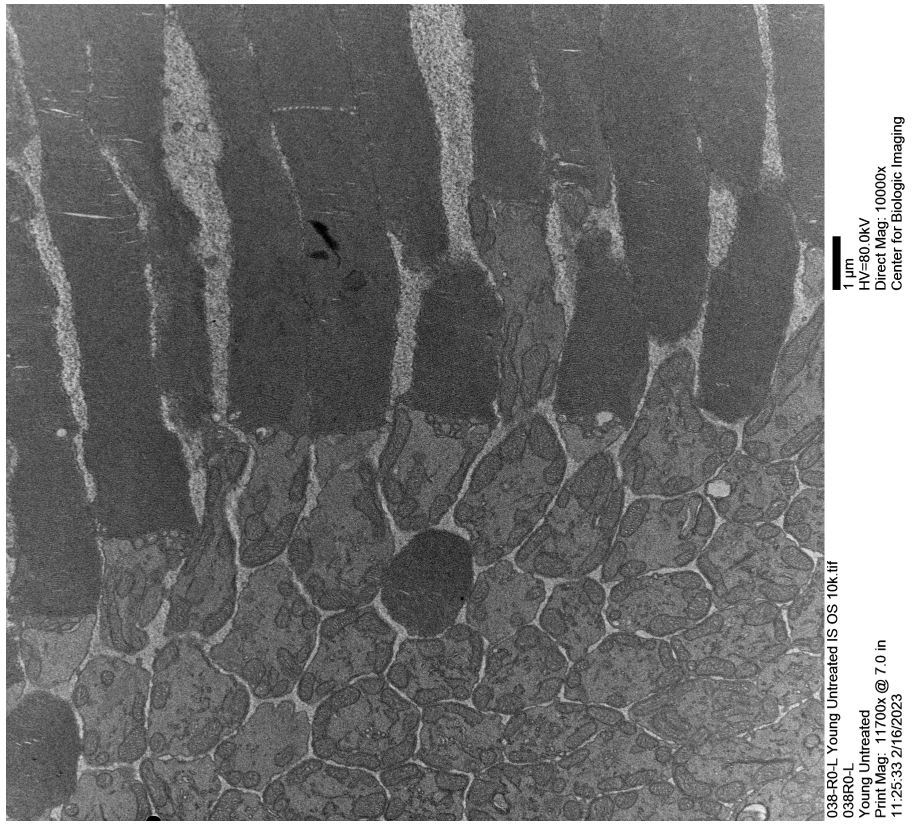

1 µm
HV=80.0kV
Direct Mag: 10000x
Center for Biologic Imaging
038-R0-L Young Untreated IS OS 10k.tif
038R0-L
Young Untreated
Print Mag: 11700x @ 7.0 in
11:25:33 2/16/2023

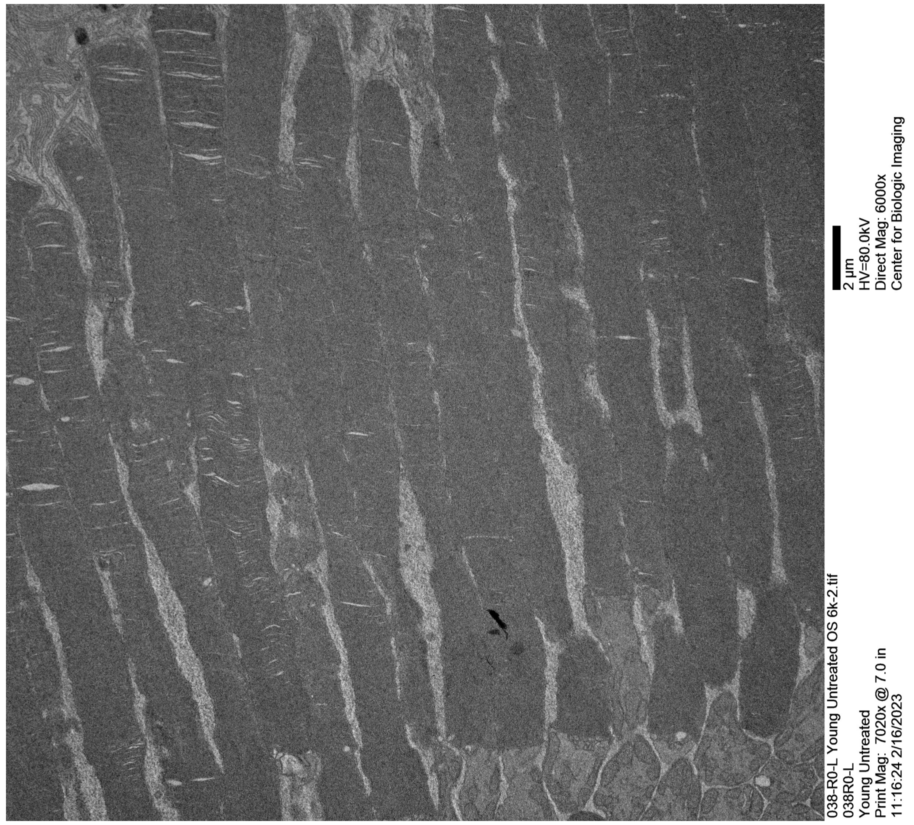

2 µm
HV=80.0kV
Direct Mag: 6000x
Center for Biologic Imaging
038-R0-L Young Untreated OS 6k-2.tif
038R0-L
Young Untreated
Print Mag: 7020x @ 7.0 in
11:16:24 2/16/2023

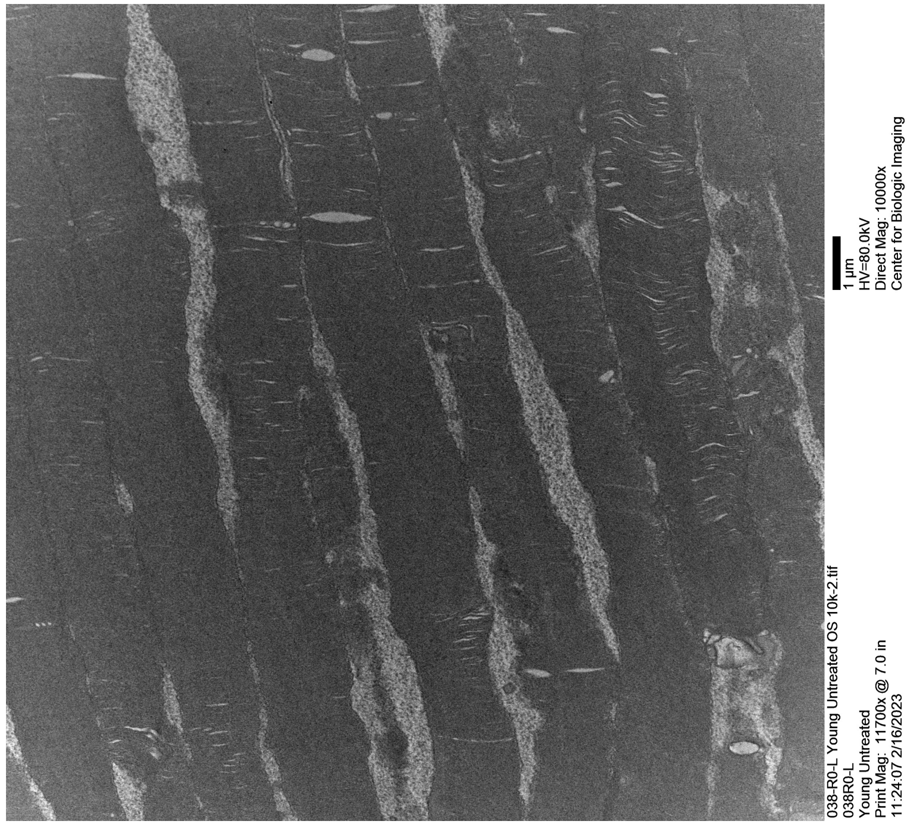

1 µm
HV=80.0kV
Direct Mag: 10000x
Center for Biologic Imaging
038-R0-L Young Untreated OS 10k-2.tif
038R0-L
Young Untreated
Print Mag: 11700x @ 7.0 in
11:24:07 2/16/2023

Young OS

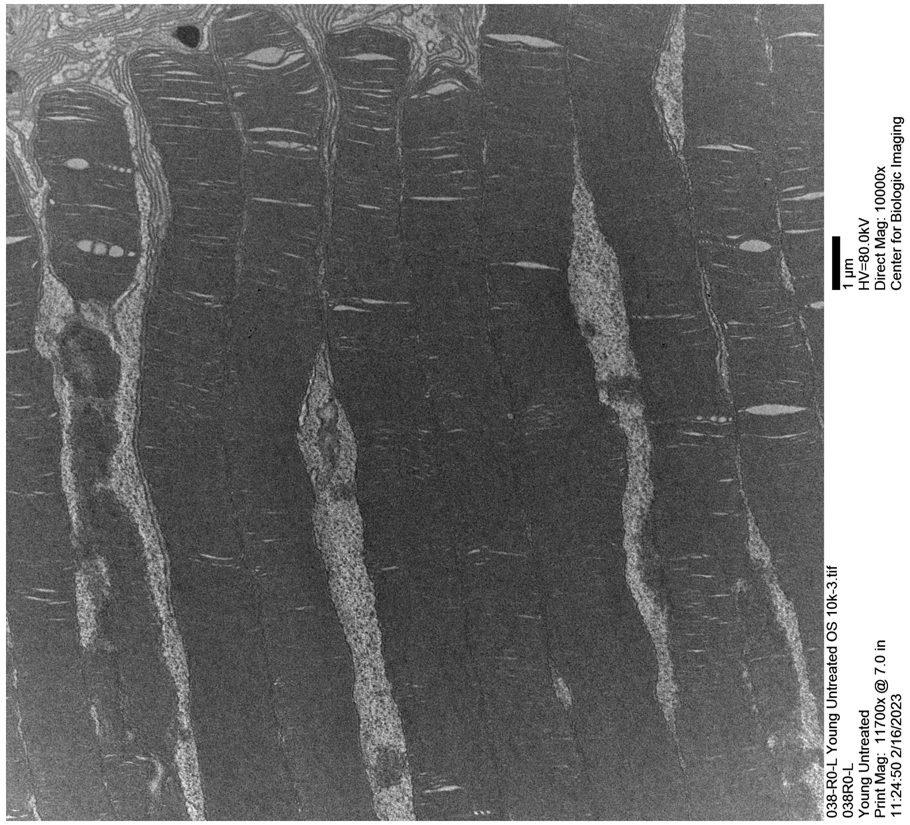
038-R0-L Young Untreated OS 10k-3.tif
038R0-L
Young Untreated
Print Mag: 11700x @ 7.0 in
11:24:50 2/16/2023
1 µm
HV=80.0kV
Direct Mag: 10000x
Center for Biologic Imaging

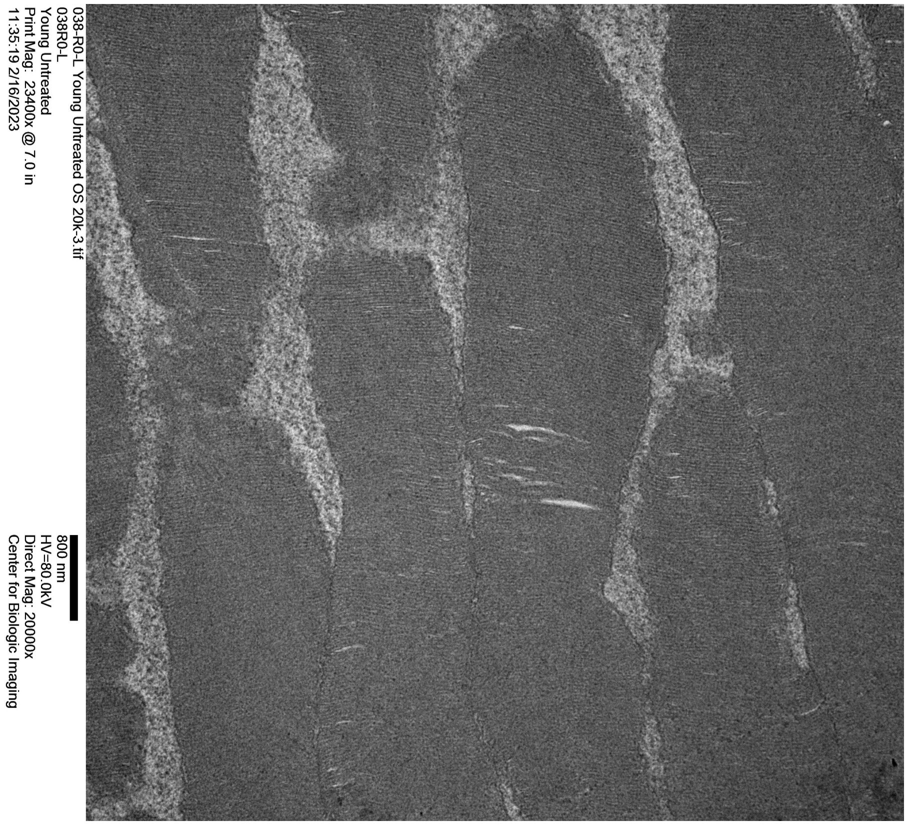
038-R0-L Young Untreated OS 20k-3.tif
038R0-L
Young Untreated
Print Mag: 23400x @ 7.0 in
11:35:19 2/16/2023
800 nm
HV=80.0kV
Direct Mag: 20000x
Center for Biologic Imaging

Young RPE

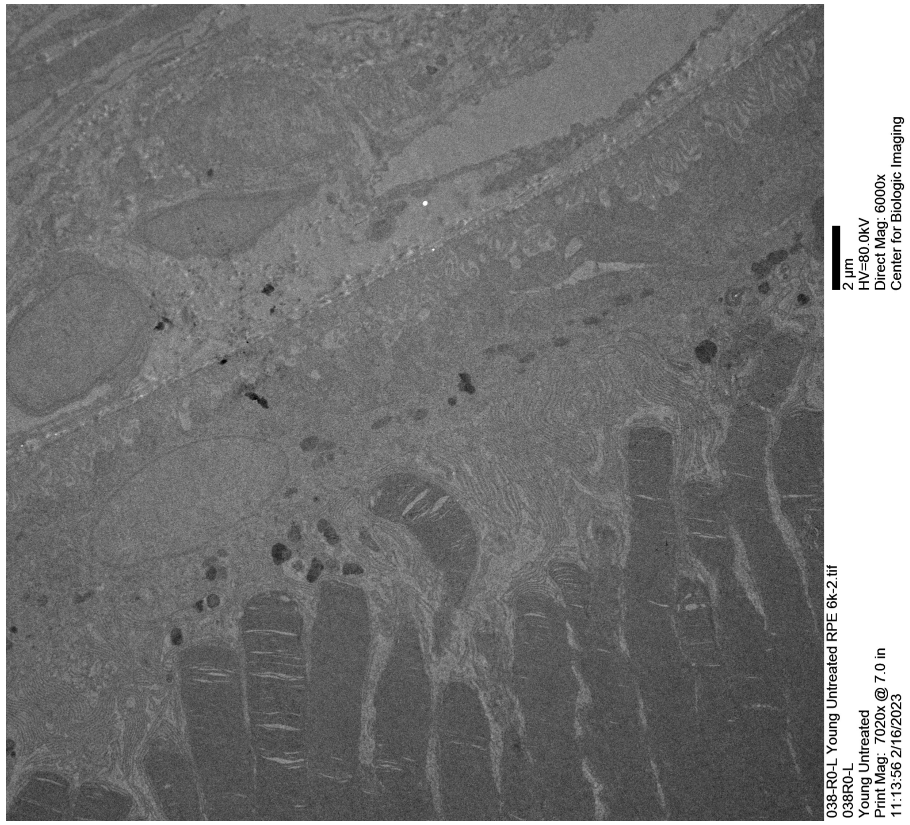

2 µm
HV=80.0kV
Direct Mag: 6000x
Center for Biologic Imaging
038-R0-L Young Untreated RPE 6k-2.tif
038R0-L
Young Untreated
Print Mag: 7020x @ 7.0 in
11:13:56 2/16/2023

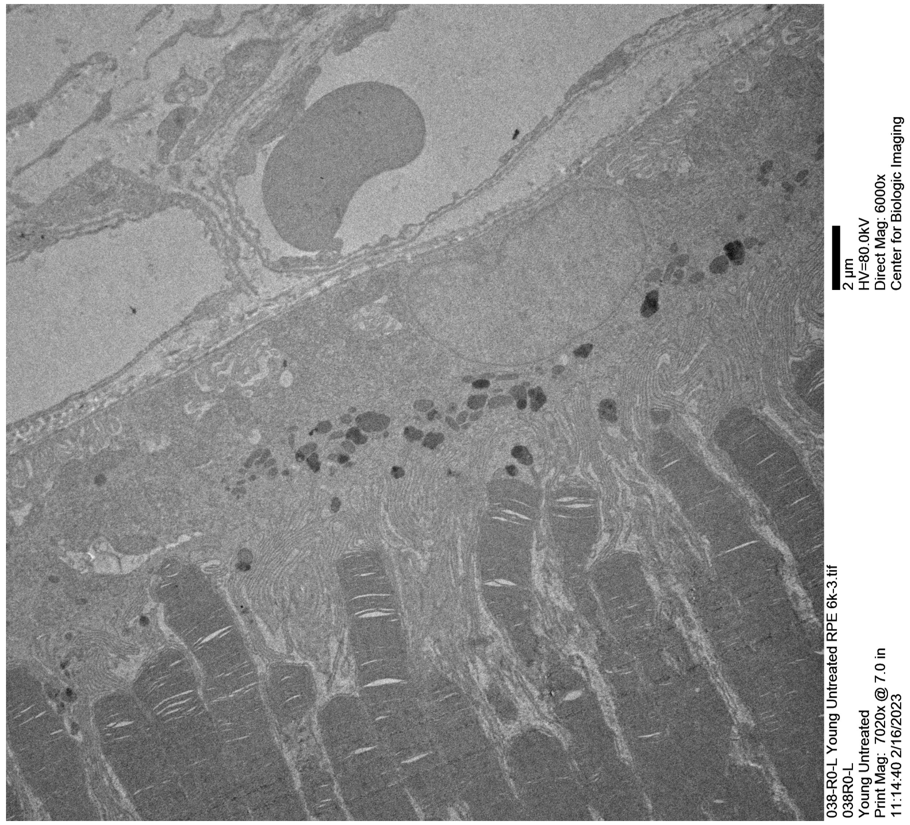

2 µm
HV=80.0kV
Direct Mag: 6000x
Center for Biologic Imaging
038-R0-L Young Untreated RPE 6k-3.tif
038R0-L
Young Untreated
Print Mag: 7020x @ 7.0 in
11:14:40 2/16/2023

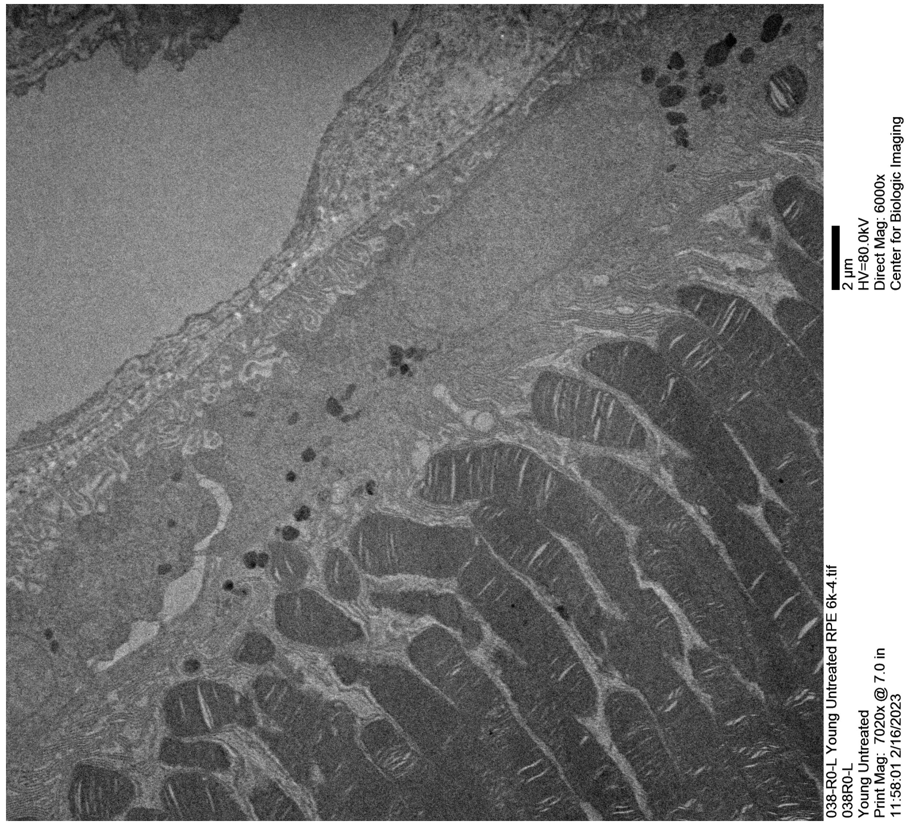

2 µm
HV=80.0kV
Direct Mag: 6000x
Center for Biologic Imaging
038-R0-L Young Untreated RPE 6k-4.tif
038R0-L
Young Untreated
Print Mag: 7020x @ 7.0 in
11:58:01 2/16/2023

Young RPE

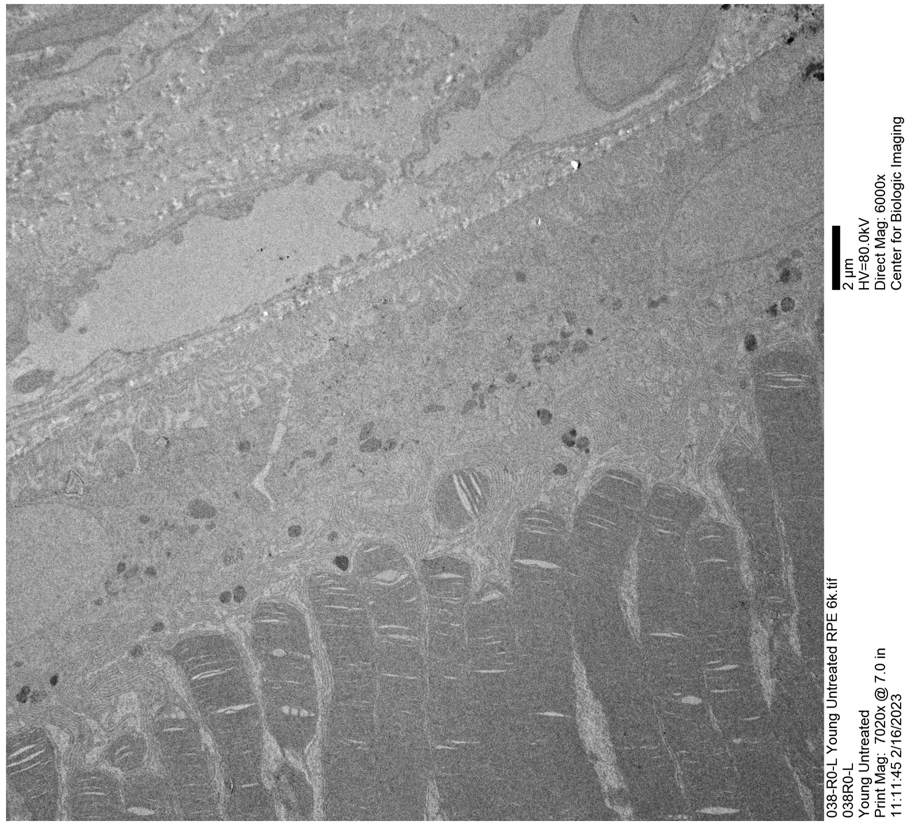
2 µm
HV=80.0kV
Direct Mag: 6000x
Center for Biologic Imaging
038-R0-L Young Untreated RPE 6k.tif
038R0-L
Young Untreated
Print Mag: 7020x @ 7.0 in
11:11:45 2/16/2023

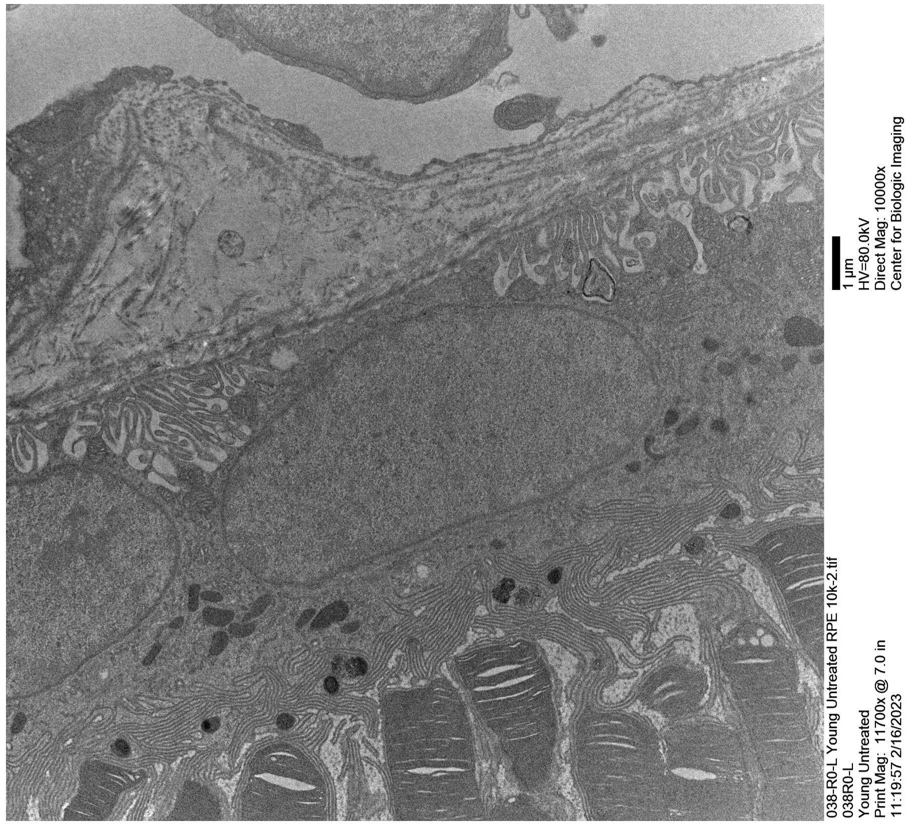
1 µm
HV=80.0kV
Direct Mag: 10000x
Center for Biologic Imaging
038-R0-L Young Untreated RPE 10k-2.tif
038R0-L
Young Untreated
Print Mag: 11700x @ 7.0 in
11:19:57 2/16/2023

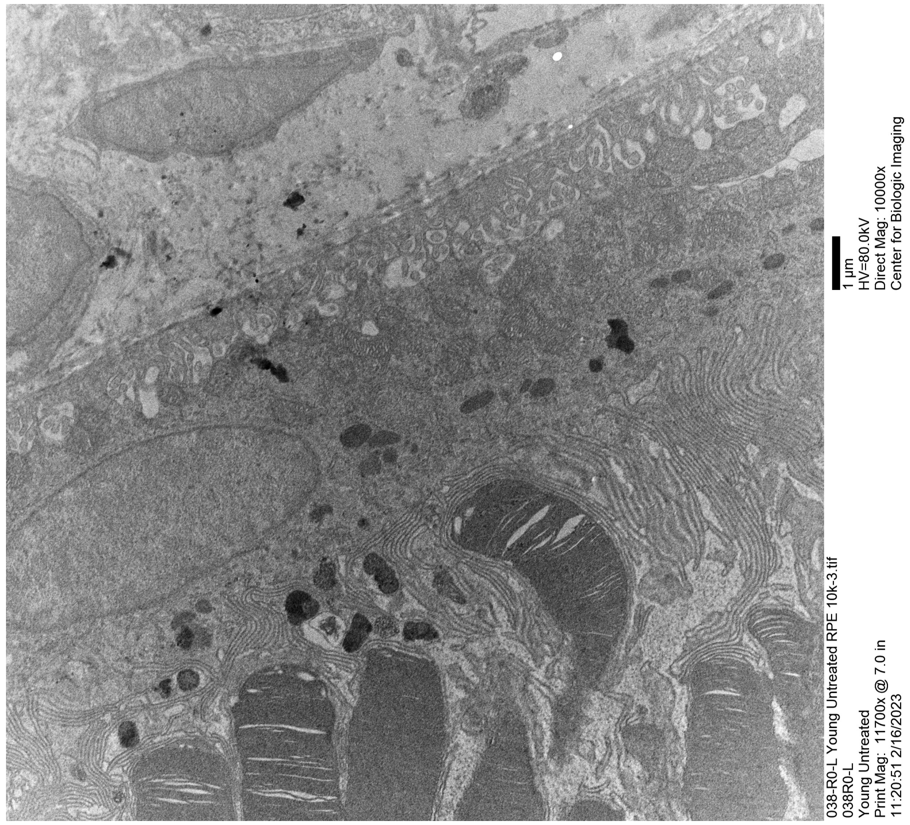
1 µm
HV=80.0kV
Direct Mag: 10000x
Center for Biologic Imaging
038-R0-L Young Untreated RPE 10k-3.tif
038R0-L
Young Untreated
Print Mag: 11700x @ 7.0 in
11:20:51 2/16/2023

Young RPE

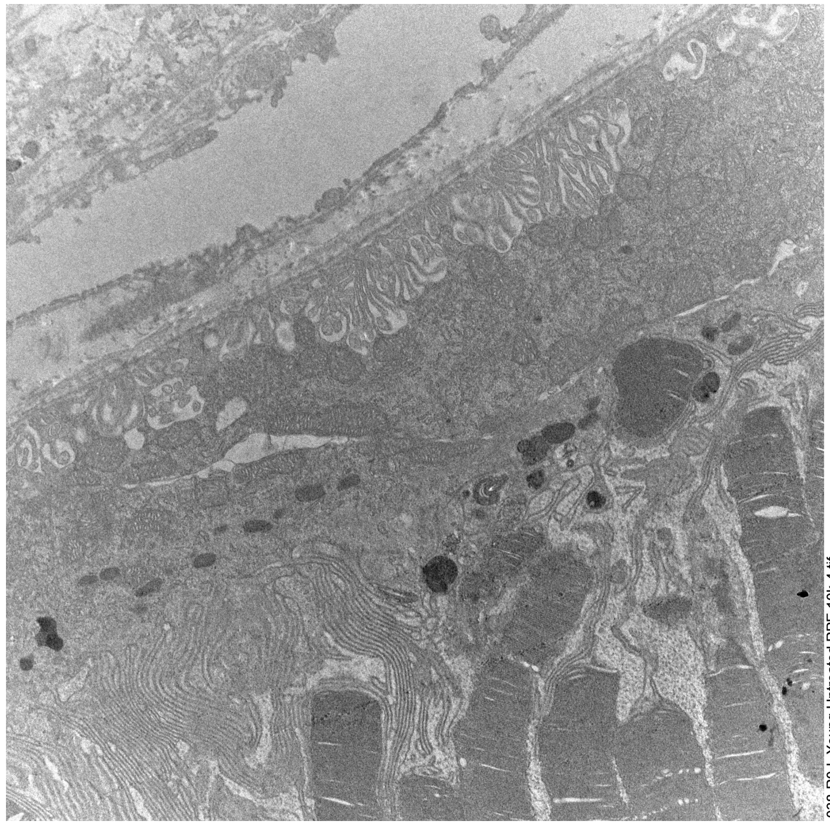

038-R0-L Young Untreated RPE 10k-4.tif
038R0-L
Young Untreated
Print Mag: 11700x @ 7.0 in
11:21:42 2/16/2023

1 µm
HV=80.0kV
Direct Mag: 10000x
Center for Biologic Imaging

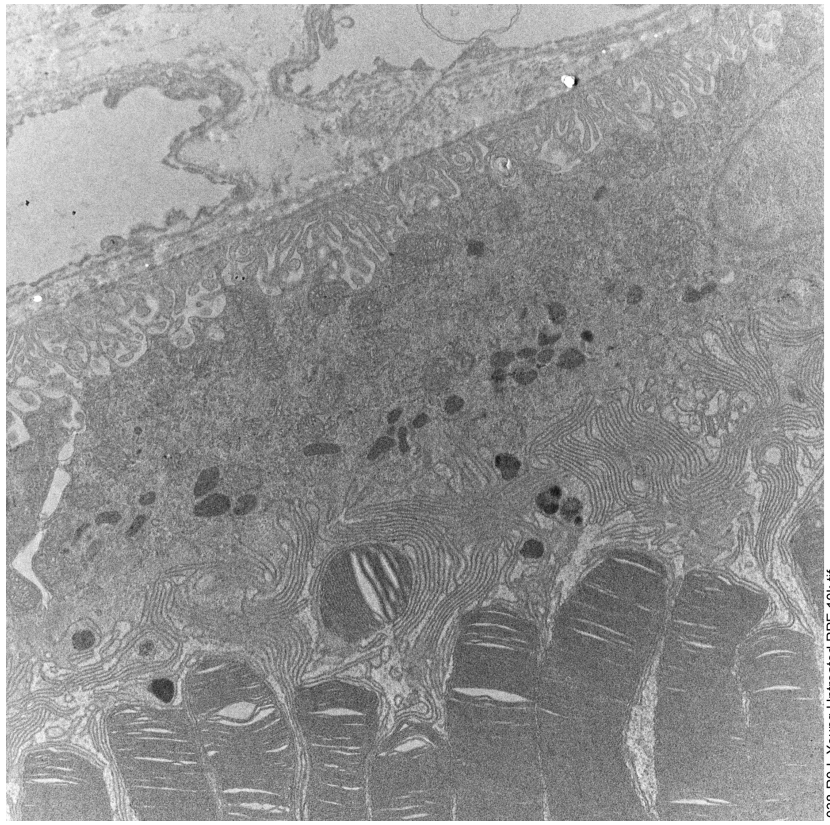

038-R0-L Young Untreated RPE 10k.tif
038R0-L
Young Untreated
Print Mag: 11700x @ 7.0 in
11:18:35 2/16/2023

1 µm
HV=80.0kV
Direct Mag: 10000x
Center for Biologic Imaging

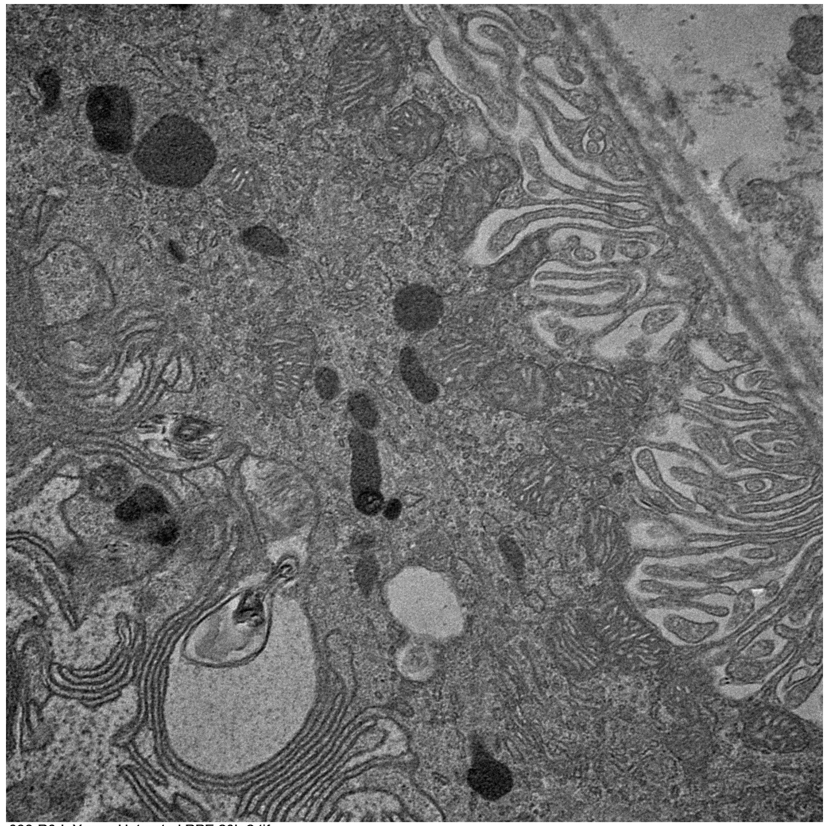

038-R0-L Young Untreated RPE 20k-2.tif
038R0-L
Young Untreated
Print Mag: 23400x @ 7.0 in
11:29:50 2/16/2023

800 nm
HV=80.0kV
Direct Mag: 20000x
Center for Biologic Imaging

Young RPE

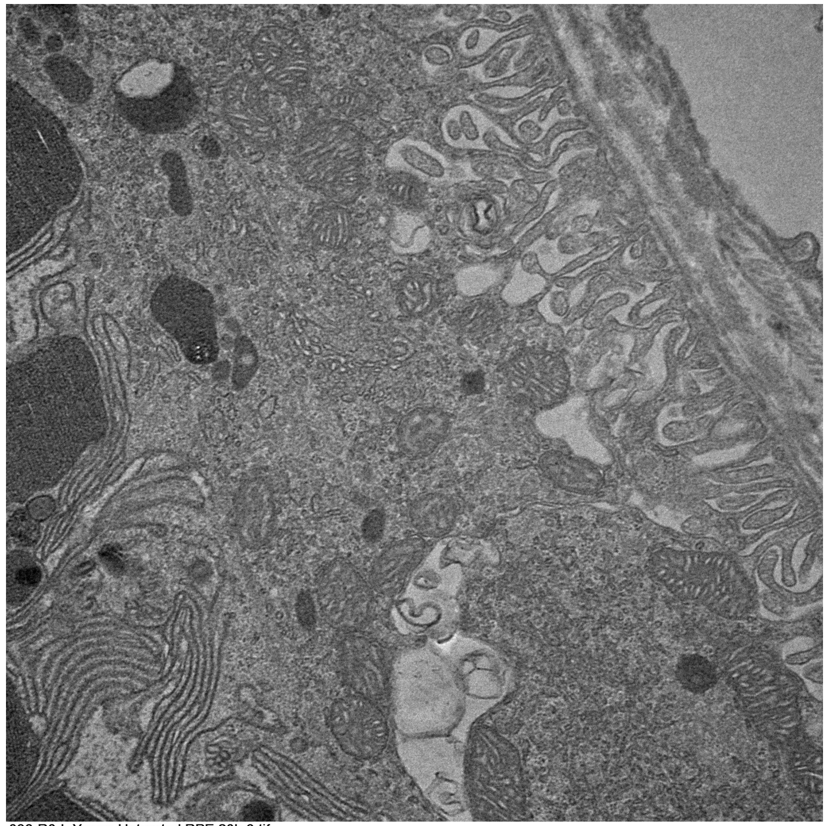

038-R0-L Young Untreated RPE 20k-3.tif
038R0-L
Young Untreated
Print Mag: 23400x @ 7.0 in
11:30:50 2/16/2023

800 nm
HV=80.0kV
Direct Mag: 20000x
Center for Biologic Imaging

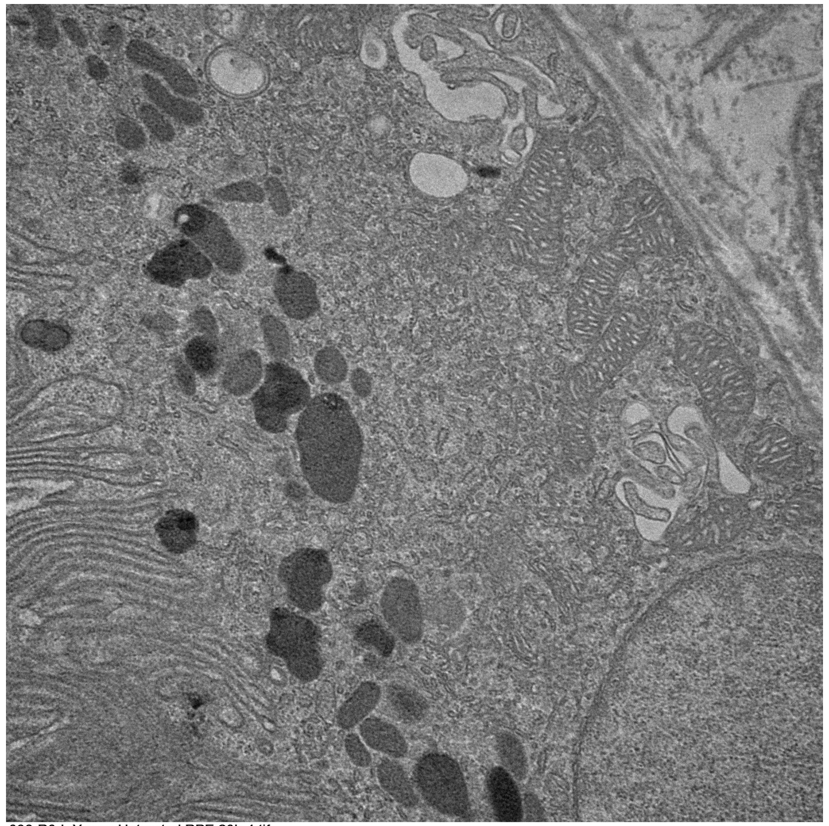

038-R0-L Young Untreated RPE 20k-4.tif
038R0-L
Young Untreated
Print Mag: 23400x @ 7.0 in
11:31:51 2/16/2023

800 nm
HV=80.0kV
Direct Mag: 20000x
Center for Biologic Imaging

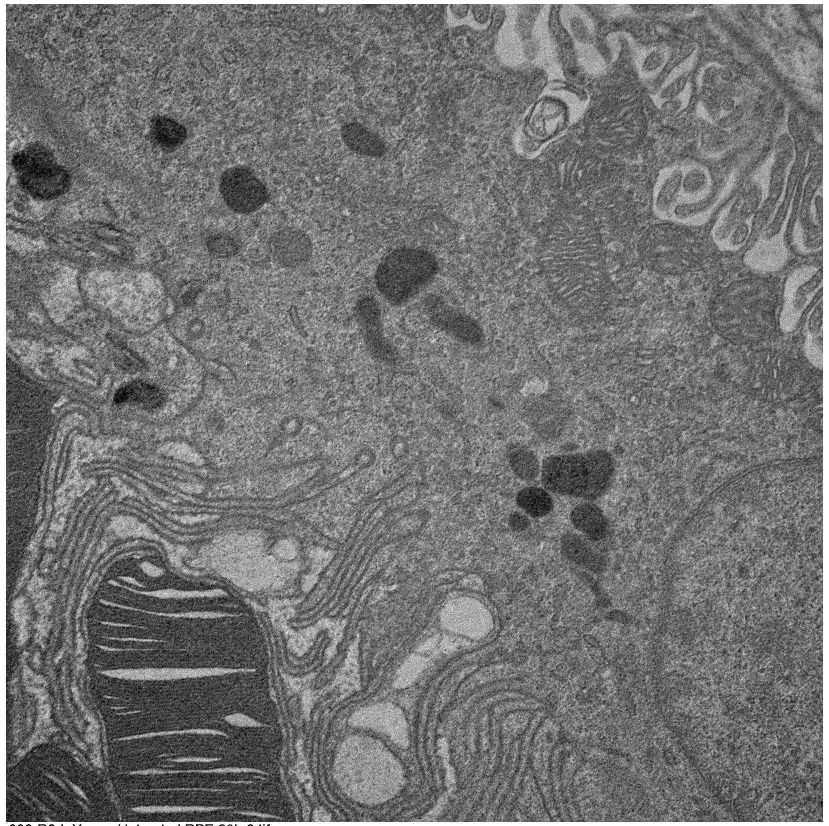

038-R0-L Young Untreated RPE 20k-6.tif
038R0-L
Young Untreated
Print Mag: 23400x @ 7.0 in
12:00:05 2/16/2023

800 nm
HV=80.0kV
Direct Mag: 20000x
Center for Biologic Imaging

Young RPE

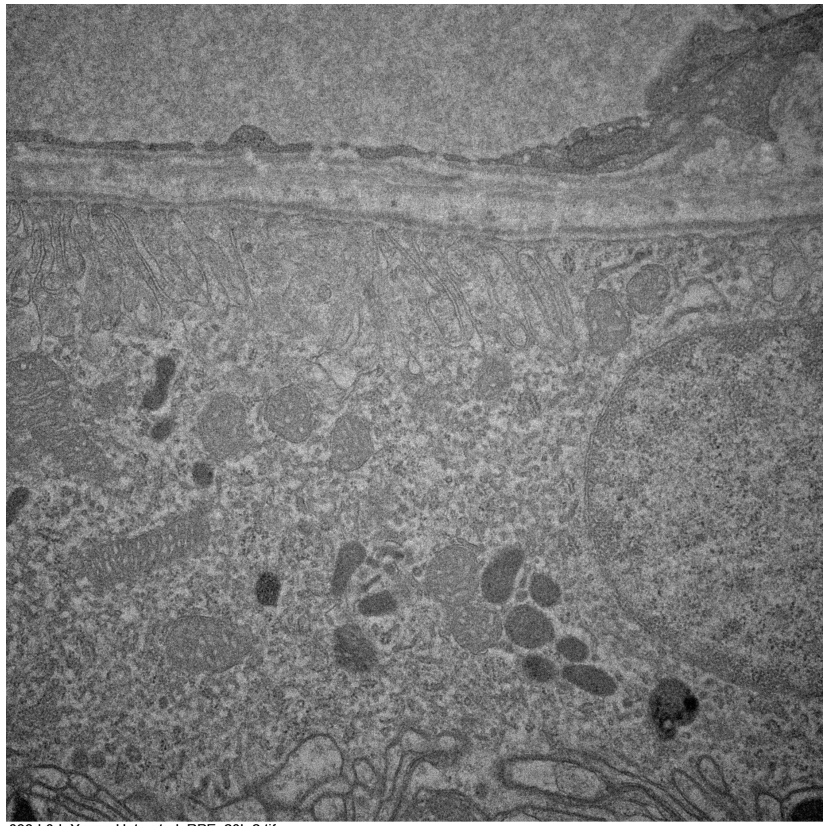

038-L0-L Young Untreated RPE 20k-2.tif
038L0-L
Young Untreated
Print Mag: 23400x @ 7.0 in
10:25:24 2/16/2023

800 nm
HV=80.0kV
Direct Mag: 20000x
Center for Biologic Imaging

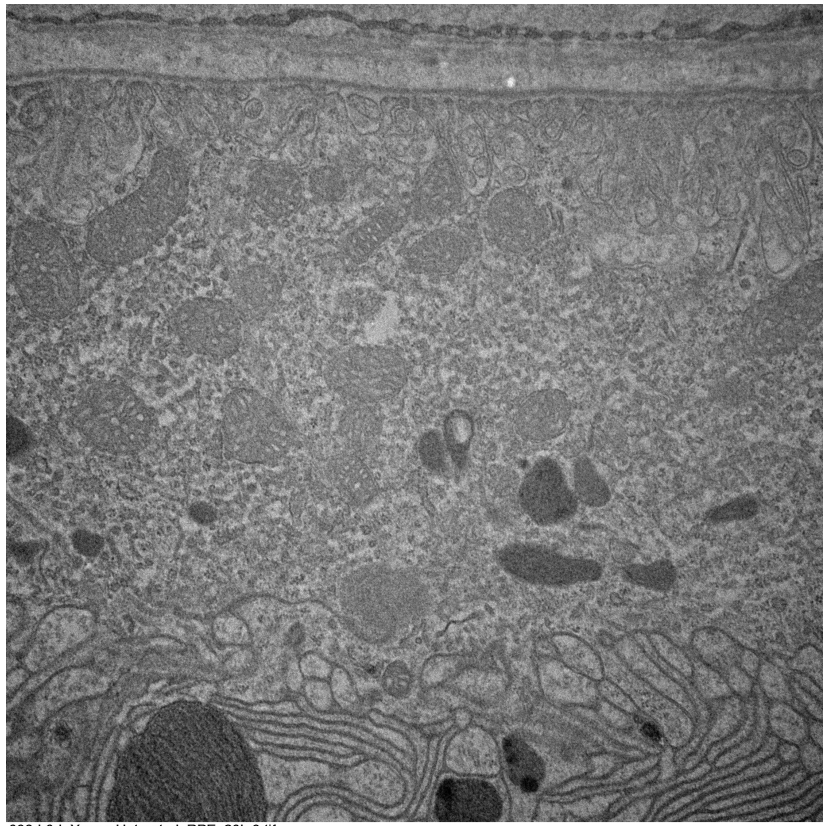

038-L0-L Young Untreated RPE 20k-3.tif
038L0-L
Young Untreated
Print Mag: 23400x @ 7.0 in
10:26:47 2/16/2023

800 nm
HV=80.0kV
Direct Mag: 20000x
Center for Biologic Imaging

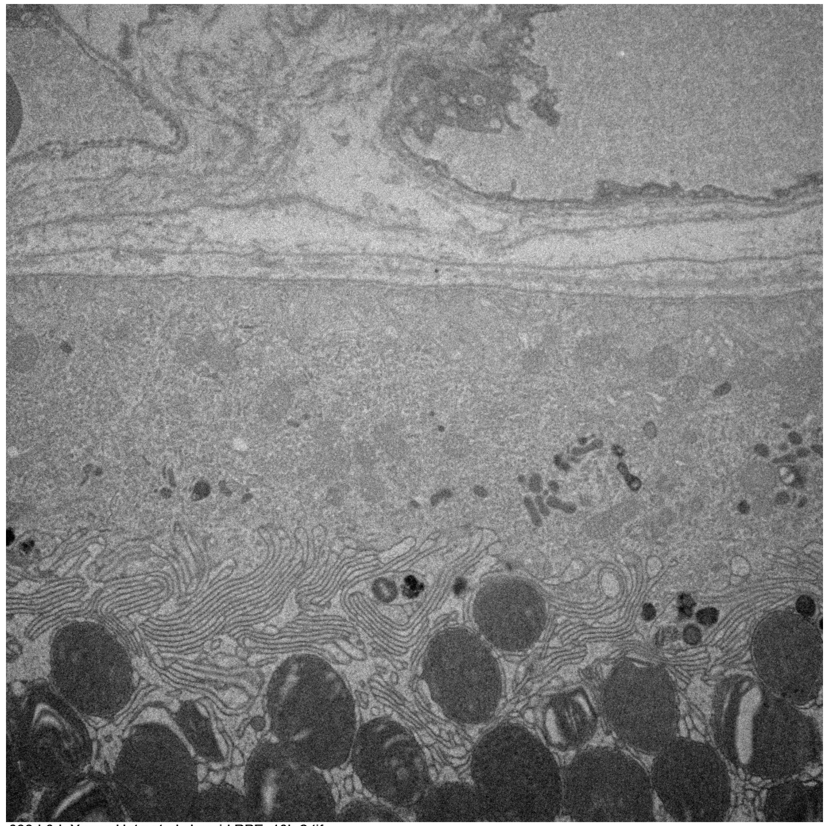

038-L0-L Young Untreated choroid RPE 10k-2.tif
038L0-L
Young Untreated
Print Mag: 11700x @ 7.0 in
10:11:10 2/16/2023

1 µm
HV=80.0kV
Direct Mag: 10000x
Center for Biologic Imaging

Young RPE

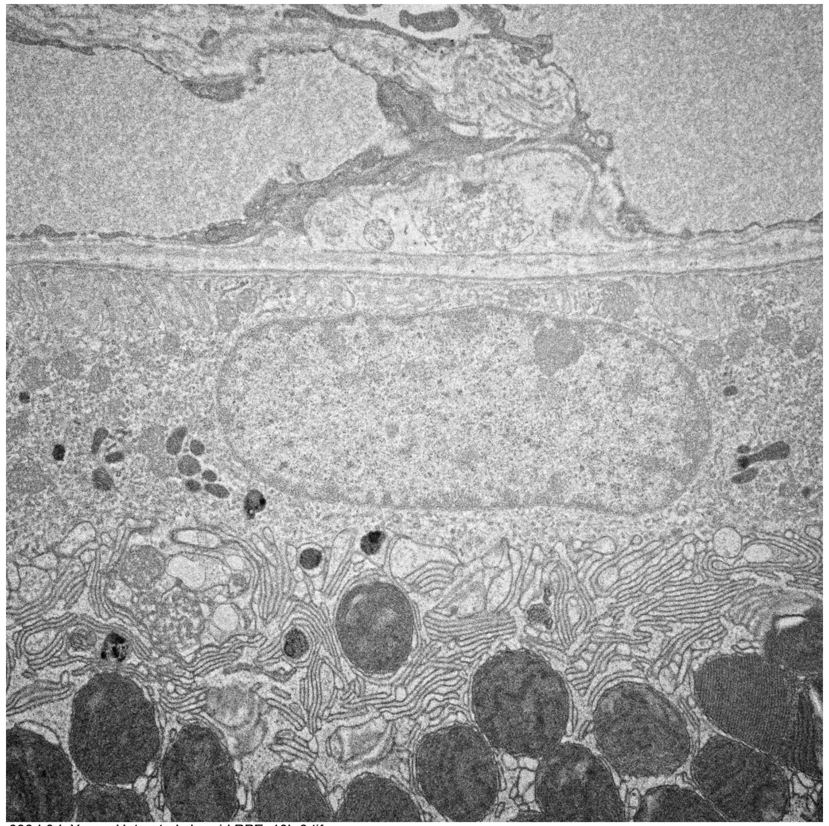

038-L0-L Young Untreated choroid RPE 10k-3.tif
038L0-L
Young Untreated
Print Mag: 11700x @ 7.0 in
10:13:12 2/16/2023

1 µm
HV=80.0kV
Direct Mag: 10000x
Center for Biologic Imaging

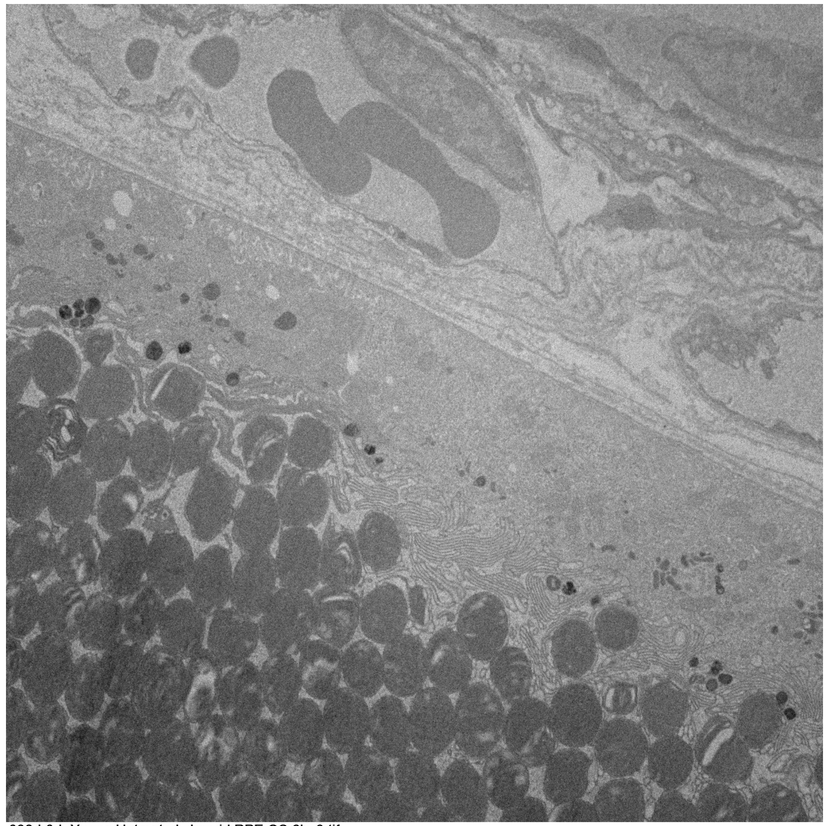

038-L0-L Young Untreated choroid RPE OS 6k -3.tif
038L0-L
Young Untreated
Print Mag: 7020x @ 7.0 in
09:58:48 2/16/2023

2 µm
HV=80.0kV
Direct Mag: 6000x
Center for Biologic Imaging

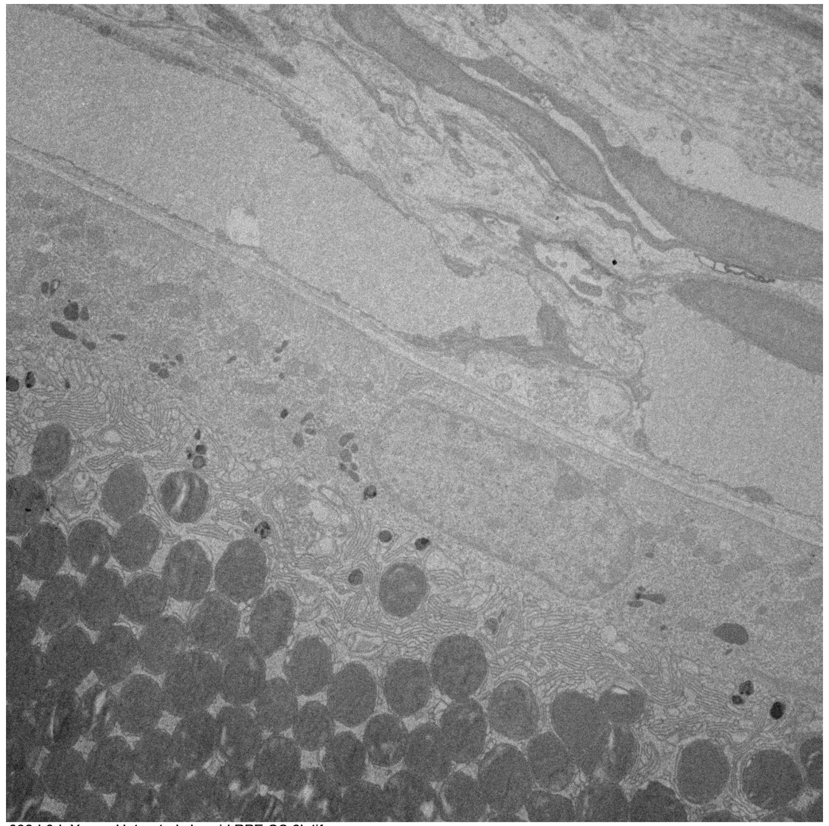

038-L0-L Young Untreated choroid RPE OS 6k.tif
038L0-L
Young Untreated
Print Mag: 7020x @ 7.0 in
09:55:51 2/16/2023

2 µm
HV=80.0kV
Direct Mag: 6000x
Center for Biologic Imaging

Young central retina low mag

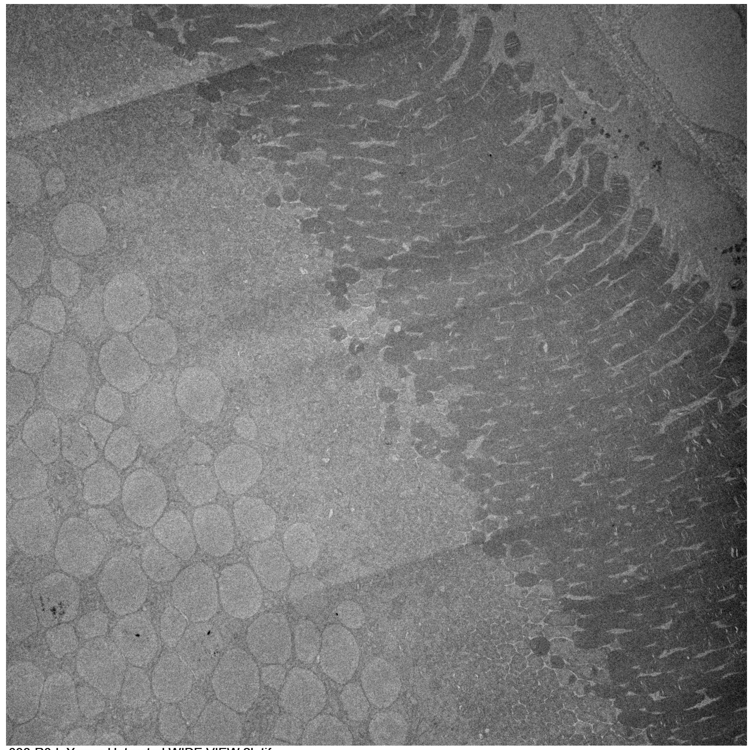

038-R0-L Young Untreated WIDE VIEW 2k.tif
038R0-L
Young Untreated
Print Mag: 2340x @ 7.0 in
11:56:17 2/16/2023

8 µm
HV=80.0kV
Direct Mag: 2000x
Center for Biologic Imaging

Young ONL

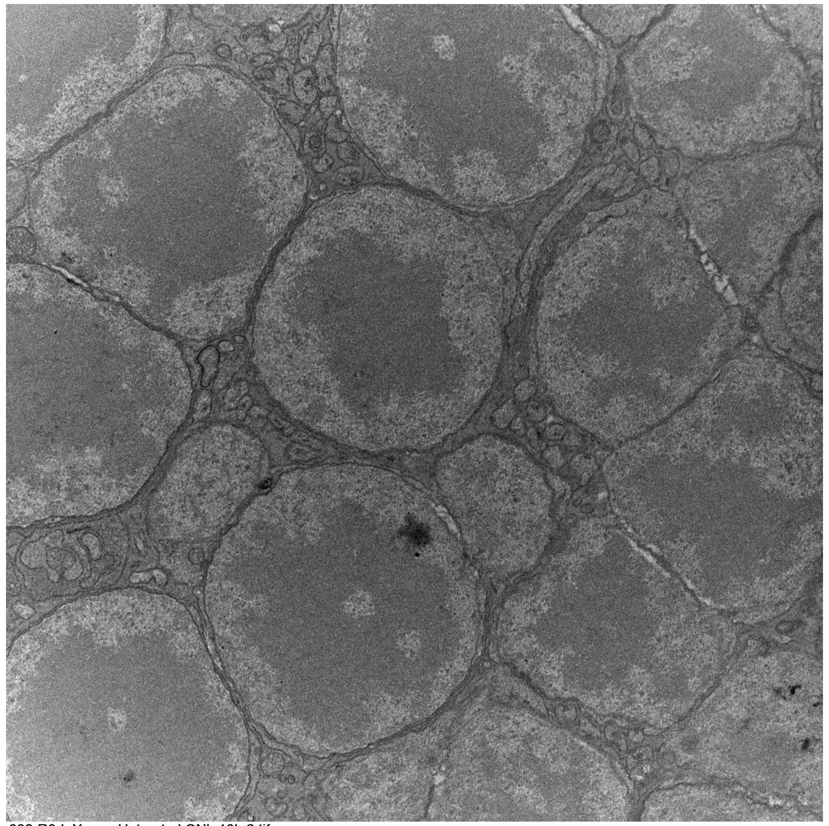

038-R0-L Young Untreated ONL 10k-2.tif
038R0-L
Young Untreated
Print Mag: 11700x @ 7.0 in
11:55:02 2/16/2023

1 µm
HV=80.0kV
Direct Mag: 10000x
Center for Biologic Imaging

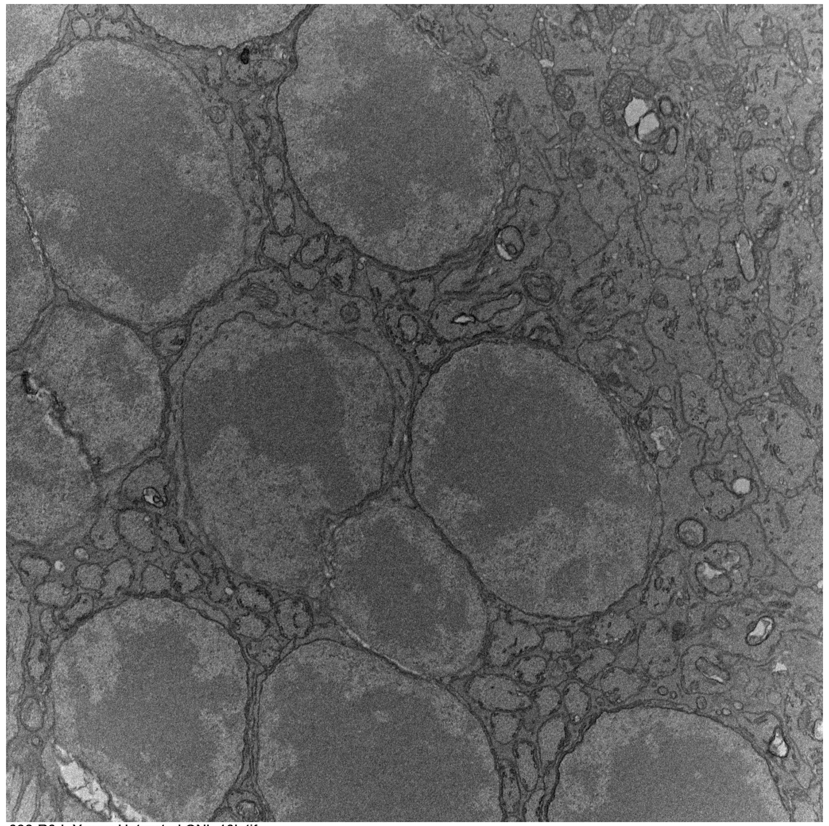

038-R0-L Young Untreated ONL 10k.tif
038R0-L
Young Untreated
Print Mag: 11700x @ 7.0 in
11:40:13 2/16/2023

1 µm
HV=80.0kV
Direct Mag: 10000x
Center for Biologic Imaging

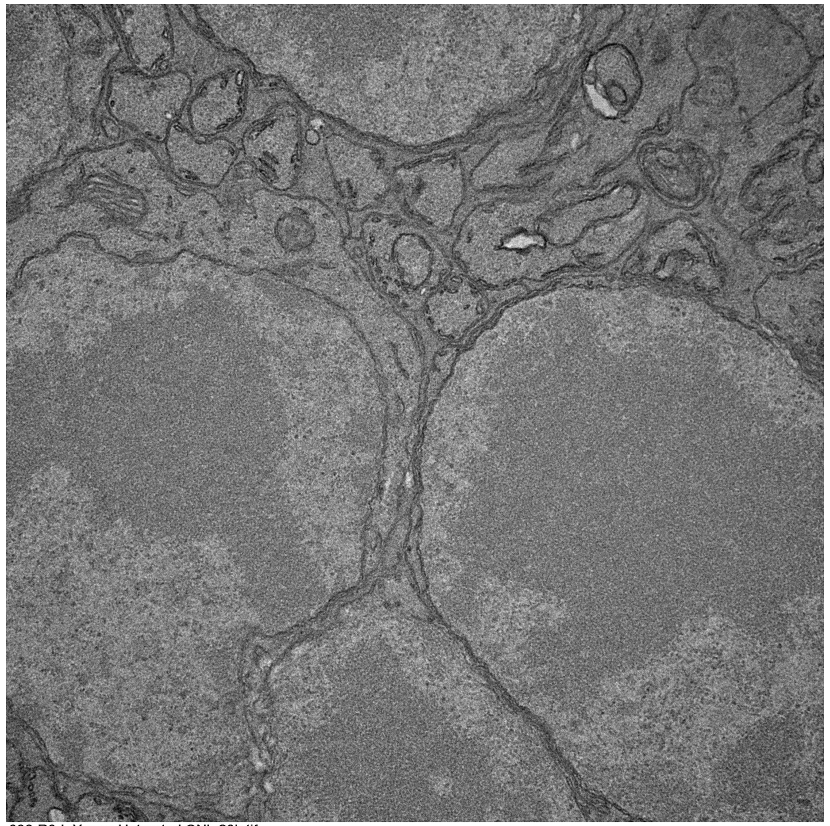

038-R0-L Young Untreated ONL 20k.tif
038R0-L
Young Untreated
Print Mag: 23400x @ 7.0 in
11:40:56 2/16/2023

800 nm
HV=80.0kV
Direct Mag: 20000x
Center for Biologic Imaging

Young ONL

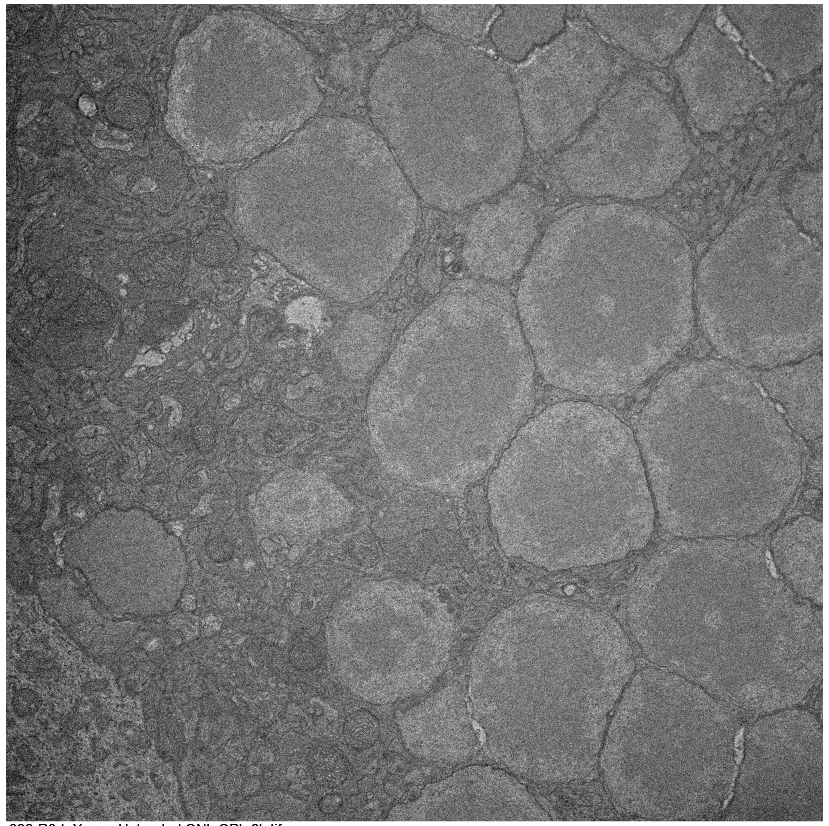

038-R0-L Young Untreated ONL OPL 6k.tif
038R0-L
Young Untreated
Print Mag: 7020x @ 7.0 in
11:42:52 2/16/2023

2 µm
HV=80.0kV
Direct Mag: 6000x
Center for Biologic Imaging

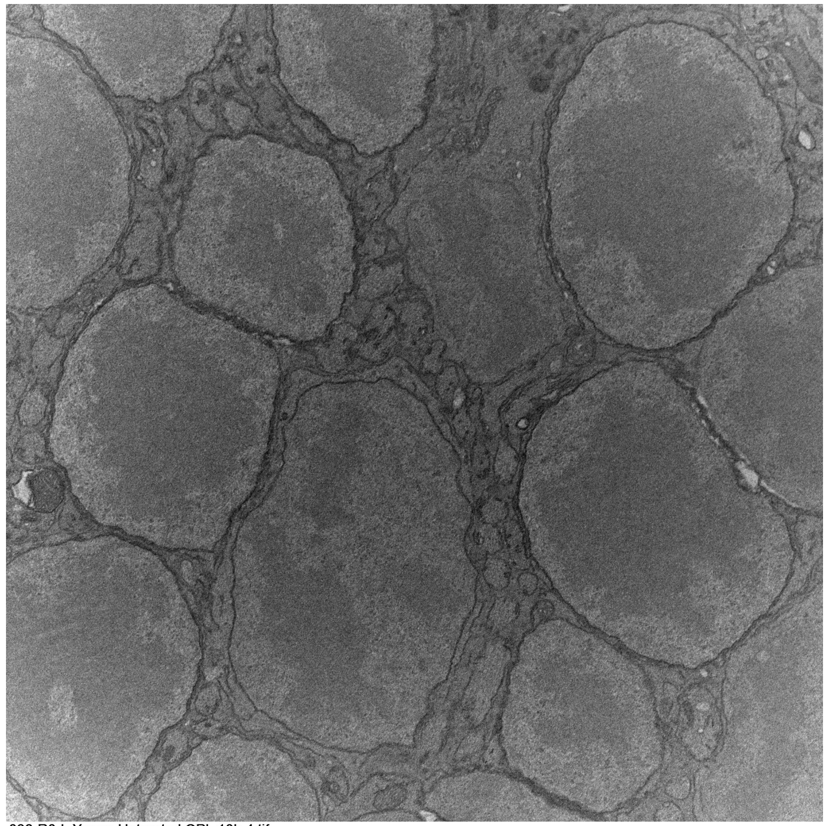

038-R0-L Young Untreated OPL 10k-4.tif
038R0-L
Young Untreated
Print Mag: 11700x @ 7.0 in
12:02:03 2/16/2023

1 µm
HV=80.0kV
Direct Mag: 10000x
Center for Biologic Imaging

Young OPL

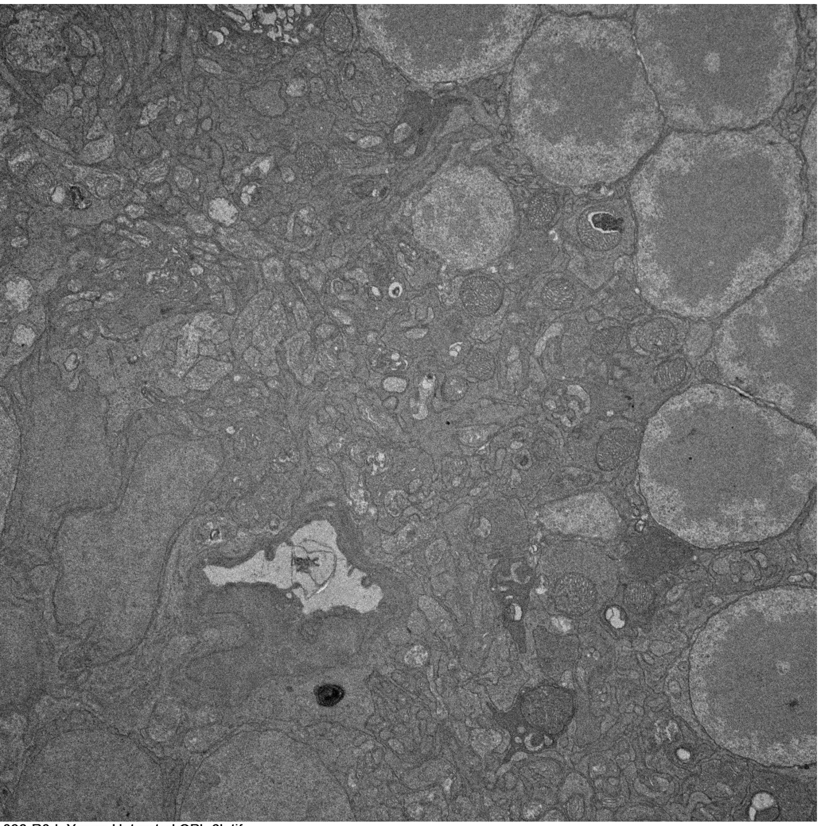

038-R0-L Young Untreated OPL 6k.tif
038R0-L
Young Untreated
Print Mag: 7020x @ 7.0 in
11:53:19 2/16/2023

2 µm
HV=80.0kV
Direct Mag: 6000x
Center for Biologic Imaging

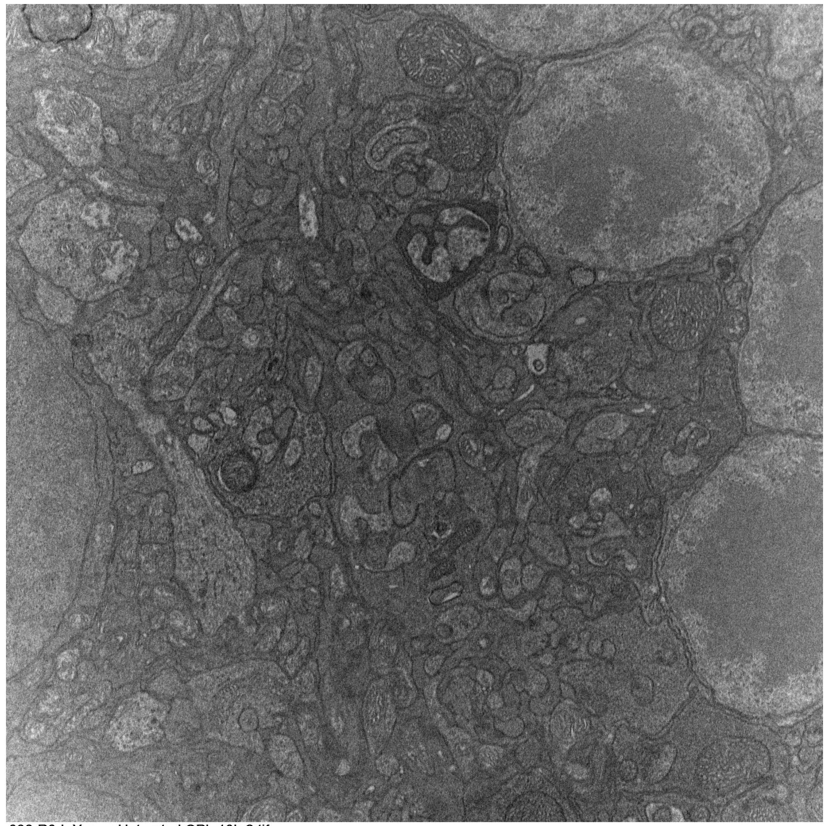

038-R0-L Young Untreated OPL 10k-2.tif
038R0-L
Young Untreated
Print Mag: 11700x @ 7.0 in
11:45:36 2/16/2023

1 µm
HV=80.0kV
Direct Mag: 10000x
Center for Biologic Imaging

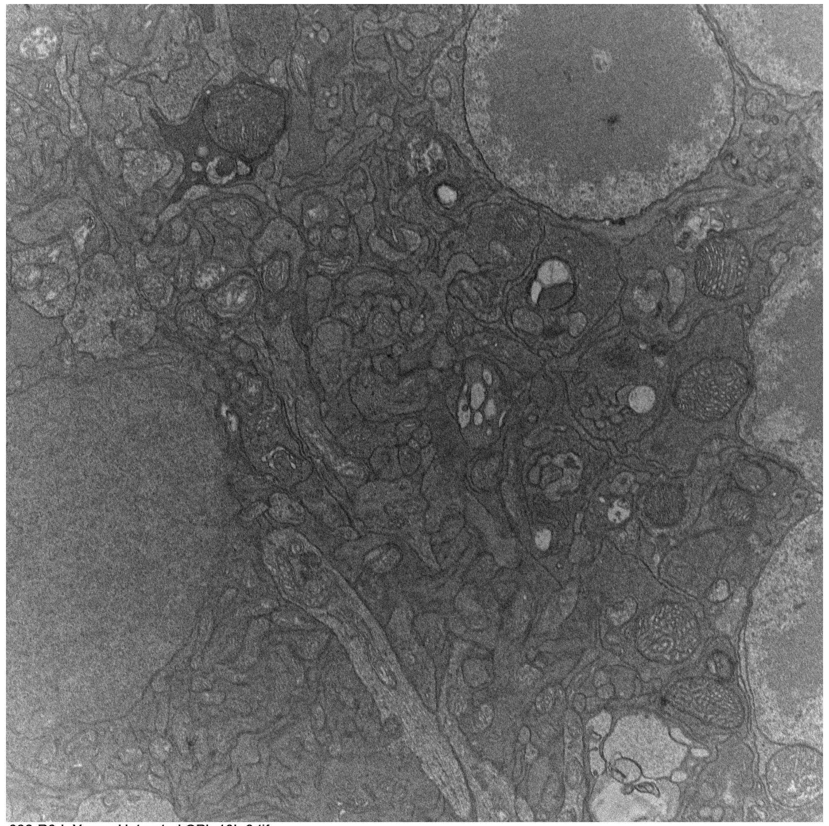

038-R0-L Young Untreated OPL 10k-3.tif
038R0-L
Young Untreated
Print Mag: 11700x @ 7.0 in
11:54:12 2/16/2023

1 µm
HV=80.0kV
Direct Mag: 10000x
Center for Biologic Imaging

Young OPL

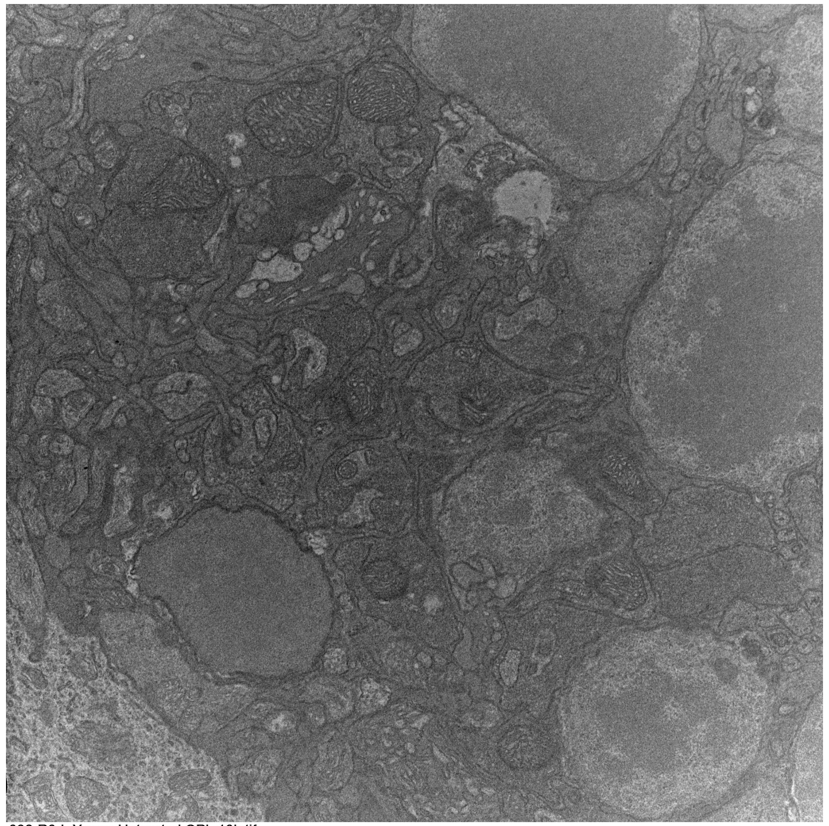

038-R0-L Young Untreated OPL 10k.tif
038R0-L
Young Untreated
Print Mag: 11700x @ 7.0 in
11:44:01 2/16/2023

1 µm
HV=80.0kV
Direct Mag: 10000x
Center for Biologic Imaging

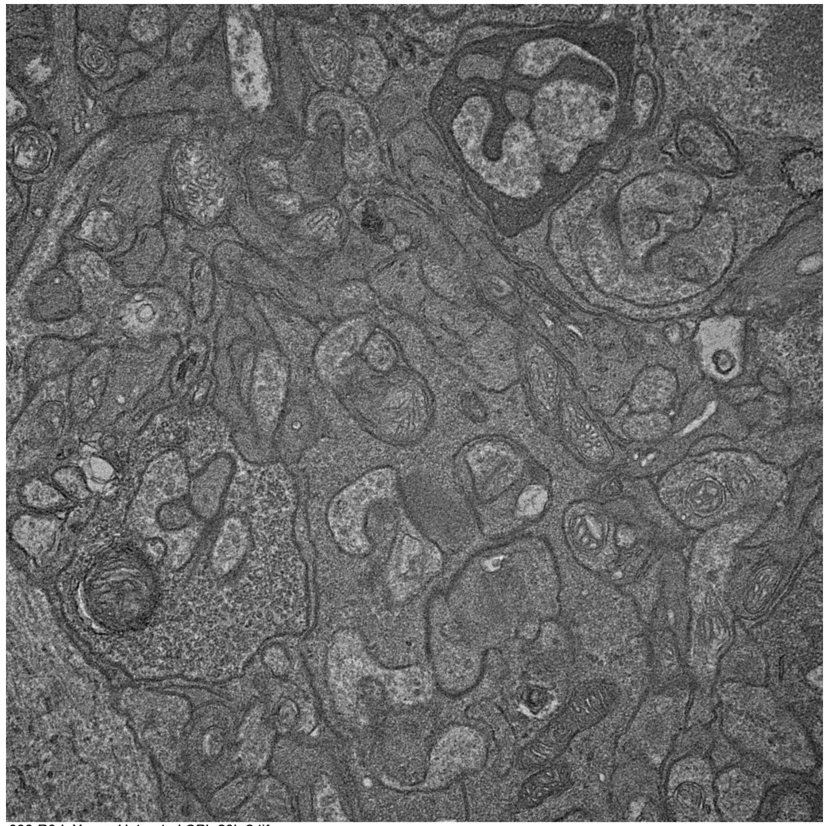

038-R0-L Young Untreated OPL 20k-2.tif
038R0-L
Young Untreated
Print Mag: 23400x @ 7.0 in
11:47:16 2/16/2023

800 nm
HV=80.0kV
Direct Mag: 20000x
Center for Biologic Imaging

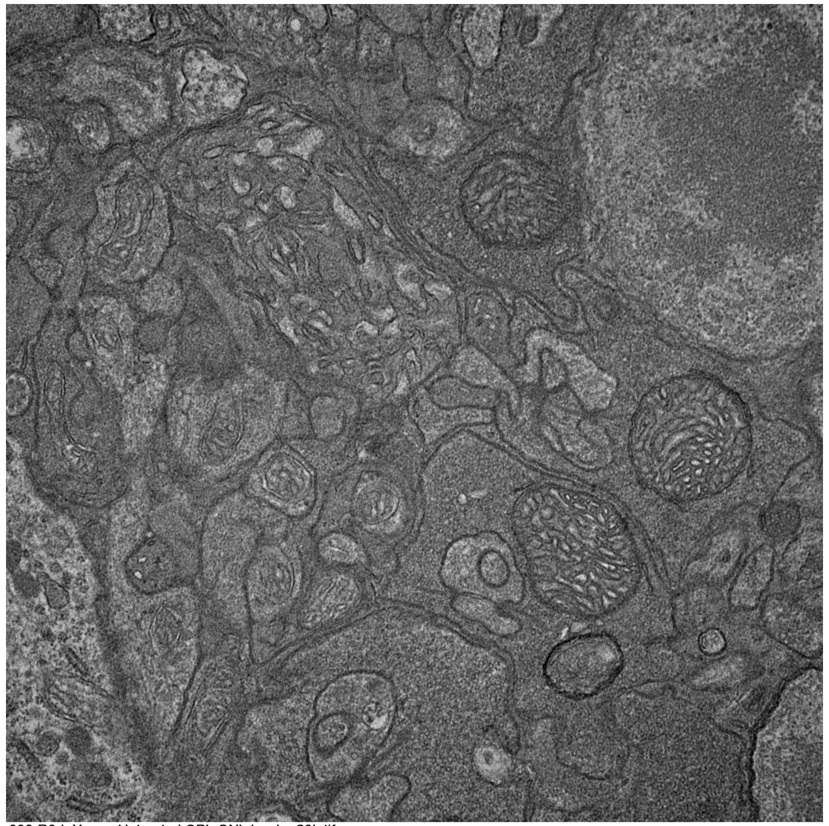

038-R0-L Young Untreated OPL ONL border 20k.tif
038R0-L
Young Untreated
Print Mag: 23400x @ 7.0 in
11:48:22 2/16/2023

800 nm
HV=80.0kV
Direct Mag: 20000x
Center for Biologic Imaging

Central Aged 8-AG Treated

Aged 8-AG Tx RPE (+ choroid)

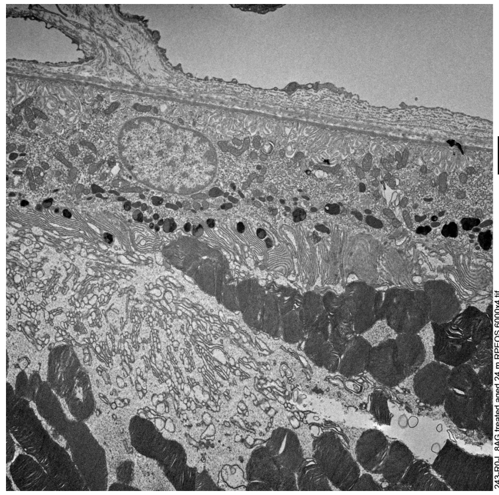

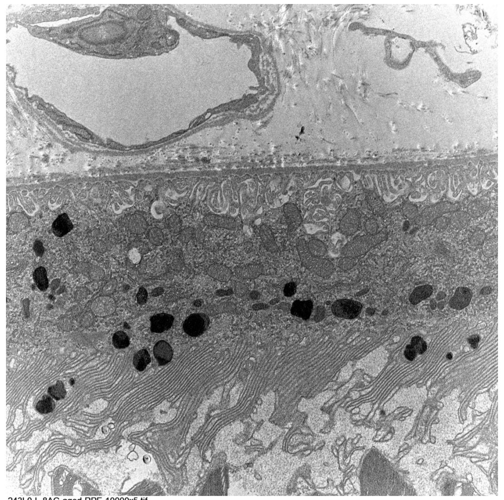

243L0-L-8AG-aged-RPE-10000x5.tif
243L0-L aged 8AG treated
RPE10000x5
Print Mag: 11700x @ 7.0 in
15:30:48 3/8/2023
1 µm
HV=80.0kV
Direct Mag: 10000x
Center for Biologic Imaging

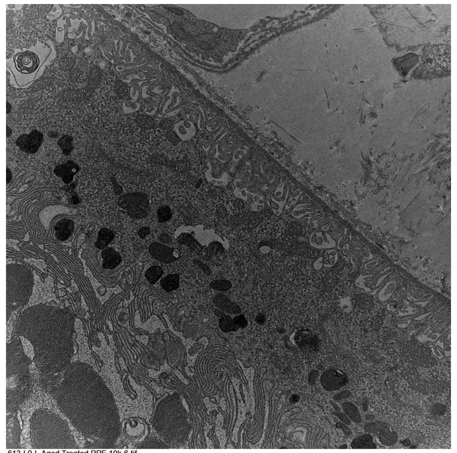

613-L0-L Aged Treated RPE 10k-6.tif
613-L0-L Aged Treated
Print Mag: 11700x @ 7.0 in
14:44:21 4/28/2023
1 µm
HV=80.0kV
Direct Mag: 10000x
Center for Biologic Imaging

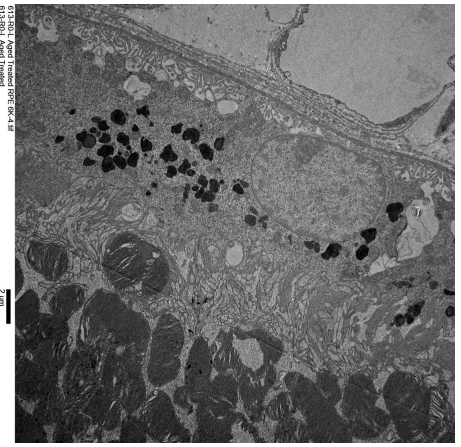

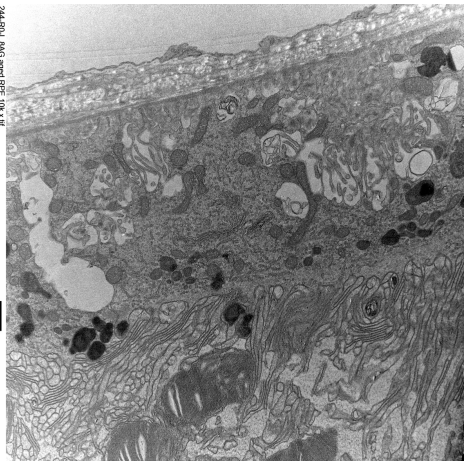

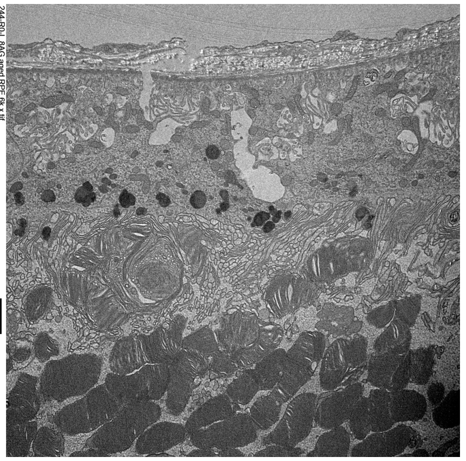

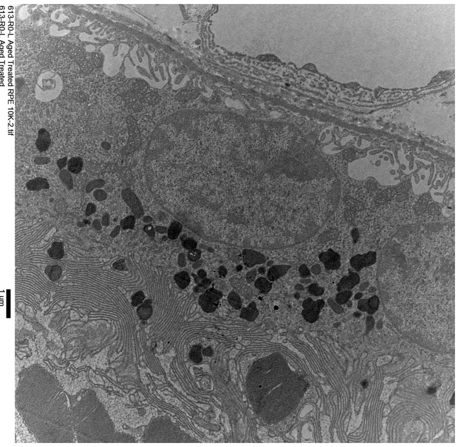

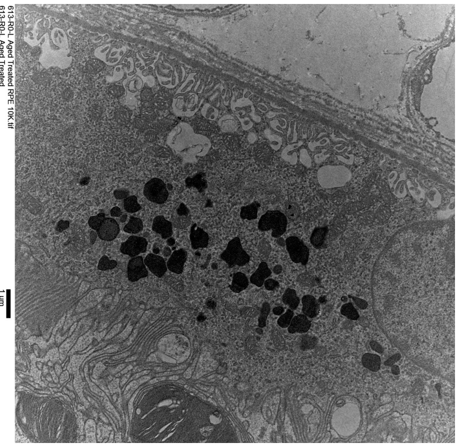

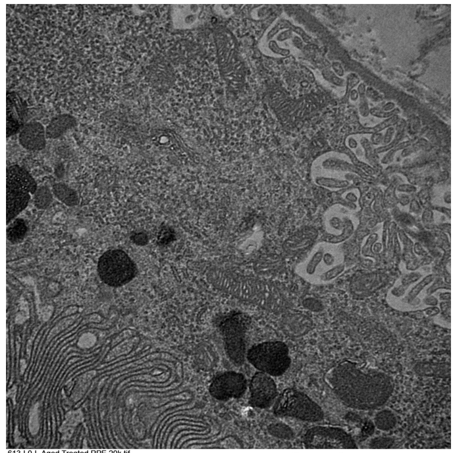

613-L0-L Aged Treated RPE 20k.tif
613-L0-L Aged Treated
Print Mag: 23400x @ 7.0 in
14:41:20 4/28/2023
800 nm
HV=80.0kV
Direct Mag: 20000x
Center for Biologic Imaging

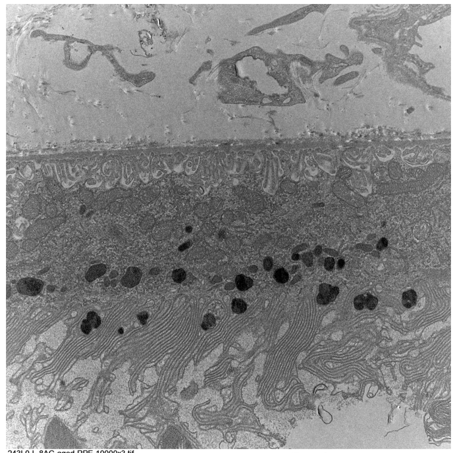

243L0-L-8AG-aged-RPE-10000x3.tif
243L0-L aged 8AG treated
RPE10000x4
Print Mag: 11700x @ 7.0 in
15:29:33 3/8/2023
1 µm
HV=80.0kV
Direct Mag: 10000x
Center for Biologic Imaging

Aged 8-Ag Tx Broadview (RPE, IS/OS, ONL)

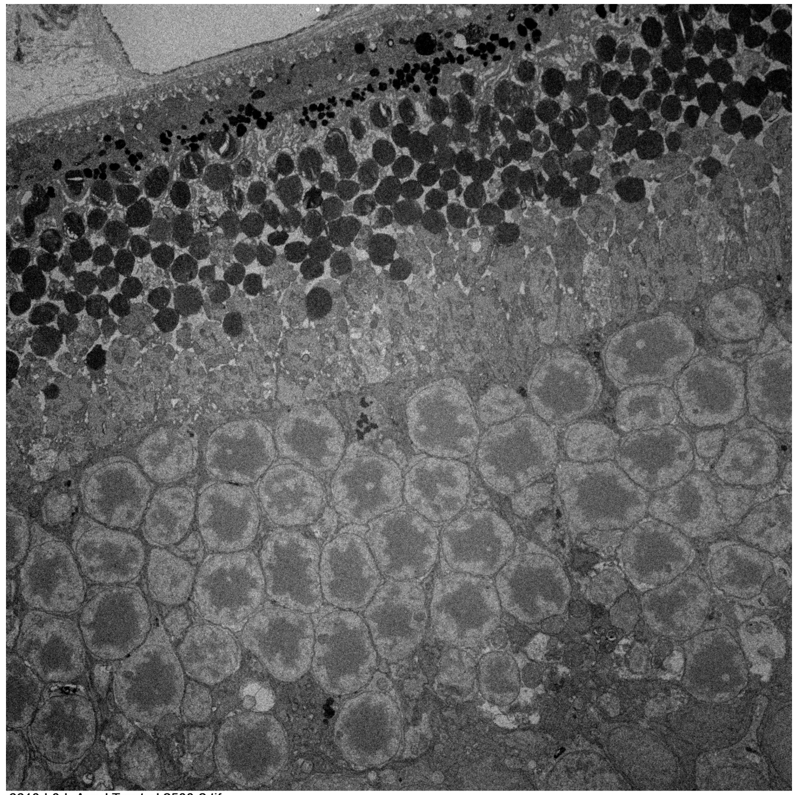

6610-L0-L Aged Treated 2500-2.tif
611-R0-R Aged Untreated
Print Mag: 2920x @ 7.0 in
12:52:17 4/27/2023

6 µm
HV=80.0kV
Direct Mag: 2500x
Center for Biologic Imaging

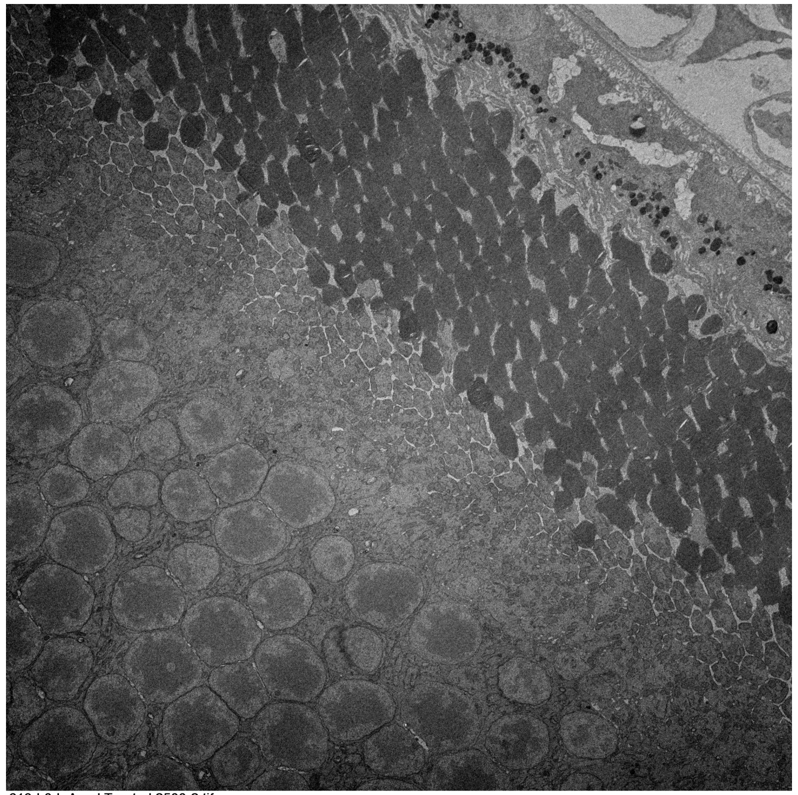

613-L0-L Aged Treated 2500-2.tif
613-L0-L Aged Treated
Print Mag: 2920x @ 7.0 in
14:44:55 4/28/2023

6 µm
HV=80.0kV
Direct Mag: 2500x
Center for Biologic Imaging

Aged 8-AG Tx RPE + OS

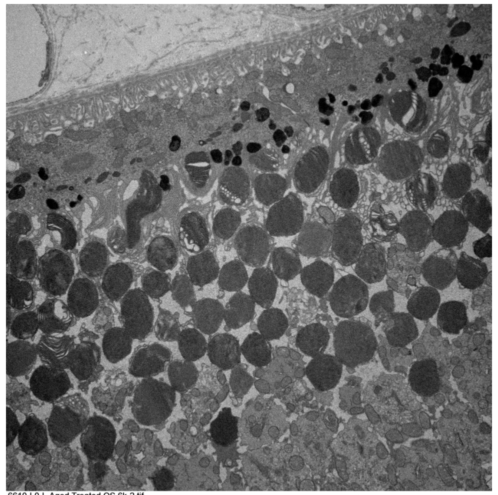

6610-L0-L Aged Treated OS 6k-2.tif
611-R0-R Aged Untreated
Print Mag: 7020x @ 7.0 in
12:47:16 4/27/2023

2 µm
HV=80.0kV
Direct Mag: 6000x
Center for Biologic Imaging

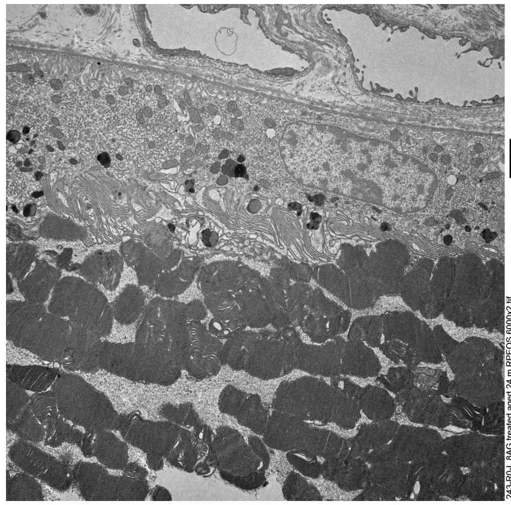

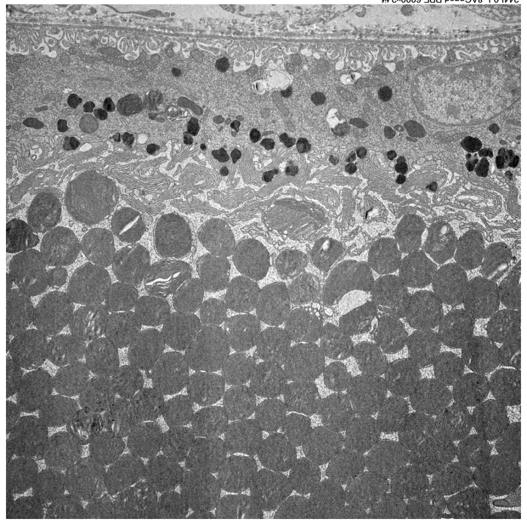

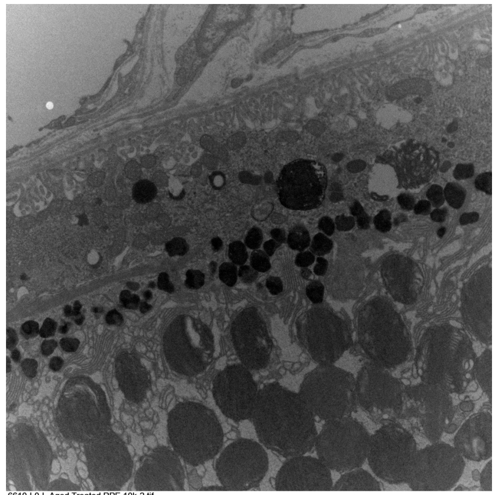

6610-L0-L Aged Treated RPE 10k-2.tif
611-R0-R Aged Untreated
Print Mag: 11700x @ 7.0 in
12:38:11 4/27/2023

1 µm
HV=80.0kV
Direct Mag: 10000x
Center for Biologic Imaging

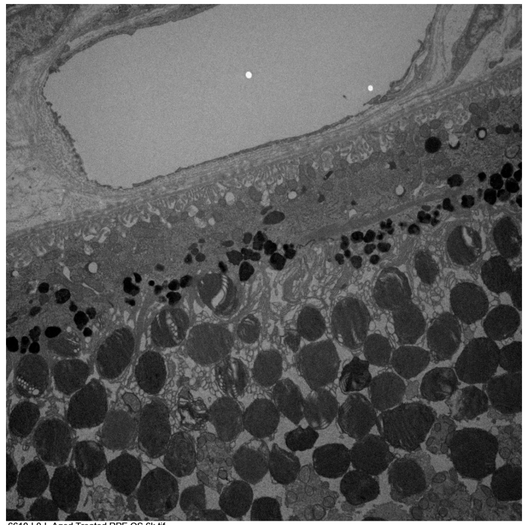

6610-L0-L Aged Treated RPE OS 6k.tif
611-R0-R Aged Untreated
Print Mag: 7020x @ 7.0 in
12:46:35 4/27/2023

2 µm
HV=80.0kV
Direct Mag: 6000x
Center for Biologic Imaging

Aged 8-AG Tx OS

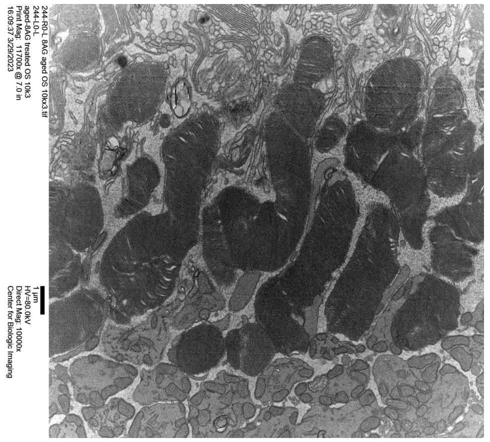

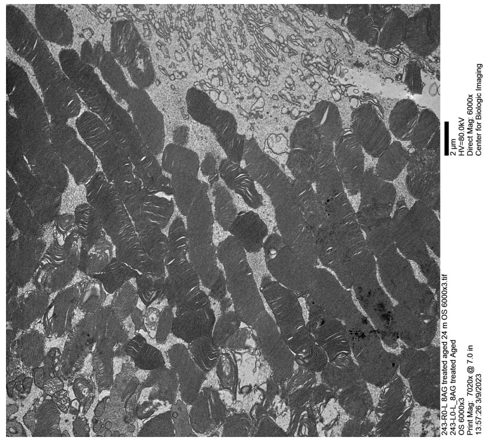

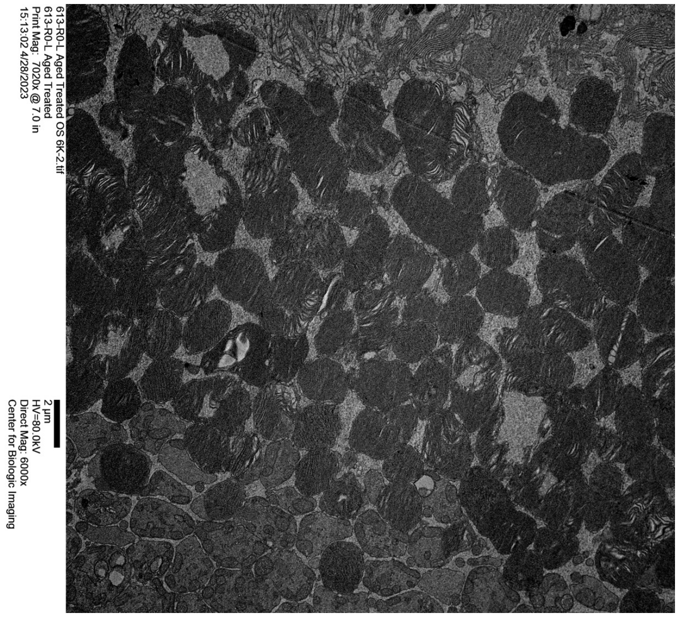

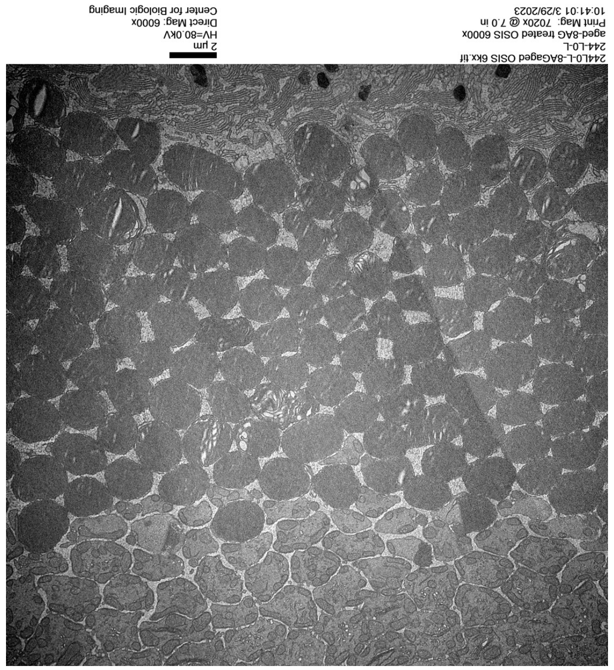

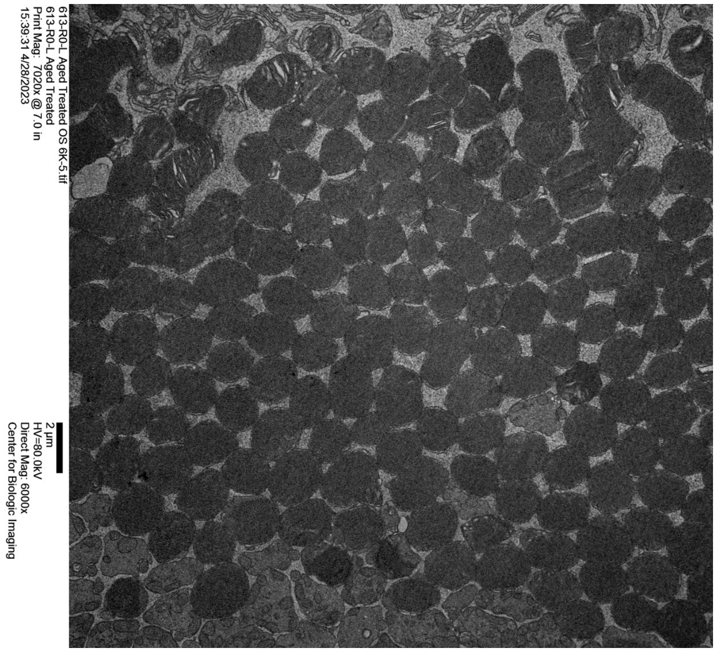

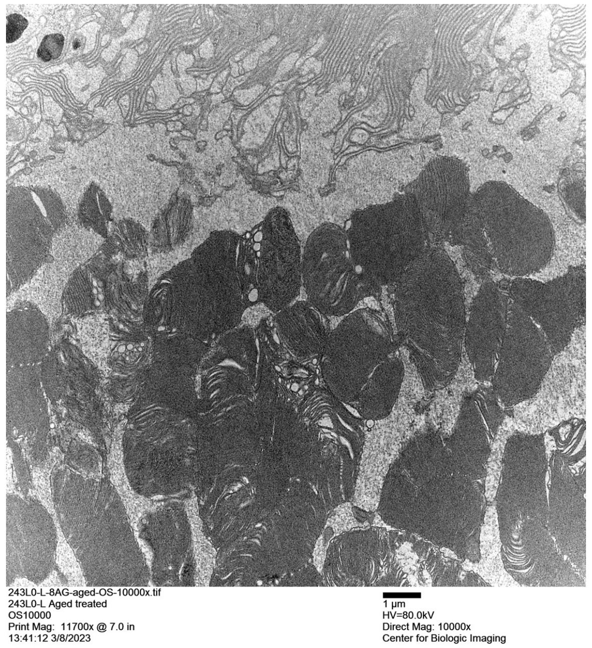

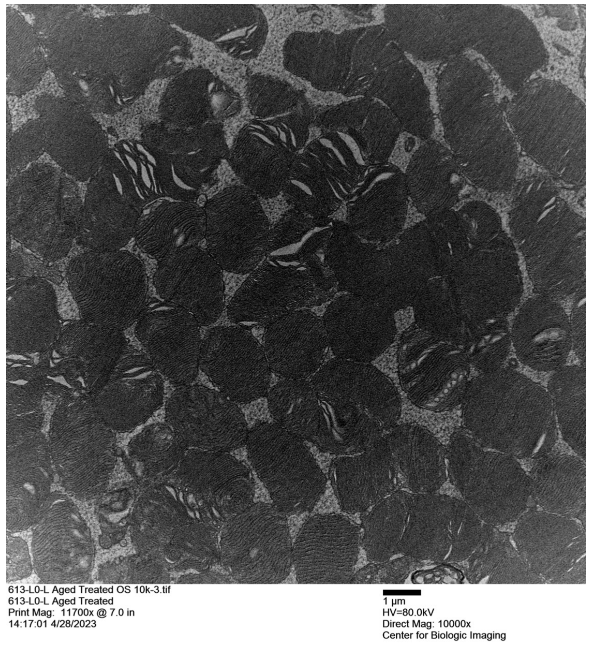

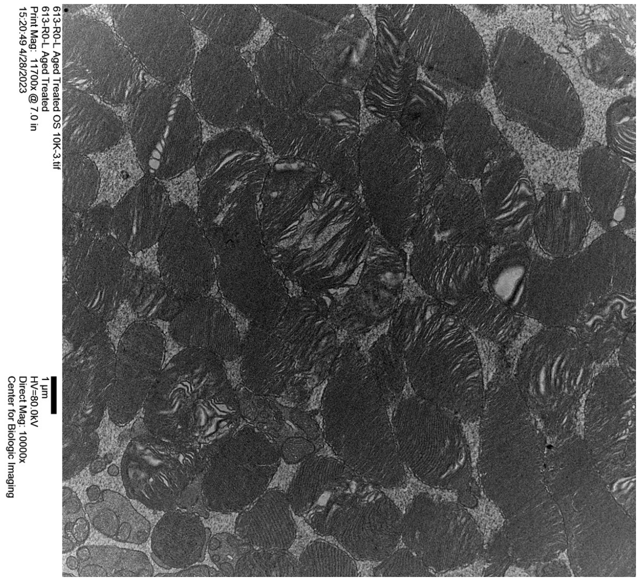

Aged 8-AG IS

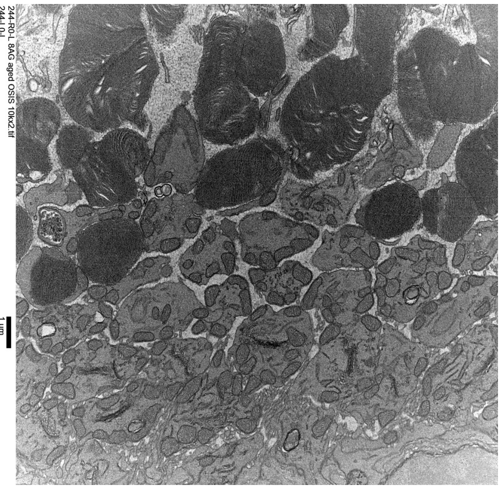

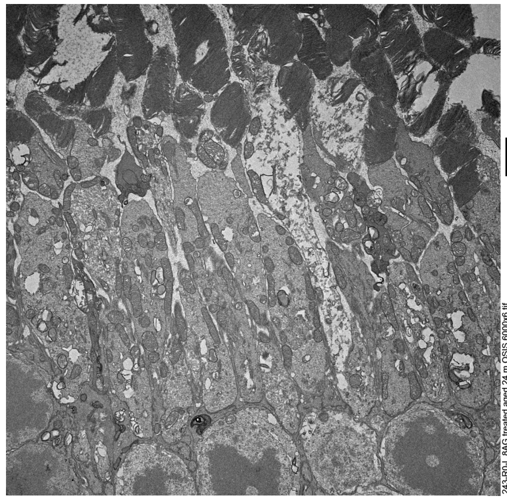

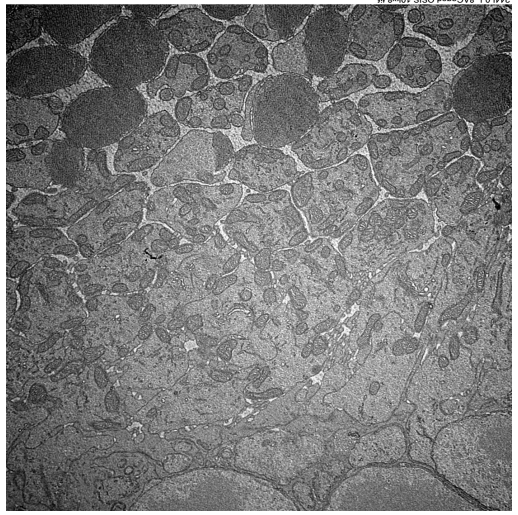

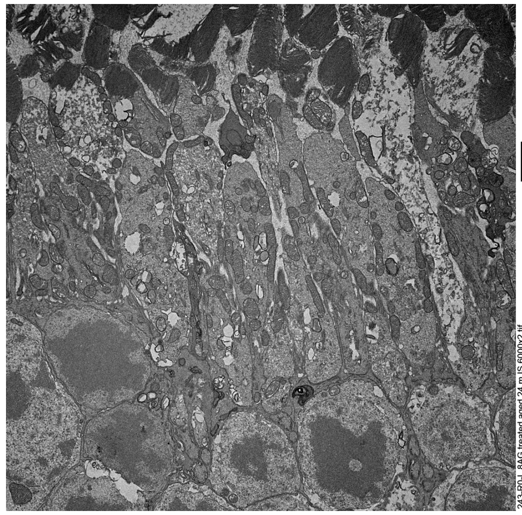

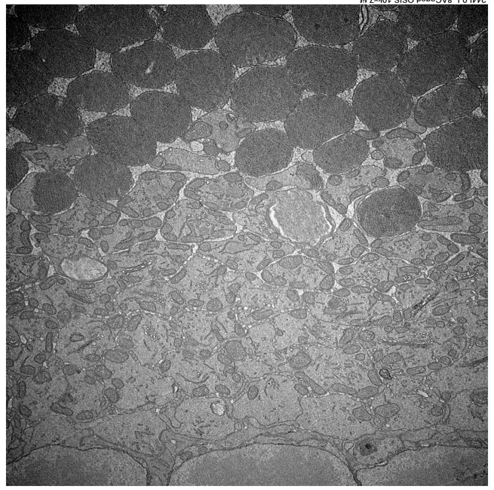

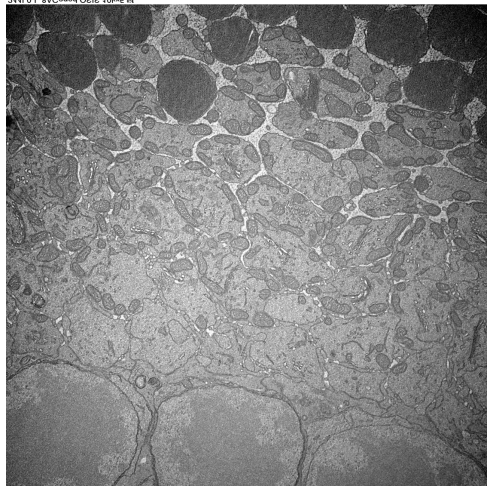

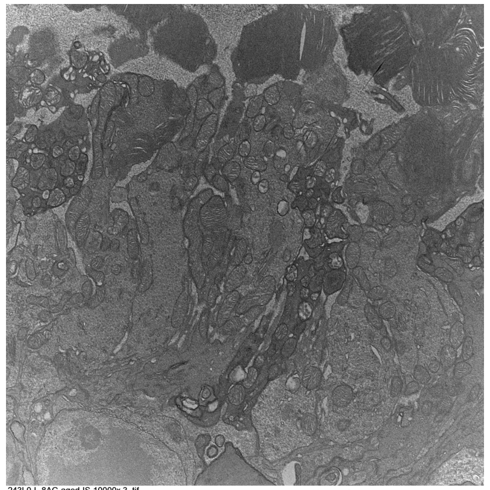
243L0-L-8AG-aged-IS-10000x 3 .tif
243L0-L aged 8AG treated
IS10000x3
Print Mag: 11700x @ 7.0 in
15:52:34 3/8/2023
1 μm
HV=80.0kV
Direct Mag: 10000x
Center for Biologic Imaging

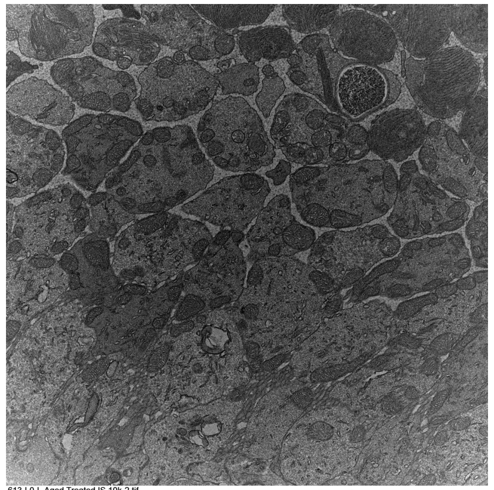
613-L0-L Aged Treated IS 10k-2.tif
613-L0-L Aged Treated
Print Mag: 11700x @ 7.0 in
14:15:46 4/28/2023
1 μm
HV=80.0kV
Direct Mag: 10000x
Center for Biologic Imaging

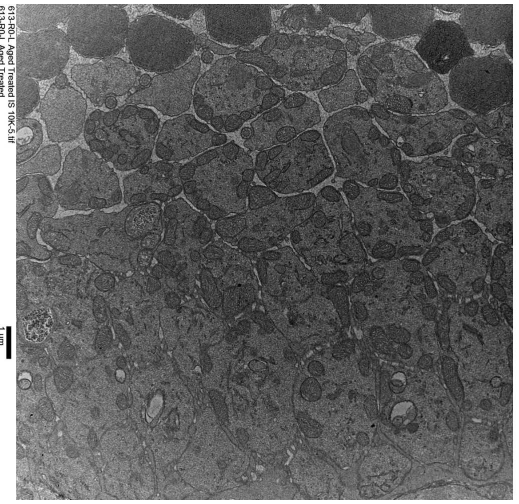

Aged 8-AG Tx IS/OS/ONL

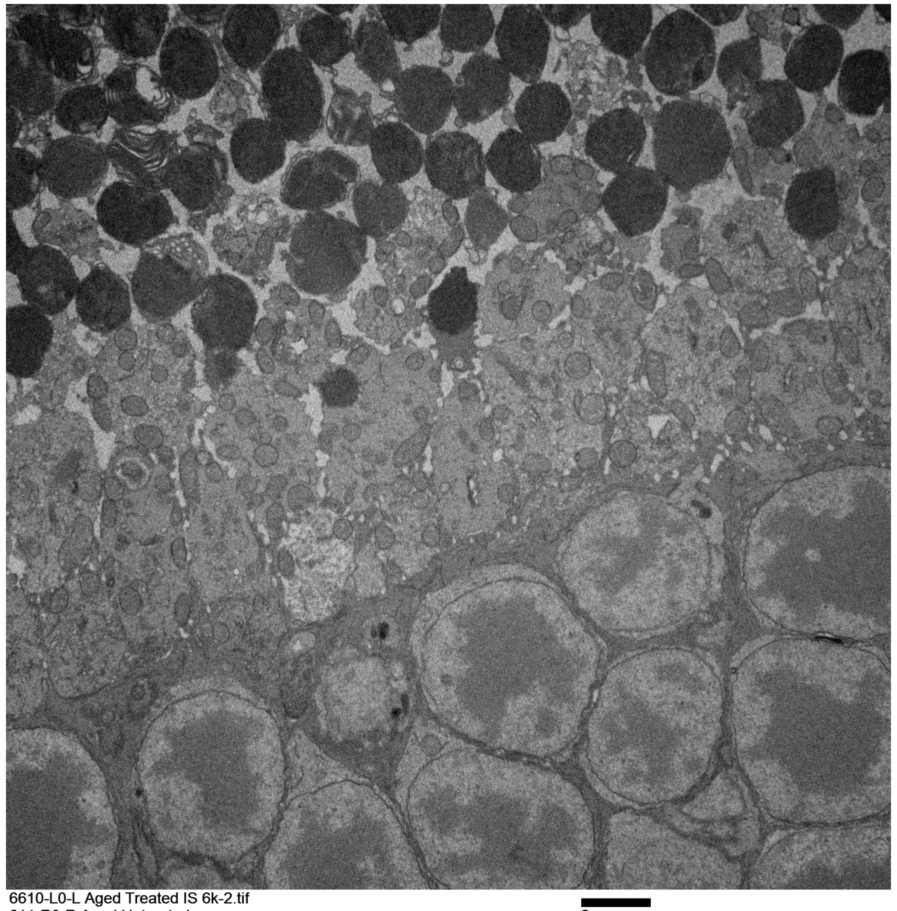

6610-L0-L Aged Treated IS 6k-2.tif
611-R0-R Aged Untreated
Print Mag: 7020x @ 7.0 in
12:47:50 4/27/2023

2 µm
HV=80.0kV
Direct Mag: 6000x
Center for Biologic Imaging

Aged 8-AG Tx ONL

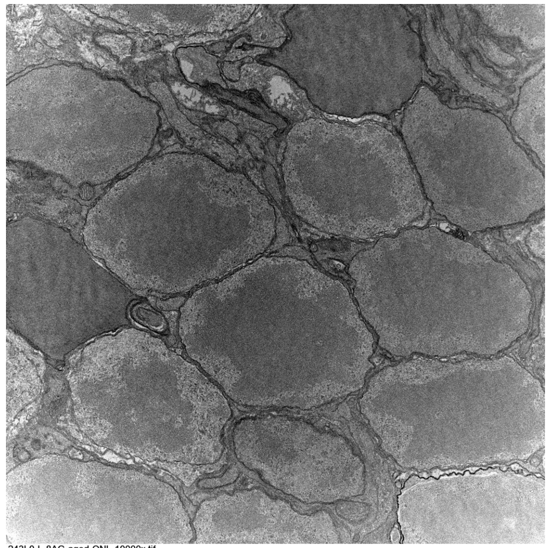

243L0-L-8AG-aged-ONL-10000x.tif
243L0-L aged 8AG treated
ONL 10000x
Print Mag: 11700x @ 7.0 in
14:04:10 3/8/2023

1 µm
HV=80.0kV
Direct Mag: 10000x
Center for Biologic Imaging

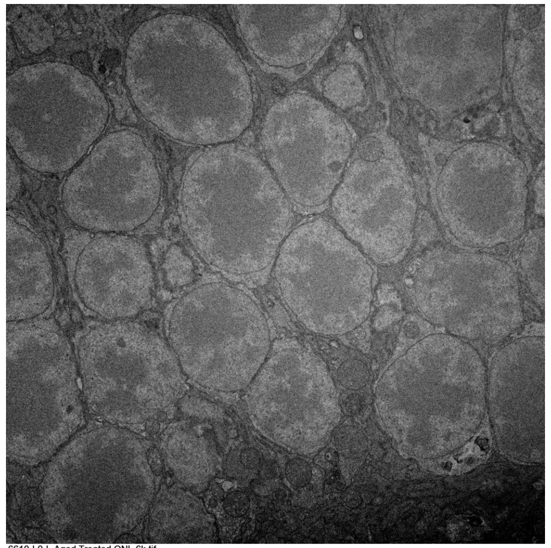

6610-L0-L Aged Treated ONL 6k.tif
611-R0-R Aged Untreated
Print Mag: 7020x @ 7.0 in
12:50:25 4/27/2023

2 µm
HV=80.0kV
Direct Mag: 6000x
Center for Biologic Imaging

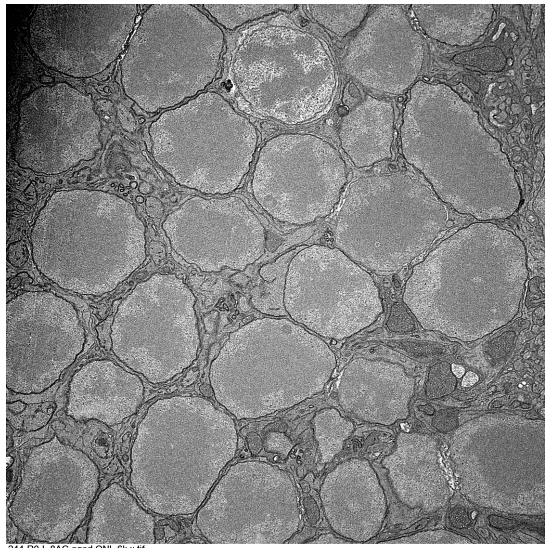

244-R0-L 8AG aged ONL 6k x.tif
244-L0-L
aged-8AG treated ONL 6kx
Print Mag: 7020x @ 7.0 in
16:31:10 3/29/2023

2 µm
HV=80.0kV
Direct Mag: 6000x
Center for Biologic Imaging

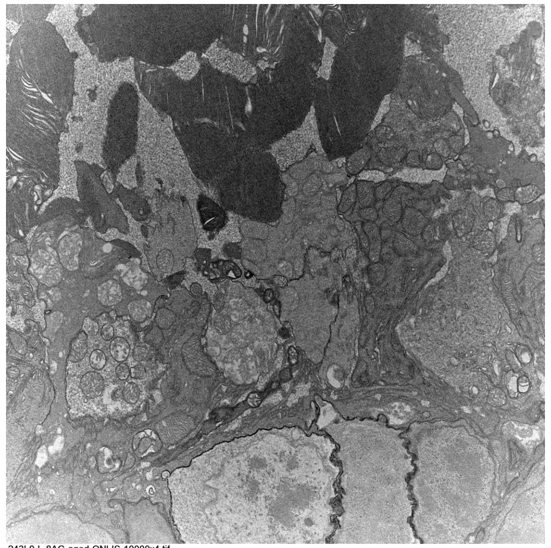

243L0-L-8AG-aged-ONLIS-10000x4.tif
243L0-L aged 8AG treated
IS_ONL 10000x 4
Print Mag: 11700x @ 7.0 in
14:19:51 3/8/2023

1 µm
HV=80.0kV
Direct Mag: 10000x
Center for Biologic Imaging

Aged 8-AG Tx OPL

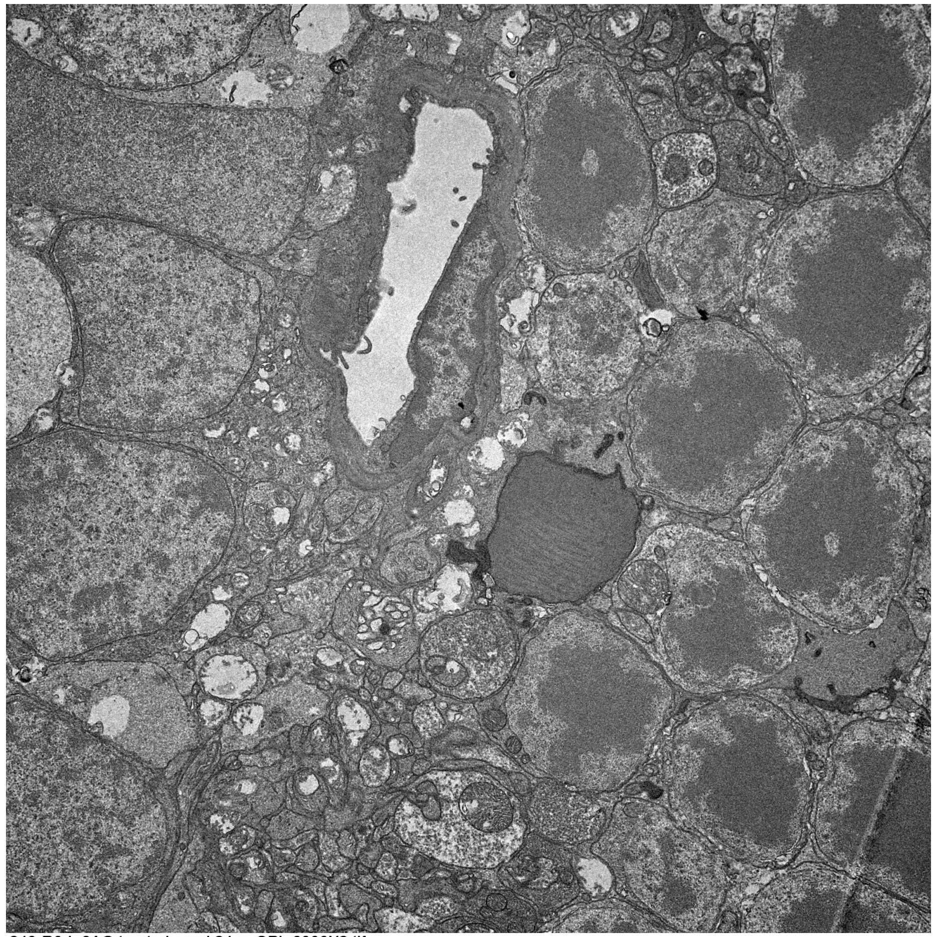

243-R0-L 8AG treated aged 24 m OPL 6000X2.tif
243-L0-L_8AG treated Aged
OPL 6000X2
Print Mag: 7020x @ 7.0 in
14:21:40 3/9/2023

2 µm
HV=80.0kV
Direct Mag: 6000x
Center for Biologic Imaging

Aged 8-AG Tx IPL

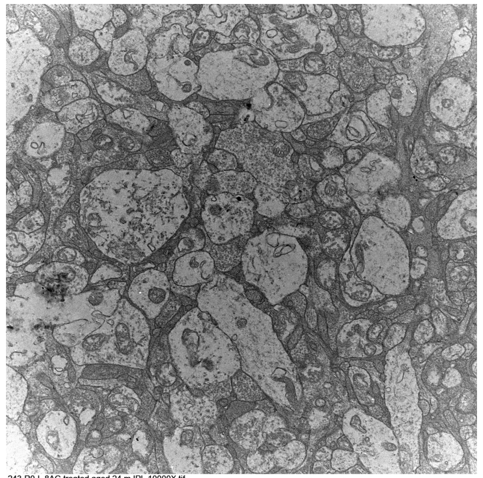

243-R0-L 8AG treated aged 24 m IPL 10000X.tif
243-L0-L_8AG treated Aged
IPL 10000X
Print Mag: 11700x @ 7.0 in
15:32:03 3/9/2023

1 µm
HV=80.0kV
Direct Mag: 10000x
Center for Biologic Imaging

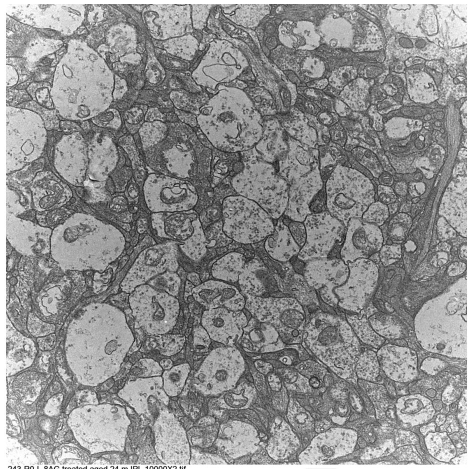

243-R0-L 8AG treated aged 24 m IPL 10000X2.tif
243-L0-L_8AG treated Aged
IPL 10000X2
Print Mag: 11700x @ 7.0 in
15:34:39 3/9/2023

1 µm
HV=80.0kV
Direct Mag: 10000x
Center for Biologic Imaging

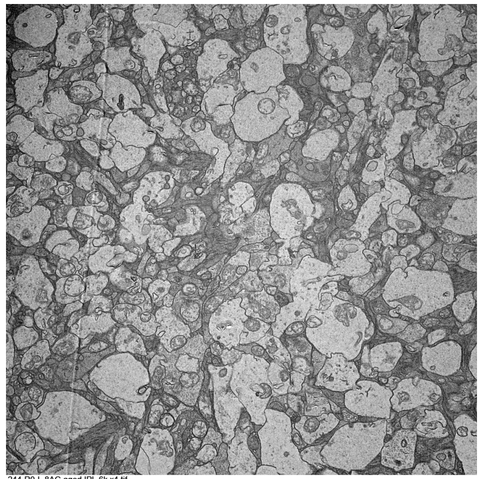

244-R0-L 8AG aged IPL 6k x4.tif
244-L0-L
aged-8AG treated IPL 6kx 4
Print Mag: 7020x @ 7.0 in
16:59:48 3/29/2023

2 µm
HV=80.0kV
Direct Mag: 6000x
Center for Biologic Imaging

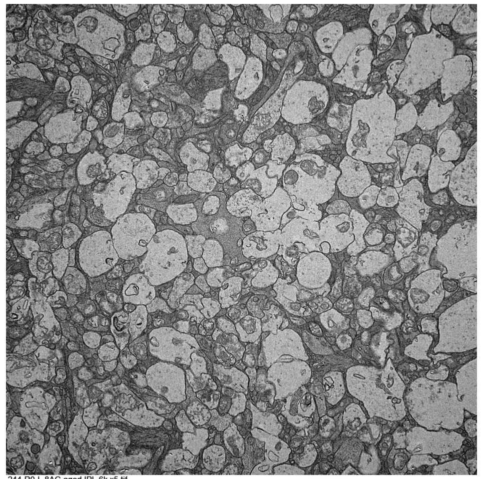

244-R0-L 8AG aged IPL 6k x5.tif
244-L0-L
aged-8AG treated IPL 6kx 5
Print Mag: 7020x @ 7.0 in
17:00:56 3/29/2023

2 µm
HV=80.0kV
Direct Mag: 6000x
Center for Biologic Imaging

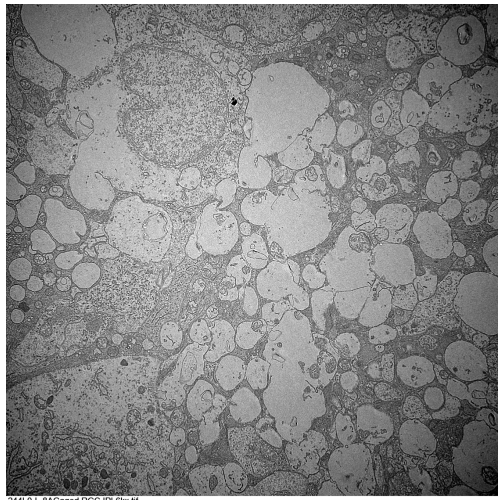

244L0-L-8AGaged RGC IPL6kx.tif
244-L0-L
aged-8AG treated RGC IPL 6kx
Print Mag: 7020x @ 7.0 in
14:01:14 3/29/2023

2 µm
HV=80.0kV
Direct Mag: 6000x
Center for Biologic Imaging

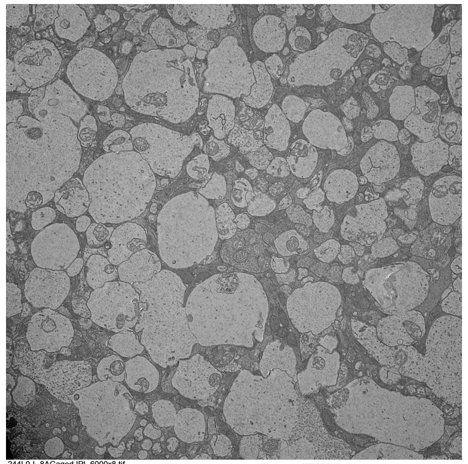

244L0-L-8AGaged IPL 6000x8.tif
244-L0-L
aged-8AG treated IPL 6kx2
Print Mag: 7020x @ 7.0 in
14:14:36 3/29/2023

2 µm
HV=80.0kV
Direct Mag: 6000x
Center for Biologic Imaging

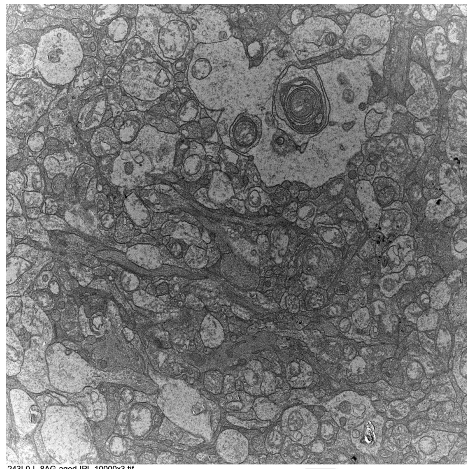

243L0-L-8AG-aged-IPL-10000x3.tif
243L0-L aged 8AG treated
IPL10000x3
Print Mag: 11700x @ 7.0 in
15:18:42 3/8/2023

1 µm
HV=80.0kV
Direct Mag: 10000x
Center for Biologic Imaging

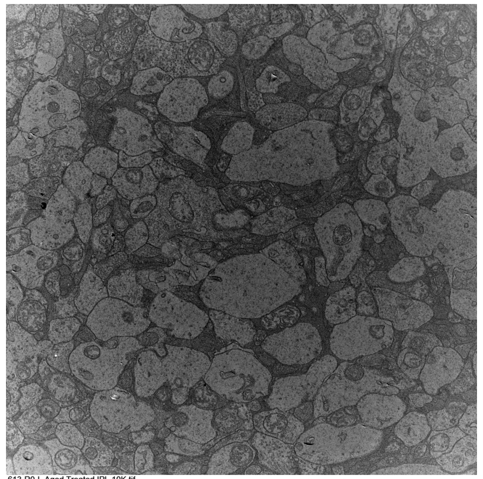

613-R0-L Aged Treated IPL 10K.tif
613-R0-L Aged Treated
Print Mag: 11700x @ 7.0 in
15:28:31 4/28/2023

1 µm
HV=80.0kV
Direct Mag: 10000x
Center for Biologic Imaging

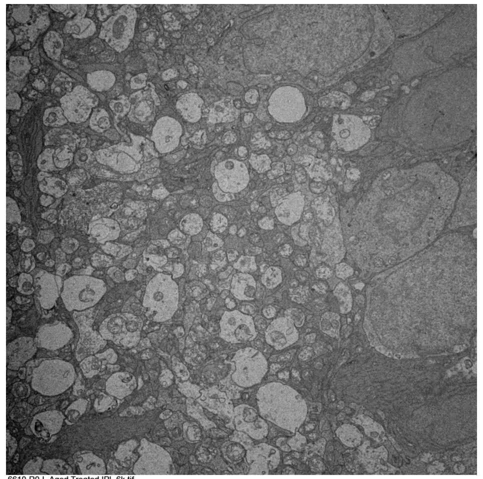

6610-R0-L Aged Treated IPL 6k.tif
6610-R0-L Aged Treated
Print Mag: 7020x @ 7.0 in
13:30:53 4/28/2023

2 µm
HV=80.0kV
Direct Mag: 6000x
Center for Biologic Imaging

Central Retina Young Untreated

Young Untreated RPE

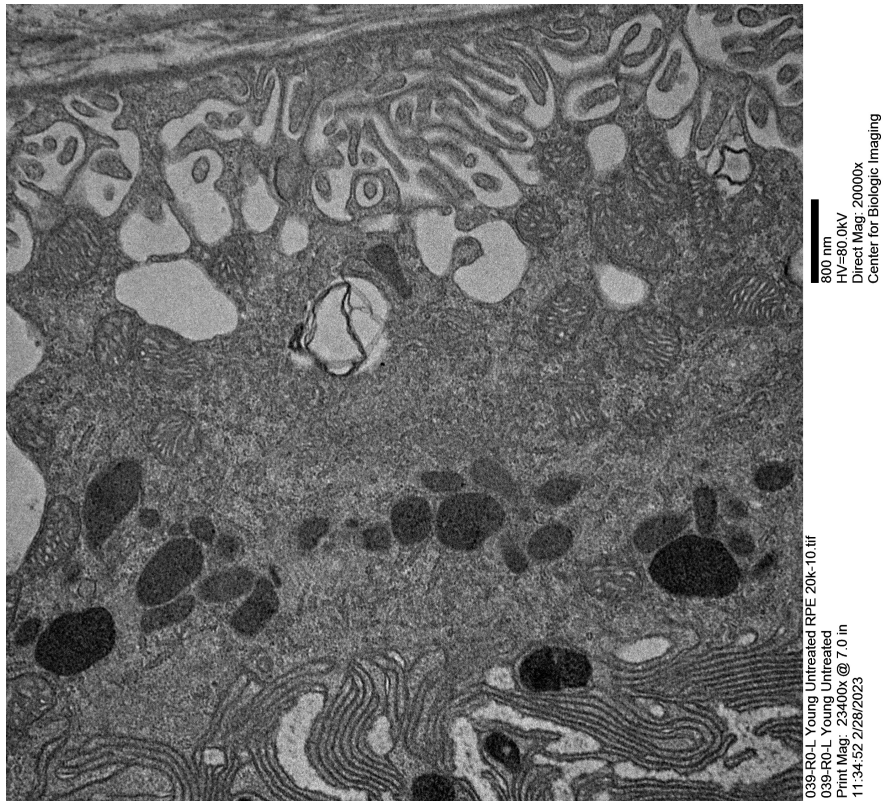

Young Untreated RPE + OS

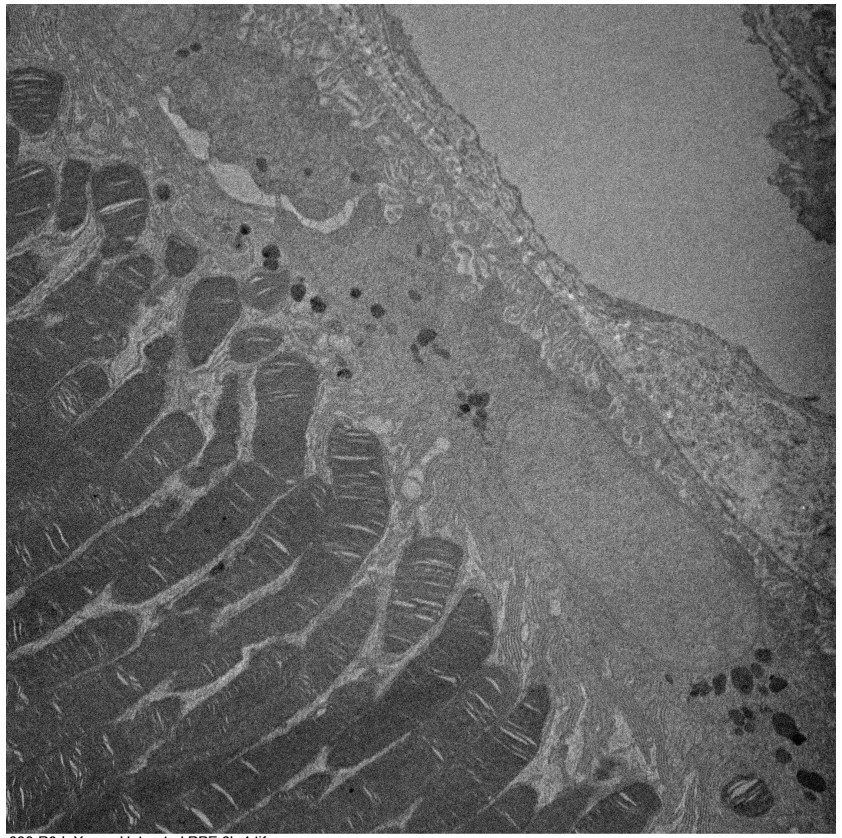

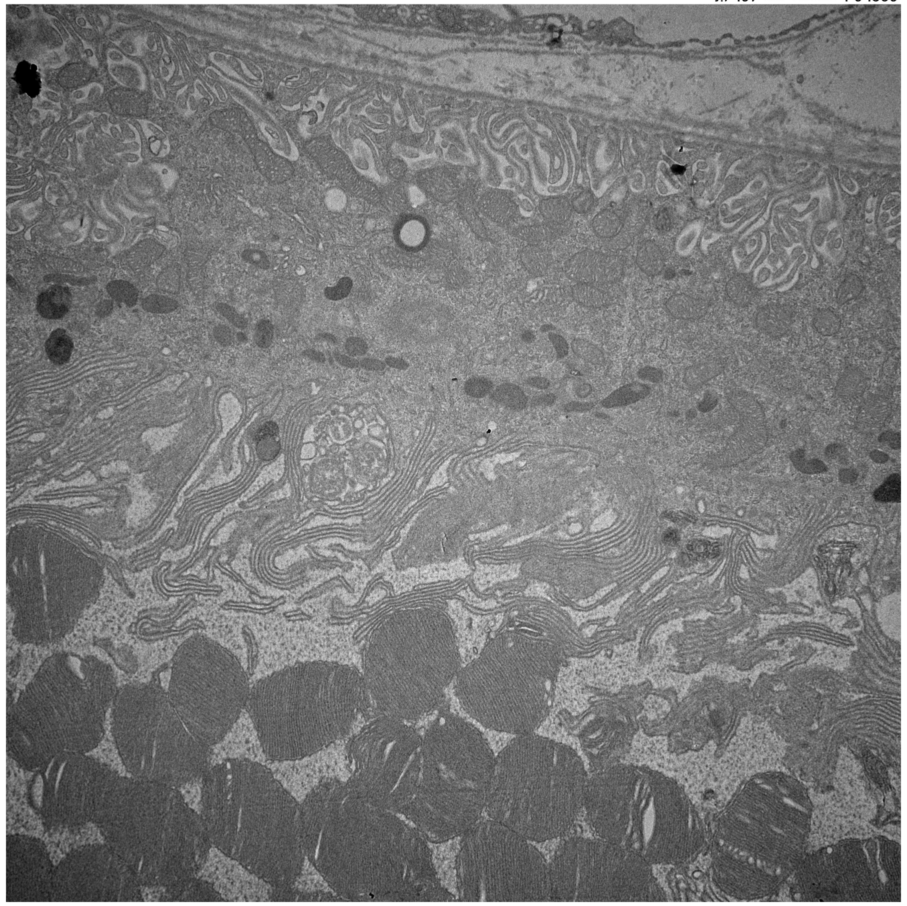

Young untreated OS

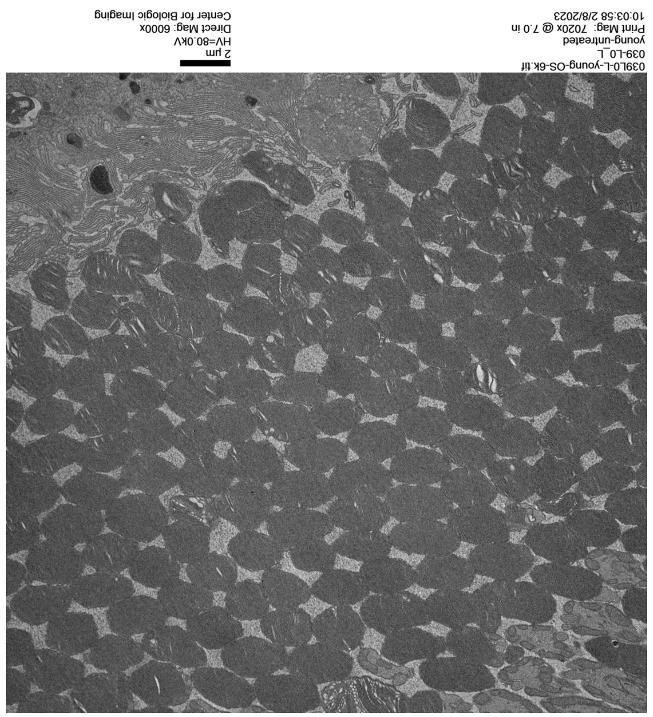

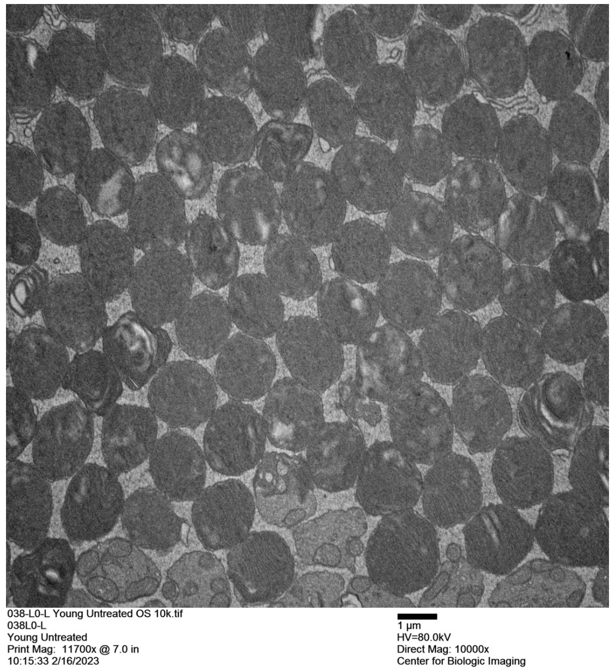

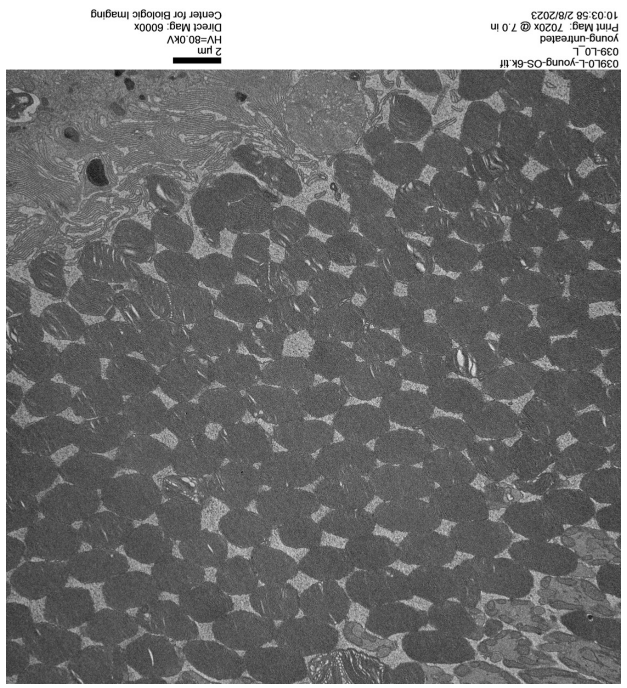

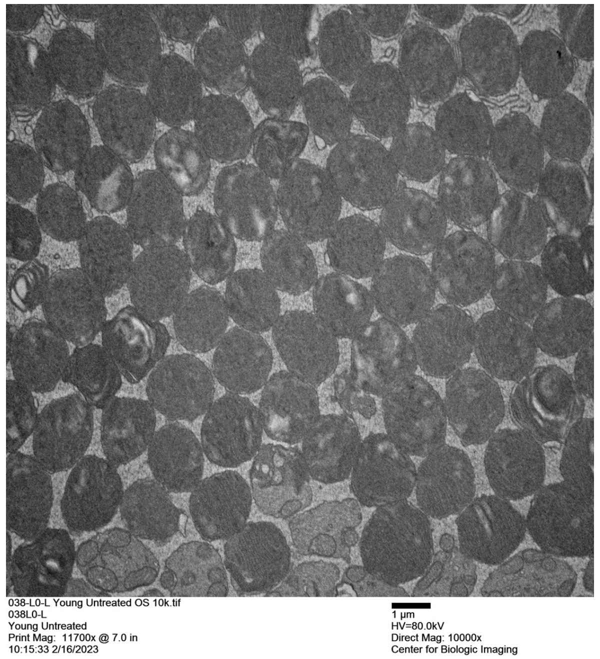

Young untreated IS

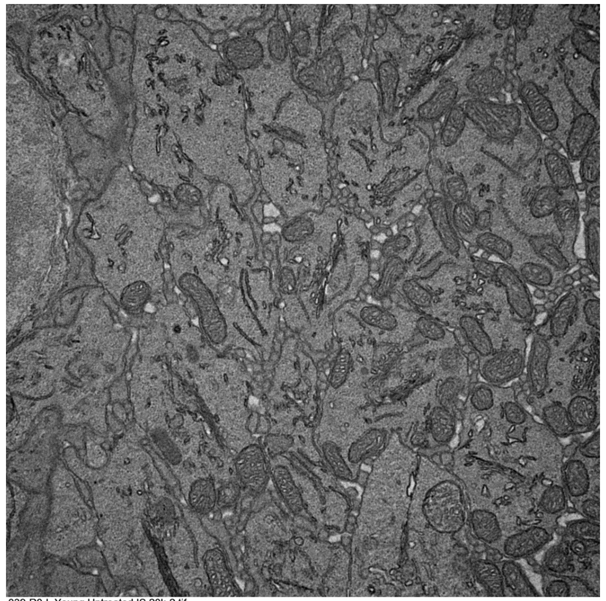

039-R0-L Young Untreated IS 20k-2.tif
039-R0-L Young Untreated
Print Mag: 23400x @ 7.0 in
10:53:33 2/28/2023

800 nm
HV=80.0kV
Direct Mag: 20000x
Center for Biologic Imaging

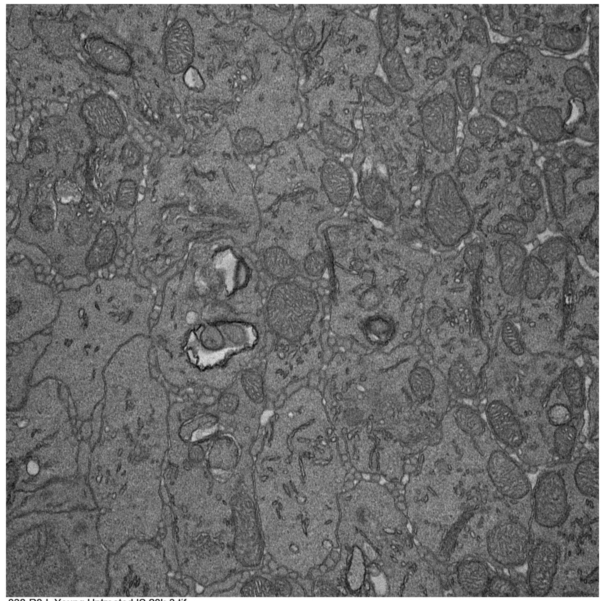

038-R0-L Young Untreated IS 20k-3.tif
038R0-L
Young Untreated
Print Mag: 23400x @ 7.0 in
11:38:10 2/16/2023

800 nm
HV=80.0kV
Direct Mag: 20000x
Center for Biologic Imaging

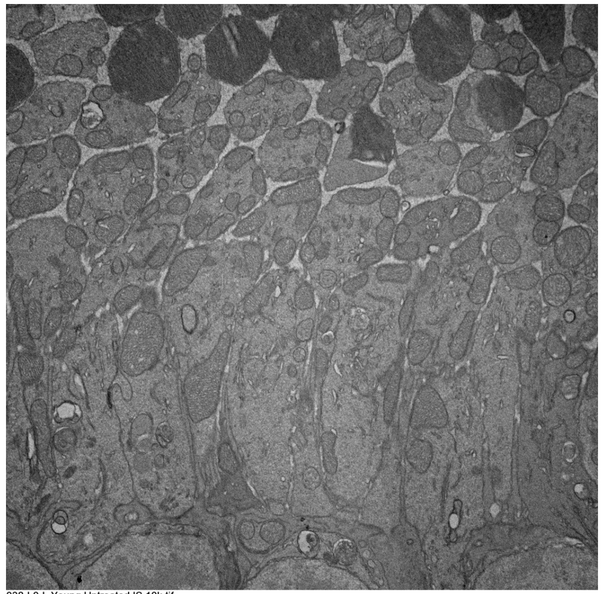

038-L0-L Young Untreated IS 10k.tif
038L0-L
Young Untreated
Print Mag: 11700x @ 7.0 in
10:18:49 2/16/2023

1 µm
HV=80.0kV
Direct Mag: 10000x
Center for Biologic Imaging

Young untreated ONL

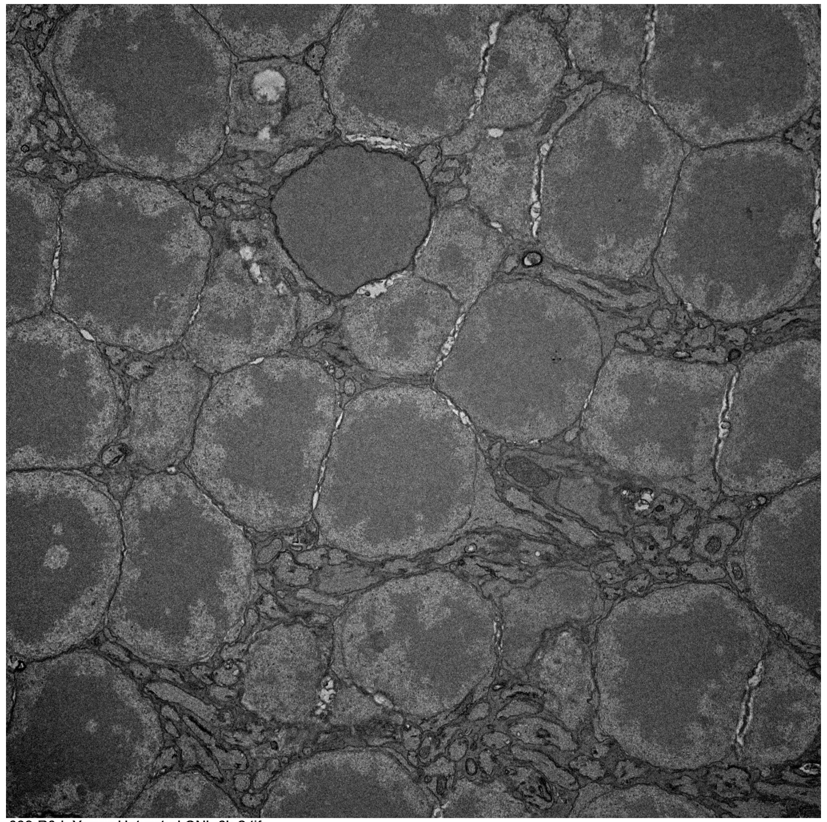

039-R0-L Young Untreated ONL 6k-2.tif
039-R0-L Young Untreated
Print Mag: 7020x @ 7.0 in
10:21:42 2/28/2023

2 µm
HV=80.0kV
Direct Mag: 6000x
Center for Biologic Imaging

Young untreated IPL

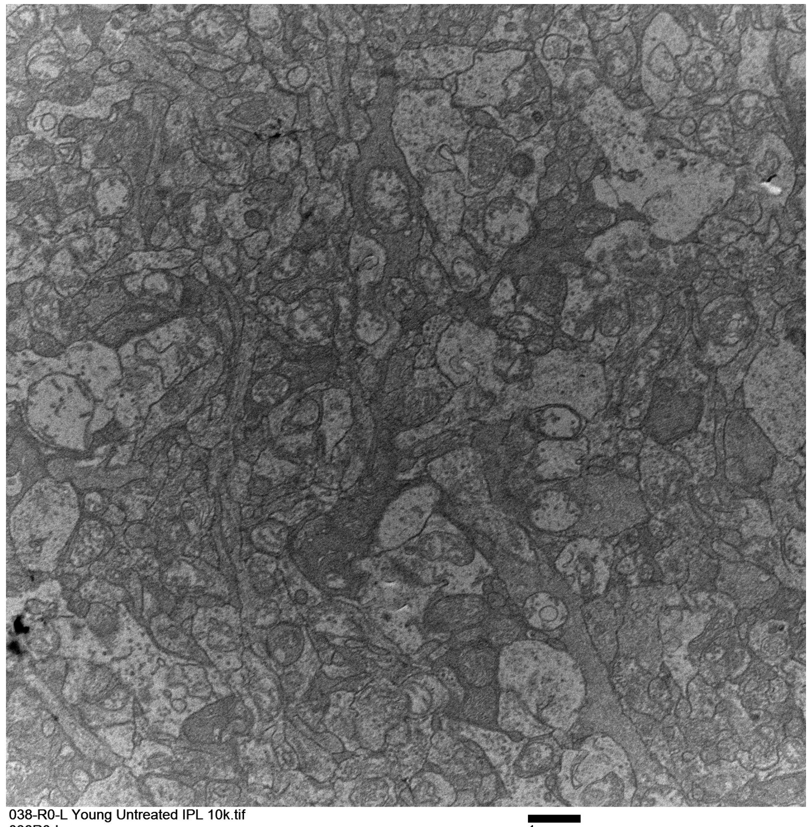

038-R0-L Young Untreated IPL 10k.tif
038R0-L
Young Untreated
Print Mag: 11700x @ 7.0 in
11:49:44 2/16/2023

1 µm
HV=80.0kV
Direct Mag: 10000x
Center for Biologic Imaging

Aged Untreated Central Retinae

Aged Untreated RPE/Choroid

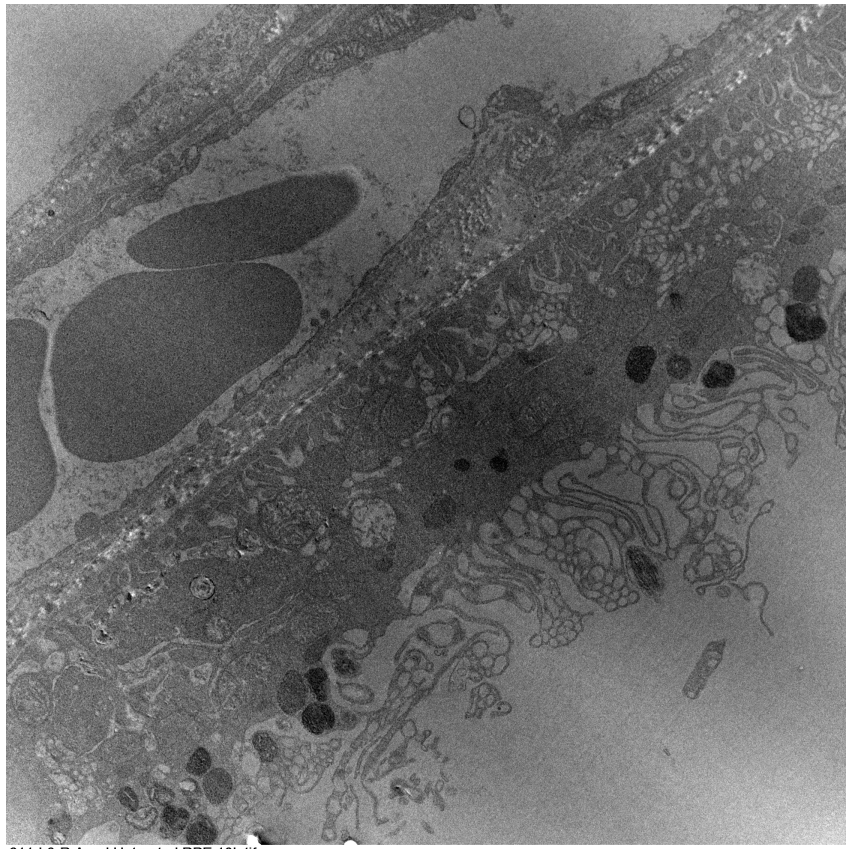

611-L0-R Aged Untreated RPE 10k.tif
6609-L0-L Aged Untreated 24mo
Print Mag: 11700x @ 7.0 in
15:29:56 4/13/2023

1 µm
HV=80.0kV
Direct Mag: 10000x
Center for Biologic Imaging

Aged Untreated RPE

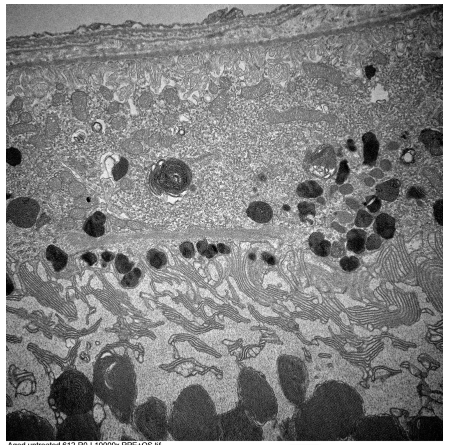

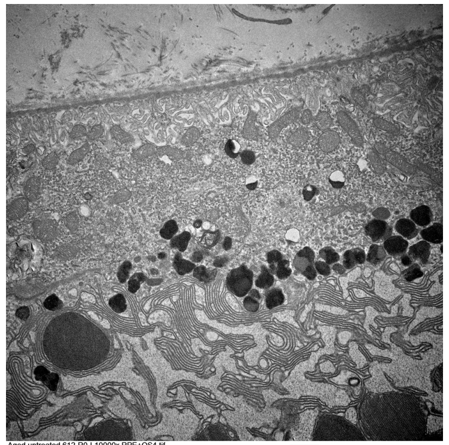

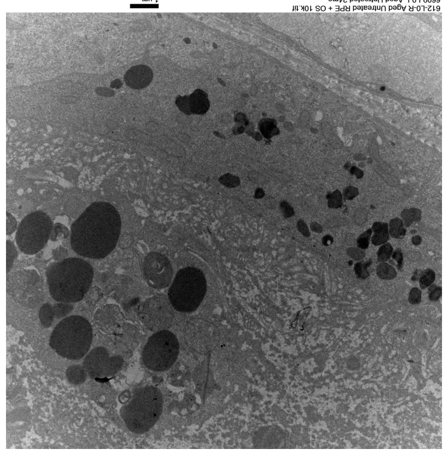

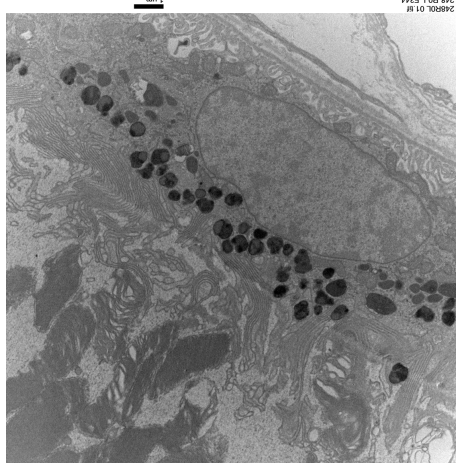

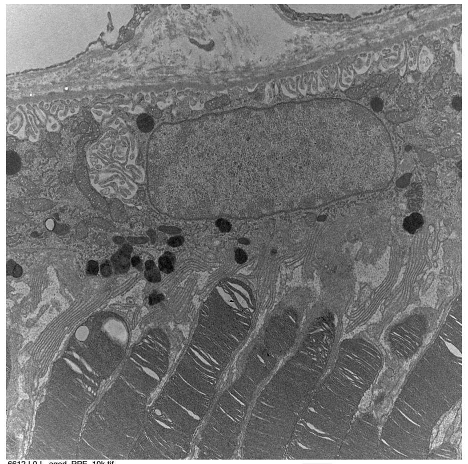

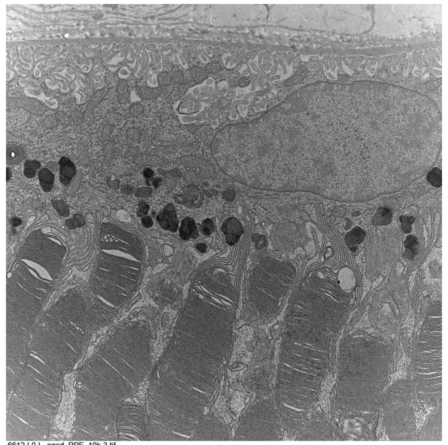

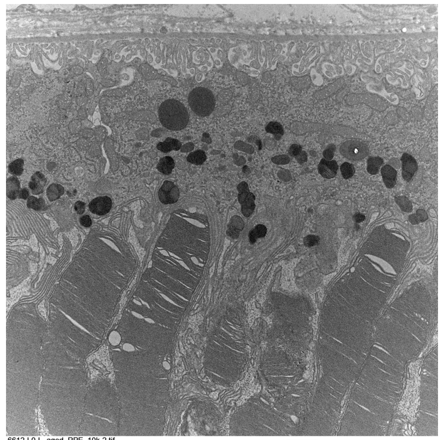

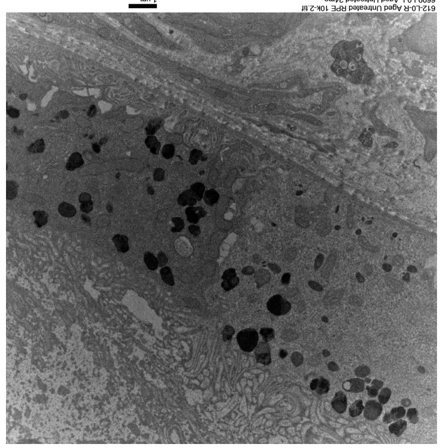

Aged Untreated RPE

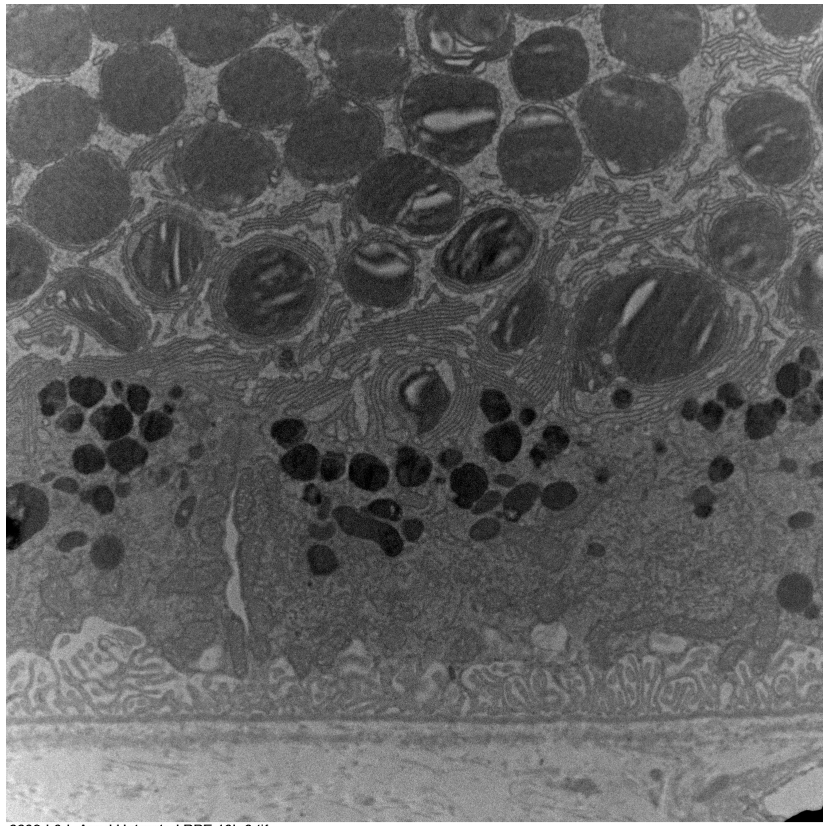

6609-L0-L Aged Untreated RPE 10k-3.tif
6609-L0-L Aged Untreated 24mo
Print Mag: 11700x @ 7.0 in
13:37:33 4/13/2023

1 µm
HV=80.0kV
Direct Mag: 10000x
Center for Biologic Imaging

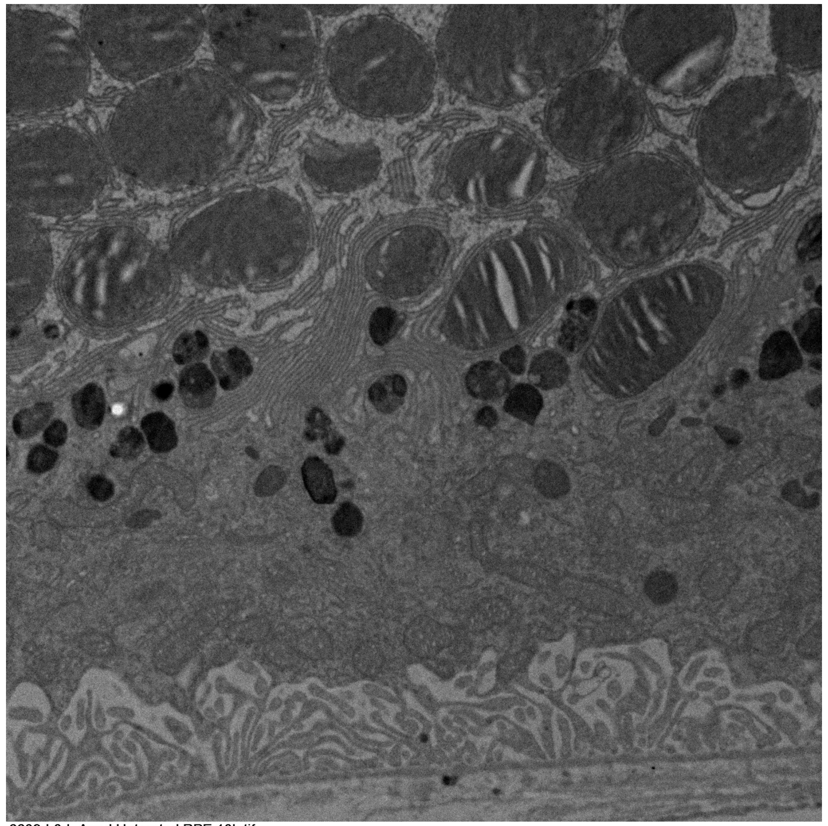

6609-L0-L Aged Untreated RPE 10k.tif
6609-L0-L Aged Untreated 24mo
Print Mag: 14000x @ 7.0 in
13:35:27 4/13/2023

1 µm
HV=80.0kV
Direct Mag: 12000x
Center for Biologic Imaging

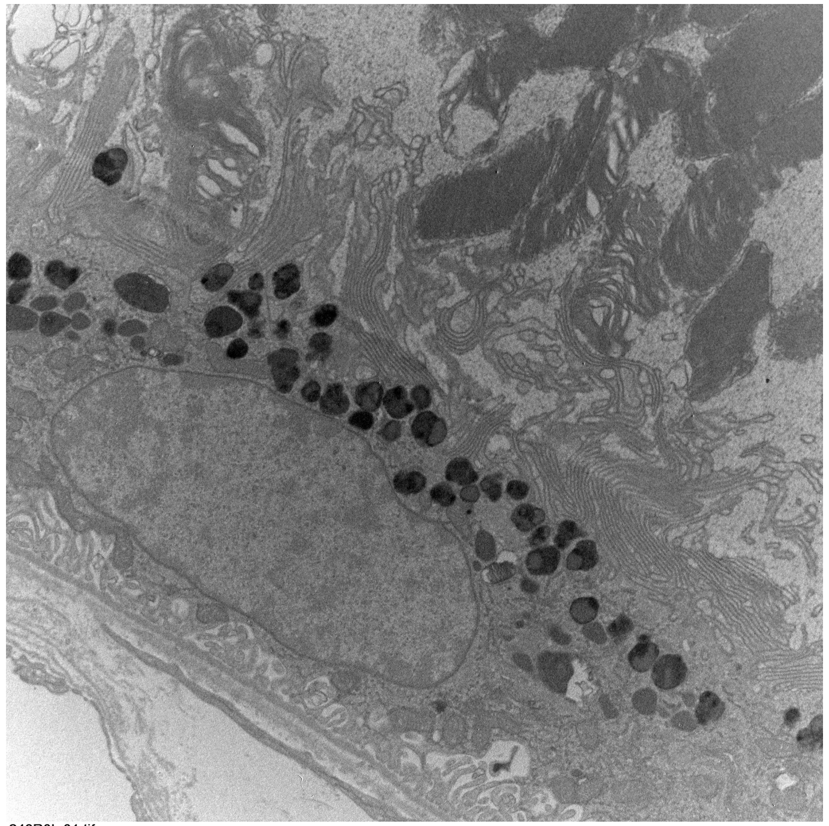

248R0L 01.tif
248-R0-L F344
aged untreated female
Print Mag: 11700x @ 7.0 in
13:35:39 2/2/2023

1 µm
HV=80.0kV
Direct Mag: 10000x
Center for Biologic Imaging

Aged Untreated RPE

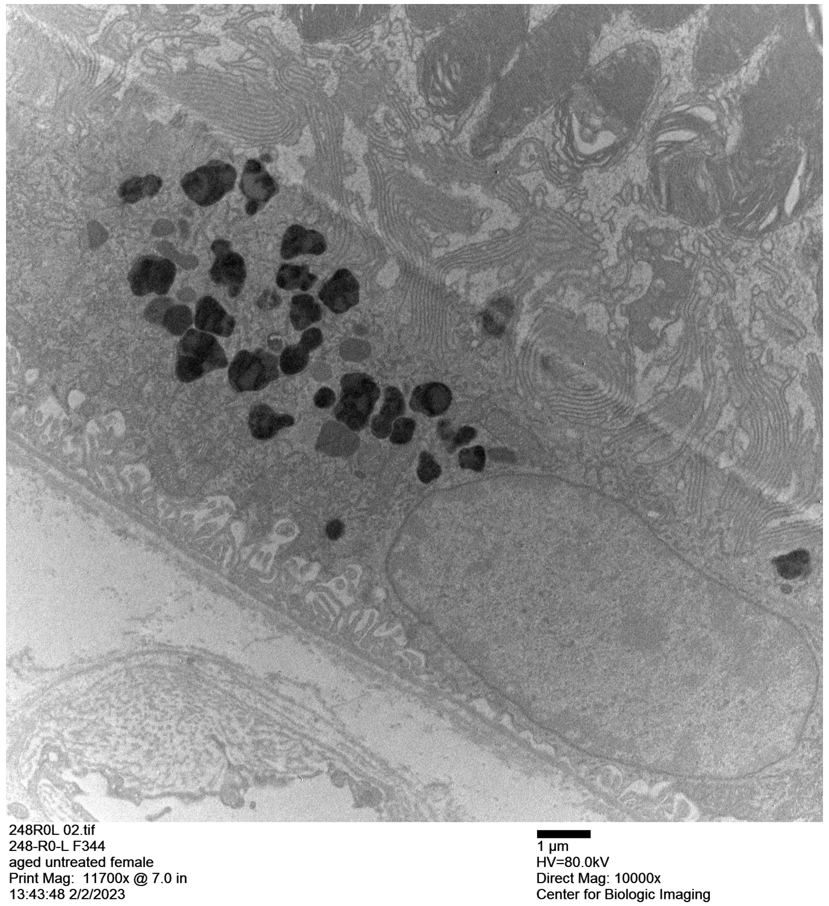

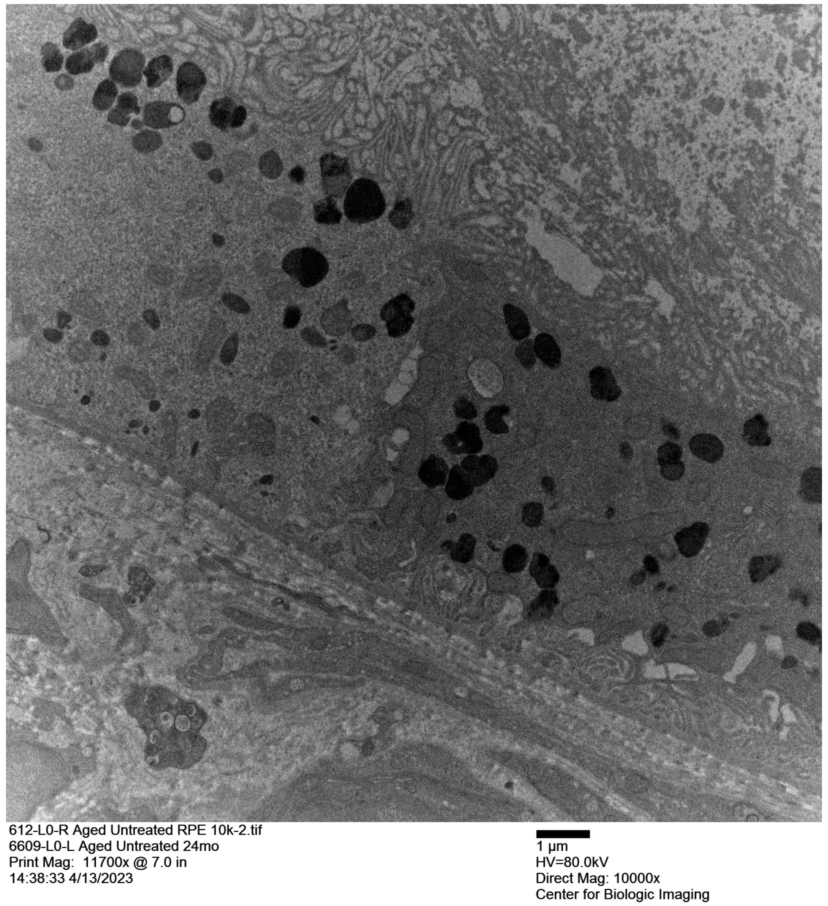

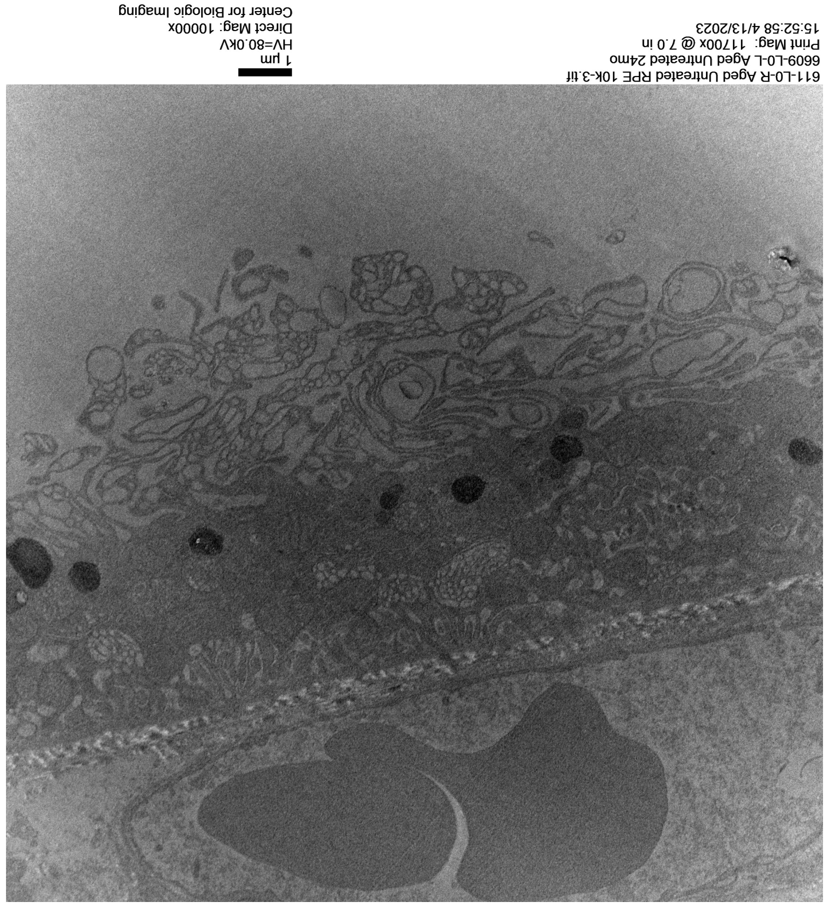

Aged Untreated RPE

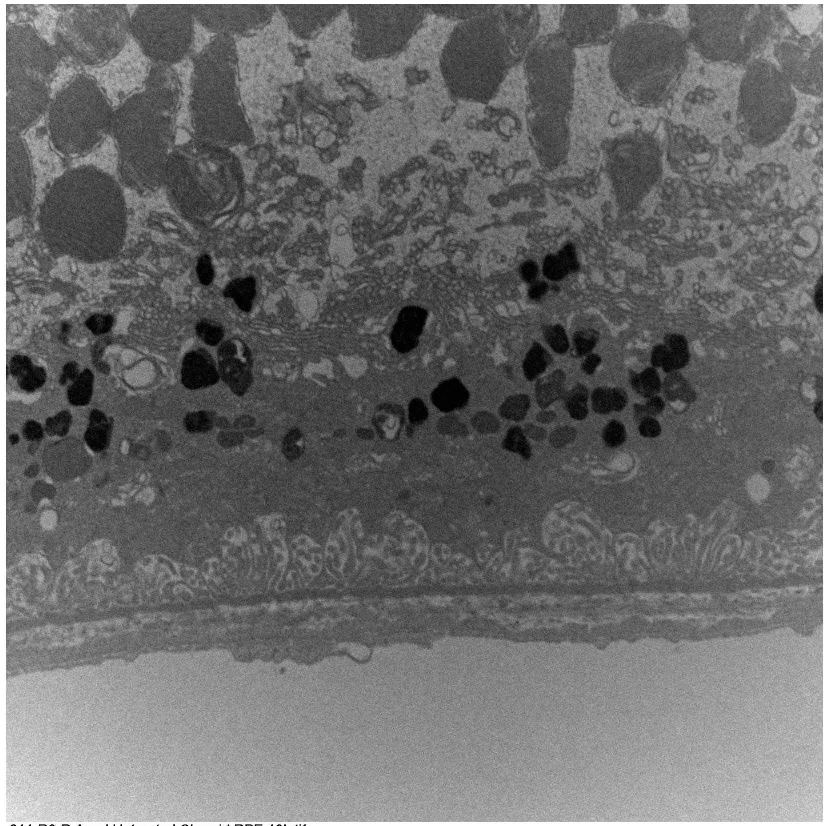

611-R0-R Aged Untreated Choroid RPE 10k.tif
611-R0-R Aged Untreated
Print Mag: 11700x @ 7.0 in
11:39:16 4/27/2023

1 µm
HV=80.0kV
Direct Mag: 10000x
Center for Biologic Imaging

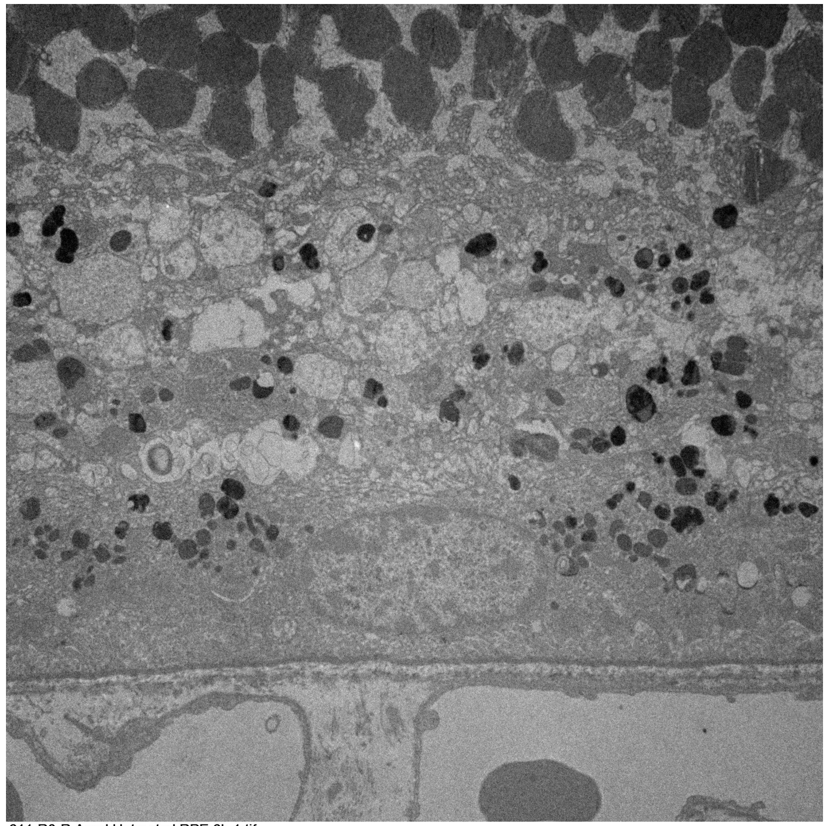

611-R0-R Aged Untreated RPE 6k-1.tif
611-R0-R Aged Untreated
Print Mag: 7020x @ 7.0 in
11:14:07 4/27/2023

2 µm
HV=80.0kV
Direct Mag: 6000x
Center for Biologic Imaging

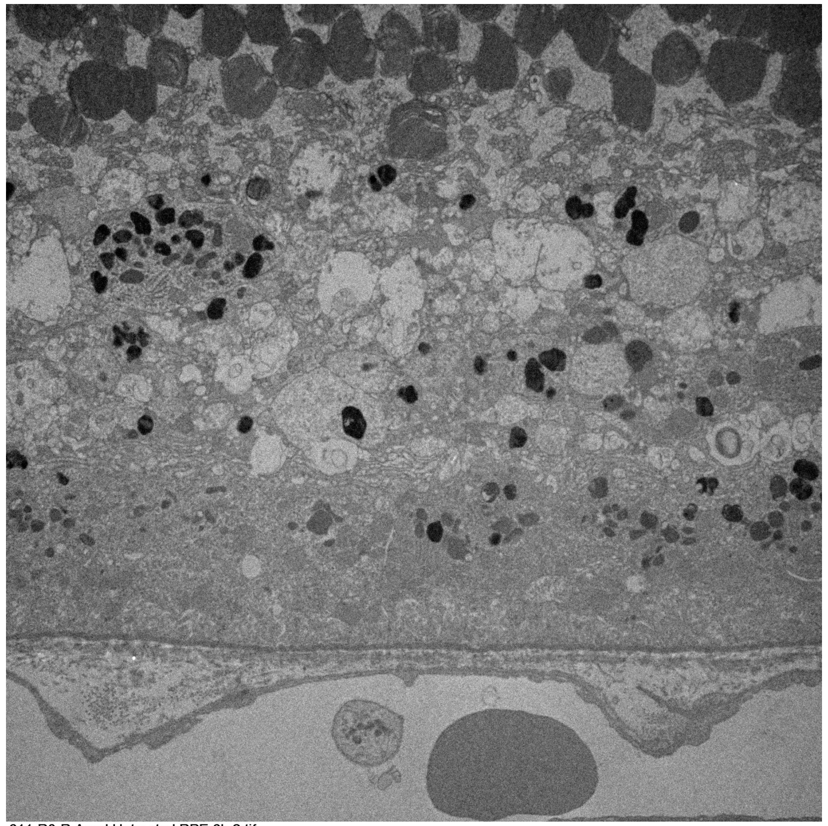

611-R0-R Aged Untreated RPE 6k-2.tif
611-R0-R Aged Untreated
Print Mag: 7020x @ 7.0 in
11:14:44 4/27/2023

2 µm
HV=80.0kV
Direct Mag: 6000x
Center for Biologic Imaging

Aged Untreated RPE

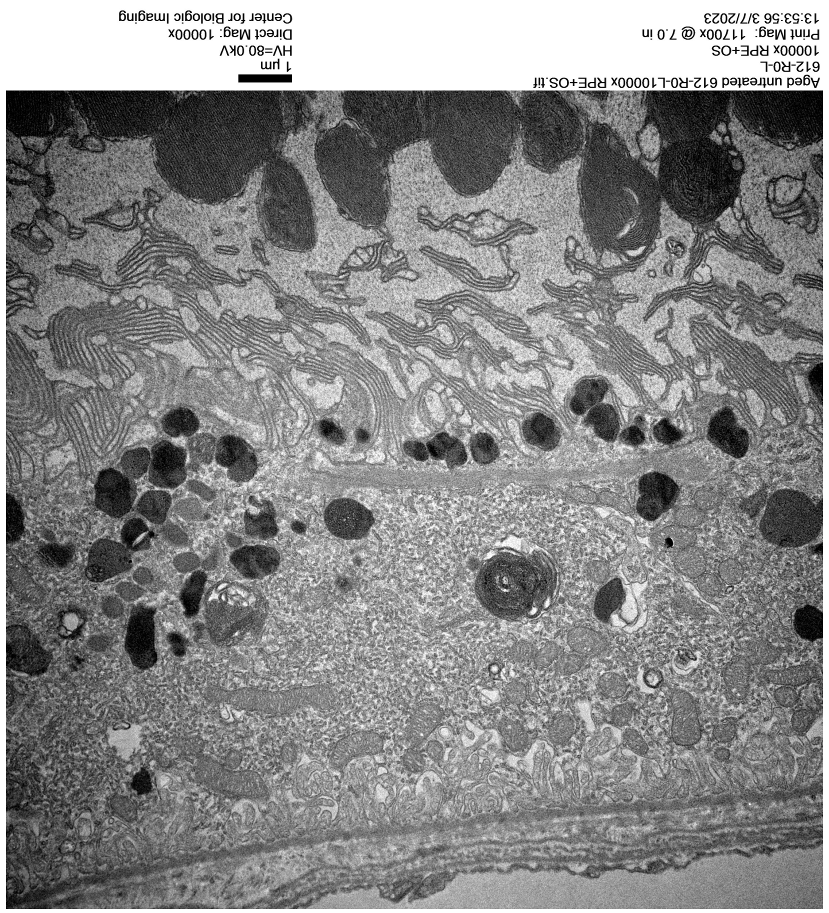

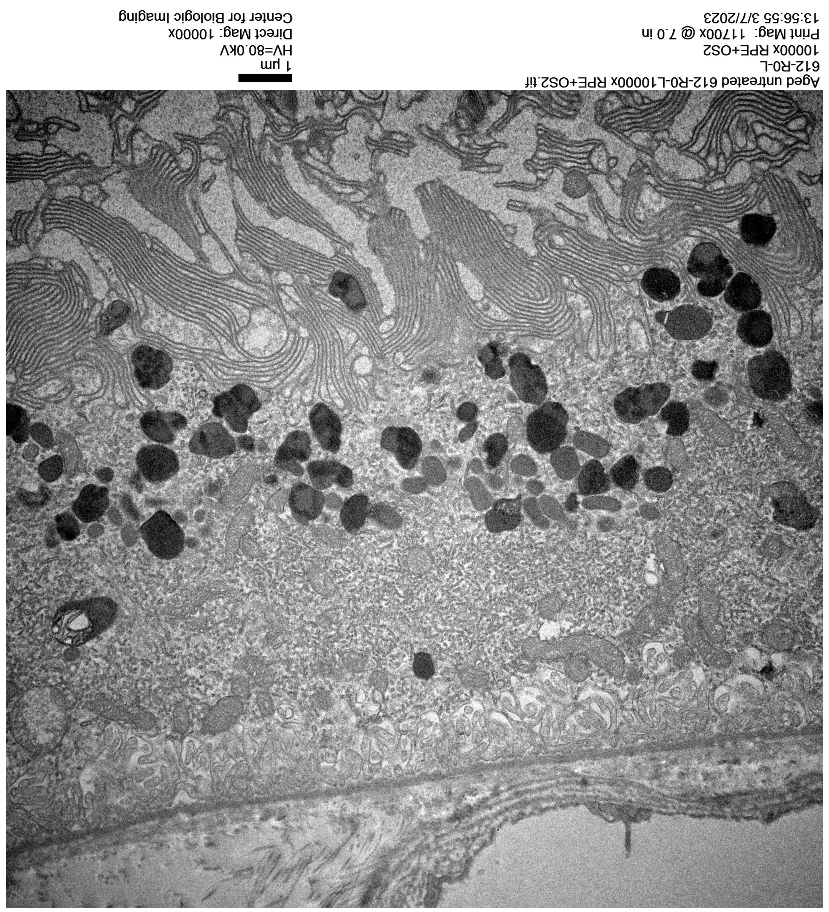

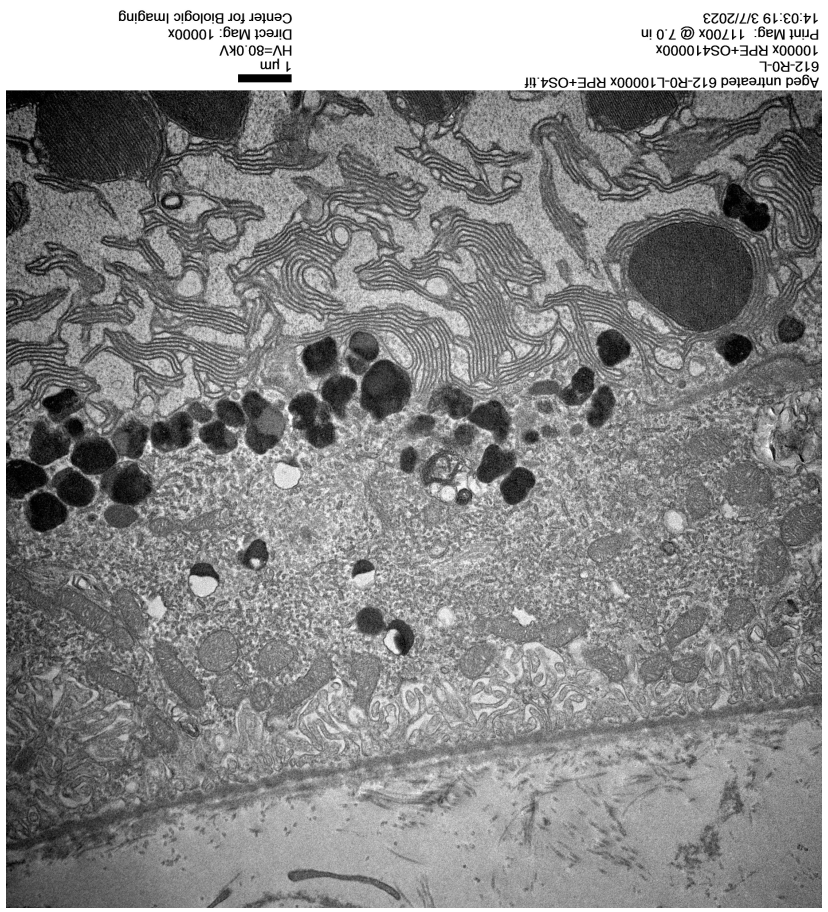

Aged Untreated RPE/OS

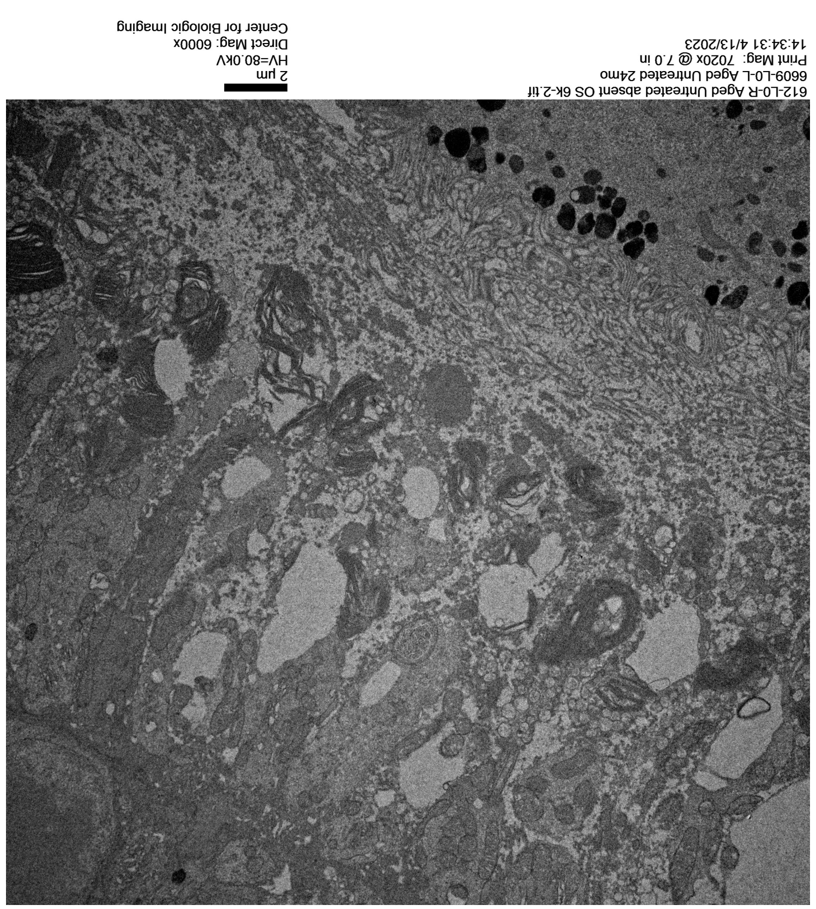

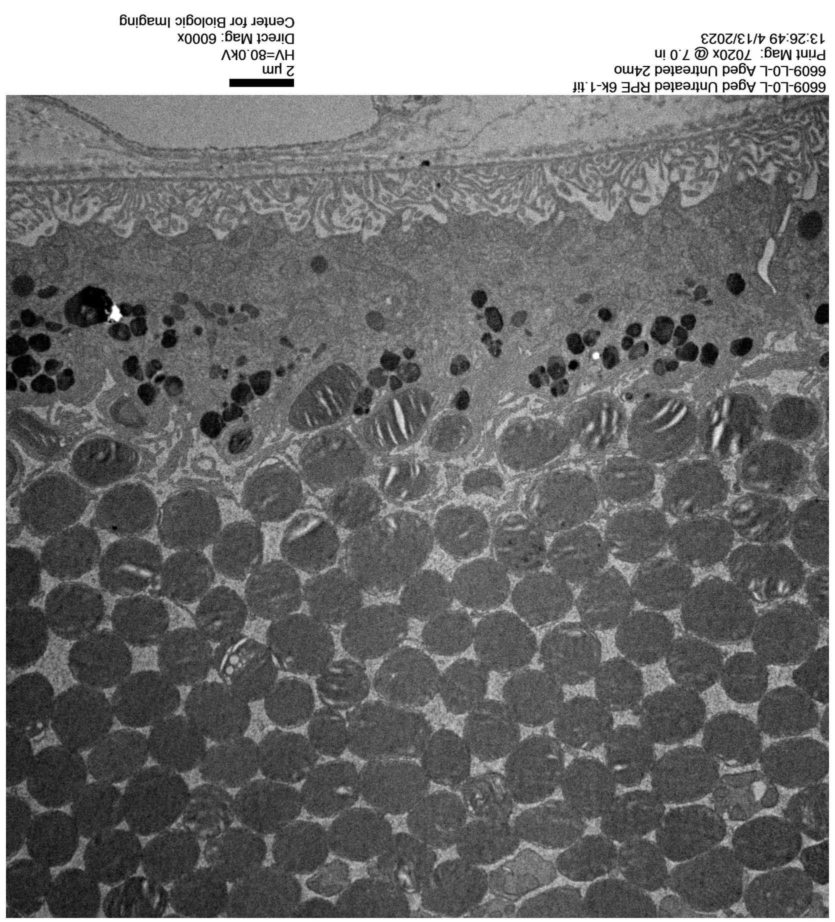

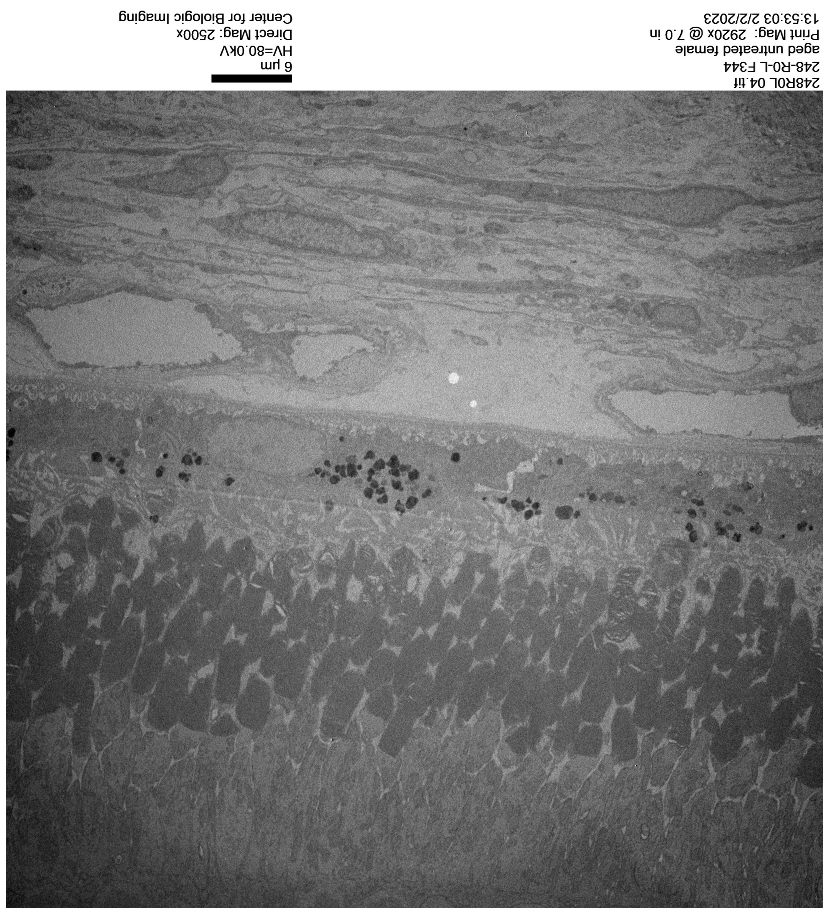

Aged Untreated RPE/OS

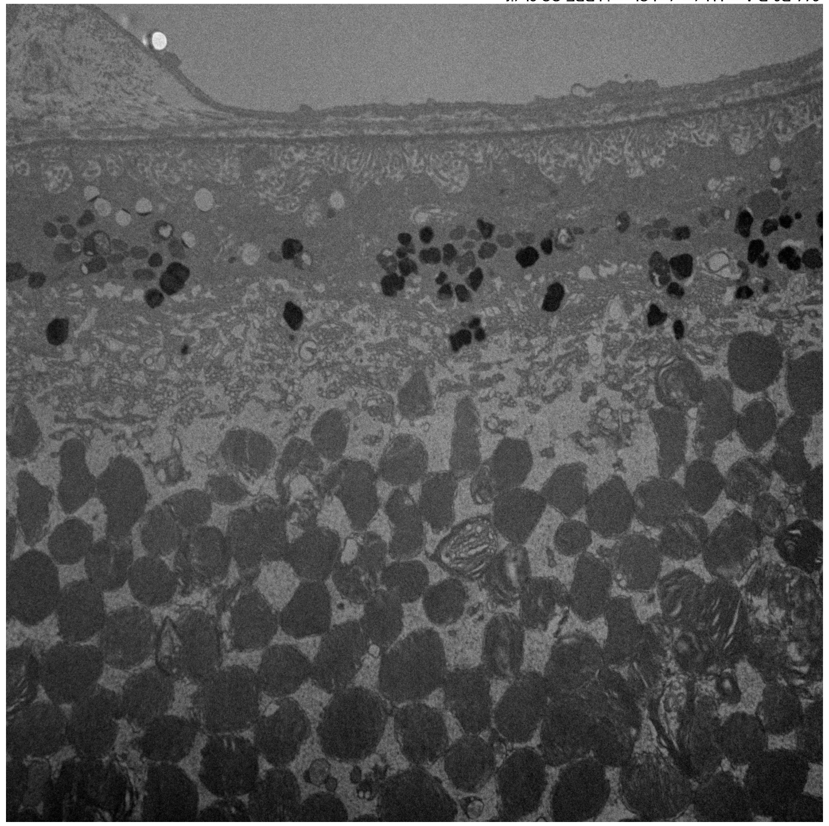

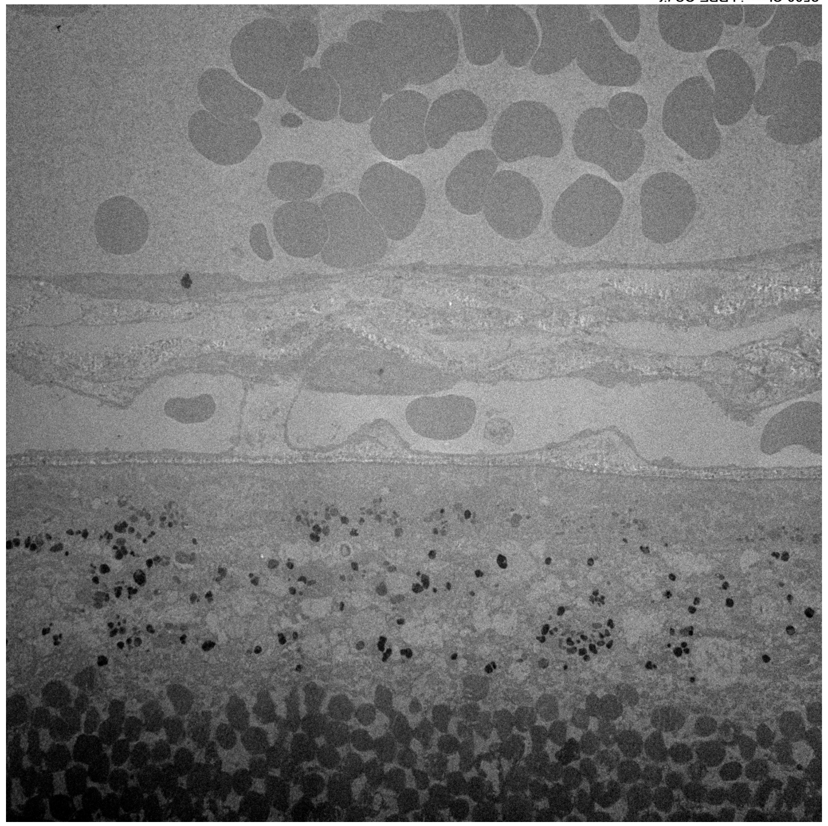

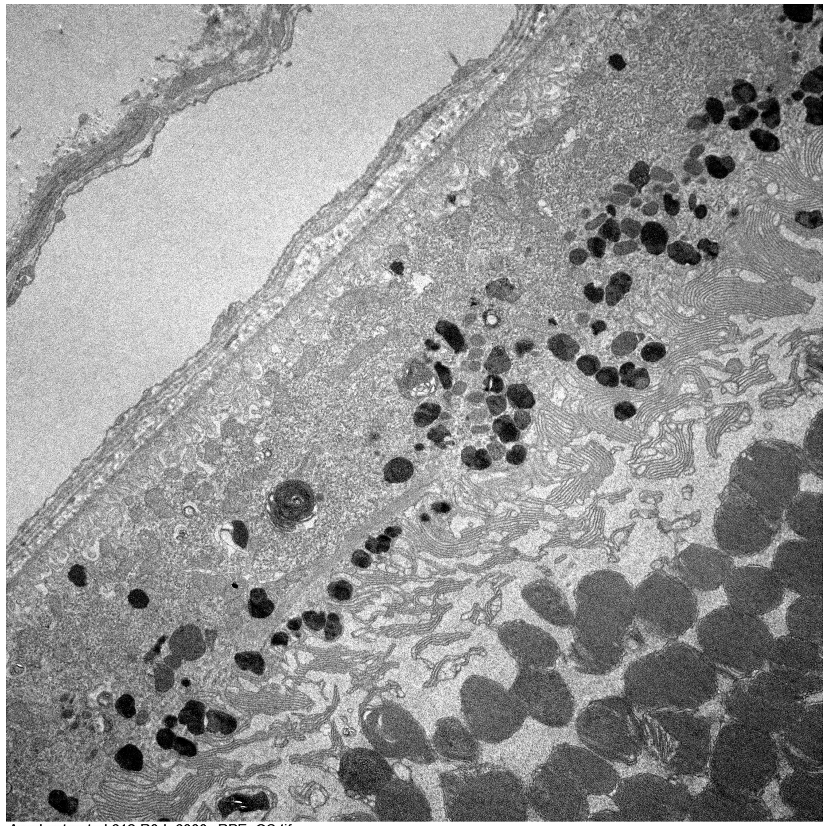

Aged Untreated RPE/OS

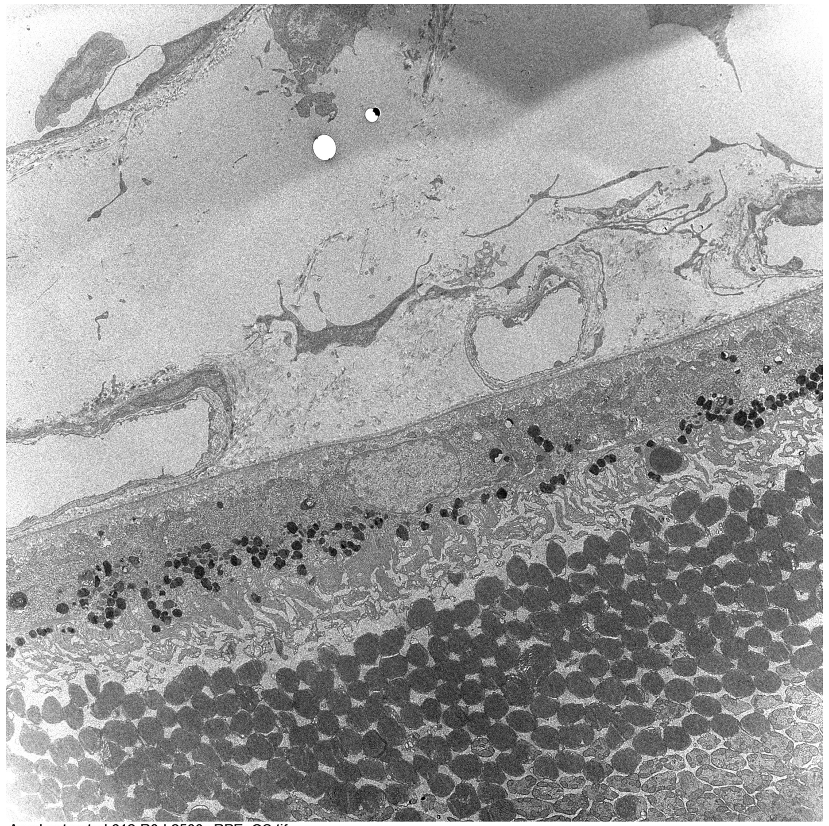

Aged untreated 612-R0-L2500x RPE+OS.tif
612-R0-L
10000x RPE+OS52500 RPE+OS10000x
Print Mag: 2920x @ 7.0 in
14:13:37 3/7/2023

6 µm
HV=80.0kV
Direct Mag: 2500x
Center for Biologic Imaging

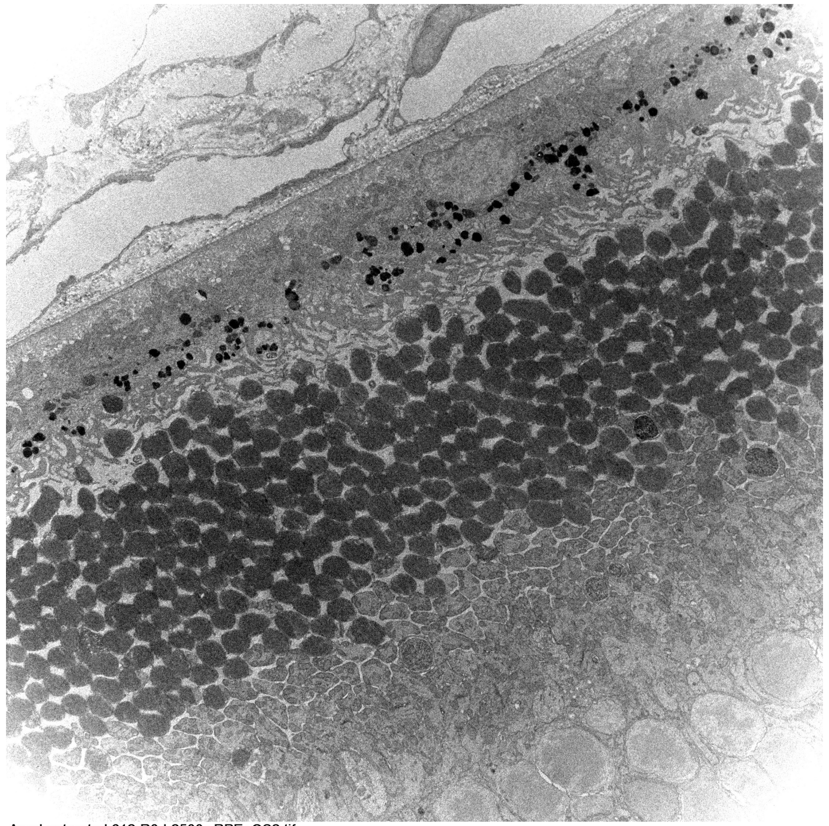

Aged untreated 612-R0-L2500x RPE+OS2.tif
612-R0-L
10000x RPE+OS52500 RPE+OS210000x
Print Mag: 2920x @ 7.0 in
14:24:57 3/7/2023

6 µm
HV=80.0kV
Direct Mag: 2500x
Center for Biologic Imaging

Aged Untreated OS

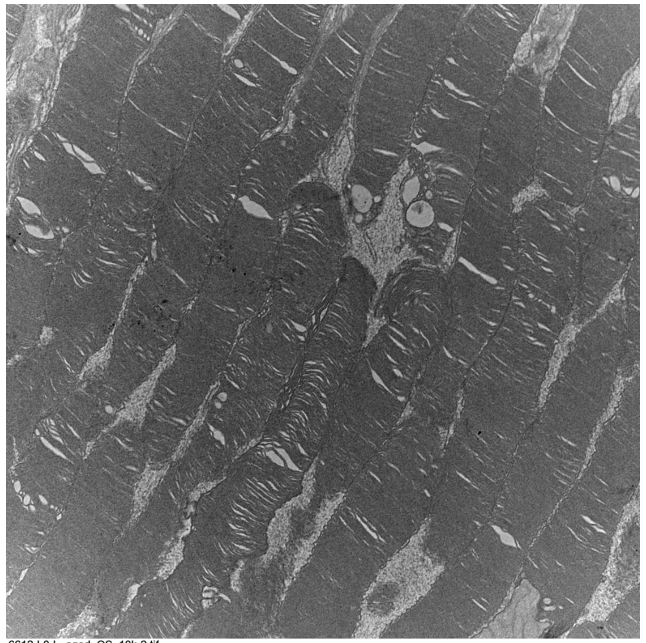

6612-L0-L_aged_OS_10k-2.tif
6612-L0-L aged untreated 24 mo
Print Mag: 11700x @ 7.0 in
11:09:15 2/17/2023

1 µm
HV=80.0kV
Direct Mag: 10000x
Center for Biologic Imaging

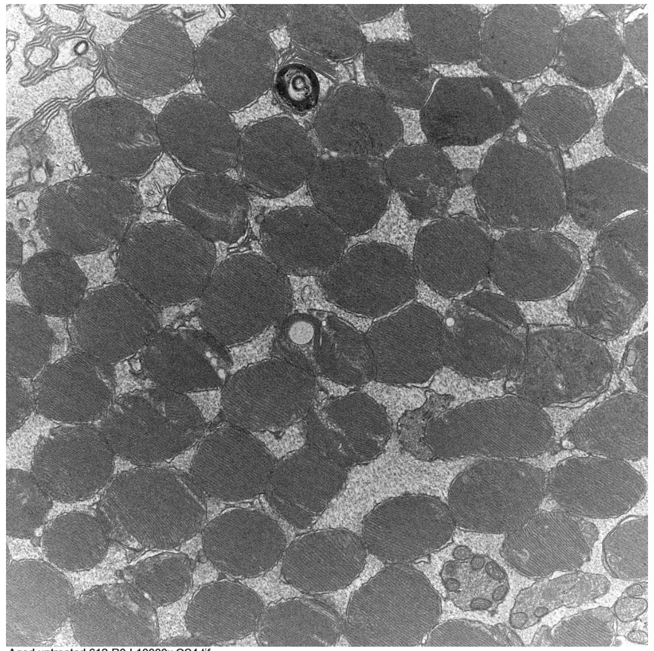

Aged untreated 612-R0-L10000x OS4.tif
612-R0-L
10000x OS42500 RPE+OS210000x
Print Mag: 11700x @ 7.0 in
14:41:18 3/7/2023

1 µm
HV=80.0kV
Direct Mag: 10000x
Center for Biologic Imaging

6609-L0-L_Aged Untreated OS 10k-2.tif
6609-L0-L Aged Untreated 24mo
Print Mag: 11700x @ 7.0 in
13:39:42 4/13/2023

1 µm
HV=80.0kV
Direct Mag: 10000x
Center for Biologic Imaging

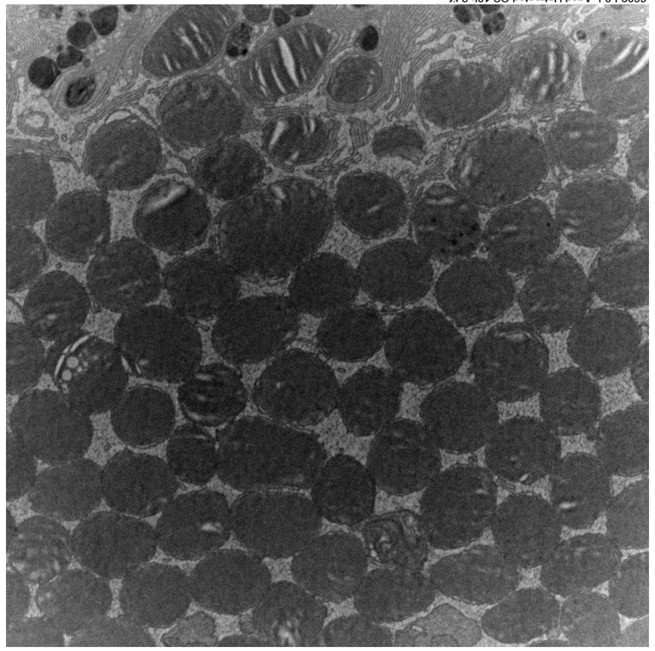

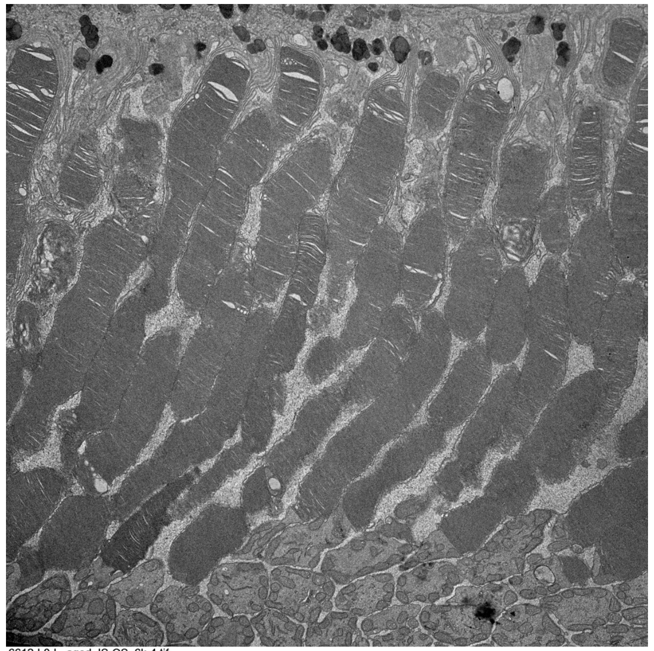

6612-L0-L_aged_IS-OS_6k-4.tif
6612-L0-L aged untreated 24 mo
Print Mag: 7020x @ 7.0 in
09:52:05 2/17/2023

2 µm
HV=80.0kV
Direct Mag: 6000x
Center for Biologic Imaging

Aged untreated OS

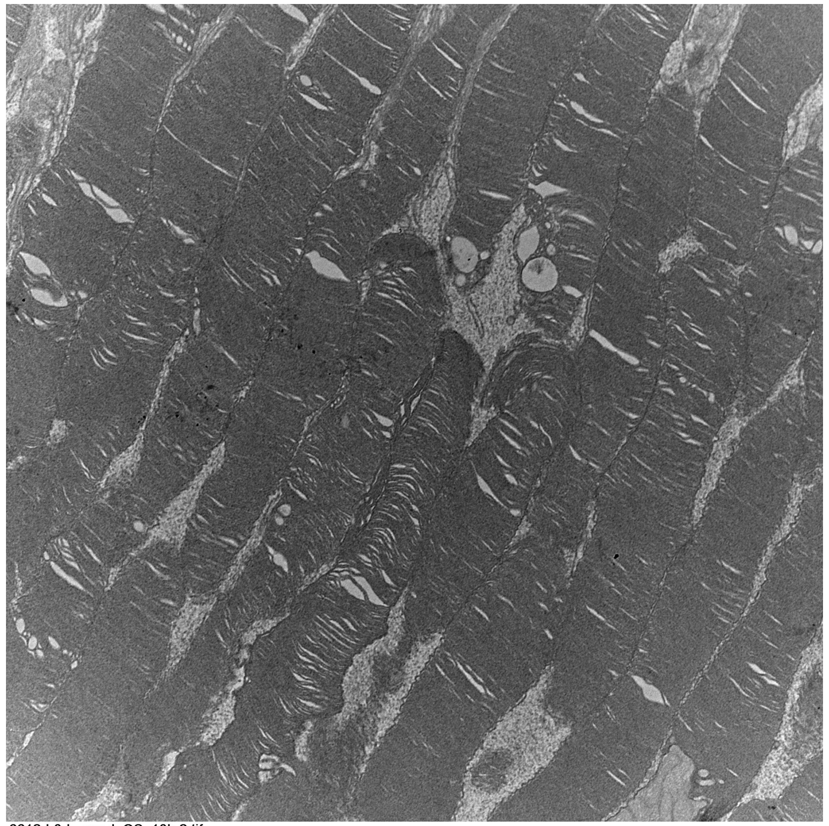

6612-L0-L_aged_OS_10k-2.tif
6612-L0-L aged untreated 24 mo
Print Mag: 11700x @ 7.0 in
11:09:15 2/17/2023

1 µm
HV=80.0kV
Direct Mag: 10000x
Center for Biologic Imaging

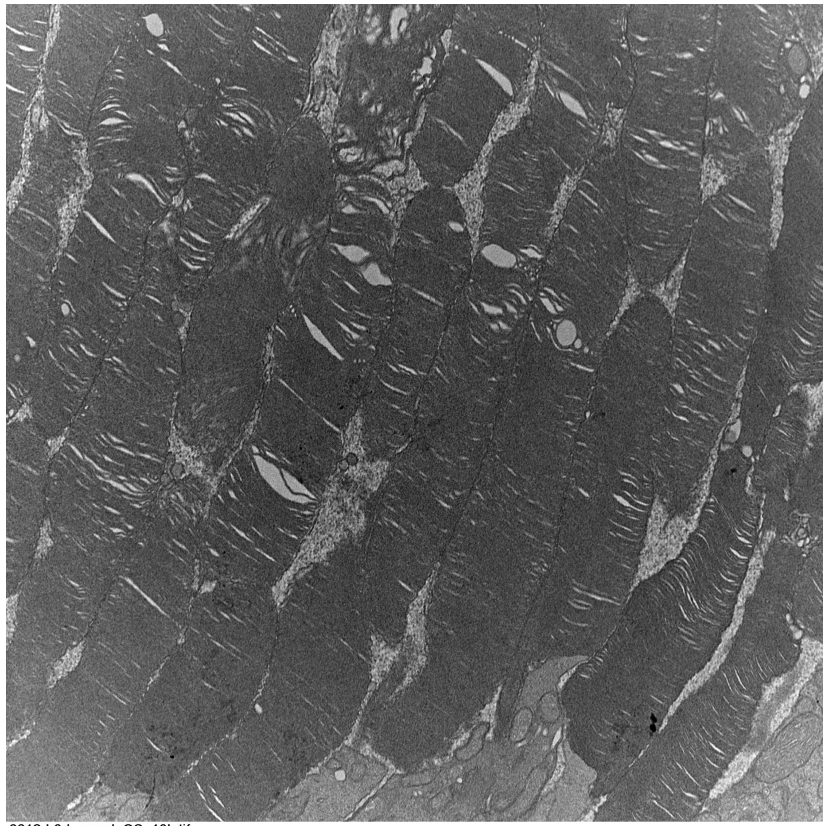

6612-L0-L_aged_OS_10k.tif
6612-L0-L aged untreated 24 mo
Print Mag: 11700x @ 7.0 in
11:08:28 2/17/2023

1 µm
HV=80.0kV
Direct Mag: 10000x
Center for Biologic Imaging

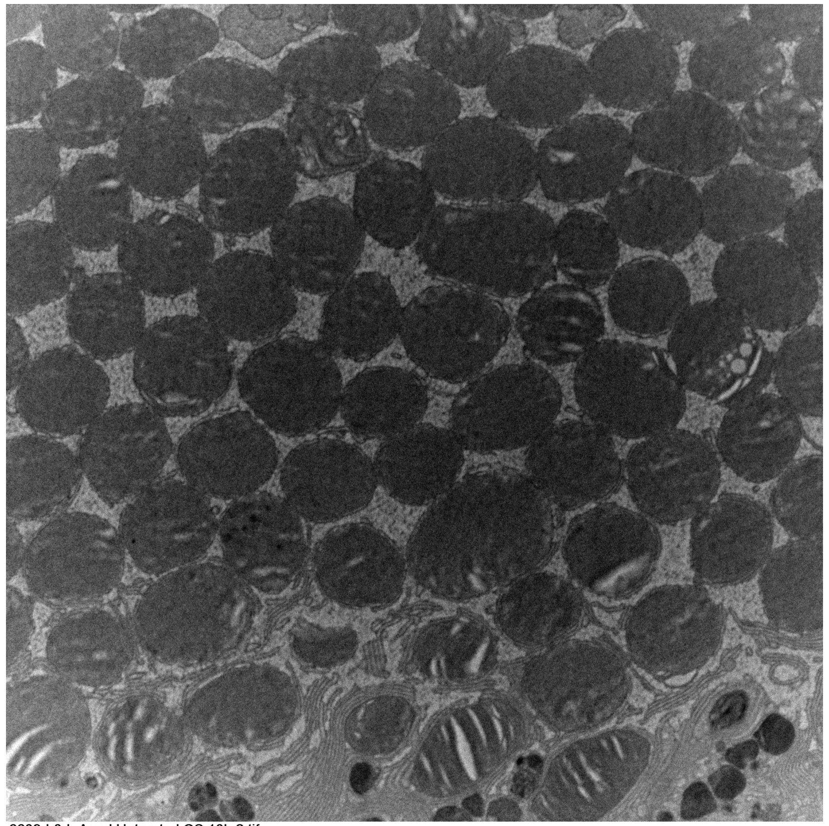

6609-L0-L Aged Untreated OS 10k-2.tif
6609-L0-L Aged Untreated 24mo
Print Mag: 11700x @ 7.0 in
13:39:42 4/13/2023

1 µm
HV=80.0kV
Direct Mag: 10000x
Center for Biologic Imaging

Aged Untreated OS

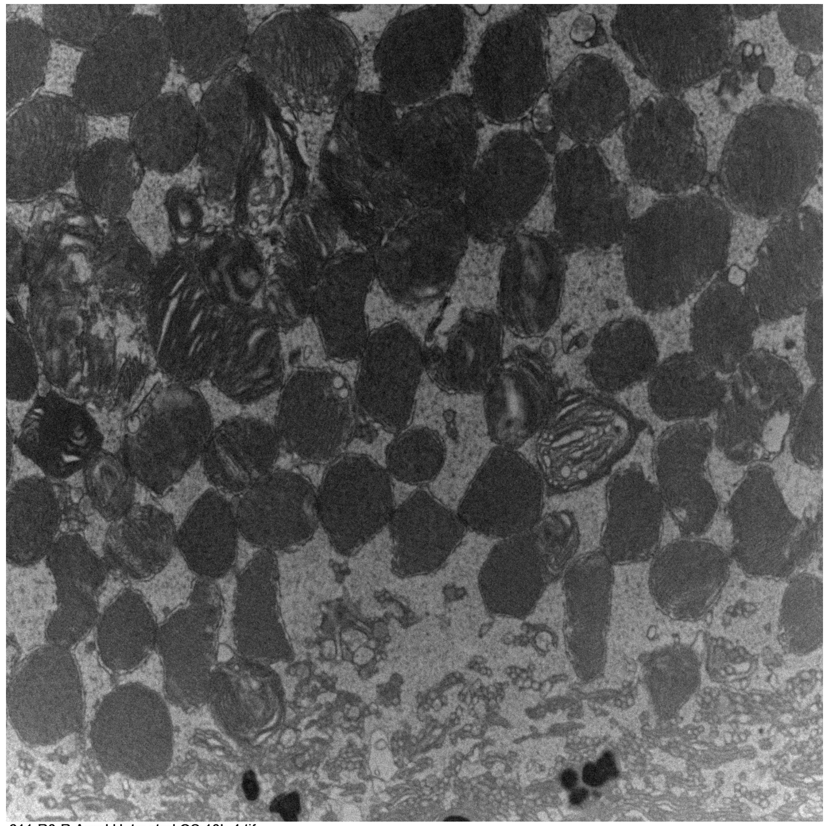

611-R0-R Aged Untreated OS 10k-4.tif
611-R0-R Aged Untreated
Print Mag: 11700x @ 7.0 in
11:41:14 4/27/2023

1 µm
HV=80.0kV
Direct Mag: 10000x
Center for Biologic Imaging

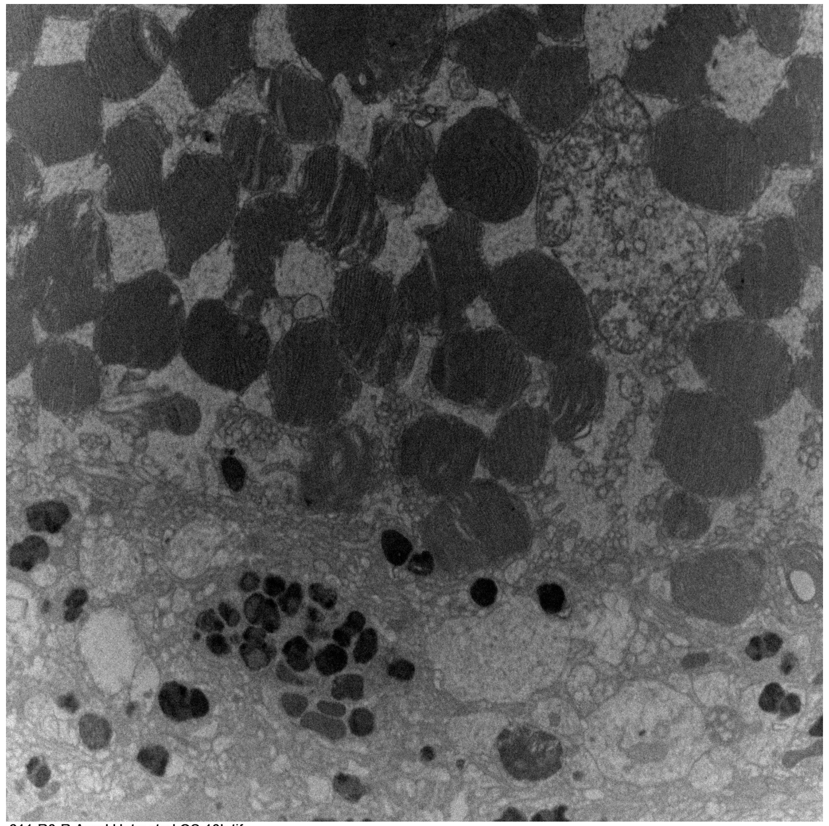

611-R0-R Aged Untreated OS 10k.tif
611-R0-R Aged Untreated
Print Mag: 11700x @ 7.0 in
11:24:45 4/27/2023

1 µm
HV=80.0kV
Direct Mag: 10000x
Center for Biologic Imaging

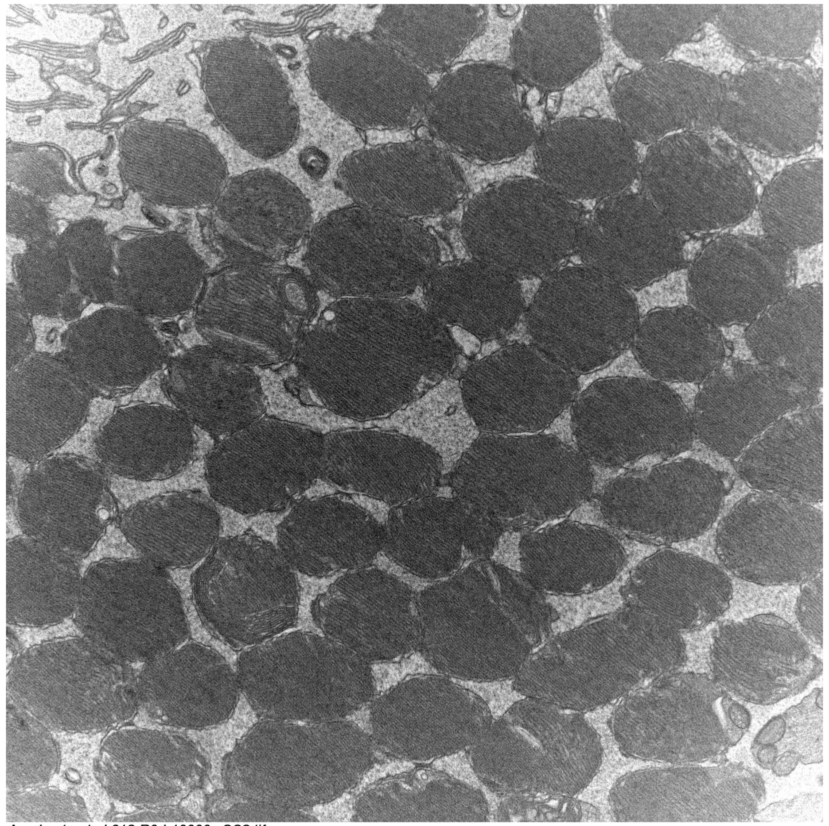

Aged untreated 612-R0-L10000x OS2.tif
612-R0-L
10000x OS22500 RPE+OS210000x
Print Mag: 11700x @ 7.0 in
14:30:07 3/7/2023

1 µm
HV=80.0kV
Direct Mag: 10000x
Center for Biologic Imaging

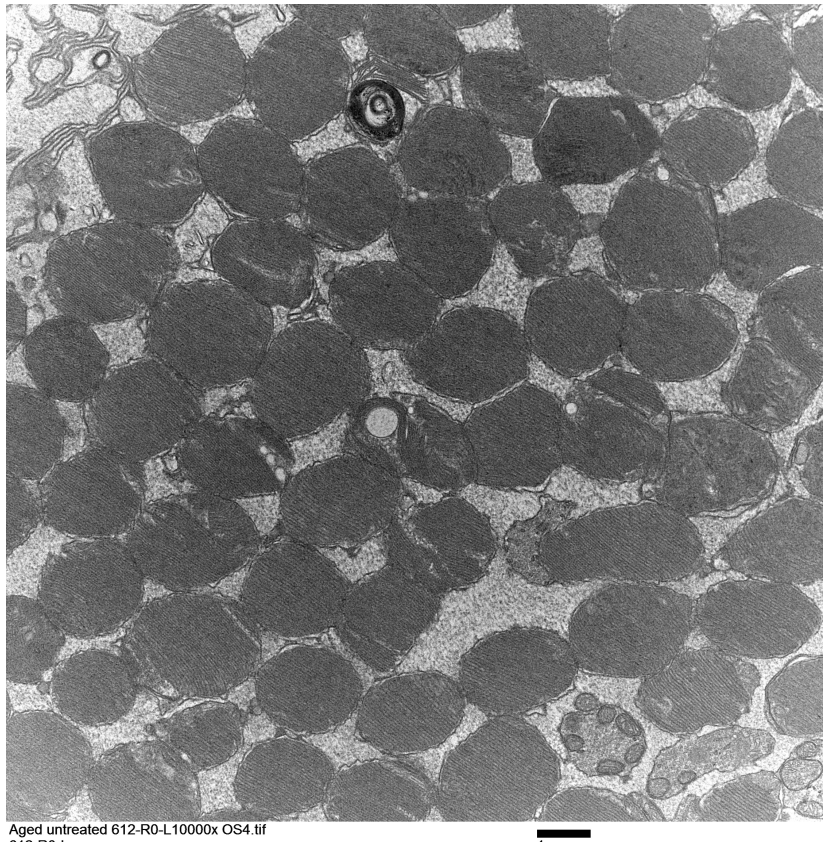

Aged untreated 612-R0-L10000x OS4.tif
612-R0-L
10000x OS42500 RPE+OS210000x
Print Mag: 11700x @ 7.0 in
14:41:18 3/7/2023

1 µm
HV=80.0kV
Direct Mag: 10000x
Center for Biologic Imaging

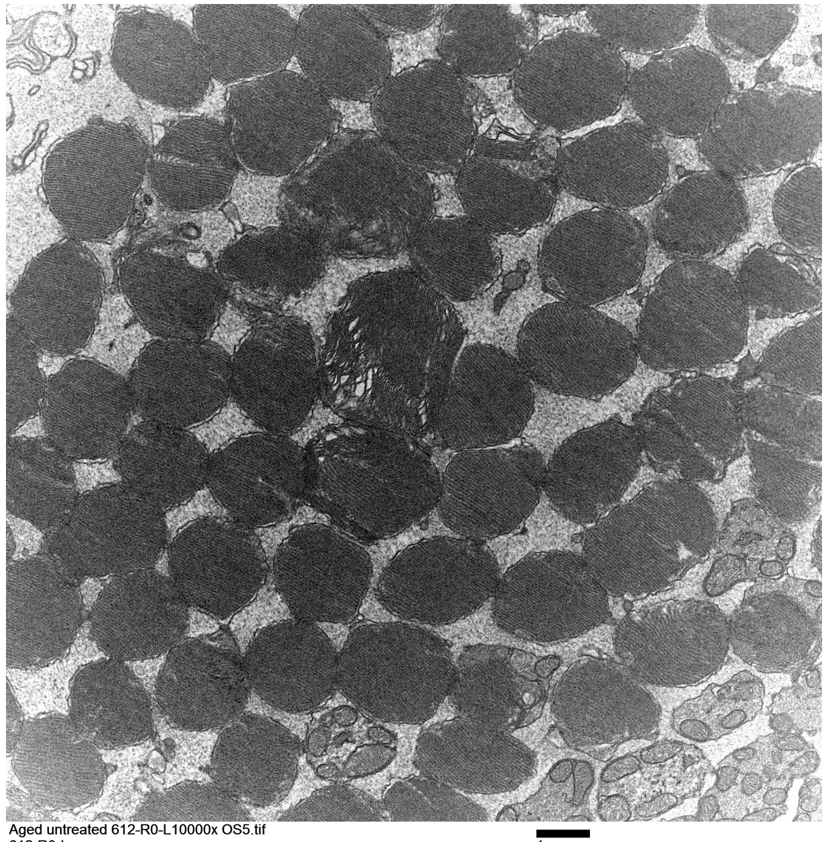

Aged untreated 612-R0-L10000x OS5.tif
612-R0-L
10000x OS52500 RPE+OS210000x
Print Mag: 11700x @ 7.0 in
14:42:45 3/7/2023

1 µm
HV=80.0kV
Direct Mag: 10000x
Center for Biologic Imaging

Aged Untreated IS/OS

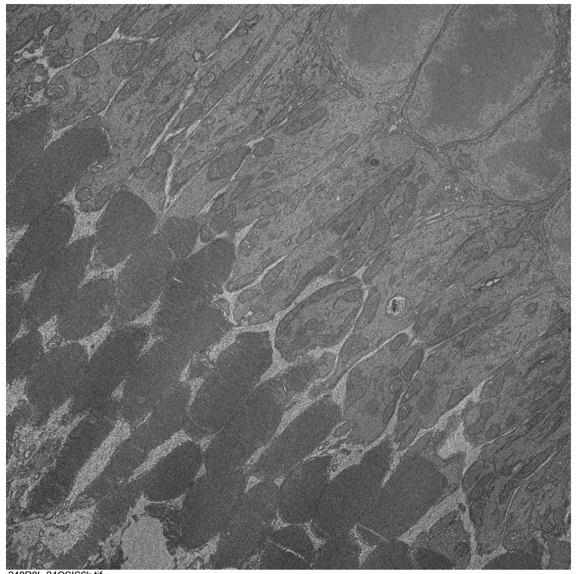

248R0L-24OSIS6k.tif
248-R0-L F344
aged untreated female
Print Mag: 7020x @ 7.0 in
16:01:09 2/2/2023

2 µm
HV=80.0kV
Direct Mag: 6000x
Center for Biologic Imaging

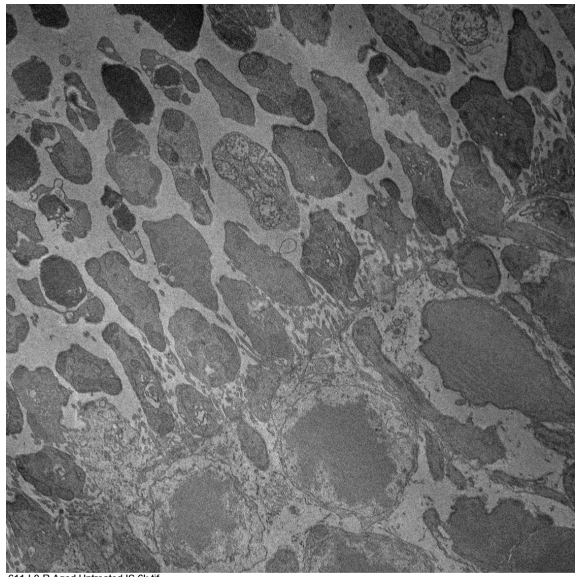

611-L0-R Aged Untreated IS 6k.tif
6609-L0-L Aged Untreated 24mo
Print Mag: 7020x @ 7.0 in
15:23:39 4/13/2023

2 µm
HV=80.0kV
Direct Mag: 6000x
Center for Biologic Imaging

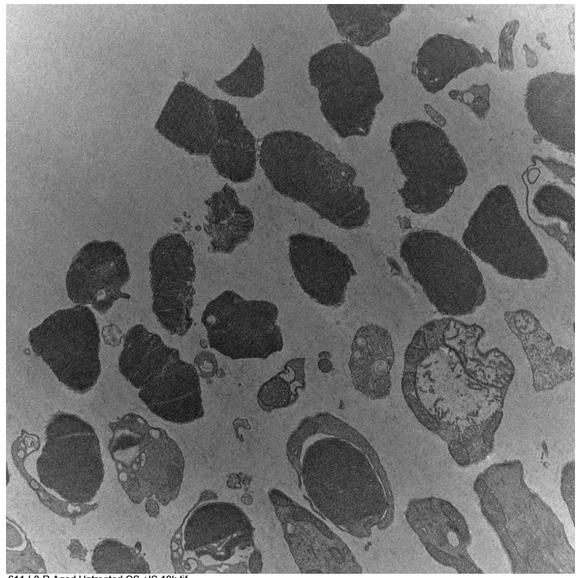

611-L0-R Aged Untreated OS +IS 10k.tif
6609-L0-L Aged Untreated 24mo
Print Mag: 11700x @ 7.0 in
15:31:09 4/13/2023

1 µm
HV=80.0kV
Direct Mag: 10000x
Center for Biologic Imaging

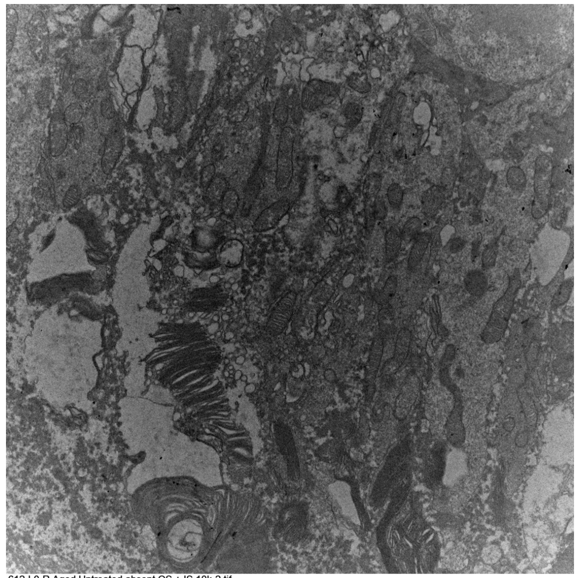

612-L0-R Aged Untreated absent OS + IS 10k-2.tif
6609-L0-L Aged Untreated 24mo
Print Mag: 11700x @ 7.0 in
15:08:49 4/13/2023

1 µm
HV=80.0kV
Direct Mag: 10000x
Center for Biologic Imaging

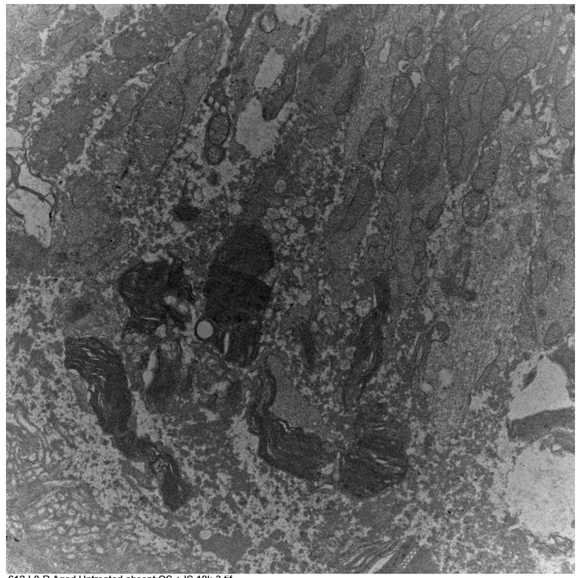

612-L0-R Aged Untreated absent OS + IS 10k-3.tif
6609-L0-L Aged Untreated 24mo
Print Mag: 11700x @ 7.0 in
15:09:30 4/13/2023

1 µm
HV=80.0kV
Direct Mag: 10000x
Center for Biologic Imaging

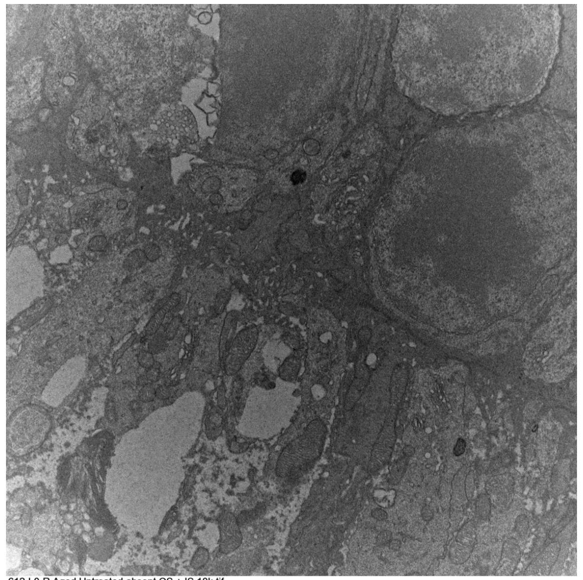

612-L0-R Aged Untreated absent OS + IS 10k.tif
6609-L0-L Aged Untreated 24mo
Print Mag: 11700x @ 7.0 in
15:05:19 4/13/2023

1 µm
HV=80.0kV
Direct Mag: 10000x
Center for Biologic Imaging

Aged Untreated IS

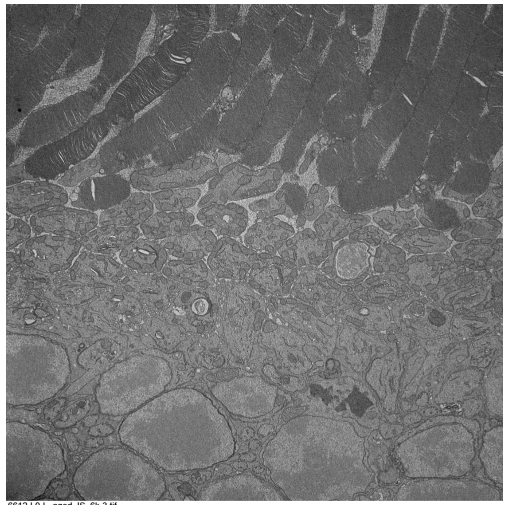

6612-L0-L_aged_IS_6k-3.tif
6612-L0-L aged untreated 24 mo
Print Mag: 7020x @ 7.0 in
10:03:11 2/17/2023
2 µm
HV=80.0kV
Direct Mag: 6000x
Center for Biologic Imaging

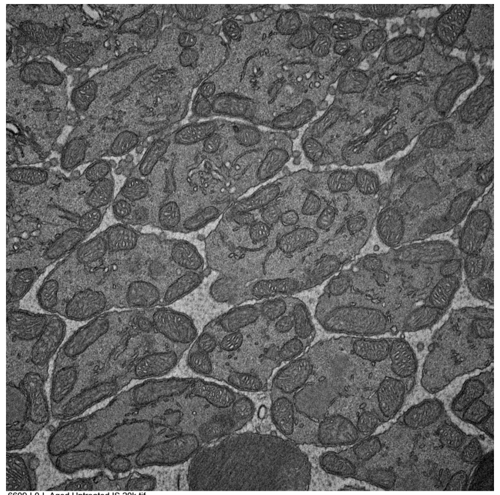

6609-L0-L Aged Untreated IS 20k.tif
6609-L0-L Aged Untreated 24mo
Print Mag: 23400x @ 7.0 in
14:19:32 4/13/2023
800 nm
HV=80.0kV
Direct Mag: 20000x
Center for Biologic Imaging

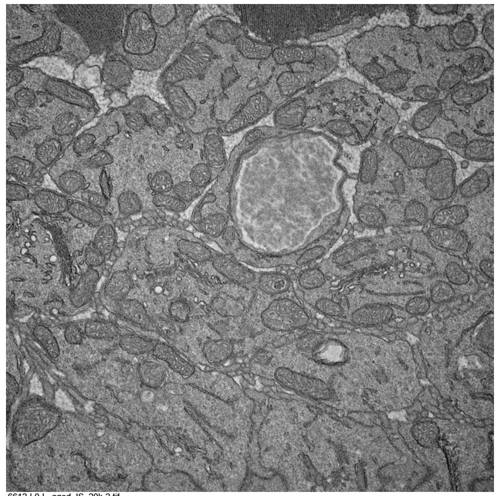

6612-L0-L_aged_IS_20k-2.tif
6612-L0-L aged untreated 24 mo
Print Mag: 23400x @ 7.0 in
11:42:33 2/17/2023
800 nm
HV=80.0kV
Direct Mag: 20000x
Center for Biologic Imaging

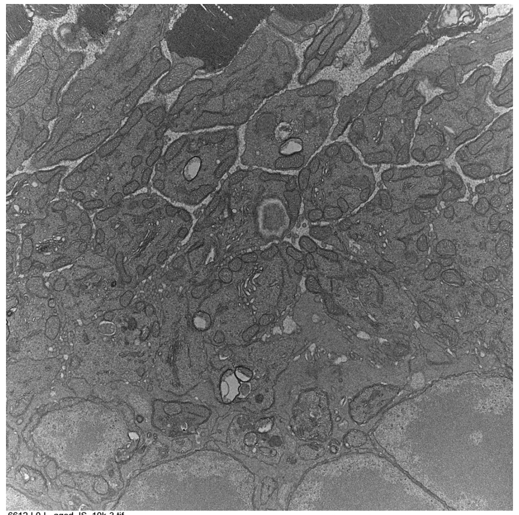

6612-L0-L_aged_IS_10k-3.tif
6612-L0-L aged untreated 24 mo
Print Mag: 11700x @ 7.0 in
11:07:43 2/17/2023
1 µm
HV=80.0kV
Direct Mag: 10000x
Center for Biologic Imaging

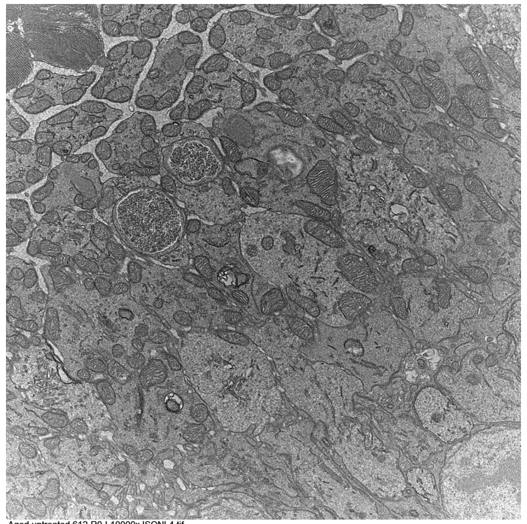

Aged untreated 612-R0-L10000x ISONL4.tif
612-R0-L
10000x ISONL42500 RPE+OS210000x
Print Mag: 11700x @ 7.0 in
15:01:32 3/7/2023
1 µm
HV=80.0kV
Direct Mag: 10000x
Center for Biologic Imaging

6609-L0-L Aged Untreated IS 10k-3.tif
6609-L0-L Aged Untreated 24mo
Print Mag: 11700x @ 7.0 in
13:42:21 4/13/2023
1 µm
HV=80.0kV
Direct Mag: 10000x
Center for Biologic Imaging

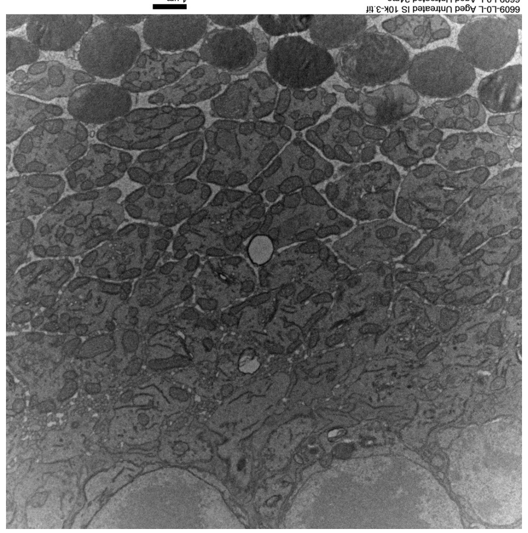

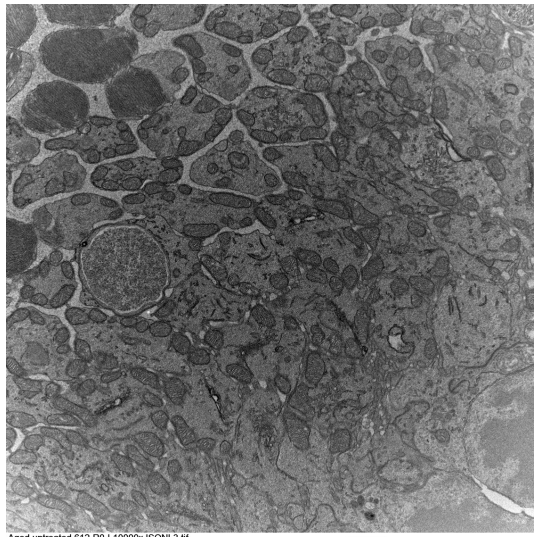

Aged untreated 612-R0-L10000x ISONL3.tif
612-R0-L
10000x ISONL32500 RPE+OS210000x
Print Mag: 11700x @ 7.0 in
14:58:51 3/7/2023
1 µm
HV=80.0kV
Direct Mag: 10000x
Center for Biologic Imaging

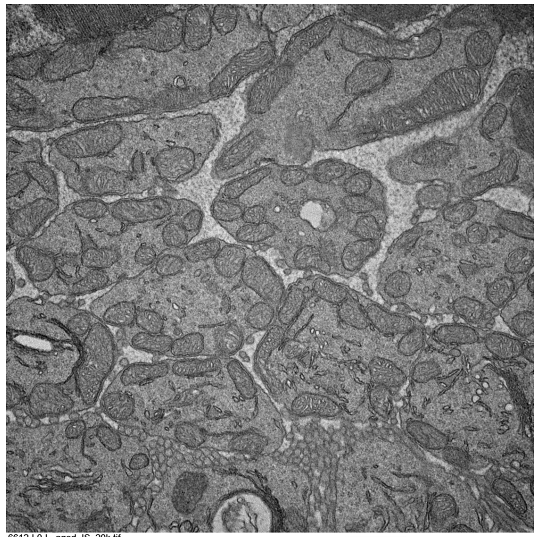

6612-L0-L_aged_IS_20k.tif
6612-L0-L aged untreated 24 mo
Print Mag: 23400x @ 7.0 in
11:41:13 2/17/2023
800 nm
HV=80.0kV
Direct Mag: 20000x
Center for Biologic Imaging

Aged untreated IS

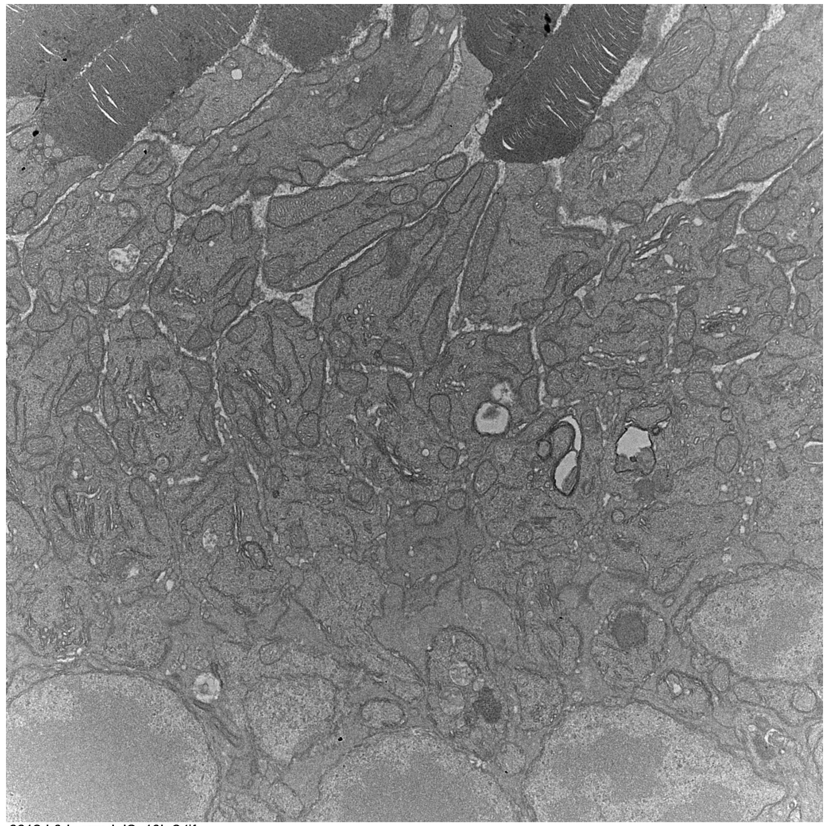

6612-L0-L_aged_IS_10k-2.tif
6612-L0-L aged untreated 24 mo
Print Mag: 11700x @ 7.0 in
11:06:11 2/17/2023

1 μm
HV=80.0kV
Direct Mag: 10000x
Center for Biologic Imaging

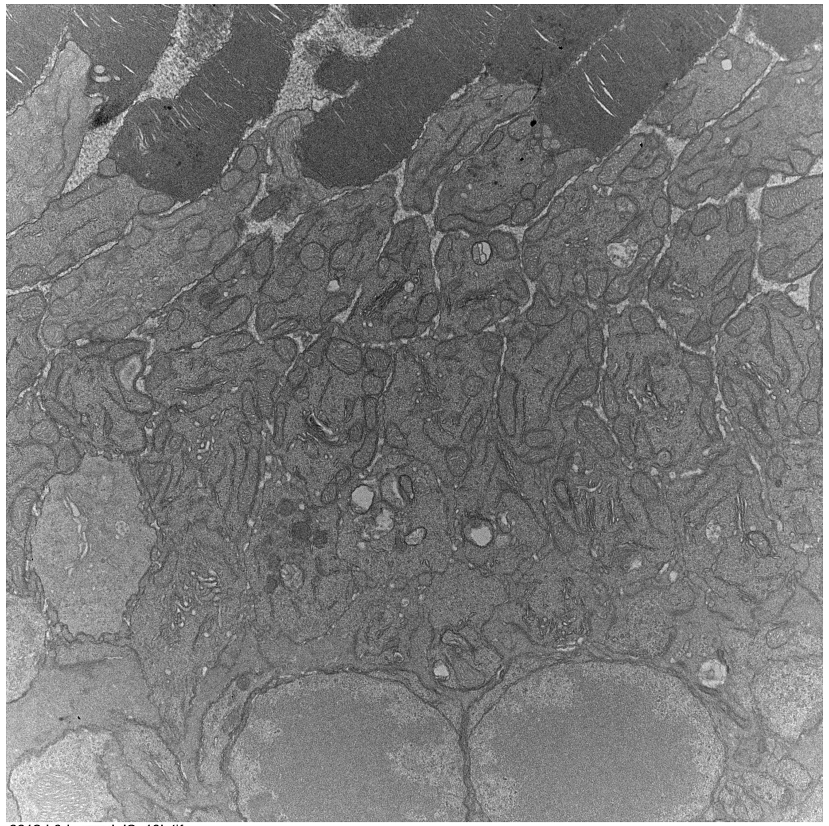

6612-L0-L_aged_IS_10k.tif
6612-L0-L aged untreated 24 mo
Print Mag: 11700x @ 7.0 in
11:00:01 2/17/2023

1 μm
HV=80.0kV
Direct Mag: 10000x
Center for Biologic Imaging

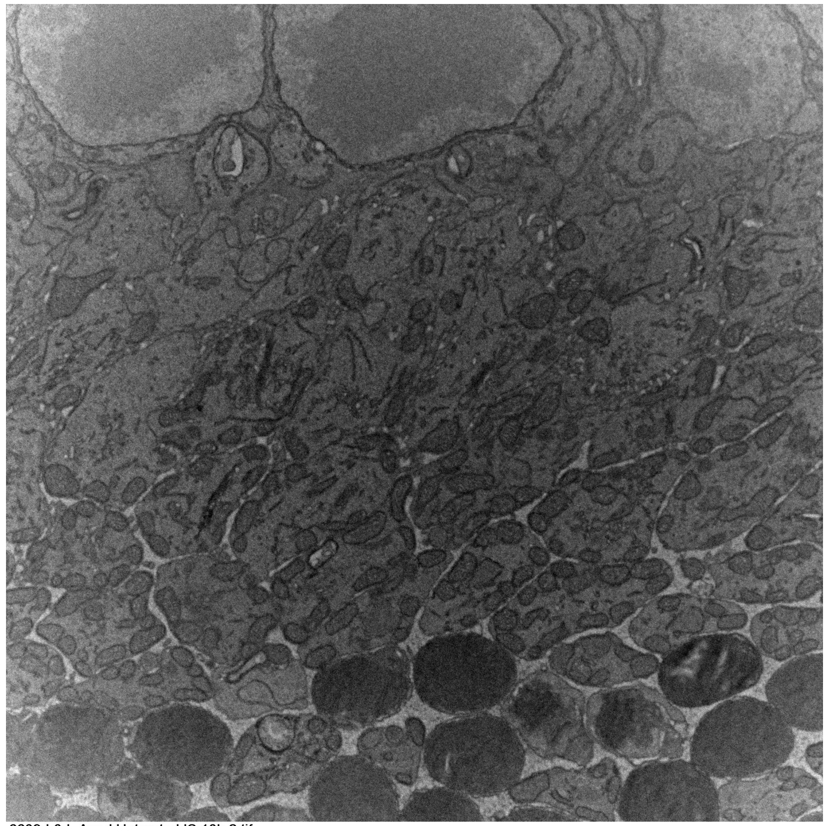

6609-L0-L Aged Untreated IS 10k-2.tif
6609-L0-L Aged Untreated 24mo
Print Mag: 11700x @ 7.0 in
13:41:33 4/13/2023

1 μm
HV=80.0kV
Direct Mag: 10000x
Center for Biologic Imaging

Aged Untreated IS

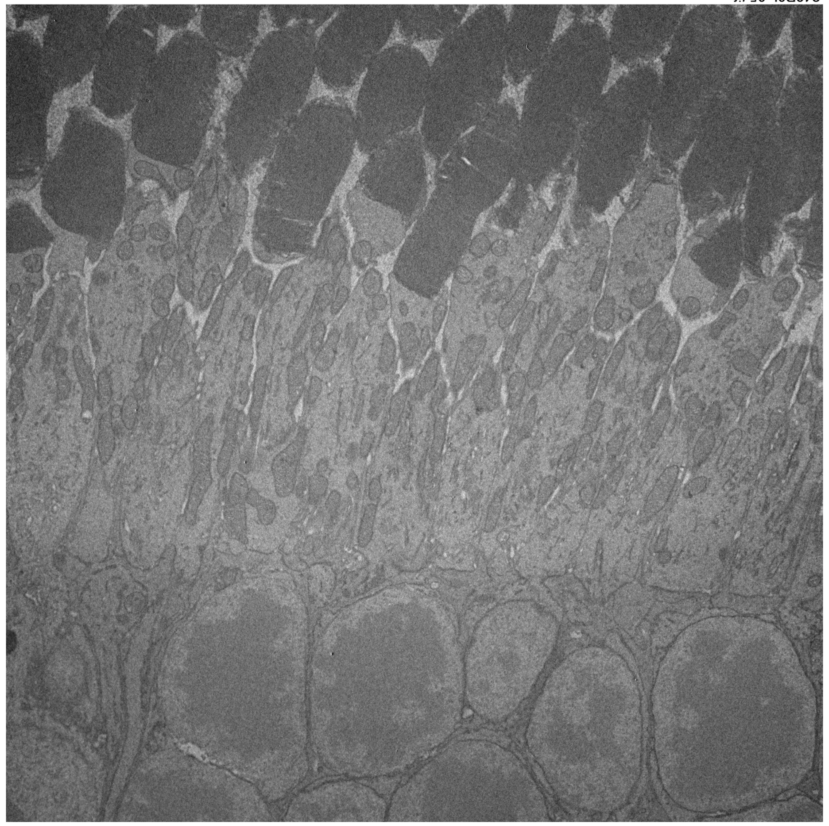

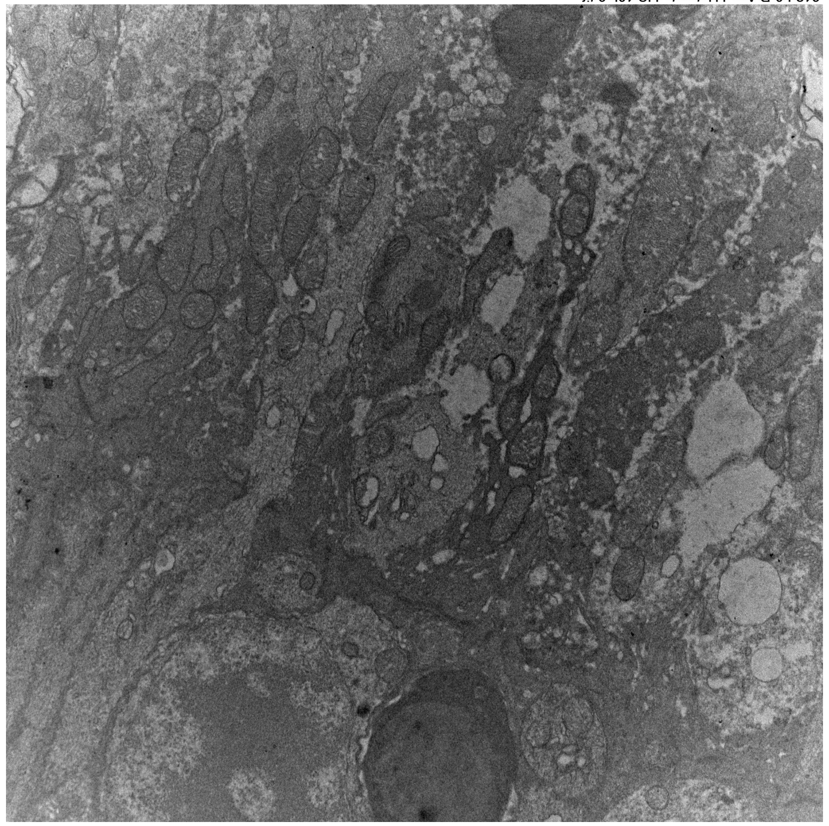

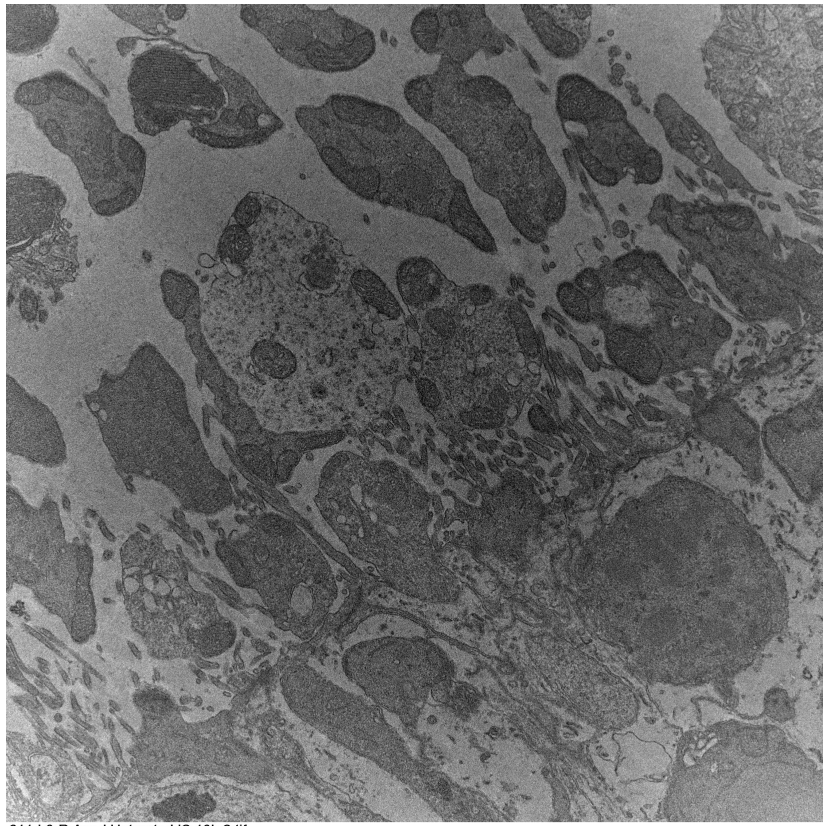

Aged Untreated IS

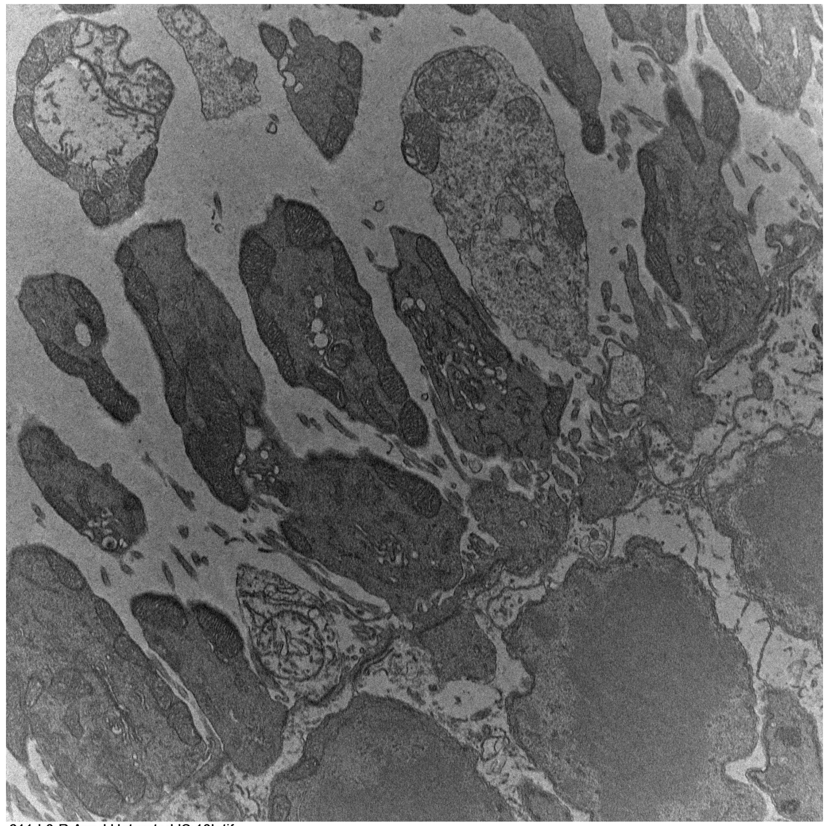

611-L0-R Aged Untreated IS 10k.tif
6609-L0-L Aged Untreated 24mo
Print Mag: 11700x @ 7.0 in
15:31:56 4/13/2023

1 μm
HV=80.0kV
Direct Mag: 10000x
Center for Biologic Imaging

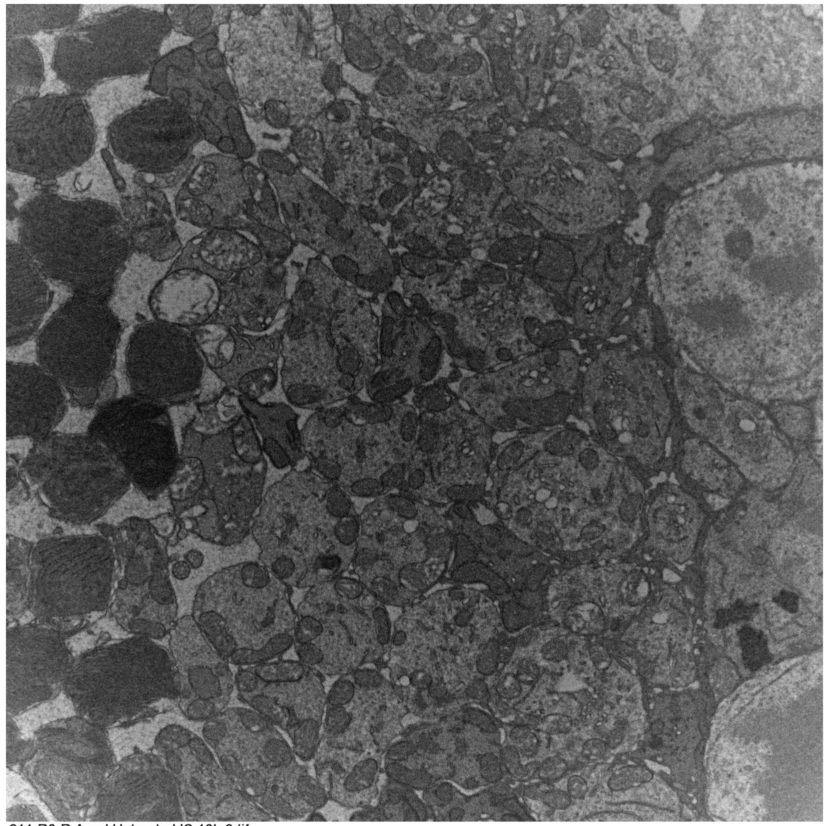

611-R0-R Aged Untreated IS 10k-3.tif
611-R0-R Aged Untreated
Print Mag: 11700x @ 7.0 in
12:03:33 4/27/2023

1 μm
HV=80.0kV
Direct Mag: 10000x
Center for Biologic Imaging

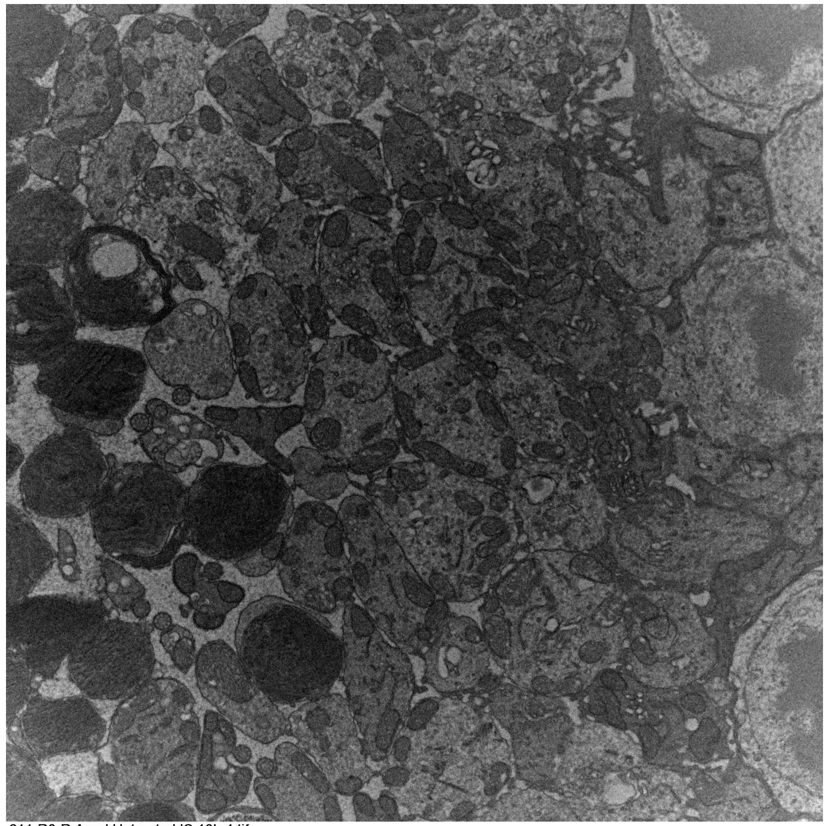

611-R0-R Aged Untreated IS 10k-4.tif
611-R0-R Aged Untreated
Print Mag: 11700x @ 7.0 in
12:06:54 4/27/2023

1 μm
HV=80.0kV
Direct Mag: 10000x
Center for Biologic Imaging

Aged Untreated IS

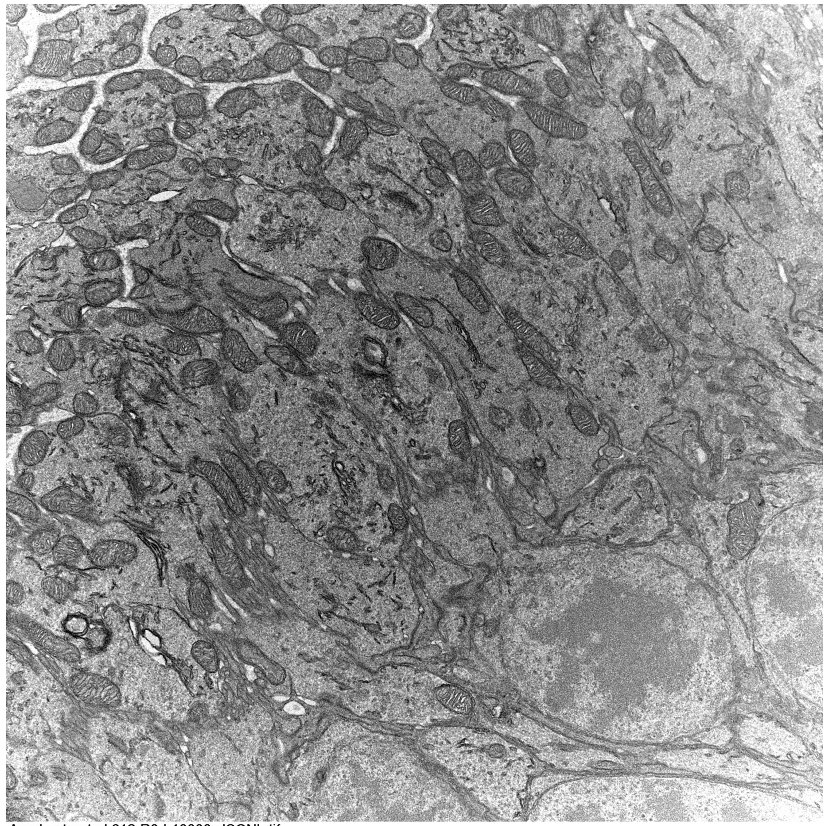

Aged untreated 612-R0-L10000x ISONL.tif
612-R0-L
10000x ISONL2500 RPE+OS210000x
Print Mag: 11700x @ 7.0 in
14:52:34 3/7/2023

1 µm
HV=80.0kV
Direct Mag: 10000x
Center for Biologic Imaging

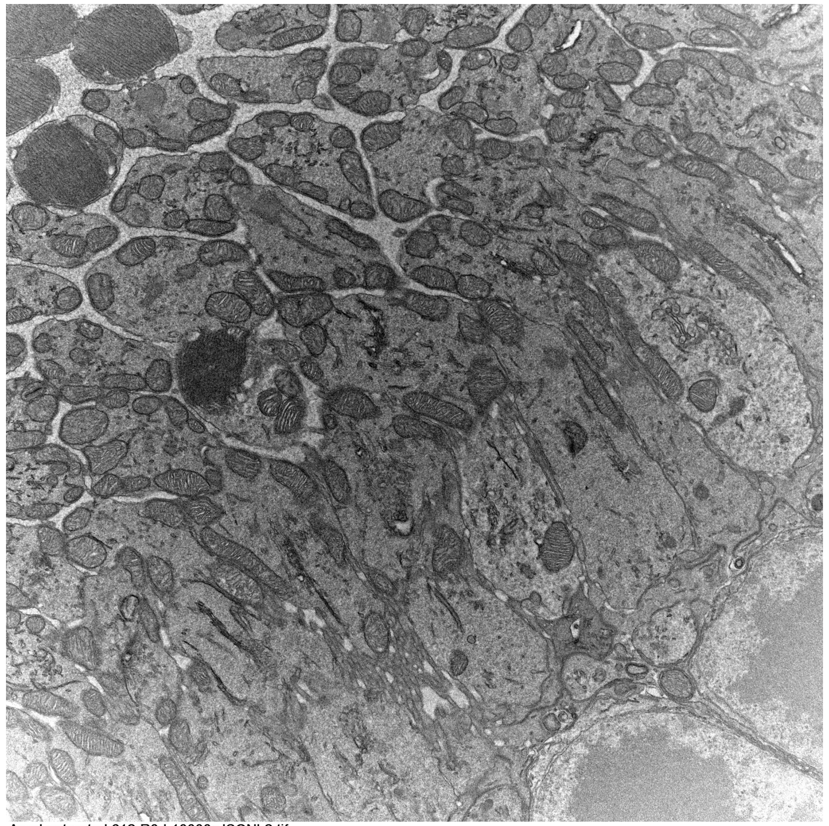

Aged untreated 612-R0-L10000x ISONL2.tif
612-R0-L
10000x ISONL22500 RPE+OS210000x
Print Mag: 11700x @ 7.0 in
14:56:55 3/7/2023

1 µm
HV=80.0kV
Direct Mag: 10000x
Center for Biologic Imaging

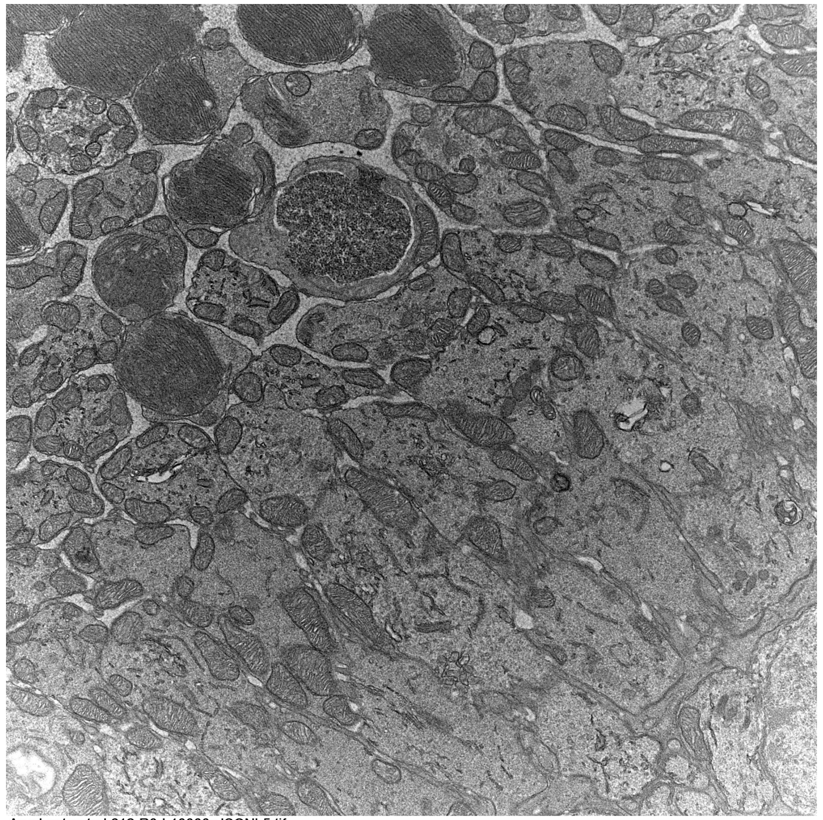

Aged untreated 612-R0-L10000x ISONL5.tif
612-R0-L
10000x ISONL52500 RPE+OS210000x
Print Mag: 11700x @ 7.0 in
15:09:59 3/7/2023

1 µm
HV=80.0kV
Direct Mag: 10000x
Center for Biologic Imaging

Aged Untreated ONL

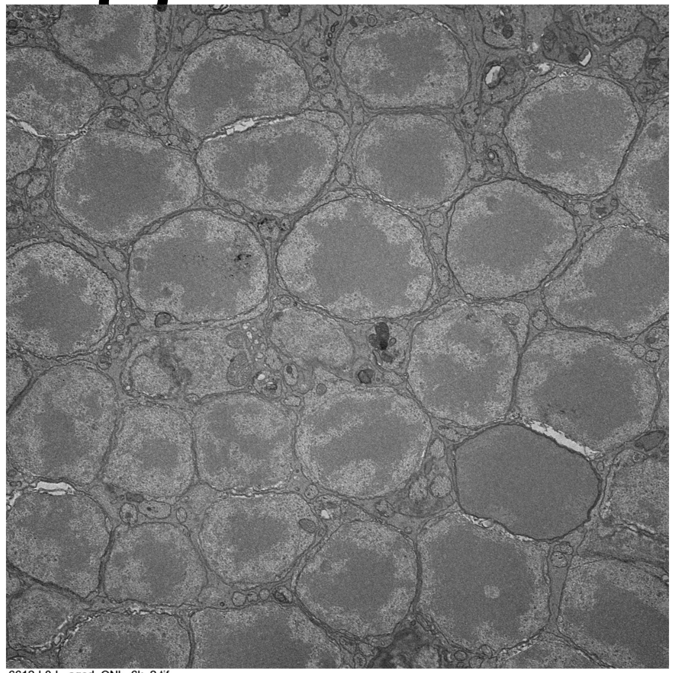

6612-L0-L_aged_ONL_6k_2.tif
6612-L0-L aged untreated 24 mo
Print Mag: 7020x @ 7.0 in
10:07:43 2/17/2023
2 µm
HV=80.0kV
Direct Mag: 6000x
Center for Biologic Imaging

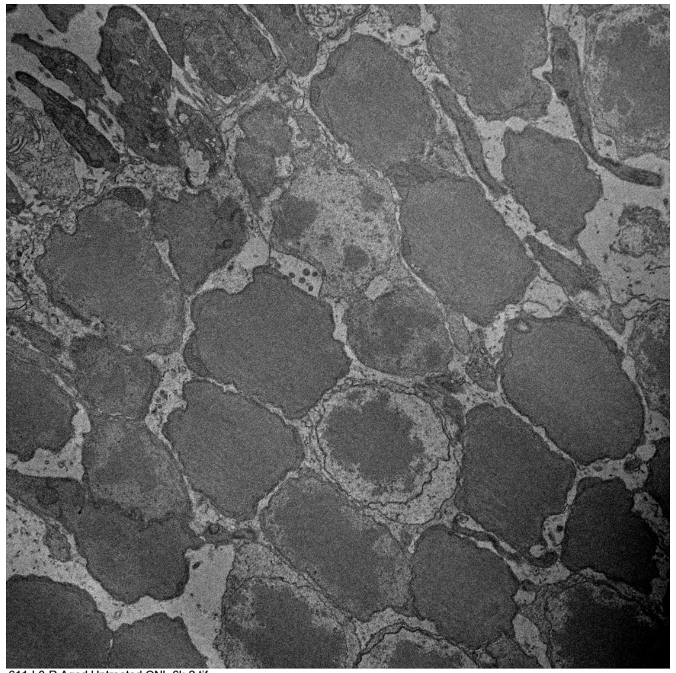

611-L0-R Aged Untreated ONL 6k-2.tif
6609-L0-L Aged Untreated 24mo
Print Mag: 7020x @ 7.0 in
15:25:18 4/13/2023
2 µm
HV=80.0kV
Direct Mag: 6000x
Center for Biologic Imaging

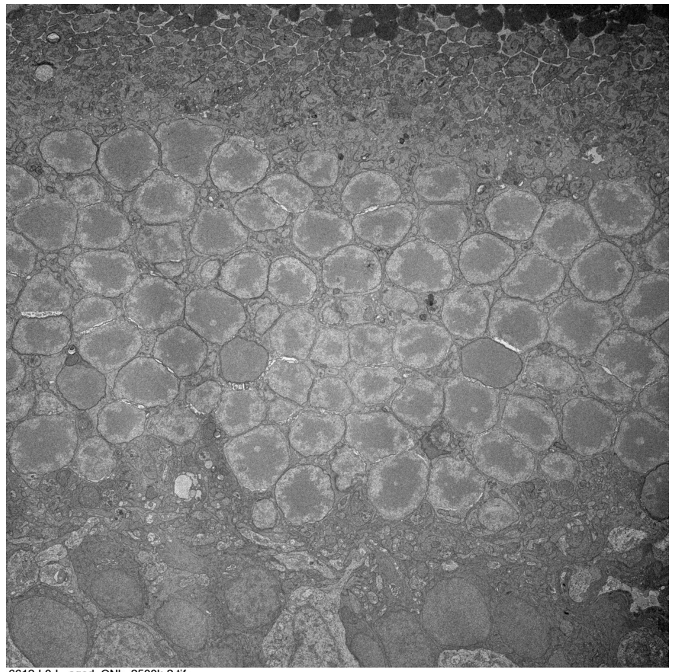

6612-L0-L_aged_ONL_2500k-2.tif
6612-L0-L aged untreated 24 mo
Print Mag: 2920x @ 7.0 in
10:11:58 2/17/2023
6 µm
HV=80.0kV
Direct Mag: 2500x
Center for Biologic Imaging

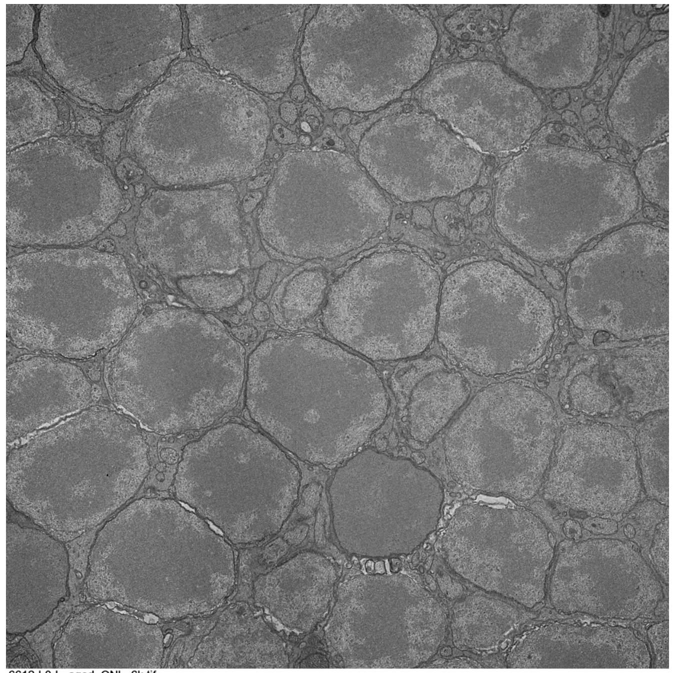

6612-L0-L_aged_ONL_6k.tif
6612-L0-L aged untreated 24 mo
Print Mag: 7020x @ 7.0 in
10:07:07 2/17/2023
2 µm
HV=80.0kV
Direct Mag: 6000x
Center for Biologic Imaging

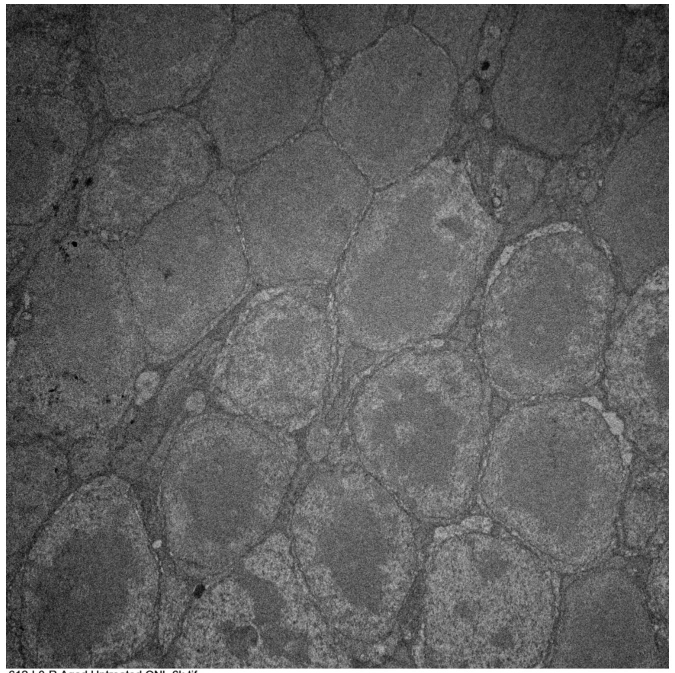

612-L0-R Aged Untreated ONL 6k.tif
6609-L0-L Aged Untreated 24mo
Print Mag: 7020x @ 7.0 in
14:36:33 4/13/2023
2 µm
HV=80.0kV
Direct Mag: 6000x
Center for Biologic Imaging

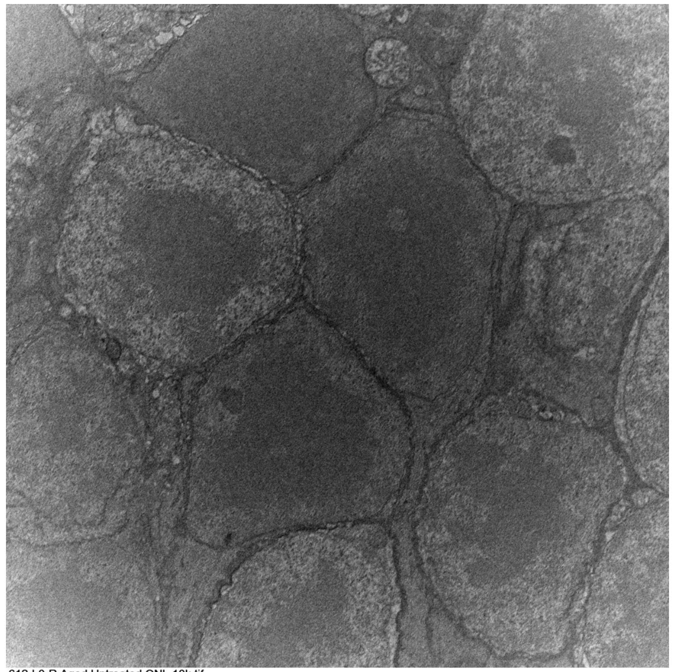

612-L0-R Aged Untreated ONL 10k.tif
6609-L0-L Aged Untreated 24mo
Print Mag: 11700x @ 7.0 in
14:43:12 4/13/2023
1 µm
HV=80.0kV
Direct Mag: 10000x
Center for Biologic Imaging

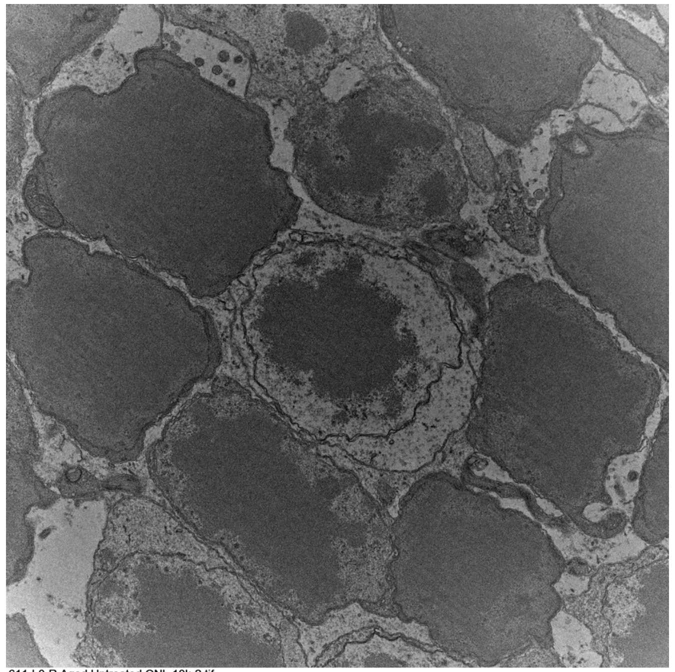

611-L0-R Aged Untreated ONL 10k-2.tif
6609-L0-L Aged Untreated 24mo
Print Mag: 11700x @ 7.0 in
15:33:37 4/13/2023
1 µm
HV=80.0kV
Direct Mag: 10000x
Center for Biologic Imaging

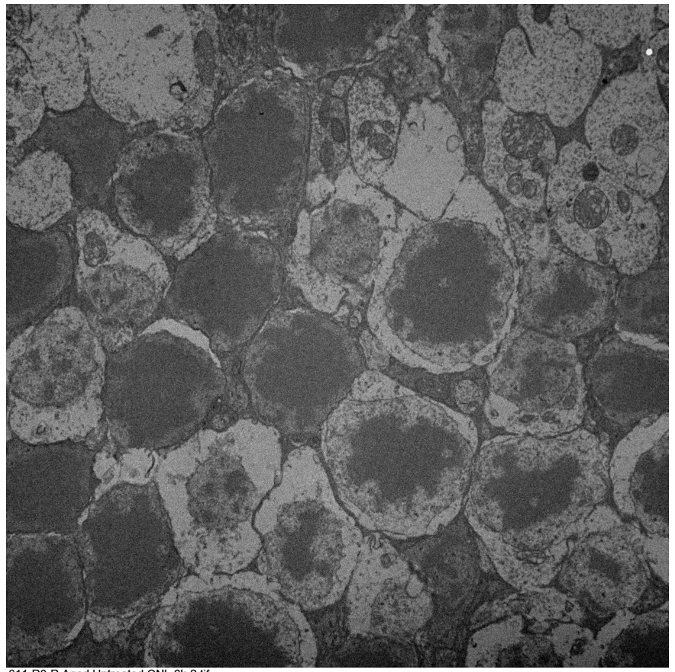

611-R0-R Aged Untreated ONL 6k-2.tif
611-R0-R Aged Untreated
Print Mag: 7020x @ 7.0 in
11:34:53 4/27/2023
2 µm
HV=80.0kV
Direct Mag: 6000x
Center for Biologic Imaging

Aged Untreated ONL

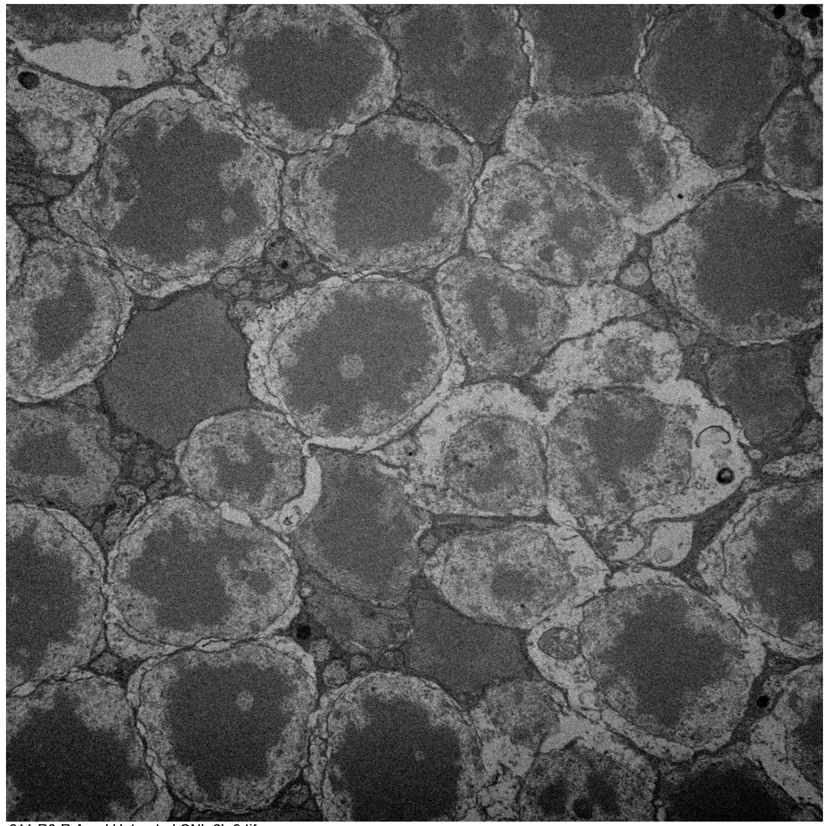

611-R0-R Aged Untreated ONL 6k-3.tif
611-R0-R Aged Untreated
Print Mag: 7020x @ 7.0 in
11:52:53 4/27/2023

2 µm
HV=80.0kV
Direct Mag: 6000x
Center for Biologic Imaging

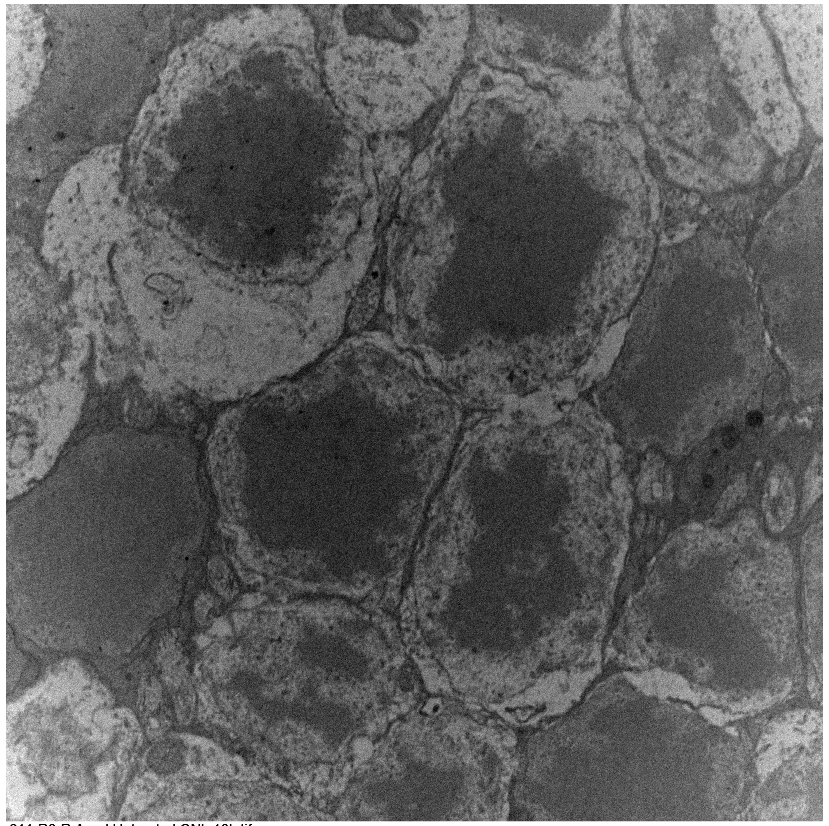

611-R0-R Aged Untreated ONL 10k.tif
611-R0-R Aged Untreated
Print Mag: 11700x @ 7.0 in
11:26:59 4/27/2023

1 µm
HV=80.0kV
Direct Mag: 10000x
Center for Biologic Imaging

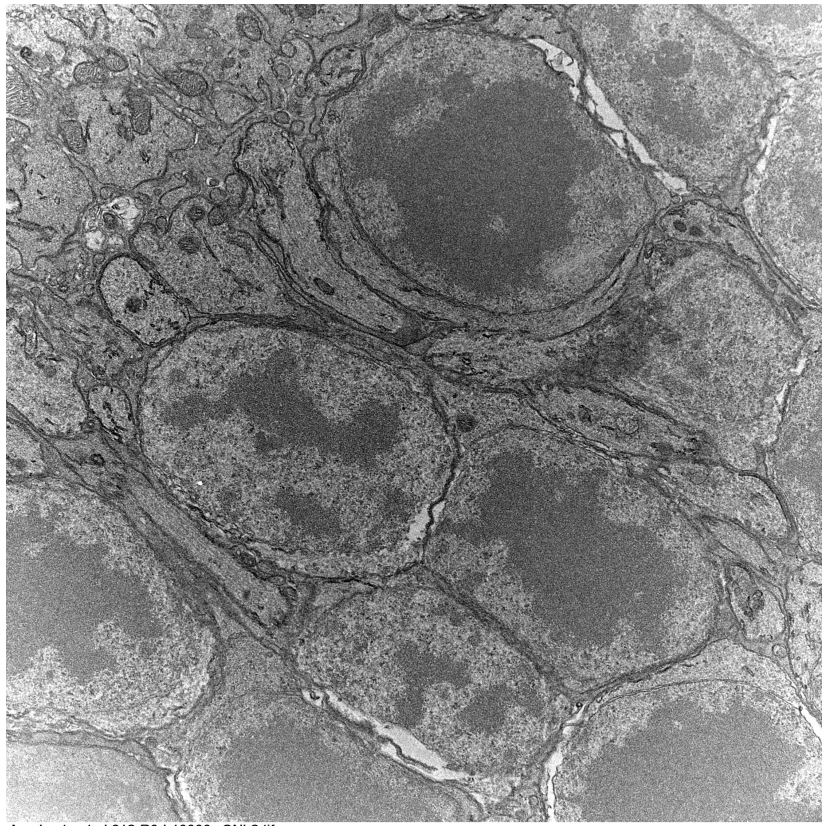

Aged untreated 612-R0-L10000x ONL2.tif
612-R0-L
10000xONL22500 RPE+OS210000x
Print Mag: 11700x @ 7.0 in
15:16:44 3/7/2023

1 µm
HV=80.0kV
Direct Mag: 10000x
Center for Biologic Imaging

Aged Untreated OPL

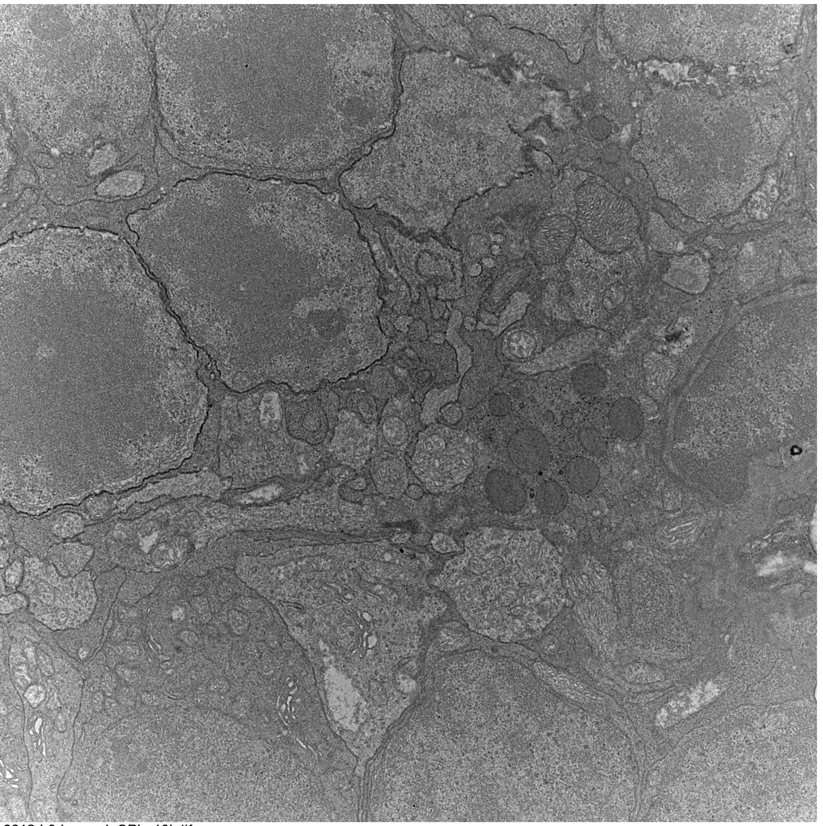

6612-L0-L_aged_OPL_10k.tif
6612-L0-L aged untreated 24 mo
Print Mag: 11700x @ 7.0 in
10:52:54 2/17/2023

1 µm
HV=80.0kV
Direct Mag: 10000x
Center for Biologic Imaging

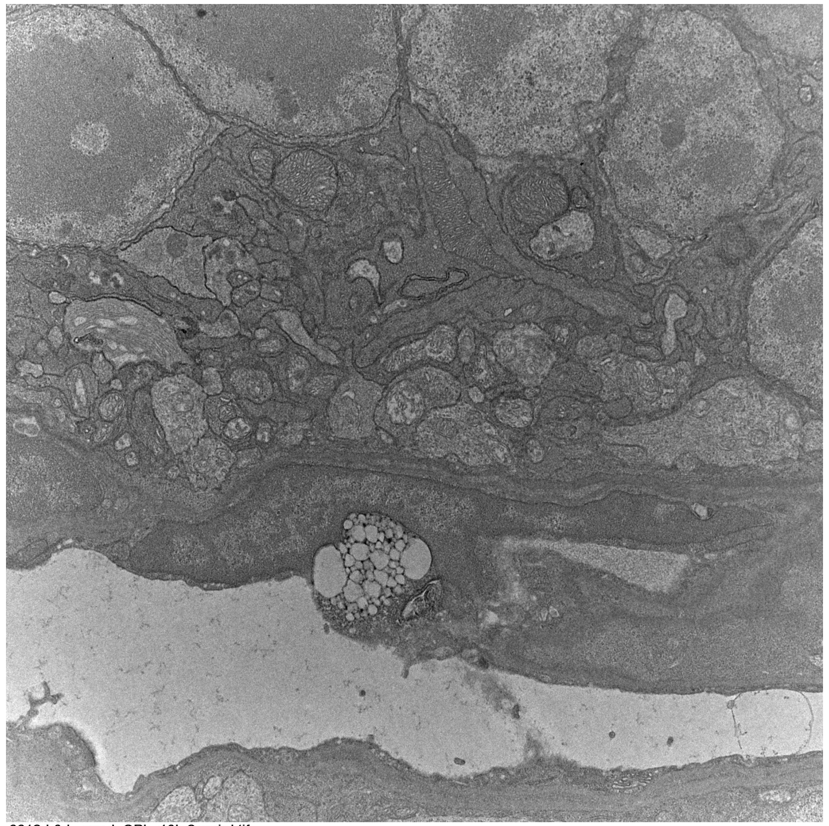

6612-L0-L_aged_OPL_10k-2 weird.tif
6612-L0-L aged untreated 24 mo
Print Mag: 11700x @ 7.0 in
10:55:12 2/17/2023

1 µm
HV=80.0kV
Direct Mag: 10000x
Center for Biologic Imaging

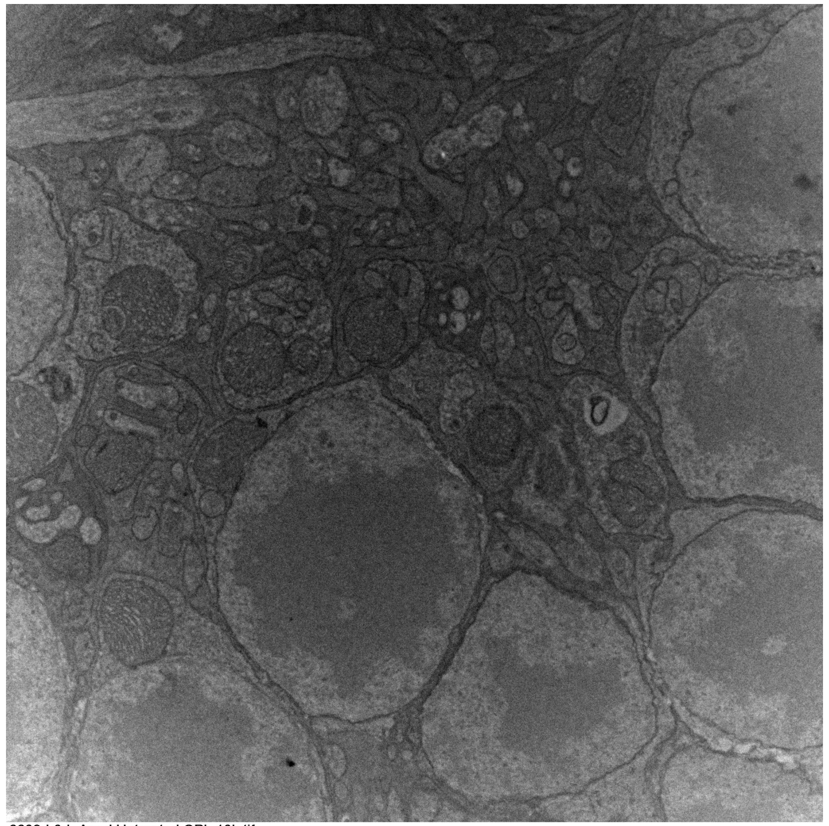

6609-L0-L Aged Untreated OPL 10k.tif
6609-L0-L Aged Untreated 24mo
Print Mag: 11700x @ 7.0 in
13:43:50 4/13/2023

1 µm
HV=80.0kV
Direct Mag: 10000x
Center for Biologic Imaging

Aged Untreated OPL

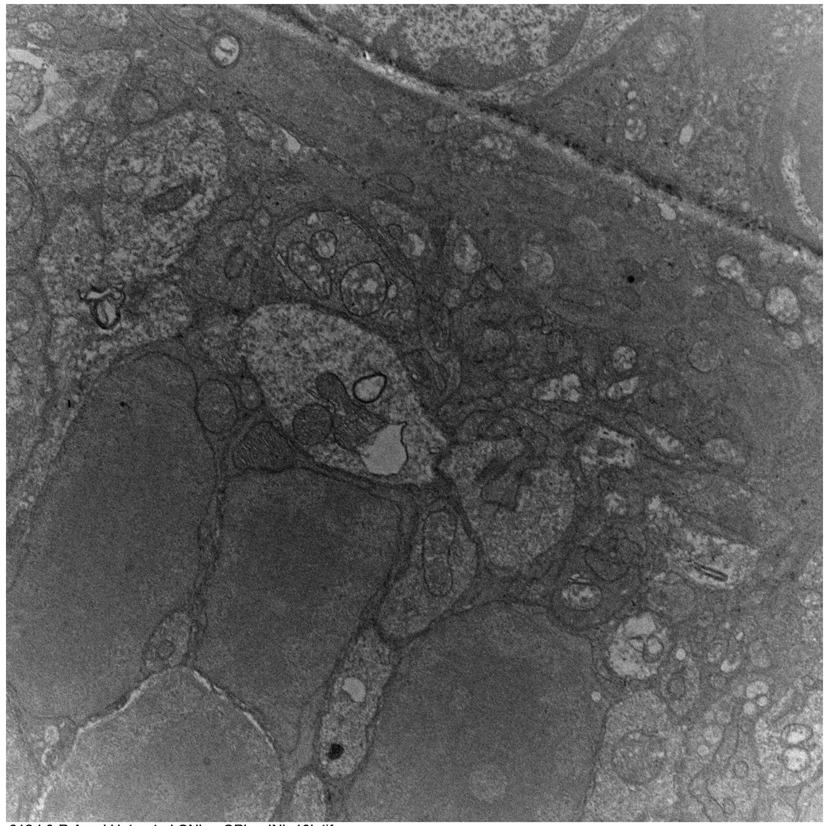

612-L0-R Aged Untreated ONL + OPL + INL 10k.tif
6609-L0-L Aged Untreated 24mo
Print Mag: 11700x @ 7.0 in
15:12:49 4/13/2023

1 µm
HV=80.0kV
Direct Mag: 10000x
Center for Biologic Imaging

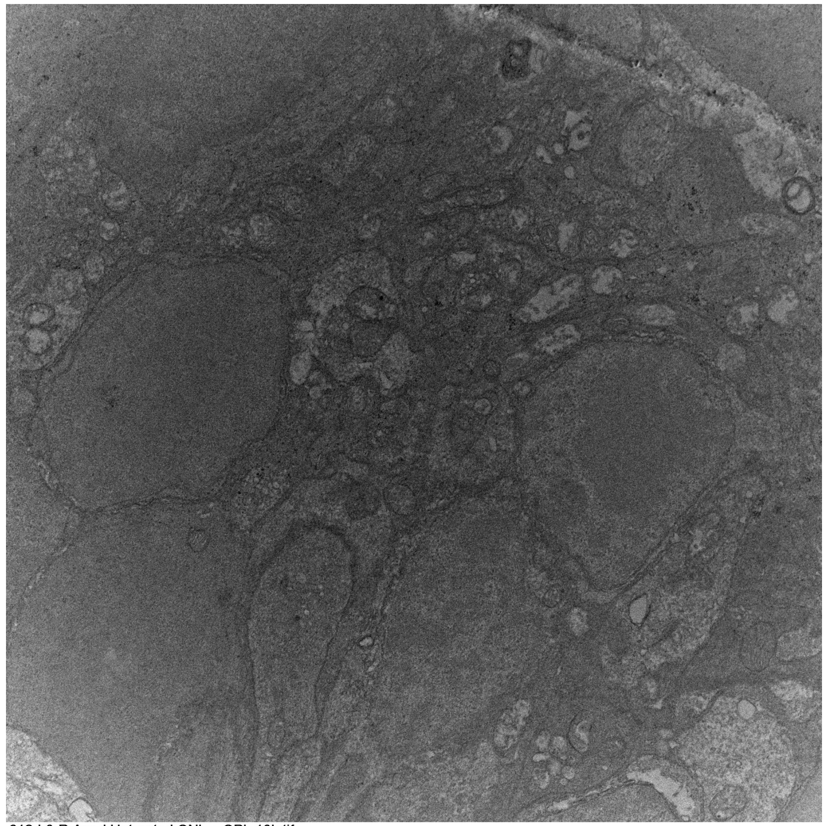

612-L0-R Aged Untreated ONL + OPL 10k.tif
6609-L0-L Aged Untreated 24mo
Print Mag: 11700x @ 7.0 in
14:44:37 4/13/2023

1 µm
HV=80.0kV
Direct Mag: 10000x
Center for Biologic Imaging

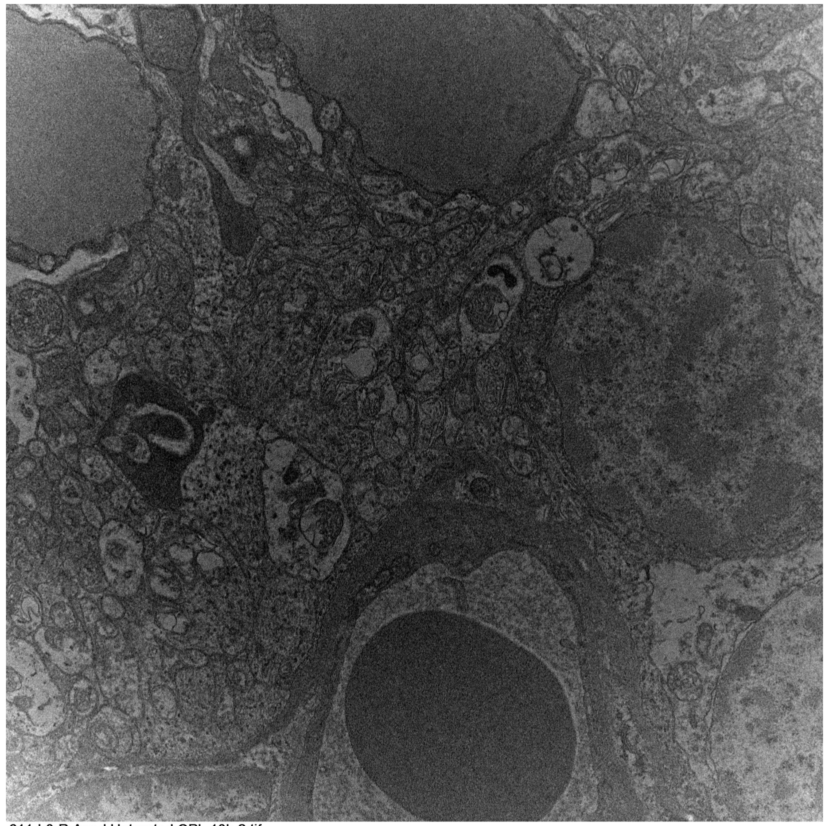

611-L0-R Aged Untreated OPL 10k-2.tif
6609-L0-L Aged Untreated 24mo
Print Mag: 11700x @ 7.0 in
15:43:13 4/13/2023

1 µm
HV=80.0kV
Direct Mag: 10000x
Center for Biologic Imaging

Aged Untreated OPL

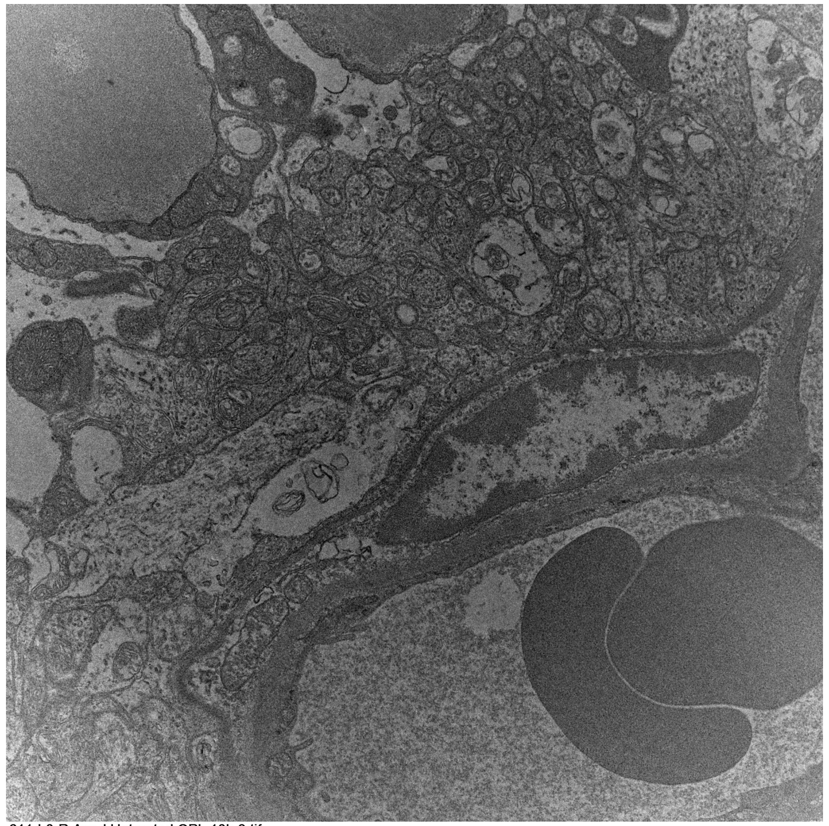

611-L0-R Aged Untreated OPL 10k-3.tif
6609-L0-L Aged Untreated 24mo
Print Mag: 11700x @ 7.0 in
15:44:23 4/13/2023

1 μm
HV=80.0kV
Direct Mag: 10000x
Center for Biologic Imaging

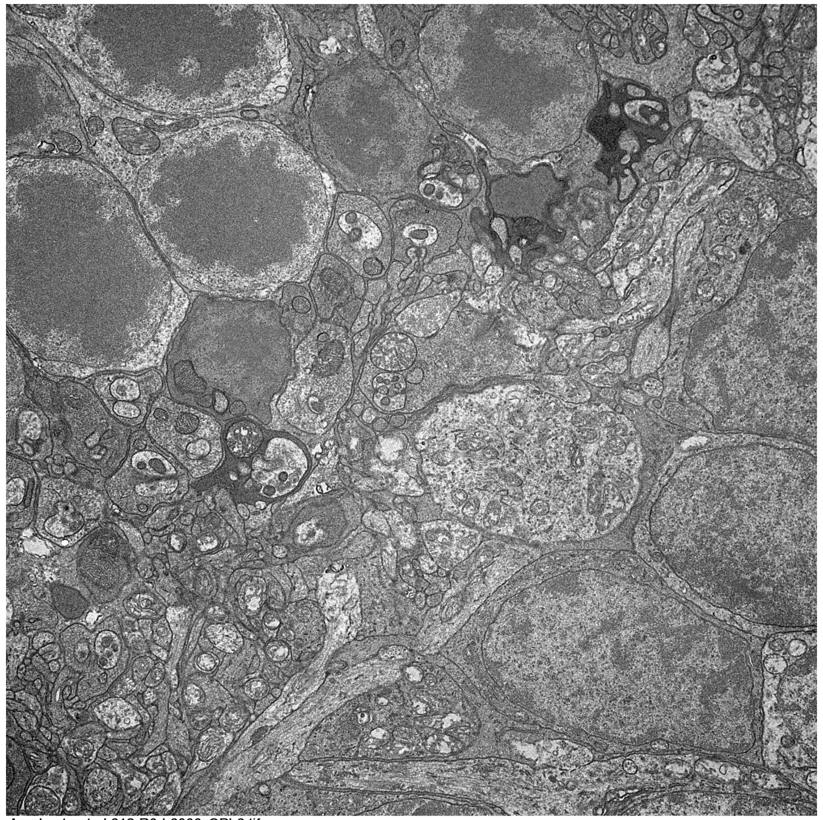

Aged untreated 612-R0-L6000xOPL6.tif
612-R0-L
OPL 6000x6
Print Mag: 7020x @ 7.0 in
16:37:27 3/7/2023

2 μm
HV=80.0kV
Direct Mag: 6000x
Center for Biologic Imaging

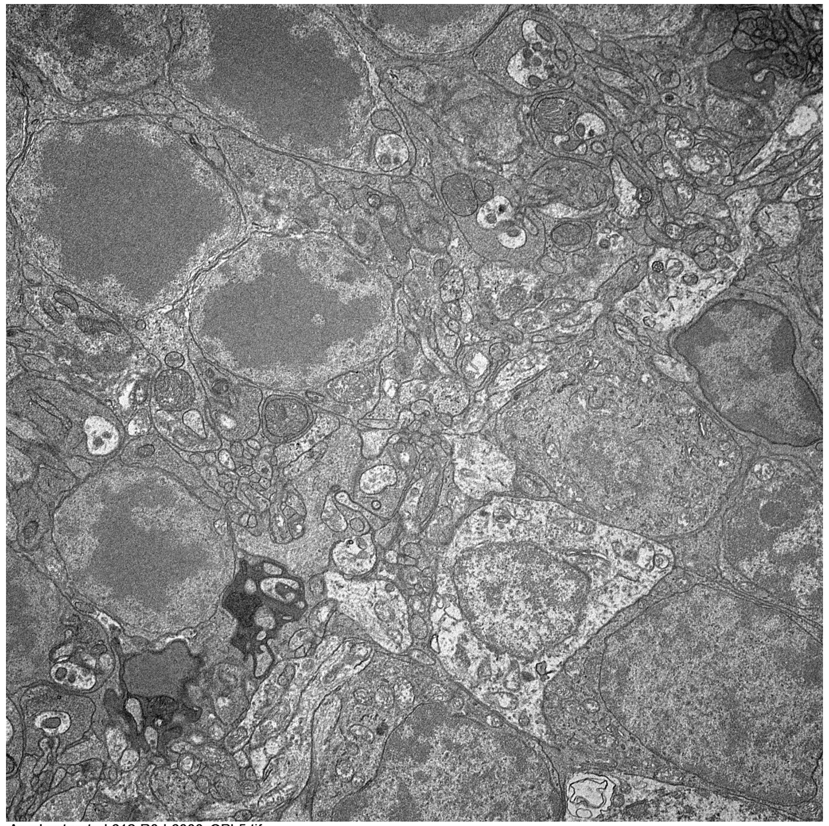

Aged untreated 612-R0-L6000xOPL5.tif
612-R0-L
OPL 6000x5
Print Mag: 7020x @ 7.0 in
16:36:20 3/7/2023

2 μm
HV=80.0kV
Direct Mag: 6000x
Center for Biologic Imaging

Aged Untreated OPL

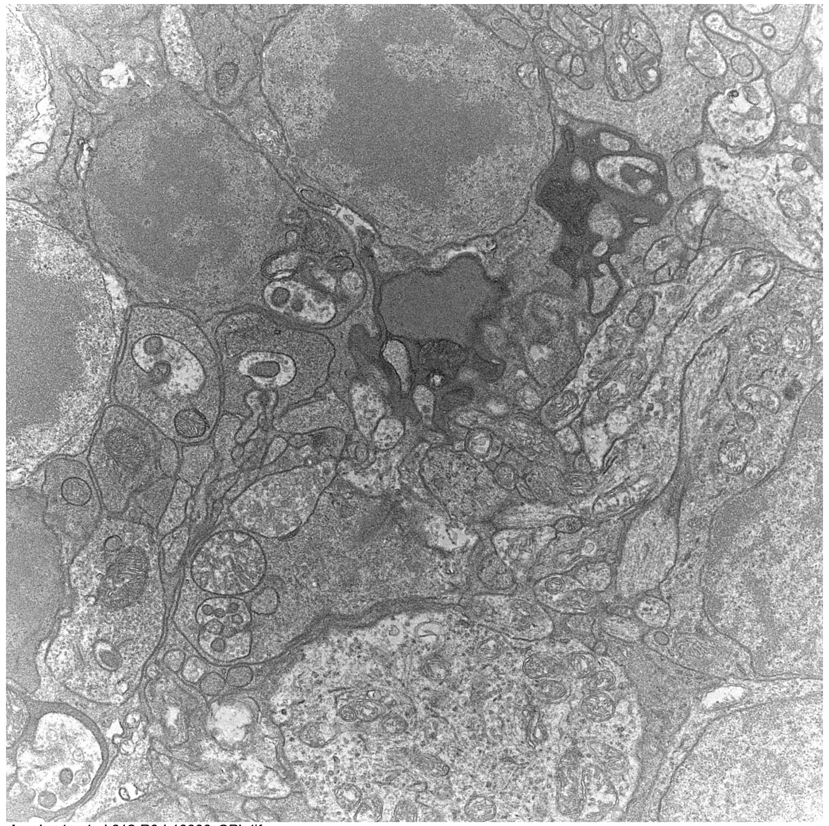

Aged untreated 612-R0-L10000xOPL.tif
612-R0-L
10000xOPL2500 RPE+OS210000x
Print Mag: 11700x @ 7.0 in
16:16:08 3/7/2023

1 µm
HV=80.0kV
Direct Mag: 10000x
Center for Biologic Imaging

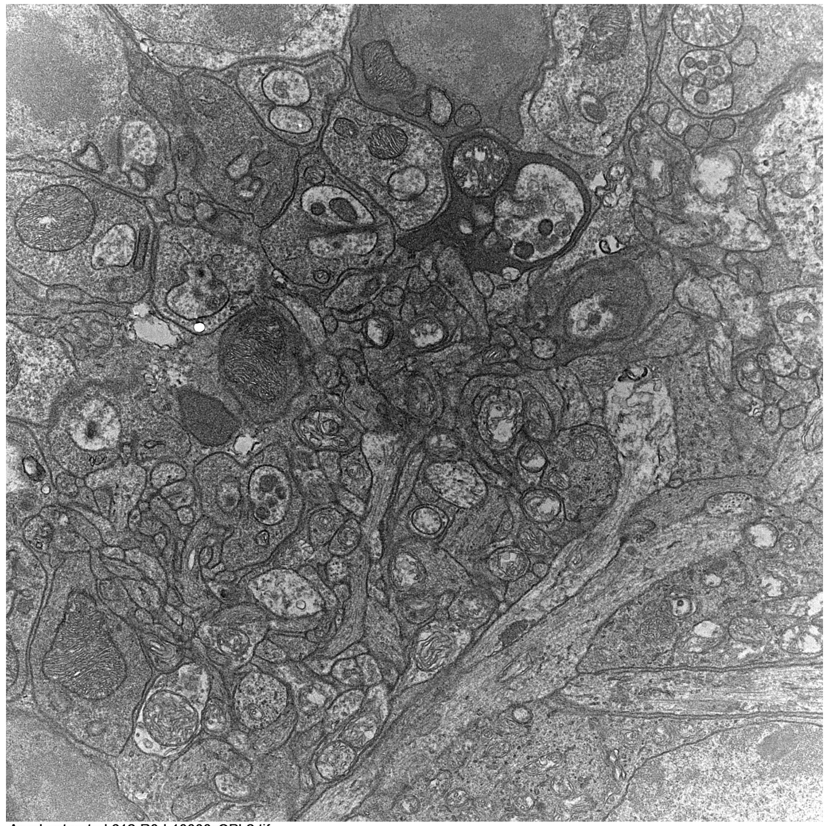

Aged untreated 612-R0-L10000xOPL2.tif
612-R0-L
10000xOPL22500 RPE+OS210000x
Print Mag: 11700x @ 7.0 in
16:17:57 3/7/2023

1 µm
HV=80.0kV
Direct Mag: 10000x
Center for Biologic Imaging

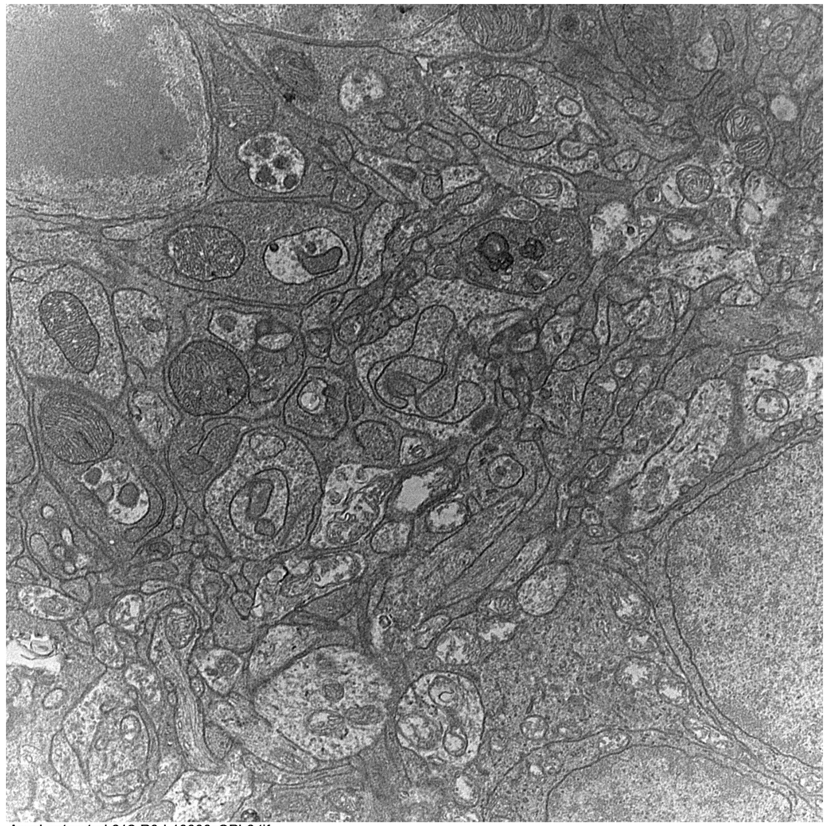

Aged untreated 612-R0-L10000xOPL6.tif
612-R0-L
10000xOPL62500 RPE+OS210000x
Print Mag: 11700x @ 7.0 in
16:26:26 3/7/2023

1 µm
HV=80.0kV
Direct Mag: 10000x
Center for Biologic Imaging

Aged Untreated INL

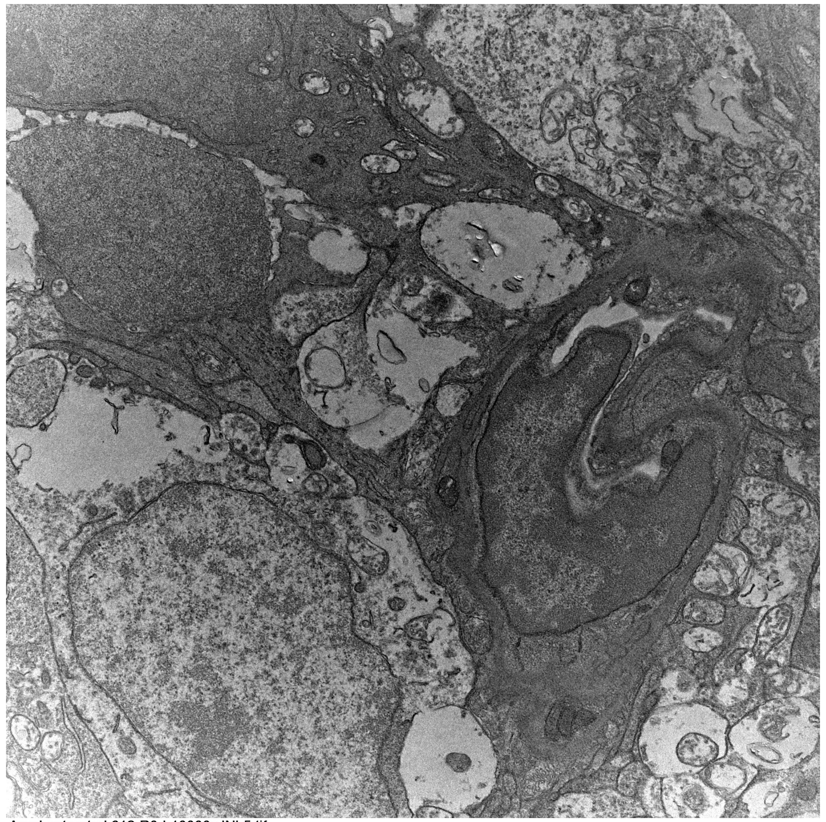

Aged untreated 612-R0-L10000x INL5.tif
612-R0-L
10000xINL52500 RPE+OS210000x
Print Mag: 11700x @ 7.0 in
15:27:48 3/7/2023

1 µm
HV=80.0kV
Direct Mag: 10000x
Center for Biologic Imaging

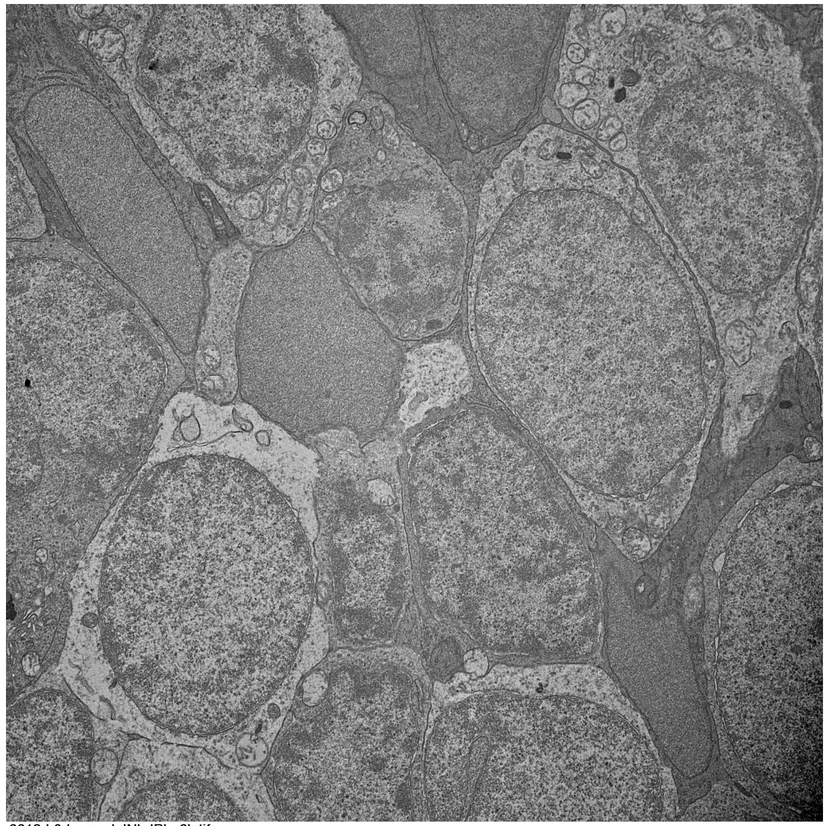

6612-L0-L_aged_INL-IPL_6k.tif
6612-L0-L aged untreated 24 mo
Print Mag: 7020x @ 7.0 in
10:20:36 2/17/2023

2 µm
HV=80.0kV
Direct Mag: 6000x
Center for Biologic Imaging

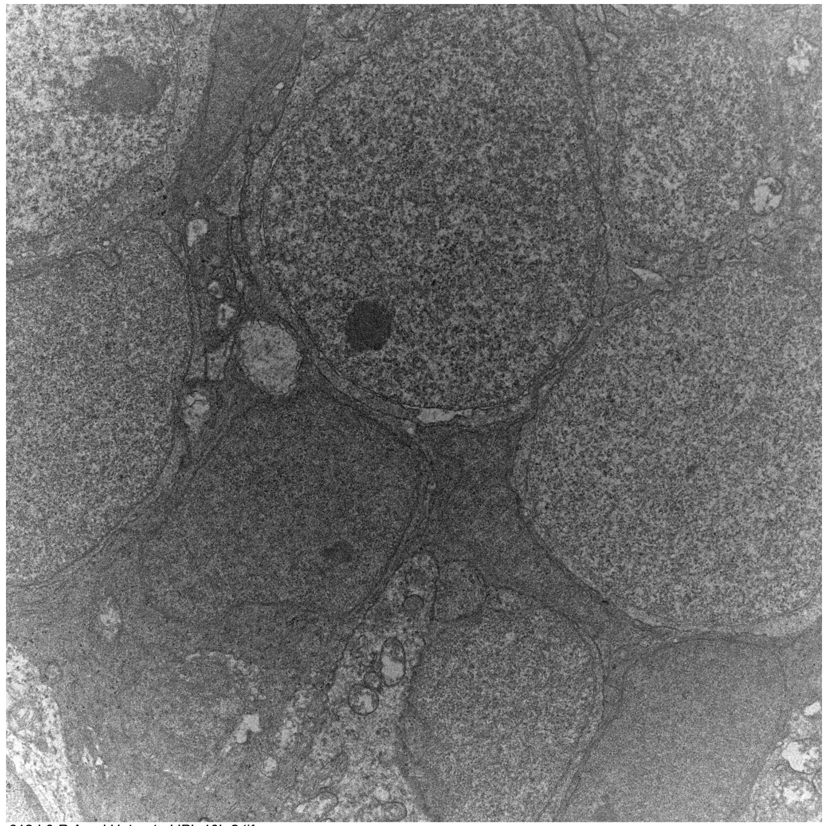

612-L0-R Aged Untreated IPL 10k-2.tif
6609-L0-L Aged Untreated 24mo
Print Mag: 11700x @ 7.0 in
15:00:54 4/13/2023

1 µm
HV=80.0kV
Direct Mag: 10000x
Center for Biologic Imaging

Aged Untreated INL

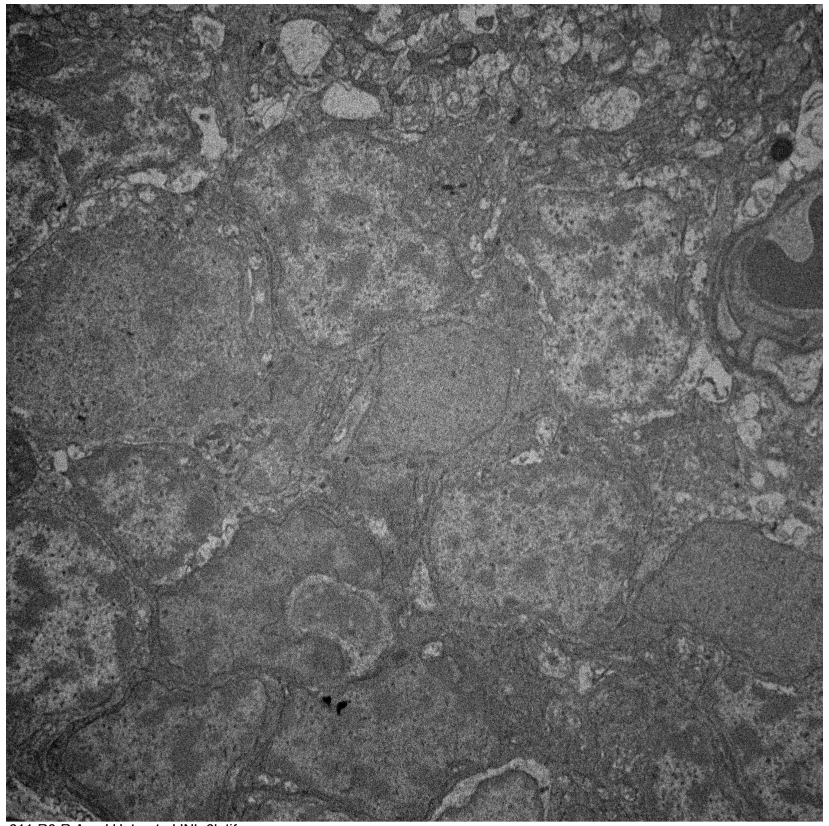

611-R0-R Aged Untreated INL 6k.tif
611-R0-R Aged Untreated
Print Mag: 7020x @ 7.0 in
11:30:55 4/27/2023

2 μm
HV=80.0kV
Direct Mag: 6000x
Center for Biologic Imaging

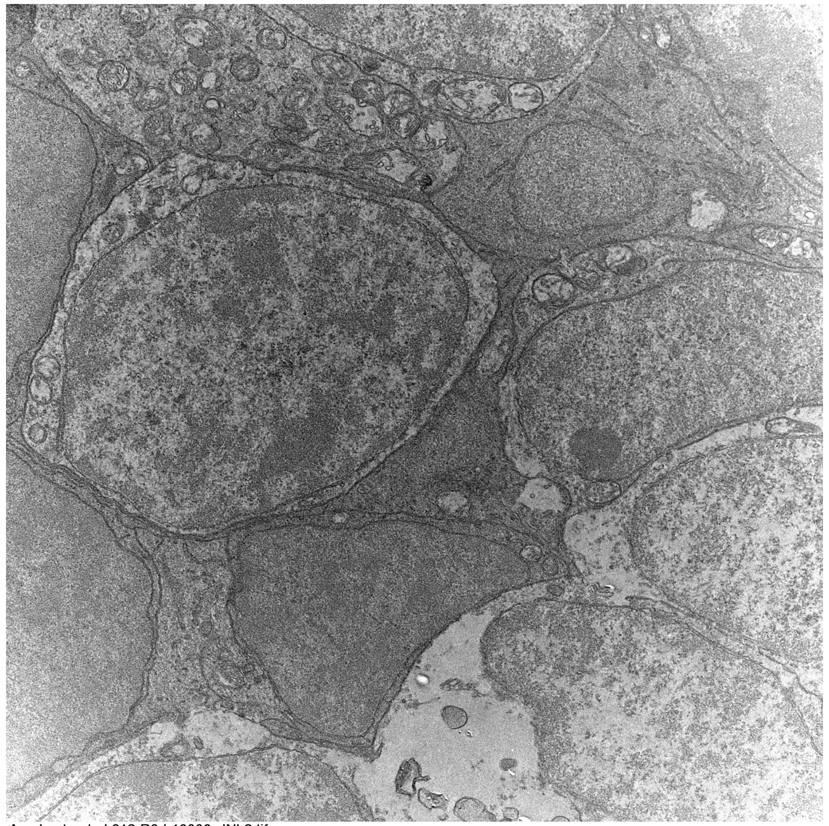

Aged untreated 612-R0-L10000x INL2.tif
612-R0-L
10000xINL22500 RPE+OS210000x
Print Mag: 11700x @ 7.0 in
15:21:39 3/7/2023

1 μm
HV=80.0kV
Direct Mag: 10000x
Center for Biologic Imaging

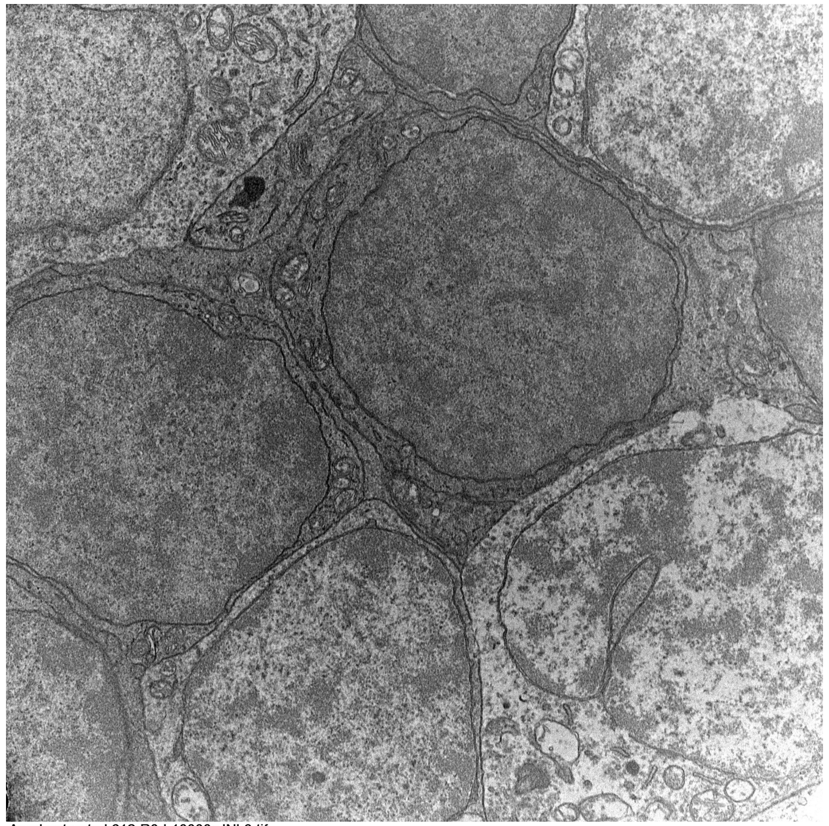

Aged untreated 612-R0-L10000x INL3.tif
612-R0-L
10000xINL32500 RPE+OS210000x
Print Mag: 11700x @ 7.0 in
15:23:21 3/7/2023

1 μm
HV=80.0kV
Direct Mag: 10000x
Center for Biologic Imaging

Aged Untreated IPL

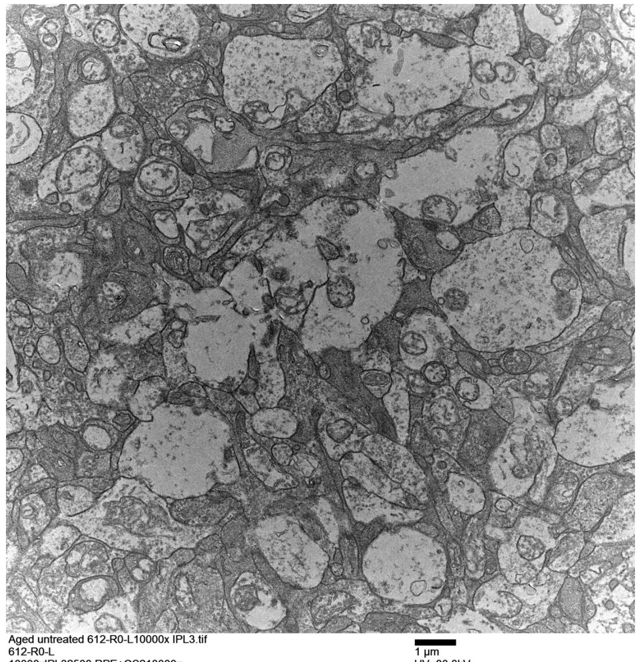

Aged untreated 612-R0-L10000x IPL3.tif
612-R0-L
10000xIPL32500 RPE+OS210000x
Print Mag: 11700x @ 7.0 in
15:35:06 3/7/2023

1 µm
HV=80.0kV
Direct Mag: 10000x
Center for Biologic Imaging

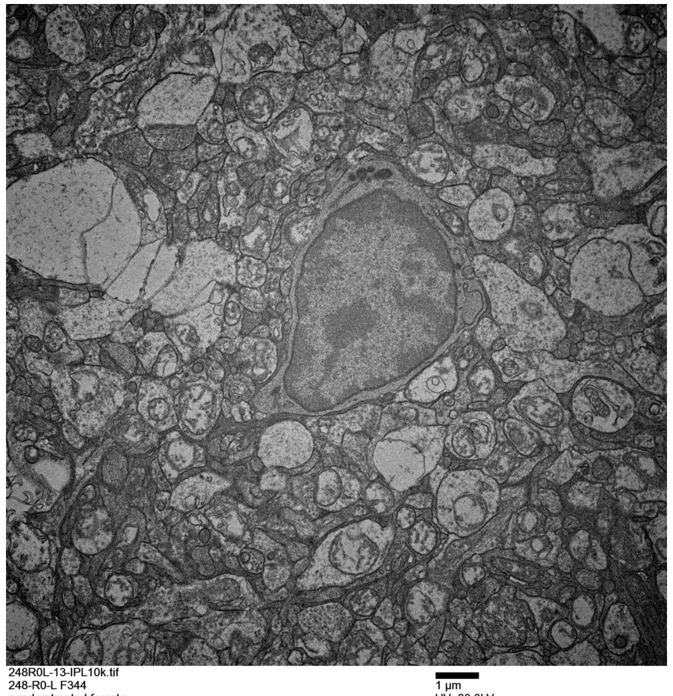

248R0L-13-IPL10k.tif
248-R0-L F344
aged untreated female
Print Mag: 11700x @ 7.0 in
15:11:51 2/2/2023

1 µm
HV=80.0kV
Direct Mag: 10000x
Center for Biologic Imaging

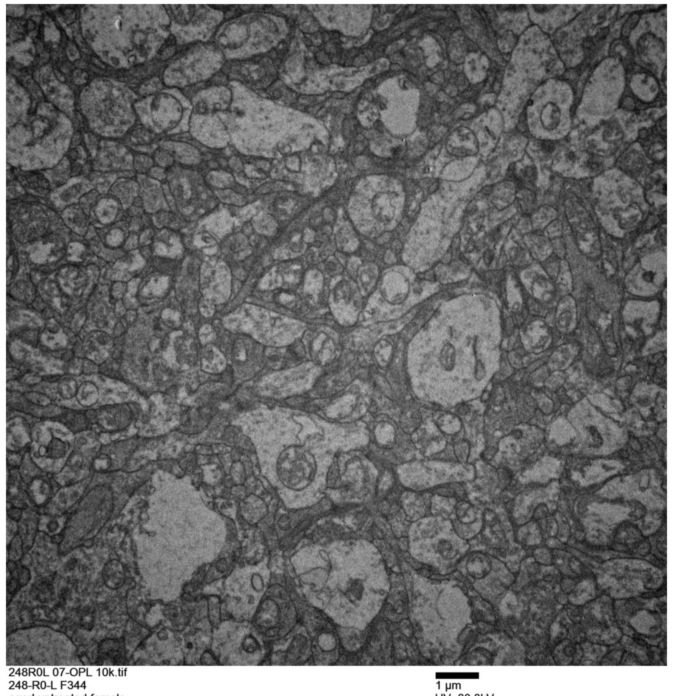

248R0L 07-OPL 10k.tif
248-R0-L F344
aged untreated female
Print Mag: 11700x @ 7.0 in
14:13:24 2/2/2023

1 µm
HV=80.0kV
Direct Mag: 10000x
Center for Biologic Imaging

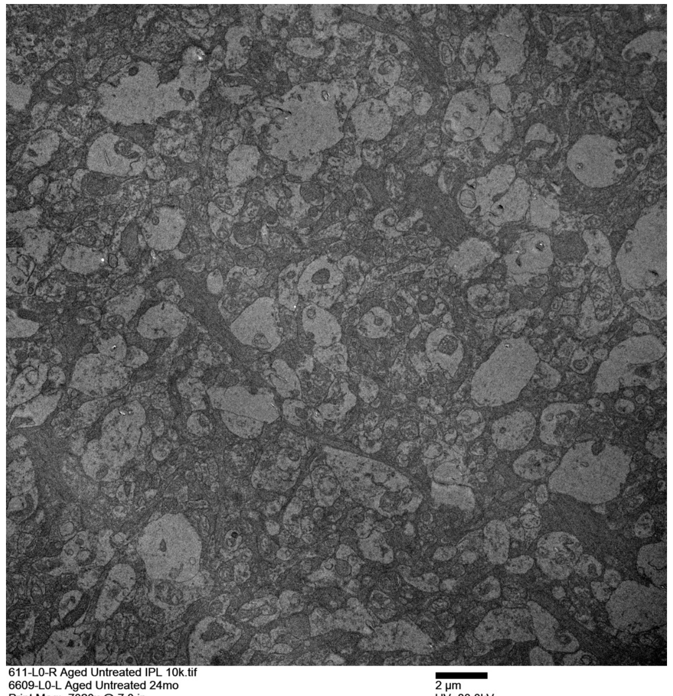

611-L0-R Aged Untreated IPL 10k.tif
6609-L0-L Aged Untreated 24mo
Print Mag: 7020x @ 7.0 in
15:49:15 4/13/2023

2 µm
HV=80.0kV
Direct Mag: 6000x
Center for Biologic Imaging

Aged Untreated IPL

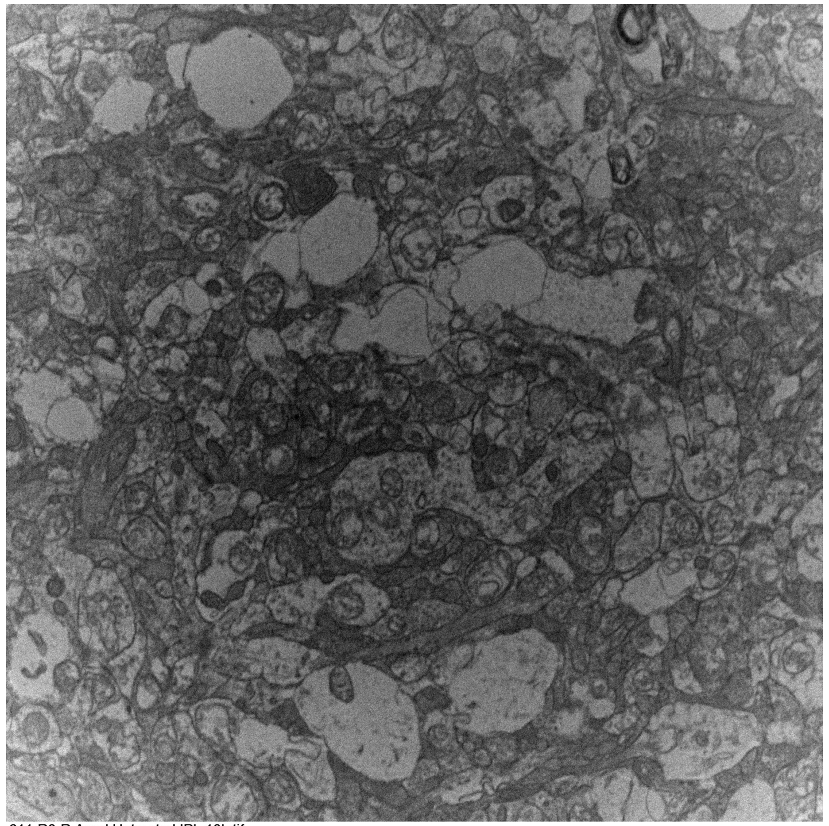

611-R0-R Aged Untreated IPL 10k.tif
611-R0-R Aged Untreated
Print Mag: 11700x @ 7.0 in
11:33:19 4/27/2023

1 µm
HV=80.0kV
Direct Mag: 10000x
Center for Biologic Imaging

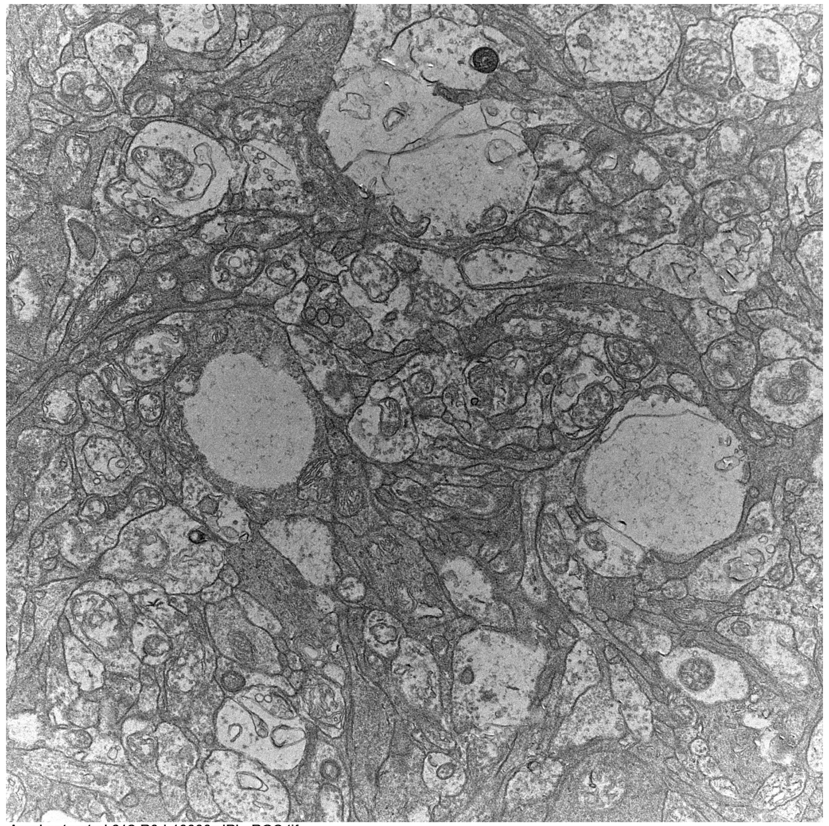

Aged untreated 612-R0-L10000x IPL_RGC.tif
612-R0-L
10000xIPLRGC2500 RPE+OS210000x
Print Mag: 11700x @ 7.0 in
15:40:35 3/7/2023

1 µm
HV=80.0kV
Direct Mag: 10000x
Center for Biologic Imaging

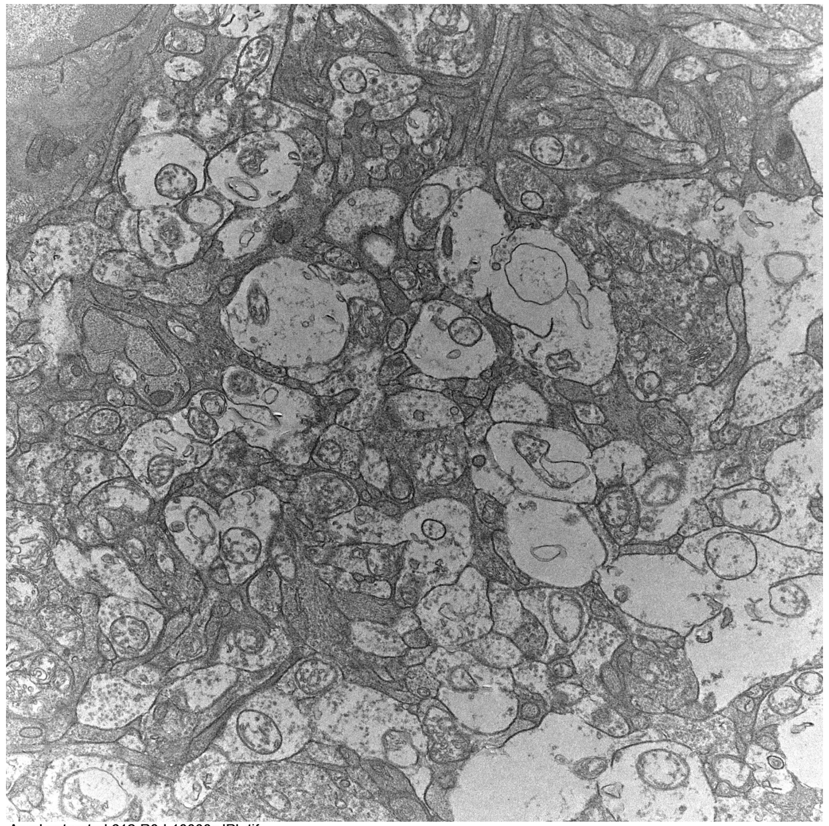

Aged untreated 612-R0-L10000x IPL.tif
612-R0-L
10000xIPL2500 RPE+OS210000x
Print Mag: 11700x @ 7.0 in
15:30:22 3/7/2023

1 µm
HV=80.0kV
Direct Mag: 10000x
Center for Biologic Imaging

Untreated aged central-RGC-RNFL

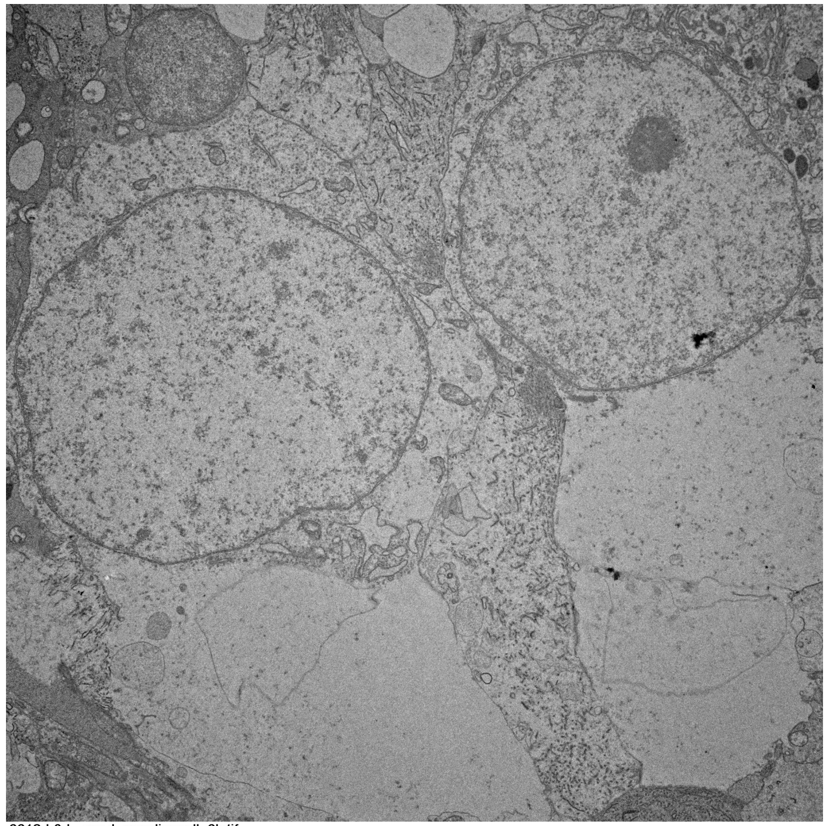

6612-L0-L_aged_ganglioncell_6k.tif
6612-L0-L aged untreated 24 mo
Print Mag: 7020x @ 7.0 in
12:01:56 2/17/2023

2 µm
HV=80.0kV
Direct Mag: 6000x
Center for Biologic Imaging

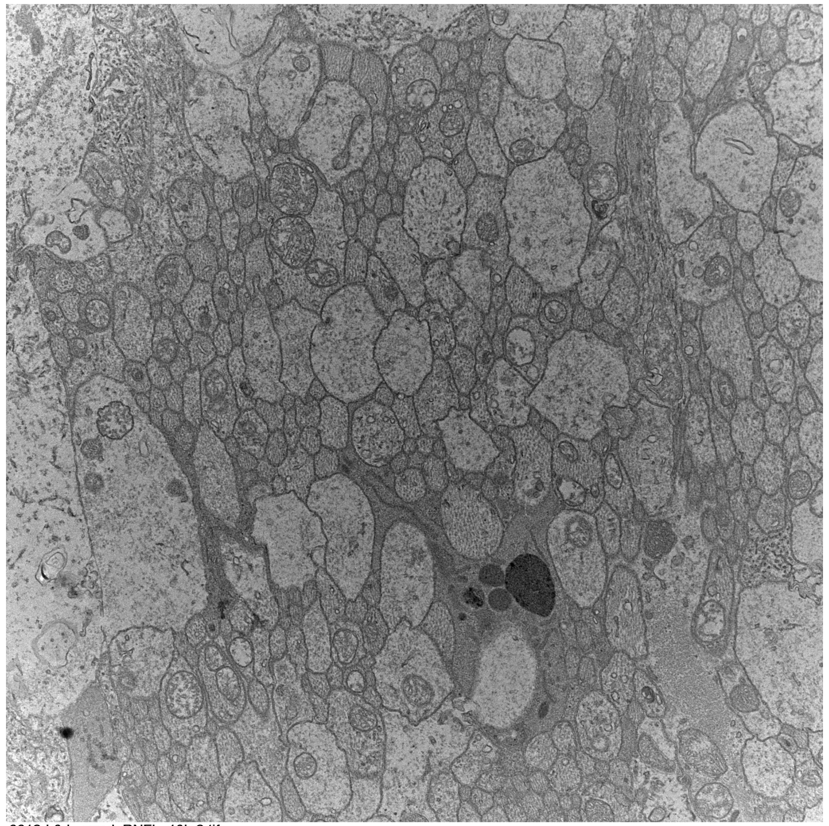

6612-L0-L_aged_RNFL_10k-2.tif
6612-L0-L aged untreated 24 mo
Print Mag: 11700x @ 7.0 in
10:46:15 2/17/2023

1 µm
HV=80.0kV
Direct Mag: 10000x
Center for Biologic Imaging

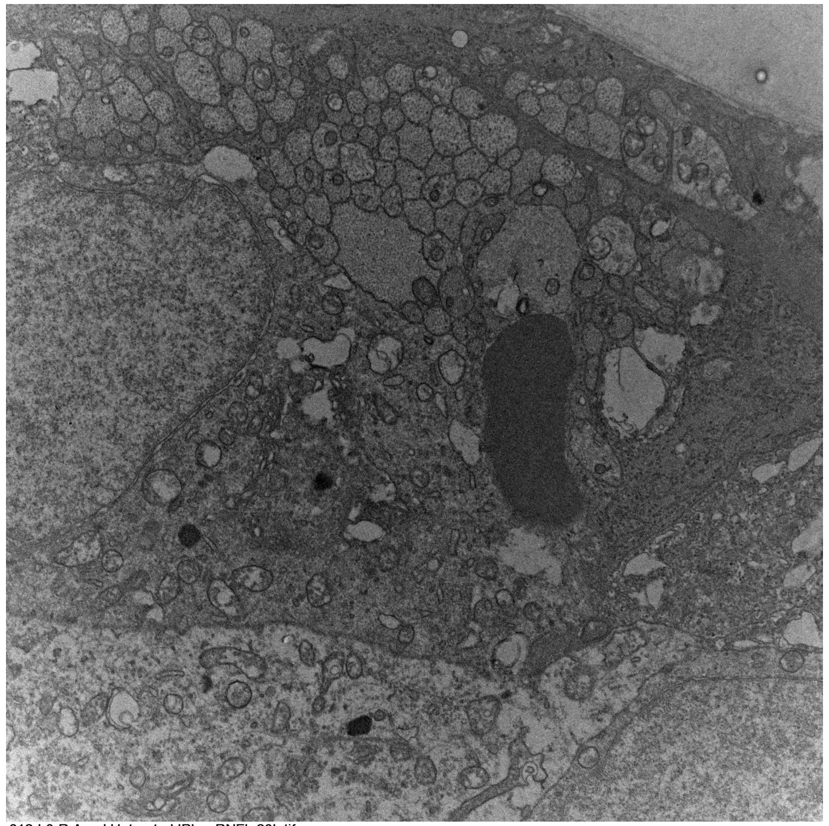

612-L0-R Aged Untreated IPL + RNFL 20k.tif
6609-L0-L Aged Untreated 24mo
Print Mag: 11700x @ 7.0 in
14:57:32 4/13/2023

1 µm
HV=80.0kV
Direct Mag: 10000x
Center for Biologic Imaging

Aged Untreated RGC-RNFL

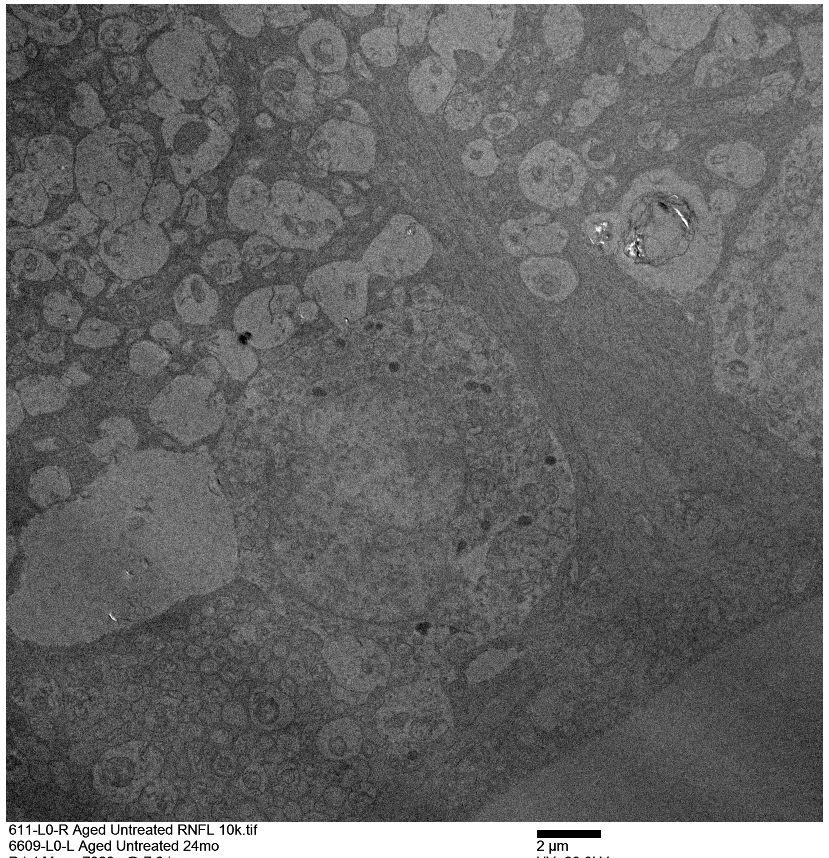

611-L0-R Aged Untreated RNFL 10k.tif
6609-L0-L Aged Untreated 24mo
Print Mag: 7020x @ 7.0 in
15:35:57 4/13/2023
2 µm
HV=80.0kV
Direct Mag: 6000x
Center for Biologic Imaging

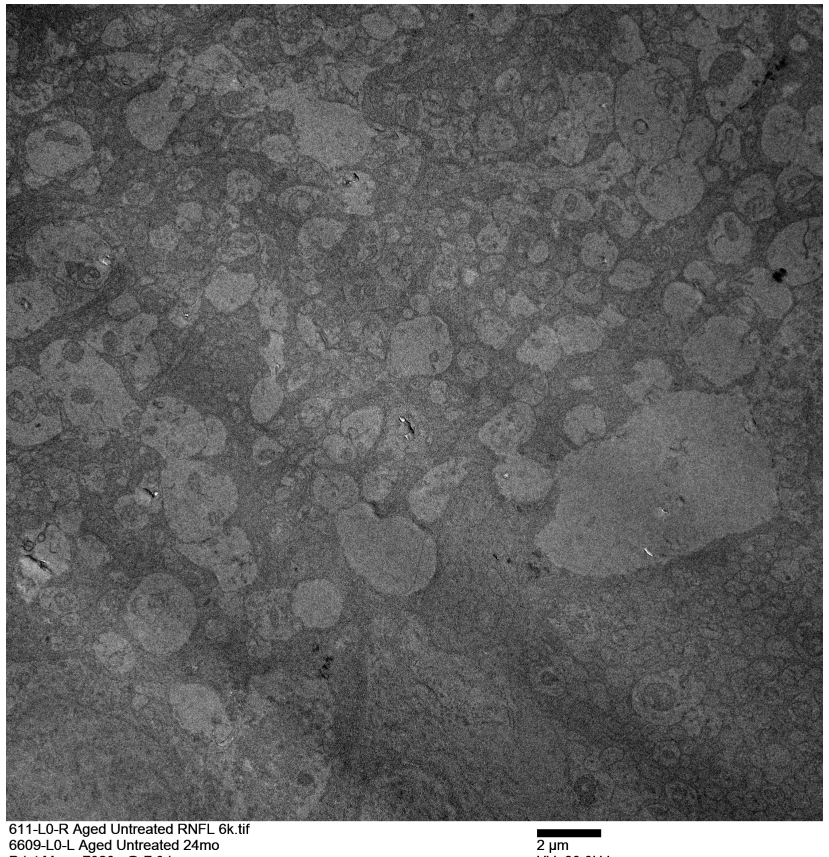

611-L0-R Aged Untreated RNFL 6k.tif
6609-L0-L Aged Untreated 24mo
Print Mag: 7020x @ 7.0 in
15:26:51 4/13/2023
2 µm
HV=80.0kV
Direct Mag: 6000x
Center for Biologic Imaging

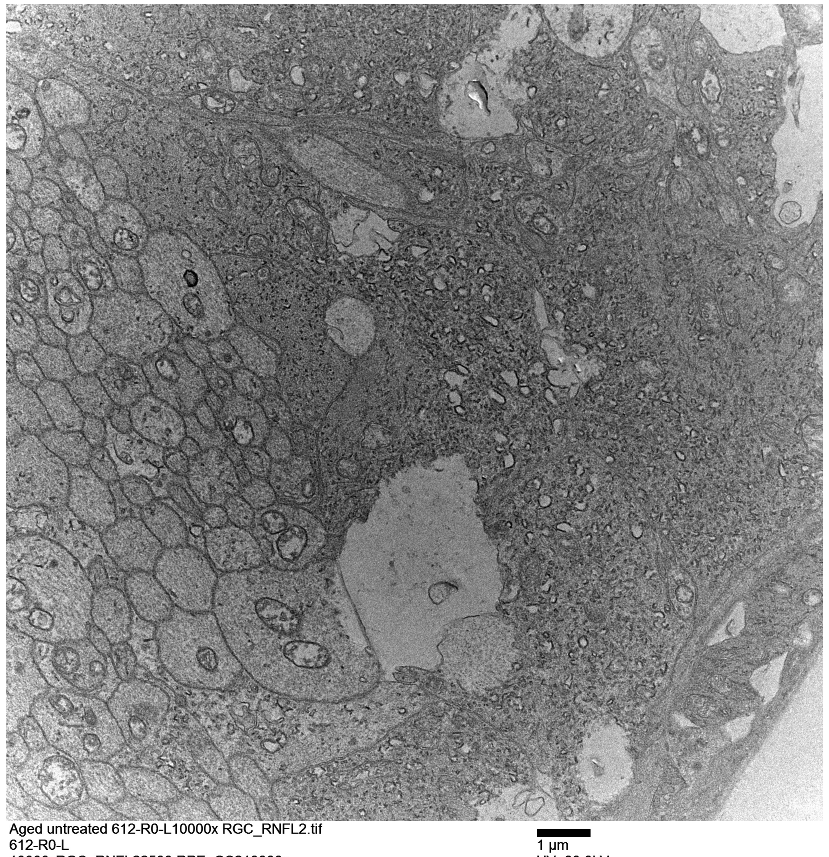

Aged untreated 612-R0-L10000x RGC_RNFL2.tif
612-R0-L
10000xRGC_RNFL22500 RPE+OS210000x
Print Mag: 11700x @ 7.0 in
15:50:10 3/7/2023
1 µm
HV=80.0kV
Direct Mag: 10000x
Center for Biologic Imaging

Aged Untreated RGC-RNFL

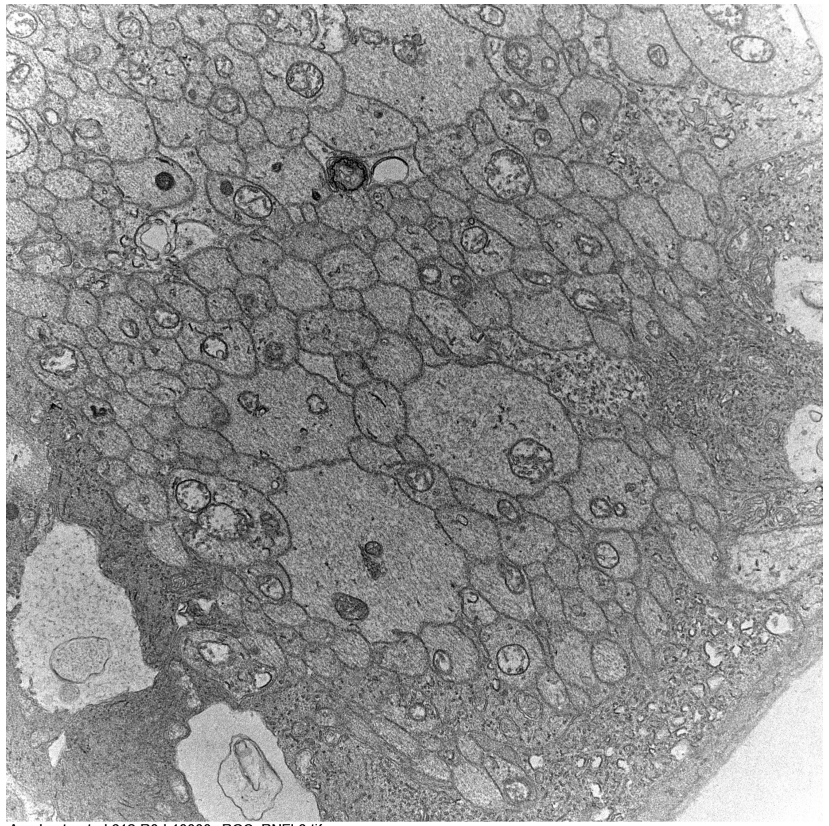

Aged untreated 612-R0-L10000x RGC_RNFL3.tif
612-R0-L
10000xRGC_RNFL32500 RPE+OS210000x
Print Mag: 11700x @ 7.0 in
15:52:56 3/7/2023

1 μm
HV=80.0kV
Direct Mag: 10000x
Center for Biologic Imaging

8-AG treated Aged Untreated Central Retinae

8-AG treated aged central retina-RGC-RNFL

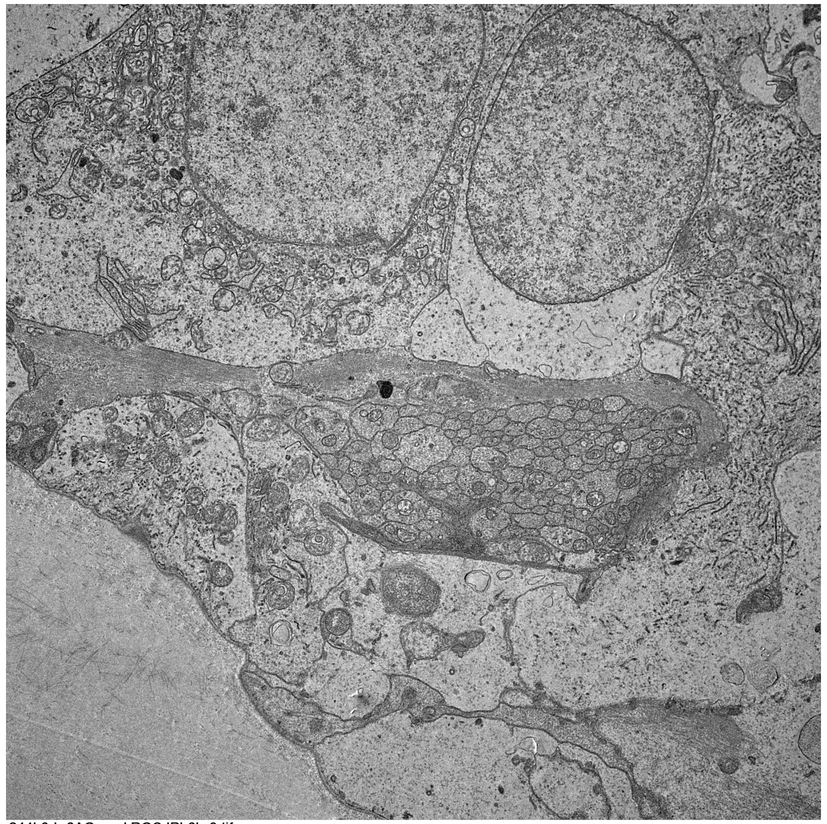

244L0-L-8AGaged RGC IPL6kx3.tif
244-L0-L
aged-8AG treated RNFL RGC 6kx3
Print Mag: 7020x @ 7.0 in

2 µm
HV=80.0kV
Direct Mag: 6000x

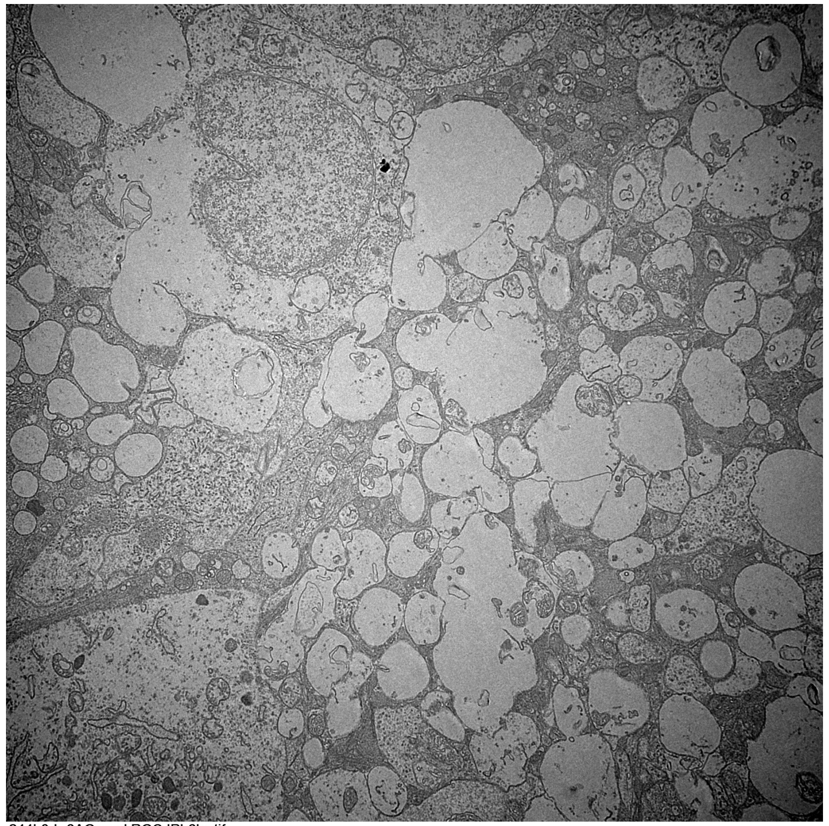

244L0-L-8AGaged RGC IPL6kx.tif
244-L0-L
aged-8AG treated RGC IPL 6kx
Print Mag: 7020x @ 7.0 in

2 µm
HV=80.0kV
Direct Mag: 6000x

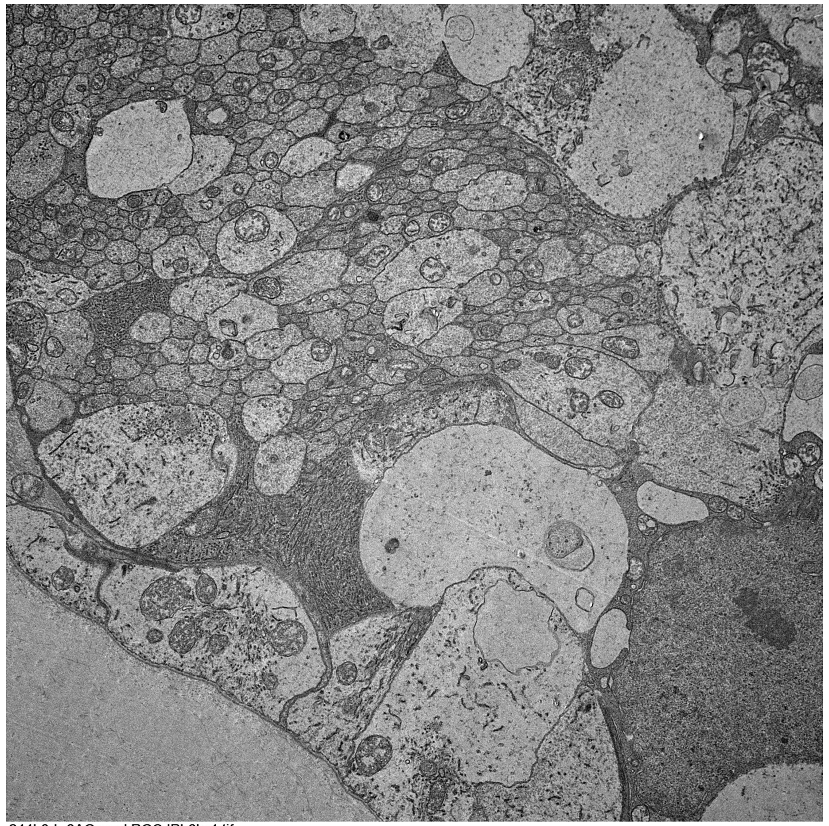

244L0-L-8AGaged RGC IPL6kx4.tif
244-L0-L
aged-8AG treated RNFL RGC 6kx4
Print Mag: 7020x @ 7.0 in

2 µm
HV=80.0kV
Direct Mag: 6000x

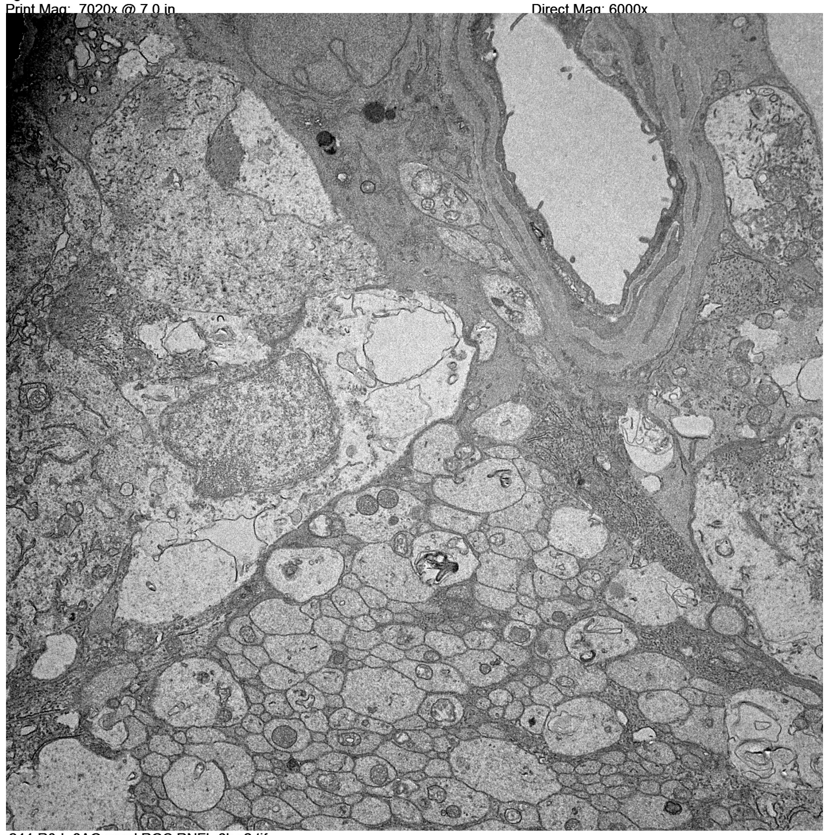

244-R0-L 8AG aged RGC RNFL 6k x2.tif
244-L0-L
aged-8AG treated RGC RNFL 6kx2
Print Mag: 7020x @ 7.0 in
14:47:04 3/29/2023

2 µm
HV=80.0kV
Direct Mag: 6000x
Center for Biologic Imaging

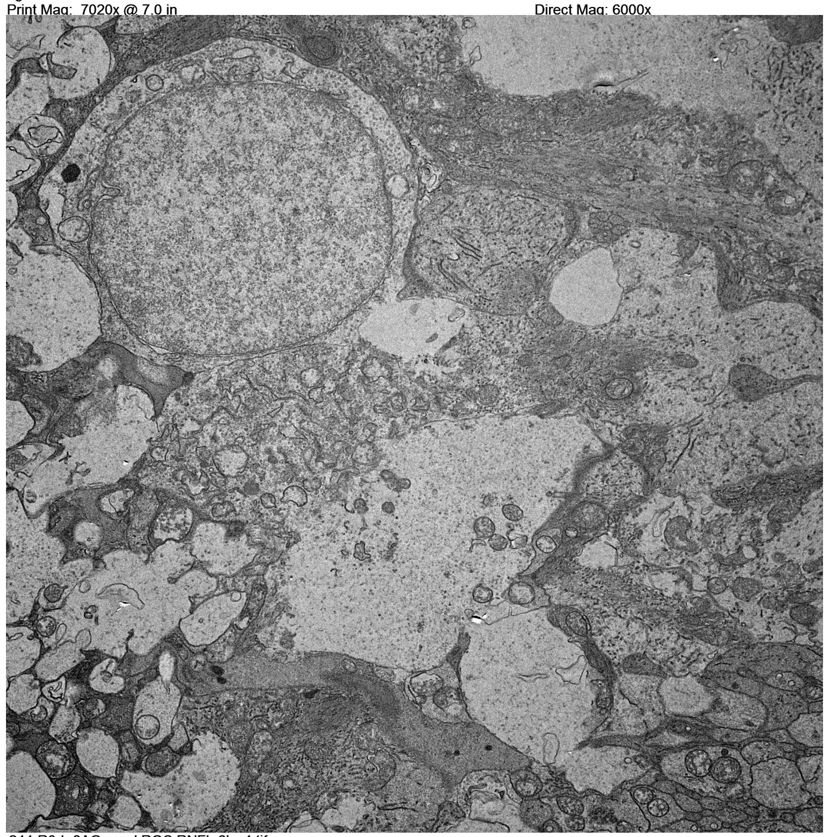

244-R0-L 8AG aged RGC RNFL 6k x4.tif
244-L0-L
aged-8AG treated RGC RNFL 6kx4
Print Mag: 7020x @ 7.0 in
14:53:25 3/29/2023

2 µm
HV=80.0kV
Direct Mag: 6000x
Center for Biologic Imaging

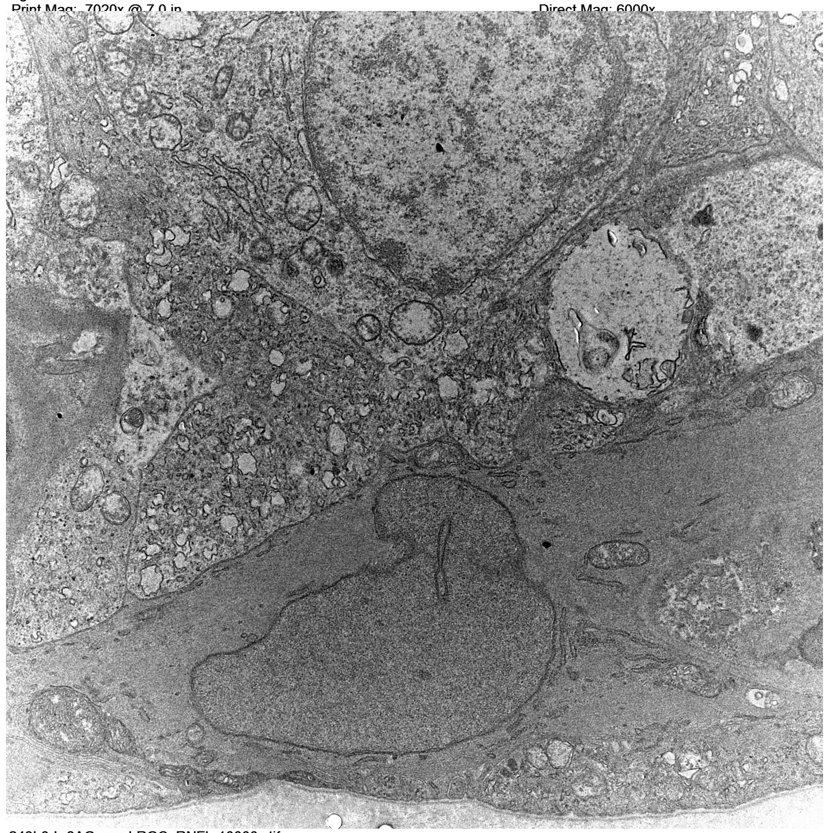

243L0-L-8AG-aged-RGC_RNFL-10000x.tif
243L0-L aged 8AG treated
RGC-RNFL10000x
Print Mag: 11700x @ 7.0 in
14:47:19 3/8/2023

1 µm
HV=80.0kV
Direct Mag: 10000x
Center for Biologic Imaging

8-AG treated aged central retina- RGC-RNFL

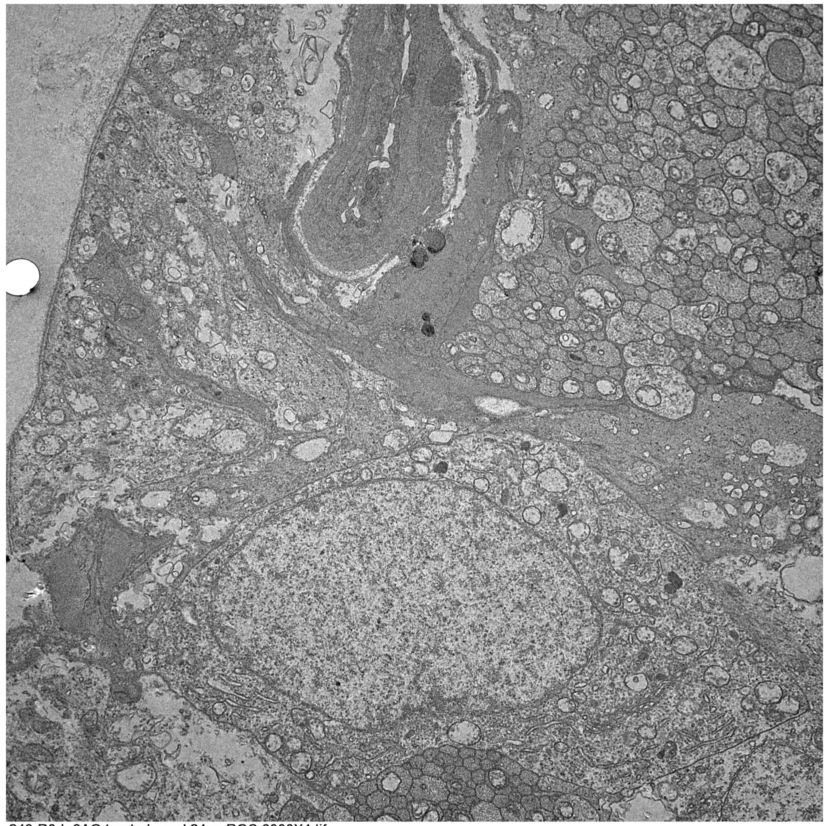

243-R0-L 8AG treated aged 24 m RGC 6000X4.tif
243-L0-L_8AG treated Aged
RGC 6000X4
Print Mag: 7020x @ 7.0 in
15:49:00 3/9/2023

2 µm
HV=80.0kV
Direct Mag: 6000x
Center for Biologic Imaging

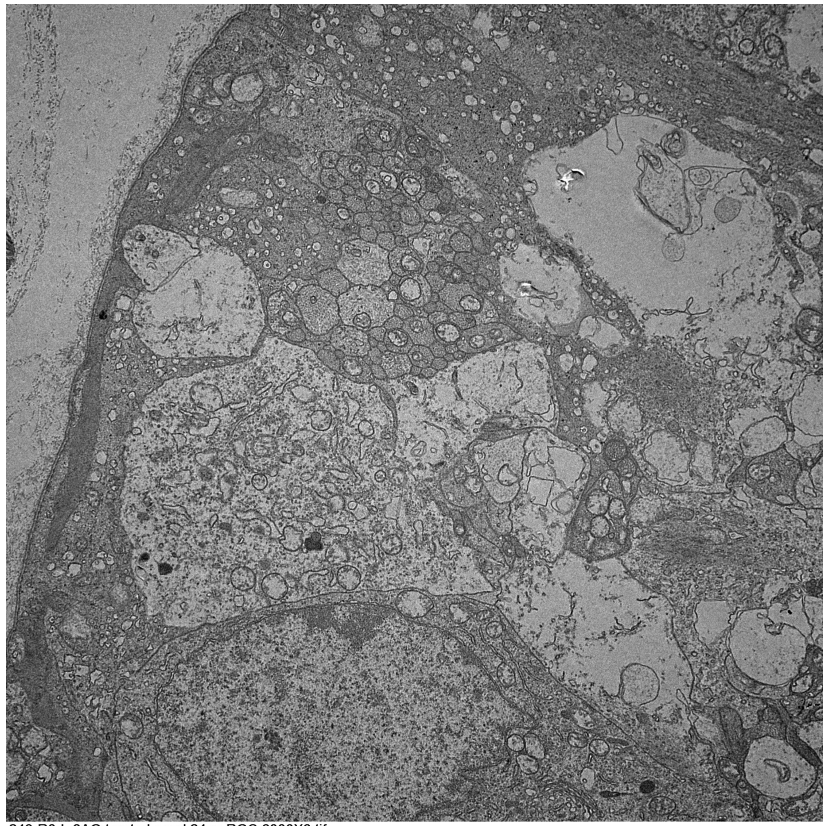

243-R0-L 8AG treated aged 24 m RGC 6000X6.tif
243-L0-L_8AG treated Aged
RGC 6000X6
Print Mag: 7020x @ 7.0 in
15:53:26 3/9/2023

2 µm
HV=80.0kV
Direct Mag: 6000x
Center for Biologic Imaging

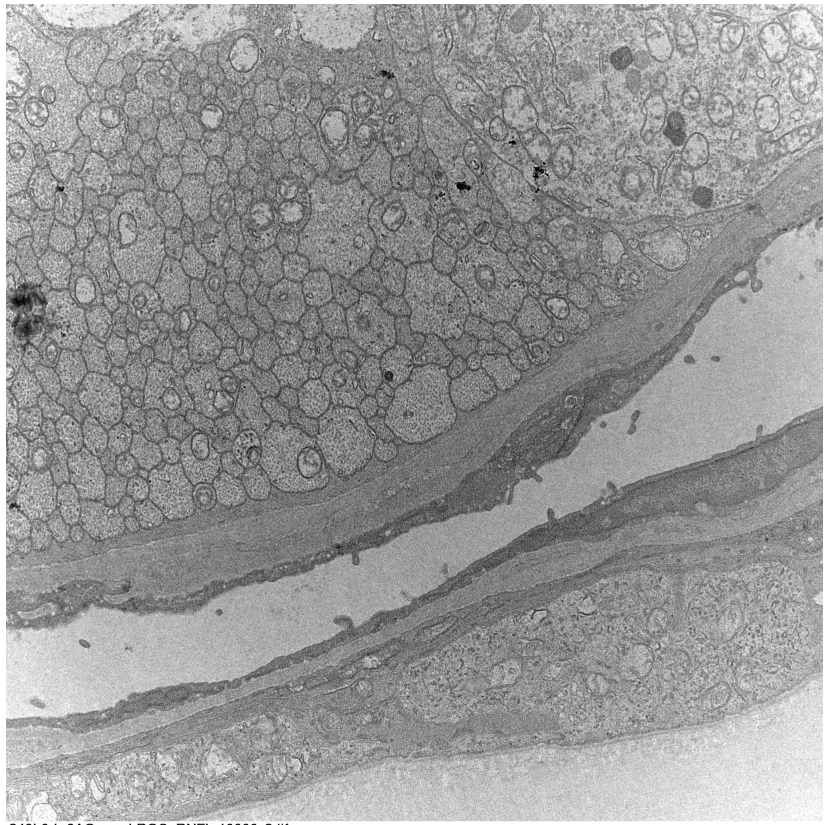

243L0-L-8AG-aged-RGC_RNFL-10000x2.tif
243L0-L aged 8AG treated
RGC-RNFL10000x2
Print Mag: 11700x @ 7.0 in
14:51:53 3/8/2023

1 µm
HV=80.0kV
Direct Mag: 10000x
Center for Biologic Imaging

8-AG treated aged central retina-IPL

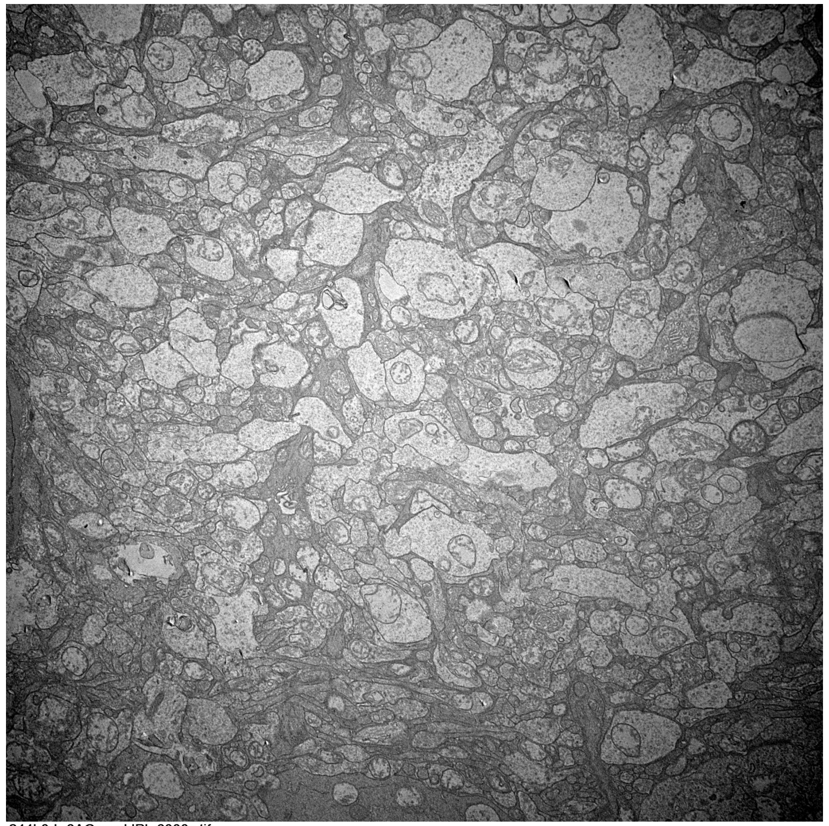

244L0-L-8AGaged IPL 6000x.tif
244-L0-L
aged-8AG treated IPL 6kx
Print Mag: 7020x @ 7.0 in
13:13:18 3/29/2023

2 µm
HV=80.0kV
Direct Mag: 6000x
Center for Biologic Imaging

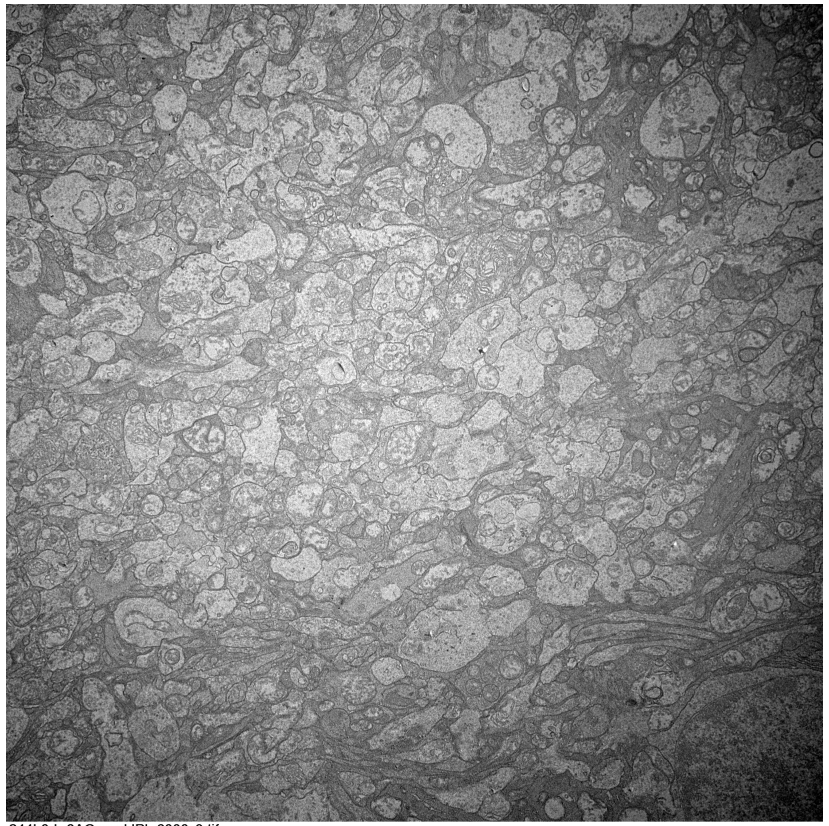

244L0-L-8AGaged IPL 6000x3.tif
244-L0-L
aged-8AG treated IPL 6kx3
Print Mag: 7020x @ 7.0 in
13:19:44 3/29/2023

2 µm
HV=80.0kV
Direct Mag: 6000x
Center for Biologic Imaging

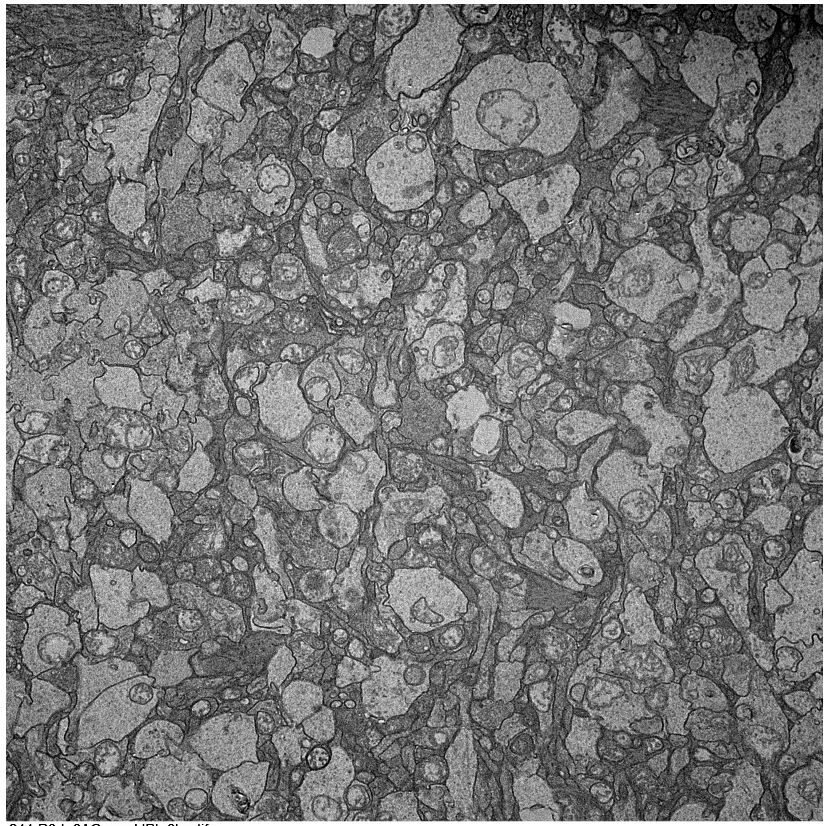

244-R0-L 8AG aged IPL 6k x.tif
244-L0-L
aged-8AG treated IPL 6kx
Print Mag: 7020x @ 7.0 in
16:55:55 3/29/2023

2 µm
HV=80.0kV
Direct Mag: 6000x
Center for Biologic Imaging

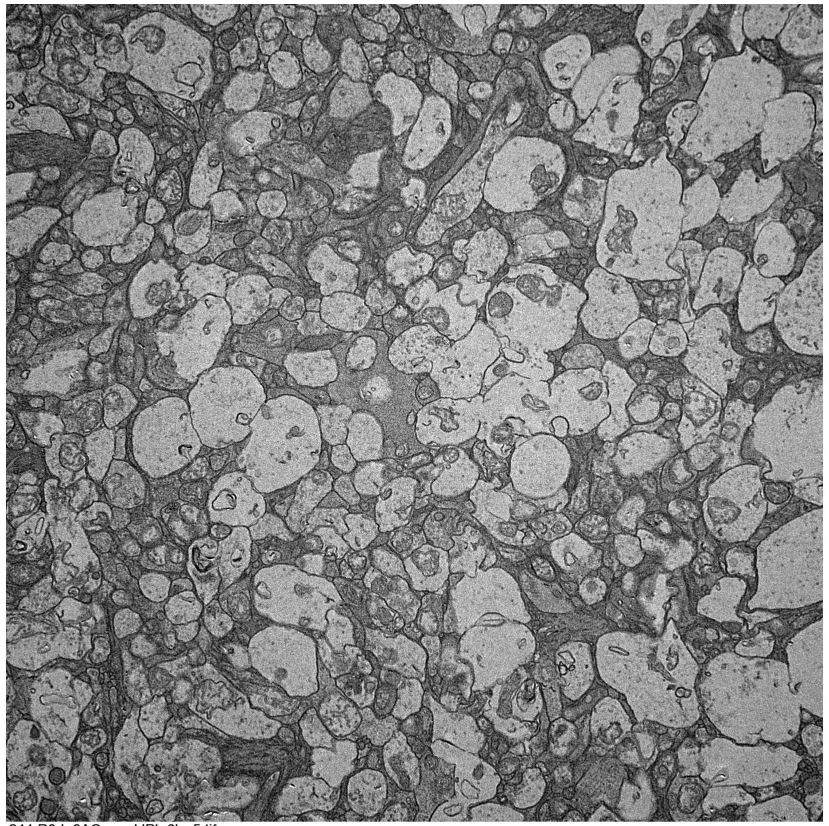

244-R0-L 8AG aged IPL 6k x5.tif
244-L0-L
aged-8AG treated IPL 6kx 5
Print Mag: 7020x @ 7.0 in
17:00:56 3/29/2023

2 µm
HV=80.0kV
Direct Mag: 6000x
Center for Biologic Imaging

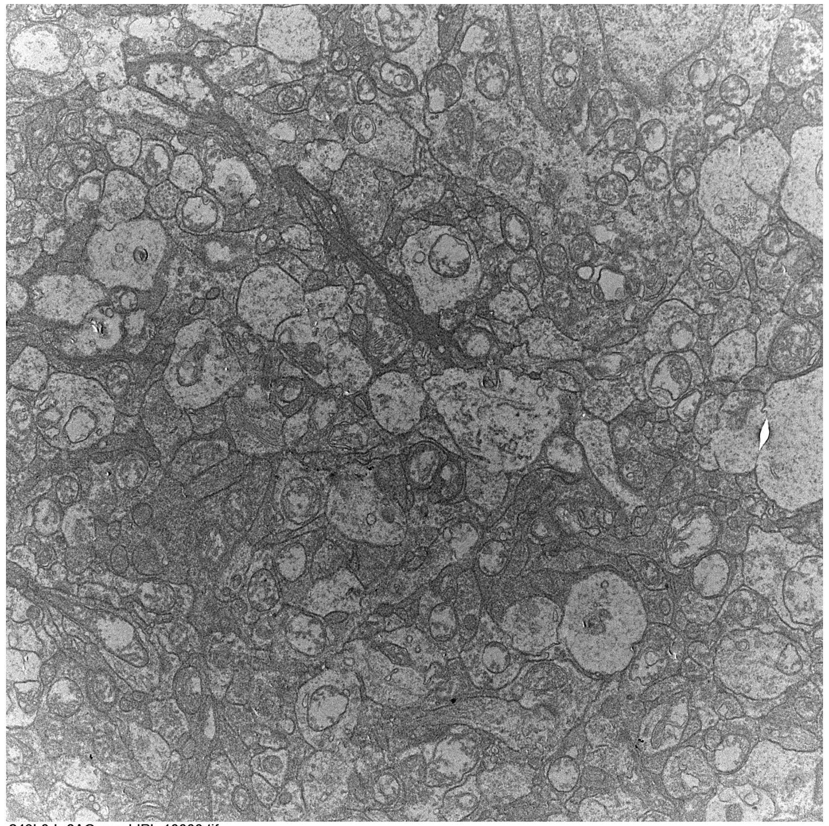

243L0-L-8AG-aged-IPL-10000.tif
243L0-L aged 8AG treated
IPL10000x
Print Mag: 11700x @ 7.0 in
15:13:54 3/8/2023

1 µm
HV=80.0kV
Direct Mag: 10000x
Center for Biologic Imaging

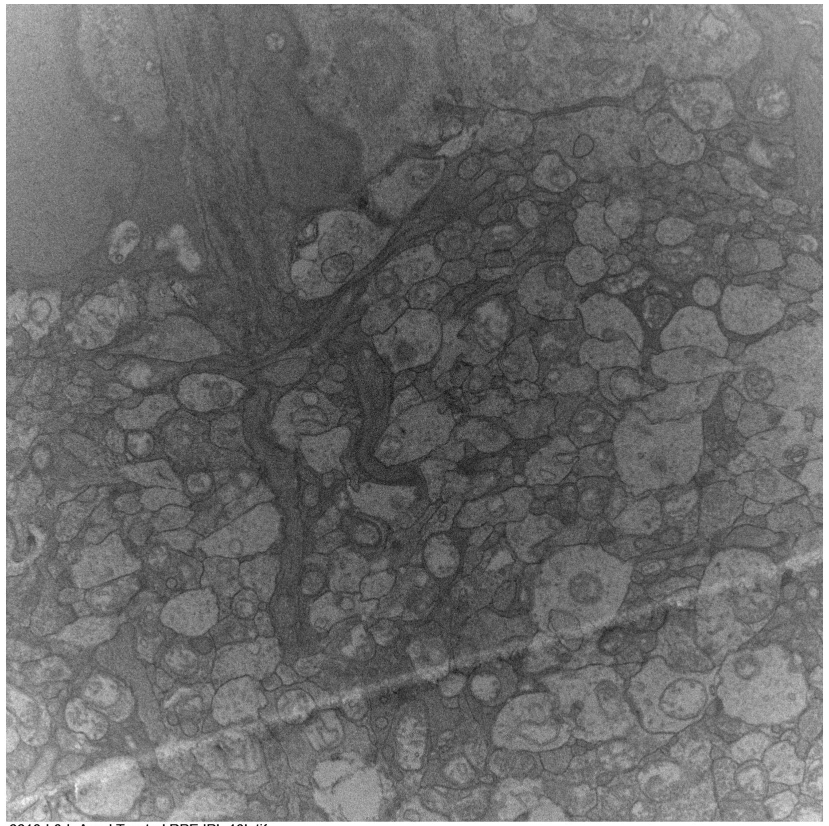

6610-L0-L Aged Treated RPE IPL 10k.tif
611-R0-R Aged Untreated
Print Mag: 11700x @ 7.0 in
12:56:58 4/27/2023

1 µm
HV=80.0kV
Direct Mag: 10000x
Center for Biologic Imaging

8-AG treated aged central retina-IPL

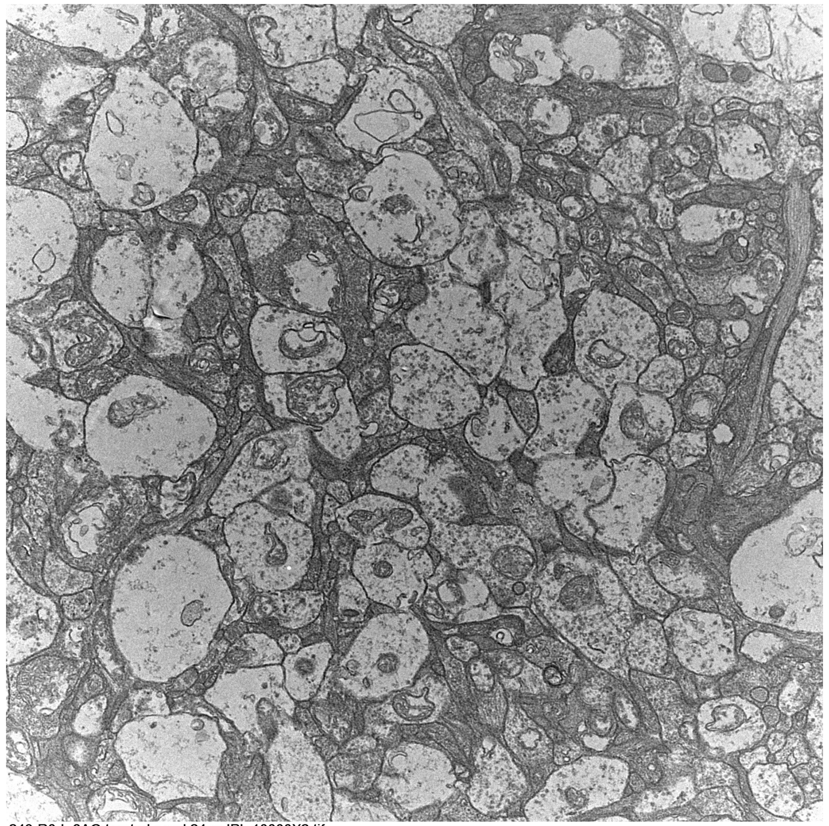

243-R0-L 8AG treated aged 24 m IPL 10000X2.tif
243-L0-L_8AG treated Aged
IPL 10000X2
Print Mag: 11700x @ 7.0 in
15:34:39 3/9/2023

1 μm
HV=80.0kV
Direct Mag: 10000x
Center for Biologic Imaging

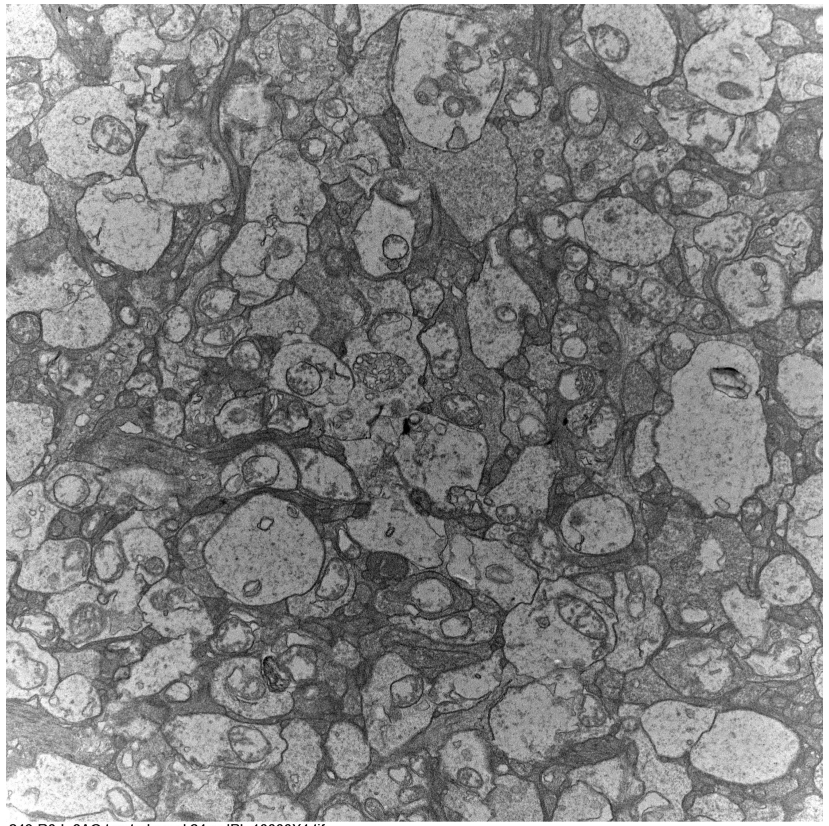

243-R0-L 8AG treated aged 24 m IPL 10000X4.tif
243-L0-L_8AG treated Aged
IPL 10000X3
Print Mag: 11700x @ 7.0 in
15:59:21 3/9/2023

1 μm
HV=80.0kV
Direct Mag: 10000x
Center for Biologic Imaging

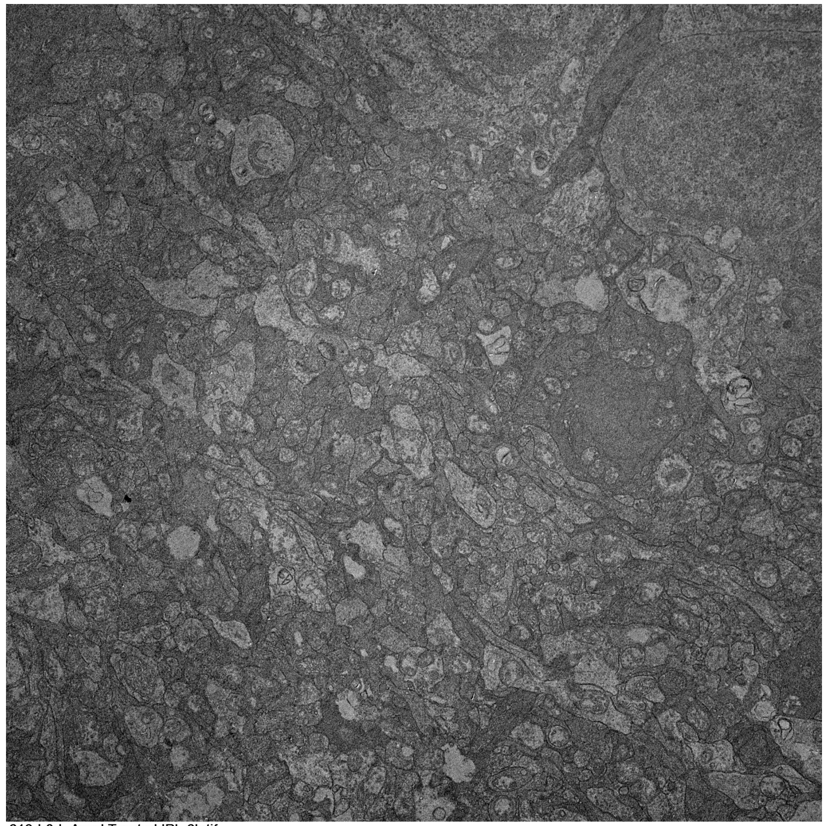

613-L0-L Aged Treated IPL 6k.tif
613-L0-L Aged Treated
Print Mag: 7020x @ 7.0 in
14:36:50 4/28/2023

2 μm
HV=80.0kV
Direct Mag: 6000x
Center for Biologic Imaging

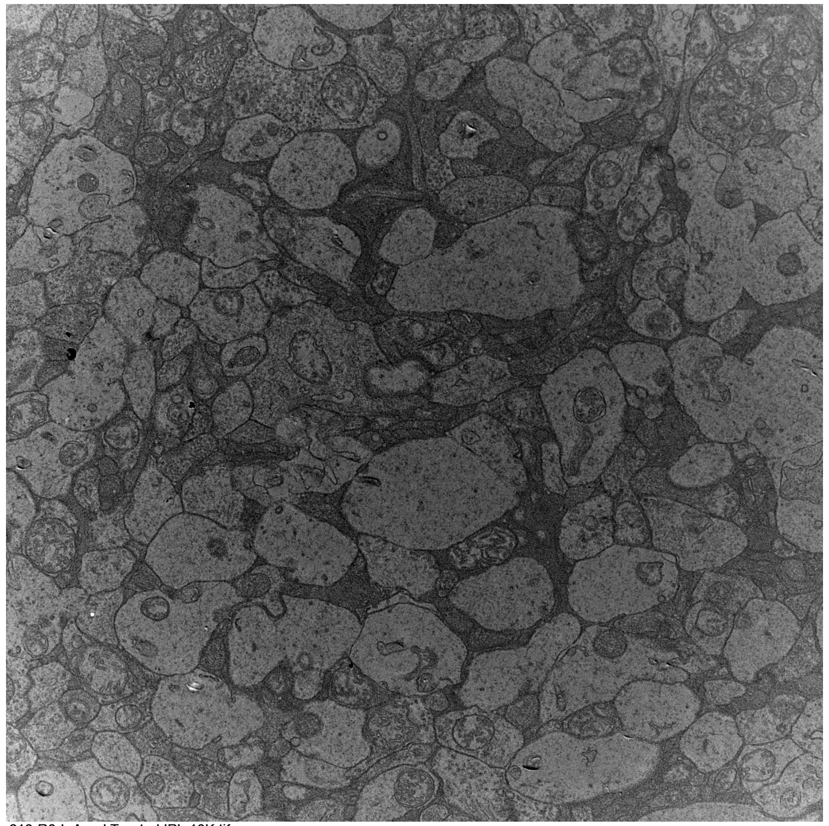

613-R0-L Aged Treated IPL 10K.tif
613-R0-L Aged Treated
Print Mag: 11700x @ 7.0 in
15:28:31 4/28/2023

1 μm
HV=80.0kV
Direct Mag: 10000x
Center for Biologic Imaging

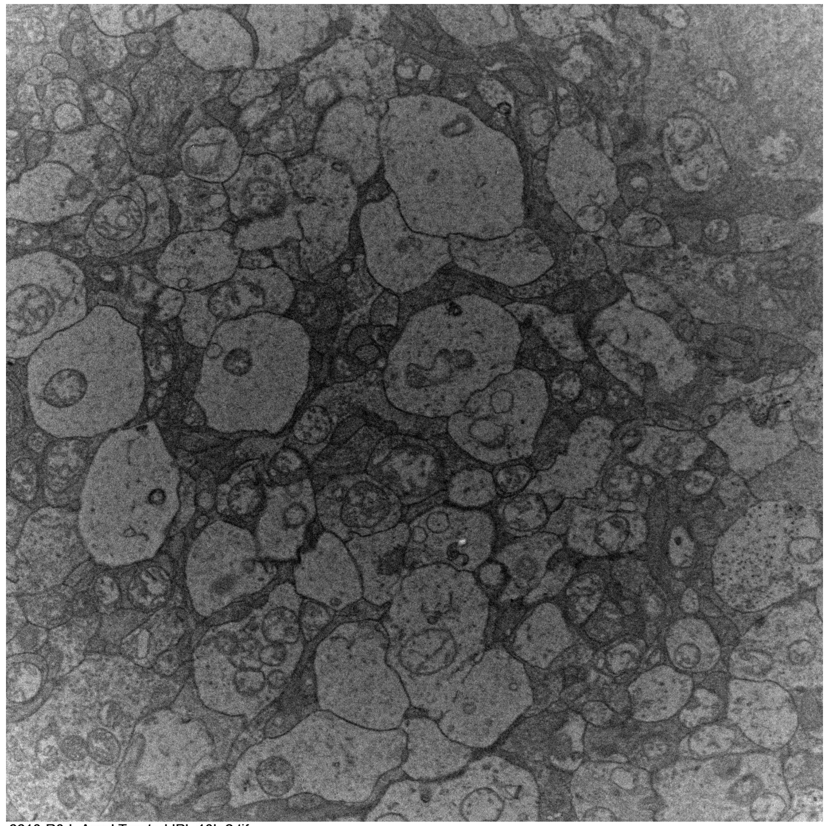

6610-R0-L Aged Treated IPL 10k-2.tif
6610-R0-L Aged Treated
Print Mag: 11700x @ 7.0 in
13:42:55 4/28/2023

1 μm
HV=80.0kV
Direct Mag: 10000x
Center for Biologic Imaging

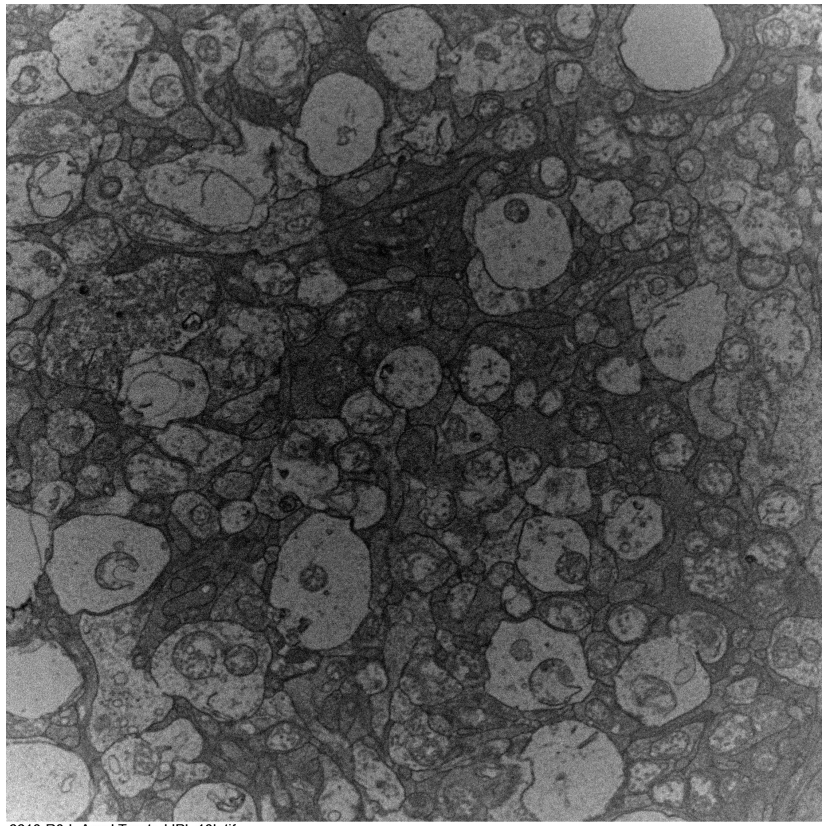

6610-R0-L Aged Treated IPL 10k.tif
6610-R0-L Aged Treated
Print Mag: 11700x @ 7.0 in
13:30:19 4/28/2023

1 μm
HV=80.0kV
Direct Mag: 10000x
Center for Biologic Imaging

8-AG treated aged central retina-INL

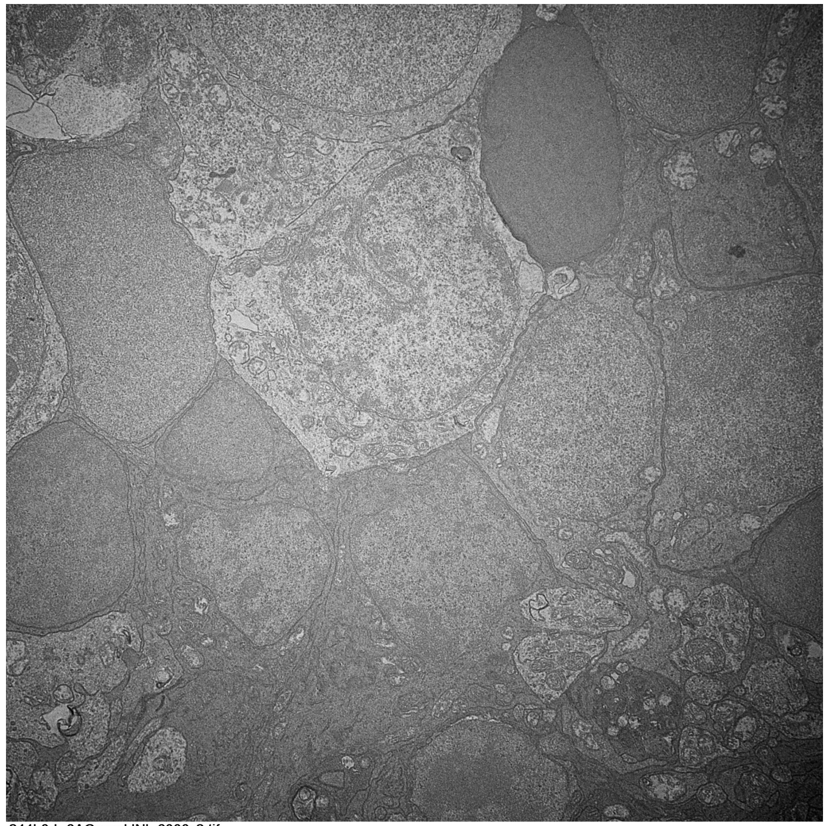

244L0-L-8AGaged INL 6000x2.tif
244-L0-L
aged-8AG treated INL 6kx2
Print Mag: 7020x @ 7.0 in
13:26:03 3/29/2023

2 µm
HV=80.0kV
Direct Mag: 6000x
Center for Biologic Imaging

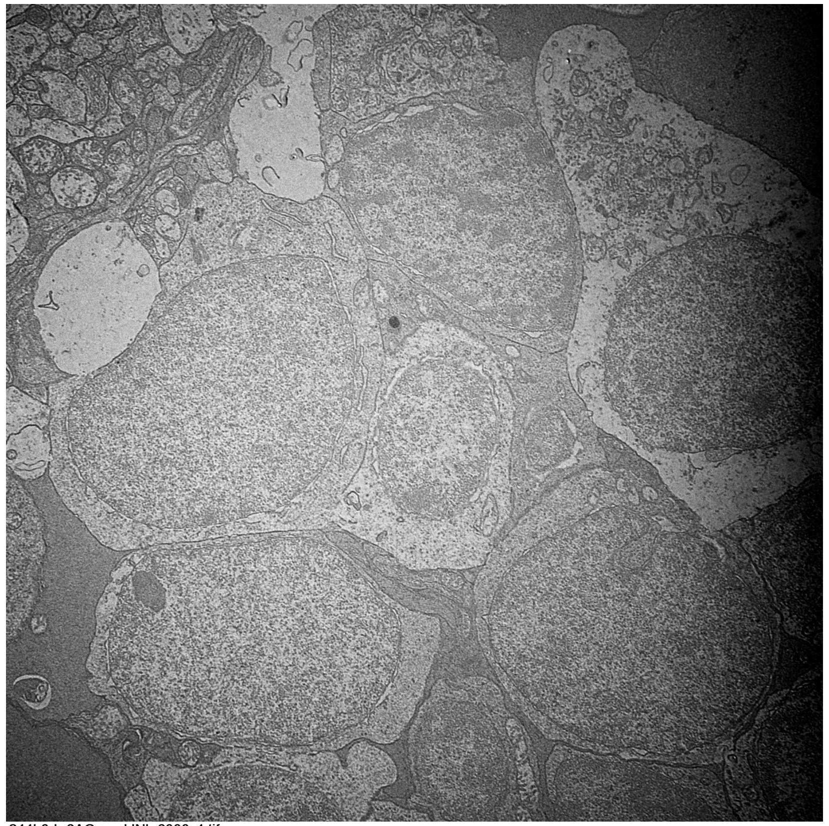

244L0-L-8AGaged INL 6000x4.tif
244-L0-L
aged-8AG treated INL 6kx3
Print Mag: 7020x @ 7.0 in
13:50:47 3/29/2023

2 µm
HV=80.0kV
Direct Mag: 6000x
Center for Biologic Imaging

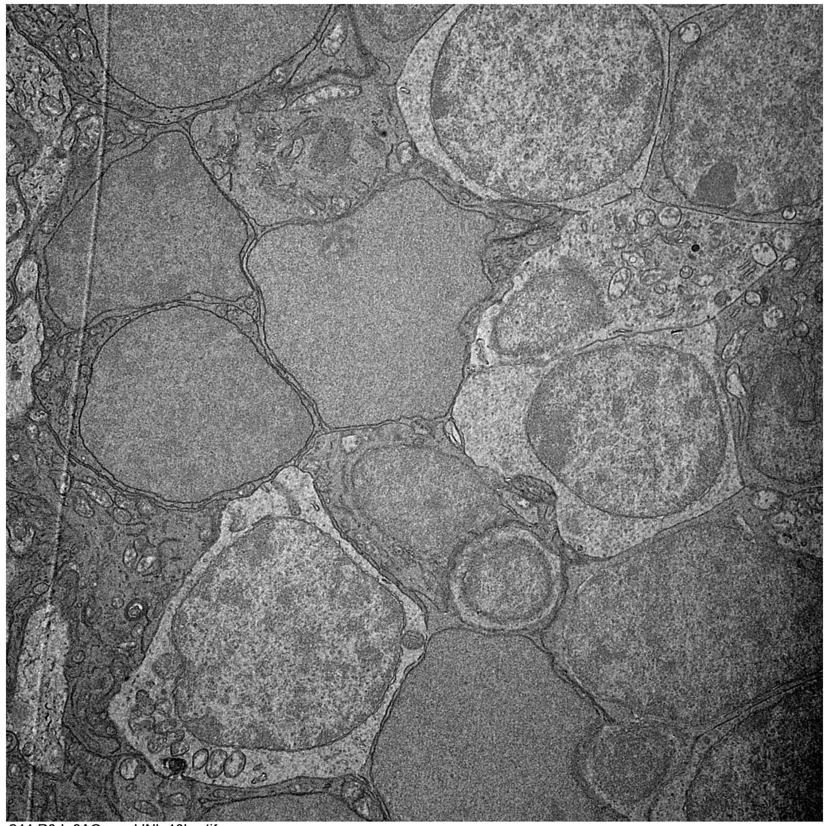

244-R0-L 8AG aged INL 10k x.tif
244-L0-L
aged-8AG treated INL10kx
Print Mag: 7020x @ 7.0 in
16:45:45 3/29/2023

2 µm
HV=80.0kV
Direct Mag: 6000x
Center for Biologic Imaging

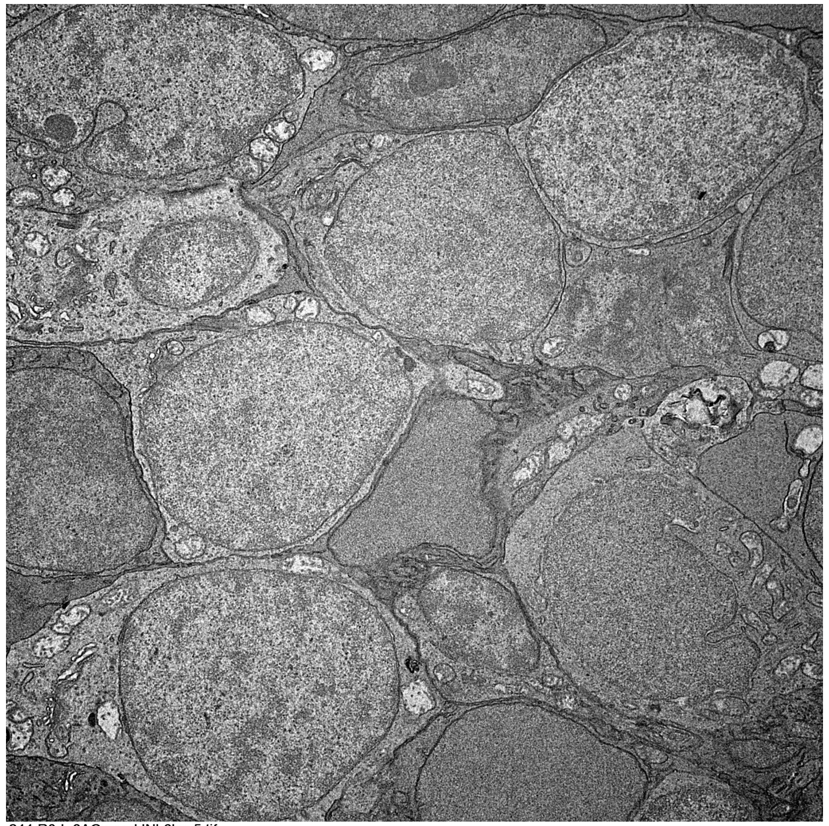

244-R0-L 8AG aged INL6k x 5.tif
244-L0-L
aged-8AG treated INL6kx4
Print Mag: 7020x @ 7.0 in
16:53:21 3/29/2023

2 µm
HV=80.0kV
Direct Mag: 6000x
Center for Biologic Imaging

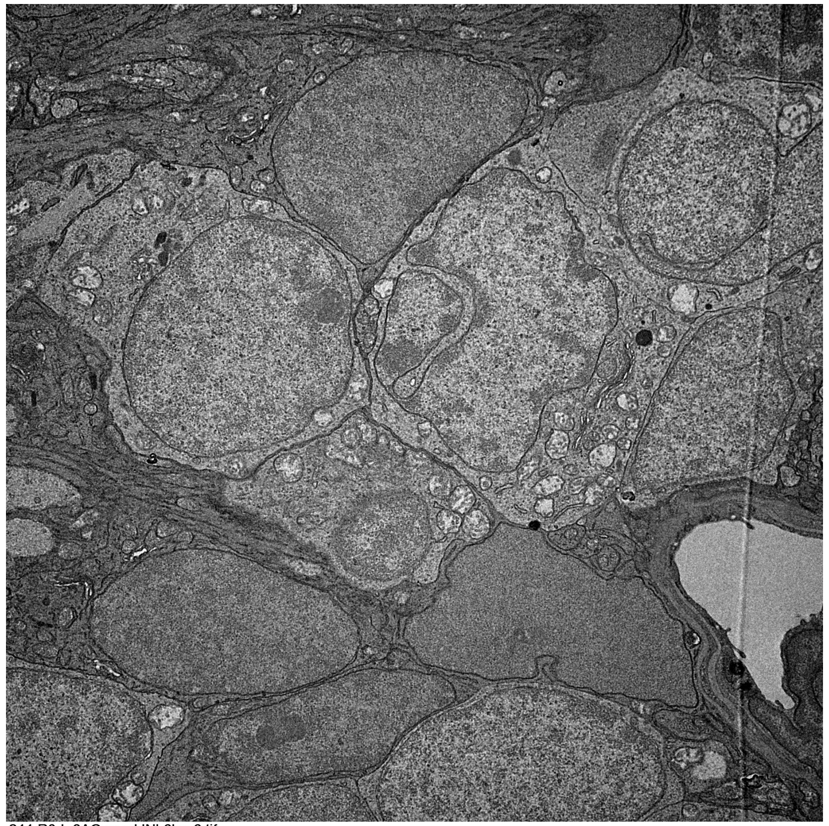

244-R0-L 8AG aged INL6k x 6.tif
244-L0-L
aged-8AG treated INL6kx5
Print Mag: 7020x @ 7.0 in
16:54:46 3/29/2023

2 µm
HV=80.0kV
Direct Mag: 6000x
Center for Biologic Imaging

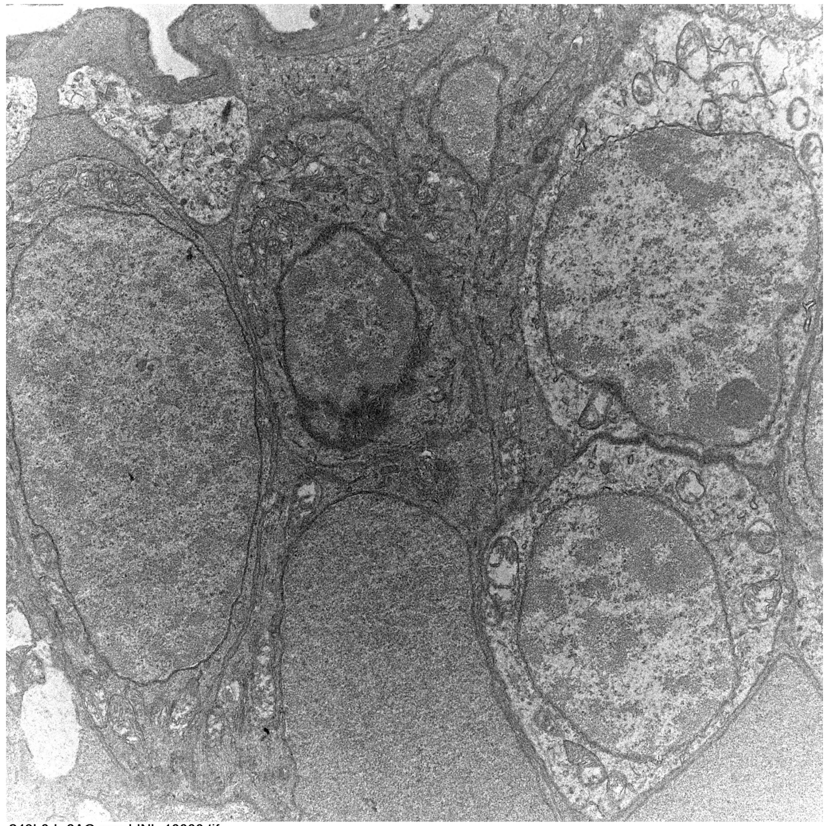

243L0-L-8AG-aged-INL-10000.tif
243L0-L Aged treated
INL10000
Print Mag: 11700x @ 7.0 in
13:45:28 3/8/2023

1 µm
HV=80.0kV
Direct Mag: 10000x
Center for Biologic Imaging

8-AG treated aged central retina-INL

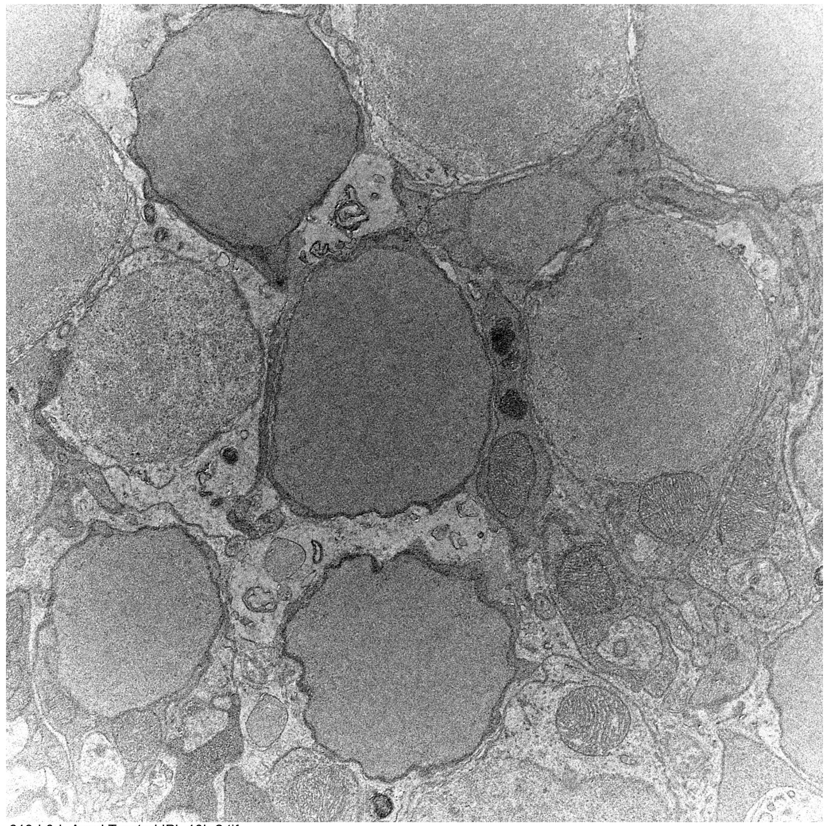

613-L0-L Aged Treated IPL 10k-2.tif
613-L0-L Aged Treated
Print Mag: 11700x @ 7.0 in
14:11:08 4/28/2023

1 µm
HV=80.0kV
Direct Mag: 10000x
Center for Biologic Imaging

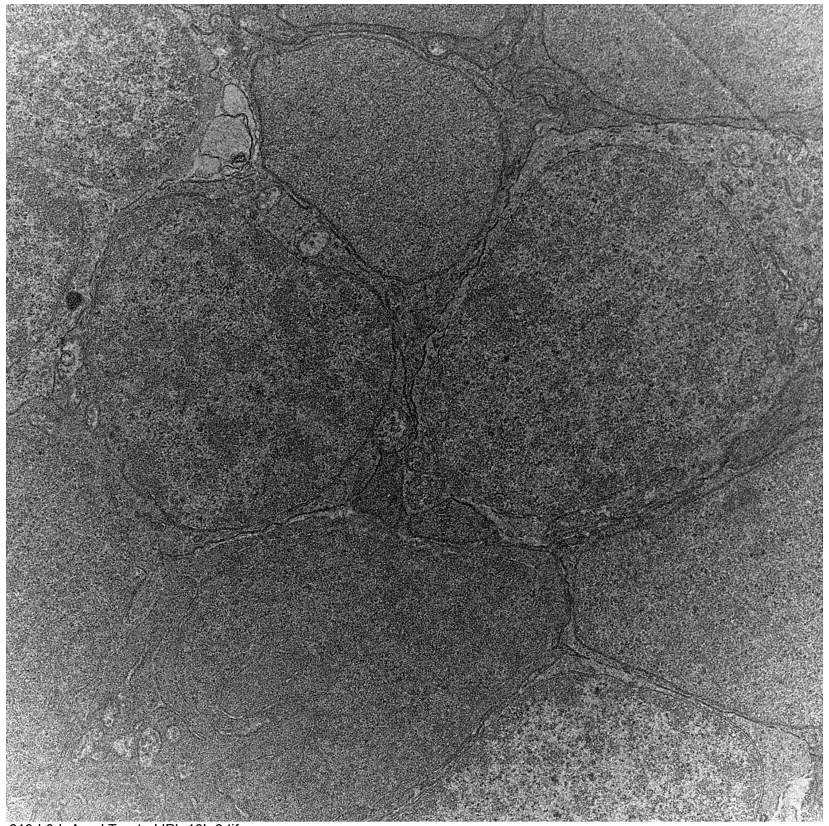

613-L0-L Aged Treated IPL 10k-3.tif
613-L0-L Aged Treated
Print Mag: 11700x @ 7.0 in
14:34:25 4/28/2023

1 µm
HV=80.0kV
Direct Mag: 10000x
Center for Biologic Imaging

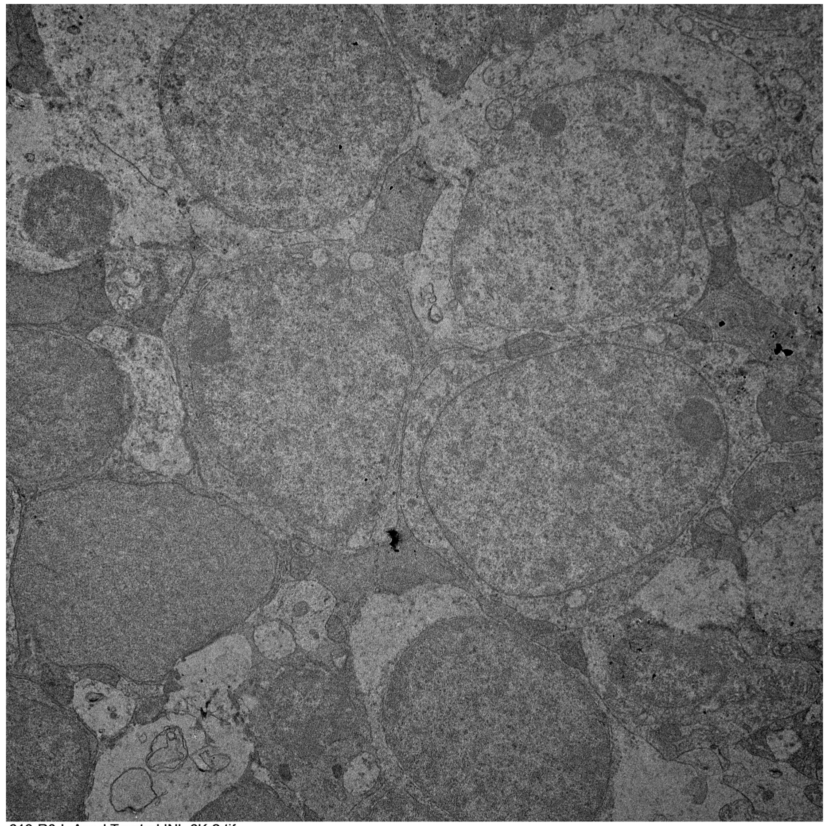

613-R0-L Aged Treated INL 6K-2.tif
613-R0-L Aged Treated
Print Mag: 7020x @ 7.0 in
15:26:58 4/28/2023

2 µm
HV=80.0kV
Direct Mag: 6000x
Center for Biologic Imaging

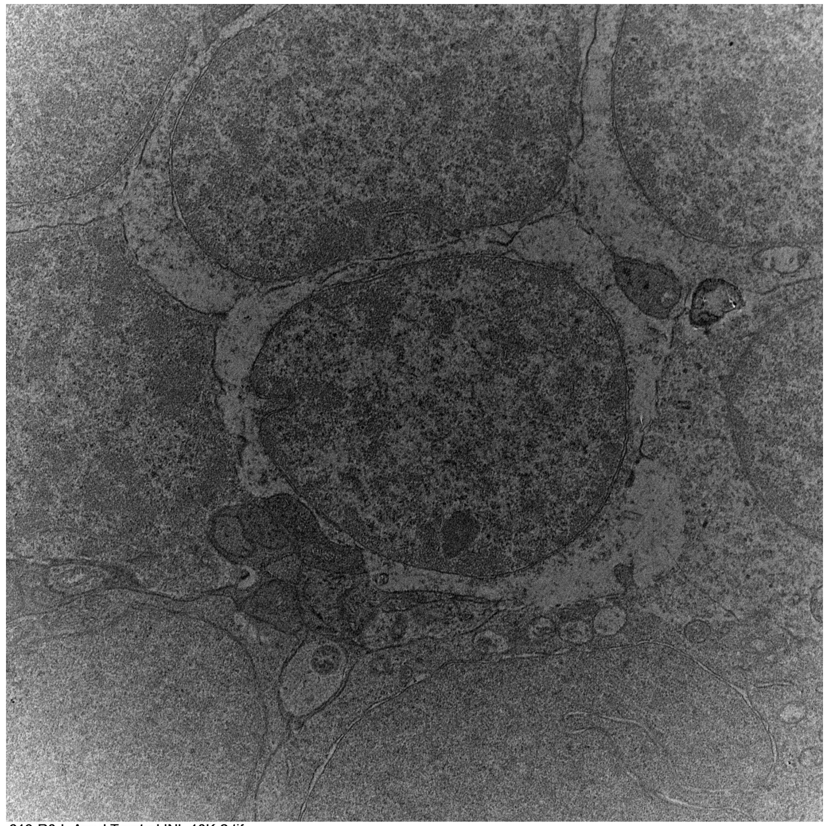

613-R0-L Aged Treated INL 10K-2.tif
613-R0-L Aged Treated
Print Mag: 11700x @ 7.0 in
15:48:11 4/28/2023

1 µm
HV=80.0kV
Direct Mag: 10000x
Center for Biologic Imaging

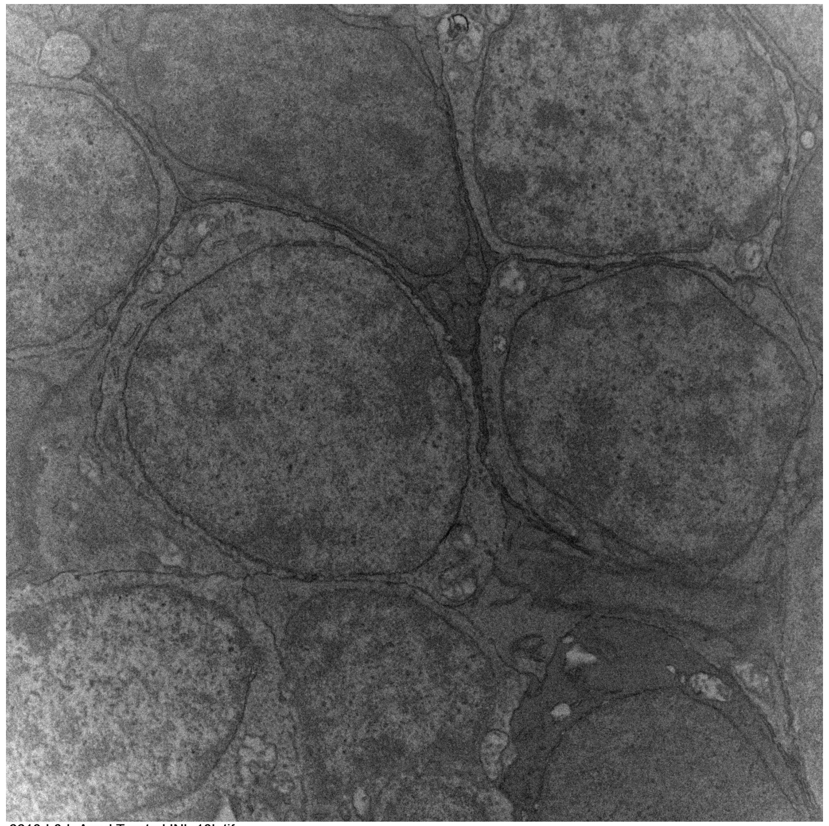

6610-L0-L Aged Treated INL 10k.tif
611-R0-R Aged Untreated
Print Mag: 11700x @ 7.0 in
12:45:14 4/27/2023

1 µm
HV=80.0kV
Direct Mag: 10000x
Center for Biologic Imaging

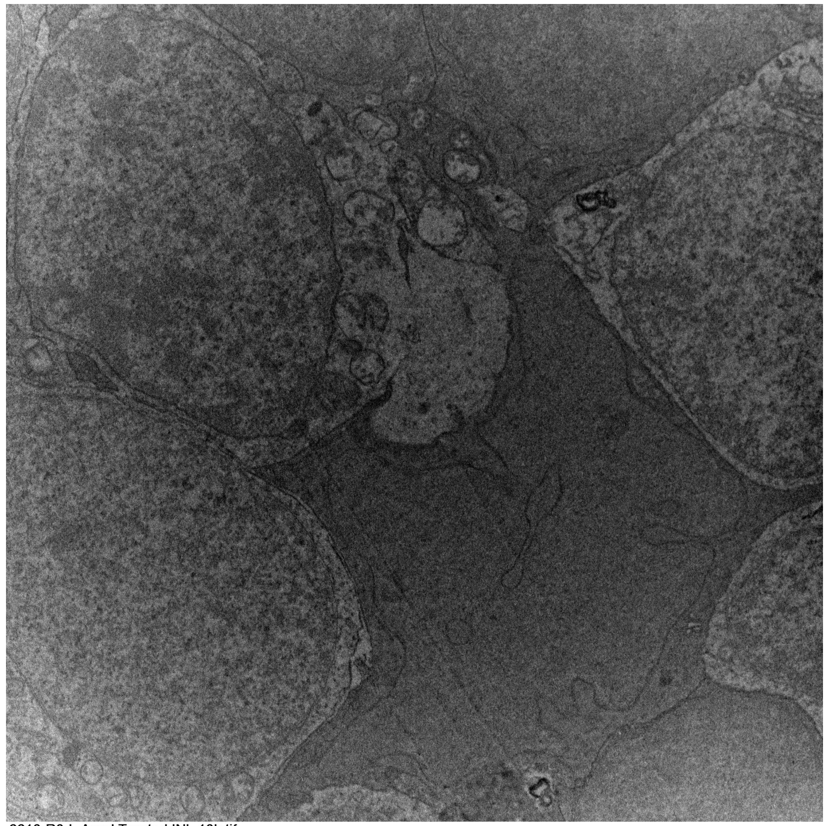

6610-R0-L Aged Treated INL 10k.tif
6610-R0-L Aged Treated
Print Mag: 11700x @ 7.0 in
13:41:47 4/28/2023

1 µm
HV=80.0kV
Direct Mag: 10000x
Center for Biologic Imaging

8-AG treated aged central-INL

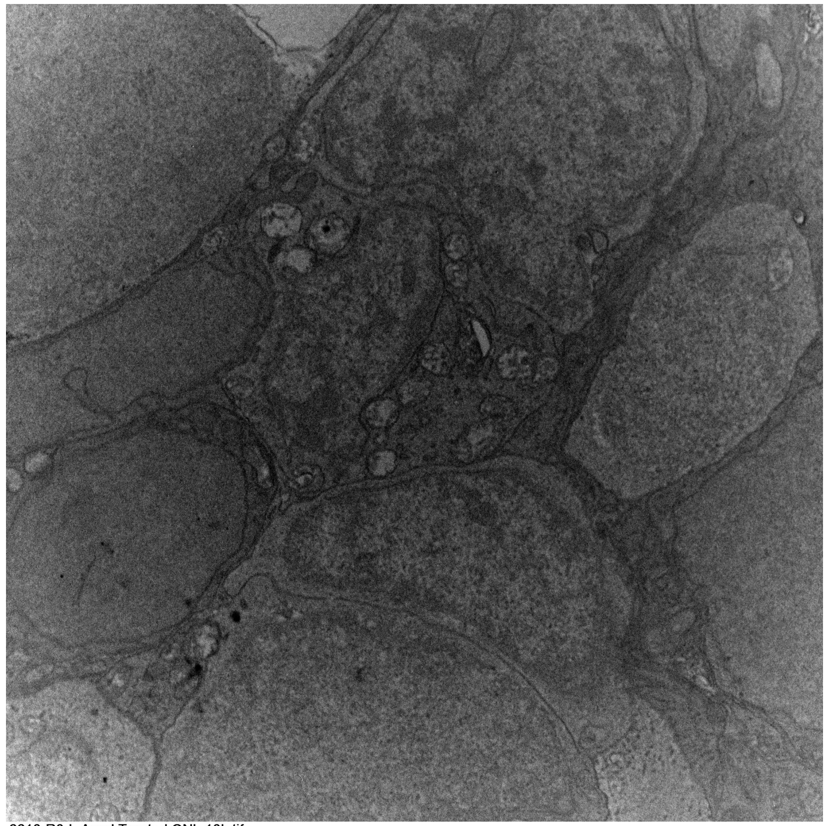

6610-R0-L Aged Treated ONL 10k.tif
6610-R0-L Aged Treated
Print Mag: 11700x @ 7.0 in
13:27:39 4/28/2023

1 µm
HV=80.0kV
Direct Mag: 10000x
Center for Biologic Imaging

8-AG treated aged central retina-ONL

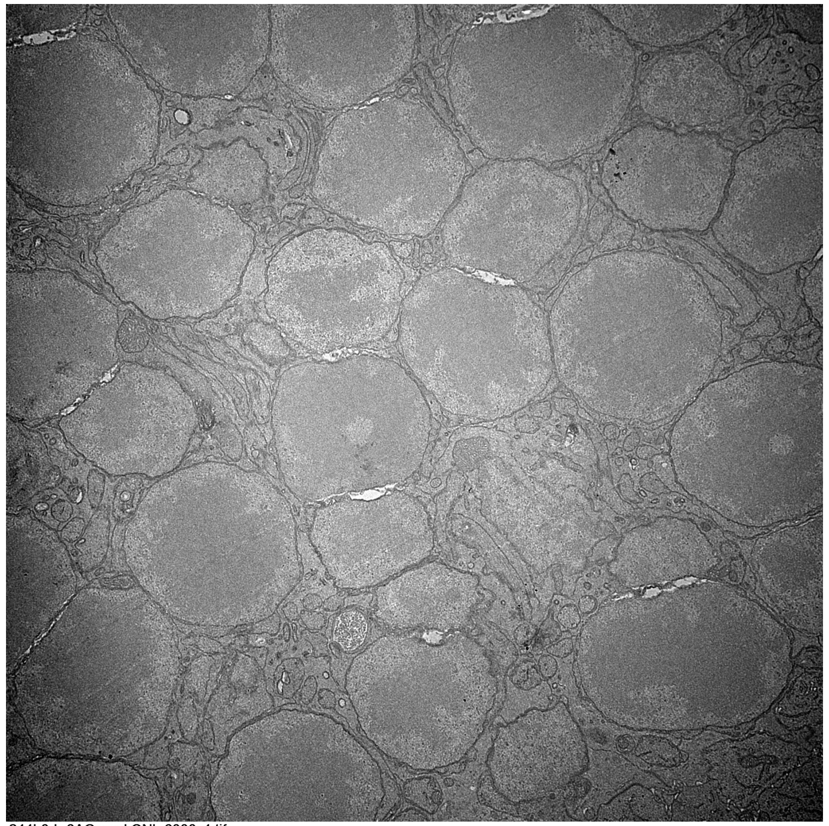

244L0-L-8AGaged ONL 6000x4.tif
244-L0-L
aged-8AG treated ONL 6kx4
Print Mag: 7020x @ 7.0 in
13:33:46 3/29/2023

2 µm
HV=80.0kV
Direct Mag: 6000x
Center for Biologic Imaging

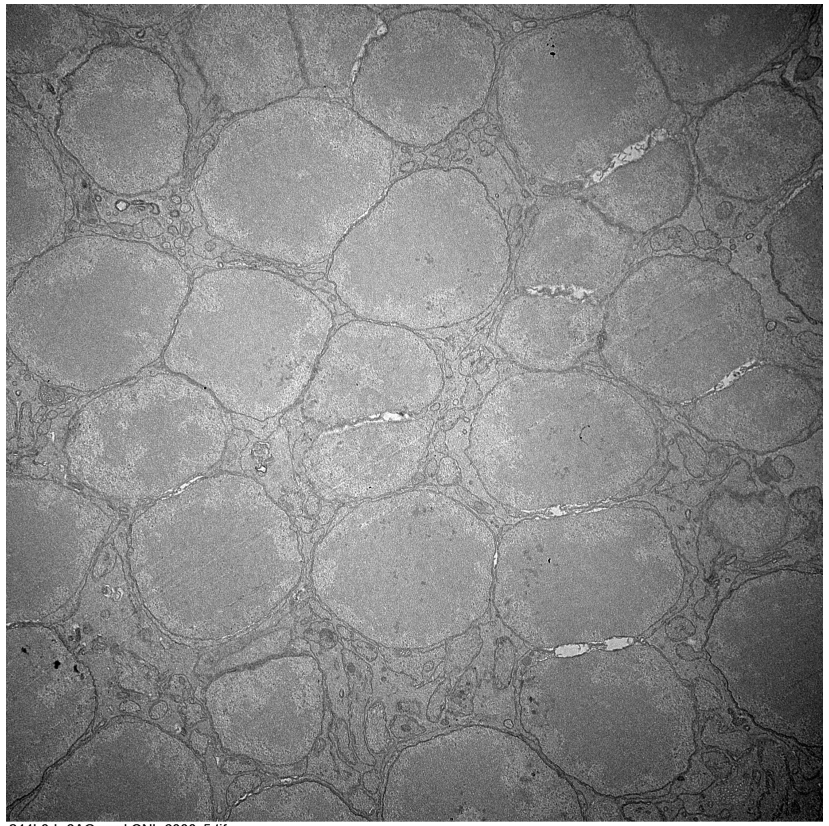

244L0-L-8AGaged ONL 6000x5.tif
244-L0-L
aged-8AG treated ONL 6kx5
Print Mag: 7020x @ 7.0 in
13:35:38 3/29/2023

2 µm
HV=80.0kV
Direct Mag: 6000x
Center for Biologic Imaging

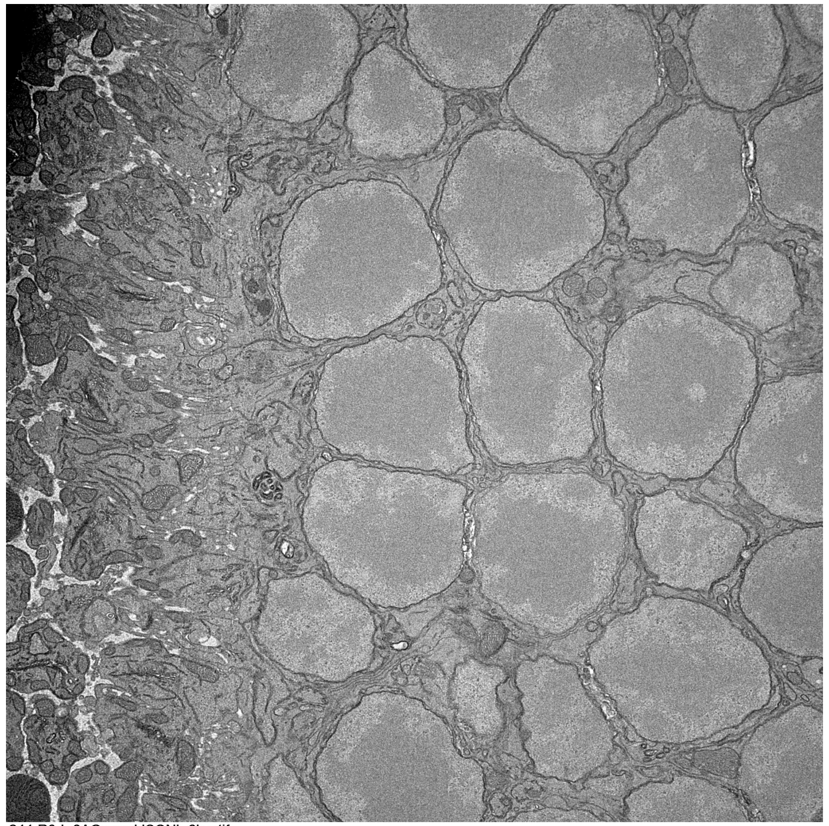

244-R0-L 8AG aged ISONL 6k x.tif
244-L0-L
aged-8AG treated ONL IS 6kx
Print Mag: 7020x @ 7.0 in
16:28:26 3/29/2023

2 µm
HV=80.0kV
Direct Mag: 6000x
Center for Biologic Imaging

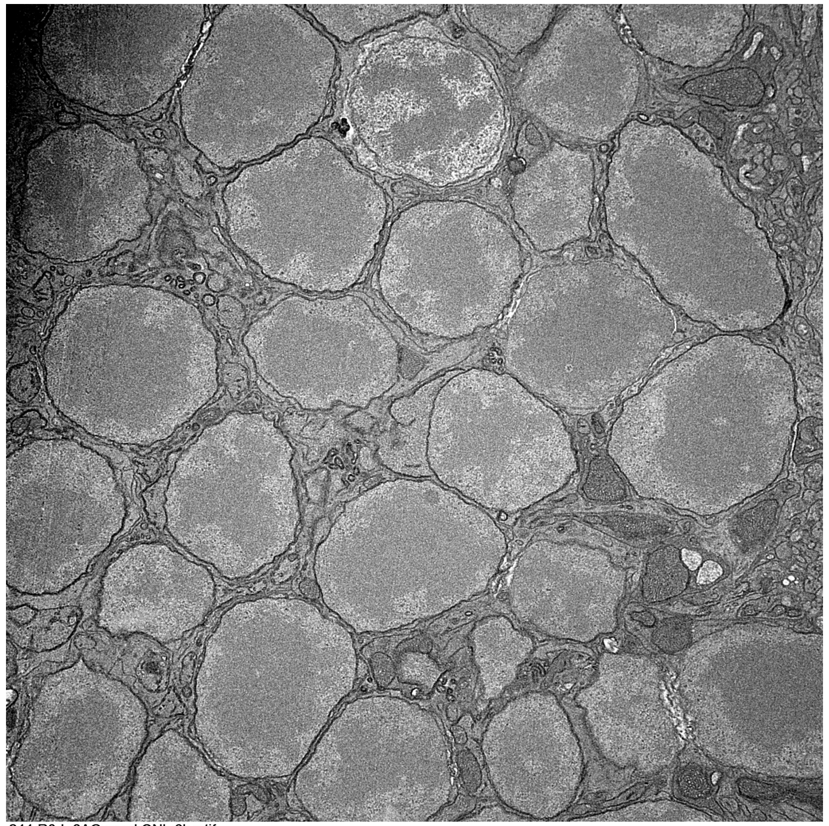

244-R0-L 8AG aged ONL 6k x.tif
244-L0-L
aged-8AG treated ONL 6kx
Print Mag: 7020x @ 7.0 in
16:31:10 3/29/2023

2 µm
HV=80.0kV
Direct Mag: 6000x
Center for Biologic Imaging

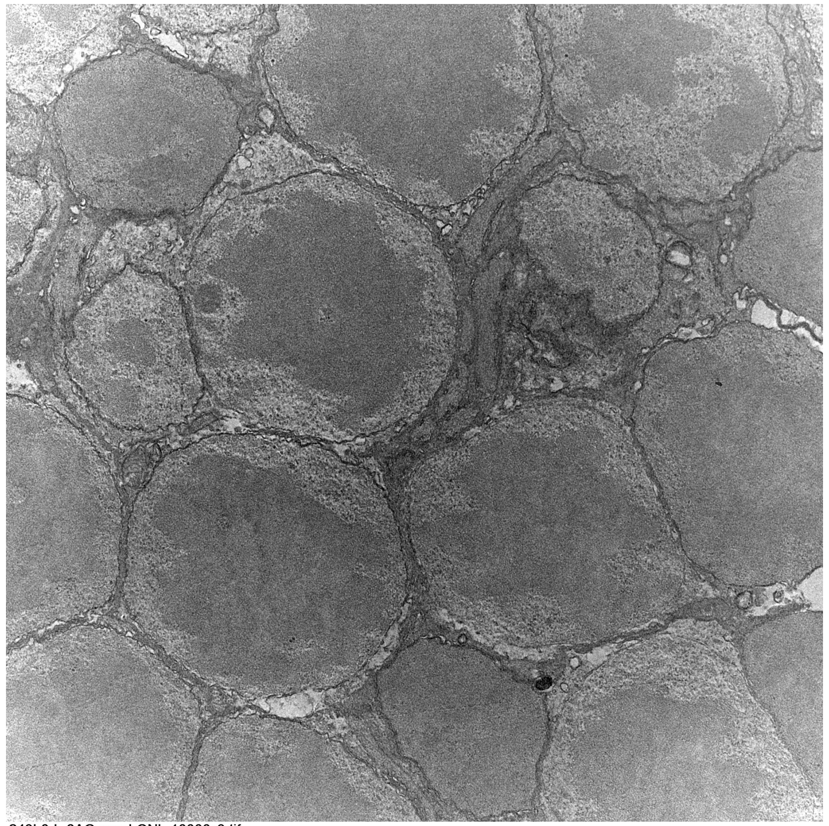

243L0-L-8AG-aged-ONL-10000x3.tif
243L0-L aged 8AG treated
ONL 10000x3
Print Mag: 11700x @ 7.0 in
14:36:35 3/8/2023

1 µm
HV=80.0kV
Direct Mag: 10000x
Center for Biologic Imaging

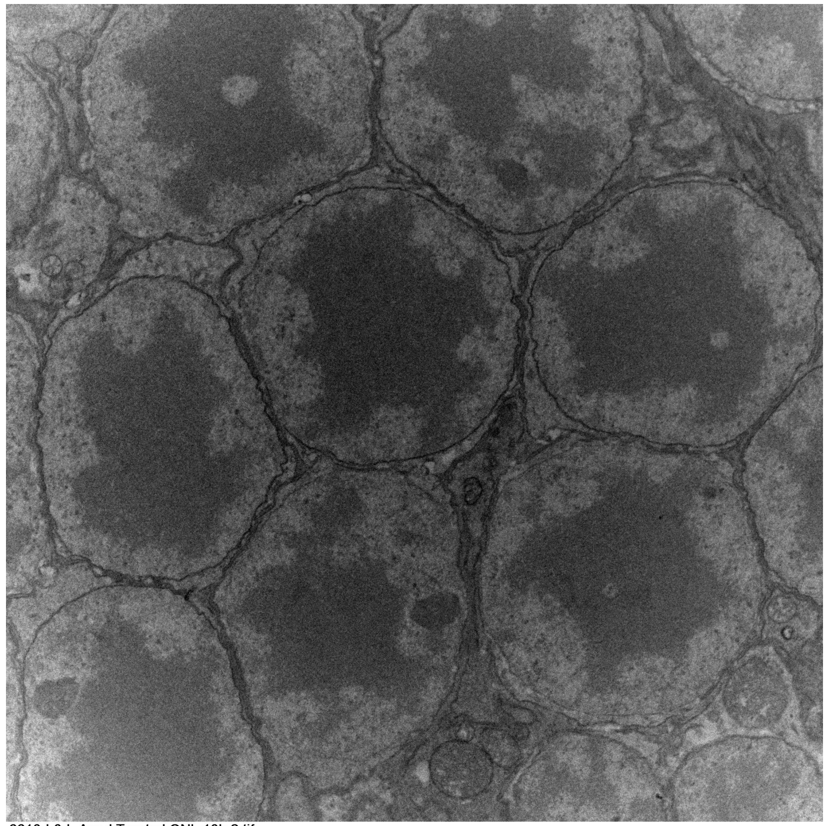

6610-L0-L Aged Treated ONL 10k-2.tif
611-R0-R Aged Untreated
Print Mag: 11700x @ 7.0 in
12:43:32 4/27/2023

1 µm
HV=80.0kV
Direct Mag: 10000x
Center for Biologic Imaging

8-AG treated aged central-ONL

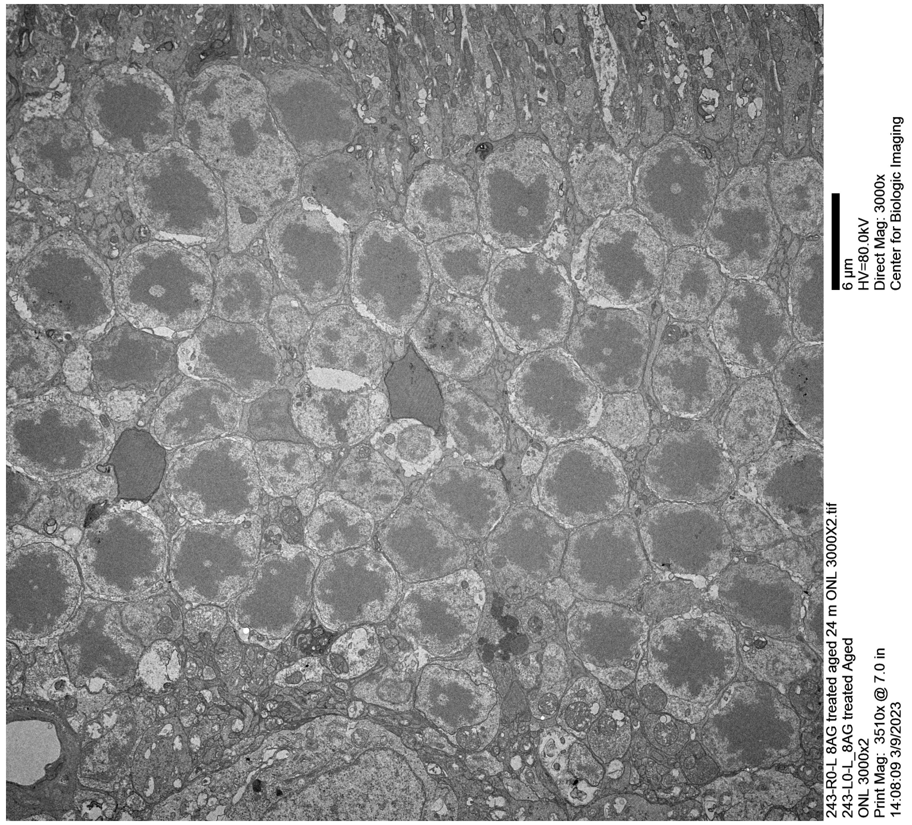

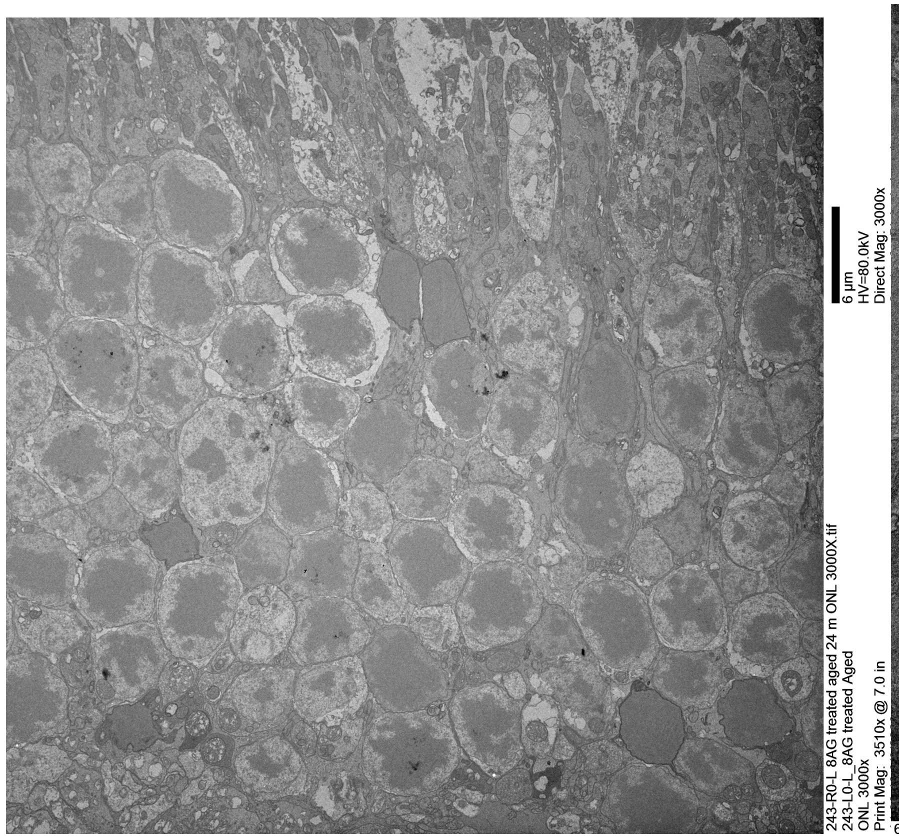

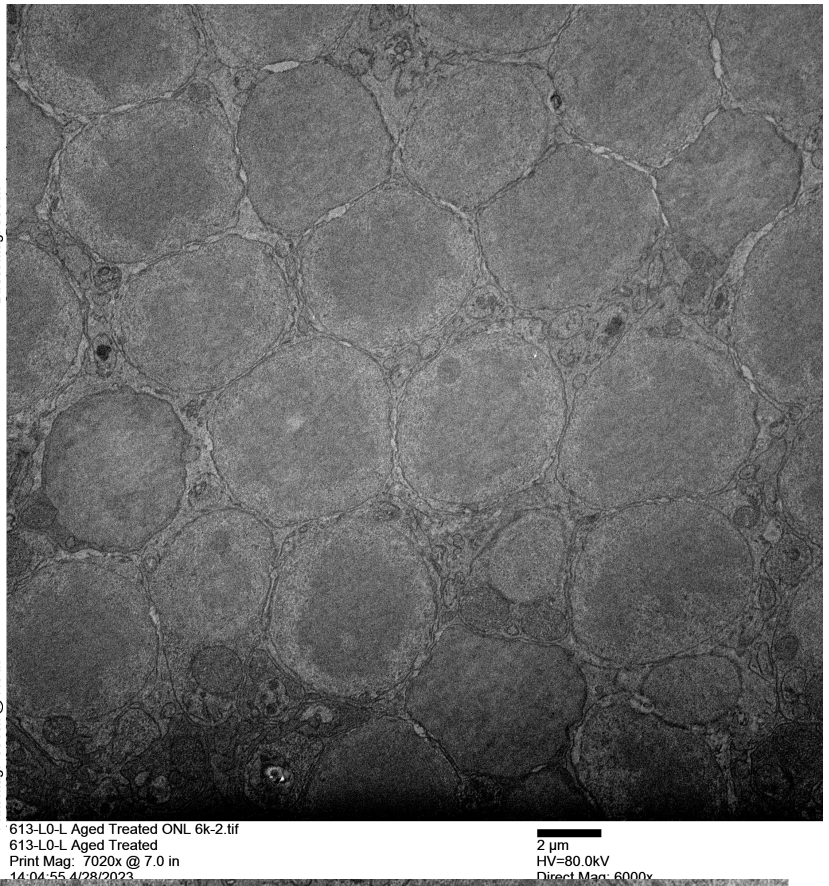

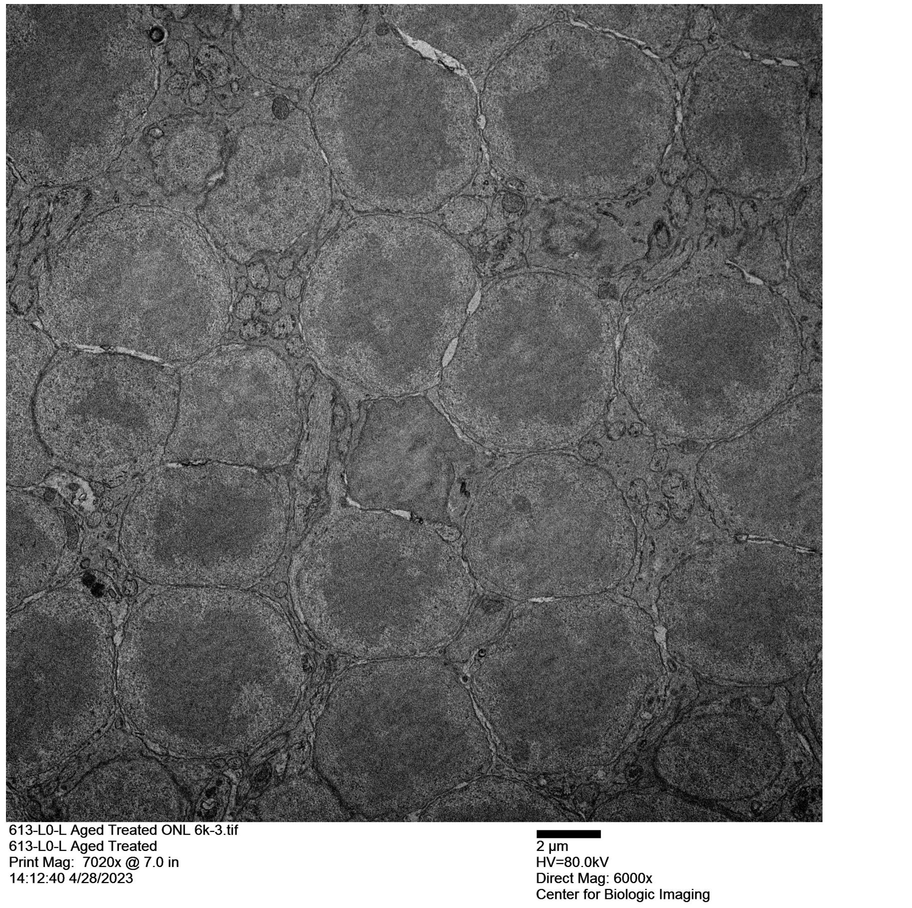

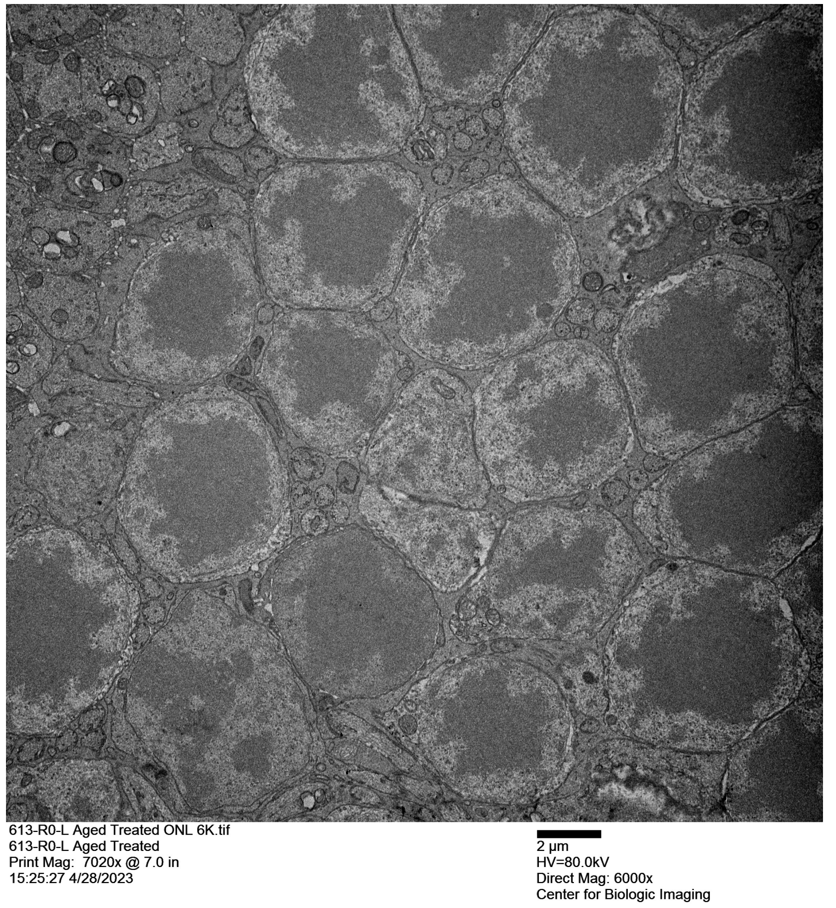

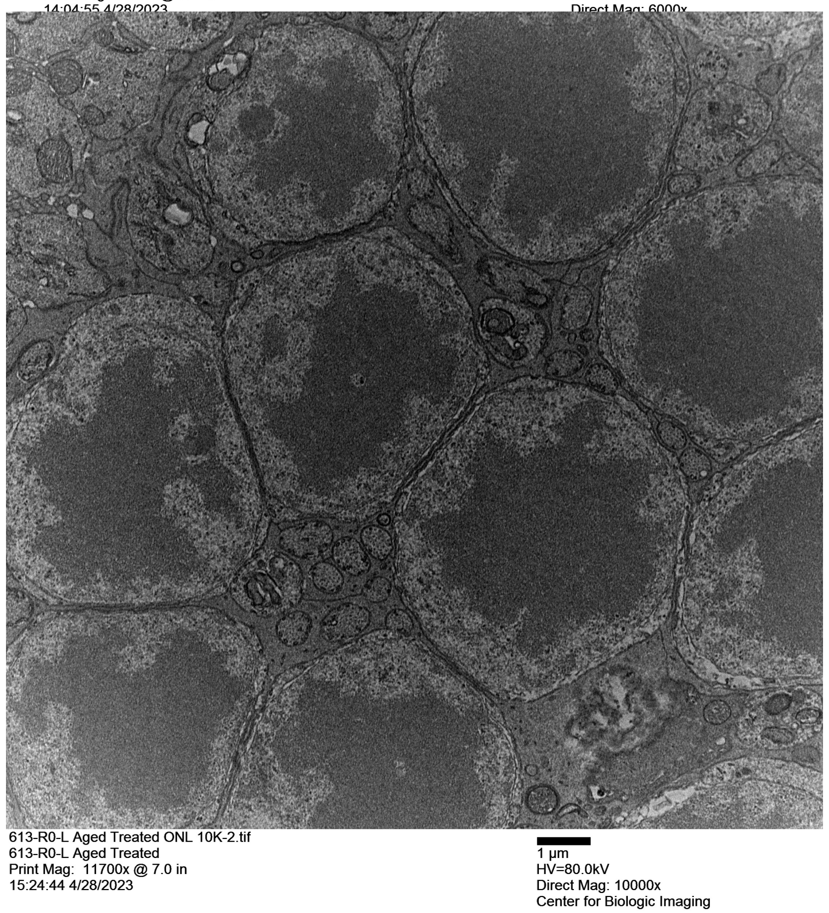

8-AG treated aged central-ONL

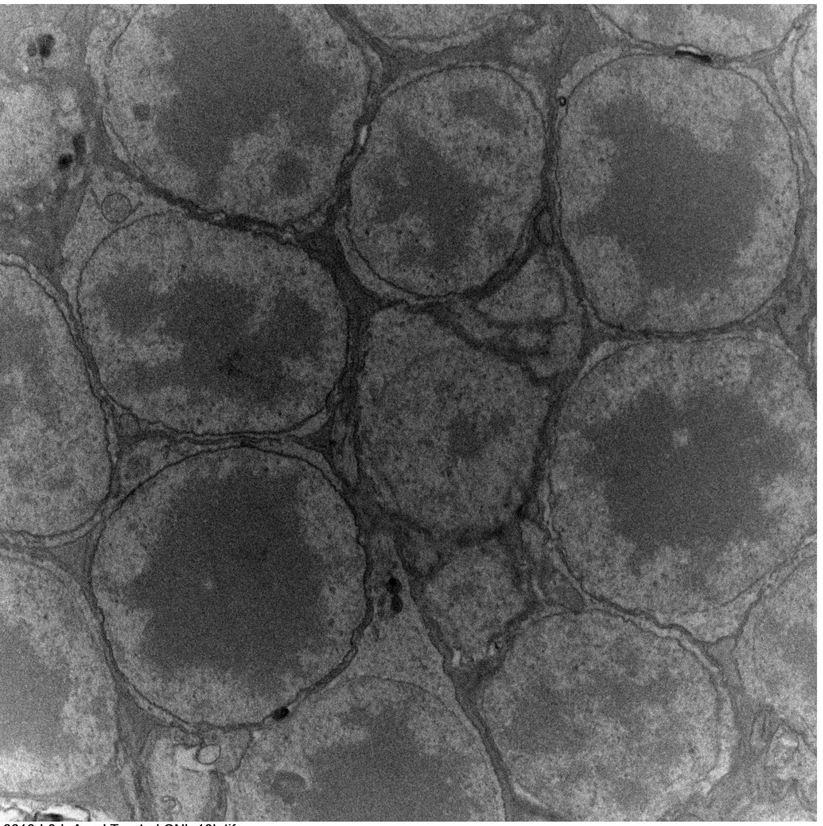

6610-L0-L Aged Treated ONL 10k.tif
611-R0-R Aged Untreated
Print Mag: 11700x @ 7.0 in
12:42:50 4/27/2023

1 µm
HV=80.0kV
Direct Mag: 10000x
Center for Biologic Imaging

8-AG treated aged central retina-OPL

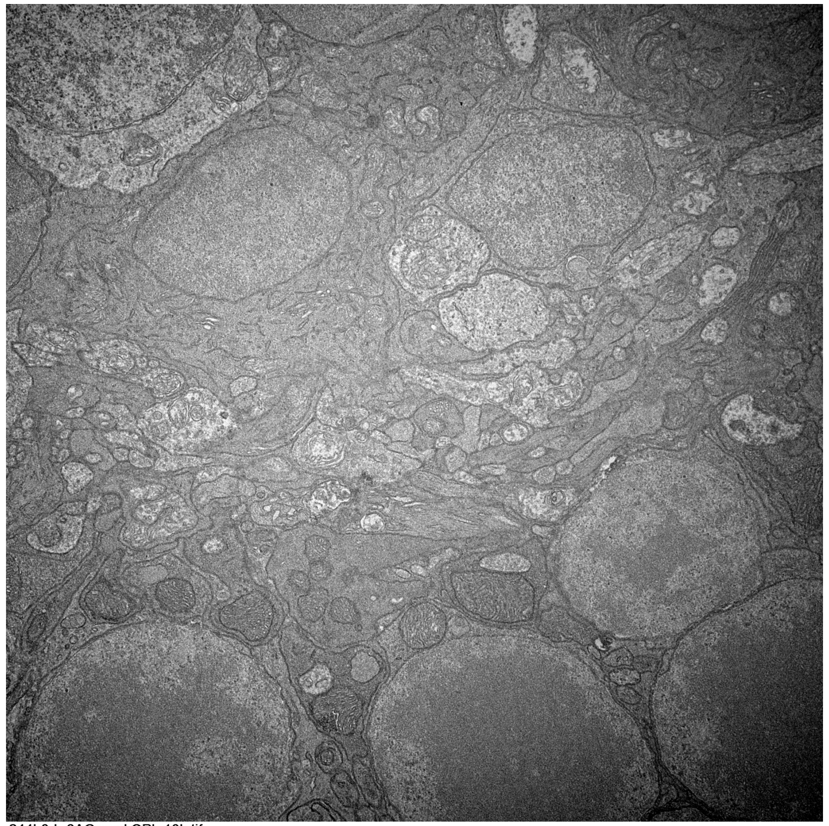

244L0-L-8AGaged OPL 10k.tif
244-L0-L
aged-8AG treated OPL10kx
Print Mag: 11700x @ 7.0 in
11:51:14 3/29/2023

1 µm
HV=80.0kV
Direct Mag: 10000x
Center for Biologic Imaging

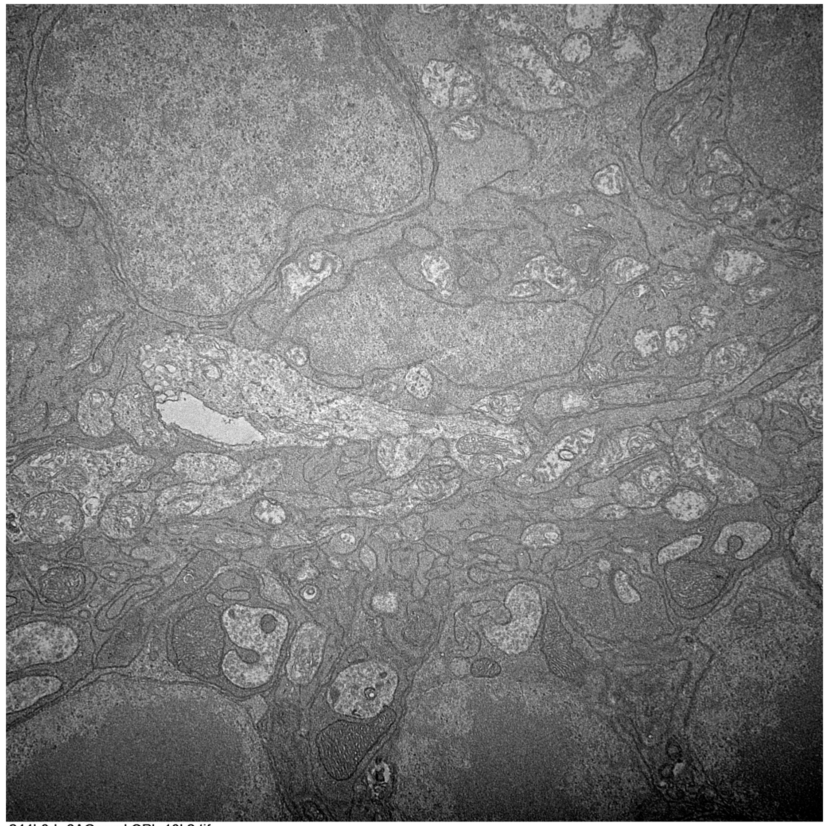

244L0-L-8AGaged OPL 10k2.tif
244-L0-L
aged-8AG treated OPL10kx2
Print Mag: 11700x @ 7.0 in
11:55:06 3/29/2023

1 µm
HV=80.0kV
Direct Mag: 10000x
Center for Biologic Imaging

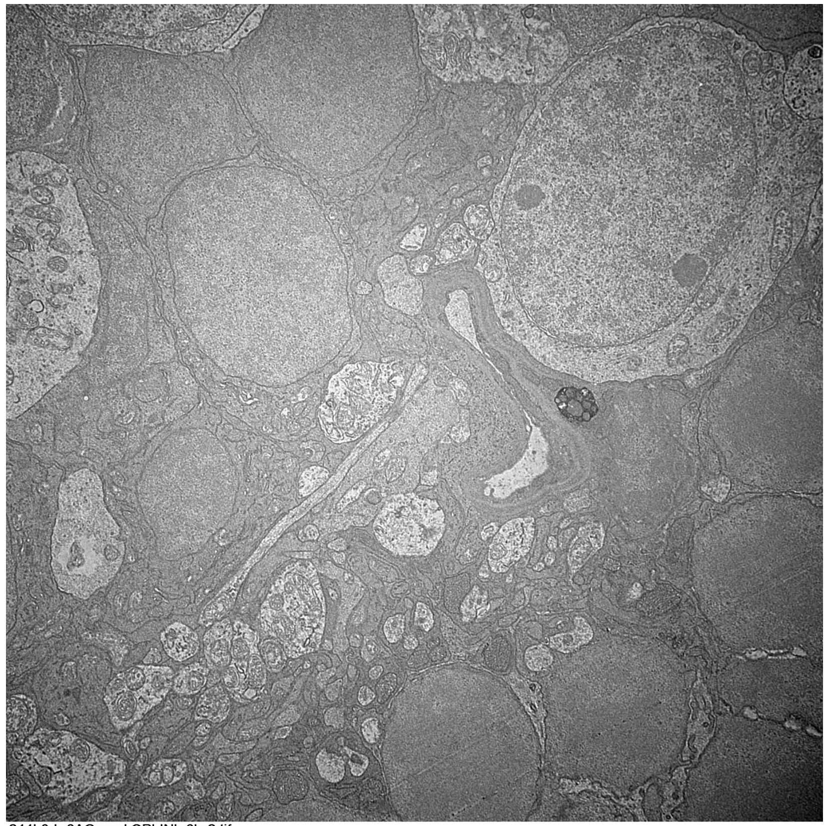

244L0-L-8AGaged OPLINL 6kx2.tif
244-L0-L
aged-8AG treated OPLINL 6kx
Print Mag: 7020x @ 7.0 in
13:47:48 3/29/2023

2 µm
HV=80.0kV
Direct Mag: 6000x
Center for Biologic Imaging

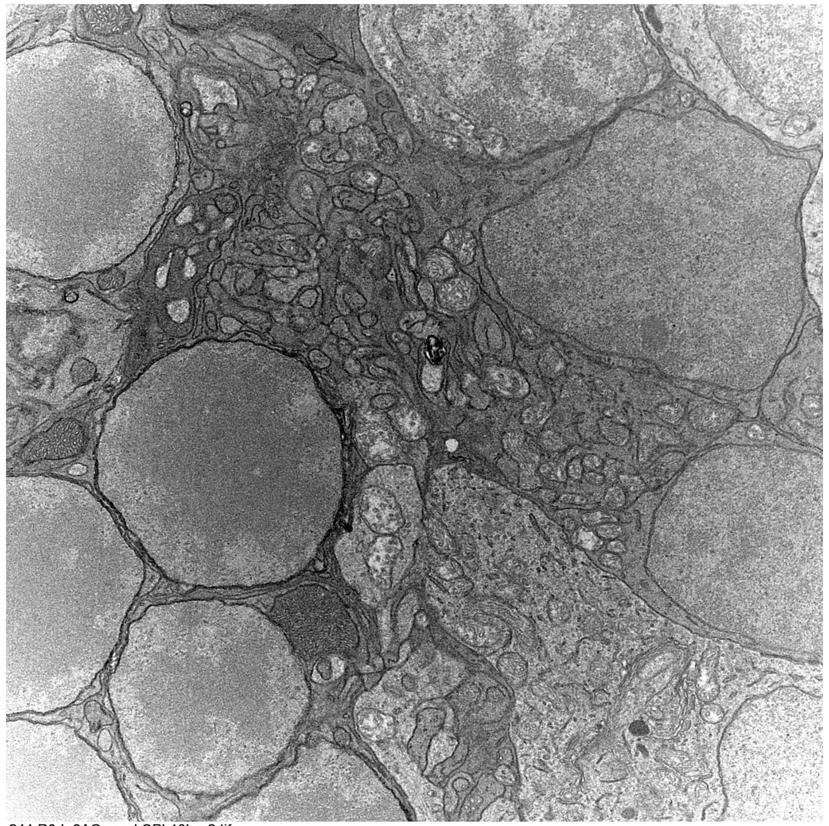

244-R0-L 8AG aged OPL10k x 2.tif
244-L0-L
aged-8AG treated ONL OPL 10kx2
Print Mag: 11700x @ 7.0 in
16:38:22 3/29/2023

1 µm
HV=80.0kV
Direct Mag: 10000x
Center for Biologic Imaging

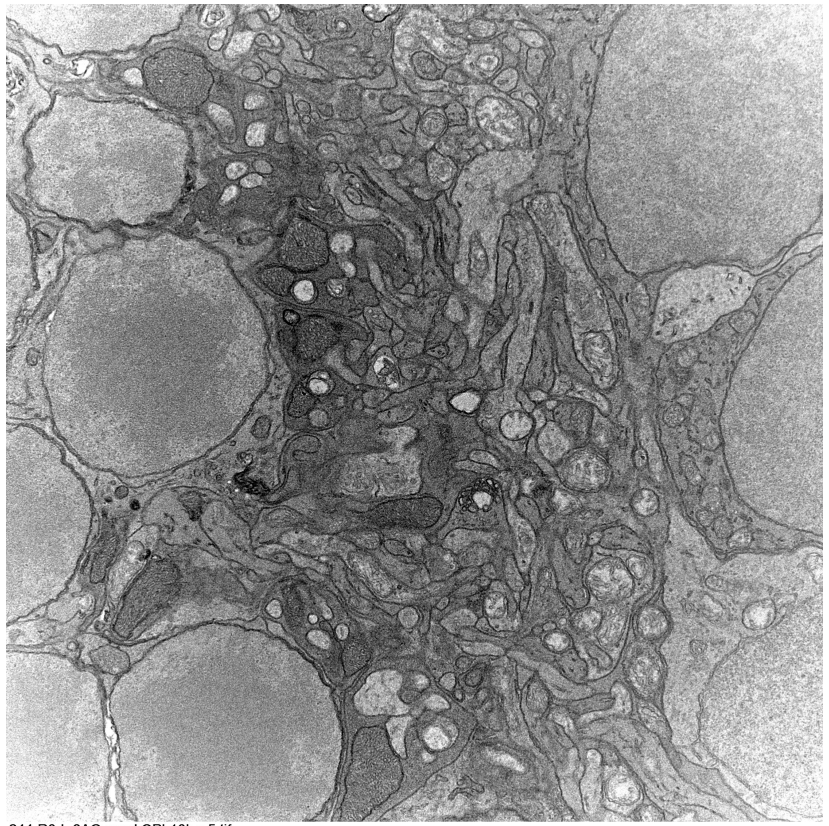

244-R0-L 8AG aged OPL10k x 5.tif
244-L0-L
aged-8AG treated ONL OPL 10kx5
Print Mag: 11700x @ 7.0 in
16:42:38 3/29/2023

1 µm
HV=80.0kV
Direct Mag: 10000x
Center for Biologic Imaging

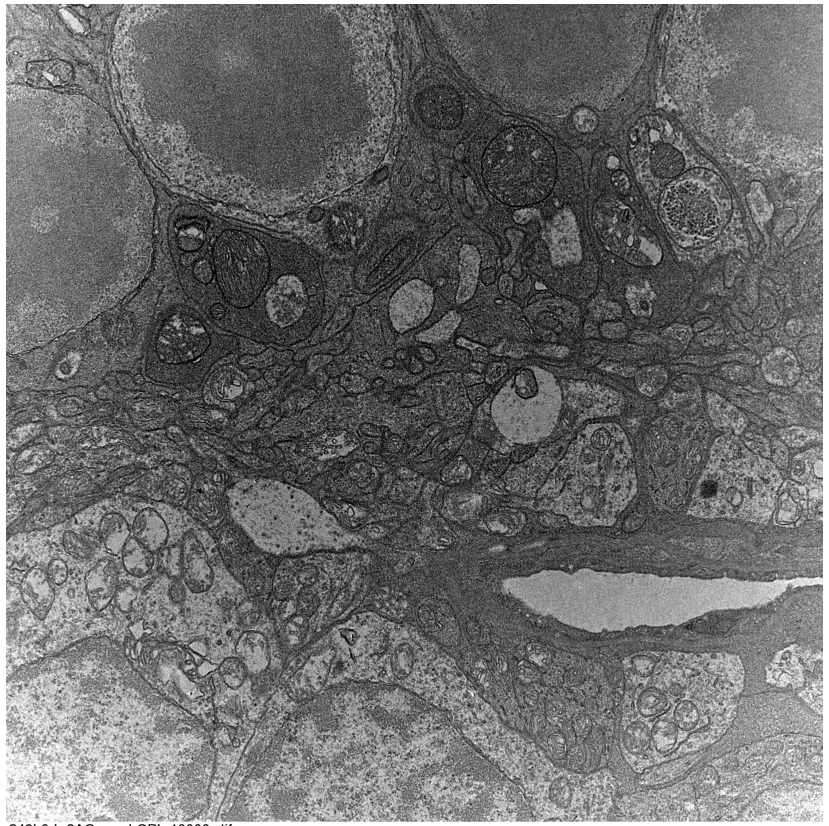

243L0-L-8AG-aged-OPL-10000x.tif
243L0-L Aged treated
OPL10000
Print Mag: 11700x @ 7.0 in
13:48:36 3/8/2023

1 µm
HV=80.0kV
Direct Mag: 10000x
Center for Biologic Imaging

8-AG treated aged central retina-OPL

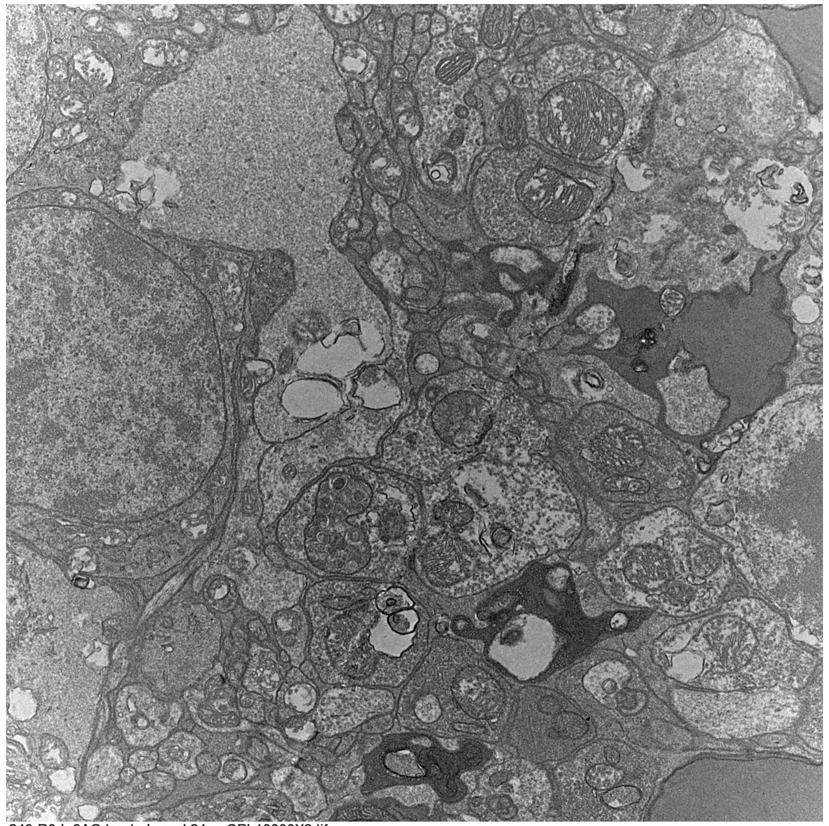

243-R0-L 8AG treated aged 24 m OPL10000X2.tif
243-L0-L_8AG treated Aged
OPL 10000X2
Print Mag: 11700x @ 7.0 in
16:02:34 3/9/2023

1 µm
HV=80.0kV
Direct Mag: 10000x
Center for Biologic Imaging

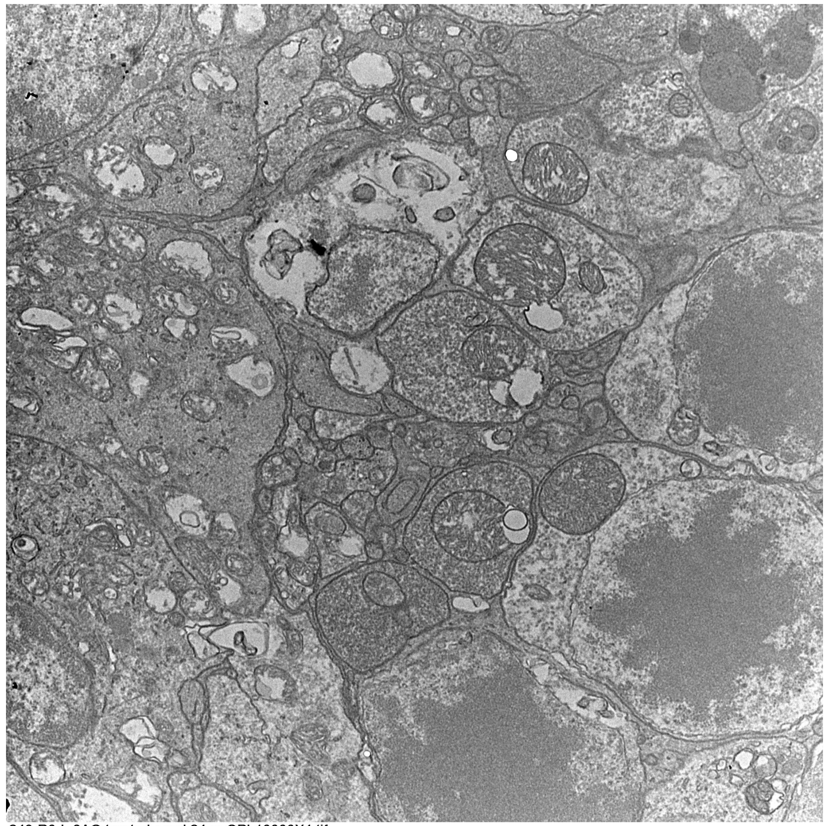

243-R0-L 8AG treated aged 24 m OPL10000X4.tif
243-L0-L_8AG treated Aged
OPL 10000X4
Print Mag: 11700x @ 7.0 in
16:05:51 3/9/2023

1 µm
HV=80.0kV
Direct Mag: 10000x
Center for Biologic Imaging

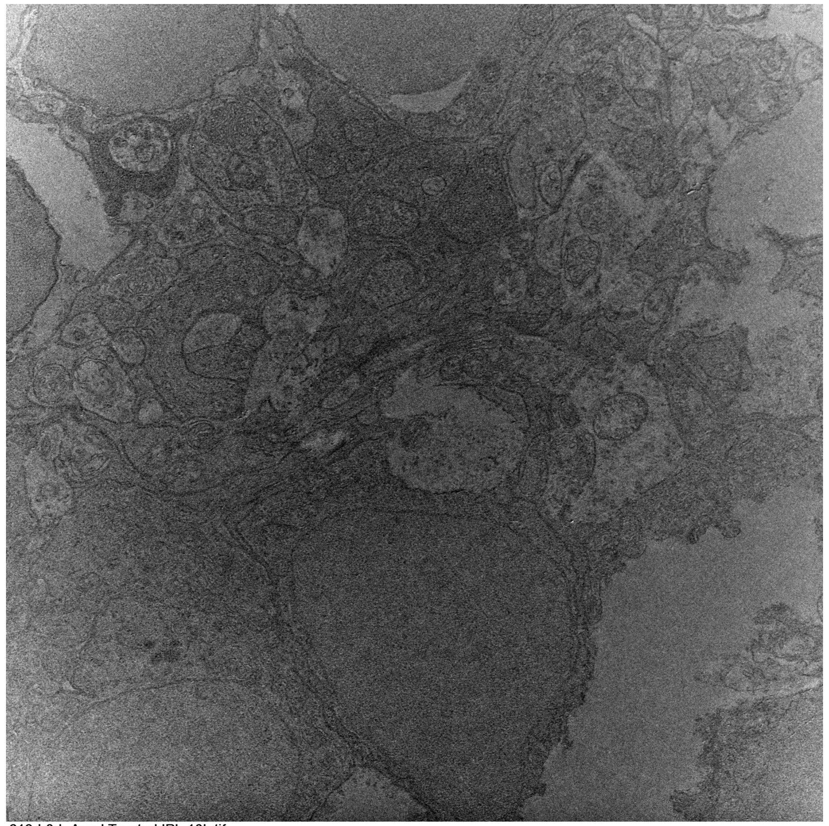

613-L0-L Aged Treated IPL 10k.tif
613-L0-L Aged Treated
Print Mag: 11700x @ 7.0 in
14:10:03 4/28/2023

1 µm
HV=80.0kV
Direct Mag: 10000x
Center for Biologic Imaging

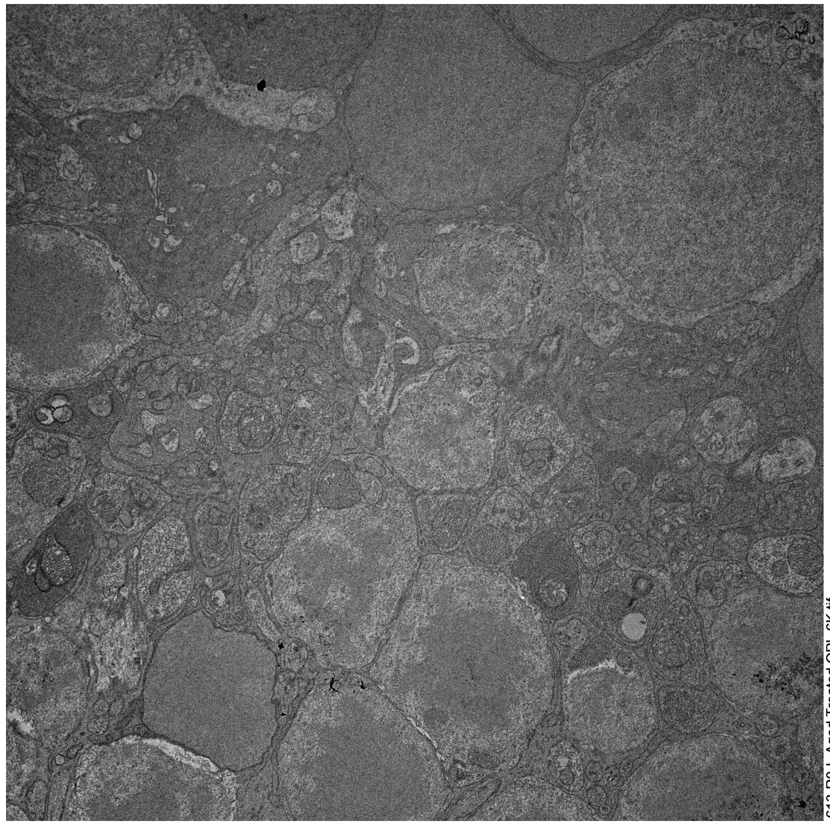

613-R0-L Aged Treated OPL 6K.tif
613-R0-L Aged Treated
Print Mag: 7020x @ 7.0 in

2 µm
HV=80.0kV

6610-L0-L Aged Treated OPL 10k.tif
611-R0-R Aged Untreated
Print Mag: 11700x @ 7.0 in
12:44:32 4/27/2023

1 µm
HV=80.0kV
Direct Mag: 10000x
Center for Biologic Imaging

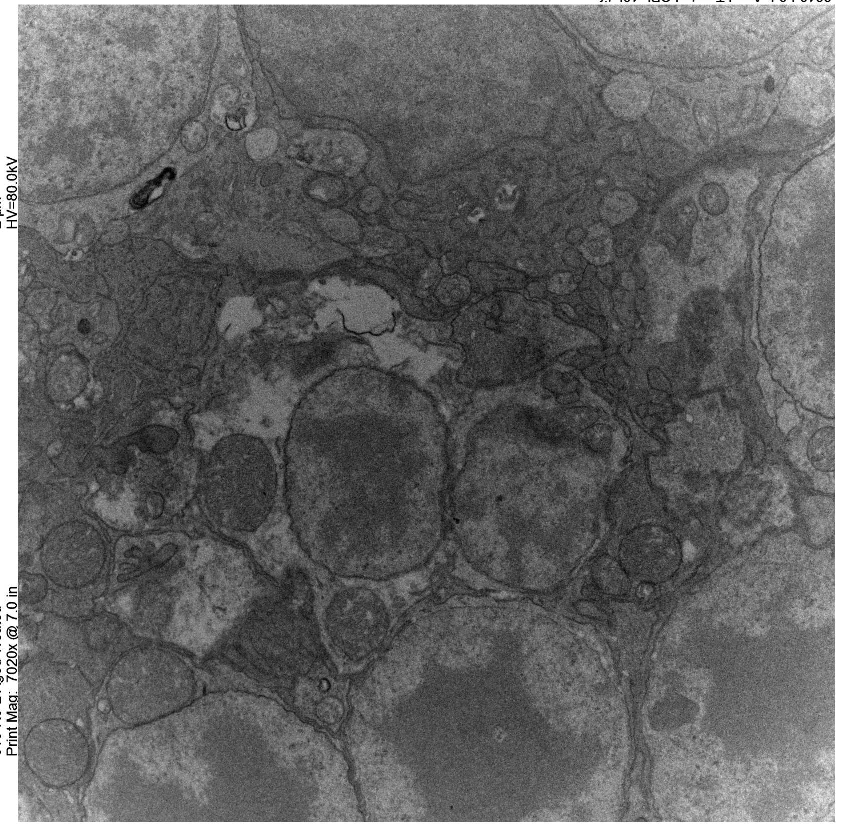

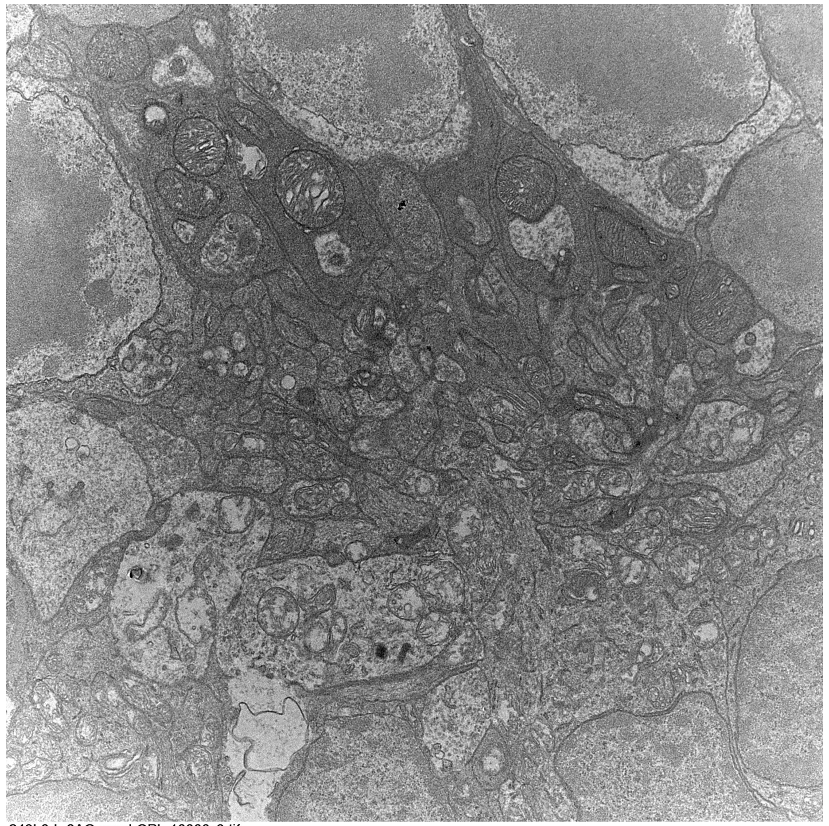

243L0-L-8AG-aged-OPL-10000x3.tif
243L0-L Aged treated
OPL10000x 3
Print Mag: 11700x @ 7.0 in
13:52:23 3/8/2023

1 µm
HV=80.0kV
Direct Mag: 10000x
Center for Biologic Imaging

8-AG treated aged central retina-OS

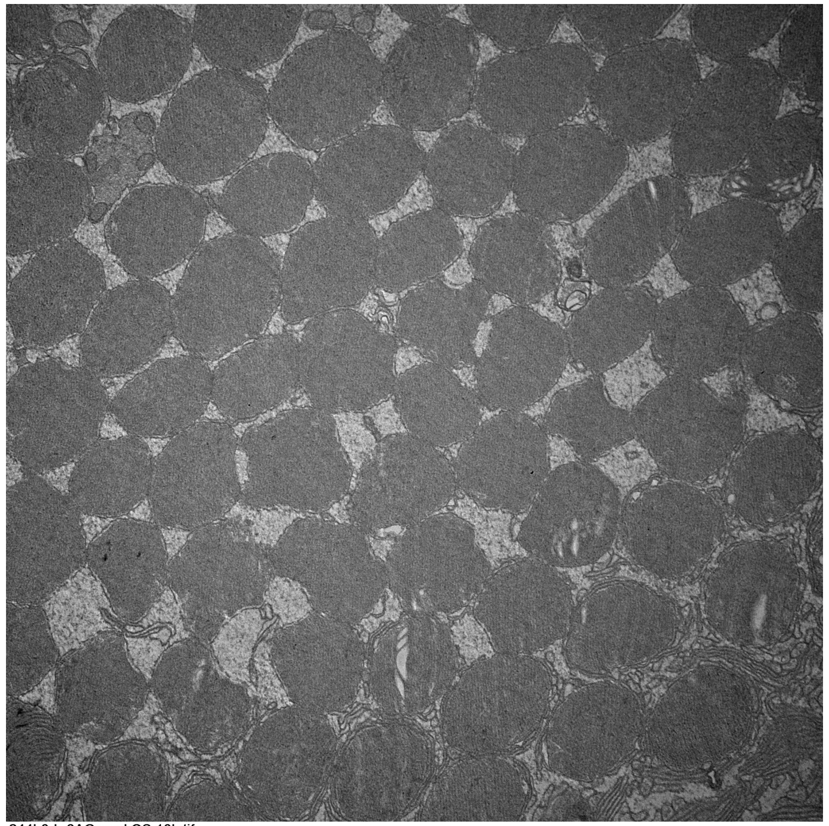

244L0-L-8AGaged OS 10k.tif
244-L0-L
aged-8AG treated OS10kx
Print Mag: 11700x @ 7.0 in
11:42:15 3/29/2023

1 μm
HV=80.0kV
Direct Mag: 10000x
Center for Biologic Imaging

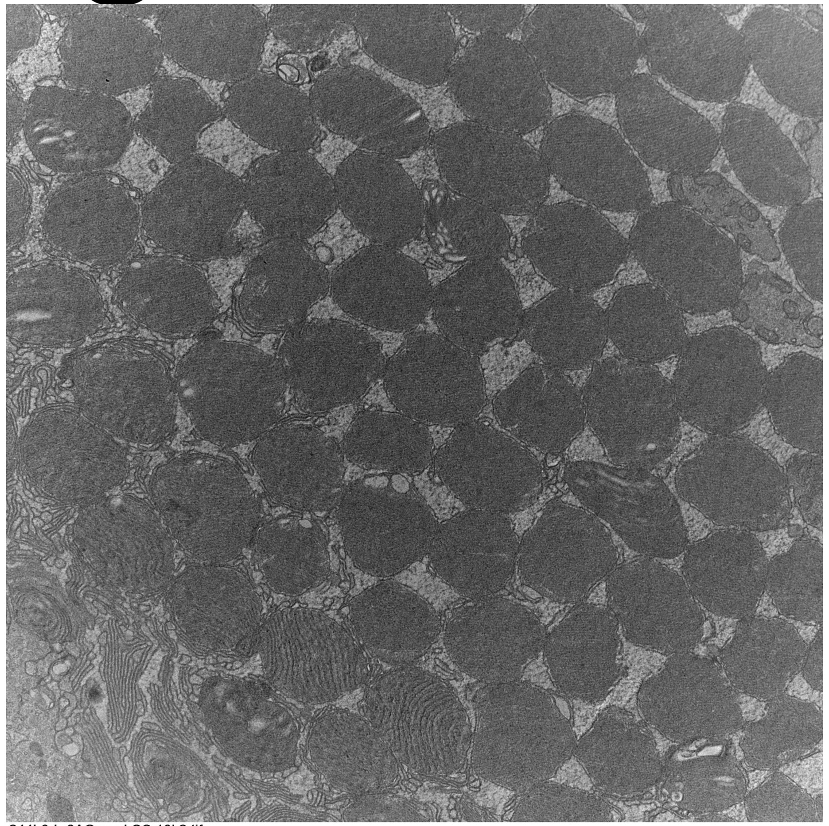

244L0-L-8AGaged OS 10k2.tif
244-L0-L
aged-8AG treated OS10kx2
Print Mag: 11700x @ 7.0 in
11:44:36 3/29/2023

1 μm
HV=80.0kV
Direct Mag: 10000x
Center for Biologic Imaging

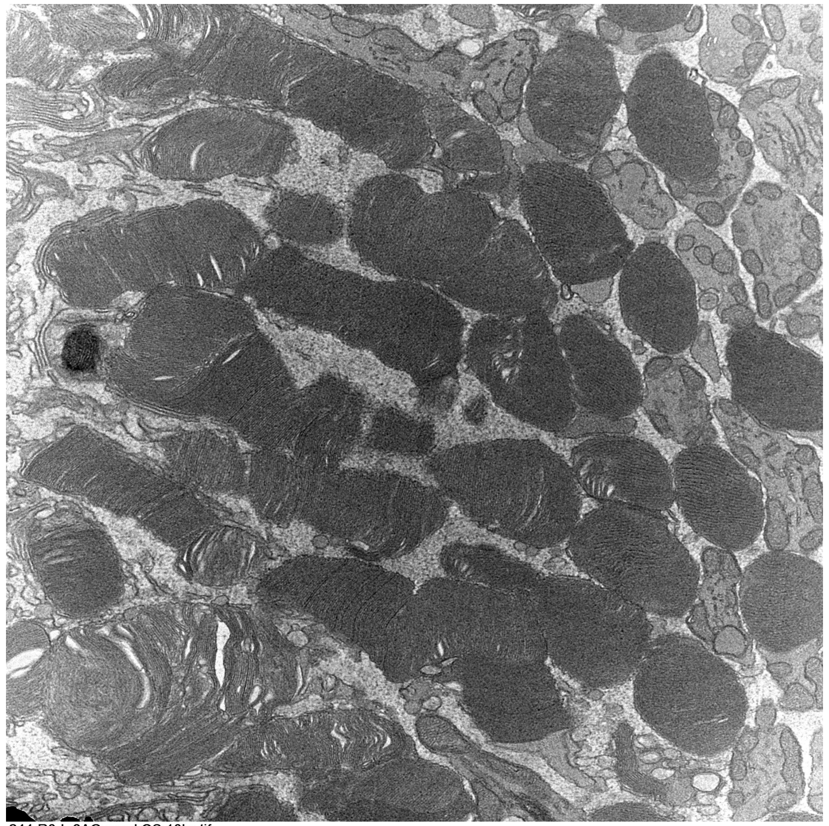

244-R0-L 8AG aged OS 10kx.tif
244-L0-L
aged-8AG treated OS 10k
Print Mag: 11700x @ 7.0 in
16:05:13 3/29/2023

1 μm
HV=80.0kV
Direct Mag: 10000x
Center for Biologic Imaging

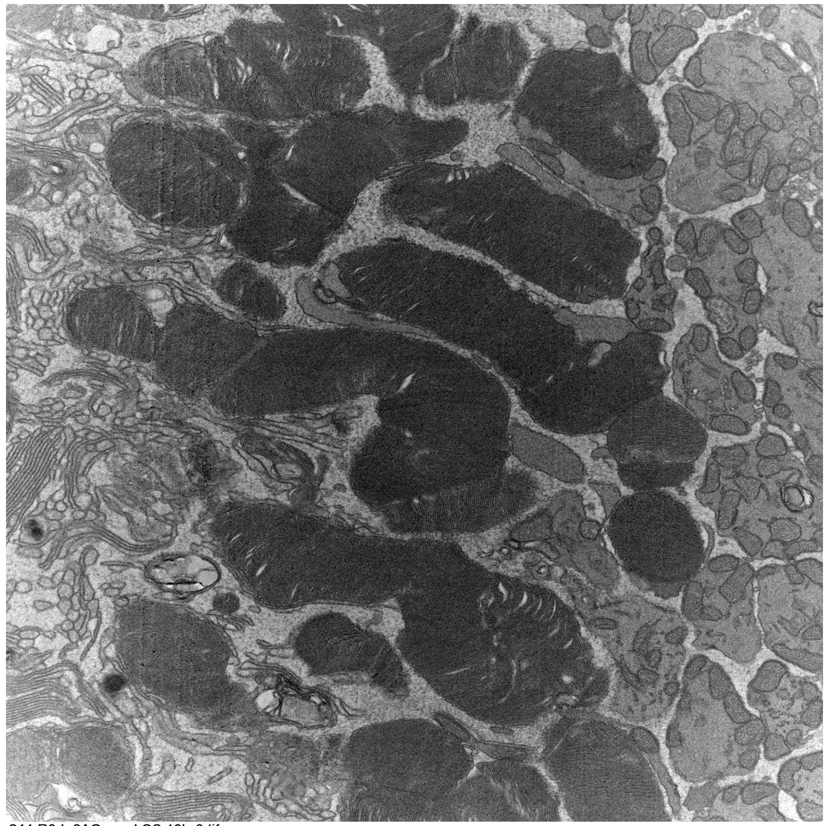

244-R0-L 8AG aged OS 10kx3.tif
244-L0-L
aged-8AG treated OS 10k3
Print Mag: 11700x @ 7.0 in
16:09:37 3/29/2023

1 μm
HV=80.0kV
Direct Mag: 10000x
Center for Biologic Imaging

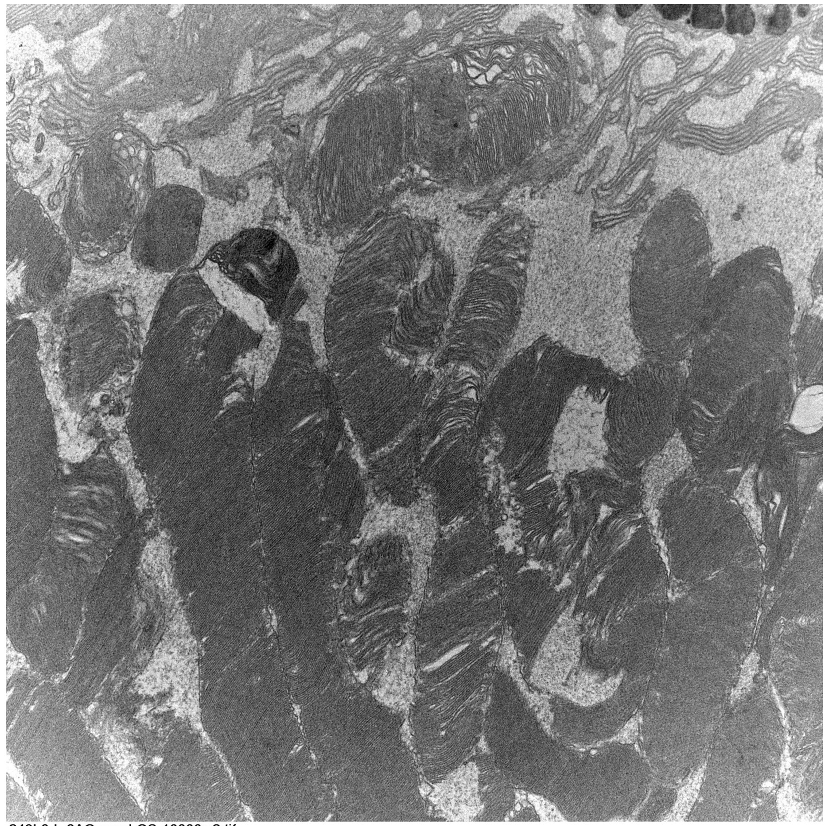

243L0-L-8AG-aged-OS-10000x 2.tif
243L0-L aged 8AG treated
OS10000x2
Print Mag: 11700x @ 7.0 in
15:44:49 3/8/2023

1 μm
HV=80.0kV
Direct Mag: 10000x
Center for Biologic Imaging

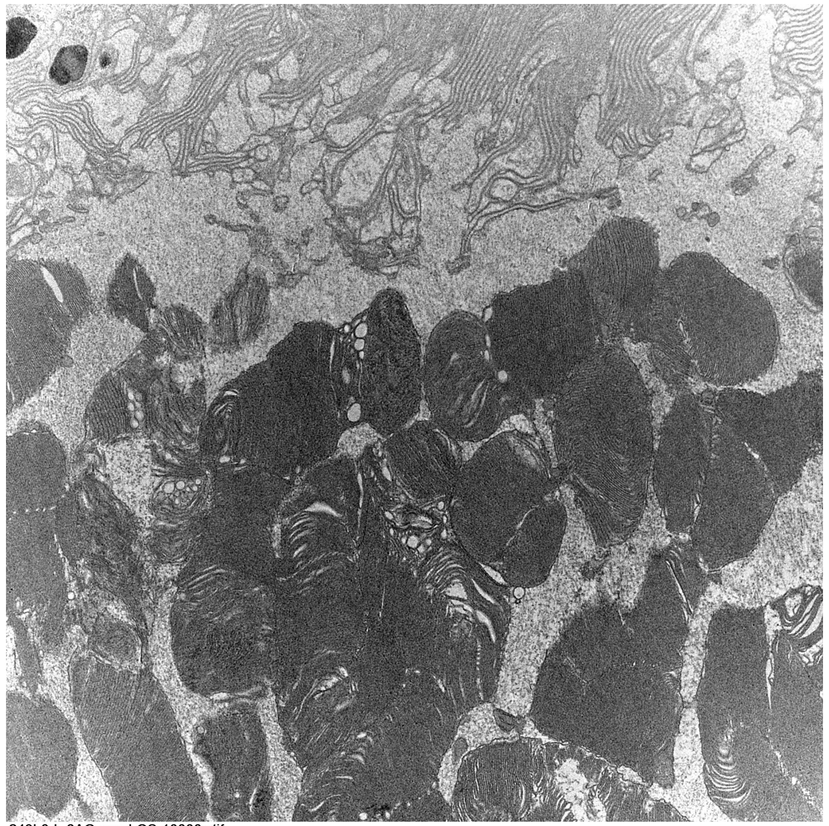

243L0-L-8AG-aged-OS-10000x.tif
243L0-L Aged treated
OS10000
Print Mag: 11700x @ 7.0 in
13:41:12 3/8/2023

1 μm
HV=80.0kV
Direct Mag: 10000x
Center for Biologic Imaging

8-AG treated aged central retina-OS

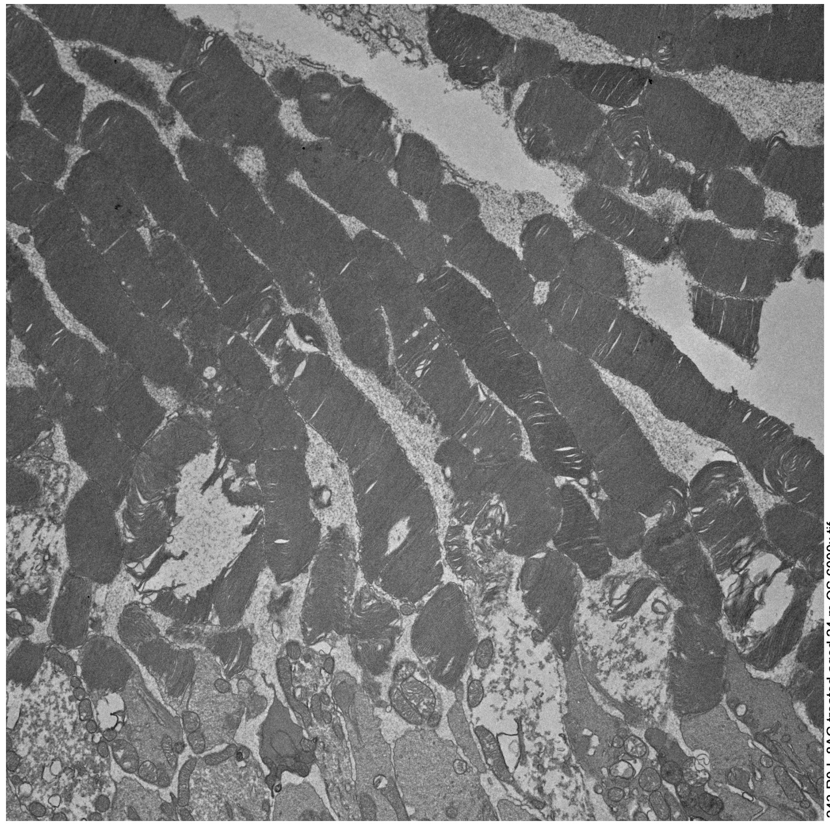

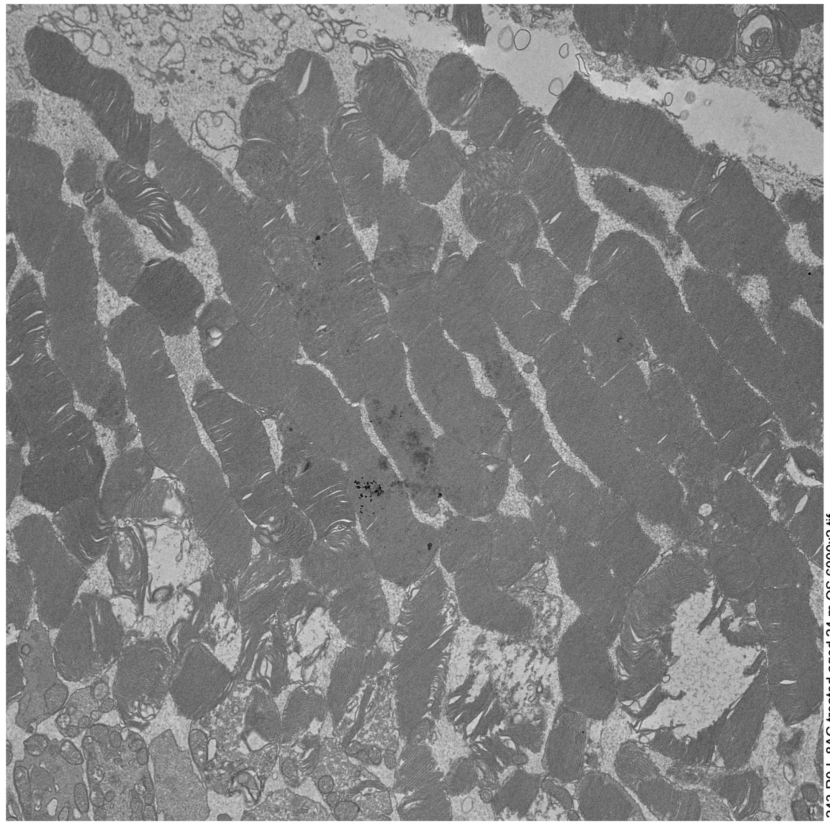

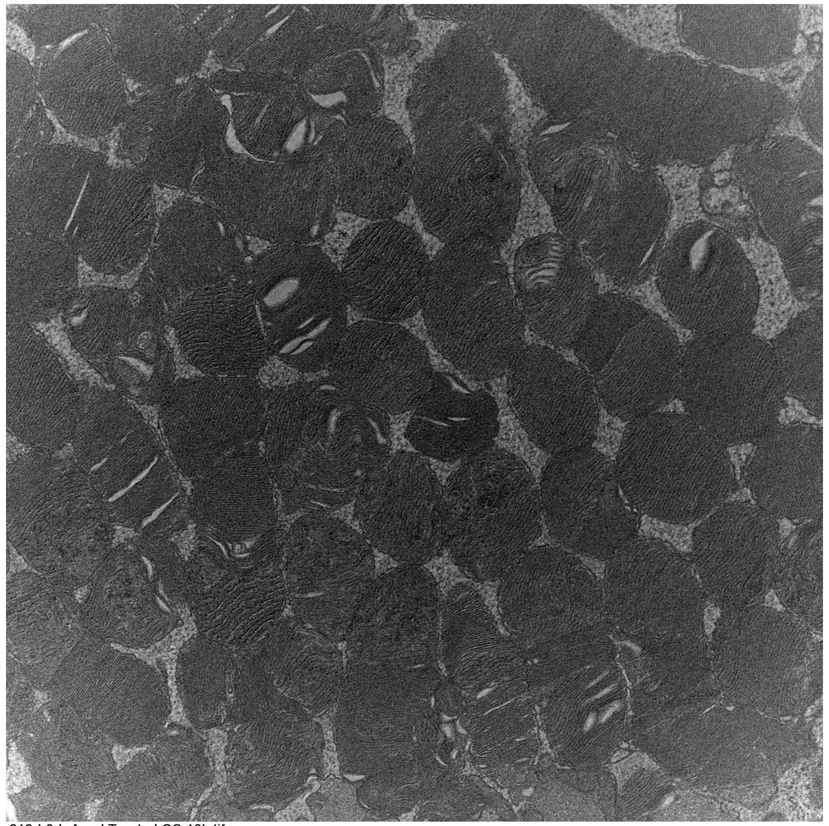

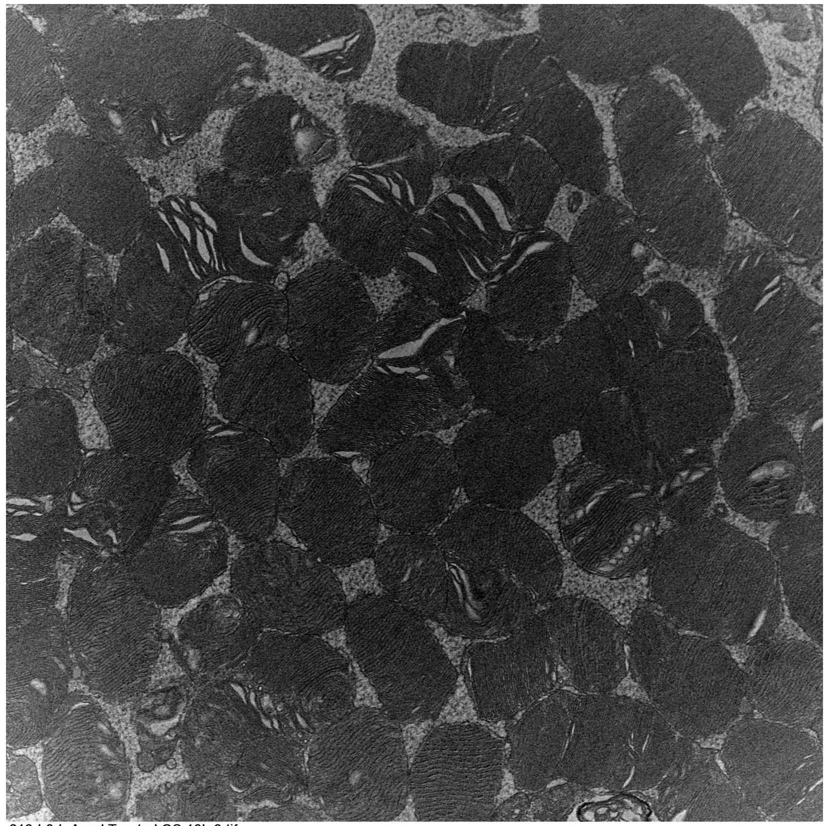

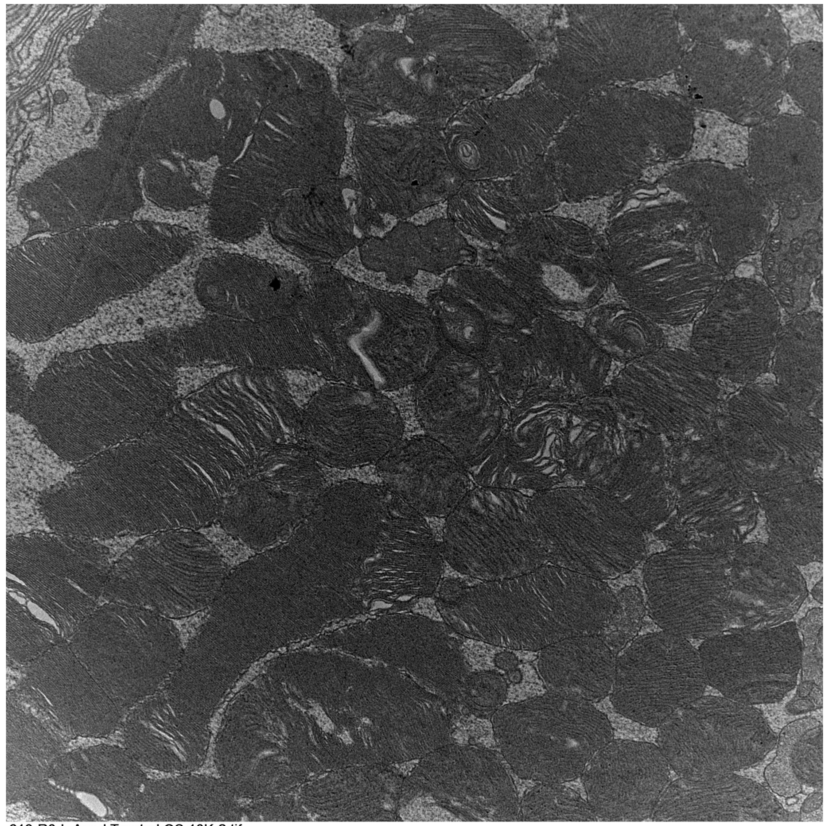

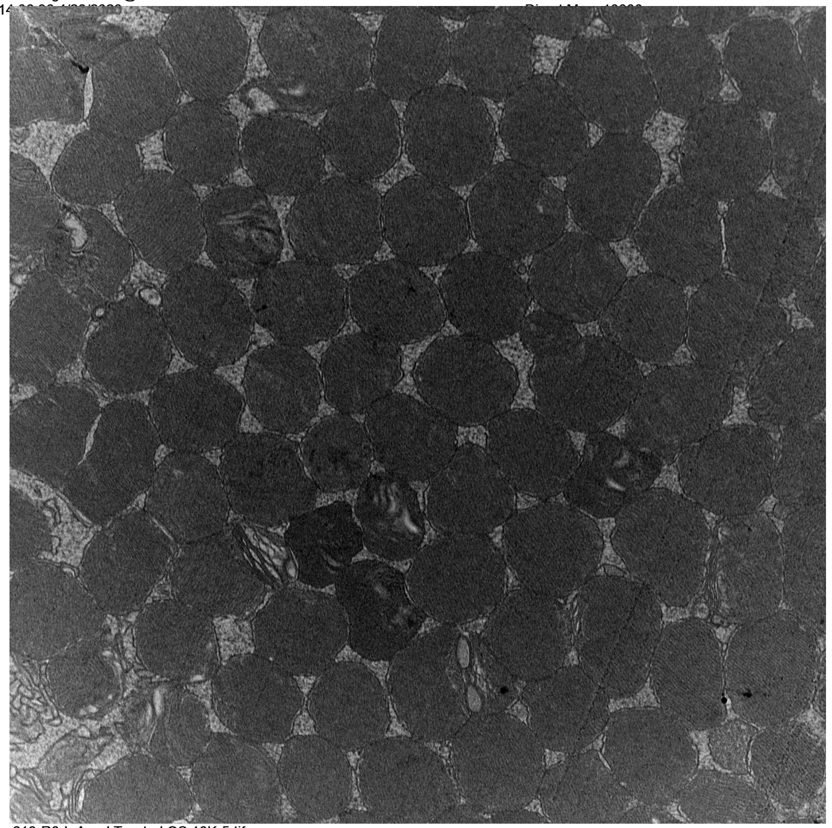

8-AG treated aged central-OS

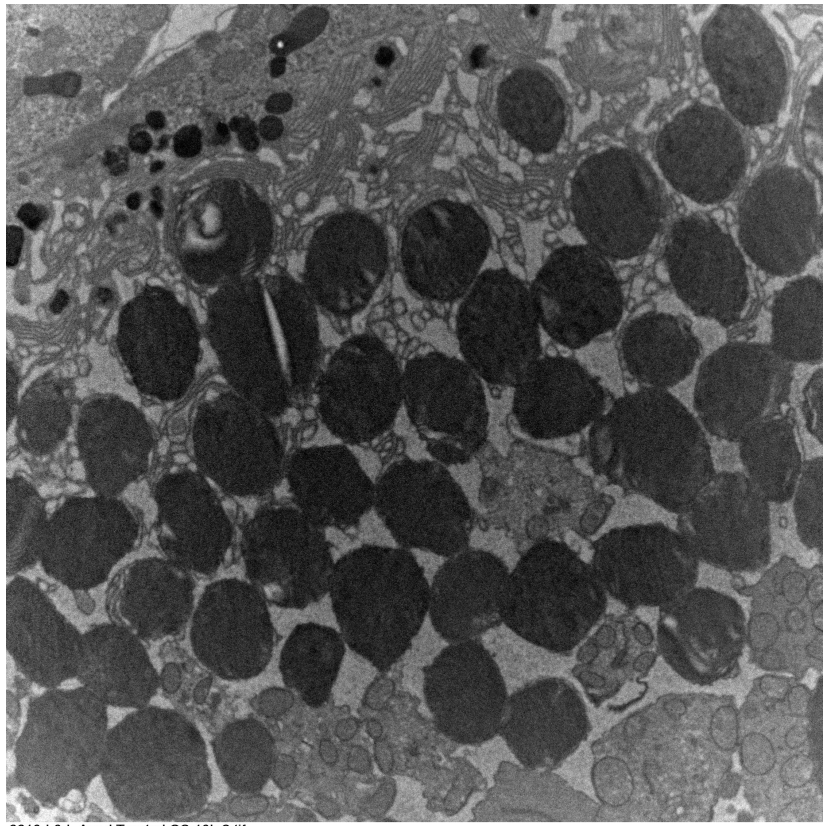

6610-L0-L Aged Treated OS 10k-2.tif
611-R0-R Aged Untreated
Print Mag: 11700x @ 7.0 in
12:33:26 4/27/2023

1 µm
HV=80.0kV
Direct Mag: 10000x
Center for Biologic Imaging

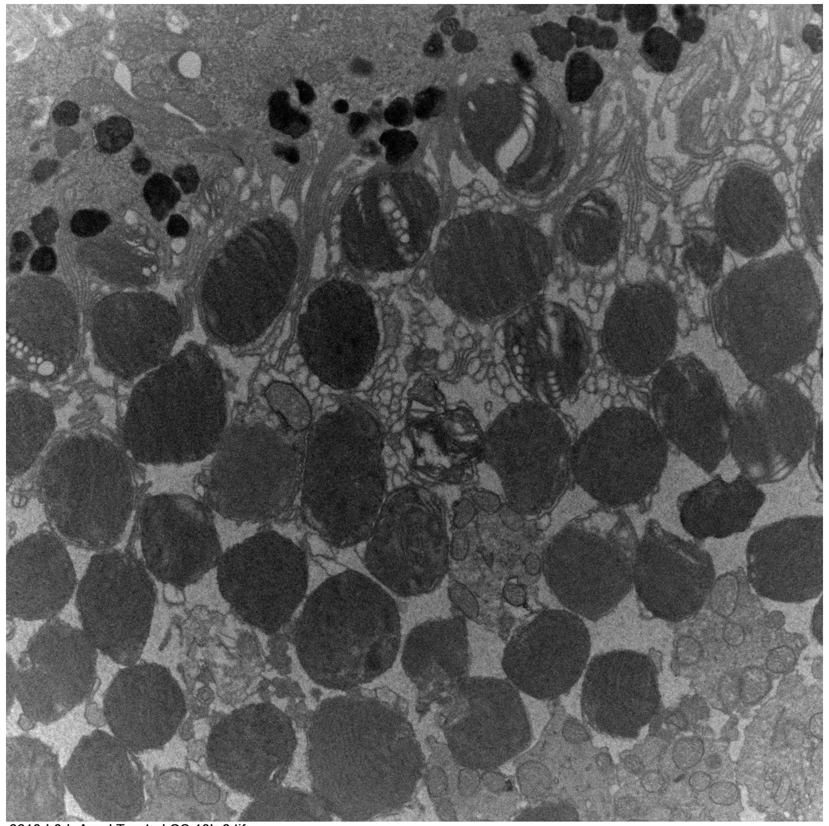

6610-L0-L Aged Treated OS 10k-6.tif
611-R0-R Aged Untreated
Print Mag: 11700x @ 7.0 in
12:40:41 4/27/2023

1 µm
HV=80.0kV
Direct Mag: 10000x
Center for Biologic Imaging

8-AG treated aged central retina-IS

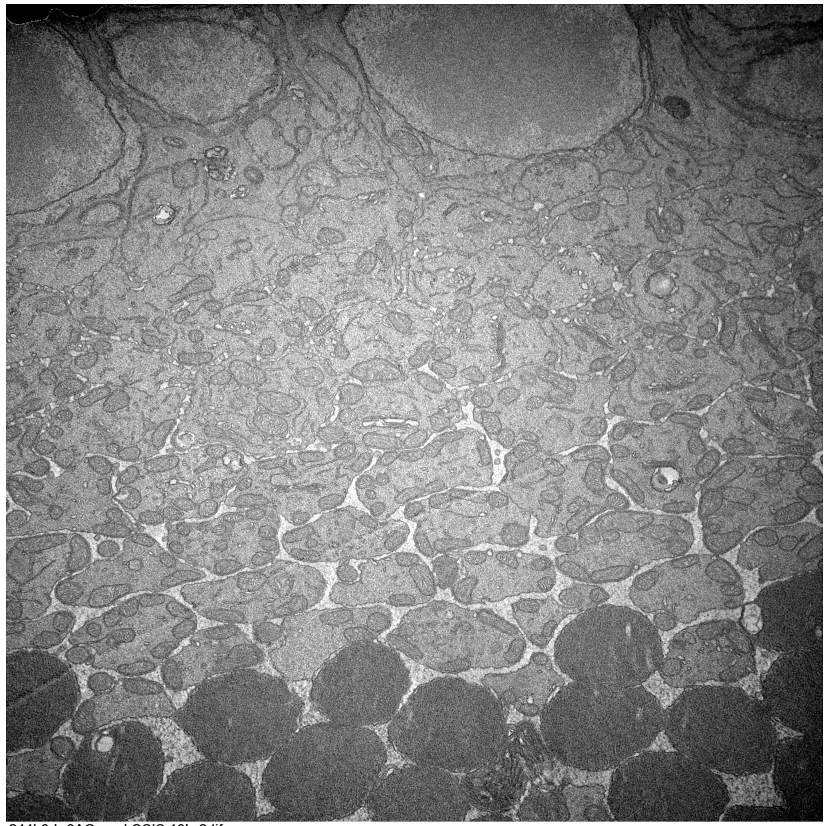

244L0-L-8AGaged OSIS 10kx2.tif
244-L0-L
aged-8AG treated OSIS 10000x2
Print Mag: 11700x @ 7.0 in
10:48:18 3/29/2023

1 µm
HV=80.0kV
Direct Mag: 10000x
Center for Biologic Imaging

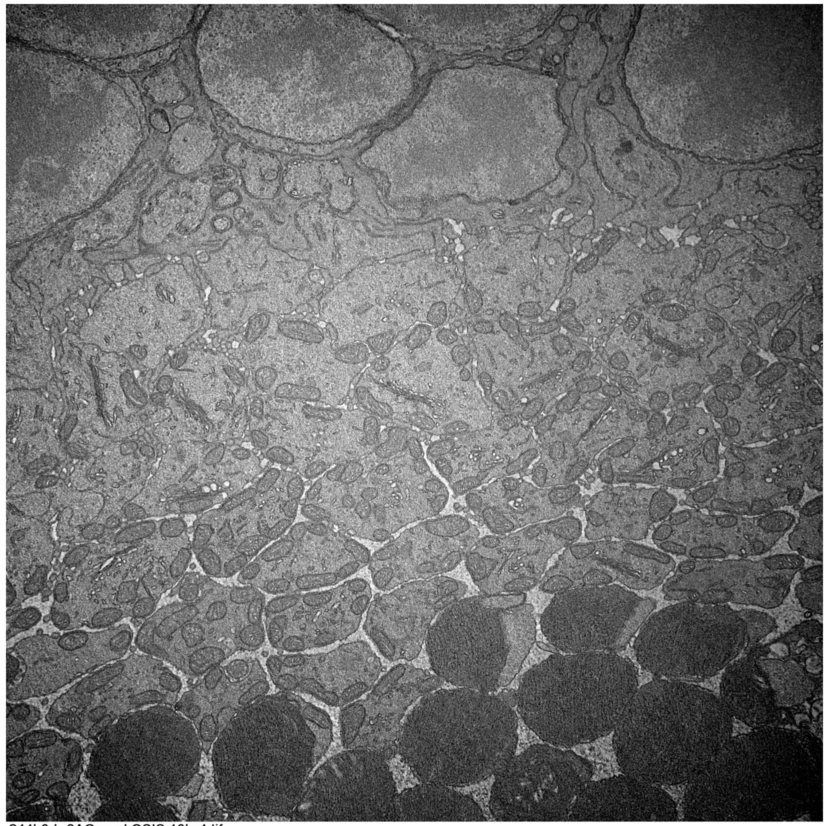

244L0-L-8AGaged OSIS 10kx4.tif
244-L0-L
aged-8AG treated OSIS 10000x4
Print Mag: 11700x @ 7.0 in
10:54:25 3/29/2023

1 µm
HV=80.0kV
Direct Mag: 10000x
Center for Biologic Imaging

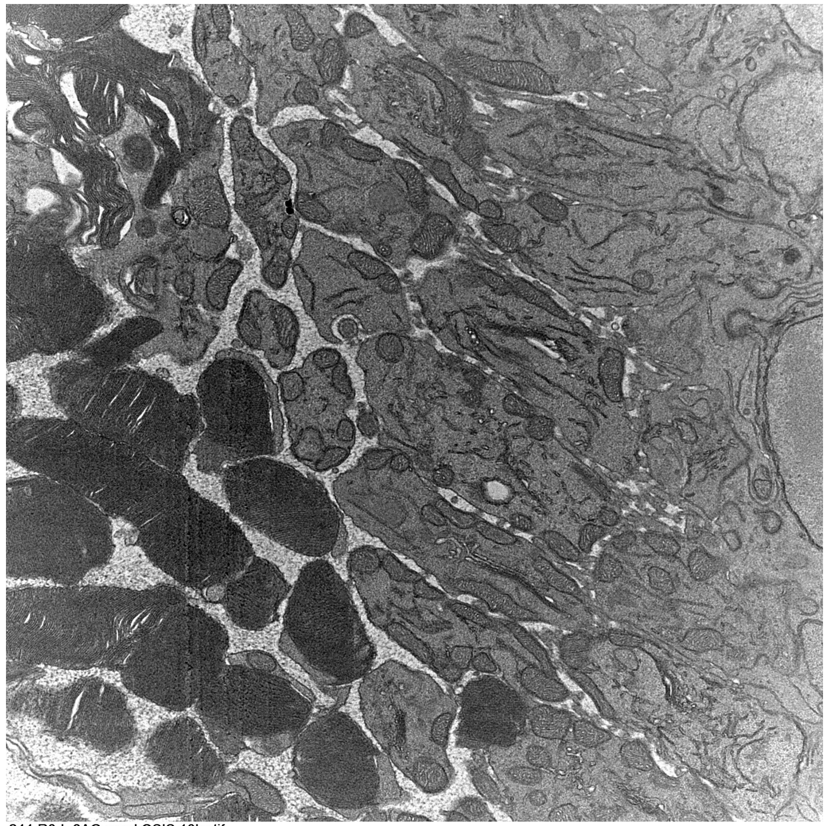

244-R0-L 8AG aged OSIS 10kx.tif
244-L0-L
aged-8AG treated OSIS 10kx
Print Mag: 11700x @ 7.0 in
15:54:12 3/29/2023

1 µm
HV=80.0kV
Direct Mag: 10000x
Center for Biologic Imaging

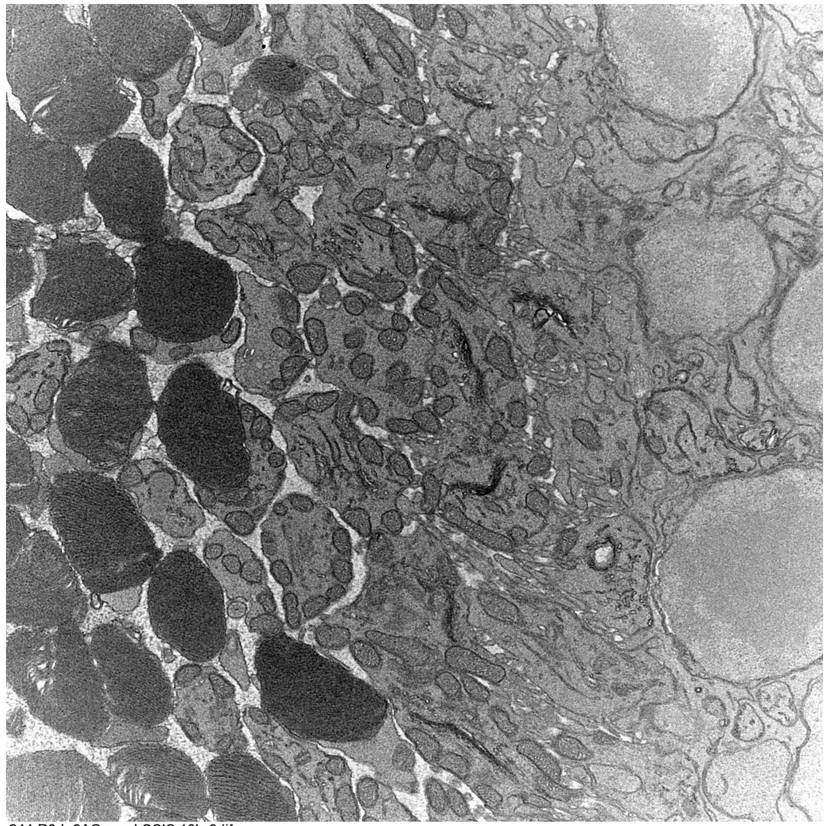

244-R0-L 8AG aged OSIS 10kx3.tif
244-L0-L
aged-8AG treated OSIS 10kx3
Print Mag: 11700x @ 7.0 in
16:02:01 3/29/2023

1 µm
HV=80.0kV
Direct Mag: 10000x
Center for Biologic Imaging

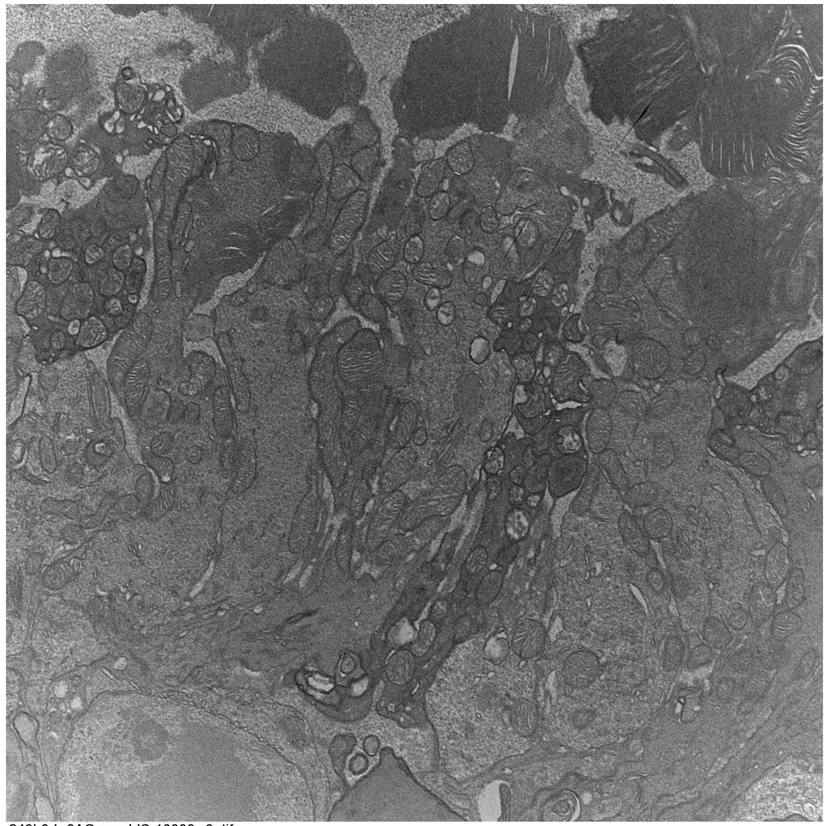

243L0-L-8AG-aged-IS-10000x 3 .tif
243L0-L aged 8AG treated
IS10000x3
Print Mag: 11700x @ 7.0 in
15:52:34 3/8/2023

1 µm
HV=80.0kV
Direct Mag: 10000x
Center for Biologic Imaging

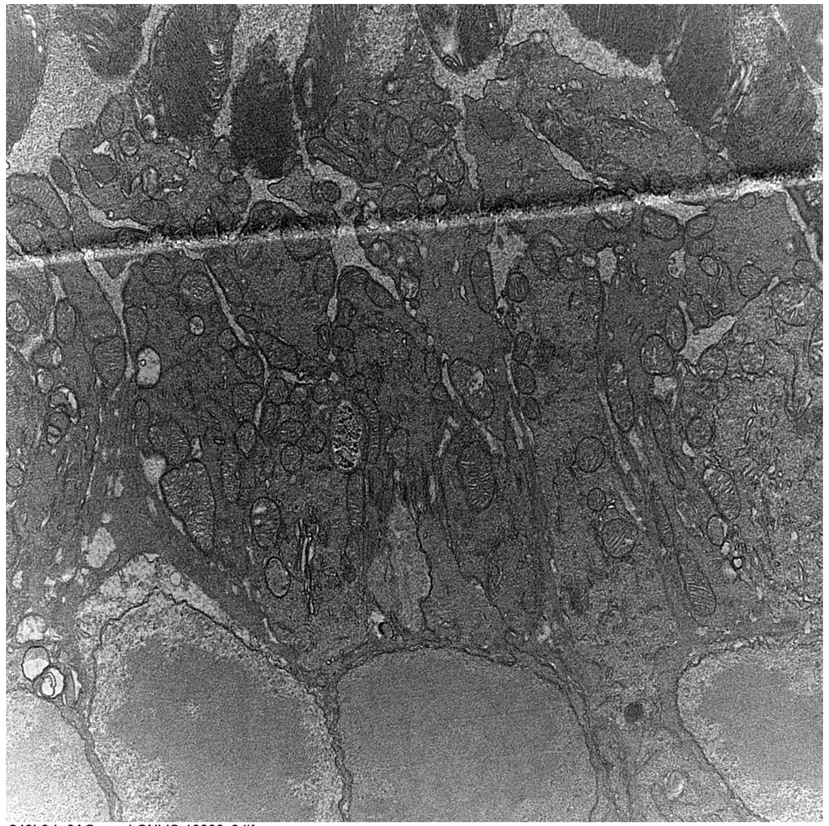

243L0-L-8AG-aged-ONLIS-10000x6.tif
243L0-L aged 8AG treated
IS_ONL 10000x 6
Print Mag: 11700x @ 7.0 in
14:26:41 3/8/2023

1 µm
HV=80.0kV
Direct Mag: 10000x
Center for Biologic Imaging

8-AG treated aged central retina-IS

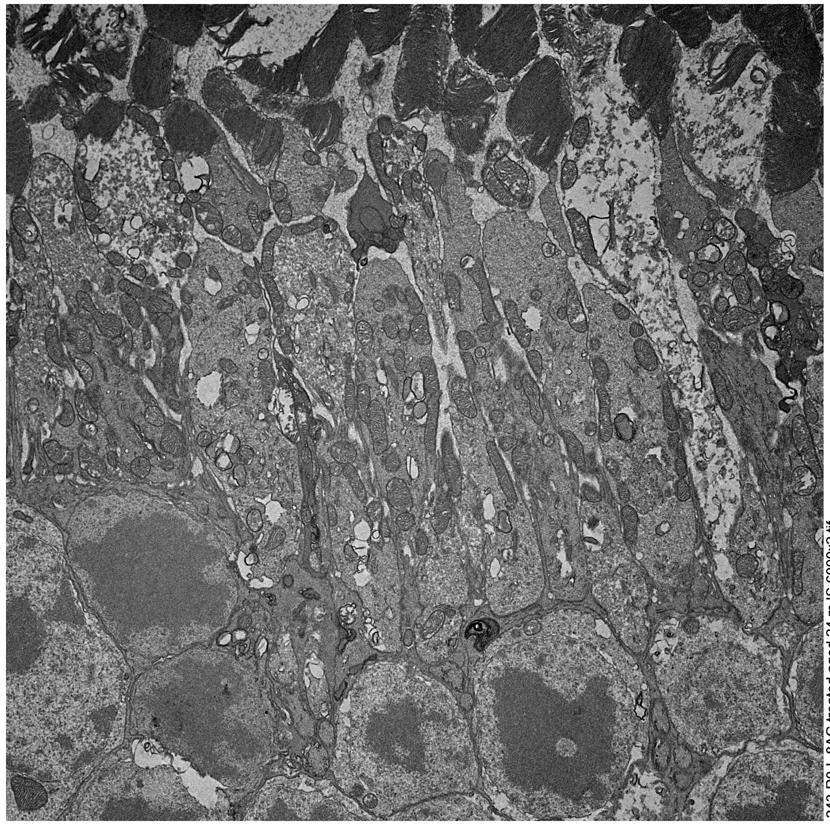

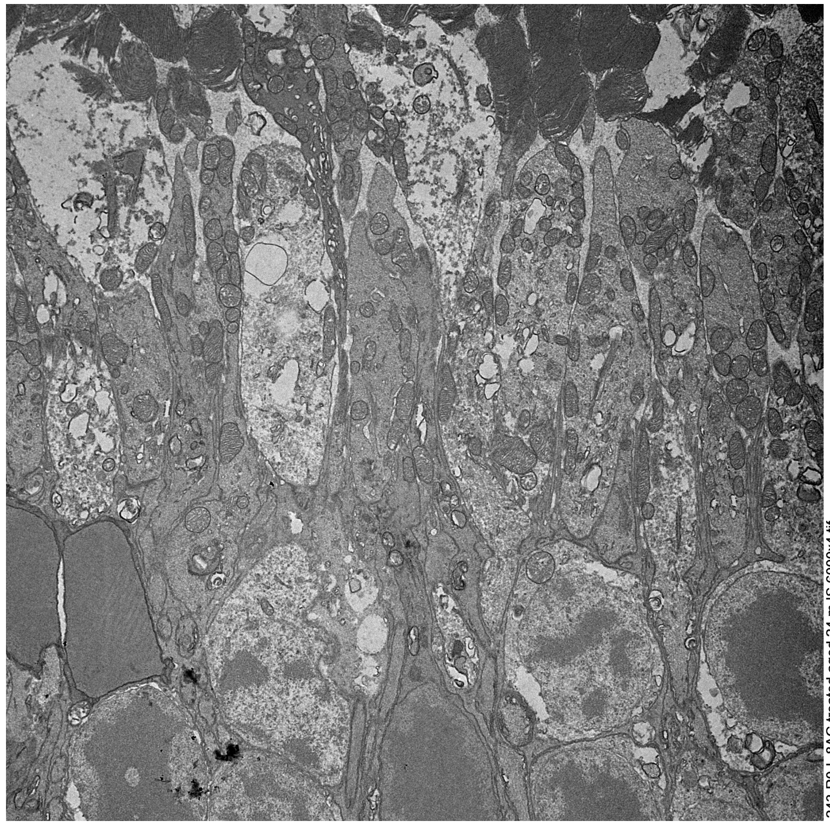

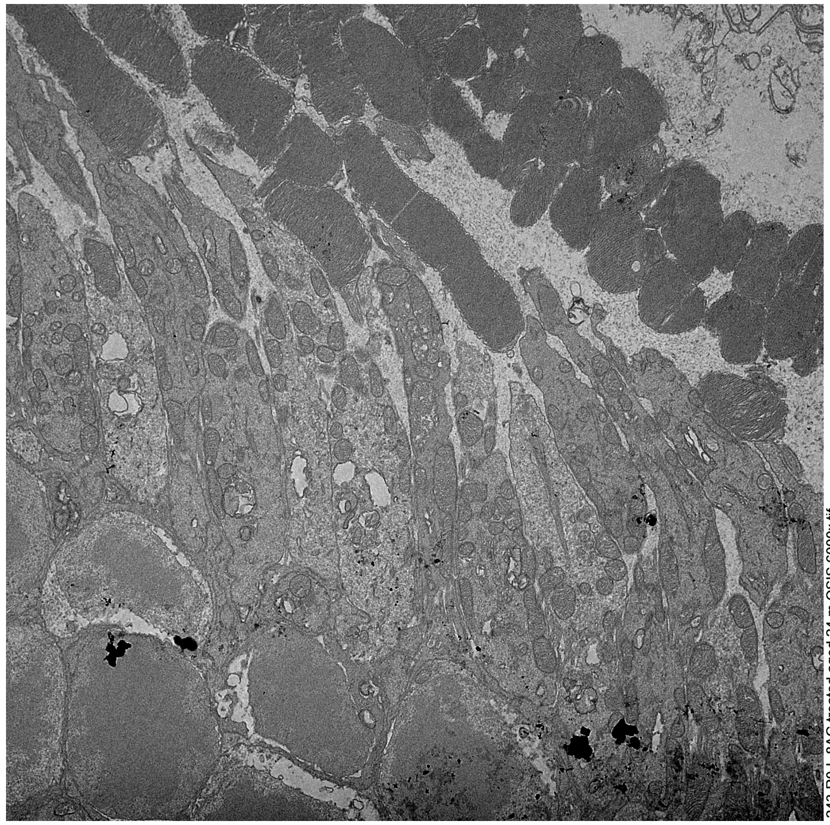

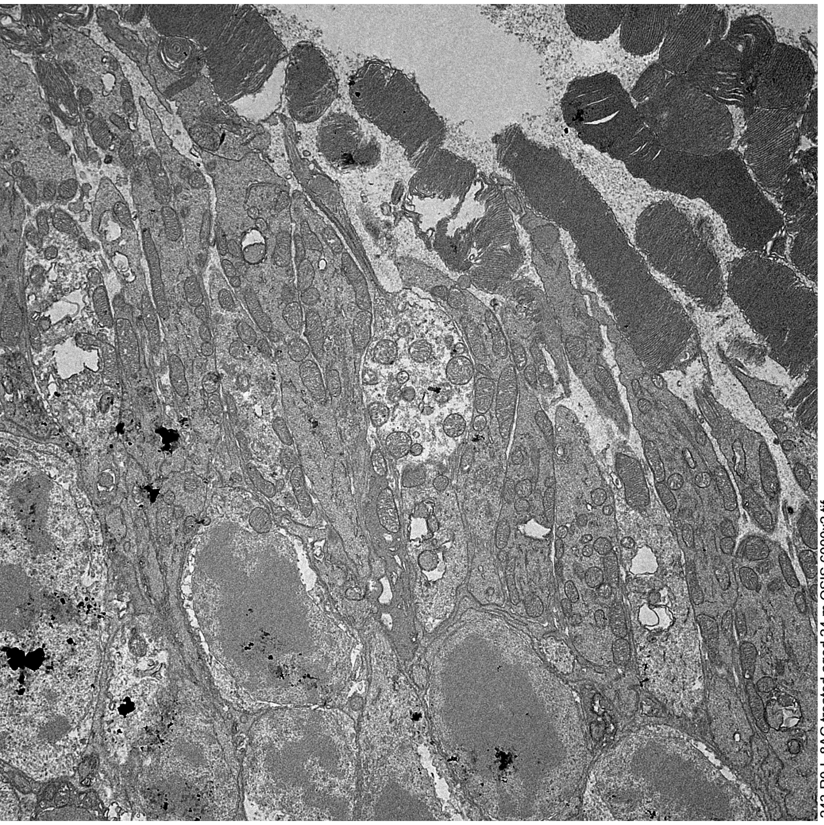

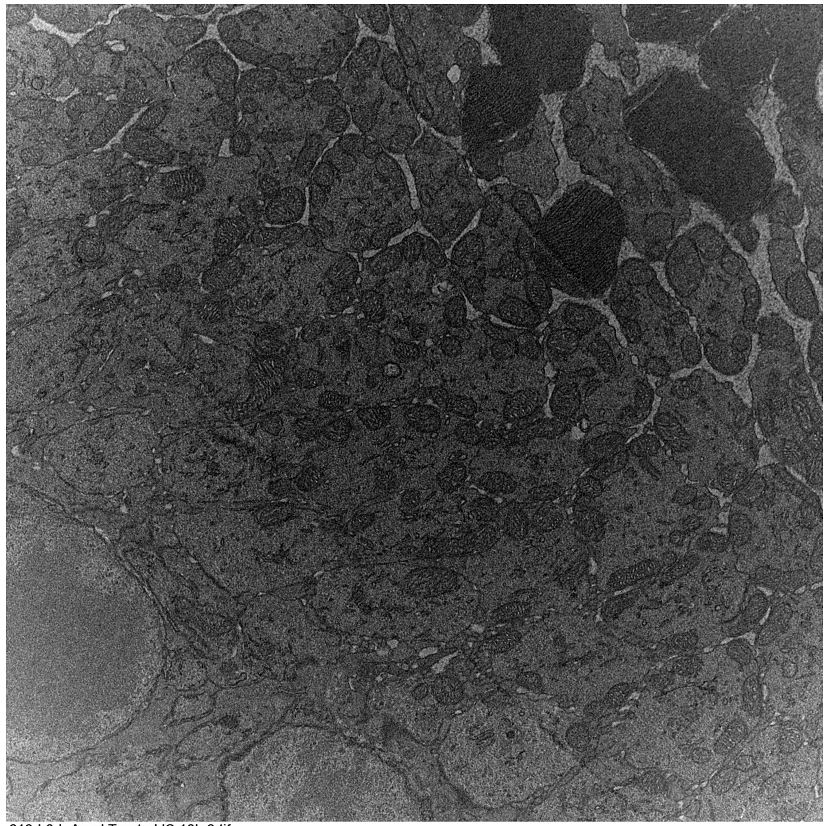

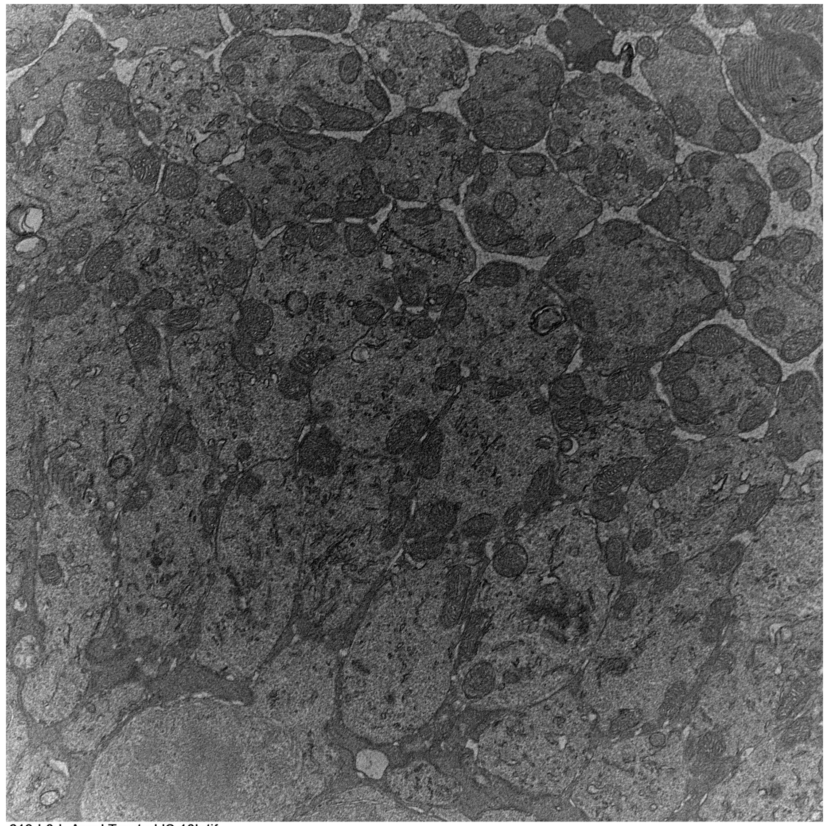

8-AG treated aged central retina-IS

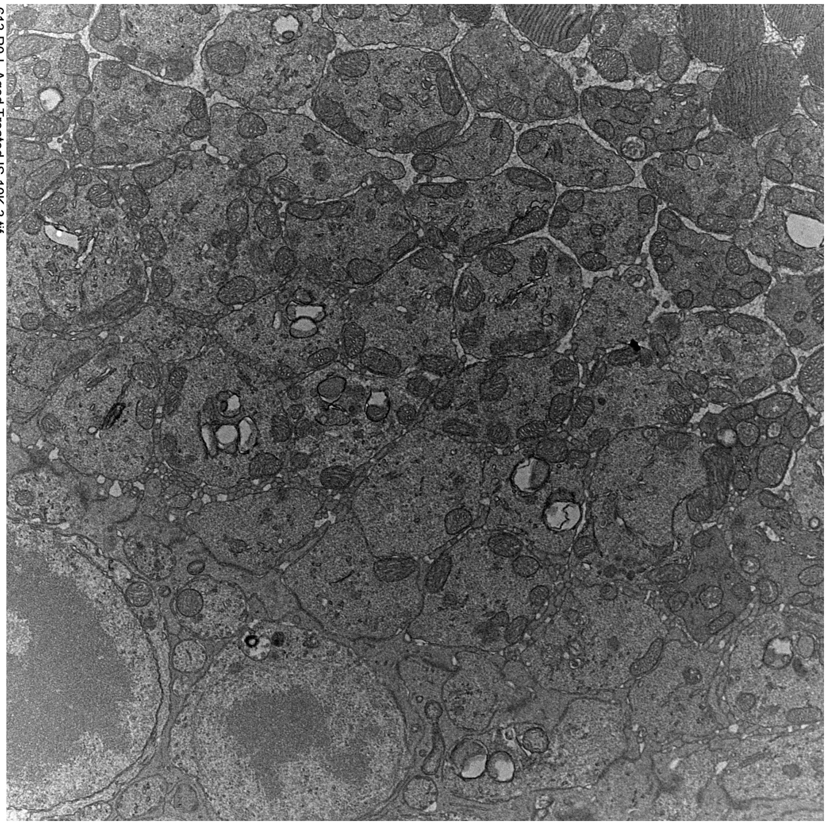

613-R0-L Aged Treated IS 10K-3.tif
613-R0-L Aged Treated
Print Mag: 11700x @ 7.0 in
15:23:24 4/28/2023

1 µm
HV=80.0kV
Direct Mag: 10000x
Center for Biologic Imaging

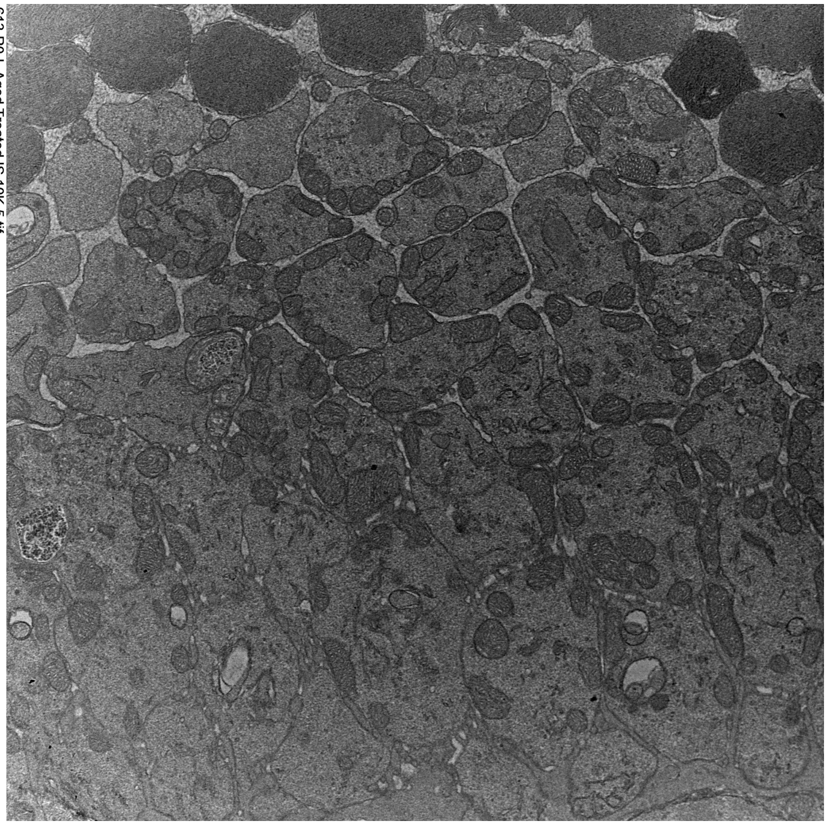

613-R0-L Aged Treated IS 10K-5.tif
613-R0-L Aged Treated
Print Mag: 11700x @ 7.0 in
15:47:19 4/28/2023

1 µm
HV=80.0kV
Direct Mag: 10000x
Center for Biologic Imaging

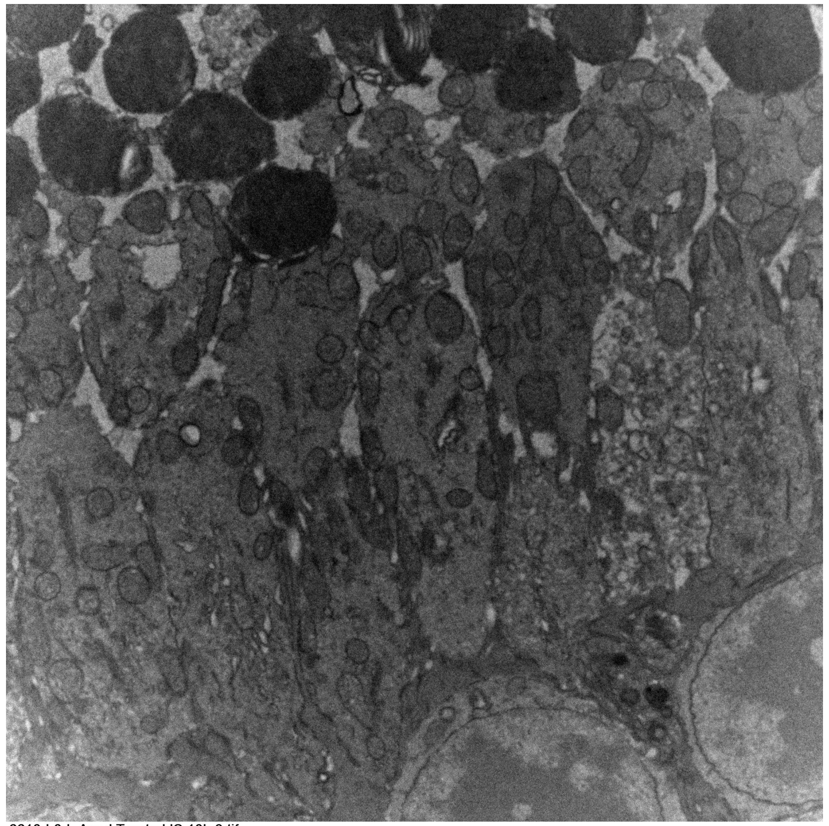

6610-L0-L Aged Treated IS 10k-3.tif
611-R0-R Aged Untreated
Print Mag: 11700x @ 7.0 in
12:30:53 4/27/2023

1 µm
HV=80.0kV
Direct Mag: 10000x
Center for Biologic Imaging

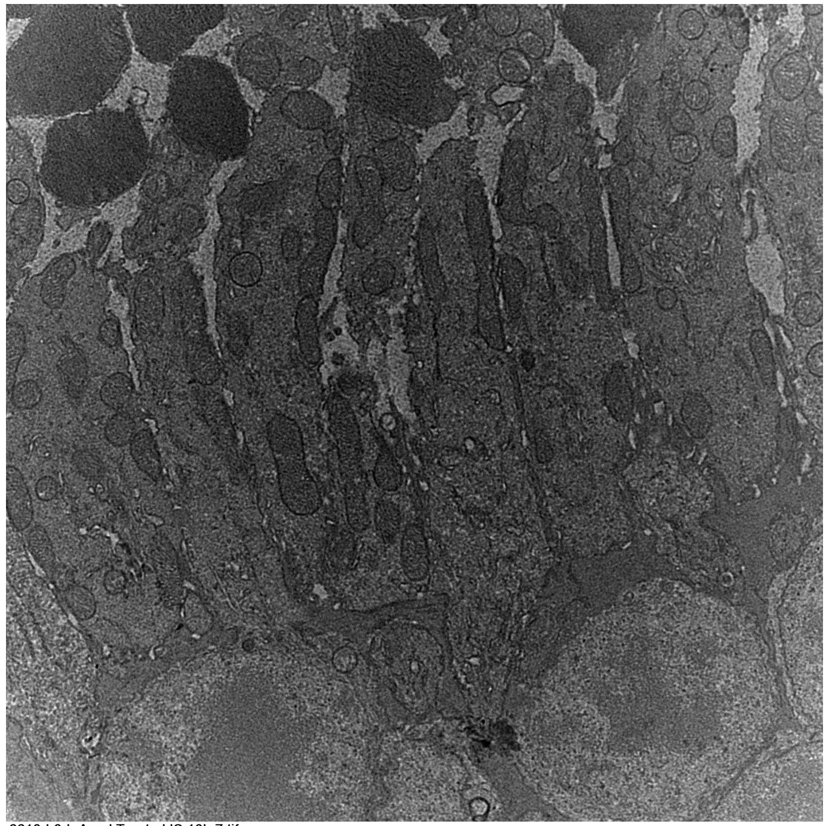

6610-L0-L Aged Treated IS 10k-7.tif
611-R0-R Aged Untreated
Print Mag: 11700x @ 7.0 in
13:04:14 4/27/2023

1 µm
HV=80.0kV
Direct Mag: 10000x
Center for Biologic Imaging

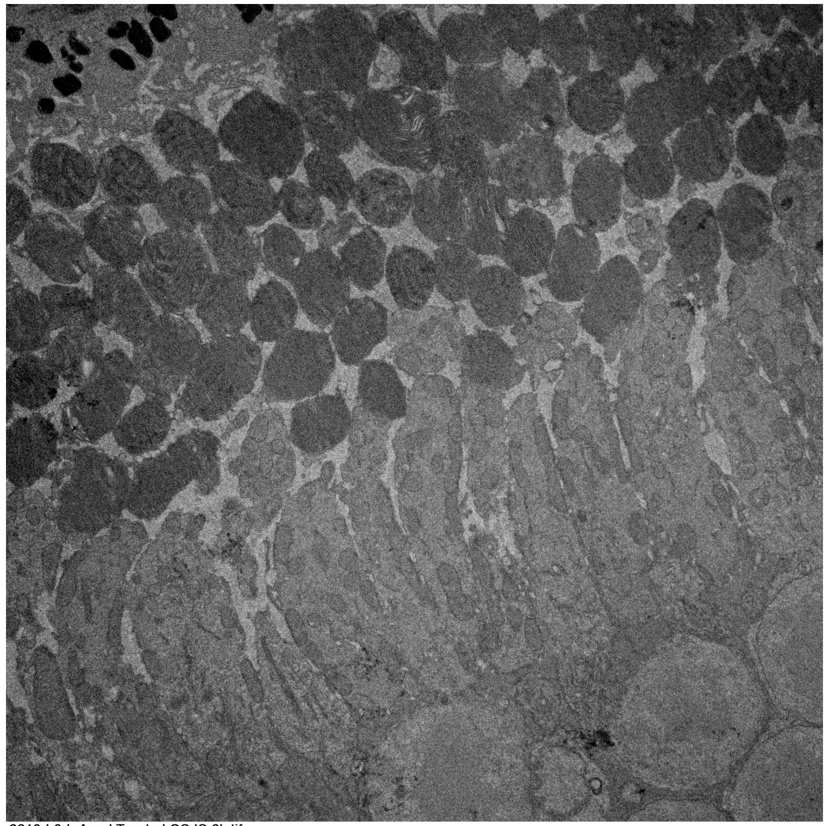

6610-L0-L Aged Treated OS IS 6k.tif
611-R0-R Aged Untreated
Print Mag: 7020x @ 7.0 in
13:00:34 4/27/2023

2 µm
HV=80.0kV
Direct Mag: 6000x
Center for Biologic Imaging

8-AG treated aged central retina-RPE

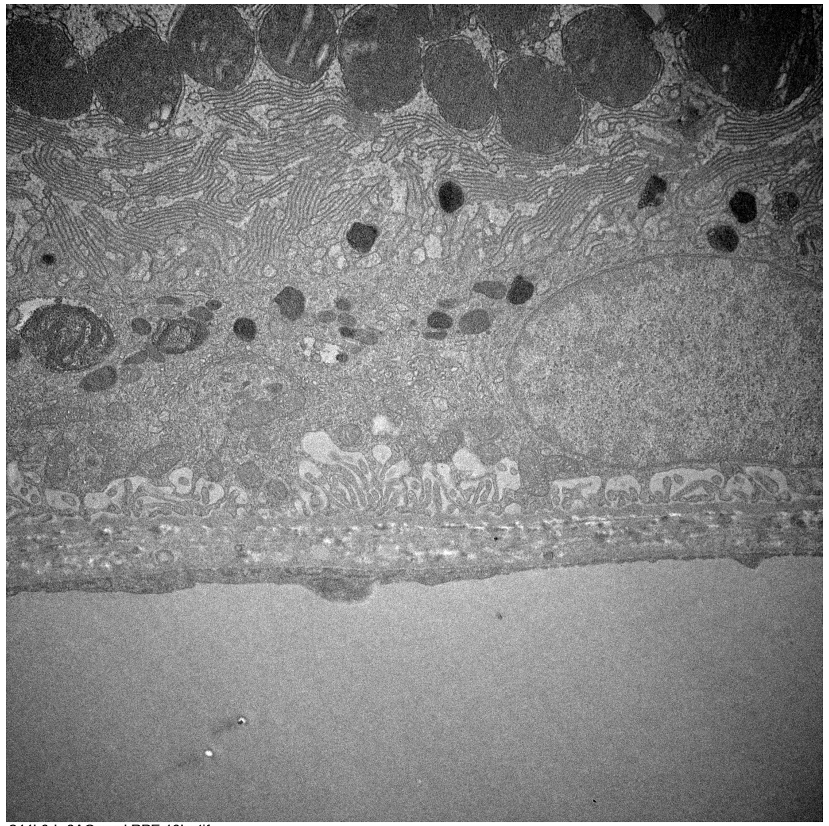

244L0-L-8AGaged RPE 10kx.tif
244-L0-L
aged-8AG treated RPE10kx
Print Mag: 11700x @ 7.0 in
10:35:56 3/29/2023
1 µm
HV=80.0kV
Direct Mag: 10000x
Center for Biologic Imaging

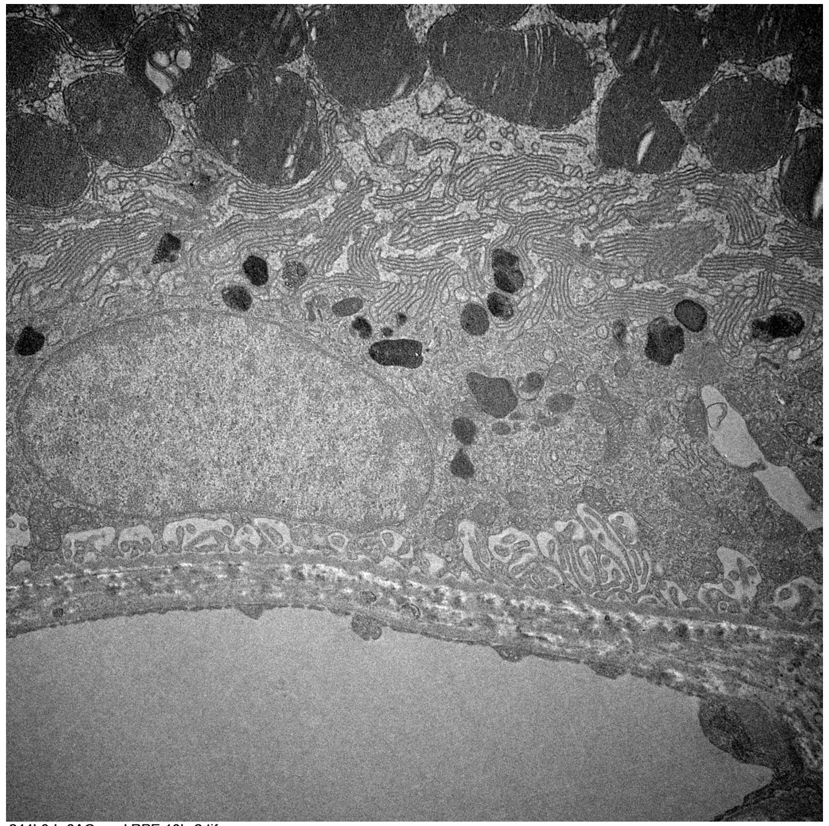

244L0-L-8AGaged RPE 10kx2.tif
244-L0-L
aged-8AG treated RPE10kx2
Print Mag: 11700x @ 7.0 in
10:38:39 3/29/2023
1 µm
HV=80.0kV
Direct Mag: 10000x
Center for Biologic Imaging

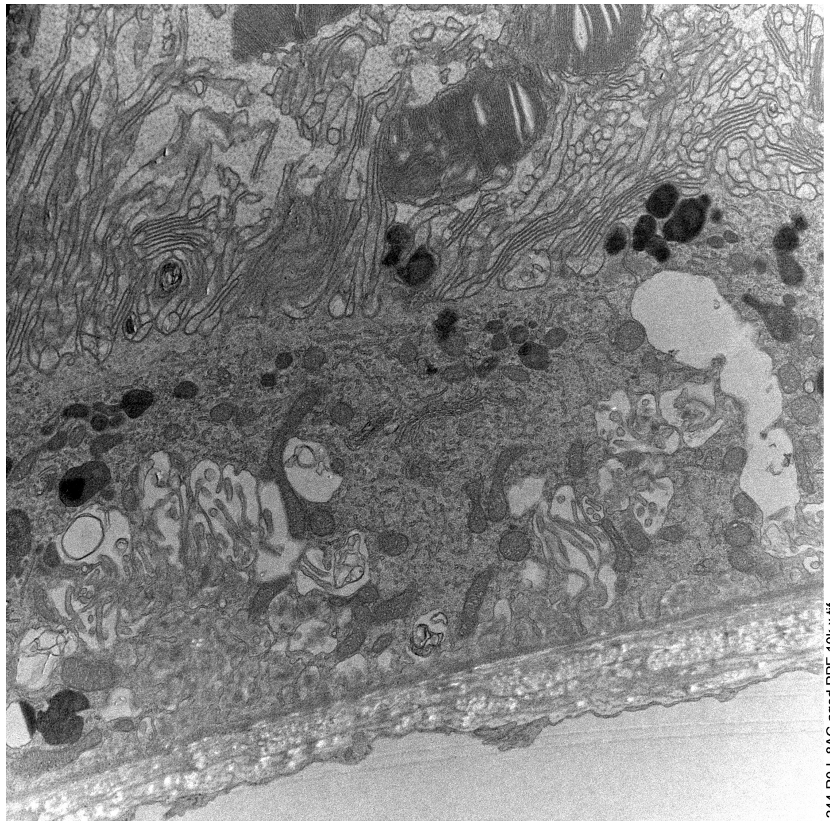

1 µm
HV=80.0kV
Direct Mag: 10000x
Center for Biologic Imaging
244-R0-L 8AG aged RPE 10k x.tif
244-L0-L
aged-8AG treatedRPE 10kx
Print Mag: 11700x @ 7.0 in
16:22:11 3/29/2023

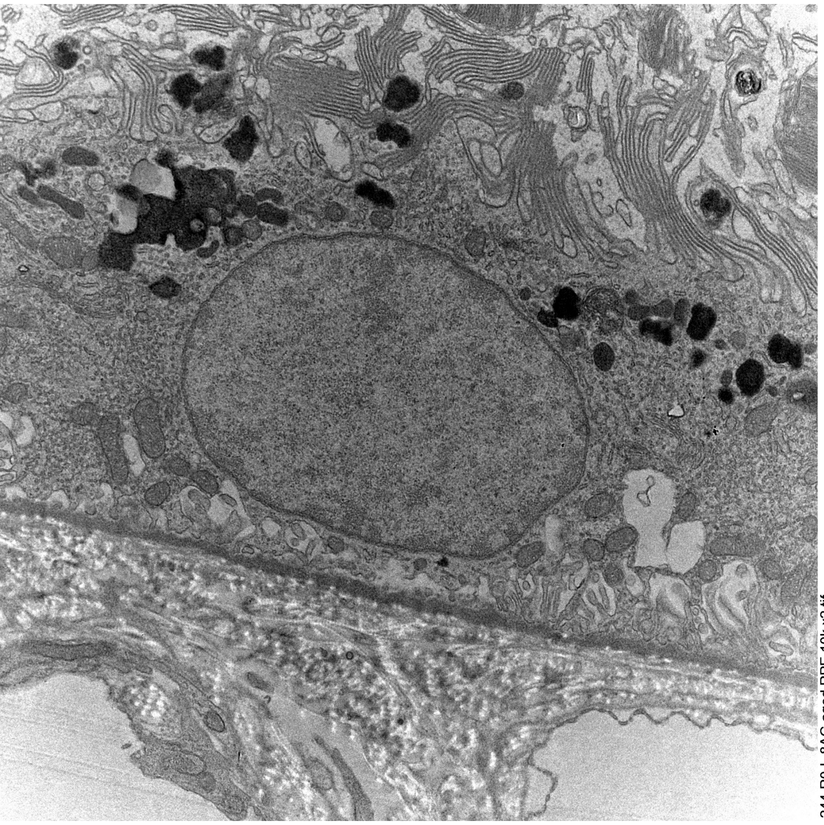

1 µm
HV=80.0kV
Direct Mag: 10000x
Center for Biologic Imaging
244-R0-L 8AG aged RPE 10k x2.tif
244-L0-L
aged-8AG treatedRPE 10kx2
Print Mag: 11700x @ 7.0 in
16:26:15 3/29/2023

243L0-L-8AG-aged-RPE-10000x.tif
243L0-L Aged treated
RPE OS10000x
Print Mag: 11700x @ 7.0 in
13:37:15 3/8/2023
1 µm
HV=80.0kV
Direct Mag: 10000x
Center for Biologic Imaging

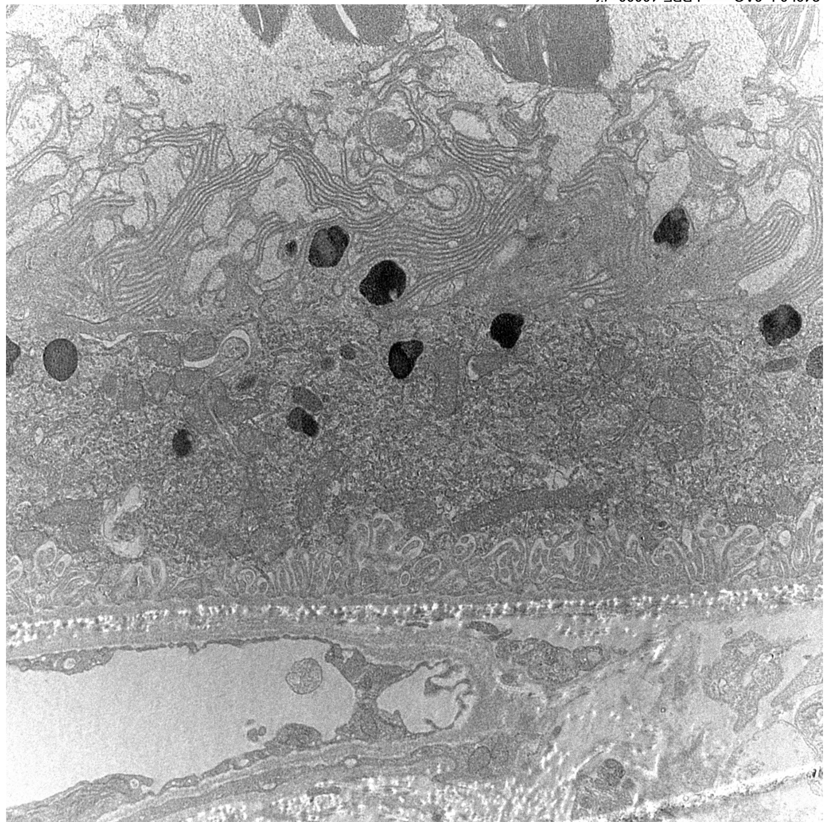

243L0-L-8AG-aged-RPE-10000x7.tif
243L0-L aged 8AG treated
RPE10000x7
Print Mag: 11700x @ 7.0 in
15:37:46 3/8/2023
1 µm
HV=80.0kV
Direct Mag: 10000x
Center for Biologic Imaging

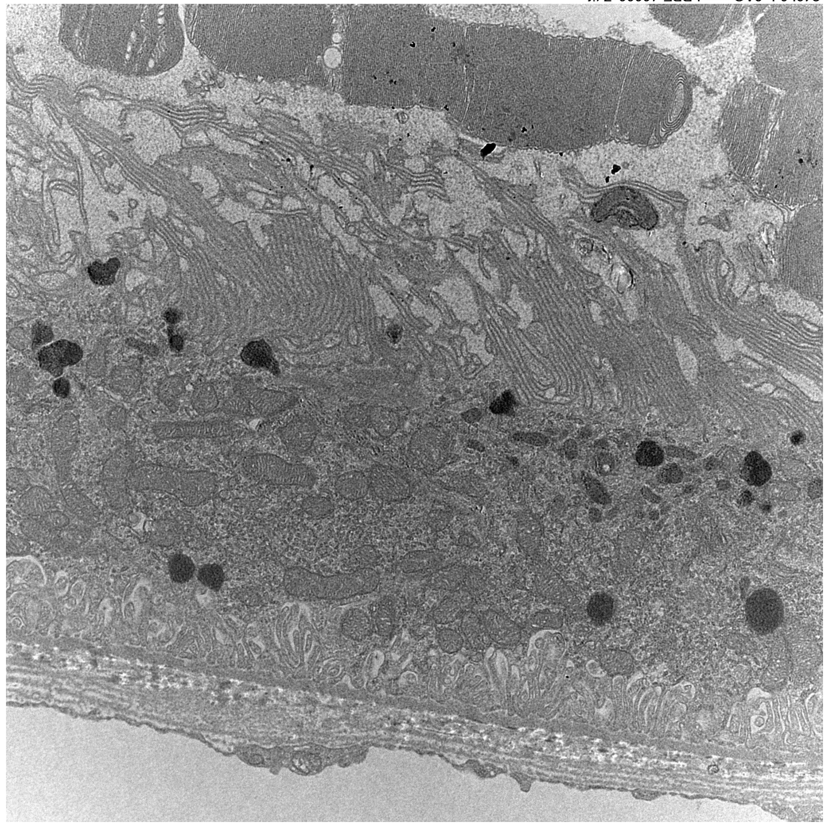

8-AG treated aged central retina-RPE

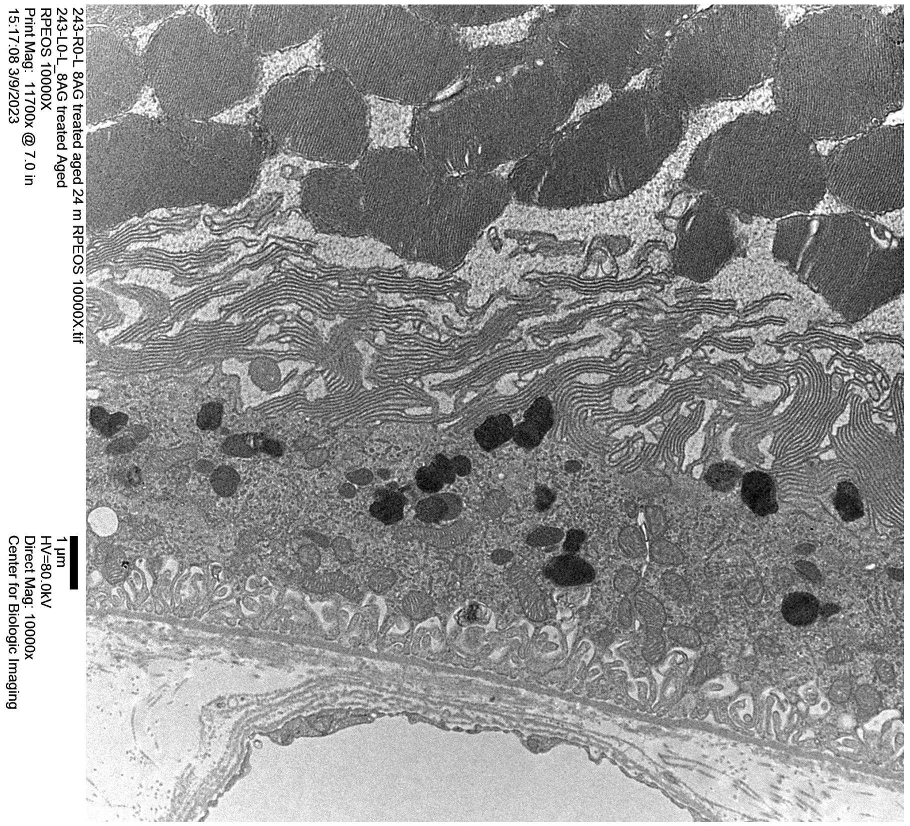

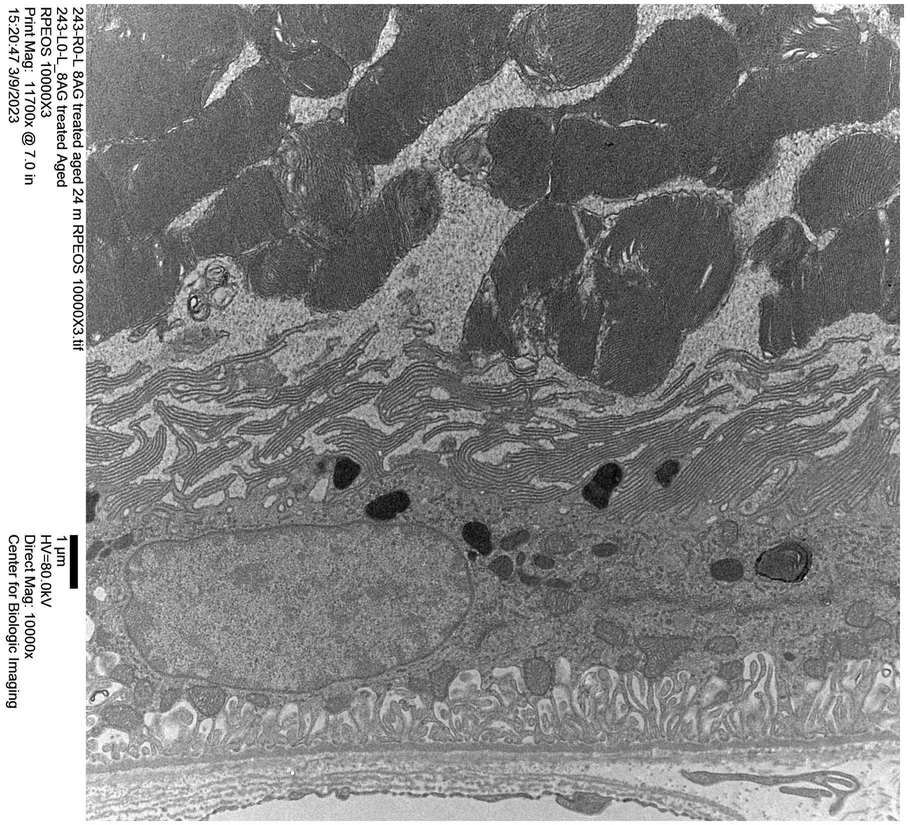

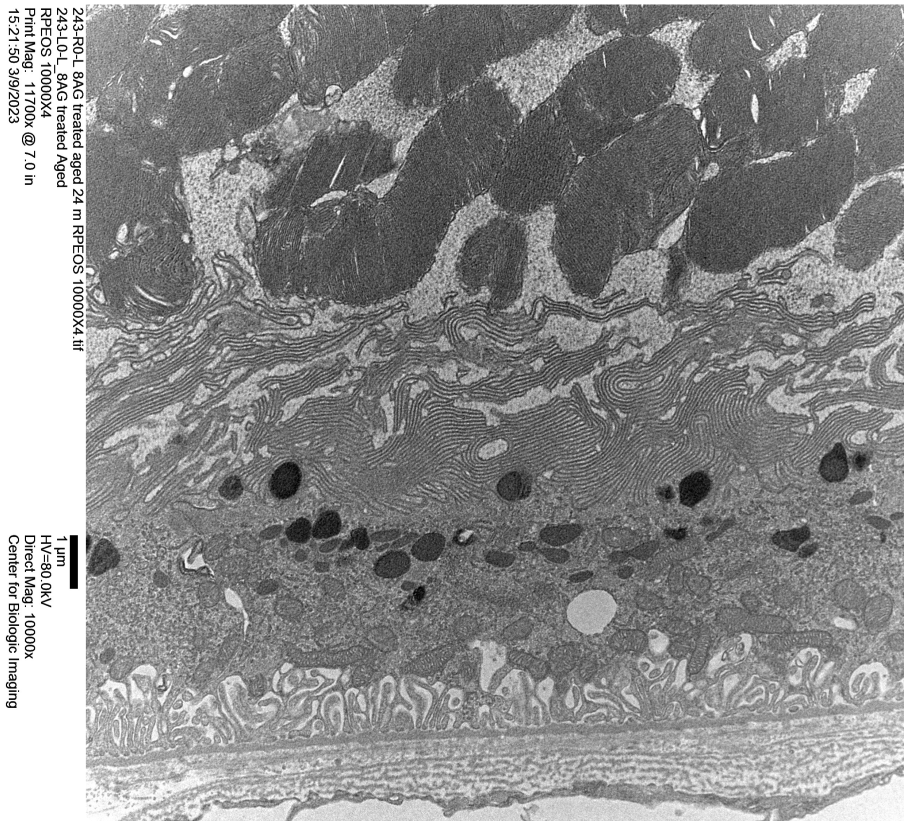

8-AG treated aged central retina-RPE

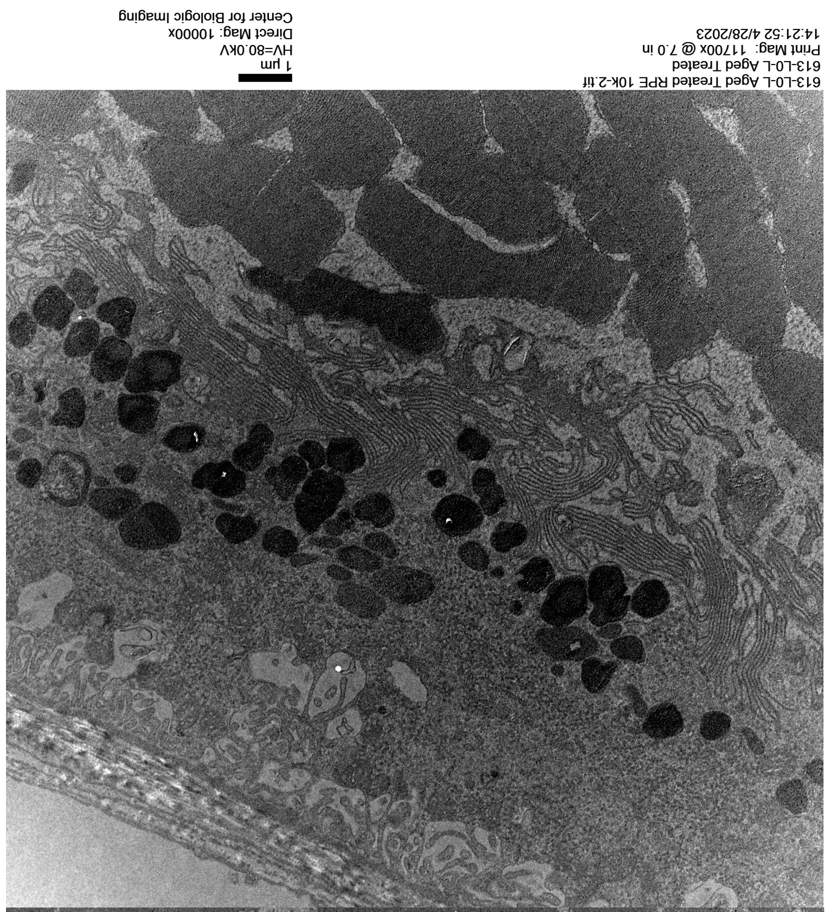

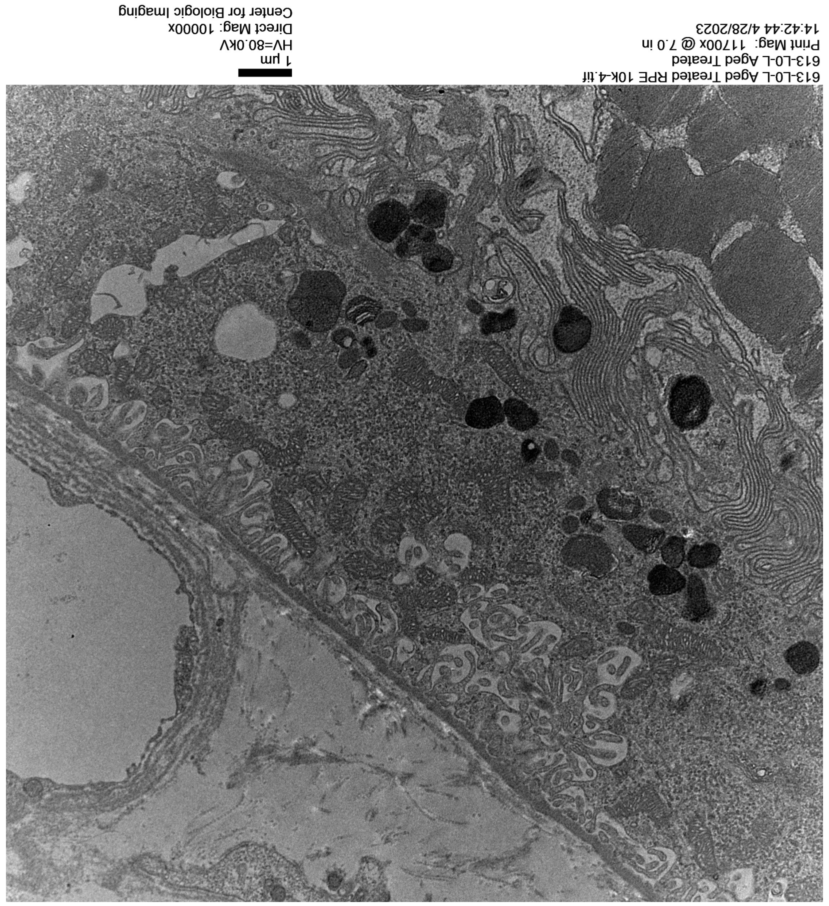

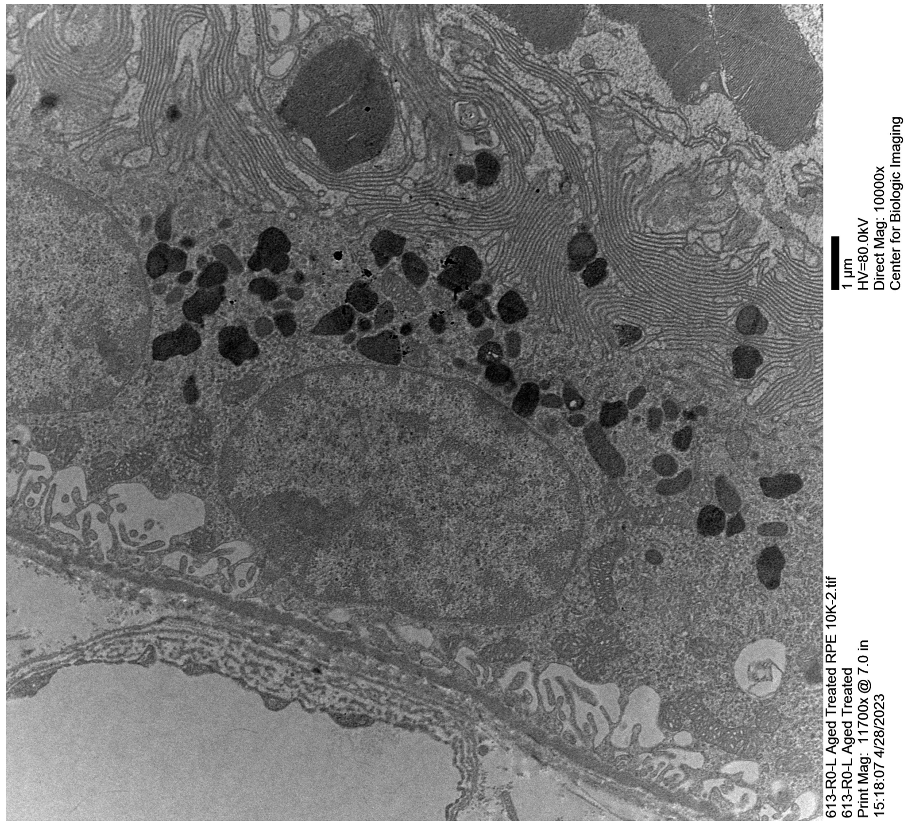

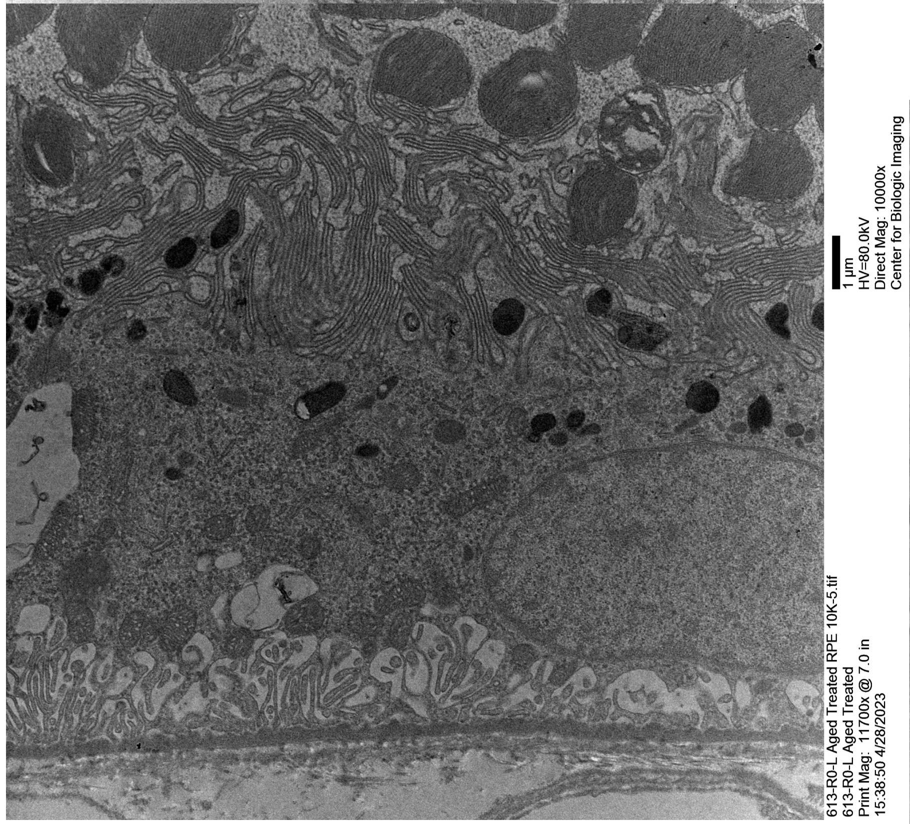

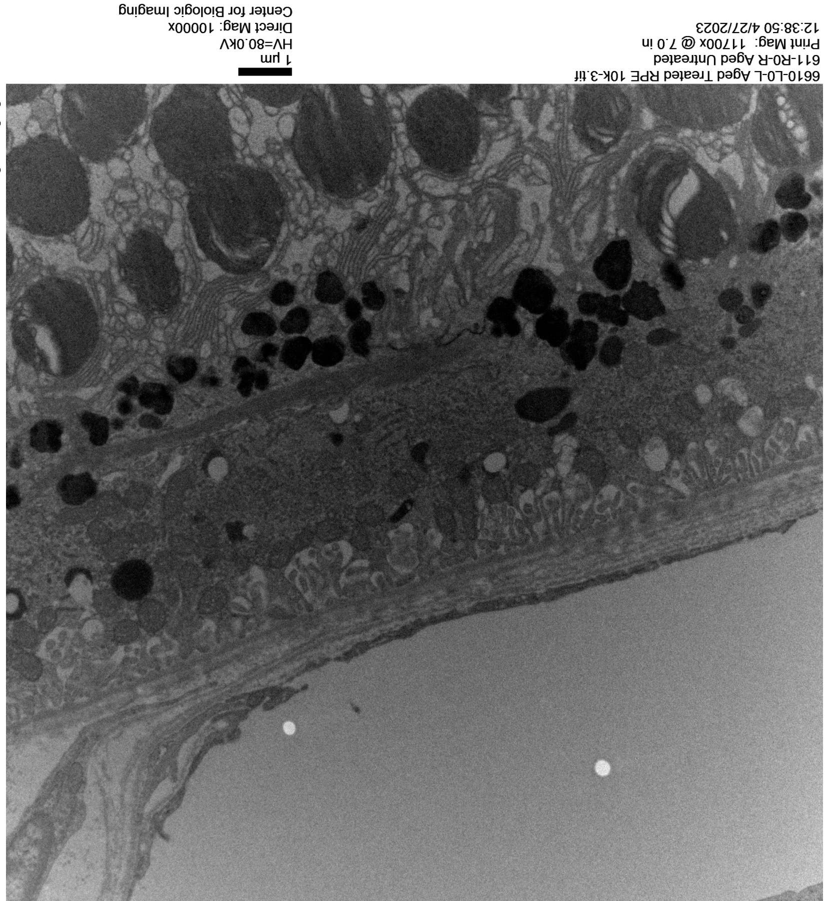

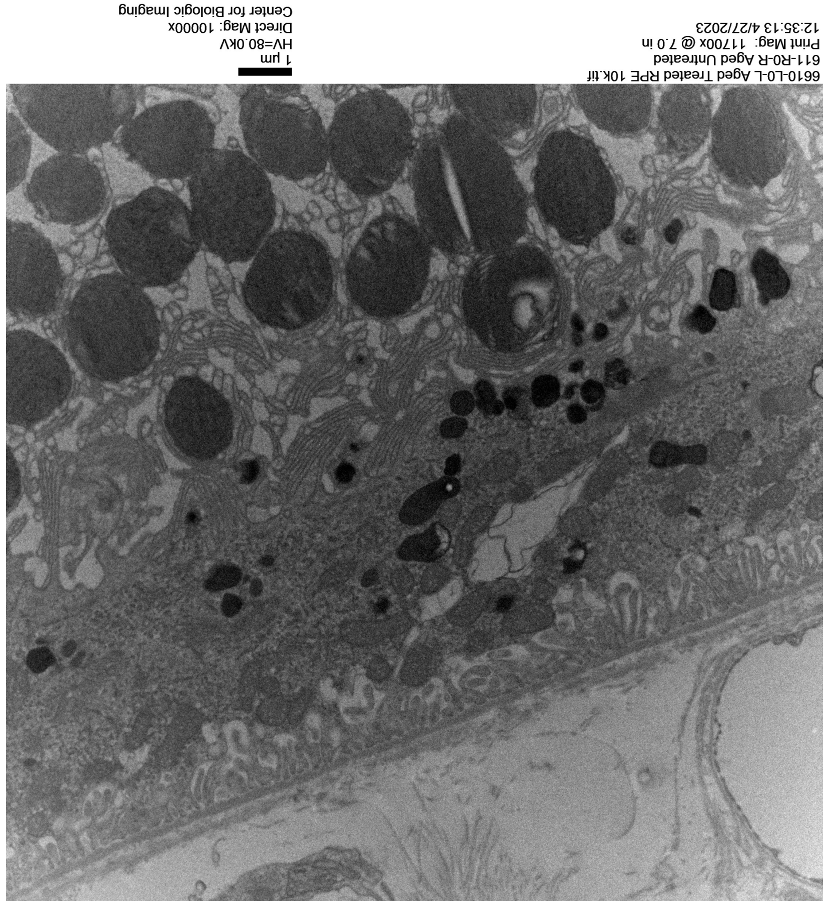

8-AG treated aged central-RPE

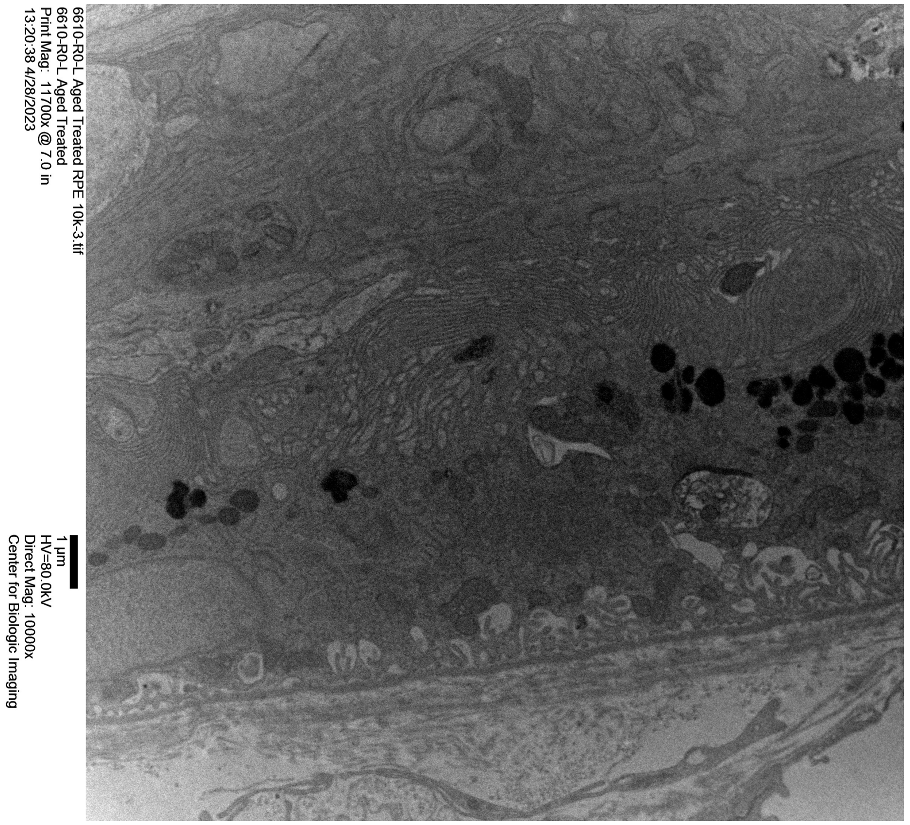

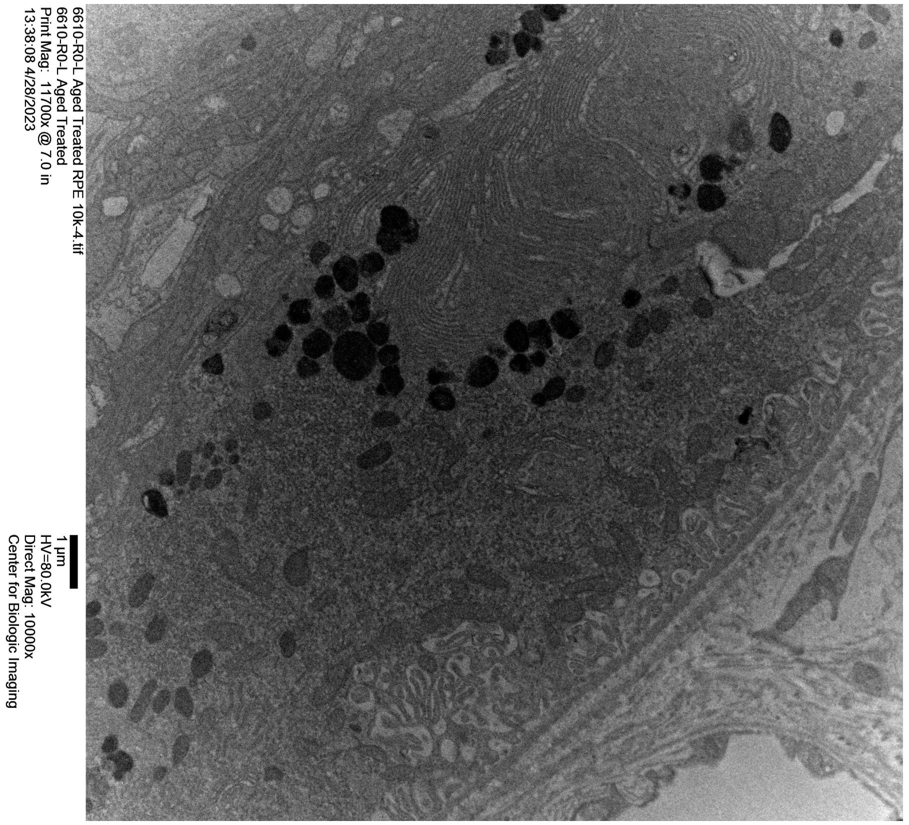

8-AG treated aged central retina-RPE-OS

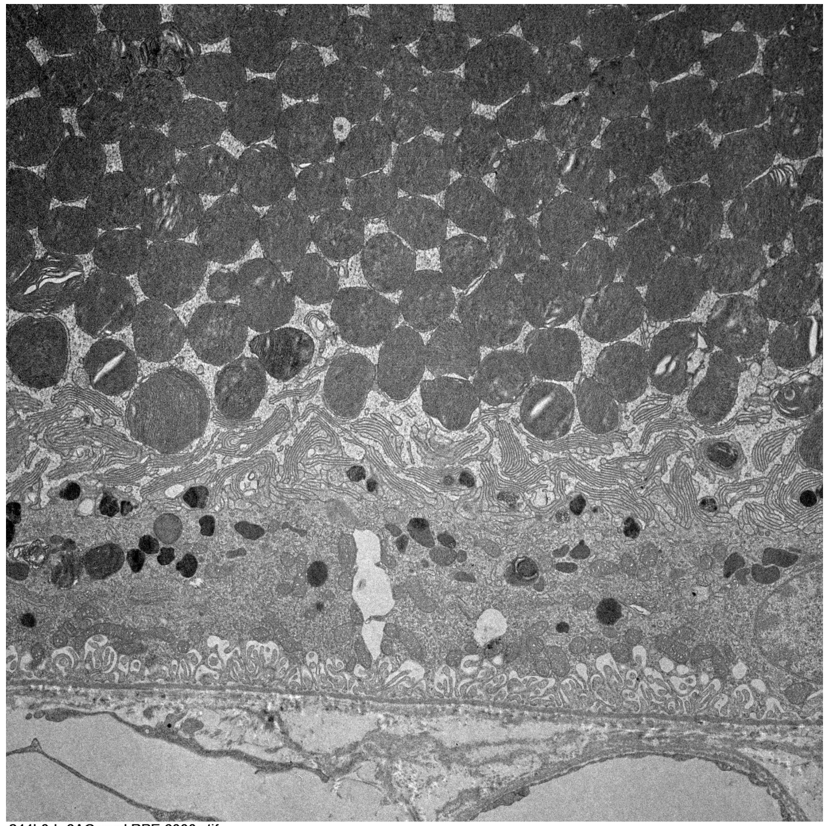

244L0-L-8AGaged RPE 6000x.tif
244-L0-L
aged-8AG treated RPE6000x
Print Mag: 7020x @ 7.0 in
09:52:42 3/29/2023

2 µm
HV=80.0kV
Direct Mag: 6000x
Center for Biologic Imaging

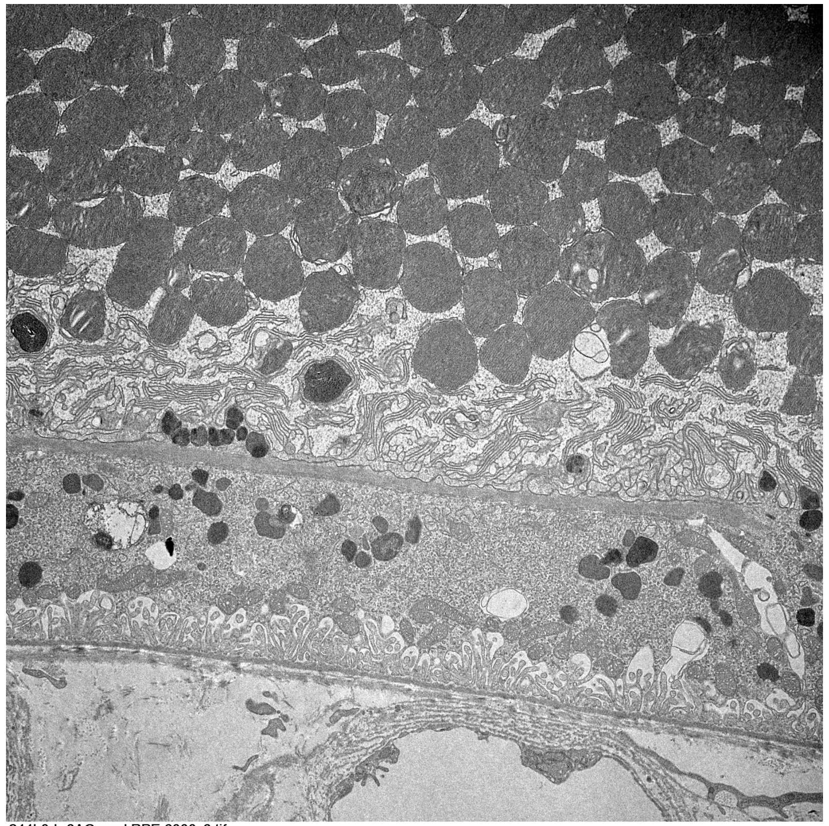

244L0-L-8AGaged RPE 6000x6.tif
244-L0-L
aged-8AG treated RPE6000x6
Print Mag: 7020x @ 7.0 in
10:04:19 3/29/2023

2 µm
HV=80.0kV
Direct Mag: 6000x
Center for Biologic Imaging

243L0-L-8AG-aged-RPE-6000x.tif
243L0-L Aged treated
RPE_OS6000x
Print Mag: 7020x @ 7.0 in
13:33:26 3/8/2023

2 µm
HV=80.0kV
Direct Mag: 6000x
Center for Biologic Imaging

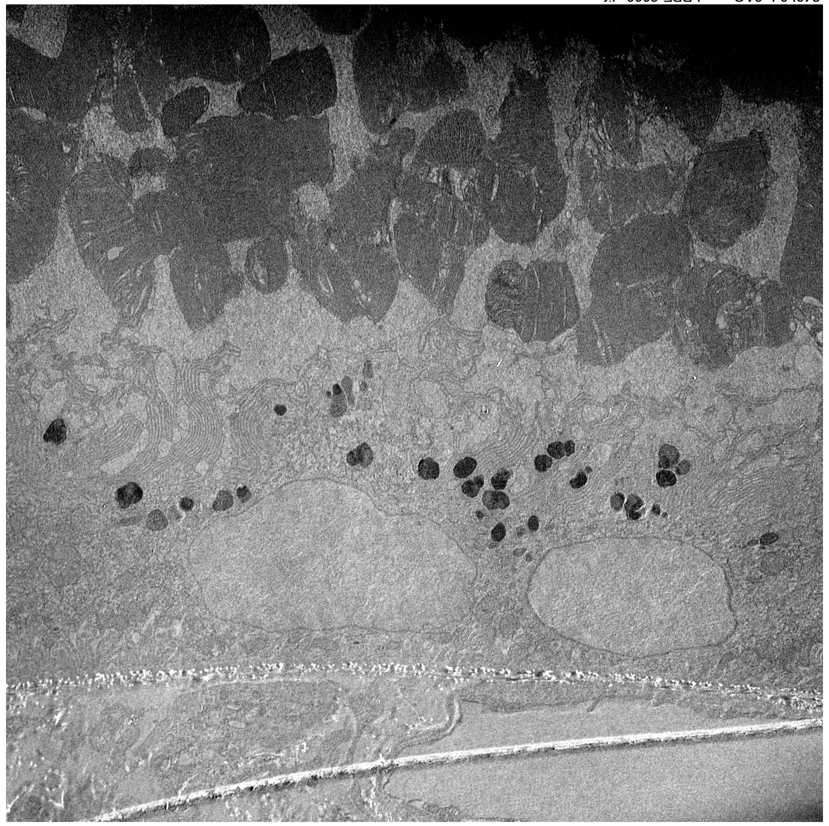

8-AG treated aged central retina-RPE-OS

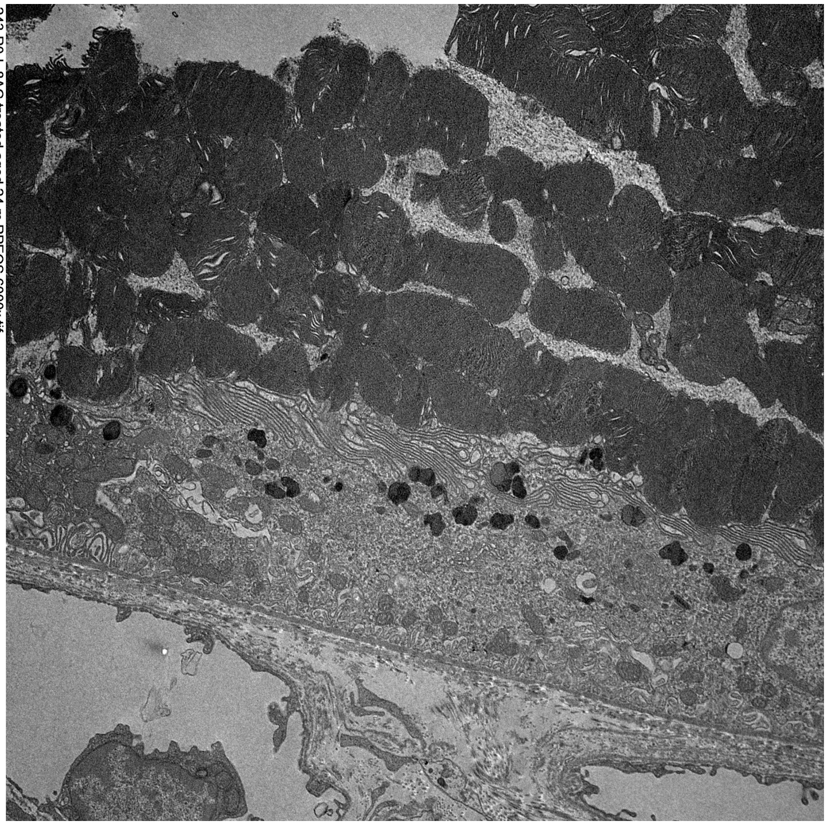

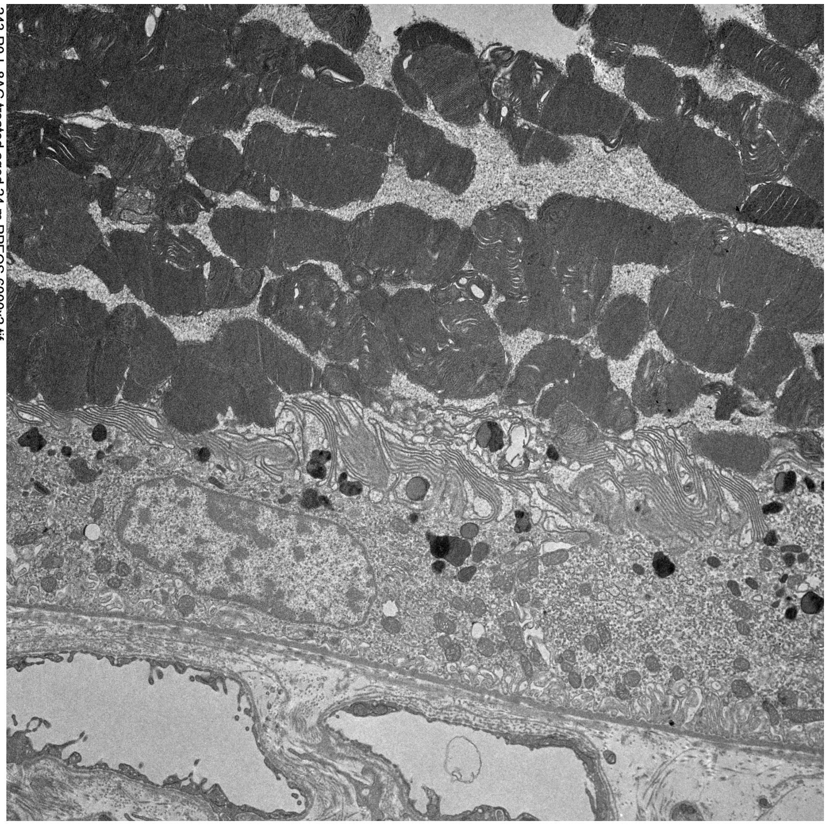

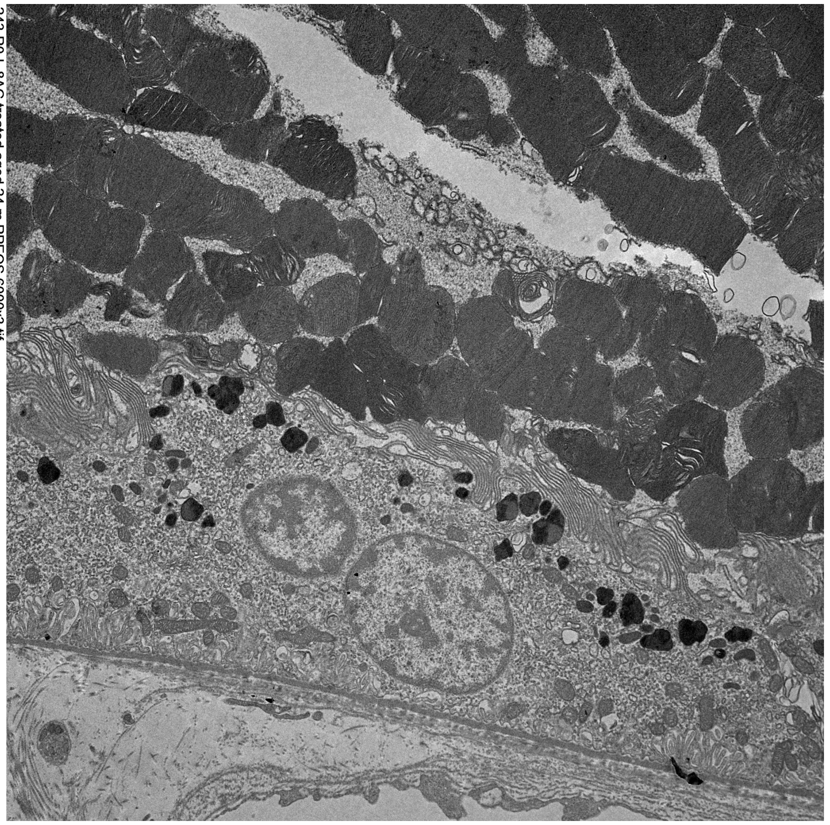

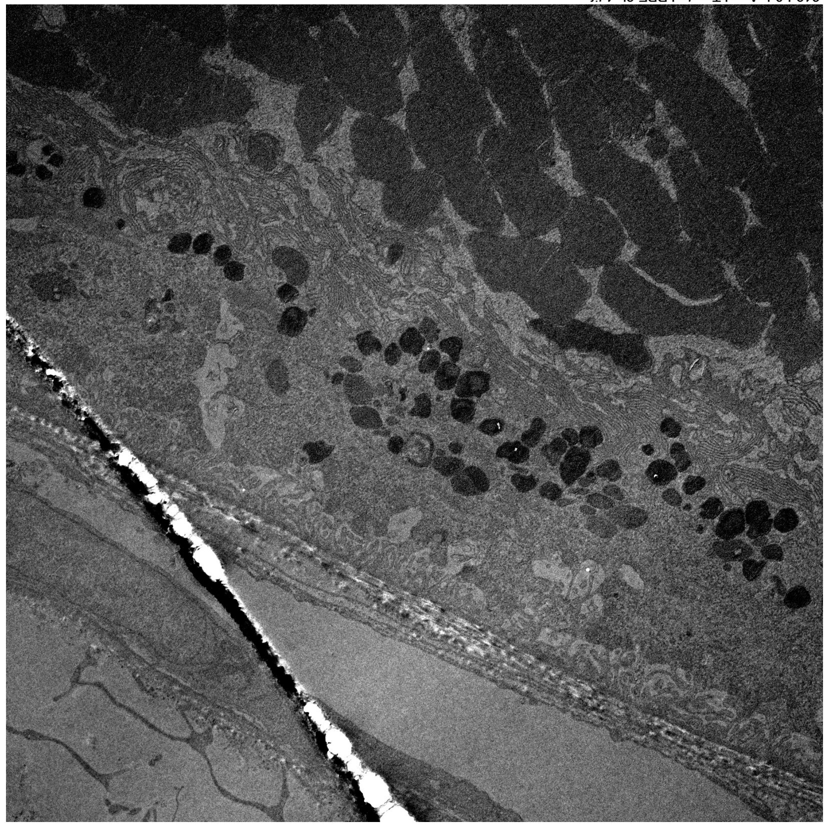

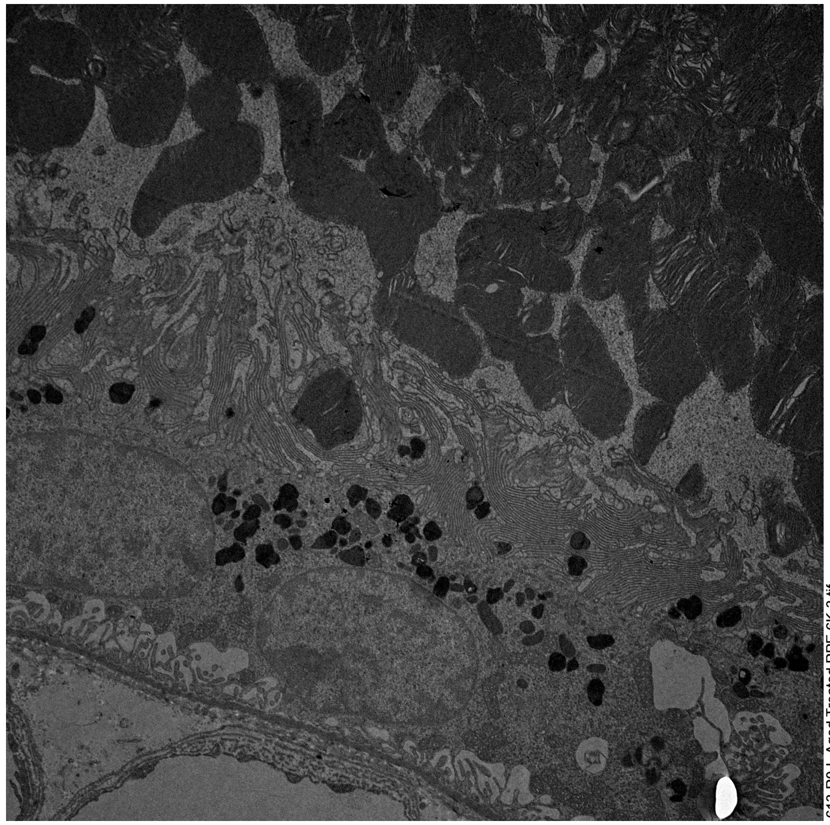

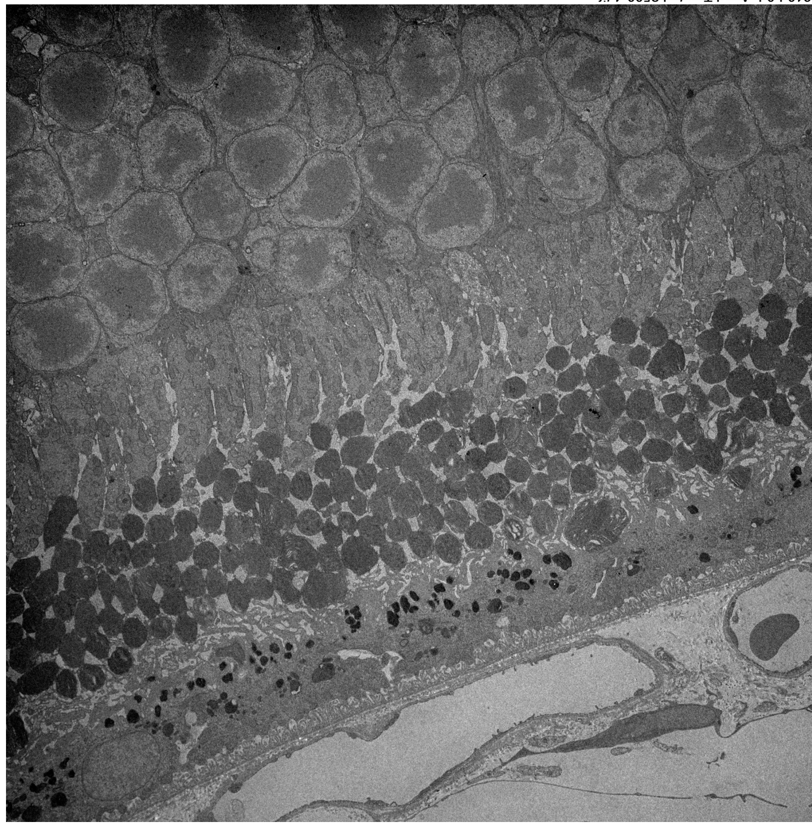

8-AG treated aged central-RPE-OS

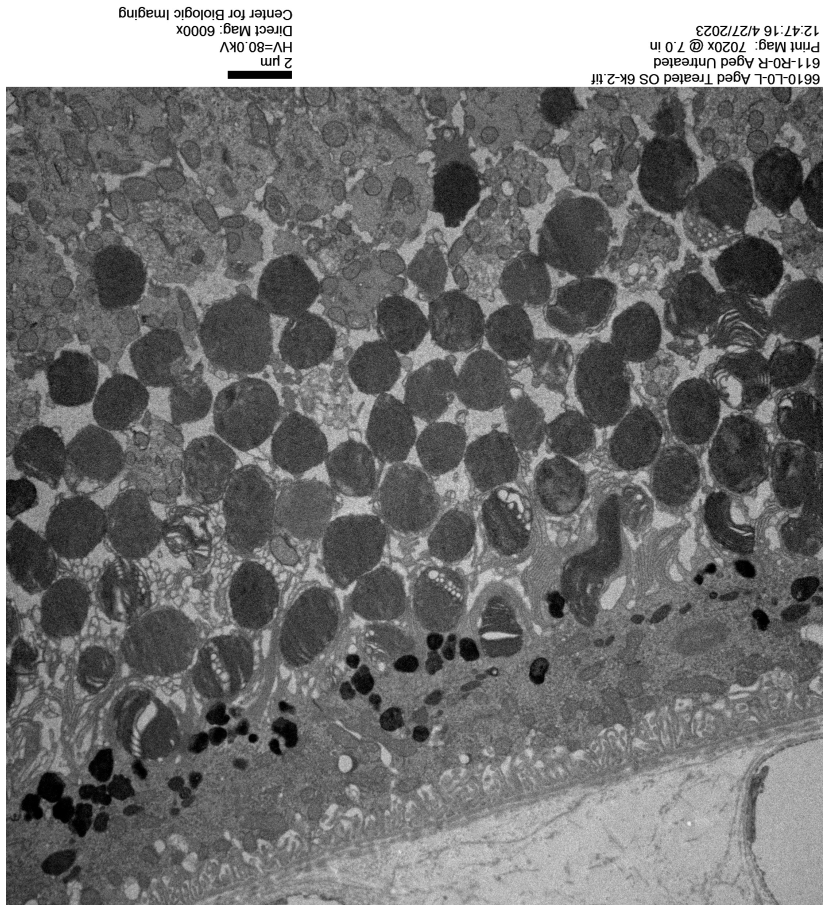

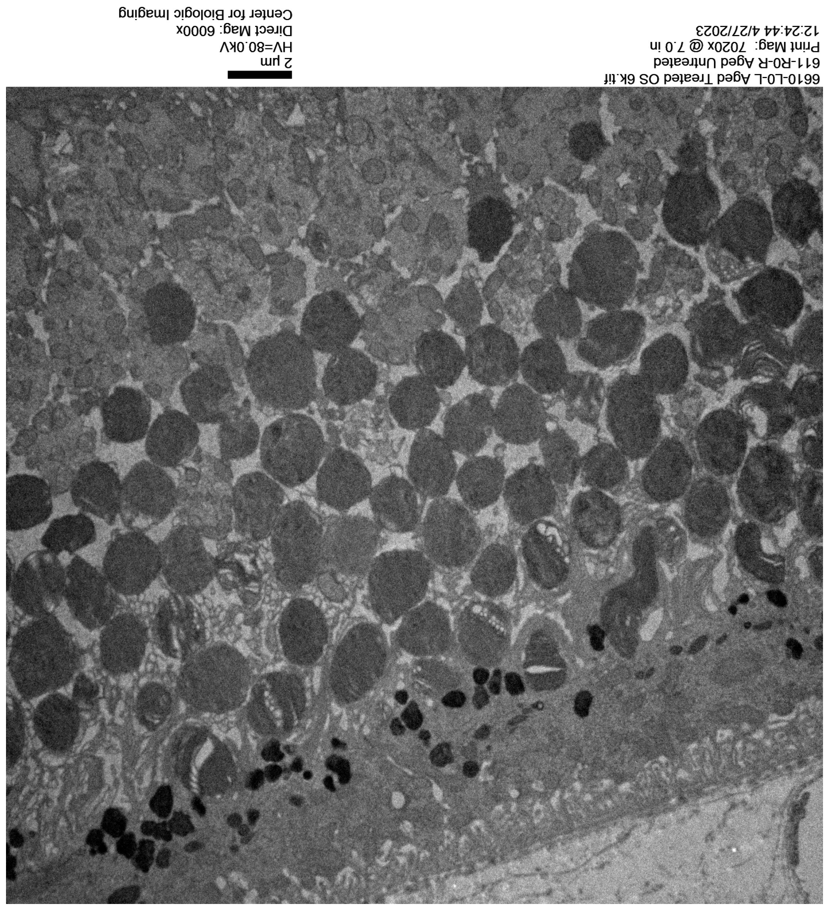

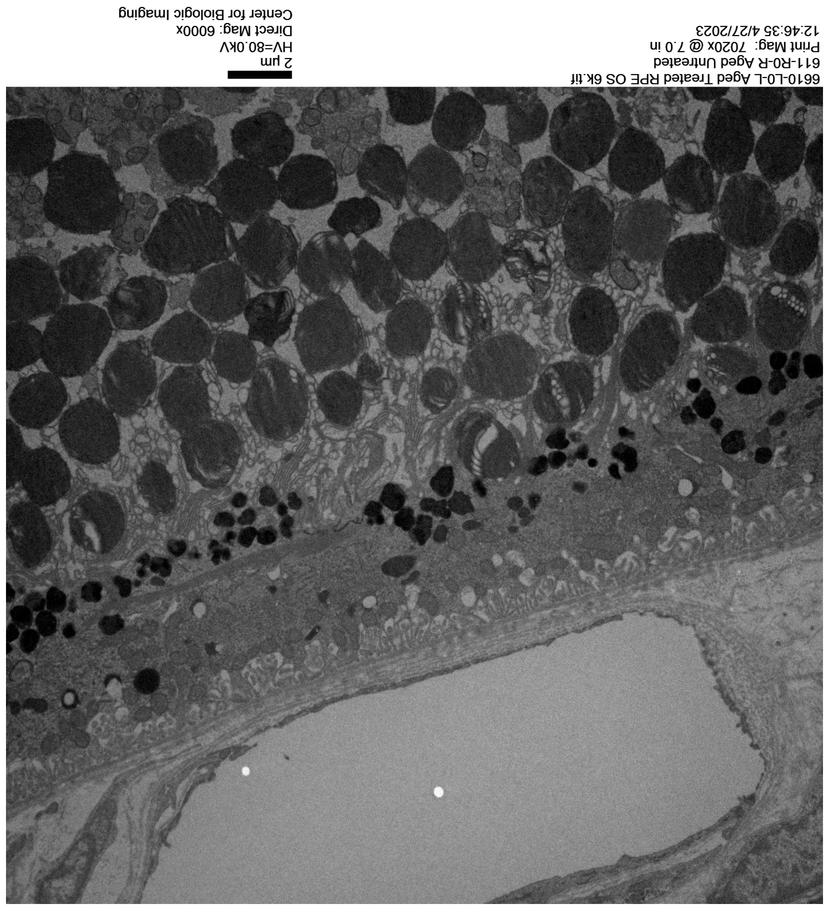

Supplement: Supplementary file 14 — Supplementary Data 12 [file 42003_2025_8242_MOESM14_ESM.pdf]
